# Transcriptomics Underlying Pulmonary Ozone Pathogenesis Regulated by Inflammatory Mediators in Mice

**DOI:** 10.3390/antiox10091489

**Published:** 2021-09-18

**Authors:** Hye-Youn Cho, Anne E. Jedlicka, Frederick H. Chang, Jacqui Marzec, Alison K. Bauer, Steven R. Kleeberger

**Affiliations:** 1 Immunity, Inflammation and Disease Laboratory, National Institute of Environmental Health Sciences, National Institutes of Health, Durham, NC 27709, USA; fhchang@sas.upenn.edu (F.H.C.); marzec@niehs.nih.gov (J.M.); alison.bauer@cuanschutz.edu (A.K.B.); kleeber1@niehs.nih.gov (S.R.K.); 2 Department of Molecular Biology and Immunology, Bloomberg School of Public Health, Johns Hopkins University, Baltimore, MD 21205, USA; ajedlic1@jhu.edu; 3 School of Arts and Sciences, University of Pennsylvania, Philadelphia, PA 19104, USA; 4 Department of Environmental and Occupational Health, Colorado School of Public Health, University of Colorado Anschutz, Aurora, CO 80045, USA

**Keywords:** ozone, mice, lung, microarray, TNF receptor, NF-κB, IL-6

## Abstract

Ozone (O_3_) is the predominant oxidant air pollutant associated with airway inflammation, lung dysfunction, and the worsening of preexisting respiratory diseases. We previously demonstrated the injurious roles of pulmonary immune receptors, tumor necrosis factor receptor (TNFR), and toll-like receptor 4, as well as a transcription factor NF-κB, in response to O_3_ in mice. In the current study, we profiled time-dependent and TNFR- and NF-κB-regulated lung transcriptome changes by subacute O_3_ to illuminate the underlying molecular events and downstream targets. Mice lacking *Tnfr1*/*Tnfr2* (*Tnfr^-/-^*) or *Nfkb1* (*Nfkb1^-/-^*) were exposed to air or O_3_. Lung RNAs were prepared for cDNA microarray analyses, and downstream and upstream mechanisms were predicted by pathway analyses of the enriched genes. O_3_ significantly altered the genes involved in inflammation and redox (24 h), cholesterol biosynthesis and vaso-occlusion (48 h), and cell cycle and DNA repair (48–72 h). Transforming growth factor-β1 was a predicted upstream regulator. Lack of *Tnfr* suppressed the immune cell proliferation and lipid-related processes and heightened epithelial cell integrity, and *Nfkb1* deficiency markedly suppressed lung cell cycle progress during O_3_ exposure. Common differentially regulated genes by TNFR and NF-κB1 (e.g., *Casp8*, *Il6*, and *Edn1*) were predicted to protect the lungs from cell death, connective tissue injury, and inflammation. *Il6*-deficient mice were susceptible to O_3_-induced protein hyperpermeability, indicating its defensive role, while *Tnf*-deficient mice were resistant to overall lung injury caused by O_3_. The results elucidated transcriptome dynamics and provided new insights into the molecular mechanisms regulated by TNFR and NF-κB1 in pulmonary subacute O_3_ pathogenesis.

## 1. Introduction

Ozone (O_3_) is a highly reactive gaseous oxidant air pollutant. Elevated levels of ambient O_3_ have been associated with increased hospital visits and respiratory symptoms, including chest discomfort, breathing difficulties, coughs, and lung function decrement [1,2,3]. Subjects with pre-existing diseases such as asthma, rhinitis, and chronic obstructive pulmonary disorder are known to be particularly vulnerable to O_3_ and are at risk of hospitalization, exacerbations, or death [4,5,6].

Controlled O_3_ exposure to healthy volunteers and experimental animals elicit a number of pathophysiological effects, which include airway inflammation accompanied by airway hyperresponsiveness, chemokine/cytokine production, mucus overproduction and hypersecretion, reactive oxygen species production, decrements in pulmonary function, altered immune status, and epithelial damage and compensatory proliferation predominantly in ciliated cells of the upper respiratory tract and club cells in terminal bronchioles [7]. Pulmonary O_3_ responses were also augmented by metabolic disorders, including obesity and diabetes in humans, as well as in experimental animals [8,9,10], and association of air pollution and increased risk of diabetes was also reported in humans and mice [11,12]. Long term exposure to O_3_ may cause lung tumors in certain strains of mice [13].

Studies have investigated the roles of various inflammatory mediators in the pathogenic airway response to O_3_. Signal transducers, including epidermal growth factor receptor, nuclear factor kappa-light-chain-enhancer of activated B cells (NF-κB), mitogen activated kinases, and inflammasome proteins (e.g., Nlrp3) have been proposed to be downstream mechanisms of O_3_-induced airway inflammation [14,15,16]. Toll-like receptor 4 (*Tlr4*) has been identified as a O_3_-induced hyperpermeability susceptibility gene from murine genome-wide linkage analysis of subacute O_3_-induced airway hyperpermeability and injury [17,18]. Furthermore, tumor necrosis factor (*Tnf*) is a susceptibility gene for pulmonary inflammation induced by subacute O_3_ [19].

TNF is a master proinflammatory cytokine that causes diverse bioregulatory activities, including cell death, apoptosis, inflammation, and cell proliferation/differentiation [20]. TNF signaling activates NF-κB, as well as the mitogen activated kinase (MAPK), cascade/nuclear transactivation of activator protein (AP)-1, and receptor interacting serine/threonine kinase 1 [21,22]. Among the 27 TNF receptor (TNFR) superfamily, TNF binds to two distinct cellular membrane receptors, TNFR superfamily member 1A (TNF-R1p55) and 1B (TNF-R2p75) [23]. Inhibition or lack of TNF signaling significantly reduced O_3_-induced inflammation and airway hyperreactivity in rodent lungs [19,24,25,26]. Supporting the role for TNF in experimental O_3_ studies, lung functional changes were associated with a *TNF* -308G/A polymorphism in asthmatics [27]. NF-κB was proposed to play a key role in downstream of TNFR/TRAF-mediated lung injury caused by subacute O_3_ [14].

The current study was designed to identify the transcriptome events underlying pulmonary O_3_ pathogenesis and downstream targets of the TNFR and NF-κB signaling pathways. We determined time-dependent lung gene expression profiles changed by subacute O_3_ in wild-type mice and in *Tnfr*-deficient mice. We also identified NF-κB-dependent transcriptome changes using p50 NF-κB (NF-κB1)-deficient and -sufficient mice.

## 2. Materials and Methods

### 2.1. Animals and Inhalation Exposure

Male mice (6–8 weeks) deficient in TNF-specific TNFR1 and TNFR2 (B6.129S-*Tnfrsf1a^tm1Imx^Tnfrsf1b^tm1Imx^*/J; *Tnfr^-/-^*), NF-kB p50/p105 subunit (B6;129P-*Nfkb1^tm1Bal^*/J; *Nfkb1^-/-^*), TNF-a (B6.129S-*Tnf^tm1Gkl^*/J; *Tnf^-/-^*), and interleukin (IL)-6 (B6;129S2-*Il6^tm1Kopf^*/J; *Il6^-/-^*), and their respective wild-type mice (C57BL/6J for *Tnfr^+/+^* and *Tnf^+/+^*; B6129SF2/J for *Il6^+/+^*; B6129PF2/J for *Nfkb1^+/+^*), were purchased from Jackson Laboratories (Bar Harbor, ME, USA). On arrival in the National Institute of Environmental Health Sciences (NIEHS)/ALION animal facility, the mice were provided diet (NIH_31) and water ad libitum. After acclimation, the mice were placed in individual stainless-steel wire cages within a Hazelton 1000 chamber (Lab Products, Maywood, NJ, USA) equipped with a charcoal and high-efficiency particulate air-filtered air supply. The mice had free access to water and diet during exposure. *Tnfr^+/+^* and *Tnfr^-/-^* mice were exposed continuously for 6, 24, 48, or 72 h to 0.3-parts per million (ppm) O_3_. The other mice were exposed to 0.3-ppm O_3_ for 48 h. The O_3_ dosage used in the current study is a reasonable exposure level from which to make comparisons with humans, as rodents require 4−5-fold higher doses of O_3_ than humans in order to create an equal deposition and pulmonary inflammatory response, as indicated previously [14]. O_3_ was generated from ultra-high purity air (<1 ppm total hydrocarbons; National Welders, Inc., Raleigh, NC, USA) using a silent arc discharge O_3_ generator (Model L-11, Pacific Ozone Technology, Benicia, CA, USA). Constant chamber air temperature (72 ± 3° F) and relative humidity (50 ± 15%) were maintained. The O_3_ concentration was continually monitored (Dasibi model 1008-PC, Dasibi Environmental Corp., Austin, TX, USA). Parallel exposure to filtered air was done in a separate chamber. Immediately following the end of exposure, the mice were euthanized by sodium pentobarbital overdose (104 mg/kg). All animal use was approved by the NIEHS Animal Care and Use Committee.

### 2.2. Bronchoalveolar Lavage (BAL) Analyses and Lung Histopathology

The right lungs from each mouse were lavaged in situ with HBSS, and the BAL returns were analyzed for the total protein content and cell differentials, as described previously [24]. Left lung tissues from each mouse were inflated gently with 10% neutrally buffered formalin, fixed under constant pressure for 30 min, and proximal (around generation 5) and distal (approximately generation 11) levels of the main axial airway were sectioned for paraffin embedding. Tissue sections (5-μm thick) were stained with hematoxylin and eosin (H&E). The tissues were also processed for immunohistochemical staining using a rat monoclonal (IgG_1_) anti-macrophage receptor with collagenous structure (MARCO; 1:50 dilution of clone ED31, Hycult Biotech, Wayne, PA, U.S.A.). Briefly, deparaffinized and hydrated tissue sections on microscope slides were treated sequentially with antigen unmasking solution (Vector Laboratories, Burlingame, CA, USA), 0.1% proteinase K, and endogenous peroxidase quenching solution (5% H_2_O_2_) before blocking with 1.5% serum (Vectastain ABC kits). Tissue sections were then incubated overnight at 4 °C with the anti-MARCO antibody. After incubation with biotinylated rat secondary antibody (1:200, Vectastain ABC kits) and Avidin/Biotin solution, the antigens were detected by a 3,3′-diaminobenzidine-peroxidase substrate solution (10 min), and the slides were mounted with cover glasses after dehydration.

### 2.3. Lung RNA Isolation and cDNA Microarray Analysis

Lung tissues from *Tnfr^+/+^* and *Tnfr^-/-^* mice were homogenized in 2 mL Trizol (Thermo Fisher Scientific, Waltham, MA, USA) and the isolated total lung RNA was processed for Affymetrix GeneChip array analyses using mouse MOE430A arrays (Affymetrix, Inc., Santa Clara, CA, U.S.A.) in George Washington University (Dr. Andrea De Biase), as described previously [28]. The total lung RNAs from the *Nfkb1^+/+^* and *Nfkb1^-/-^* mice were isolated using RNeasy Mini Kit (Qiagen Inc., Valencia, CA, USA) and cDNA microarray was performed on mouse 430 2.0 arrays (Affymetrix) in the NIEHS Microarray Core Facility, as indicated previously [29]. Array raw data were filtered by a lower expression percentile (at least 1 sample had values within the 20% cut-off rage) and the expression levels normalized to the mean value of the experimental control (wild-type mice/air) for each gene by the quantile algorithm were analyzed statistically using GeneSpring GX14 software (Agilent Technologies, Inc., Santa Clara, CA, USA). O_3_ exposure time effects in *Tnfr^+/+^* lungs (*t*-test, *p* < 0.01) and genotype effects in air exposure (*t*-test, *p* < 0.05) or O_3_ exposure (two-way ANOVA, *p* < 0.05; Benjamin and Hochberg False Discovery Rate test for the multiple comparisons) were tested to identify the differentially expressed genes. Venn diagram analyses determined common genes varied by O_3_ between the genotypes. Ingenuity pathway analysis (IPA, Qiagen Inc., Valencia, CA, USA) was used to identify the potential molecular interactions and functions, as well as the downstream and upstream pathways. Microarray data were deposited in the Gene Expression Omnibus (accession numbers: GSE166399 for *Tnfr^+/+^* and *Tnfr^-/-^* mice and GSE166398 for *Nfkb1^+/+^* and *Nfkb1^-/-^* mice).

### 2.4. Quantitative Reverse Transcriptase-Polymerase Chain Reaction (qRT-PCR)

An aliquot of the total lung RNA was reverse transcribed into cDNAs using GeneAmp PCR System 9700 (Applied Biosystems), and cDNA (40 ng) was subjected to PCR in a 25 μL reaction containing 12.5 μL 2X Power SYBR Green Master Mix (Applied Biosystems, Foster City, CA, USA) and 240 nM of custom-designed 18s rRNA (324F 5′-tacctggttgatcctgccag-3′ and 507R 5′-ccgtcggcatgtattagctc-3′), major urinary protein 1 (*Mup1*; 228F 5′-tattatcctggcctctgacaa-3′ and 369R 5′-agataattccgagcactcttc-3′), immunoglobulin joining chain (*Jchanin*; 313F 5′-gaacaacagggagaatatct-3′ and 520R 5′-agtggtatagcacttgtttc-3′), and serum amyloid A3 (*Saa3*; 191F 5′-tacttccatgctcgggggaacta-3′ and 322R 5′-agctcttgagtcctctgctccat-3′) primers or commercially available ones (Real Time Primers, LLC, Elkins Park, PA, USA) for mouse IL-6, IL-33, tissue inhibitor of metalloproteinase 1 (*Timp1*), D site albumin promoter binding protein (*Dbp*), and pituitary tumor-transforming gene 1 (*Pttg1*), for 10 min at 95 °C, and for up to 45 cycles of 95 °C (15 s)–60 °C (1 min) using an ABI Prism 7700 Sequence Detection System (Applied Biosystems) or CFX Connect Realtime System (Bio-Rad Laboratories, Hercules, CA, USA). The relative quantification of the target gene expression was calculated using the comparative threshold cycle (C_T_) method by subtracting the fluorescence detected C_T_ of 18s rRNA from that of target gene in the same sample (ΔC_T_).

### 2.5. Protein Isolation and Western Blot Analysis

Lung cytosolic and nuclear proteins were isolated from pulverized lungs (2 pooled sample/group and 2 lungs/sample) using a kit following the manufacturer’s direction (Active Motif, Carlsbad, CA, USA). Lung total proteins were isolated from mouse lung homogenates in a radioimmunoprecipitation assay buffer (2 pooled sample/group, 2 lungs/sample). The proteins were quantified and stored in aliquots at −80 °C. The lung total or cytosolic fractions (80–100 µg) or nuclear (20 µg) proteins were separated on 10–20% Tris-HCl SDS-PAGE gels (Bio-Rad) and were analyzed by routine Western blotting using mouse specific antibodies against MARCO (Hycult Biotech), transforming growth factor (TGF)-β1 (Abcam, Cambridge, MA, USA.), c-Fos (Santa Cruz Biotechnology, Inc., Dallas, TX, U.S.A.), MUP1 (Santa Cruz), G2/mitotic-specific cyclin-B1 (CCNB1, Santa Cruz), signal transducer and activator of transcription 1 (STAT1, Santa Cruz), Lamin-B1 (Santa Cruz), and β-actin (Santa Cruz). Protein blot images were scanned and quantified using an Amersham Imager 600 (GE Healthcare Bio-Sciences Co., Piscataway, NJ, USA).

### 2.6. Sandwich Enzyme-Linked Immunosorbent Assay (ELISA) for IL-6

Aliquots of the lung cytosolic proteins (90 μg) were used to determine IL-6 using a mouse-specific ELISA kit (R&D Systems, Minneapolis, MN, USA) according to the manufacturer instructions. The optical density was measured at 450 nm and the IL-6 concentrations were determined using a standard curve.

### 2.7. Statistics

BAL, Western blotting, and qRT-PCR data are expressed as the group mean ± standard error of the mean (S.E.M.). Two-way ANOVA was used to evaluate the effects of exposure and genotype. The Student−Newman−Keuls test was used for a posteriori comparisons of the means for all multiple comparisons (*p* < 0.05). All of the statistical analyses were performed using SigmaPlot 13.0 program (Systat Software, San Jose, CA, USA)

## 3. Results

### 3.1. Time-Dependent Changes of Lung Genes by Subacute O_3_ in C57Bl/6J Mice

**Figure 1:**
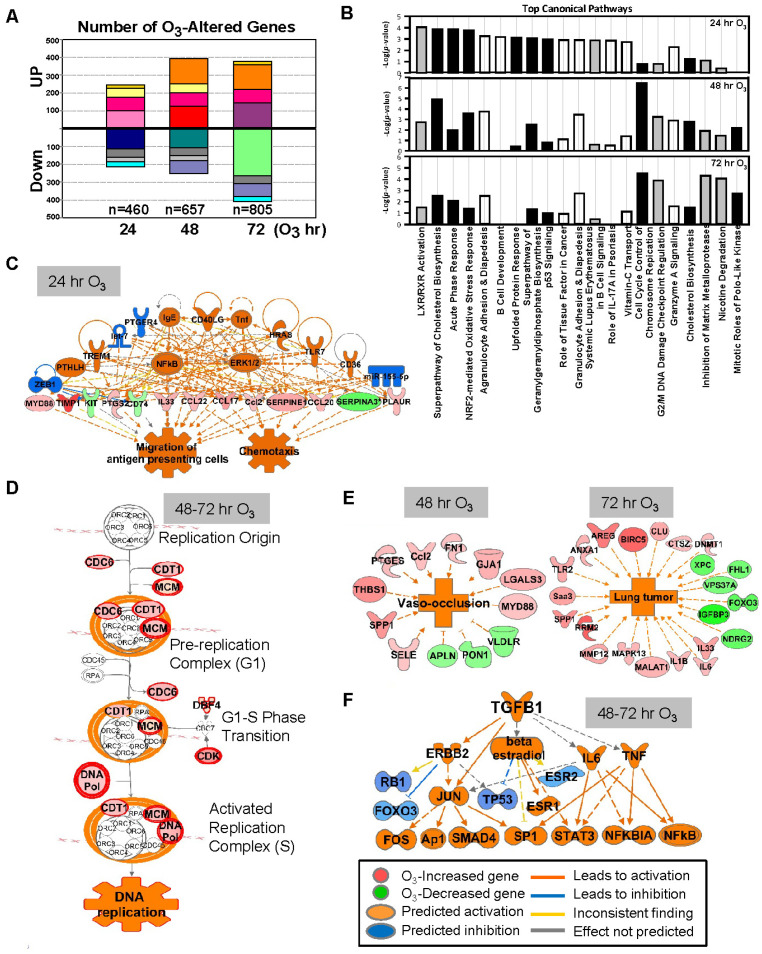
Effect of ozone (O_3_) on lung transcriptomics in C57BL/6J mice. (**A**) The number of lung genes significantly increased or decreased (*p* < 0.01 with moderated t-test) at 24 (*n* = 460), 48 (*n* = 657), and 72 (*n* = 805) h of 0.3-ppm O_3_ exposure relative to the air controls. Matching colors of stacks in the graph indicate overlapping genes between different times of exposure. (**B**) Top-ranked canonical pathway categories of the O_3_-altered genes at 24, 48, and 72 h are depicted against -log2(*P*). Black bars = positive z-score (activation); gray bars = negative z-score (inhibition); white bars = no activity pattern available. (**C**) Pathway analysis determined that tumor necrosis factor (TNF) and nuclear factor kappa-light-chain-enhancer of activated B cells (NF-κB) were potential key upstream regulators for the O_3_-responsive lung genes, which contribute to acute inflammatory and immune responses (e.g., migration of antigen presenting cells) at 24 h. (**D**) Cell cycle control of chromosome replication was the top canonical pathway of lung genes significantly upregulated by O_3_ (in red) at 48–72 h. (**E**) Top disease and biological functions of the genes altered by O_3_ included blood vessel lesion and vaso-occlusion at 48 h and lung tumor development at 72 h. (**F**) Transforming growth factor (TGF)-β1 and P53 are predicted to be one of the key upstream regulators orchestrating the lung transcriptome changes at later times (48–72 h) of O_3_ exposure. Gene or molecule colors indicate upregulation/activation (red/orange) or downregulation/inhibition (green/blue) after O_3_ exposure compared with air exposure. Analyses were done using ingenuity pathway analysis and GeneSpring software.

O_3_ caused time-dependent changes in the lung gene expressions with peak perturbation at 48–72 h of exposure (Figure 1A), when most severe lung protein edema, inflammation, and histopathologic changes take places [24,30]. Venn diagram analyses determined that most of the significantly changed genes were unique at each time (Figure 1A, and Table 1 and Table S1), and the number of common O_3_ responsive genes throughout the exposure (75 upregulated and 45 downregulated) were limited (Figure 1A). Representative canonical pathways of the enriched genes also dissociated between 24 h and 48–72 h (Figure 1B).

After 24 h of O_3_ exposure, inflammatory mediators represented by TNF and NF-κB were predicted to be upstream regulators of O_3_-altered genes (Figure 1C), and acute phase and inflammatory response (e.g., *Alb*, *Saa3*, *Myd88*, *Socs3*, *Ccl17*, and *Cxcl14*) and Nrf2-mediated oxidative stress response (e.g., *Nrf2*, *Maff*, *Gclc*, *Gpx2*, and *Sod2*) were predominantlyactivated pathways (Figure 1B,C, and Table 1 and Table S1). At later times of exposure (48 and 72 h), O_3_ most markedly activated cell cycle and inhibited DNA damage repair responses (e.g., *Ccnb1*, *Cdc6*, *Cdt1*, *Cdk1*, *Plk1*, *Mcm* family, *Cks2*, and *Pcna*; Figure 1B,D and Table S1). Distinctively, after 48 h of O_3_, the enriched genes were predicted to activate cholesterol biosynthesis and affect leucocyte migration (e.g., *Ccl17*, *Retnla*, and *Timp1*) and blood vessel lesion/vaso-occlusion (e.g., *Thbs1*, *Spp1*, and *Vldlr*; Figure 1B,E). Transcriptomics changes at 72 h of O_3_ may suppress xenobiotic degradation (e.g., *Cyp4b1*, *Fmo3*, and *Aox3*) and tissue repair (e.g., *Timp1*, *Mmp12*, and *Sdc2*) and induce lung tumorigenesis (e.g., *Rrm2*, *Birc5*, and *Areg*; Figure 1B,E). Upstream molecules including TGF-β1 and P53 were indicated to affect O_3_-induced transcriptome changes at later times (Figure 1F). Among the upstream regulators of O_3_-responsive transcriptome (24–72 h), chemical drugs including simvastatin, acetaminophen, and sulforaphane (Table S2) were suggested as therapeutic intervention to reverse O_3_ toxicity, such as reducing inflammation, reactive oxygen species, and lipids. Downregulated lung genes by O_3_ included clusters of *Mup*; insulin-like growth factor binding protein 3 (*Igfbp3*); and xenobiotic metabolizing enzymes including cytochrome P450, family 1, subfamily a, polypeptide 1 (*Cyp1a1*), and aldehyde oxidase 3 (Table 1 and Table S1). While *Hspa1a* and *Hspa1b* encoding TLR4-dependent heat shock protein 70 (HSP70) [28] were upregulated by O_3_, many other HSP genes (e.g., *Hspb1*, and *Hsph1*) were significantly decreased at 48 h of O_3_ (Table S1).

### 3.2. TNFR-Dependent Lung Transcriptome Changes

#### 3.2.1. Air-Exposed Lungs

**Figure 2:**
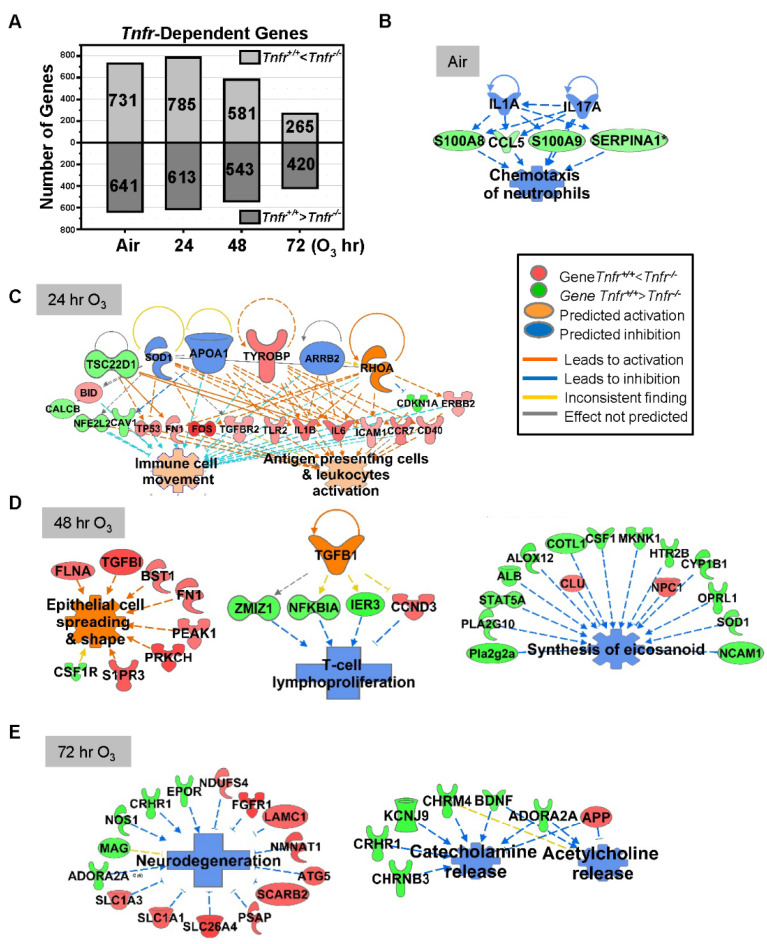
Tumor necrosis receptor (TNFR)-dependently regulated lung genes. (**A**) The number of lung transcripts significantly (*p* < 0.05) upregulated (light gray) or downregulated (dark gray) different *Tnfr*-deficient (*Tnfr^-/-^*) mice relative to wild-type (*Tnfr^+/+^*) mice at baseline (air, moderated t-test) or after 24, 48, and 72 h of 0.3-ppm ozone (O_3_) exposure (two-way ANOVA). (**B**) In air-exposed basal lungs, the inhibition of interleukins (ILs) 1A and 17A were predicted to suppress TNFR-dependent lung genes (e.g., *Ccl5*, *S100a8*, *S100a9*, and *Serpina1*), leading to the inhibition of inflammatory cell chemotaxis. (**C**) After 24 h O_3_ exposure, a compensatory increase of the genes involved in immune cell activation and movement were manifest in *Tnfr^-/-^* lungs compared with *Tnfr^+/+^* mice. (**D**) After 48 h of O_3_ exposure, when lung injury and inflammation are greatest, modulation of potential upstream regulators including transforming growth factor (TGF)-β1 may change transcriptomes to suppress lymphocyte proliferation and eicosanoid synthesis and activate epithelial cell spreading/integrity in *Tnfr^-/-^* lungs. (**E**) *Tnfr^-/-^* mouse lungs after 72 h of O_3_ exposure had transcriptome changes to suppress the release of neurotransmitters and inhibit neurodegeneration. Gene or molecule colors indicate upregulation/activation (red or orange) or downregulation/inhibition (green or blue) in *Tnfr^-/-^* mice compared with *Tnfr^+/+^* mice after air or O_3_ exposure. Analyses were done using Ingenuity Pathway Analysis and GeneSpring software.

Lung genes basally expressed lower in *Tnfr^-/-^* mice than in *Tnfr^+/+^* mice (Table 2 and Table S3) were represented by *Mup* clusters, *Pttg1*, HSPs (*Hsph1* and *Hspa4l*), S100 calcium binding proteins (*S100a8* and *S100a9*), chemokines (*Ccl5* and *Ccl17*), cytochrome P450 family (*Cyp1a1* and *Cyp3a11*), and apolipoproteins (*Apoa2* and *Apoa1*). In contrast, basally overexpressed lung genes in *Tnfr^-/-^* mice compared with *Tnfr^+/+^* mice (Table 2 and Table S3) included *Dbp* and nicotineamide nucleotide transhydrogenase (*Nnt*). Basally *Tnf*-dependently expressed genes were related to vascular disorders, as well as to the inhibition of inflammatory cell response (Figure 2A,B and Table 3).

#### 3.2.2. O_3_-Exposed Lungs

*Tnfr*-dependent variation of lung gene expression was more marked at 24–48 h than at 72 h of O_3_ exposure (Figure 2A). Transcriptome changes that occurred in *Tnfr^-/-^* mice at 24 h were predicted to potentiate immune and inflammatory systems (Figure 2C, and Table 2, Table 3 and Table S4), suggesting a compensatory or adaptive immune response in the absence of TNFR1 and TNFR2 signaling. Upstream regulators of this TNFR-dependent early transcriptome response may include tyrosine kinase-binding protein (TYROBP), APOA1, and arrestin beta-2 (ARRB2) in *Tnfr^-/-^* mice (Figure 2C). At 48 h of O_3_ the pulmonary epithelial proliferation, inflammatory cell influxes, and epithelial injury were significantly more suppressed in *Tnfr^-/-^* mice than in *Tnfr^+/+^* mice [24]. TGF-β1 and protein O-GlcNAcase or P53 were potential key upstream regulators, and TNFR-dependently enriched genes in *Tnfr^-/-^* mice were predicted to inhibit lymphocyte proliferation/eicosanoid synthesis (e.g., *H2-Q4*, *Il33*, *Nfkbia*, and *Tgfbi*) and macromolecule oxidation (e.g., multiple cytochrome P450 subfamilies) and activate epithelial cell spreading/integrity (e.g., *Fn1* and *Flna*; Figure 2D, and Table 2, Table 3 and Table S4). Many lipid metabolism (e.g., *Fdps*, *Got2,* and *Sgms1*) and eicosanoid synthesis (e.g., *Alb*, *Alox12*, and *Ptgs1*) genes were also relatively suppressed in *Tnfr^-/-^* mice compared with *Tnfr^+/+^* mice at 48 h O_3_ (Figure 2D, and Table 2, Table 3, and Table S4). At 72 h O_3_, lack of TNFR signaling inhibited transcriptomes of neurodegeneration (e.g., *Slc26a4*, *Epor*, and *Nmnat1*) and transport for neurotransmitters, acidic amino acids, and anions (e.g., *Chrm4*, *App*, and *Kcnj9*; Figure 2E, Table 2, Table 3 and Table S4).

### 3.3. Nfkb1-Dependent Lung Transcriptome Changes

#### 3.3.1. Air-Exposed Lungs

NF-κB1 (p50/p105) forms the most abundant heterodimer with RelA, but it also forms a p50−p50 homodimer. The NF-κB1 homodimer is known to work as a transcriptional activator, similar to other NF-κB heterodimer complexes (e.g., RelA-p50 and c-Rel-p50), as well as a transcriptional repressor by inhibiting the binding of other NF-κB dimers to lead to the suppression of NF-κB target gene expressions during innate immune responses [31,32]. Supporting the transcriptional repressor role of NF-κB1, basally different lung genes in *Nfkb1^-/-^* mice compared with *Nfkb1^+/+^* mice (*t*-test *p* < 0.05 *n* = 1395 genes; Table 4 and Table S5) were predominantly enriched to increase leukocyte extravasation/adhesion genes (e.g., CCL and CXCL chemokines, *Ccr2*, claudins, integrins, *Tnfrsf1b*, *Sell*, *Cd14*, and *Lbp*). In addition, enriched genes for the antigen presentation to CD8^+^ T lymphocytes (e.g., *B2m*, *Hla-G*, *Nlrc5*, *Psmb8*, *Psmb9*, and *Tap1*) were overexpressed in *Nfkb1^-/-^* lungs compared with *Nfkb1^+/+^* lungs (Table 4 and Table S5). Furthermore, the downregulation of other sets of immune genes (e.g., *Jchain*, *Cxcl13*, *Pcdhb3*, and *Marco*) were also marked in *Nfkb1^-/-^* mice compared with in *Nfkb1^+/+^* mice (Table 4 and Table S5). Activation of interferon (IFN) regulatory factors (IRF3 and IRF7) has been predicted to serve as upstream regulators of NF-κB1-dependent genes (e.g., *Ifit3*, *Stat1*, and *Oas1*), which would cause IFN-mediated decrease in infectivity in basal lungs deficient in *Nfkb1* (Table 4 and Table S5). This is consistent with the known *Nfkb1^-/-^* mouse phenotypes such as defective responses to infection and specific antibody production [33].

#### 3.3.2. O_3_-Exposed Lungs

**Figure 3:**
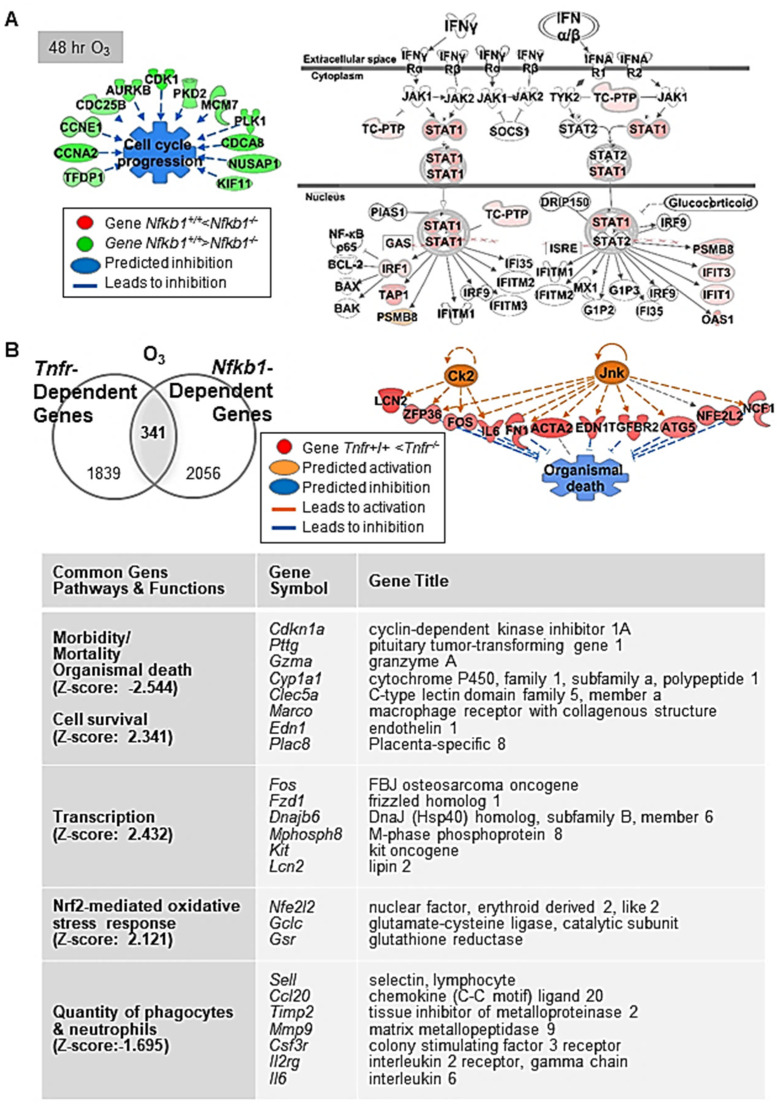
Nuclear factor kappa-light-chain-enhancer of activated B cells (NF-κB) dependently regulated lung genes during ozone (O_3_)-induced lung injury development. (**A**) At 48 h O_3_ exposure, downregulation of multiple genes in the family of cyclin (e.g., *Ccnb1*), cell division cycle (e.g., *Cdca8*), and centromere protein (e.g., *Cenph*) in *Nfkb1*-deficient mice (*Nfkb1^-/-^*) compared with wild-type (*Nfkb1^+/+^*) mice was marked, suggesting suppressed cell proliferation and enhanced DNA damage checkpoint regulation pathways in *Nfkb1^-/-^* mice. In contrast, heightened interferon (IFN) signaling genes (e.g., *Irf1*, *Psmb8*, *Tap1*, and *Stat1*) and predicted activation of upstream regulators (e.g., IRF7 and IFN type I receptor) indicated an enhanced innate immunity in *Nfkb1^-/-^* mice than in *Nfkb1^+/+^* mice during O_3_-induced lung injury development. (**B**) Common differentially regulated genes between *Tnfr^+/+^* and *Tnfr^-/-^*, and *Nfkb1^+/+^* and *Nfkb1^-/-^* (*n* = 341) were determined by Venn diagram analysis. Transcriptome signatures of TNFR/NF-κB-axis mediated pulmonary O_3_ toxicity were predicted to modulate cell/organism death and survival, transcriptional regulation, oxidative stress, and inflammation (**B**). Gene or molecule colors for (**A**,**B**) indicate upregulation/activation (red/orange) or downregulation/inhibition (green/blue) in *Nfkb1^-/-^* mice compared with *Nfkb1^+/+^* mice after O_3_ exposure. Analyses were done using Ingenuity Pathway Analysis and GeneSpring software.

After 48 h of O_3_, lack of *Nfkb1* predominantly suppressed lung cell cycle progression and enhanced DNA damage checkpoint regulation pathways through downregulation of multiple genes in the families of cyclin, cell division cycle, centromere protein, and centrosomal protein (Figure 3A, and Table 4 and Table S6). This corresponded to the significant decrease in O_3_-induced centriacinar cell proliferation in *Nfkb1^-/-^* mice compared with *Nfkb1^+/+^* mice [14]. Similar to basal lung transcriptomics, pathway analyses indicated heightened IFN signaling genes (e.g., *Irf1*, *Psmb8*, *Oas1*, *Tap1*, and *Stat1*) and activated upstream regulators, IRF7 and IFN type I receptor (IFAR), in O_3_-exposed *Nfkb1^-/-^* mice compared with *Nfkb1^+/+^* mice (Figure 3A, and Table 4 and Table S6). The results demonstrated suppressed lung cell proliferation and heightened antimicrobial and immune response transcriptomes noticeable in *Nfkb1^-/-^* mice relative to *Nfkb1^+/+^* mice after O_3_. Certain inflammatory genes bearing potential or confirmed NF-kB binding sites were (e.g., *Ccl20*, *Saa3*, *Fos*, *Il6*, *Ido1*, *Mmp9*, and *Psmb9*) more heightened in *Nfkb1^-/-^* mice than in *Nfkb1^+/+^* mice (Table 4 and Table S6), which is suggestive of p50−p50 homodimer-mediated suppression.

### 3.4. Common Differentially Expressed Genes in Tnfr^-/-^ and Nfkb1^-/-^ Mice Exposed to O_3_

Venn diagram analysis determined the common genes (n = 341) differentially expressed between *Tnfr^+/+^* and *Tnfr^-/-^*, and *Nfkb1^+/+^* and *Nfkb1^-/-^* genotypes after O_3_ exposure (Table S7). These common differentially regulated genes were enriched in host defense functions through activation of cell survival and the inhibition of cell death/mortality and oxidative stress (Figure 3B). A subset of genes suppressed in both *Tnfr^-/-^* and *Nfkb1^-/-^* mice relative to their wild-type controls may be modulated by the axis of TNFR and p50−p65 NF-κB heterodimers (i.e., transcription activator), while the genes suppressed in *Tnfr^-/-^* mice but heightened in *Nfkb1^-/-^* mice may include the ones regulated by TNFR and p50−p50 NF-κB homodimer (i.e., transcription suppressor) signaling axis. Among them, mouse *Col1a2*, *Gclc*, *Il6*, *Ido1*, *Junb*, *Lcn2*, *Mmp9*, *Psmb9*, and *Psme1* are known to possess functional NF-κB binding sites, and the human homology of many other genes (e.g., *CCL20*, *EDN1*, *HSP90AA1*, *TNC*) are known to be direct NF-κB downstream targets (https://www.bu.edu/nf-kb/gene-resources/target-genes; https://bioinfo.lifl.fr/NF-KB, searched on 30 July 2017). We searched the DECODE database (SAbiosciences.com, searched on 30 July 2017) to find potential NF-κB p50−p65 target genes (bearing NF-κB binding consensus sequences 5′-GGGRNYYYCC-3′ or 5′-GGAATTYCCC-3′; R is A/G, Y is T/C, N is any nucleotide) in the promoter of selected common differentially regulated genes (i.e., genes > 1.5-fold lower in O_3_-*Nfkb1^-/-^* than in O_3_-*Nfkb1^+/+^* genotypes in Table S7). Genes including endothelin 1 (*Edn1*), prostate stem cell antigen (*Psca*), *Dbp*, and chemokine (C-C motif) ligand 22 (*Ccl22*) had predicted consensus sequences indicating potential TNFR-NF-κB target genes during O_3_-induced pulmonary pathogenesis (Table 5).

### 3.5. Effects of Tnf and Il6 on O_3_-Induced Lung Injury

**Figure 4:**
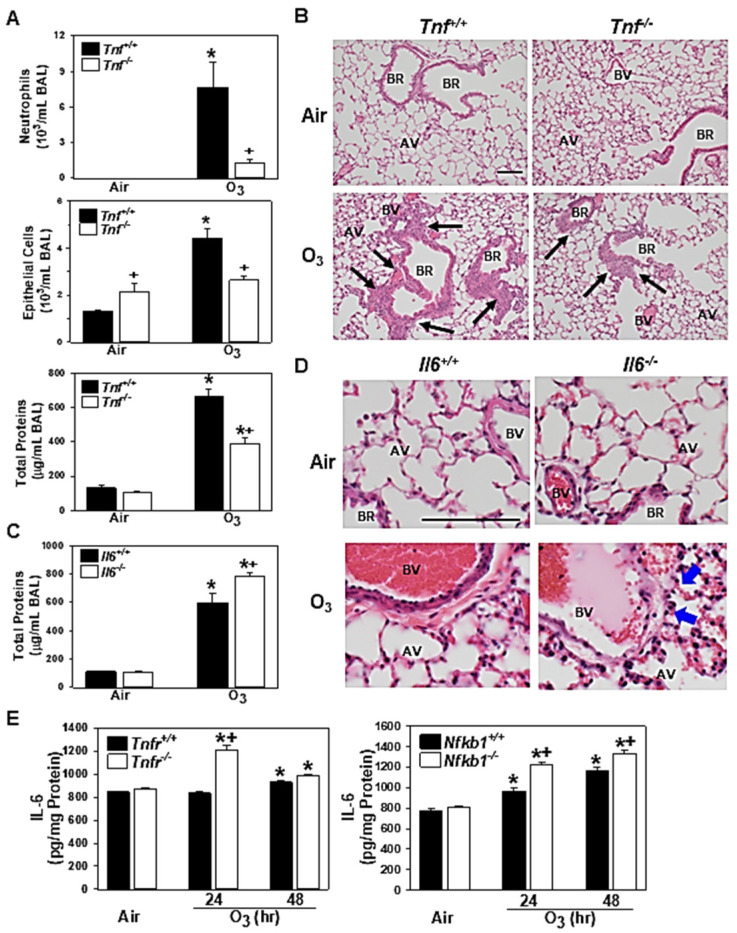
Functional roles of tumor necrosis factor (TNF) and interleukin 6 (IL-6) in pulmonary O_3_ pathogenesis. Effect of targeted disruption of *Tnf* (**A**) or *Il6* (**C**) was determined by bronchoalveolar lavage (BAL) analyses in gene knockout mice (*Tnfr^-/-^*, *Il6^-/-^*) and their wild-type mice (*Tnf^+/+^*, *Il6^+/+^*) after 48 h of exposure to air and 0.3-ppm O_3_. Data are presented as means ± S.E.M (*n* = 4–5 mice/group). Representative light photomicrographs of H&E-stained lung sections from *Tnf^+/+^* and *Tnfr^-/-^* mice, (**B**) and *Il6^+/+^* and *Il6^-/-^* mice (**D**) exposed to air or O_3_ (48 h). Black arrows depict bronchiolar/terminal bronchiolar epithelium under proliferation. Blue arrows depict protein exudation in perivascular regions. AV = alveoli. BV = blood vessel. BR = bronchiole or terminal bronchiole. Bars = 100 μm. TNFR- and NF-κB1-dependent level of lung IL-6 proteins determined by enzyme-linked immunosorbent assay (**E**). Data are presented as means + S.E.M (*n* = 3/group). *, significantly different from genotype-matched air control mice (*p* < 0.05). +, significantly different from O_3_-exposed corresponding wild-type (*Tnf^+/+^, Il6^+/+^*, *Tnfr^+/+^*, or *Nfkb1^+/+^*) mice (*p* < 0.05).

As seen in *Tnfr^-/-^* mice [24], the mice deficient in *Tnf* cluster genes (*Tnf*, *Lta*, and *Ltb*) [34], and the mice treated with the TNF antibody [19], BAL fluids from *Tnf^-/-^* mice had significantly reduced numbers of lung neutrophils and epithelial cells and amounts of proteins compared with those from *Tnf^+/+^* mice at 48 h of O_3_ (Figure 4A). The histopathologic analysis indicated that O_3_-induced centriacinal proliferation indicated by thickened bronchiolar and terminal bronchiolar epithelium (arrows) were also less marked in *Tnf^-/-^* mice compared with *Tnf^+/+^* mice (Figure 4B). The current microarray analysis and a previous study [14] demonstrated that the abundance of *Il6* mRNA was higher in both *Tnfr^-/-^* and *Nfkb1^-/-^* mice compared with their corresponding wild-type mice after O_3_ (Table 2, Tables S4 and S6). In *Il6^-/-^* mice, lung protein hyperpermeability determined by the BAL protein concentration was significantly higher than that in *Il6^+/+^* mice (Figure 4C). However, the numbers of O_3_-induced neutrophils or epithelial cells in BAL fluids were not significantly different between the two genotypes (data not shown). Consistent with the heightened BAL protein level in *Il6^-/-^* mice, H&E-stained lung tissue sections depicted more marked edema and permeability in the perivascular region (arrows), which accompanied protein exudation (pink staining) and congestion (red blood cells) into the alveolar air space in *Il6^-/-^* mice compared with *Il6^+/+^* mice after O_3_ (Figure 4D). Gene expression data and BAL analysis suggested a potential protective role for IL-6 in this model. ELISA determined significantly increased levels of IL-6 in *Tnf^+/+^* (48 h) and *Nfkb1^+/+^* (24 and 48 h) mouse lungs after O_3_ exposure (Figure 4E). The O_3_-enhanced IL-6 protein amounts were significantly higher in *Tnfr^-/-^* and *Nfkb1^-/-^* mice compared with their corresponding wild-type mice (Figure 4E), which supported TNFR- and NF-κB1-dependent *Il6* mRNA abundance.

### 3.6. Validation of Microarray Results

**Figure 5:**
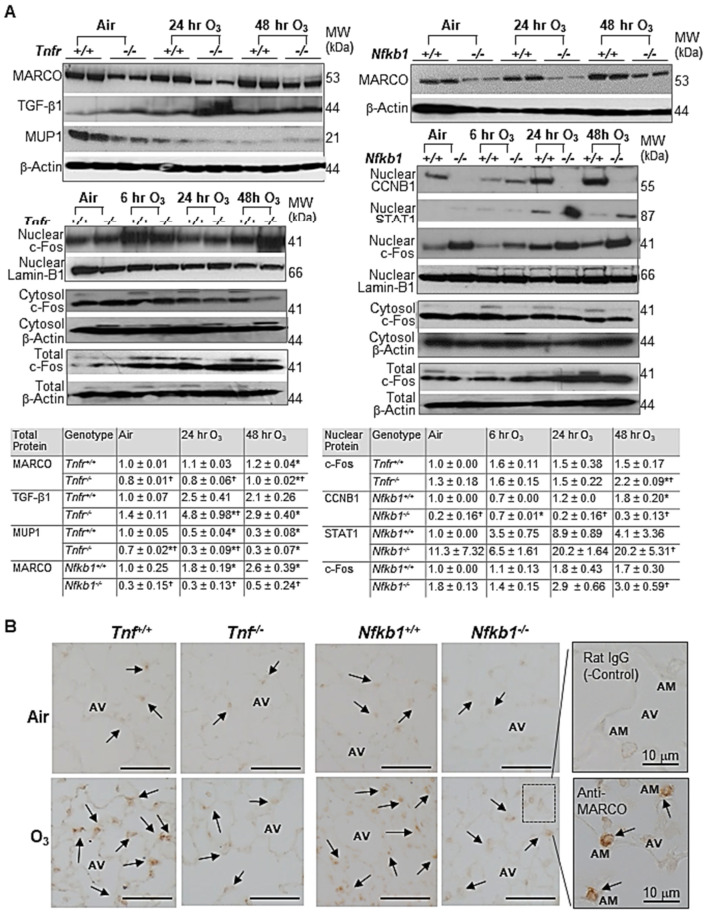
Microarray analysis validation of tumor necrosis factor receptor (TNFR)- and NF-κB1-dependent transcriptomics in response to ozone (O_3_). (**A**) Western blot analyses determined TNFR- and/or NF-κB1-dependent expression of a lung macrophage receptor with a collagenous structure (MARCO), transforming growth factor (TGF)-β1, major urinary protein 1 (MUP1), nuclear c-Fos, nuclear G2/mitotic-specific cyclin-B1 (CCNB1), and nuclear signal transducer and activator of transcription 1 (STAT1). β-Actin (for total and cytosol) and Lamin-B1 (for nuclear) levels detected as the internal controls. Representative digitized lot images are presented. Group mean ± S.E.M. presented for quantified digitized images (*n* = 4/group for total protein bands, *n* = 2/group for nuclear protein bands). * Significantly different from genotype-matched air control (*p* < 0.05). ^†^ Significantly different from exposure-matched wild-type mice (*p* < 0.05). (**B**) TNFR- and NF-κB1- dependent MARCO protein expression was localized on mouse lungs by an immunohistochemical method after air or O_3_ (48 h) exposure. Representative light photomicrographs of lung sections (*n* = 2–3/group) presented. Arrows depict MARCO staining on the plasma membrane and/or cytoplasm of alveolar macrophages (AMs) in the alveoli (AV). A box depicts representative images of magnified AMs in AV stained with rat IgG (negative control) or ant-MARCO antibody. Bars (unlabeled) = 50 μm.

qRT-PCR determined TNFR-dependently increased tissue inhibitor of metalloproteinase (*Timp1*) and *Il33* or decreased *Mup1* after air and O_3_ exposure (Supplemental Figure S1A). *Timp1* and *Il33* mRNAs were significantly upregulated in O_3_-resistant *Tnfr^-/-^* mice compared with susceptible *Tnfr^+/+^* mice. A significant decline of *Mup1* mRNA abundance by O_3_ was greater in *Tnfr^-/-^* mice than in *Tnfr^+/+^* mice. Differential expression of NF-κB1-dependent genes, *Jchain*, *Dbp*, and *Saa3*, were also significantly different between two genotypes at baseline and/or after 48 h O_3_ (Figure S1B). The mRNA expressions of common differentially regulated genes *Pttg1* and *Il6* were significantly lower or higher, respectively, in both *Tnfr^-/-^* and *Nfkb1^-/-^* mice compared with their corresponding wild-type mice (Figure S1C). Western blot analyses found TNFR-dependent variations of the total TGF-β1 and MUP1 proteins and NF-κB1-dependent level of nuclear CCNB1 and STAT1 proteins in the lungs exposed to O_3_ (Figure 5A). The amount of total MARCO and nuclear c-Fos proteins, common differentially regulated gene products, were also varied similarly in *Tnfr^-/-^* and *Nfkb1^-/-^* mice compared with their corresponding wild-type mice (Figure 5A). The total lung protein levels of c-Fos were time-dependently increased by O_3_ in all mice, while the cytoplasmic c-Fos abundances were marginally changed or decreased by O_3_ (Figure 5A). MARCO was detected in alveolar macrophages and was localized mostly in their plasma membranes and/or cytoplasm (Figure 5B). Consistent with the differential protein levels detected by Western blotting, lower levels of MARCO localization were found in *Tnfr^-/-^* and *Nfkb1^-/-^* mouse lungs relative to their wild-type mice after 48 h of O_3_ exposure (Figure 5B).

## 4. Discussion

We elucidated murine lung transcriptional profiles that were time-dependently changed by subacute O_3_ exposure. Comparative analyses between wild-type and gene knockout mice enriched pulmonary genes modulated via TNFR and/or p50 NF-κB pathways during O_3_ exposure. Supporting our previous findings that *Tnf* is a susceptibility gene for subacute O_3_-induced pulmonary inflammation [19] and that lack of *Tnfr* and *Nfkb1* alleviated pulmonary O_3_-induced injuries [14,24], the enriched genes may play key roles in pulmonary O_3_ pathogenesis in mice.

There are limited resources of global cDNA expression data in O_3_-exposed airways. In humans, the microRNA (miRNA) profile on sputum samples exposed to controlled O_3_ (0.4 ppm for 2 h during exercise) disrupted immune and inflammatory-related miRNAs including neutrophil-specific miR-143 and myeloid cell specific miRNA-223, which supported an increased number of neutrophils in the sputum [35]. The O_3_-responsive miRNAs were also predicted to post-transcriptionally alter the genes involved in cell cycle (e.g., *Ccnd1*) and cellular growth and survival (e.g., *Arhgdia*, *Sod2*) [35]. In rodents, Gohil et al. [36] first demonstrated microarray profiles after acute exposure to O_3_ (1 ppm, 8 h/day for 3 days) in adult C57BL/6J mice predominantly upregulated multiple cell cycle progression genes (e.g., *Septin5*, *Nap1*, and *Cdc2a*) and NF-κB-activated genes such as *Saa3* and plate-derived growth factor receptor alpha (*Pdgfra*) in the lungs. In contrast, the suppression of families of transcripts encoding contractile proteins such as troponins, myosins, and actins; cytochrome P450s; and antigen presenting and immune surveillance molecules (e.g., cluster of MHC class and immunoglobulins) were found after O_3_ exposure [36]. O_3_-induced repression of the muscle-specific proteins and cytochrome P450 transcription may activate NF-κB [37,38]. Acute O_3_ (0.8 ppm, 8 h/day for 2 days) also altered the genes involved in oxidative stress and defense, including NRF2 target antioxidants (e.g., *Gclc*, *Gst*, and *Homx1*) in C57BL/6J mouse lungs [39]. The authors did not find significant differences in the lung inflammation and gene expression profiles between mice that lacked *Ercc6*, the DNA excision repair protein gene, and their heterozygous controls [39]. A recent RNA-seq analysis of acute O_3_ exposure (1 or 2 ppm for 3 h) was done in two compartments of the lung, dissected conducting airways (no parenchyma) and macrophages collected from BAL, from adult female C57BL/6J mice, in order to segregate transcriptomics in inflammation and tissue injury. Concentration- and compartment-specific profile comparisons indicated more dynamic transcriptional changes in the conducting airways than in the macrophages [40]. Alteration of antioxidant (e.g., *Gsta1*), immune (e.g., *Cx3cl1*), cell cycle (e.g., *Mcm* family and *Cdk1*), and acute phase (e.g., *Saa3* and *Lcn2*) genes were common in both compartments of the airways, while decreases in the epithelium barrier and marker genes (e.g., *Foxj1*, *Cyp2f2*, and *Scgb1a1*) were distinct in conducting airway compartments, supporting epithelial cell sloughing and metaplasia/hyperplasia in the conducting airways [40]. Neutrophil activation/degranulation and immune signaling genes (e.g., *Ccl17* and *Retnla*) were the most enriched in alveolar macrophage transcriptome after O_3_ [40]. Another RNA-seq analysis on three compartments of C57BL/6J lungs (i.e., tracheobronchial epithelium, lung parenchyma, and CD11b^+^ BAL macrophages) exposed to subchronic levels of O_3_ (0.8 ppm, 4 h/day for total 14 days) determined that lung parenchyma and macrophages were enriched with inflammatory pathway genes (e.g., *Ccl2*, *Ccl17*, *Timp1*, *Saa3*, *Lcn2*, and *Mmp14*) [41]. Transcriptomes of tracheobronchial epithelium and parenchyma, on the other hand, had predominant changes in cell cycle and DNA repair genes (e.g., *Cdc20b*, *Cdk1*, and *Retnla*) in response to subchronic O_3_ [41]. In this study, most transcriptome changes by subchronic O_3_ were common in male and female mice, while female mice were more susceptible to inflammatory cell influx, epithelial loss, and compensatory proliferation, which was supported by the more robust changes of the gene expression in females mice [41]. In Fischer rat lungs, acute O_3_ (5 ppm, 2 h) altered genes with similar functions reported in mouse studies, and upregulation of inflammatory and redox (e.g., *Jun*, *Cxcl2*, *Nos2*, *Hsp27*, and *Nfkb1*), cell cycle and DNA repair (*Ccne1*, *Cdc2*, and Arrb15b), and lipid metabolism (e.g., *Faah* and *Plaa*) genes were evident [42]. In developmental mouse lung at 3 days postnatal (transition from saccular to alveolar stage), transcriptome changes by acute O_3_ (1 ppm, 3 h) exposure were less robust than those seen in adult mice, and the global suppression of cell cycle-related lung genes (e.g., *Cenpf*, *Cdca8*, *Cdk1*, *Cntn14*, *Cdc45*, *Mki67*, and *Pcna*) was rather marked until 24 h postexposure [43]. These results indicate that acute O_3_ exposure disturbs cellular proliferation and differentiation of lungs undergoing development.

Although the current study demonstrated whole lung transcription profiles without dissection of compartment- or cell-specific transcriptomics, our results characterized multiphasic transcriptome changes by O_3_ exposure time, and only 18% genes were altered commonly in more than two time points. Subacute O_3_ responsive genes likely have roles in oxidative injury and antioxidant induction, chemotaxis, and immune cell development during early exposure (at 24 h); cell cycle progress, blood vessel lesions, and cholesterol biosynthesis during peak lung injury (at 48 h); and xenobiotic metabolic process, tumorigenesis, and tissue injury/repair at a later time (72 h). After comparison with compartmental transcriptome studies, we predicted the cellular or tissue origin for reproducible O_3_ responsive genes across multiple transcriptome studies; for example, increased lipocalin 2 (*Lcn2*) and small proline-rich protein 1A (*Sprr1a*) and decreased *Igfbp3* may be mainly from the parenchyma [41]; increased matrix metalloproteinase 14 (*Mmp14*) from the macrophages [41]; increased resistin like alpha (*Rentnla*)*,* leucine-rich alpha-2-glycoprotein 1 (*Lrg1*), and *Timp1* from both the macrophages and parenchyma [40,41]; increased *Saa3* from all compartments of airways [40,41]; and increased chromatin licensing and DNA replication factor 1 (*Cdk1*) and ubiquitin-conjugating enzyme E2C (*Ube2c*), as well as decreased *Cyp1a1*, *Mup* family, and serine (or cysteine) peptidase inhibitor, clade A, member 3K (*Serpina3k*) from the conducting airways [40,41].

The NF-κB family of proteins has an important role in inflammatory responses initiated by TNF [44]. Despite the identification of a few well-accepted NF-κB target genes in humans and mice (e.g., *NFKBIA*, *TNFAIP3*, and *MYC*), transcriptional outputs through NF-kB are not well understood due to the complexity of NF-κB dynamics and the NF-κB-binding landscape in the gene expression. NF-κB responses in gene transcription are known to vary depending on the cell type as well as the initiating stimulus [45]. In addition, p50 and p52, among five NF-kB family proteins, do not have a transactivation function, and they can activate transcription through heterodimerization with p65 or others [46]. Importantly, the p50−p50 homodimer binding to NF-κB motif inhibits other NF-κB dimer complex binding, and thus it often, but not always, serves as a transcriptional suppressor for NF-κB target genes [47]. The p50−p50 homodimer has thus been shown to have anti-inflammatory functions through repression of proinflammatory genes and enhancement of anti-inflammatory genes [48,49]. TNF I triggered a strong, sustained p65−p50 activation with a relatively lower level of p50−p50 [47]. Therefore, common differentially regulated genes by TNFR and NF-κB1 in the current study may include NF-κB target genes inducible by the TNFR-NF-κB (p50−p65) axis, as well as those suppressible by the p50−p50 homodimer. That is, the genes suppressed in *Tnfr*^-/-^ and *Nfkb1^-/-^* mice (e.g., *Pttg1*, *Mmp3*, and *Marco*) are likely p50−p65-inducible genes. Genes downregulated in *Tnfr*^-/-^ mice but overexpressed in *Nfkb1^-/-^* mice after O_3_ exposure (e.g., *Gzma*, *Cyp1a1*, *Nkg7*, *Il6*, *Ccl20*, and *Kit*) are possibly p50−p50-repressed genes. Functional NF-κB motifs have been discovered in the murine *Il6* promoter [50]. Therefore, together with augmented pulmonary protein hyperpermeability in *Il6*-deficient mice, IL-6 was predicted as an anti-inflammatory cytokine in the current subacute O_3_ pathogenesis and p50−p50 homodimer may modulate its transcription. We elucidated the potential NF-κB binding motifs from several common differentially regulated genes (e.g., *Psca* and *Edn1*), and these genes are postulated as direct downstream targets of the TNFR-NF-κB signaling pathways.

One of the genes modulated by both TNFR and NF-κB1 is *Marco*. MARCO expressed in alveolar macrophages recognizes oxidized lipids and provides innate defense against inhaled pathogens [51]. As a downstream effector of TLR4, which is a murine susceptibility gene for subacute O_3_-induced pulmonary hyperpermeability [17,28], MARCO plays a protective role in subacute O_3_-exposed mouse lungs through the inhibition of oxidized surfactant lipid production and inflammation [52]. As TLR4 and TNFR are key immune receptors in subacute O_3_ pathogenesis, and the NF-κB pathway is also known to play an important role in the TLR4-mediated immune responses [53,54], we compared the current transcriptome profiles with TLR4-dependent O_3_ transcriptomics (GEO accession # GSE20715, [28]). Commonly regulated genes by TLR4 and NF-κB1 were enriched in lipid derangement, including the disruption of membrane phospholipids, reaction with unsaturated fatty acids in airway lining fluids, interruption of fatty acid/steroid metabolism (e.g., *Dbp* and *Cpt1a*), as well as in cell-mediated immunity and lymphoid tissue hyperplasia (e.g., *Cxcl1*, *Ccl20*, and *Ptpn2*; Figure S2). Common gene transcripts regulated by TNFR and TLR4 were enriched in the engulfment of phagocytes (e.g., *Marco*, *Icam1*, *Lcn2*, and *Il33*), protein ubiquitination (e.g., *Dnaja1*, *Hspd1*, and *Psma3*), fatty acid metabolism (e.g., *Acot7*, *Ptges*, *Elovl1*, and *Lpin1*), and glutathione homeostasis/redox (e.g., *Gsr*, *Gstm1*, and *Gltx*; Figure S3). Overall, the TNFR-NF-κB and TLR4-NF-κB pathways or crosstalk modulated distinct transcriptomes during the development of O_3_-induced lung injury in mice. Further studies are warranted for these common genes regulated by these three critical immune and inflammatory mediators (Table S7).

Increasing evidence and TNFR-/TLR4-enriched pathways indicate an association of airway O_3_ responses with extracellular and/or cellular lipid biology. On airway epithelium lining fluids rich in surfactant, inhaled O_3_ chemically reacts with cholesterol or phospholipids and generates cytotoxic ozonolysis products represented by 5β,6β-epoxycholesterol (β-epoxide) [55,56]. These lipid-ozonized products are proinflammatory and are known to contribute to O_3_-induced airway inflammation [57,58]. Eicosanoids (e.g., prostaglandins, leukotrienes, and thromboxanes) synthesized by peroxidation of arachidonic acid by lipoxygenases, cyclooxygenases, and cytochrome P450 are also inflammatory mediators increased by O_3_ leading to airway hyperresponsiveness and extrapulmonary outcomes, including vasoconstriction [59,60]. O_3_ exposure-released adrenal-derived stress hormones (e.g., epinephrine and corticosterone) disrupted lipid and carbohydrate metabolism, leading to hyperglycemia, glucose intolerance, and lung injury in rats [61,62]. Further rodent studies demonstrated that obesity augmented acute O_3_-induced airway hyperresponsiveness and inflammation [63,64,65], and diabetes caused early and exacerbated lung inflammation and fibrotic changes in response to subchronic O_3_ (0.5 ppm, 4 h/day for 13 weeks) [10]. Epidemiological studies also showed a positive association between O_3_ exposure and adult insulin resistance and preexisting lipid disorders and metabolic conditions (e.g., obesity and diabetes) [66,67,68]. Metabolomic analysis of human serum revealed that acute O_3_ exposure markedly increased lipid mobilization and catabolism products (e.g., monoacylglycerol and medium- and long-chain free fatty acids) [69]. Interestingly, human population studies indicated an association of gain-of-function *TNF* −304G/A polymorphism with obesity-related airway hyperresponsiveness in asthmatics [70]. In obese mice, *Tnfr2* deficiency reduced body weight and acute O_3_-induced inflammation and obesity-related airway hyperresponsiveness [64,71]. Overall, these studies suggested a role of lipid derangement in airway and extrapulmonary O_3_ pathogenesis.

Our transcriptomic and pathway analyses results suggested direct effects of known or potential NF-κB motif-bearing genes in O_3_-induced pulmonary edema (e.g., *Il6*), T cell immunity (e.g., *Ccl17*, *Ccl22*, and *Il27ra*), cardiac mortality and vasoconstriction (e.g., *Edn1*), extracellular matrix degeneration (e.g., *Col1a2* and *Mmp9*), and interruption of lipid metabolism (e.g., *Dbp* and *Tef*) via the TNFR-NF-κB signaling axis. However, these TNFR-or NF-κB-dependent genes may be affected by multiple transcription factors or be regulated indirectly by other intracellular signaling pathways during O_3_ pathogenesis. We previously demonstrated that AP-1 and c-Jun NH2-terminal kinase 1 MAPK are associated with TNFR signaling [14]. The presence of functional AP-1 binding sites in many of the TNFR-or NF-κB1-dependent genes determined in the current study, such as chemokines, cyclins, and E2F transcription factors [72,73,74], supports indirect effects or complex interplays. p38 MAPK and its upstream epidermal growth factor receptor are also known to play key roles in transcriptional activity directly and/or via crosstalk with NF-κB for inflammation and airway hyperreactivity response by O_3_ [75].

In summary, the time-dependent gene expression and pathway analyses in the current study provided important insight into the downstream molecular events during the development of multi-phasic lung injury by subacute O_3_. Comparative transcriptome analyses defined common transcriptional profiles and potential cross talk between critical O_3_-related inflammatory mediators, TNFR and NF-κB, as well as TLR4. Our results increase the understanding of the molecular mechanisms of pulmonary O_3_ toxicity for further research.

## Figures and Tables

**Table 1:** Representative lung genes time-dependently changed by ozone (O_3_) in C57BL/6J mice.

Gene Symbol	FC ^†^			Gene Title	Gene Ontology
	24 h	48 h	72 h		
*Retnla*	4.32	6.65	6.74	resistin like alpha	hormone activity
*Sprr1a*	2.26	4.47	5.56	small proline-rich protein 1A	cornified envelope/keratinization
*Lrg1*	2.29	3.20	4.12	leucine-rich alpha-2-glycoprotein 1	endothelial cell proliferation/Tgfbr binding
*Timp1*	3.40	4.45	3.94	tissue inhibitor of metalloproteinase 1	cell activation/extracellular matrix
*Tnc*	2.55	2.87	3.04	tenascin C	fibronectin binding/cell adhesion
*Areg*	1.49	2.42	2.65	amphiregulin	epidermal growth factor receptor signaling
*Lcn2*	1.51	3.21	2.38	lipocalin 2	immune system process
*Socs3*	1.91	2.45	2.32	suppressor of cytokine signaling 3	protein kinase inhibitor
*Ccl17*	1.78	2.46	2.30	chemokine (C−C motif) ligand 17	monocyte chemotaxis
*Tnfrsf12a*	1.50	2.12	2.21	tumor necrosis factor receptor superfamily, member 12a	apoptosis /cell adhesion/cell differentiation
*Mcm5*	1.21	1.83	2.13	minichromosome maintenance deficient 5, cell division cycle 46	DNA replication initiation
*Mt2*	3.25	2.84	1.97	metallothionein 2	metal ion binding
*Hist1h2ao*	−1.16	3.50	6.89	histone cluster 1, H2ao	chromatin organization
*Ccnb1*	−1.00	2.30	3.82	cyclin B1	mitotic cell cycle
*Ube2c*	1.00	2.10	3.39	ubiquitin-conjugating enzyme E2C	protein polyubiquitination
*Saa3*	1.81	2.33	1.81	serum amyloid A 3	acute-phase response
*Ch25h*	1.68	2.17	1.74	cholesterol 25-hydroxylase	lipid metabolic process/monooxygenase
*Cyp51*	1.70	2.03	1.72	cytochrome P450, family 51	lipid metabolic process/steroid biosynthetic process
*Mki67*	1.10	2.60	3.12	Ki 67 antigen	meiotic nuclear division
*Cdk1*	1.11	2.15	3.09	cyclin-dependent kinase 1	protein complex assembly/nucleotide binding
*Anln*	−1.10	2.05	2.48	anillin, actin binding protein	mitotic cytokinesis
*Top2a*	1.10	1.93	2.31	topoisomerase (DNA) II alpha	meiotic recombination intermediates
*Spp1*	1.05	2.23	2.20	secreted phosphoprotein 1	cytokine activity, extracellular
*Marco*	1.30	2.25	1.70	macrophage receptor with collagenous structure	immune system process/scavenger receptor
*Cdt1*	1.07	1.41	1.66	chromatin licensing and DNA replication factor 1	DNA replication checkpoint
*Thbs1*	2.55	2.41	1.67	thrombospondin 1	MAPK/ fibronectin binding activation
*Hspa1a*	3.62	2.32	2.58	heat shock protein 1A	telomere maintenance/ubiquitin ligase complex
*Sod2*	1.30	1.48	1.38	superoxide dismutase 2, mitochondrial	redox response
*Mup1*	−6.62	−11.08	−23.14	major urinary protein1	energy reserve metabolic process/insulin-activated receptor activity
*Igfbp3*	−1.68	−3.30	−3.86	insulin-like growth factor binding protein 3	cell growth/ fibronectin binding
*Serpina3k*	−2.08	−2.18	−2.22	serine (or cysteine) peptidase inhibitor, clade A, and member 3K	serine-type endopeptidase inhibitor
*Npr3*	−1.93	−2.82	−2.10	natriuretic peptide receptor 3	skeletal system development/adenylate cyclase
*Alb*	−1.67	−1.83	−1.87	albumin	transport/maintenance of mitochondrion location
*Thbs3*	−1.65	−2.19	−1.83	thrombospondin 3	cell adhesion/calcium ion binding
*Aox3*	−1.25	−1.15	−1.88	aldehyde oxidase 3	oxidation-reduction process
*Cyp1a1*	−1.02	−2.58	−1.70	cytochrome P450, family 1, subfamily a, polypeptide 1	monooxygenase activity
*Gzma*	−1.50	−1.64	−1.51	granzyme A	endopeptidase activity/cytolysis
*Myh11*	1.00	−1.22	−1.46	myosin, heavy polypeptide 11	muscle contraction/metabolic process

^†^ Fold change of lung genes over air controls after designated duration of 0.3 parts per million (ppm) O_3_ exposure. Negative values indicate decreased expressions and positive values indicate increased expressions. Bold values indicate significant FC. Extended list of the significantly changed genes by ozone (≥1.5 fold for at least one time point, individual moderated *t*-test, *p* < 0.01) is in Supplemental Table S1.

**Table 2:** Selected tumor necrosis factor receptor (TNFR)-dependent lung genes after air or ozone (O_3_) exposure.

Exposure	Gene Symbol	FD ^†^	Gene Title	Gene Ontology
Air	Mup *	−22.23	major urinary protein cluster (1–17)	energy reserve metabolic process, insulin-activated receptor activity
	Pttg1 *	−4.27	pituitary tumor-transforming gene 1	regulation of cell growth/DNA repair
	S100a8 *	−2.46	S100 calcium binding protein A8	leukocyte migration/calcium ion binding, calgranulin A
	Gzma *	−2.10	granzyme A	proteolysis
	*Dbp*	3.66	D site albumin promoter binding protein	transcriptional activator
	Cep104 *	1.92	centrosomal protein 104	protein binding
	*Nnt*	1.92	nicotinamide nucleotide transhydrogenase	NADPH regeneration/cell redox homeostasis
O_3_ (24|48|72 h)	*Zbtb16*	**−5.56**|1.00|−1.16	zinc finger and BTB domain containing 16	transcriptional repressor
	*Pdk4*	**−3.52**|−1.62|1.05	pyruvate dehydrogenase kinase, isoenzyme 4	mitochondrion acetyl-CoA biosynthetic process
	*Dnaja1*	**−2.44**|1.19|−1.16	DnaJ (Hsp40) homolog, subfamily A, member 1	ubiquitin ligase complex/DNA damage response
	Ccl5 *	**−1.20**|−1.95|−1.62	chemokine (C-C motif) ligand 5	chronic inflammatory response
	Rab6b *	−3.96|**−4.12**|−2.90	RAB6B, member RAS oncogene family	intra-Golgi vesicle-mediated transport
	H2-Q4	1.09|**−2.08**|−1.11	histocompatibility 2, Q region locus 4	antigen processing/presentation
	Hsph1 *	**−4.51**|**1.33**|−1.17	heat shock 105 kDa/110 kDa protein 1	nucleotide and protein binding
	Pttg1 *	−4.12|**−3.09**|**−1.84**	pituitary tumor-transforming gene 1	cellular response to DNA damage stimulus/heat shock protein binding
	*Igfbp2*	−**1.36**|**−1.47**|**−1.85**	insulin-like growth factor binding protein 2	regulation of cell growth/ response to stimulus
	*Egr1*	**4.07**|1.23|1.25	early growth response 1	transcription, DNA-templated
	*Nr4a1*	**3.72**|1.32|1.07	nuclear receptor subfamily 4, group A, member 1	endothelial cell proliferation/transcriptional activator
	*Cyr61*	**2.94**|1.10|1.04	cysteine rich protein 61	regulation of cell growth/integrin binding
	*Cd79a/b*	**2.89**|1.09|1.10	CD79A/B antigen	adaptive immune response
	*Fos*	**2.51**|1.06|1.09	FBJ osteosarcoma oncogene	regulation of transcription
	*Ch25h*	**2.32**|1.18|−1.02	cholesterol 25-hydroxylase	lipid metabolic process
	*Sprr1a*	**2.18**/1.35|−1.13	small proline-rich protein 1A	leukocyte migration
	S100a9 *	**2.34**|−1.58|−2.05	S100 calcium binding protein A9	leukocyte migration, calgranulin B
	Il6 *	**1.78**|1.17|−1.01	interleukin 6	neutrophil apoptotic process
	*Clca1*	1.13|**3.08**|1.26	chloride channel accessory 1	calcium ion transport
	*Nebl*	1.45|**2.23**|1.77	nebulette	stress fiber/cardiac muscle thin filament assembly
	Pira1 *	**2.23**|**2.03**|1.62	paired-Ig-like receptor A1	B cell homeostasis
	Chil4/3 *	**2.24**|**1.81**|1.48	chitinase-like 4/3	hydrolase/carbohydrate metabolic process
	*Timp1*	**1.79**|**1.70**|1.04	tissue inhibitor of metalloproteinase 1	negative regulation of peptidase
	*Fdps*	**1.59**|**1.76**|1.46	farnesyl diphosphate synthetase	lipid metabolic process
	*Lrp2*	**1.47**|**1.71**|1.28	low density lipoprotein receptor-related protein 2	receptor-mediated endocytosis/calcium ion binding
	*Il33*	**1.46**|**1.55**|1.36	interleukin 33	negative regulation of leukocyte migration
	*Dmkn*	1.20|**1.77**|**1.64**	dermokine	cell differentiation
	*Slc26a4*	**2.42**|**1.84**|**1.35**	solute carrier family 26, member 4	chloride transport

^†^ Fold difference of gene expression between *Tbfr^+/+^* and *Tnfr^-/-^* mice at baseline (air) and after 0.3 parts per million (ppm) O_3_ exposure. Negative values indicate a suppressed expression in *Tnfr^-/-^* compared with in *Tbfr^+/+^*, positive values indicate a heightened expression in *Tnfr^-/-^* compared with in *Tbfr^+/+^*. * Genes significantly varied between *Tnfr^+/+^* and *Tnfr^-/-^* mice both after air and O_3_ exposure. FD after O_3_ exposure in order of 24, 48, and 72 h (statistically significant changes are in bold). Full lists of the significantly varied genes between two genotypes are in Supplemental Table S3 (air; moderated *t*-test, *p* < 0.05) and S4 (O_3_; 2-way ANOVA, *p* < 0.05).

**Table 3:** Representative diseases and biological functions predicted to be affected by tumor necrosis factor receptor (TNFR)-dependent lung transcriptome changes in mice determined by Ingenuity Pathway Analysis (IPA).

Exposure	Diseases or Functions Categories	*p*-Value	Activation z-Score	Selected Associated Genes
Air	Peripheral vascular disease	9.79 × 10^−8^	1	*Alb Ccl5 Cyp1a1 Hsph1 Mmp3 Nnmt Nppa Npr3 Pde4b Rab4a S100a9 Serpina3 Stip1*
	Inflammatory response	2.63 × 10^−4^	−1.876	*Ccl5 Fgg Nfil3 Nr1d1 Nr1d2 Pde4b S100a8 S100a9 Serpina1 Serpina3 Spon2 Tnc*
24 h O_3_	Quantity of immune cells	2.23 × 10^−13^	1.041	*Abl1 Arid5b Bcl2l11 Blnk Cd22 Cd79a Cd79b Fes Il2rg Il6 Meis1 Pim1 Plcg2 Ptpn6 Sh3bp2 Spi1 Tp53*
	Immune response of leukocytes & phagocytes	5.22 × 10^−10^	2.161	*Ager Capg Ccr6 Cd38 Cd40 Cd44 Cd68 Ch25h Clec6a Cyp2s1 Dock2 Fas Fcgr1a Fn1 Grk6 Gsn Hgf Il1b Il33 Il6 Irf8 Itgb2 Lgals3 Map4k1 Pparg Rapgef3 Rgcc Rora S100a9 Scarb1 Sema4a Sh3bp2 Sirpa Socs1 Sphk1 Spi1 Tlr2 Trpv2*
	Lung permeability	3.83 × 10^−5^	−1.964	*Ccl11 Cd44 Hgf Il17a Nfe2l2 Timp2 Trpc1*
48 h O_3_	Lipid metabolism and transport	1.01 × 10^−6^	0.455	*Abcc6 Abcd1 Alb Bdnf Ces1 Ch25h Fdps Got2 Igf1 Npc1 Ptgs1 Sgms1 Slc25a13 Slco1a1 Slco1a4*
	Reactive oxygen species generation	6.32 × 10^−5^	−0.389	*Alb Casr Duoxa2 Edn1 Elane Hsd17b10 Itm2b Met Pink1, Pon1 Rac1 Sod1 Ubqln1*
	Epithelial cell spreading/shape change	1.11 × 10^−4^	2.113	*Bst1 Csf1r Flna Fn1 Gap43 Peak1 Prkch S1pr3 Tgfbi*
	Oxidation of hormones, lipids, and amino acids	1.43 × 10^−4^	−0.586	*Akr1c1 Alb Cyp1a1 Cyp1b1 Cyp2c8 Cyp3a5dao Hadh Hsd17b10 Ido1 Prodh*
	Eicosanoid synthesis/metabolism	5.48 × 10^−3^	−0.056	*Alb Alox12 Casr Clu Cotl1 Csf1 Cyp1b1 Edn1 Elavl1 Fads2 Fn1 Gpc1 Htr2b Igf1 Il1r2 Il33 Kit Mknk1 Ncam1 Nfkbia Npc1 Oprl1 Pla2g10 Pla2g2a Prkd1 Ptgs1 Rac1 S1pr3 Sod1 Stat5a*
72 h O_3_	Neurodegeneration	5.93 × 10^−5^	−1.179	*App Bdnf Clu Epor Ets2 Fgfr1 Grid2 Hpca Kcnma1 Mag Man2c1 Ndufs4 Nmnat1 Nos1 Nr4a3 Pax8 Plp1 Psap Scarb2 Serpinf2 Slc1a1 Slc1a3 Slc26a4 Sox10.*
	Efflux of amino acids	3.27 × 10^−4^	−1.949	*Adora2a Nos1 Alb Kncj9 Nos1 Slc1a1 Slc1a3 Slc22a13 Slco1a4 Ttr*

**Table 4:** Selected nuclear factor of kappa light polypeptide gene enhancer in B-cells p50 (NF-κB1)-dependent lung genes after air or ozone (O_3_) exposure.

Exposure	Gene Symbol	FD ^†^	Gene Title	Gene Ontology
Air	*Jchain*	−20.03	immunoglobulin joining chain	adaptive immune response
	*Trim12a*	−6.99	tripartite motif-containing 12A	metal ion binding
	*Cxcl13*	−4.26	chemokine (C-X-C motif) ligand 13	lymphocyte chemotaxis
	*Pcdhb3*	−3.33	protocadherin beta 3	cell adhesion
	*Marco*	−2.58	macrophage receptor with collagenous structure	immune system process
	*Reg3g*	25.77	regenerating islet-derived 3 gamma	MyD88-dependent toll-like receptor signaling pathway
	*Ifi44l*	8.28	interferon-induced protein 44 like	immune response
	*Saa3*	6.20	serum amyloid A 3	acute-phase response
	*Cd209a*	4.01	CD209a antigen	viral entry into host cell
	*Irf7*	3.34	interferon regulatory factor 7	immune system process
	*Ifit1*	2.32	interferon-induced protein with tetratricopeptide repeats 1	immune system process
	*Ccl5*	2.03	chemokine (C-C motif) ligand 5	positive regulation of defense response to virus by host
	*Cd14*	*1.61*	CD14 antigen	immune system process
48 h Ozone (O_3_)	*Psca*	−10.87	prostate stem cell antigen	actin binding
	*Fbp2*	−5.70	fructose bisphosphatase 2	carbohydrate metabolic process
	Pttg1 *	−2.81	pituitary tumor-transforming gene 1	cellular response to DNA damage stimulus/heat shock protein binding
	*Sprr1a*	−2.52	small proline-rich protein 1A	leukocyte migration
	*Ccnb1*	−2.44	cyclin B1	mitotic cell cycle
	*Hist1h2ao*	−2.42	histone cluster 1, H2ao	chromatin organization
	*Gclc*	−2.38	glutamate-cysteine ligase, catalytic subunit	glutathione metabolic process
	Dbp *	−2.27	D site albumin promoter binding protein	transcriptional activator
	*Cdk1*	−2.18	cyclin-dependent kinase 1	mitotic nuclear division
	*Ccl17*	−2.16	chemokine (C-C motif) ligand 17	chemotaxis
	S100a8 *	−2.46	S100 calcium binding protein A8	leukocyte migration/calcium ion binding, calgranulin A
	*Cdca8*	−1.98	cell division cycle associated 8	mitotic sister chromatid segregation
	*Edn1*	−1.96	endothelin 1	negative regulation of transcription from RNA polymerase II promoter
	*Ccl22*	−1.96	chemokine (C-C motif) ligand 22	chemotaxis
	*Marco*	−1.86	macrophage receptor with collagenous structure	immune system process/scavenger receptor
	Iigp1 *	7.39	interferon inducible GTPase 1	immune system process
	Nlrc5 *	5.08	NLR family, CARD domain containing 5	negative regulation of NF-kappa B transcription factor activity
	Gzmb *	5.07	granzyme B	T cell mediated cytotoxicity
	*Nkg7*	4.25	natural killer cell group 7 sequence	DNA binding
	Ifi47 *	4.24	interferon gamma inducible protein 47	defense response
	*Gbp2*	3.59	guanylate binding protein 2	cellular response to interferon-beta
	Oas1a *	3.10	2′-5′ oligoadenylate synthetase 1A	immune system process
	Stat1 *	3.08	signal transducer and activator of transcription 1	negative regulation of transcription from RNA polymerase II promoter
	Psmb9 *	2.80	proteasome (prosome, macropain) subunit, beta type 9 (large multifunctional peptidase 2)	immune system process
	Tap1 *	2.90	transporter 1, ATP-binding cassette, sub-family B (MDR/TAP)	adaptive immune response
	Ncr1 *	1.74	natural cytotoxicity triggering receptor 1	defense response to virus

^†^ Fold difference of lung gene expressions between *Nfkb1^+/+^* (B6129PF2/J) and *Nfkb1^-/-^* (B6;129PNfkb1^tm1Bal^/J) mice at baseline (air) and after 48 h of 0.3-parts per million O_3_ exposure. Negative values indicate a lowered expression in *Nfkb1^-/-^* than in *Nfkb1^+/+^*, positive values indicate a heightened expression in *Nfkb1^-/-^* than in *Nfkb1^+/+^*. Full lists of the significantly varied genes between two negative values indicate genotypes determined by moderated *t*-test or two-way ANOVA (*p* < 0.05, ≥1.5 FD) are available in Supplemental Tables S5 and S6. * Genes significantly varied between *Nfkb1^+/+^* and *Nfkb1^-/-^* mice after air and O_3_ exposure.

**Table 5:** Potential NF-κB binding motifs on selected *Nfkb1*-dependently regulated lung genes after ozone (O_3_) exposure.

Accession	Gene Symbol	Gene Title	Position * & Motif Orientation	Binding Sequence
NM_028216	*Psca* ^†^	prostate stem cell antigen	−7506 F (chr15: 74537552/74537762)	AGGGATGCCC (74537547-74537556)
NM_010104	*Edn1* ^†^	endothelin 1	+5601 R (chr13: 42402568/42402446)	TGTGGAATCTCCTG (42402561-42402574)
NM_028149	*Fbxl20* ^†^	F-box and leucine-rich repeat protein 20	−7394 F (chr11: 98018224/98018324)	GGGGACTTCCCC (98018218-98018229)
			−8408 F (chr11: 98019575/98019338)	ATGGGACCCCCCGG (98019568-98019581)
NM_001025427	*Hmga1* ^†^	high mobility group AT-hook 17.2	−9191 R (chr17: 27684368/27701127)	AGGGACTTCCCT (27684362-27684373)
			+7600 F (chr17: 27700556/27701127)	GGGGCTTCCT (27700551-27700560)
			+8603 R (chr17: 27702173/27702130)	GCGGAGCCCC (27702168-27702177)
NM_011121	*Plk1* ^†^	polo-like kinase 1	−11380 F (chr7: 129291587/129291572)	GGGGAGCTCC (129291582-129291591)
			+8579 R (chr7: 129311120/129311530)	GGGGGTTCTCCA (129311114-129311125)
NM_016671	*Il27ra* ^†^	interleukin 27 receptor, alpha	−516 R (chr8: 86566513/86566990)	GGGACTTCCCG (86566507-86566518)
NM_016974	*Dbp*	D site albumin promoter binding protein	+712 R (chr7: 52961056/52955496)	GGGGGGCCCC (52961051-52961060)
			−2500 R (chr7: 52957884/52958102)	GGGGACCCCC (52957879-52957888)
			−5106 R (chr7: 52955545/52955496)	CGGGAGCCCC (52955540-52955549)
NM_017376	*Tef* ^†^	NM_017376.2	na R (chr15: 81642127/na)	GGGGGTTCTCCA (81642121-81642132)
NM_026785	*Ube2c*	ubiquitin-conjugating enzyme E2C	2452 R (chr2: 164597717/164597881)	GGGGTTTTTCCA (164597711-164597722)
NM_011332	*Ccl17*	chemokine (C-C motif) ligand 17	−2620 F (chr8: 97331526/97331749)	GGGGAGTTTCCA (97331520-97331531)
			−3542 R (chr8: 97330838/97330827)	TGGGGACCTTCCA (97330832-97330844)
NM_009137	*Ccl22*	chemokine (C-C motif) ligand 22	−506 F (chr8: 97269386/97268984)	GGGGACTTTACA (97269380-97269391)
NM_011369.2	*Shcbp1*	Shc SH2-domain binding protein 1	+287 F (chr8: 4779209/4779247)	GGGAATGTCC (4779204-4779213)
NM_001286404	*Ghrl*	ghrelin	na R (chr6: 113659936/na)	GGGGCTGCCC (113659931-113659940)

DECODE database search for NF-κB1/NF-κB binding consensus sequence (5′-GGGRNYYYCC-3′ or 5′-GGAATTYCCC-3′; R is purine A/G, Y is a pyrimidine T/C, N is any nucleotide) validated by chromatin immunoprecipitation (ChIP)-qPCR from −20 kb to + 10 kb relative to the transcription start site (SAbiosciences.com, http://www.sabiosciences.com/chipqpcrsearch.php?species_id=1&factor=NF-kappaB1&gene=HSPA1A&nfactor=n&ninfo=n&ngene=n&B2=Search (accessed on 30 July 2021), * NCBI Mus musculus Build Number: 37 Version 1. ^†^ Common regulated genes by TNFR and NF-κB1. FC = fold change. FD = fold difference in *Nfkb1^-/-^* mice vs. *Nfkb1^+/+^* mice. F = forward. R = reverse. na = not available. †Common differentially regulated genes in *Tnfr^-/-^* and *Nfkb1^-/-^* lungs.

## Data Availability

Microarray data (Gene Expression Omnibus accession numbers: GSE166399, GSE166398) are deposited in a public database repository. Data is contained within the article or supplementary material.

## Supplementary Materials

The following are available online at https://www.mdpi.com/article/10.3390/antiox10091489/s1.
