# Supplementary material for: Transcriptomics Underlying Pulmonary Ozone Pathogenesis Regulated by Inflammatory Mediators in Mice
Source: Antioxidants (Basel). 2021 Sep 18;10(9):1489. doi: 10.3390/antiox10091489 (PMC8466999; doi:10.3390/antiox10091489)
Supplement: Supplementary file 1 [file antioxidants-10-01489-s001.zip › antioxidants-1340817-supplementary.pdf]

Figure S1

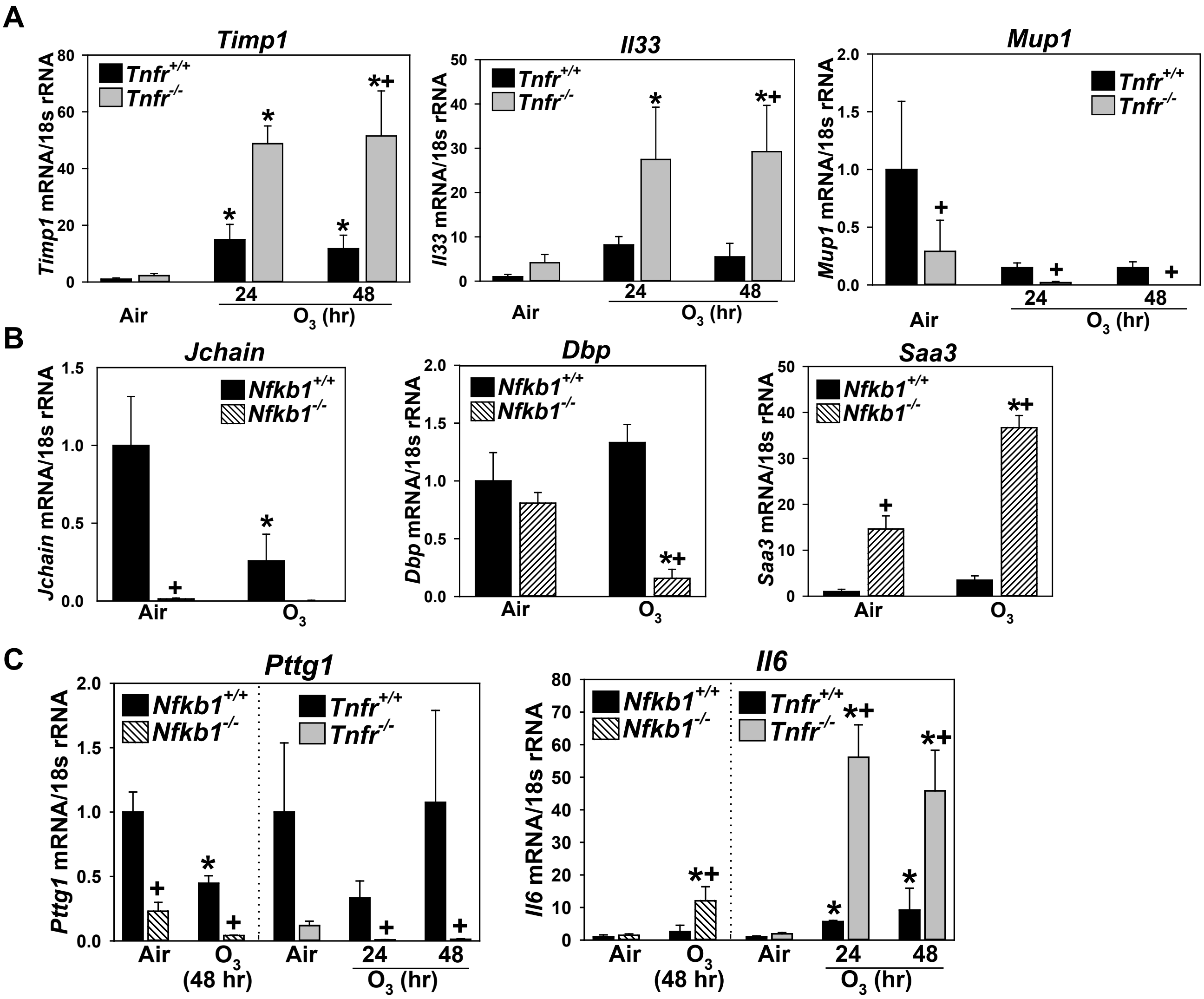

Figure S1. Microarray analysis validation of tumor necrosis factor receptor (TNFR)- and NF-κB1-dependent transcriptomics in response to ozone (O_3) by qRT-PCR. (A) Differential transcript levels of TNFR-dependent tissue inhibitor of metalloproteinase 1 (*Timp1*), interleukin-33 (*Il33*) and major urinary protein 1 (*Mup1*) in *Tnfr*-deficient (*Tnfr*$^{-/-}$) and wild-type (*Tnf*$^{+/+}$) mouse lungs by qRT-PCR. (B) Differential transcript levels of NF-κB1 (p50)-dependent immunoglobulin joining chain (*Jchain*), D site albumin promoter binding protein (*Dbp*) and serum amyloid A3 (*Saa3*) in *Nfkb1*-deficient (*Nfkb1*$^{-/-}$) and wild-type (*Nfkb1*$^{+/+}$) mouse lungs by qRT-PCR. (C) qRT-PCR also determined expression of common differentially regulated genes, pituitary tumor-transforming gene 1 (*Pttg1*) and *Il6*, between *Tnfr*$^{-/-}$ and *Tnf*$^{+/+}$ mice and *Nfkb1*$^{-/-}$ and *Nfkb1*$^{+/+}$ mice. 18s rRNA-normalized group means $\pm$ S.E.M presented (n = 3/group). *, significantly different from genotype-matched air control mice ($p < 0.05$). +, significantly different from O_3-exposed wild-type mice ($p < 0.05$).

A

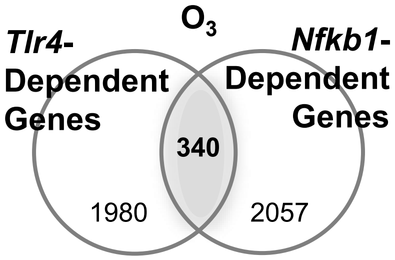

B

Commonly Gene Disease & Functions	Gene Symbol	Gene Title
**Lipid metabolism**	*Dbp*<br>*Cpt1a*<br>*Tef*<br>*Txnip*<br>*Ctfr*	D site albumin promoter binding protein<br>carnitine palmitoyltransferase 1a, liver<br>thyrotroph embryonic factor<br>thioredoxin interacting protein<br>cystic fibrosis transmembrane conductance regulator
**Cell-mediated immunity & phagocytosis**	*Bhlhe40*<br>*Cxcl1*<br>*Ccl20*<br>*Arntl*<br>*Marco*	basic helix-loop-helix family, member e40<br>chemokine (C-X-C motif) ligand 1<br>chemokine (C-C motif) ligand 20<br>aryl hydrocarbon receptor nuclear translocator-like<br>macrophage receptor with collagenous structure
**Inflammation and lymphoid tissue hyperplasia**	*Lcn2*<br>*Ptpn2*<br>*Casp8*<br>*Ncr1*<br>*Saa3*	lipocalin 2<br>protein tyrosine phosphatase, non-receptor type 2<br>caspase 8<br>natural cytotoxicity triggering receptor<br>serum amyloid A3

C

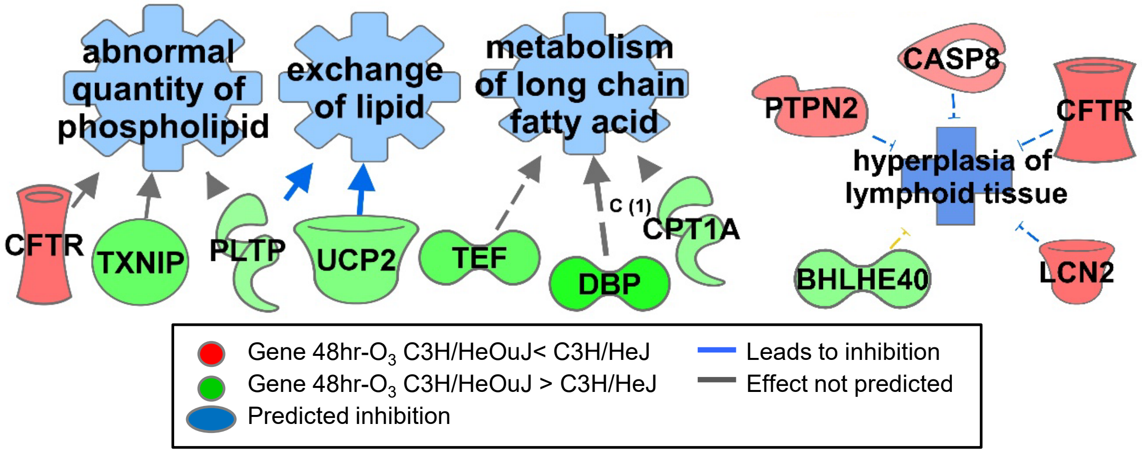

Figure S2. Common differentially regulated lung genes by toll-like receptor 4 (TLR4) and NF-κB1 in response to ozone (O_3). (A) Venn Diagram analysis determined common O_3-responsive genes differentially expressed between C3H/HeOuJ (*Tlr4*-normal) - C3H/HeJ (*Tlr4*-mutated) and wild-type (*Nfkb1*$^{+/+}$) - *Nfkb1*-deficient (*Nfkb1*$^{-/-}$) mice (n=340). (B) Pathway analysis determined functions and pathways of the common genes. (C) Common genes enriched to fatty acid metabolism included *Dbp* and *Cpt1a* and to cell-mediated immunity and inflammation included *Lcn2*, Ccl20, and *Casp8*. Molecule colors indicated relative expression levels in C3H/HeJ mice compared to C3H/HeOuJ mice after 48 hr exposure to 0.3-parts per million O_3. Analyses were done using Ingenuity Pathway Analysis and GeneSpring software.

Figure S3

A

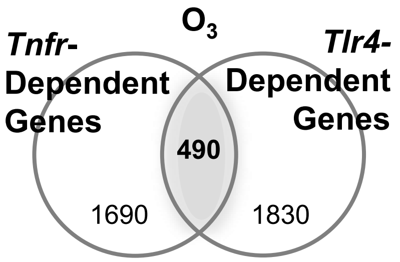

B

Commonly Gene Disease & Functions	Gene Symbol	Gene Title
**Engulfment of phagocytes**	*Marco*<br>*Il33*<br>*S100a8*<br>*Icam1*	macrophage receptor with collagenous structure<br>interleukin 33<br>S100 calcium binding protein A8 (calgranulin A)<br>intercellular adhesion molecule 1
**Protein ubiquitination**	*Dnaja1*<br>*Hspd1*<br>*Hsph1*<br>*Psma3*<br>*Psmc1*	Dnaj heat shock protein family (Hsp40) member 1<br>heat shock protein family D (Hsp60) member 1<br>heat shock protein family H (Hsp110) member 1<br>proteasome 20S subunit alpha 3<br>proteasome 26S subunit, ATPase 1
**Fatty acid metabolism**	*Acot7*<br>*Ptges*<br>*Plpp3*<br>*Elovl1*<br>*Lpin1*	acyl-CoA thioesterase 7<br>prostaglandin E synthase<br>phosphlipid phosphatase 3<br>ELOVL family member 1, elongation of long chain fatty acids<br>lipin 1
**Glutathione homeostasis/Redox**	*Gltx*<br>*Gstm1*<br>*Gsr*	glutaredoxin<br>glutathione S-transferase, mu 1<br>glutathione reductase

C

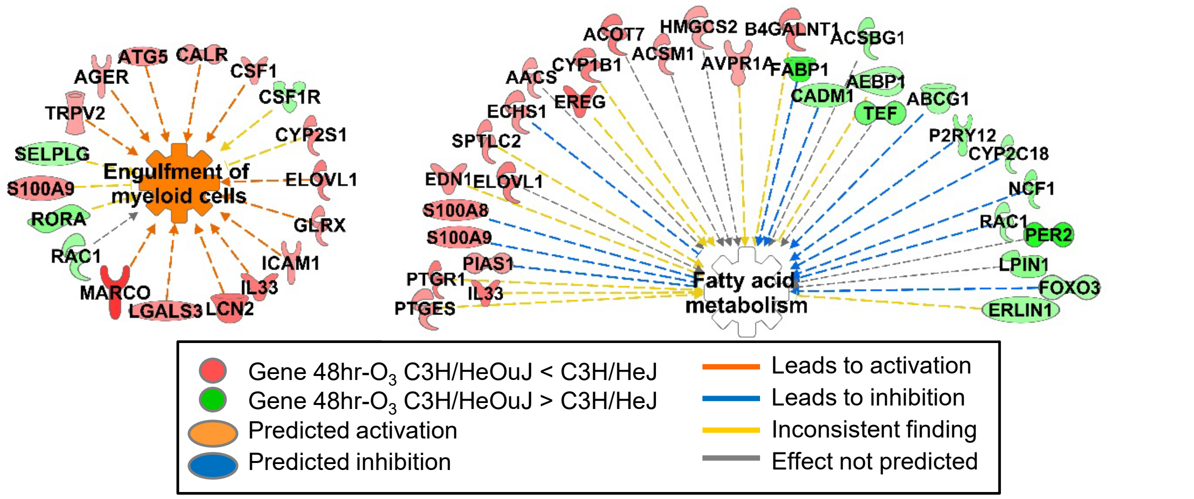

Figure S3. Common differentially regulated lung genes by tumor necrosis factor receptor (TNFR) and toll-like receptor 4 (TLR4) in response to ozone (O_3). (A) Venn Diagram analysis determined common O_3-responsive genes differentially expressed between wild-type (*Tnfr*$^{+/+}$) - *Tnfr*-deficient (*Tnfr*$^{-/-}$) mice and C3H/HeOuJ (*Tlr4*-normal) - C3H/HeJ (*Tlr4*-mutated) mice (n=490). (B-C) Pathway analysis determined top functions and pathways of the common genes. Common genes enriched to phagocytosis included *Marco*, *Il33*, and *Icam1,* to protein ubiquitination included *Dnaja1*, *Hspd1*, and *Psma3*, and to fatty acid metabolism included *Acot7, Ptges*, and *Elovl1*. Molecule colors indicated relative expression levels in C3H/HeJ mice compared to C3H/HeOuJ mice after 48 hr exposure to 0.3-parts per million O_3. Analyses were done using Ingenuity Pathway Analysis and GeneSpring software.

Table S1. Time-dependent changes in lung genes by 0.3-parts per million (ppm) ozone (O_3) exposure in C57BL/6J mice.

Data arranged by fold change (FC) at 72 hr. >1.5 FC at least one time point displayed.

Bold font-significant change (p < 0.01 with moderated t-test).

RefSeq Transcript ID	p (24h n=460)	p (48h n=657)	p (72h n=805)	FC 24h	FC 48h	FC 72h	Gene Symbol	Gene Title	Gene Ontology Biological Process
NM_001177544///NM_178185		1.00E-05	4.27E-05	-1.16	**3.50**	**6.89**	***Hist1h2ao///Hist1h2ap***	histone cluster 1, H2ao///histone cluster 1, H2ap	0006325 // chromatin organization // not recorded///0006342 // chromatin silencing // not recorded///0008285 // negative regulation of cell proliferation // not recorded
NM_020509	8.10E-06	3.30E-07	2.00E-07	**4.32**	**6.65**	**6.74**	***Retnla***	resistin like alpha	0005179 // hormone activity // inferred from electronic annotation
NM_009264	1.38E-04	4.10E-05	1.01E-05	**2.26**	**4.46**	**5.56**	***Sprr1a***	small proline-rich protein 1A	0018149 // peptide cross-linking // not recorded///0030216 // keratinocyte differentiation // not recorded///0031424 // keratinization // inferred from electronic annotation
NM_029796	1.43E-05	2.85E-07	5.28E-05	**2.29**	**3.20**	**4.12**	***Lrg1***	leucine-rich alpha-2-glycoprotein 1	0001938 // positive regulation of endothelial cell proliferation // inferred from mutant phenotype///0030511 // positive regulation of transforming growth factor beta receptor signali
NM_001044384///NM	7.60E-07	1.43E-07	8.61E-08	**3.40**	**4.45**	**3.94**	***Timp1***	tissue inhibitor of metalloproteinase 1	0001775 // cell activation // not recorded///0007568 // aging // inferred from electronic annotation///0008284 // positive regulation of cell proliferation // not recorded///0009725 /
NM_172301///XM_01	1.60E-04	1.22E-04	1.99E-04	**-1.00**	**2.30**	**3.82**	***Ccnb1///Gm5593***	cyclin B1///predicted gene 5593	0000278 // mitotic cell cycle // inferred from electronic annotation///0001556 // oocyte maturation // not recorded///0001701 // in utero embryonic development // inferred from r
NM_026785		5.76E-06	3.03E-05	-1.03	**2.10**	**3.39**	***Ube2c***	ubiquitin-conjugating enzyme E2C	0000209 // protein polyubiquitination // not recorded///0006511 // ubiquitin-dependent protein catabolic process // not recorded///0007049 // cell cycle // inferred from electronic
NM_009104		1.42E-04	8.35E-06	1.13	**2.50**	**3.13**	***Rrm2***	ribonucleotide reductase M2	0006260 // DNA replication // inferred from electronic annotation///0009186 // deoxyribonucleoside diphosphate metabolic process // inferred from electronic annotation///00092€
NM_001081117///XM_006507411//,		9.76E-05	6.36E-05	1.10	**2.60**	**3.12**	***Mki67***	antigen identified by monoclonal antibody Ki 67	0006259 // DNA metabolic process // inferred from electronic annotation///0007126 // meiotic nuclear division // inferred from direct assay///0008283 // cell proliferation // inferrec
NM_007659		3.11E-04	3.34E-05	1.11	**2.15**	**3.09**	***Cdk1***	cyclin-dependent kinase 1	0006461 // protein complex assembly // not recorded///0006468 // protein phosphorylation // inferred from direct assay///0006915 // apoptotic process // inferred from electronic
NM_011607///XM_00	7.19E-05	4.07E-04	8.78E-04	**2.55**	**2.87**	**3.04**	***Tnc***	tenascin C	0001649 // osteoblast differentiation // not recorded///0007155 // cell adhesion // inferred from electronic annotation///0007162 // negative regulation of cell adhesion // not recor
NM_001012272///NM_001012273//		5.06E-06	5.11E-04	-1.02	**2.76**	**3.03**	***Birc5***	baculoviral IAP repeat-containing 5	0000086 // G2/M transition of mitotic cell cycle // not recorded///0000226 // microtubule cytoskeleton organization // inferred from mutant phenotype///0000910 // cytokinesis // r
NM_023209///XM_006519257		1.35E-04	6.78E-06	1.12	**1.86**	**2.74**	***Pbk***	PDZ binding kinase	0001933 // negative regulation of protein phosphorylation // inferred from mutant phenotype///0006468 // protein phosphorylation // inferred from electronic annotation///001631
NM_009704	0.00238687	8.16E-05	0.00138973	**1.49**	**2.42**	**2.65**	***Areg***	amphiregulin	0007173 // epidermal growth factor receptor signaling pathway // not recorded///0007186 // G-protein coupled receptor signaling pathway // not recorded///0008284 // positive reg
NM_001177625///NM_001177626//	0.003232483	0.00476644		1.08	**1.66**	**2.64**	***Ect2***	ect2 oncogene	0000902 // cell morphogenesis // inferred from direct assay///0000910 // cytokinesis // not recorded///0006810 // transport // inferred from electronic annotation///0007049 // cell
NM_010479	0.00128837	0.002790963		**3.62**	**2.32**	2.58	***Hspa1a***	heat shock protein 1A	0000723 // telomere maintenance // inferred from mutant phenotype///0006281 // DNA repair // inferred from mutant phenotype///0006402 // mRNA catabolic process // inferred
NM_016904		1.86E-05	1.42E-05	1.23	**2.12**	**2.53**	***Cks1b***	CDC28 protein kinase 1b	0007049 // cell cycle // inferred from electronic annotation///0045859 // regulation of protein kinase activity // inferred from electronic annotation///0051301 // cell division // infer
NM_028390///XM_006510581///XM	0.001230148	7.72E-05		-1.10	**2.05**	**2.48**	***Anln***	anillin, actin binding protein	0000281 // mitotic cytokinesis // not recorded///0002244 // hematopoietic progenitor cell differentiation // inferred from genetic interaction///0007049 // cell cycle // inferred from
NM_023223		2.50E-05	0.00114813	1.01	**1.65**	**2.48**	***Cdc20***	cell division cycle 20	0007049 // cell cycle // inferred from electronic annotation///0007067 // mitotic nuclear division // inferred from electronic annotation///0007399 // nervous system development //
NM_009828		2.75E-04	1.04E-05	1.04	**1.74**	**2.47**	***Ccna2***	cyclin A2	0000079 // regulation of cyclin-dependent protein serine/threonine kinase activity // inferred from electronic annotation///0007049 // cell cycle // inferred from electronic annotatior
NM_007630		3.69E-06	4.54E-05	-1.12	**1.77**	**2.43**	***Ccnb2***	cyclin B2	0001701 // in utero embryonic development // inferred from mutant phenotype///0007049 // cell cycle // inferred from electronic annotation///0007067 // mitotic nuclear division /
NM_025415		0.002650347	3.27E-04	-1.02	**1.76**	**2.39**	***Cks2***	CDC28 protein kinase regulatory subunit 2	0007049 // cell cycle // inferred from electronic annotation///0007127 // meiosis I // inferred from mutant phenotype///0016310 // phosphorylation // inferred from electronic anno
NM_001291892///NM_001291893//		0.001058841	4.70E-06	1.34	**1.84**	**2.39**	***Lilr4b///Lilrb4a***	leukocyte immunoglobulin-like receptor, subfamily B, member 4B/	0002250 // adaptive immune response // inferred from electronic annotation///0002376 // immune system process // inferred from electronic annotation
NM_008491	4.21E-04	2.89E-06	4.51E-04	**1.51**	**3.21**	**2.38**	***Lcn2***	lipocalin 2	0002376 // immune system process // inferred from electronic annotation///0006810 // transport // inferred from electronic annotation///0006811 // ion transport // inferred from
NM_001190320///NM_001190321//		4.70E-04	8.31E-04	1.20	**1.92**	**2.37**	***Clec4n***	C-type lectin domain family 4, member n	0002250 // adaptive immune response // inferred from electronic annotation///0002376 // immune system process // inferred from electronic annotation///0043123 // positive regu
NM_001163525///NM	0.00101274	2.19E-05	9.57E-04	**1.52**	**2.15**	**2.32**	***S100a14***	S100 calcium binding protein A14	0006915 // apoptotic process // inferred from electronic annotation///0032496 // response to lipopolysaccharide // inferred from electronic annotation///0034142 // toll-like recepto
NM_016748///XM_00	7.70E-05	1.40E-05	2.08E-05	**1.81**	**2.31**	**2.32**	***Ctps***	cytidine 5'-triphosphate synthase	0002376 // immune system process // inferred from electronic annotation///0006221 // pyrimidine nucleotide biosynthetic process // inferred from electronic annotation///0006241
NM_007707///XM_01	2.11E-05	5.02E-06	7.01E-06	**1.91**	**2.45**	**2.32**	***Socs3***	suppressor of cytokine signaling 3	0001666 // response to hypoxia // inferred from electronic annotation///0001932 // regulation of protein phosphorylation // inferred from direct assay///0006469 // negative regulat
NM_011623///XM_006533153///XM		6.59E-04	4.81E-04	1.10	**1.93**	**2.31**	***Top2a***	topoisomerase (DNA) II alpha	0000712 // resolution of meiotic recombination intermediates // not recorded///0000819 // sister chromatid segregation // not recorded///0002244 // hematopoietic progenitor cell
NM_011332///XM_00	6.54E-05	1.55E-06	3.36E-05	**1.78**	**2.46**	**2.30**	***Ccl17***	chemokine (C-C motif) ligand 17	0002548 // monocyte chemotaxis // ---///0006935 // chemotaxis // traceable author statement///0006954 // inflammatory response // ---///0006955 // immune response // inferrec
NM_011817	0.004028	0.00107945	0.00692808	**1.41**	**1.61**	**2.28**	***Gadd45g***	growth arrest and DNA-damage-inducible 45 gamma	0000185 // activation of MAPKKK activity // not recorded///0000186 // activation of MAPKK activity // inferred from direct assay///0006469 // negative regulation of protein kinase a
NM_001164362///NM_028293///NM		9.22E-04	1.54E-04	1.03	**1.69**	**2.23**	***Cep55***	centrosomal protein 55	0000281 // mitotic cytokinesis // not recorded///0000920 // cell separation after cytokinesis // not recorded///0007049 // cell cycle // inferred from electronic annotation///0007067
NM_001161746///NM	8.66E-05	3.96E-04	1.50E-04	**1.50**	**2.12**	**2.21**	***Tnfrsf12a***	tumor necrosis factor receptor superfamily, member 12a	0001525 // angiogenesis // inferred from electronic annotation///0006915 // apoptotic process // inferred from electronic annotation///0006931 // substrate-dependent cell migratic
NM_001204201///NM_001204202//		5.33E-06	8.69E-04	1.05	**2.26**	**2.19**	***Spp1***	secreted phosphoprotein 1	0001503 // ossification // inferred from electronic annotation///0001649 // osteoblast differentiation // not recorded///0006954 // inflammatory response // inferred from electronic
NM_011867///XM_006515091///XM		4.86E-05	3.99E-05	1.52	**1.78**	**2.17**	***Slc26a4***	solute carrier family 26, member 4	0006810 // transport // inferred from electronic annotation///0006821 // chloride transport // not recorded///0006885 // regulation of pH // inferred from mutant phenotype///000
NM_010664	5.61E-04	2.40E-05	9.83E-07	**1.26**	**1.73**	**2.15**	***Krt18***	keratin 18	0033209 // tumor necrosis factor-mediated signaling pathway // inferred from genetic interaction///0043000 // Golgi to plasma membrane CFTR protein transport // not recorded///
NM_009369	0.00269734	3.06E-04	7.07E-06	**1.25**	**1.75**	**2.14**	***Tgfbi***	transforming growth factor, beta induced	0001525 // angiogenesis // inferred from electronic annotation///0002062 // chondrocyte differentiation // inferred from electronic annotation///0007155 // cell adhesion // inferrec
NM_001302540///NM	0.00406378	5.53E-06	5.64E-04	**1.21**	**1.83**	**2.13**	***Mcm5***	minichromosome maintenance deficient 5, cell division cycle 46 (S.	0006260 // DNA replication // inferred from electronic annotation///0006270 // DNA replication initiation // inferred from electronic annotation///0007049 // cell cycle // inferred fro
NM_010171	0.00202076	6.24E-04	0.00170283	**1.86**	**2.41**	**2.12**	***F3***	coagulation factor III	0001938 // positive regulation of endothelial cell proliferation // not recorded///0002541 // activation of plasma proteins involved in acute inflammatory response // not recorded///
NM_011234		3.50E-04	5.35E-05	1.05	**1.61**	**2.11**	***Rad51***	RAD51 homolog	0000724 // double-strand break repair via homologous recombination // inferred from direct assay///0000724 // double-strand break repair via homologous recombination // inferre
NM_145126///NM_009892		0.006972907	0.0053045	-1.17	**2.06**	**2.07**	***Chil4///Chil3***	chitinase-like 4///chitinase-like 3	0005975 // carbohydrate metabolic process // inferred from electronic annotation///0006032 // chitin catabolic process // not recorded///0006954 // inflammatory response // infer
NM_009344///XR_871	4.17E-04	9.76E-04	0.00103555	**1.63**	**2.42**	**2.06**	***Phlda1***	pleckstrin homology-like domain, family A, member 1	0006915 // apoptotic process // inferred from electronic annotation///0021879 // forebrain neuron differentiation // inferred from electronic annotation///0043065 // positive regula
NM_001309637///NM_020008///NR		0.007199291	0.00377891	1.16	**2.05**	**2.06**	***Clec7a***	C-type lectin domain family 7, member a	0001878 // response to yeast // inferred from direct assay///0001879 // detection of yeast // inferred from direct assay///0002221 // pattern recognition receptor signaling pathway /
NM_001161714///NM	0.00125905	3.45E-05	1.39E-04	**1.47**	**2.12**	**2.05**	***Tgm1***	transglutaminase 1, K polypeptide	0009887 // organ morphogenesis // inferred from mutant phenotype///0018149 // peptide cross-linking // inferred from electronic annotation///0019538 // protein metabolic proces
NM_010415	6.54E-05	0.00113656	2.19E-06	**1.34**	**1.51**	**2.03**	***Hbegf***	heparin-binding EGF-like growth factor	0001525 // angiogenesis // non-traceable author statement///0001832 // blastocyst growth // traceable author statement///0007173 // epidermal growth factor receptor signaling p
NM_008012	4.52E-05	5.10E-05	1.34E-05	**1.61**	**2.03**	**2.01**	***Akr1b8***	aldo-keto reductase family 1, member B8	0016488 // farnesol catabolic process // not recorded///0044597 // daunorubicin metabolic process // not recorded///0044598 // doxorubicin metabolic process // not recorded///0
NM_001110101///NM_001204273//		3.19E-04		**1.19**	**1.69**	**2.00**	***Lsm2***	LSM2 homolog, U6 small nuclear RNA associated (S. cerevisiae)	0000244 // spliceosomal tri-snRNP complex assembly // not recorded///0000398 // mRNA splicing, via spliceosome // not recorded///0006397 // mRNA processing // inferred from e
NM_011369		6.27E-05	2.89E-06	1.03	**1.63**	1.99	***Shcbp1***	Shc SH2-domain binding protein 1	0008543 // fibroblast growth factor receptor signaling pathway // inferred from mutant phenotype///2000177 // regulation of neural precursor cell proliferation // inferred from muta
NM_031170	0.00207908	5.74E-04	1.35E-04	1.26	**1.92**	**1.98**	***Krt8***	keratin 8	0000904 // cell morphogenesis involved in differentiation // traceable author statement///0007275 // multicellular organismal development // traceable author statement///003320!
NM_008630	2.85E-06	6.98E-05	1.13E-04	**3.25**	**2.84**	**1.97**	***Mt2***	metallothionein 2	0006882 // cellular zinc ion homeostasis // inferred from mutant phenotype///0007263 // nitric oxide mediated signal transduction // inferred from mutant phenotype///0010273 //
NM_183249		0.003977341	7.17E-05	-1.00	**1.74**	**1.96**	***Wfdc21***	WAP four-disulfide core domain 21	0010466 // negative regulation of peptidase activity // inferred from electronic annotation///0032496 // response to lipopolysaccharide // inferred from direct assay///0034612 // res
NM_001166406///NM	1.84E-04		1.59E-05	**-1.04**	1.25	**1.95**	***Kif20a***	kinesin family member 20A	0000281 // mitotic cytokinesis // not recorded///0000910 // cytokinesis // not recorded///0000920 // cell separation after cytokinesis // not recorded///0001578 // microtubule bunc
NM_010478	0.00991306			**2.15**	1.48	1.95	***Hspa1b***	heat shock protein 1B	0007339 // binding of sperm to zona pellucida // inferred from direct assay///0009408 // response to heat // inferred from direct assay///0043066 // negative regulation of apoptotic
NM_011121		4.59E-05	3.09E-05	1.05	**1.55**	**1.95**	***Plk1***	polo-like kinase 1	0000070 // mitotic sister chromatid segregation // not recorded///0000086 // G2/M transition of mitotic cell cycle // not recorded///0000122 // negative regulation of transcription fr
NM_001081088	5.66E-04	1.81E-04	2.28E-04	**1.36**	**1.72**	**1.92**	***Lrp2***	low density lipoprotein receptor-related protein 2	0003281 // ventricular septum development // inferred from mutant phenotype///0006766 // vitamin metabolic process // inferred from mutant phenotype///0006897 // endocytos
NM_018754	0.00146954	0.003028516	0.00477337	**1.55**	**1.93**	**1.90**	***Sfn***	stratifin	0000079 // regulation of cyclin-dependent protein serine/threonine kinase activity // inferred from direct assay///0001836 // release of cytochrome c from mitochondria // not recor
NM_172301///XM_011244665///XR_		0.001493795	1.32E-04	1.01	**1.38**	**1.89**	***Ccnb1///Gm5593***	cyclin B1///predicted gene 5593	0000278 // mitotic cell cycle // inferred from electronic annotation///0001556 // oocyte maturation // not recorded///0001701 // in utero embryonic development // inferred from r
NM_009892///NM_145126		0.002987196	0.00189448	-1.10	**1.93**	**1.89**	***Chil3///Chil4***	chitinase-like 3///chitinase-like 4	0000272 // polysaccharide catabolic process // inferred from electronic annotation///0005975 // carbohydrate metabolic process // inferred from electronic annotation///0006032 /
NM_001171187///NM	0.00543237	6.84E-05	4.24E-05	**1.19**	**1.57**	**1.88**	***Mal***	myelin and lymphocyte protein, T cell differentiation protein	0042552 // myelination // not recorded///1902043 // positive regulation of extrinsic apoptotic signaling pathway via death domain receptors // inferred from direct assay
NM_033075///XR_871	2.03E-04	0.003982373		**1.34**	**1.65**	1.88	***D17H6S56E-5***	DNA segment, Chr 17, human D6S56E 5	0019058 // viral life cycle // inferred from electronic annotation
NM_001205339///NM_177420		0.002927174	0.00185353	1.22	**1.71**	**1.87**	***Psat1***	phosphoserine aminotransferase 1	0006564 // L-serine biosynthetic process // inferred from electronic annotation///0008152 // metabolic process // inferred from electronic annotation///0008652 // cellular amino ac
NM_001291058///NM_009853///NR		4.64E-06	0.00428983	1.04	**1.99**	**1.87**	***Cd68***	CD68 antigen	0071310 // cellular response to organic substance // inferred from direct assay
NM_001302129///NM_001302130//		0.005232663	0.0016117	-1.05	**1.29**	**1.85**	***Cenpa***	centromere protein A	0000132 // establishment of mitotic spindle orientation // not recorded///0051382 // kinetochore assembly // not recorded///0071459 // protein localization to chromosome, centrc
NM_011496///XM_006532725		3.10E-04	2.95E-05	1.02	**1.41**	**1.85**	***Aurkb***	aurora kinase B	0000122 // negative regulation of transcription from RNA polymerase II promoter // not recorded///0002903 // negative regulation of B cell apoptotic process // not recorded///000€
NM_001190717///NM_013726///XM_011240670			4.57E-04	1.04	**1.30**	**1.85**	***Dbf4***	DBF4 homolog (S. cerevisiae)	0006260 // DNA replication // inferred from electronic annotation///0006468 // protein phosphorylation // ---///0007049 // cell cycle // inferred from electronic annotation///00070
NM_033601///XM_00	3.02E-04	0.005906933	0.00800366	**1.49**	**1.67**	**1.85**	***Bcl3***	B cell leukemia/lymphoma 3	0000060 // protein import into nucleus, translocation // not recorded///0002268 // follicular dendritic cell differentiation // inferred from mutant phenotype///0002315 // marginal z
NM_001111078///NM_001111079//		1.66E-05	0.00208285	1.17	**1.78**	**1.84**	***Uhrf1***	ubiquitin-like, containing PHD and RING finger domains, 1	0000122 // negative regulation of transcription from RNA polymerase II promoter // inferred from direct assay///0000122 // negative regulation of transcription from RNA polymeras
NM_008021///NM_027697		7.54E-05	1.74E-05	1.15	**1.52**	**1.84**	***4933413G19Rik///Foxm1***	RIKEN cDNA 4933413G19 gene///forkhead box M1	0000086 // G2/M transition of mitotic cell cycle // not recorded///0000122 // negative regulation of transcription from RNA polymerase II promoter // not recorded///0000302 // res
NM_001313695///NM	0.00501023	0.001502541	8.53E-06	**1.20**	**1.53**	**1.83**	***Mcm6***	minichromosome maintenance deficient 6 (MIS5 homolog, S. pom	0006260 // DNA replication // inferred from electronic annotation///0006268 // DNA unwinding involved in DNA replication // inferred from physical interaction///0006270 // DNA re
NM_001253808///NM_001253809//		0.001827413	1.05E-04	-1.04	**1.29**	**1.83**	***Racgap1***	Rac GTPase-activating protein 1	0000281 // mitotic cytokinesis // not recorded///0000915 // actomyosin contractile ring assembly // not recorded///0006810 // transport // inferred from electronic annotation///00
NM_010655		0.003095412	8.97E-04	1.16	**1.65**	**1.82**	***Kpna2***	karyopherin (importin) alpha 2	0006606 // protein import into nucleus // inferred from direct assay///0006607 // NLS-bearing protein import into nucleus // not recorded///0006810 // transport // inferred from ele
NM_001164724///NM	1.77E-04	0.002093131	8.89E-04	**1.36**	**1.67**	**1.82**	***Il33***	interleukin 33	0002686 // negative regulation of leukocyte migration // inferred from genetic interaction///0002826 // negative regulation of T-helper 1 type immune response // inferred from gene
NM_001164787///NM_001309382//		0.002400771	1.05E-04	1.01	**1.27**	**1.81**	***Sprr2a1///Sprr2a2///Sprr2a3***	small proline-rich protein 2A1///small proline-rich protein 2A2///sı	0008360 // regulation of cell shape // traceable author statement///0008544 // epidermis development // non-traceable author statement///0018149 // peptide cross-linking // not r
NM_011315	1.19E-06	1.15E-04	5.68E-05	**1.81**	**2.33**	**1.81**	***Saa3***	serum amyloid A 3	0006953 // acute-phase response // inferred from electronic annotation///0007252 // I-kappaB phosphorylation // inferred from direct assay///0035634 // response to stilbenoid // ir
NM_001166409///NM_001166410//		2.02E-04		1.58	**2.66**	1.80	***Gm15453///Rbm3***	predicted gene 15453///RNA binding motif protein 3	0006412 // translation // inferred from direct assay///0006417 // regulation of translation // inferred from direct assay///0009409 // response to cold // inferred from direct assay///
NM_001166409///NM_001166410//		2.02E-04		1.58	**2.66**	1.80	***Gm15453///Rbm3***	predicted gene 15453///RNA binding motif protein 3	0006412 // translation // inferred from direct assay///0006417 // regulation of translation // inferred from direct assay///0009409 // response to cold // inferred from direct assay///
NM_008416	0.00103719	0.004419017	0.00300824	**1.29**	**1.57**	**1.79**	***Junb***	jun B proto-oncogene	0000122 // negative regulation of transcription from RNA polymerase II promoter // not recorded///0001570 // vasculogenesis // inferred from mutant phenotype///0001649 // oste
NM_010288///XM_006512541///XM		0.003596864	0.00470297	1.37	**1.71**	**1.78**	***Gja1***	gap junction protein, alpha 1	0001649 // osteoblast differentiation // inferred from mutant phenotype///0001701 // in utero embryonic development // inferred from mutant phenotype///0001764 // neuron mig
NM_001285997///NM_001285998//		0.001642908	6.70E-04	-1.01	**1.35**	**1.78**	***Prc1***	protein regulator of cytokinesis 1	0000226 // microtubule cytoskeleton organization // inferred from electronic annotation///0000910 // cytokinesis // not recorded///0007049 // cell cycle // inferred from electronic
NM_009272	0.00458492	0.003818341	0.00350854	**1.26**	**1.53**	**1.77**	***Srm***	spermidine synthase	0006595 // polyamine metabolic process // inferred from electronic annotation///0006596 // polyamine biosynthetic process // inferred from electronic annotation///0008295 // spe
NM_010436		0.004716281	0.00983426	1.05	**1.35**	**1.77**	***H2afx***	H2A histone family, member X	0000077 // DNA damage checkpoint // inferred from mutant phenotype///0000724 // double-strand break repair via homologous recombination // inferred from mutant phenotype/
NM_023627	0.00989229		0.00340502	**1.17**	1.23	**1.76**	***Isyna1***	myo-inositol 1-phosphate synthase A1	0006021 // inositol biosynthetic process // inferred from electronic annotation///0006629 // lipid metabolic process // inferred from electronic annotation///0008654 // phospholipid
NM_011498		4.98E-05	9.85E-05	1.23	**1.80**	**1.75**	***Bhlhe40***	basic helix-loop-helix family, member e40	0000122 // negative regulation of transcription from RNA polymerase II promoter // not recorded///0006351 // transcription, DNA-templated // inferred from electronic annotation//

RefSeq Transcript ID	p (24h n=460)	p (48h n=657)	p (72h n=805)	FC 24h	FC 48h	FC 72h	Gene Symbol	Gene Title	Gene Ontology Biological Process
NM_001313963///NM	7.58E-04	0.001150255	2.22E-04	1.15	1.98	1.75	*Krt19*	keratin 19	0007219 // Notch signaling pathway // inferred from direct assay///0043627 // response to estrogen // inferred from electronic annotation///0045214 // sarcomere organization // n
NM_013602///XM_00	1.12E-05	2.53E-04	1.26E-04	2.15	2.08	1.75	*Mt1*	metallothionein 1	0000032 // cell wall mannoprotein biosynthetic process // ---///0006486 // protein glycosylation // inferred from electronic annotation///0006493 // protein O-linked glycosylation //
NM_007802///XM_006500974		0.001038144	1.41E-04	1.08	1.60	1.74	*Ctsk*	cathepsin K	0001957 // intramembranous ossification // inferred from electronic annotation///0006508 // proteolysis // inferred from electronic annotation///0030574 // collagen catabolic proc
NM_001113179///NM_009772///XM		0.004492327	7.74E-04	-1.03	1.49	1.74	*Bub1*	budding uninhibited by benzimidazoles 1 homolog (S. cerevisiae)	0006468 // protein phosphorylation // inferred from electronic annotation///0006915 // apoptotic process // inferred from electronic annotation///0007049 // cell cycle // inferred fr
NM_009890	8.96E-05	2.74E-05	3.54E-04	1.68	2.17	1.74	*Ch25h*	cholesterol 25-hydroxylase	0006629 // lipid metabolic process // inferred from electronic annotation///0006633 // fatty acid biosynthetic process // inferred from electronic annotation///0006694 // steroid bio
NM_008889	0.00310169	0.004915863	0.0030352	1.18	1.42	1.73	*Ppp1r14b*	protein phosphatase 1, regulatory (inhibitor) subunit 14B	0042325 // regulation of phosphorylation // inferred from electronic annotation///0043086 // negative regulation of catalytic activity // not recorded///0050790 // regulation of cata
NM_009364///XM_006505043///XM		0.006131017	5.76E-05	1.30	1.50	1.72	*Tfpi2*	tissue factor pathway inhibitor 2	0007596 // blood coagulation // inferred from electronic annotation///0007599 // hemostasis // inferred from electronic annotation///0010466 // negative regulation of peptidase ac
NM_001033166///NM_001037279			0.00404433	1.02	1.41	1.72	*2700094K13Rik*	RIKEN cDNA 2700094K13 gene	0045454 // cell redox homeostasis // inferred from electronic annotation
NM_001291185///NM_011497///XM		7.60E-04	1.40E-04	1.04	1.34	1.72	*Aurka*	aurora kinase A	0000212 // meiotic spindle organization // inferred from mutant phenotype///0000226 // microtubule cytoskeleton organization // inferred from genetic interaction///0000278 // mi
NM_001159299///NM	7.13E-05		0.00267216	1.39	1.66	1.72	*Itih4*	inter alpha-trypsin inhibitor, heavy chain 4	0006953 // acute-phase response // inferred from electronic annotation///0010466 // negative regulation of peptidase activity // inferred from electronic annotation///0010951 // ne
NM_020010	7.94E-05	2.01E-06	4.44E-04	1.70	2.03	1.72	*Cyp51*	cytochrome P450, family 51	0006629 // lipid metabolic process // inferred from electronic annotation///0006694 // steroid biosynthetic process // not recorded///0006695 // cholesterol biosynthetic process //
NM_145360///XM_00	4.63E-04	1.26E-04	4.04E-04	1.59	1.84	1.71	*Gm38481///Idi1*	predicted gene, 38481///isopentenyl-diphosphate delta isomerase	0006629 // lipid metabolic process // inferred from electronic annotation///0006694 // steroid biosynthetic process // inferred from electronic annotation///0006695 // cholesterol b
NM_013492///XM_006518503///XM		3.28E-04	1.59E-04	1.05	1.38	1.71	*Clu*	clusterin	0000902 // cell morphogenesis // not recorded///0001774 // microglial cell activation // not recorded///0008219 // cell death // inferred from electronic annotation///0008284 // po
NM_008605///XM_011242357///XM_011242358			0.00785685	1.11	1.47	1.70	*Mmp12*	matrix metallopeptidase 12	0006508 // proteolysis // inferred from electronic annotation///0014070 // response to organic cyclic compound // inferred from electronic annotation///0035313 // wound healing,
NM_010766		3.78E-05	7.05E-05	1.30	2.25	1.70	*Marco*	macrophage receptor with collagenous structure	0002376 // immune system process // inferred from electronic annotation///0006897 // endocytosis // inferred from mutant phenotype///0006898 // receptor-mediated endocytosi
NM_022430		0.007245855	2.28E-04	1.02	1.50	1.70	*Ms4a8a*	membrane-spanning 4-domains, subfamily A, member 8A	
NM_011239		0.00127528	0.00199509	1.11	1.40	1.70	*Ranbp1*	RAN binding protein 1	0007051 // spindle organization // inferred from direct assay///0010976 // positive regulation of neuron projection development // not recorded///0035690 // cellular response to dr
NM_145480		3.58E-04	0.00111975	1.07	1.45	1.68	*Rfc4*	replication factor C (activator 1) 4	0006260 // DNA replication // inferred from electronic annotation///0006261 // DNA-dependent DNA replication // not recorded///0006281 // DNA repair // not recorded
NM_011495///NM_173169///NR_04		0.003996193	3.33E-04	1.08	1.40	1.68	*Plk4*	polo-like kinase 4	0006468 // protein phosphorylation // inferred from electronic annotation///0007099 // centriole replication // inferred from mutant phenotype///0007099 // centriole replication //
NM_001313914///NM	6.43E-05	5.63E-04		2.55	2.41	1.67	*Thbs1*	thrombospondin 1	0000187 // activation of MAPK activity // not recorded///0001937 // negative regulation of endothelial cell proliferation // inferred from mutant phenotype///0001937 // negative re
NM_011905///XM_006501460		2.95E-04	1.71E-05	1.06	1.50	1.67	*Tlr2*	toll-like receptor 2	0001774 // microglial cell activation // not recorded///0002224 // toll-like receptor signaling pathway // not recorded///0002237 // response to molecule of bacterial origin // inferre
NM_011568///NM_019484///XR_38		0.001122384		1.05	1.35	1.67	*Alyref///Alyref2*	Aly/REF export factor///Aly/REF export factor 2	0006397 // mRNA processing // inferred from electronic annotation///0006406 // mRNA export from nucleus // inferred from electronic annotation///0006810 // transport // inferre
NM_019499///XM_006506406		0.005283785	0.00202498	1.02	1.48	1.67	*Mad2l1*	MAD2 mitotic arrest deficient-like 1	0000070 // mitotic sister chromatid segregation // inferred from mutant phenotype///0007049 // cell cycle // inferred from electronic annotation///0007067 // mitotic nuclear divisic
NM_026014		7.49E-04	0.00250318	1.07	1.40	1.66	*Cdt1*	chromatin licensing and DNA replication factor 1	0000076 // DNA replication checkpoint // inferred from direct assay///0000076 // DNA replication checkpoint // not recorded///0006260 // DNA replication // inferred from electroni
NM_026835			0.0010361	1.32	1.53	1.65	*Ms4a6d*	membrane-spanning 4-domains, subfamily A, member 6D	
NM_010295///XM_00	3.81E-04		1.02E-04	1.54	1.24	1.65	*Gclc*	glutamate-cysteine ligase, catalytic subunit	0006534 // cysteine metabolic process // not recorded///0006536 // glutamate metabolic process // not recorded///0006749 // glutathione metabolic process // inferred from muta
NM_133762///XM_006516349///XM_006516350//			0.00204582	1.02	1.50	1.64	*Ncapg2*	non-SMC condensin II complex, subunit G2	0001833 // inner cell mass cell proliferation // inferred from mutant phenotype///0007049 // cell cycle // inferred from electronic annotation///0007067 // mitotic nuclear division //
NM_011227///XM_01	3.56E-04	1.94E-04	1.85E-04	1.43	1.56	1.63	*Rab20*	RAB20, member RAS oncogene family	0006810 // transport // inferred from electronic annotation///0006886 // intracellular protein transport // not recorded///0007264 // small GTPase mediated signal transduction // in
NM_010172		2.03E-04	1.05E-04	1.06	1.46	1.63	*F7*	coagulation factor VII	0002690 // positive regulation of leukocyte chemotaxis // not recorded///0006508 // proteolysis // inferred from electronic annotation///0007596 // blood coagulation // inferred fro
NM_001033484///XM_006501643		1.77E-04	3.76E-05	1.07	1.40	1.62	*Iqgap3*	IQ motif containing GTPase activating protein 3	0000082 // G1/S transition of mitotic cell cycle // inferred from mutant phenotype///0000187 // activation of MAPK activity // inferred from mutant phenotype///0001934 // positive
NM_020047	2.41E-04	5.60E-05	0.0038563	1.31	1.62	1.62	*Tacstd2*	tumor-associated calcium signal transducer 2	0010633 // negative regulation of epithelial cell migration // inferred from direct assay///0050678 // regulation of epithelial cell proliferation // inferred from expression pattern///005
NM_001297747///NM	0.00298634	7.39E-04	0.00601065	1.22	1.50	1.61	*Btg3///Gm7334*	B cell translocation gene 3///B-cell translocation gene 3 pseudoger	0045930 // negative regulation of mitotic cell cycle // not recorded
NM_001316995///NM_016750///XM		1.08E-04	1.93E-04	1.09	1.59	1.61	*Gm8203///H2afz*	predicted pseudogene 8203///H2A histone family, member Z	0006325 // chromatin organization // not recorded///0006342 // chromatin silencing // not recorded///0007275 // multicellular organismal development // inferred from electronic a
NM_010790///XM_006537640			3.86E-04	-1.05	1.23	1.61	*Melk*	maternal embryonic leucine zipper kinase	0006468 // protein phosphorylation // inferred from electronic annotation///0006915 // apoptotic process // not recorded///0007049 // cell cycle // inferred from electronic annotat
NM_023284///XM_006496958		0.003325016		1.02	1.35	1.61	*Nuf2*	NUF2, NDC80 kinetochore complex component, homolog (S. cerev	0007049 // cell cycle // inferred from electronic annotation///0007067 // mitotic nuclear division // inferred from electronic annotation///0008608 // attachment of spindle microtub
NM_026438	0.0013035	6.85E-04	0.00236947	1.25	1.43	1.61	*Ppa1*	pyrophosphatase (inorganic) 1	0006796 // phosphate-containing compound metabolic process // inferred by curator
NM_001077529///NM_008705		0.001581035	4.51E-04	1.06	1.38	1.60	*Nme2*	NME/NM23 nucleoside diphosphate kinase 2	0002762 // negative regulation of myeloid leukocyte differentiation // not recorded///0006163 // purine nucleotide metabolic process // not recorded///0006165 // nucleoside dipho
NM_010798		0.009453878	0.0029498	1.08	1.40	1.59	*Mif*	macrophage migration inhibitory factor	0001516 // prostaglandin biosynthetic process // not recorded///0001934 // positive regulation of protein phosphorylation // inferred from direct assay///0002035 // brain renin-ang
NM_026410		4.05E-04	6.53E-05	1.03	1.43	1.59	*Cdca5*	cell division cycle associated 5	0000082 // G1/S transition of mitotic cell cycle // not recorded///0006302 // double-strand break repair // not recorded///0007049 // cell cycle // inferred from electronic annotation
NM_001145953///NM	2.00E-04	9.01E-05	7.30E-05	1.30	1.73	1.59	*Lgals3*	lectin, galactose binding, soluble 3	0001501 // skeletal system development // inferred from genetic interaction///0002376 // immune system process // inferred from electronic annotation///0002548 // monocyte ch
NM_025443		0.006398648	0.00593472	1.30	1.42	1.59	*Pno1*	partner of NOB1 homolog (S. cerevisiae)	
NM_001199123///NM_001199124//		7.23E-05	4.72E-05	1.12	1.42	1.59	*Spc25*	SPC25, NDC80 kinetochore complex component, homolog (S. cere	0007049 // cell cycle // inferred from electronic annotation///0007052 // mitotic spindle organization // not recorded///0007059 // chromosome segregation // not recorded///0007
NM_008102	0.00106521	0.00156661		1.39	1.45	1.58	*Gch1*	GTP cyclohydrolase 1	0006461 // protein complex assembly // not recorded///0006729 // tetrahydrobiopterin biosynthetic process // not recorded///0006729 // tetrahydrobiopterin biosynthetic process ,
NM_016723///NM_033607///XM_0C		0.007923941	0.00390497	1.21	1.41	1.58	*Uchl3///Uchl4*	ubiquitin carboxyl-terminal esterase L3 (ubiquitin thiolesterase)///	0006508 // proteolysis // inferred from electronic annotation///0006511 // ubiquitin-dependent protein catabolic process // inferred from electronic annotation///0007628 // adult w
NM_011072		0.007228755		1.02	1.30	1.58	*Pfn1*	profilin 1	0001843 // neural tube closure // inferred from genetic interaction///0006357 // regulation of transcription from RNA polymerase II promoter // inferred from genetic interaction///0
NM_011311///XM_006501177///XM_006501178			0.00139097	1.17	1.31	1.58	*S100a4*	S100 calcium binding protein A4	0043123 // positive regulation of I-kappaB kinase/NF-kappaB signaling // not recorded
NM_001308227///NM	3.83E-04	3.58E-04	0.00585075	1.78	1.64	1.58	*Dnajb1*	DnaJ (Hsp40) homolog, subfamily B, member 1	0006457 // protein folding // inferred from electronic annotation///0032781 // positive regulation of ATPase activity // not recorded///0051085 // chaperone mediated protein foldin
NM_146171///XM_006506531///XM		2.20E-04	0.00136684	-1.04	1.47	1.57	*Ncapd2*	non-SMC condensin I complex, subunit D2	0007049 // cell cycle // inferred from electronic annotation///0007067 // mitotic nuclear division // inferred from electronic annotation///0007076 // mitotic chromosome condensa
NM_009841	4.27E-06	1.80E-05	4.68E-04	1.61	1.86	1.57	*Cd14*	CD14 antigen	0002237 // response to molecule of bacterial origin // inferred from direct assay///0002376 // immune system process // inferred from electronic annotation///0006898 // receptor-r
NM_028128		0.00132008	9.28E-05	1.10	1.27	1.57	*Rfc5*	replication factor C (activator 1) 5	0006260 // DNA replication // inferred from electronic annotation///0006261 // DNA-dependent DNA replication // not recorded///0006281 // DNA repair // not recorded
NM_011045	0.00176512	2.49E-04		1.22	1.57	1.57	*Pcna*	proliferating cell nuclear antigen	0000122 // negative regulation of transcription from RNA polymerase II promoter // inferred from direct assay///0006260 // DNA replication // inferred from electronic annotation///
NM_133897///XM_00	0.00132762	9.05E-05	1.25E-04	1.31	1.74	1.56	*Lrrc8c*	leucine rich repeat containing 8 family, member C	0006810 // transport // inferred from electronic annotation///0006811 // ion transport // inferred from electronic annotation///0045444 // fat cell differentiation // inferred from dir
NM_026816		0.003901336	0.00330454	1.27	1.41	1.56	*Gtf2f2*	general transcription factor IIF, polypeptide 2	0006351 // transcription, DNA-templated // inferred from electronic annotation///0006355 // regulation of transcription, DNA-templated // inferred from electronic annotation///00C
NM_197982///XM_00	0.00690338	4.72E-05	1.95E-04	1.21	1.54	1.56	*Ddx39*	DEAD (Asp-Glu-Ala-Asp) box polypeptide 39	0000398 // mRNA splicing, via spliceosome // not recorded///0006397 // mRNA processing // inferred from electronic annotation///0006406 // mRNA export from nucleus // not rec
NM_011955///XR_384565		6.56E-04	0.0011203	1.14	1.38	1.56	*Nubp1*	nucleotide binding protein 1	0006879 // cellular iron ion homeostasis // not recorded///0010826 // negative regulation of centrosome duplication // inferred from mutant phenotype///0016049 // cell growth //
NM_009252///XM_01	1.85E-04	1.10E-04	2.53E-04	1.41	2.01	1.55	*Serpina3n*	serine (or cysteine) peptidase inhibitor, clade A, member 3N	0006953 // acute-phase response // inferred from electronic annotation///0010466 // negative regulation of peptidase activity // inferred from electronic annotation///0010951 // ne
NM_009139			0.00320978	-1.05	1.48	1.55	*Ccl6*	chemokine (C-C motif) ligand 6	0002548 // monocyte chemotaxis // ---///0006935 // chemotaxis // inferred from electronic annotation///0006955 // immune response // inferred from electronic annotation///000
NM_001081029		7.29E-05		1.23	1.71	1.54	*Tmem243*	transmembrane protein 243, mitochondrial	
NM_026631	7.86E-04	0.00137641	4.31E-04	1.25	1.70	1.54	*Nhp2*	NHP2 ribonucleoprotein	0006364 // rRNA processing // inferred from electronic annotation///0031118 // rRNA pseudouridine synthesis // inferred from sequence or structural similarity///0042254 // ribosor
NM_001159646///NM_023595		0.004399282		1.09	1.25	1.54	*Dut*	deoxyuridine triphosphatase	0006226 // dUMP biosynthetic process // not recorded///0043497 // regulation of protein heterodimerization activity // not recorded///0046080 // dUTP metabolic process // inferre
NM_007534///NM_007536///NM_0(		5.36E-04	0.00135674	1.14	1.69	1.54	*Bcl2a1a///Bcl2a1b///Bcl2a1d*	B cell leukemia/lymphoma 2 related protein A1a///B cell leukemia,	0001782 // B cell homeostasis // inferred from direct assay///0002903 // negative regulation of B cell apoptotic process // inferred from direct assay///0006915 // apoptotic process /
NM_001313711///NM_016764///XM		0.00168253	3.74E-04	1.00	1.30	1.54	*Prdx4*	peroxiredoxin 4	0007283 // spermatogenesis // inferred from mutant phenotype///0008584 // male gonad development // inferred from mutant phenotype///0019471 // 4-hydroxyproline metaboli
NM_001267695///NM_021281///XM_006500975			0.00346088	-1.08	1.17	1.54	*Ctss*	cathepsin S	0002250 // adaptive immune response // inferred from electronic annotation///0006508 // proteolysis // inferred from direct assay///0006508 // proteolysis // not recorded///0006
NM_001109748///NM_016871			0.00271894	1.17	1.27	1.54	*Tomm40*	translocase of outer mitochondrial membrane 40 homolog (yeast)	0006626 // protein targeting to mitochondrion // not recorded///0006810 // transport // inferred from electronic annotation///0006811 // ion transport // inferred from electronic a
NM_007988		0.005565248		1.08	1.65	1.53	*Fasn*	fatty acid synthase	0001649 // osteoblast differentiation // not recorded///0006084 // acetyl-CoA metabolic process // not recorded///0006629 // lipid metabolic process // inferred from electronic ann
NM_001316995///NM_016750///XM		4.05E-04	6.46E-04	1.05	1.55	1.53	*Gm8203///H2afz*	predicted pseudogene 8203///H2A histone family, member Z	0006325 // chromatin organization // not recorded///0006342 // chromatin silencing // not recorded///0007275 // multicellular organismal development // inferred from electronic a
NM_026623		0.005949182		1.08	1.39	1.53	*Nudt21*	nudix (nucleoside diphosphate linked moiety X)-type motif 21	0006378 // mRNA polyadenylation // not recorded///0006379 // mRNA cleavage // not recorded///0006397 // mRNA processing // not recorded///0051262 // protein tetramerizatic
NM_011568///XR_388401///NM_01		0.002020814	0.00631495	1.12	1.30	1.53	*Alyref///Alyref2*	Aly/REF export factor///Aly/REF export factor 2	0006397 // mRNA processing // inferred from electronic annotation///0006810 // transport // inferred from electronic annotation///0008380 // RNA splicing // inferred from electror
NM_001252588///NM_053082///XM		0.002247224	2.55E-05	1.19	1.50	1.53	*Tspan4*	tetraspanin 4	0006461 // protein complex assembly // not recorded///0007166 // cell surface receptor signaling pathway // not recorded
NM_010730			0.00386172	1.12	1.38	1.52	*Anxa1*	annexin A1	0000733 // DNA strand renaturation // not recorded///0001780 // neutrophil homeostasis // inferred from mutant phenotype///0001780 // neutrophil homeostasis // not recorded/
NM_008969///XM_00	0.00897244	0.001487609	0.00123294	1.21	1.46	1.51	*Ptgs1*	prostaglandin-endoperoxide synthase 1	0001516 // prostaglandin biosynthetic process // inferred from mutant phenotype///0001516 // prostaglandin biosynthetic process // not recorded///0006629 // lipid metabolic proc
NM_001278115///NM_026024			0.00352537	1.00	1.26	1.51	*Ube2t*	ubiquitin-conjugating enzyme E2T (putative)	0000209 // protein polyubiquitination // not recorded///0006281 // DNA repair // not recorded///0006513 // protein monoubiquitination // not recorded///0006974 // cellular respo
NR_002847			0.00757484	1.03	-1.05	1.51	*Malat1*	metastasis associated lung adenocarcinoma transcript 1 (non-codi	0000381 // regulation of alternative mRNA splicing, via spliceosome // inferred from mutant phenotype///0035063 // nuclear speck organization // inferred from mutant phenotype/
NM_011638///XM_00	0.00597165			1.44	1.53	1.51	*Tfrc*	transferrin receptor	0001666 // response to hypoxia // inferred from electronic annotation///0006879 // cellular iron ion homeostasis // inferred from mutant phenotype///0006897 // endocytosis // inf
NM_001164086///NM_001164087//		0.009353247	0.00358742	1.15	1.34	1.51	*Homer2*	homer homolog 2 (Drosophila)	0007216 // G-protein coupled glutamate receptor signaling pathway // traceable author statement///0008277 // regulation of G-protein coupled receptor protein signaling pathway /
NM_009404///XM_00	2.14E-04	2.90E-05	8.34E-04	1.32	2.07	1.51	*Tnfsf9*	tumor necrosis factor (ligand) superfamily, member 9	0006955 // immune response // inferred from electronic annotation///0007267 // cell-cell signaling // not recorded///0032729 // positive regulation of interferon-gamma production
NM_027204	0.00224774	0.001273689		1.19	1.40	1.50	*Mrpl12*	mitochondrial ribosomal protein L12	0006390 // transcription from mitochondrial promoter // not recorded///0006412 // translation // inferred from sequence or structural similarity///0045893 // positive regulation of
NM_001271729///NM_009387///XM		1.27E-05		1.02	1.71	1.50	*Tk1*	thymidine kinase 1	0001889 // liver development // inferred from electronic annotation///0009157 // deoxyribonucleoside monophosphate biosynthetic process // inferred from direct assay///0009157
NM_001163763///NM_001163764//		0.001597252	0.00203503	1.06	1.40	1.50	*Tcf19*	transcription factor 19	
NM_008503///XM_006523728///XR_861768///XR_			0.006864	1.06	1.26	1.50	*LOC105242669///LOC105243270*	40S ribosomal protein S2 pseudogene///40S ribosomal protein S2-	0000028 // ribosomal small subunit assembly // not recorded///0001731 // formation of translation preinitiation complex // not recorded///0006412 // translation // inferred from el
NM_009912			0.00554155	-1.02	1.34	1.49	*Ccr1*	chemokine (C-C motif) receptor 1	0006816 // calcium ion transport // not recorded///0006874 // cellular calcium ion homeostasis // not recorded///0006887 // exocytosis // not recorded///0006935 // chemotaxis //
NM_009903		0.00829391	0.00808543	1.09	1.35	1.49	*Cldn4*	claudin 4	0007565 // female pregnancy // inferred from electronic annotation///0007623 // circadian rhythm // inferred from electronic annotation///0016338 // calcium-independent cell-cell
NM_026473		7.66E-04	6.83E-04	1.19	1.48	1.49	*Tubb6*	tubulin, beta 6 class V	0007017 // microtubule-based process // inferred from electronic annotation///0008152 // metabolic process // inferred from electronic annotation///0051258 // protein polymeriza
NM_001301728///NM	6.61E-04	0.00952863		1.20	1.51	1.49	*Fdx1*	ferredoxin 1	0006629 // lipid metabolic process // inferred from electronic annotation///0006694 // steroid biosynthetic process // inferred from electronic annotation///0006810 // transport // i
NM_145409///XM_006524017			5.28E-04	1.01	1.25	1.49	*Chtf18*	CTF18, chromosome transmission fidelity factor 18	0006260 // DNA replication // inferred from electronic annotation///0007049 // cell cycle // inferred from electronic annotation///0007131 // reciprocal meiotic recombination // inf
NM_009448	0.00730558	8.42E-05	7.46E-05	1.25	1.47	1.48	*Tuba1c*	tubulin, alpha 1C	0007017 // microtubule-based process // inferred from electronic annotation///0008152 // metabolic process // inferred from electronic annotation///0051258 // protein polymeriza

RefSeq Transcript ID	p (24h n=460)	p (48h n=657)	p (72h n=805)	FC 24h	FC 48h	FC 72h	Gene Symbol	Gene Title	Gene Ontology Biological Process
NM_001172117///NM	0.00215644	0.001511017	2.38E-04	1.27	1.34	1.48	Hck	hemopoietic cell kinase	0002376 // immune system process // inferred from electronic annotation///0006468 // protein phosphorylation // inferred from electronic annotation///0006887 // exocytosis // in
NM_009226			0.00823008	1.27	1.18	1.47	Snrpd1	small nuclear ribonucleoprotein D1	0000245 // spliceosomal complex assembly // not recorded///0000387 // spliceosomal snRNP assembly // not recorded///0002230 // positive regulation of defense response to virus
NM_007573	0.0013884	0.009837708		1.31	1.28	1.47	C1qbp	complement component 1, q subcomponent binding protein	0000122 // negative regulation of transcription from RNA polymerase II promoter // not recorded///0002250 // adaptive immune response // inferred from electronic annotation///0
NM_001161737///NM_013929			0.00979084	1.12	1.58	1.47	Siva1	SIVA1, apoptosis-inducing factor	0006915 // apoptotic process // inferred from electronic annotation///0006924 // activation-induced cell death of T cells // not recorded///0032088 // negative regulation of NF-kapp
NM_001290660///NM_025422			9.34E-04	1.09	1.42	1.47	Cd302	CD302 antigen	0006909 // phagocytosis // not recorded
NM_008503///XM_006523728///XR_		0.007315373		1.06	1.27	1.46	LOC105242669///LOC105243270	40S ribosomal protein S2 pseudogene///40S ribosomal protein S2-	0000028 // ribosomal small subunit assembly // not recorded///0001731 // formation of translation preinitiation complex // not recorded///0006412 // translation // inferred from el
NM_153566	7.57E-04	0.003407072		1.26	1.24	1.46	Yrdc	yrdC domain containing (E.coli)	0051051 // negative regulation of transport // not recorded
NM_001025779///NM_011799///XM		0.001022888		1.07	1.43	1.46	Cdc6	cell division cycle 6	0006260 // DNA replication // inferred from electronic annotation///0006270 // DNA replication initiation // inferred from electronic annotation///0007049 // cell cycle // inferred fro
NM_024245///XM_006511452///XM		0.005921938	0.00101346	1.06	1.27	1.46	Kif23	kinesin family member 23	0000281 // mitotic cytokinesis // not recorded///0000910 // cytokinesis // not recorded///0007018 // microtubule-based movement // not recorded///0007049 // cell cycle // inferre
NM_031198///XM_006505035///XM		0.003699567	0.00996861	1.20	1.48	1.46	Tfec	transcription factor EC	0006351 // transcription, DNA-templated // inferred from electronic annotation///0006355 // regulation of transcription, DNA-templated // not recorded///0006366 // transcription t
NM_019432///XR_878316		0.001244167	5.82E-04	1.09	1.71	1.46	Tmem37	transmembrane protein 37	0006810 // transport // inferred from electronic annotation///0006811 // ion transport // inferred from electronic annotation///0006816 // calcium ion transport // inferred from ele
NM_033073			0.00112779	1.00	1.39	1.45	Krt7	keratin 7	
NM_001110265///NM_001284272///NM_009445//			0.00202557	1.21	1.28	1.45	Ttk	Ttk protein kinase	0006468 // protein phosphorylation // inferred from electronic annotation///0007093 // mitotic cell cycle checkpoint // inferred from electronic annotation///0016310 // phosphoryl
NM_008495		0.007730463		1.12	1.53	1.45	Lgals1	lectin, galactose binding, soluble 1	0002317 // plasma cell differentiation // inferred from direct assay///0006915 // apoptotic process // inferred from electronic annotation///0007157 // heterophilic cell-cell adhesion
NM_009391///XM_006504280///XM		0.002499048	2.11E-04	1.14	1.31	1.45	LOC102641332///Ran	GTP-binding nuclear protein Ran///RAN, member RAS oncogene fa	0000054 // ribosomal subunit export from nucleus // not recorded///0000055 // ribosomal large subunit export from nucleus // not recorded///0000056 // ribosomal small subunit ex
NM_008564		6.06E-04	0.00233453	1.10	1.32	1.45	Mcm2	minichromosome maintenance deficient 2 mitotin (S. cerevisiae)	0006260 // DNA replication // inferred from electronic annotation///0006268 // DNA unwinding involved in DNA replication // inferred from direct assay///0006270 // DNA replicatio
NM_001164733///NM_001164734//		2.81E-04		1.13	1.43	1.45	Mpp6	membrane protein, palmitoylated 6 (MAGUK p55 subfamily memb	0046037 // GMP metabolic process // inferred from sequence or structural similarity///0046710 // GDP metabolic process // inferred from sequence or structural similarity///004693
NM_023587///XM_006522570///XM		0.001676333		1.02	1.35	1.45	Hacd2	3-hydroxyacyl-CoA dehydratase 2	0006629 // lipid metabolic process // inferred from electronic annotation///0006631 // fatty acid metabolic process // inferred from electronic annotation///0006633 // fatty acid bio
NM_026545		0.001469113	0.00146929	-1.04	1.34	1.45	Psmd8	proteasome (prosome, macropain) 26S subunit, non-ATPase, 8	0006508 // proteolysis // inferred from electronic annotation///0031647 // regulation of protein stability // not recorded///0043161 // proteasome-mediated ubiquitin-dependent pr
NM_007413		0.001711548	0.00241723	1.16	1.33	1.45	Adora2b	adenosine A2b receptor	0001973 // adenosine receptor signaling pathway // inferred from electronic annotation///0002882 // positive regulation of chronic inflammatory response to non-antigenic stimulus
NM_134156///XM_006515384///XM		0.007809449		1.05	1.34	1.45	Actn1	actinin, alpha 1	0007015 // actin filament organization // not recorded///0030220 // platelet formation // not recorded///0030865 // cortical cytoskeleton organization // traceable author statemen
NM_010516		0.002322059	0.00254977	-1.01	1.48	1.44	Cyr61	cysteine rich protein 61	0001558 // regulation of cell growth // inferred from electronic annotation///0001649 // osteoblast differentiation // inferred from genetic interaction///0001934 // positive regulatio
NM_008568///XM_006504555///XM		1.25E-04	0.00292747	1.01	1.46	1.44	Mcm7	minichromosome maintenance deficient 7 (S. cerevisiae)	0006260 // DNA replication // inferred from electronic annotation///0006268 // DNA unwinding involved in DNA replication // inferred from physical interaction///0006270 // DNA re
NM_011950		0.002006226	1.25E-04	1.16	1.27	1.43	Mapk13	mitogen-activated protein kinase 13	0000165 // MAPK cascade // not recorded///0006351 // transcription, DNA-templated // inferred from electronic annotation///0006355 // regulation of transcription, DNA-templated
NM_016957		0.007600313		1.06	1.63	1.43	Hmgn2	high mobility group nucleosomal binding domain 2	0000122 // negative regulation of transcription from RNA polymerase II promoter // inferred from mutant phenotype///0006357 // regulation of transcription from RNA polymerase I
NM_001163476///NM_027014///XM		0.00143673	7.49E-04	1.08	1.27	1.43	Gins1	GINS complex subunit 1 (Psf1 homolog)	0001833 // inner cell mass cell proliferation // inferred from mutant phenotype///0006260 // DNA replication // inferred from mutant phenotype///1902983 // DNA strand elongatio
NM_027430		0.004797708		1.08	1.40	1.43	Mpc2	mitochondrial pyruvate carrier 2	0006090 // pyruvate metabolic process // inferred from genetic interaction///0006810 // transport // inferred from electronic annotation///0006850 // mitochondrial pyruvate trans
NM_001252563///NM_013592///XM		0.008045459		-1.07	-1.32	1.43	Matn4	matrilin 4	0048678 // response to axon injury // inferred from direct assay
NM_008563		9.13E-04	0.00684152	1.11	1.30	1.43	Mcm3	minichromosome maintenance deficient 3 (S. cerevisiae)	0006260 // DNA replication // inferred from electronic annotation///0006270 // DNA replication initiation // inferred from electronic annotation///0007049 // cell cycle // inferred fro
NM_133807		0.004138883	1.01E-04	1.12	1.24	1.43	Lrrc59	leucine rich repeat containing 59	
NM_001081363///XM_006497119			0.00111868	-1.04	1.20	1.43	Cenpf	centromere protein F	0000278 // mitotic cell cycle // not recorded///0002724 // regulation of T cell cytokine production // inferred from mutant phenotype///0002730 // regulation of dendritic cell cytoki
NM_001163489///NM_001163490//		9.64E-04		1.15	1.29	1.42	Sema4a	sema domain, immunoglobulin domain (Ig), transmembrane doma	0001525 // angiogenesis // inferred from electronic annotation///0001755 // neural crest cell migration // not recorded///0002250 // adaptive immune response // inferred from ele
NM_001287738///NM	4.16E-04			1.35	1.29	1.42	Cebpb	CCAAT/enhancer binding protein (C/EBP), beta	0001541 // ovarian follicle development // inferred from mutant phenotype///0001892 // embryonic placenta development // inferred from genetic interaction///0002432 // granulo
NM_001102404///NM_001102405//		5.36E-04	1.89E-04	1.03	1.49	1.42	Acp5	acid phosphatase 5, tartrate resistant	0006470 // protein dephosphorylation // inferred from electronic annotation///0016311 // dephosphorylation // inferred from direct assay///0032496 // response to lipopolysacchar
NM_001141975///NM_001141976//		0.005837853	0.00813172	1.06	1.23	1.42	Tpx2	TPX2, microtubule-associated protein homolog (Xenopus laevis)	0006915 // apoptotic process // inferred from electronic annotation///0007049 // cell cycle // inferred from electronic annotation///0007067 // mitotic nuclear division // inferred fro
NM_008892///XM_006527905///XR_878095///XR_			0.00486879	1.04	1.15	1.42	Pola1	polymerase (DNA directed), alpha 1	0000731 // DNA synthesis involved in DNA repair // inferred from sequence or structural similarity///0006260 // DNA replication // inferred from direct assay///0006260 // DNA replic
NM_007421		0.002353767		1.12	1.69	1.42	Adssl1	adenylosuccinate synthetase like 1	0006163 // purine nucleotide metabolic process // inferred from direct assay///0006164 // purine nucleotide biosynthetic process // inferred from electronic annotation///0006167 /,
NM_001127382///NM_001291226///NM_139065//			0.00730261	1.04	1.05	1.42	Rbm47	RNA binding motif protein 47	0002244 // hematopoietic progenitor cell differentiation // inferred from mutant phenotype///0016554 // cytidine to uridine editing // inferred from genetic interaction
NM_026405			0.0062981	1.09	1.32	1.41	Rab32	RAB32, member RAS oncogene family	0006886 // intracellular protein transport // not recorded///0007264 // small GTPase mediated signal transduction // inferred from electronic annotation///0008152 // metabolic pro
NM_001304758///NM	0.00561732	0.001122649	0.00622649	1.21	1.34	1.41	Coq7	demethyl-Q 7	0000122 // negative regulation of transcription from RNA polymerase II promoter // not recorded///0001306 // age-dependent response to oxidative stress // inferred from mutant p
NM_023258		0.006158922	8.27E-04	1.09	1.27	1.41	Pycard	PYD and CARD domain containing	0001773 // myeloid dendritic cell activation // not recorded///0002218 // activation of innate immune response // inferred from mutant phenotype///0002218 // activation of innate
NM_001110233///NM_001110234//		0.009455777	8.43E-04	1.19	1.29	1.41	Ngfrap1	nerve growth factor receptor (TNFRSF16) associated protein 1	0006915 // apoptotic process // inferred from electronic annotation///0008625 // extrinsic apoptotic signaling pathway via death domain receptors // inferred from physical interacti
NM_010120///XM_00	2.40E-04		0.00103513	1.32	1.30	1.41	Eif1a	eukaryotic translation initiation factor 1A	0006412 // translation // inferred from electronic annotation///0006413 // translational initiation // inferred from electronic annotation
NM_008704	0.00352848	0.001617629	6.82E-04	1.23	1.26	1.41	Nme1	NME/NM23 nucleoside diphosphate kinase 1	0002762 // negative regulation of myeloid leukocyte differentiation // not recorded///0006163 // purine nucleotide metabolic process // not recorded///0006165 // nucleoside dipho
NM_011712			0.0039809	1.18	1.15	1.41	Wbp5	WW domain binding protein 5	0006357 // regulation of transcription from RNA polymerase II promoter // not recorded
NM_134081			0.00228481	-1.02	1.11	1.40	Dnajc9	DnaJ (Hsp40) homolog, subfamily C, member 9	0032781 // positive regulation of ATPase activity // not recorded///0035176 // social behavior // inferred from electronic annotation
NM_001159631///NM_021606///XM		0.003730991	5.51E-04	1.14	1.27	1.40	Nek6	NIMA (never in mitosis gene a)-related expressed kinase 6	0006468 // protein phosphorylation // not recorded///0006915 // apoptotic process // inferred from electronic annotation///0007049 // cell cycle // inferred from electronic annotat
NM_030685			0.00627522	1.02	1.19	1.40	Serp1	stress-associated endoplasmic reticulum protein 1	0001501 // skeletal system development // inferred from mutant phenotype///0006006 // glucose metabolic process // inferred from mutant phenotype///0006486 // protein glycos
NM_026114			0.00613033	1.22	1.35	1.40	Eif2s1	eukaryotic translation initiation factor 2, subunit 1 alpha	0002230 // positive regulation of defense response to virus by host // not recorded///0006412 // translation // inferred from direct assay///0006413 // translational initiation // not re
NM_008039			0.00678349	-1.10	1.41	1.40	Fpr2	formyl peptide receptor 2	0006935 // chemotaxis // inferred from electronic annotation///0007165 // signal transduction // inferred from electronic annotation///0007186 // G-protein coupled receptor signal
NM_023203		0.004964043	3.70E-04	1.11	1.30	1.40	Dctpp1	dCTP pyrophosphatase 1	0009143 // nucleoside triphosphate catabolic process // inferred from direct assay///0051289 // protein homotetramerization // inferred from direct assay
NM_028870///XM_006517418		0.008881584		1.12	1.31	1.40	Cltb	clathrin, light polypeptide (Lcb)	0006886 // intracellular protein transport // inferred from electronic annotation///0016192 // vesicle-mediated transport // inferred from electronic annotation///0072583 // clathrin
NM_013552///XM_006532276///XM_006532278///			7.92E-04	-1.02	1.17	1.40	Hmmr	hyaluronan mediated motility receptor (RHAMM)	
NM_001301373///NM_001301375//		5.74E-04		1.07	1.34	1.40	Fst	follistatin	0000122 // negative regulation of transcription from RNA polymerase II promoter // not recorded///0001501 // skeletal system development // inferred from genetic interaction///00
NM_008231///XM_00	0.00589401	0.001956797		1.17	1.24	1.40	Hdgf	hepatoma-derived growth factor	0000122 // negative regulation of transcription from RNA polymerase II promoter // not recorded///0006351 // transcription, DNA-templated // inferred from electronic annotation/,
NM_001113416///NM_145506///XM_006529397///			0.00646311	1.15	1.15	1.40	Epb41l5	erythrocyte membrane protein band 4.1 like 5	0000904 // cell morphogenesis involved in differentiation // inferred from mutant phenotype///0001701 // in utero embryonic development // inferred from mutant phenotype///00
NM_008776			6.52E-04	1.06	1.30	1.40	Pafah1b3	platelet-activating factor acetylhydrolase, isoform 1b, subunit 3	0006629 // lipid metabolic process // inferred from electronic annotation///0007283 // spermatogenesis // inferred from genetic interaction///0007283 // spermatogenesis // inferre
NM_031863		0.007738151	3.12E-04	-1.05	1.19	1.40	Cenpq	centromere protein Q	
NM_009584///XM_00	0.00795411			1.22	1.19	1.39	Dnajc2	DnaJ (Hsp40) homolog, subfamily C, member 2	0006260 // DNA replication // inferred from mutant phenotype///0006351 // transcription, DNA-templated // inferred from electronic annotation///0006355 // regulation of transcri
NM_025436	2.61E-04	5.88E-05	0.0017175	1.40	1.55	1.39	Msmo1	methylsterol monoxygenase 1	0006629 // lipid metabolic process // inferred from electronic annotation///0006633 // fatty acid biosynthetic process // inferred from electronic annotation///0006694 // steroid bio
NM_008398///XM_006513267///XM		9.65E-04	0.00743213	1.21	1.47	1.39	Itga7	integrin alpha 7	0007155 // cell adhesion // inferred from mutant phenotype///0007155 // cell adhesion // not recorded///0007229 // integrin-mediated signaling pathway // not recorded///000836
NM_001136069///NM	8.22E-04	2.17E-04	8.30E-04	1.32	1.42	1.39	Ldha	lactate dehydrogenase A	0001666 // response to hypoxia // inferred from electronic annotation///0005975 // carbohydrate metabolic process // inferred from electronic annotation///0006089 // lactate met
NM_008252///XM_006509524		0.00182127		-1.18	1.35	1.39	Hmgb2	high mobility group box 2	0001938 // positive regulation of endothelial cell proliferation // not recorded///0002437 // inflammatory response to antigenic stimulus // inferred from mutant phenotype///0006
NM_016692///XM_006526707///XM		8.15E-04	0.00130364	1.00	1.29	1.39	Incenp	inner centromere protein	0000910 // cytokinesis // not recorded///0007049 // cell cycle // inferred from electronic annotation///0007059 // chromosome segregation // not recorded///0007067 // mitotic nu
NM_001146057///NM_001146058///NM_133348//			9.16E-04	1.06	1.16	1.38	Acot7	acyl-CoA thioesterase 7	0009062 // fatty acid catabolic process // inferred from direct assay///0015937 // coenzyme A biosynthetic process // not recorded///0036114 // medium-chain fatty-acyl-CoA catab
NM_018865///XM_006520889		0.001789398	0.00131109	1.07	1.25	1.38	Wisp1	WNT1 inducible signaling pathway protein 1	0001558 // regulation of cell growth // inferred from electronic annotation///0007155 // cell adhesion // not recorded///0007165 // signal transduction // not recorded///0007267 //
NM_025647			0.00938079	1.07	1.25	1.38	Cmpk1	cytidine monophosphate (UMP-CMP) kinase 1	0006139 // nucleobase-containing compound metabolic process // inferred from electronic annotation///0006165 // nucleoside diphosphate phosphorylation // not recorded///0006
NM_008638///XM_006505697///XM_011241245			0.00152557	1.11	1.11	1.38	Mthfd2	methylenetetrahydrofolate dehydrogenase (NAD+ dependent), me	0006730 // one-carbon metabolic process // not recorded///0008152 // metabolic process // inferred from electronic annotation///0009396 // folic acid-containing compound biosy
NM_013671	1.23E-04	4.77E-04		1.30	1.48	1.38	Sod2	superoxide dismutase 2, mitochondrial	0000302 // response to reactive oxygen species // inferred from mutant phenotype///0000303 // response to superoxide // inferred from mutant phenotype///0000303 // response t
NM_001289724///NM_001289725//		0.007112625		1.19	1.37	1.38	Slbp	stem-loop binding protein	0006397 // mRNA processing // inferred from electronic annotation///0006398 // mRNA 3'-end processing by stem-loop binding and cleavage // inferred from direct assay///003326
NM_153510			3.96E-04	-1.03	1.10	1.38	Pilra	paired immunoglobin-like type 2 receptor alpha	0007165 // signal transduction // not recorded///0007169 // transmembrane receptor protein tyrosine kinase signaling pathway // not recorded
NM_001271483///NM	0.00422777	0.003851777	2.37E-04	1.20	1.25	1.38	Clip4	CAP-GLY domain containing linker protein family, member 4	
NM_021288///NR_000040///NR_03		0.004478479		-1.04	1.21	1.38	Tyms///Tyms-ps	thymidylate synthase///thymidylate synthase, pseudogene	0006206 // pyrimidine nucleobase metabolic process // inferred from electronic annotation///0006231 // dTMP biosynthetic process // not recorded///0006235 // dTTP biosynthetic
NM_001083188///NM_001199310//		1.12E-04	0.00107092	1.14	1.46	1.38	Lig1	ligase I, DNA, ATP-dependent	0006260 // DNA replication // inferred from mutant phenotype///0006266 // DNA ligation // not recorded///0006273 // lagging strand elongation // not recorded///0006281 // DNA
NM_153546///XM_011244305		0.007592567		1.20	1.41	1.37	Mboat1	membrane bound O-acyltransferase domain containing 1	0006629 // lipid metabolic process // inferred from electronic annotation///0006644 // phospholipid metabolic process // inferred from electronic annotation///0008654 // phosphol
NM_001160012///NM_008126			0.0010254	1.02	1.28	1.37	Gjb3	gap junction protein, beta 3	0001701 // in utero embryonic development // inferred from electronic annotation///0001890 // placenta development // inferred from mutant phenotype///0007154 // cell commu
NM_013614///XR_864	0.00601853	0.003480981		1.22	1.25	1.37	Gm9115///Odc1	ornithine decarboxylase, structural 1 pseudogene///ornithine deca	0001822 // kidney development // inferred from mutant phenotype///0006595 // polyamine metabolic process // not recorded///0006596 // polyamine biosynthetic process // infer
NM_009196			0.00267002	1.23	1.31	1.37	Slc16a1	solute carrier family 16 (monocarboxylic acid transporters), memb	0006629 // lipid metabolic process // inferred from mutant phenotype///0006810 // transport // inferred from electronic annotation///0015718 // monocarboxylic acid transport // i
NM_008404///XM_006513271///XM		4.06E-04	0.00388254	-1.23	1.38	1.37	Itgb2	integrin beta 2	0002523 // leukocyte migration involved in inflammatory response // not recorded///0007155 // cell adhesion // inferred from genetic interaction///0007155 // cell adhesion // infer
NM_029391///XM_006539673///XM_011250458			0.00825852	1.08	1.28	1.37	Rab4b	RAB4B, member RAS oncogene family	0006810 // transport // inferred from electronic annotation///0006886 // intracellular protein transport // not recorded///0007264 // small GTPase mediated signal transduction // in
NM_001276408///NM_001276409//		0.003153431	0.00501176	-1.02	1.33	1.37	Fn1	fibronectin 1	0001525 // angiogenesis // inferred from electronic annotation///0001775 // cell activation // not recorded///0001932 // regulation of protein phosphorylation // not recorded///00
NM_008532			0.00611229	1.06	1.13	1.37	Epcam	epithelial cell adhesion molecule	0001657 // ureteric bud development // inferred from expression pattern///0008284 // positive regulation of cell proliferation // not recorded///0023019 // signal transduction involv
NM_023731			0.00570134	1.17	1.24	1.37	Ccdc86	coiled-coil domain containing 86	
NM_001304830///NM	3.39E-04	0.001258025		1.38	1.35	1.37	Maff	v-maf musculoaponeurotic fibrosarcoma oncogene family, protein	0001701 // in utero embryonic development // inferred from genetic interaction///0006351 // transcription, DNA-templated // inferred from electronic annotation///0006355 // regu
NM_001042652///NM_133851///XM_011239246			0.00462363	1.02	1.25	1.37	Nusap1	nucleolar and spindle associated protein 1	0000070 // mitotic sister chromatid segregation // not recorded///0000226 // microtubule cytoskeleton organization // inferred from electronic annotation///0000281 // mitotic cyto
NM_011082	8.77E-05		0.00885925	2.01	1.55	1.36	Pigr	polymeric immunoglobulin receptor	0001580 // detection of chemical stimulus involved in sensory perception of bitter taste // not recorded///0001895 // retina homeostasis // inferred from electronic annotation///000

RefSeq Transcript ID	p (24h n=460)	p (48h n=657)	p (72h n=805)	FC 24h	FC 48h	FC 72h	Gene Symbol	Gene Title	Gene Ontology Biological Process
NM_001113248///NM_009188///XM		5.85E-04	0.00715407	1.10	**1.44**	**1.36**	***Sin3b***	transcriptional regulator, SIN3B (yeast)	0000122 // negative regulation of transcription from RNA polymerase II promoter // not recorded///0006351 // transcription, DNA-templated // inferred from electronic annotation/
NM_001301414///NM_146144///XM_006502939			0.00988333	1.07	1.16	**1.36**	***Usp1***	ubiquitin specific peptidase 1	0001501 // skeletal system development // inferred from mutant phenotype///0006281 // DNA repair // inferred from electronic annotation///0006282 // regulation of DNA repair /
NM_013463		0.004352849	0.00464255	1.08	**1.22**	**1.36**	***Gla***	galactosidase, alpha	0005975 // carbohydrate metabolic process // inferred from electronic annotation///0008152 // metabolic process // inferred from electronic annotation///0009311 // oligosacchari
NM_026117///NM_2C	0.00720425	0.002177797		**1.29**	**1.31**	1.36	***Dedd2***	death effector domain-containing DNA binding protein 2	0006351 // transcription, DNA-templated // inferred from electronic annotation///0006355 // regulation of transcription, DNA-templated // inferred from electronic annotation///00
NM_011081///XM_006528758///XM_011247793			0.00791023	1.29	1.13	**1.36**	***Piga***	phosphatidylinositol glycan anchor biosynthesis, class A	0006506 // GPI anchor biosynthetic process // inferred from mutant phenotype///0009058 // biosynthetic process // inferred from electronic annotation
NM_008608			0.00408104	-1.02	1.24	**1.36**	***Mmp14***	matrix metallopeptidase 14 (membrane-inserted)	0001503 // ossification // inferred from genetic interaction///0001525 // angiogenesis // inferred from electronic annotation///0001541 // ovarian follicle development // inferred fro
NM_019641			0.00389318	-1.03	1.12	**1.36**	***Stmn1***	stathmin 1	0007019 // microtubule depolymerization // not recorded///0007052 // mitotic spindle organization // not recorded///0007275 // multicellular organismal development // inferred fr
NM_013770		0.007988815	0.00126833	1.05	**1.32**	**1.36**	***Slc25a10***	solute carrier family 25 (mitochondrial carrier, dicarboxylate trans	0006810 // transport // inferred from electronic annotation///0006817 // phosphate ion transport // not recorded///0006839 // mitochondrial transport // not recorded///0008272 ,
NM_008361///XM_006498795			2.49E-04	-1.08	1.07	**1.35**	***Il1b***	interleukin 1 beta	0000122 // negative regulation of transcription from RNA polymerase II promoter // not recorded///0000165 // MAPK cascade // not recorded///0000187 // activation of MAPK activ
NM_001311790///NM_009982///XM_006507319//			0.00122507	-1.03	1.16	**1.35**	***Ctsc***	cathepsin C	0001913 // T cell mediated cytotoxicity // inferred from genetic interaction///0006508 // proteolysis // inferred from mutant phenotype///0006508 // proteolysis // not recorded///0
NM_177305			0.00925302	-1.01	1.21	**1.35**	***Arl4c***	ADP-ribosylation factor-like 4C	0006810 // transport // inferred from electronic annotation///0007264 // small GTPase mediated signal transduction // inferred from electronic annotation///0032456 // endocytic re
NM_178389///XM_006539217///XM		0.006697938	4.93E-04	1.14	**1.24**	**1.35**	***Gale***	galactose-4-epimerase, UDP	0005975 // carbohydrate metabolic process // inferred from electronic annotation///0006012 // galactose metabolic process // inferred from direct assay///0019388 // galactose cat
NM_010344		7.82E-04	0.00190678	1.19	**1.37**	**1.35**	***Gsr***	glutathione reductase	0006749 // glutathione metabolic process // inferred by curator///0006749 // glutathione metabolic process // not recorded///0007283 // spermatogenesis // not recorded///00454
NM_133722	0.00952294	0.007335682	0.00188324	**1.15**	**1.21**	**1.35**	***Abhd17c***	abhydrolase domain containing 17C	0008152 // metabolic process // inferred from electronic annotation
NM_010185			7.04E-04	1.05	1.22	**1.35**	***Fcer1g***	Fc receptor, IgE, high affinity I, gamma polypeptide	0001798 // positive regulation of type IIa hypersensitivity // inferred from mutant phenotype///0001805 // positive regulation of type III hypersensitivity // inferred from mutant phen
NM_001285423///NM	5.90E-04	7.86E-04	0.00425993	**1.33**	**1.46**	**1.35**	***Esd***	esterase D/formylglutathione hydrolase	0046294 // formaldehyde catabolic process // inferred from electronic annotation
NM_019568///XM_00	4.98E-05			**1.50**	1.24	1.35	***Cxcl14***	chemokine (C-X-C motif) ligand 14	0006955 // immune response // inferred from electronic annotation///0045662 // negative regulation of myoblast differentiation // inferred from mutant phenotype///0048839 // in
NM_011479	0.00445218	2.14E-04	0.00382231	**1.15**	**1.38**	**1.35**	***Sptlc2***	serine palmitoyltransferase, long chain base subunit 2	0006629 // lipid metabolic process // inferred from electronic annotation///0006665 // sphingolipid metabolic process // inferred from electronic annotation///0006686 // sphingomy
NM_001081212			0.00880209	1.13	1.19	**1.35**	***Irs2***	insulin receptor substrate 2	0002053 // positive regulation of mesenchymal cell proliferation // inferred from genetic interaction///0002903 // negative regulation of B cell apoptotic process // inferred from mut
NM_176979///XM_006511706///XM_006511707			8.66E-04	-1.07	1.19	**1.35**	***Topbp1***	topoisomerase (DNA) II binding protein 1	0006281 // DNA repair // inferred from electronic annotation///0006974 // cellular response to DNA damage stimulus // not recorded///0007131 // reciprocal meiotic recombination
NM_001048061///NM_010448			0.00176346	1.06	1.14	**1.35**	***Hnrnpab***	heterogeneous nuclear ribonucleoprotein A/B	0000122 // negative regulation of transcription from RNA polymerase II promoter // inferred from genetic interaction///0001837 // epithelial to mesenchymal transition // inferred fr
NM_178376			0.00603244	-1.01	1.12	**1.35**	***Rraga***	Ras-related GTP binding A	0006915 // apoptotic process // inferred from electronic annotation///0008219 // cell death // not recorded///0009267 // cellular response to starvation // not recorded///0010506 ,
NM_001177609///NM_001177610///NM_011665//			0.00328841	1.06	1.27	**1.34**	***LOC102641751///Ube2i***	SUMO-conjugating enzyme UBC9///ubiquitin-conjugating enzyme	0000122 // negative regulation of transcription from RNA polymerase II promoter // inferred from mutant phenotype///0000122 // negative regulation of transcription from RNA poly
NM_007793		2.35E-04	0.00707683	1.15	**1.48**	**1.34**	***Cstb***	cystatin B	0008344 // adult locomotory behavior // inferred from mutant phenotype///0010466 // negative regulation of peptidase activity // not recorded///0010951 // negative regulation of
NM_025407			0.00887697	1.07	1.34	**1.34**	***Uqcrc1***	ubiquinol-cytochrome c reductase core protein 1	0006122 // mitochondrial electron transport, ubiquinol to cytochrome c // inferred from direct assay///0006810 // transport // inferred from electronic annotation///0009060 // aer
NM_009127	0.00303949			**1.21**	1.33	1.34	***Scd1***	stearoyl-Coenzyme A desaturase 1	0006629 // lipid metabolic process // inferred from electronic annotation///0006631 // fatty acid metabolic process // inferred from electronic annotation///0006633 // fatty acid bio
NR_002840///NR_028543		0.008075489		1.47	**1.57**	1.34	***Gas5///Snord47***	growth arrest specific 5///small nucleolar RNA, C/D box 47	
NM_146235			0.00234453	1.07	1.12	**1.34**	***Ercc6l***	excision repair cross-complementing rodent repair deficiency com	0007049 // cell cycle // inferred from electronic annotation///0007067 // mitotic nuclear division // inferred from electronic annotation///0008152 // metabolic process // inferred fr
NM_134122			0.00520098	-1.02	1.21	**1.34**	***Nrm***	nurim (nuclear envelope membrane protein)	
NM_134009			0.00122125	1.03	1.15	**1.34**	***Ncln***	nicalin homolog (zebrafish)	0009966 // regulation of signal transduction // inferred from electronic annotation///0031648 // protein destabilization // not recorded///0043254 // regulation of protein complex a
NM_011133///XM_006515598///XM_006515599//			0.00143837	1.05	1.21	**1.34**	***Pole2***	polymerase (DNA directed), epsilon 2 (p59 subunit)	0006260 // DNA replication // inferred from electronic annotation///0006261 // DNA-dependent DNA replication // not recorded///0042276 // error-prone translesion synthesis // no
NM_009026///XM_011248826			0.0026964	1.14	1.22	**1.34**	***Rasd1***	RAS, dexamethasone-induced 1	0007165 // signal transduction // inferred from electronic annotation///0007263 // nitric oxide mediated signal transduction // not recorded///0007264 // small GTPase mediated sig
NM_025569///NM_029392		2.49E-04		1.15	**1.57**	1.34	***Mgst3***	microsomal glutathione S-transferase 3	0010243 // response to organonitrogen compound // inferred from electronic annotation///0055114 // oxidation-reduction process // not recorded
NM_001025427///NM_001039356//		0.00587571	0.00137316	1.11	**1.38**	**1.33**	***Hmga1///Hmga1-rs1***	high mobility group AT-hook 1///high mobility group AT-hook I, rel	0006284 // base-excision repair // not recorded///0006351 // transcription, DNA-templated // inferred from electronic annotation///0006355 // regulation of transcription, DNA-tem
NM_016867///XM_01	0.00494418			**1.15**	1.34	1.33	***Gipc2***	GIPC PDZ domain containing family, member 2	
NM_011662			0.00186258	-1.25	1.09	**1.33**	***Tyrobp***	TYRO protein tyrosine kinase binding protein	0002281 // macrophage activation involved in immune response // inferred from mutant phenotype///0002283 // neutrophil activation involved in immune response // inferred from
NM_138656///NR_02	0.00438684		0.0038585	**1.18**	1.35	**1.33**	***Mvd***	mevalonate (diphospho) decarboxylase	0006629 // lipid metabolic process // inferred from electronic annotation///0006694 // steroid biosynthetic process // inferred from electronic annotation///0006695 // cholesterol b
NM_001302389///NM_001302390///NM_0013024			0.00353198	1.12	1.23	**1.33**	***Plpp2***	phospholipid phosphatase 2	0006644 // phospholipid metabolic process // not recorded///0007165 // signal transduction // not recorded///0030148 // sphingolipid biosynthetic process // not recorded///00468
NM_016897		0.002421028	0.00782595	1.12	**1.30**	**1.33**	***Timm23***	translocase of inner mitochondrial membrane 23	0006810 // transport // inferred from electronic annotation///0006886 // intracellular protein transport // inferred from electronic annotation///0015031 // protein transport // infer
NM_001177352///NM	2.04E-04	3.16E-04		**1.31**	**1.58**	1.33	***Myc***	myelocytomatosis oncogene	0000122 // negative regulation of transcription from RNA polymerase II promoter // inferred from direct assay///0000122 // negative regulation of transcription from RNA polymeras
NM_018868///XM_00	3.43E-04	1.45E-04		**1.38**	**1.51**	1.33	***Nop58***	NOP58 ribonucleoprotein	0000154 // rRNA modification // not recorded///0006608 // snRNP protein import into nucleus // not recorded///0042254 // ribosome biogenesis // inferred from electronic annotat
NM_021890///XM_006527247///XM_006527248			6.92E-04	-1.02	1.24	**1.33**	***Fads3***	fatty acid desaturase 3	0006629 // lipid metabolic process // inferred from electronic annotation///0006631 // fatty acid metabolic process // inferred from electronic annotation///0006633 // fatty acid bio
NM_001163359///NM_001163360//		0.00676525	0.00273766	1.08	**1.24**	**1.33**	***Fignl1***	fidgetin-like 1	0001649 // osteoblast differentiation // inferred from mutant phenotype///0010569 // regulation of double-strand break repair via homologous recombination // not recorded///003
NM_010188///XM_006496658///XM_006496659//			0.00803502	-1.10	1.12	**1.33**	***Fcgr3***	Fc receptor, IgG, low affinity III	0001788 // antibody-dependent cellular cytotoxicity // inferred from mutant phenotype///0001798 // positive regulation of type IIa hypersensitivity // inferred from mutant phenotyp
NM_025844///XM_00	0.00209594		0.0054329	**1.53**	1.03	**1.33**	***Chordc1***	cysteine and histidine-rich domain (CHORD)-containing, zinc-bindin	0010824 // regulation of centrosome duplication // inferred from mutant phenotype///0061077 // chaperone-mediated protein folding // inferred from direct assay///1900034 // reg
NM_007856///XM_006508476///XM_006508478//			0.00593382	1.16	1.28	**1.33**	***Dhcr7***	7-dehydrocholesterol reductase	0001568 // blood vessel development // inferred from mutant phenotype///0006629 // lipid metabolic process // inferred from electronic annotation///0006694 // steroid biosynthe
NM_198613			0.00448233	1.00	1.24	**1.32**	***Ap2s1***	adaptor-related protein complex 2, sigma 1 subunit	0006810 // transport // inferred from electronic annotation///0006886 // intracellular protein transport // inferred from electronic annotation///0006897 // endocytosis // inferred fr
NM_001310469///NM	4.33E-05		0.00640368	**1.69**	1.31	**1.32**	***Osmr***	oncostatin M receptor	0007166 // cell surface receptor signaling pathway // traceable author statement///0008284 // positive regulation of cell proliferation // not recorded///0034097 // response to cytok
NM_007950	0.00442727	0.001884708		**1.37**	**1.66**	1.32	***Ereg***	epiregulin	0000187 // activation of MAPK activity // not recorded///0001525 // angiogenesis // inferred from electronic annotation///0001550 // ovarian cumulus expansion // not recorded///(
NM_001199431///NM_001199432///NM_0011994			0.00999741	1.03	1.22	**1.32**	***Dnmt1***	DNA methyltransferase (cytosine-5) 1	0000122 // negative regulation of transcription from RNA polymerase II promoter // inferred from mutant phenotype///0000183 // chromatin silencing at rDNA // traceable author st
NM_023065			0.00112659	-1.15	1.13	**1.32**	***Ifi30***	interferon gamma inducible protein 30	0002376 // immune system process // inferred from electronic annotation///0019886 // antigen processing and presentation of exogenous peptide antigen via MHC class II // inferred
NM_001302346///NM_001302347///NM_008773			0.00770895	1.18	1.17	**1.32**	***P2ry2***	purinergic receptor P2Y, G-protein coupled 2	0001701 // in utero embryonic development // inferred from electronic annotation///0007165 // signal transduction // inferred from electronic annotation///0007186 // G-protein co
NM_022415	0.00498024	7.36E-05	0.00581402	**1.16**	**1.45**	**1.32**	***Ptges***	prostaglandin E synthase	0001516 // prostaglandin biosynthetic process // not recorded///0002526 // acute inflammatory response // inferred from electronic annotation///0002544 // chronic inflammatory
NM_009270		2.66E-04		1.31	**1.47**	1.32	***Sqle***	squalene epoxidase	0006725 // cellular aromatic compound metabolic process // not recorded///0008203 // cholesterol metabolic process // not recorded///0010033 // response to organic substance /
NM_001110146///NM_021400///XM_006529963//			0.00204311	1.21	1.62	**1.32**	***Prg4***	proteoglycan 4 (megakaryocyte stimulating factor, articular superf	0006898 // receptor-mediated endocytosis // inferred from electronic annotation///0006955 // immune response // inferred from electronic annotation///0018149 // peptide cross-l
NM_001267707///NM	0.00151781		0.00858869	**1.53**	1.23	**1.32**	***Slco1a5***	solute carrier organic anion transporter family, member 1a5	0006810 // transport // inferred from electronic annotation///0006811 // ion transport // inferred from electronic annotation///0008206 // bile acid metabolic process // not recorde
NM_019553		0.004509864		1.19	**1.35**	1.32	***Ddx21***	DEAD (Asp-Glu-Ala-Asp) box polypeptide 21	0001649 // osteoblast differentiation // not recorded///0006351 // transcription, DNA-templated // inferred from electronic annotation///0006364 // rRNA processing // inferred fror
NM_001164336///NM_001164337//		0.007155914		1.09	**1.19**	1.31	***5-Mar***	membrane-associated ring finger (C3HC4) 5	0000209 // protein polyubiquitination // not recorded///0016567 // protein ubiquitination // inferred from electronic annotation///0051865 // protein autoubiquitination // not recor
NM_017373///XM_00	0.00101011	0.001408788		**1.64**	**1.57**	1.31	***Nfil3***	nuclear factor, interleukin 3, regulated	0000122 // negative regulation of transcription from RNA polymerase II promoter // not recorded///0006351 // transcription, DNA-templated // inferred from electronic annotation/
NM_001081214///NM	1.31E-04	0.005732399	6.73E-04	**1.47**	**1.53**	**1.31**	***Pprc1***	peroxisome proliferative activated receptor, gamma, coactivator-r	0006351 // transcription, DNA-templated // inferred from electronic annotation///0006355 // regulation of transcription, DNA-templated // inferred from electronic annotation///00
NM_011112///XM_006515592///XM_006515593//			0.00417569	-1.09	1.00	**1.31**	***Papola***	poly (A) polymerase alpha	0000398 // mRNA splicing, via spliceosome // ---///0006369 // termination of RNA polymerase II transcription // ---///0006378 // mRNA polyadenylation // not recorded///0006397 /
NM_024495	0.00437933	2.95E-04	0.00188418	**1.19**	**1.34**	**1.31**	***Car13***	carbonic anhydrase 13	0006730 // one-carbon metabolic process // inferred from electronic annotation
NM_001005767///XR_384575		0.002629318		1.09	**1.25**	1.31	***Parl***	presenilin associated, rhomboid-like	0006508 // proteolysis // inferred from genetic interaction///0006508 // proteolysis // inferred from mutant phenotype///0006508 // proteolysis // not recorded///0016485 // protei
NM_028355///XM_006503423			0.00329907	1.09	1.13	**1.31**	***Ndc1***	NDC1 transmembrane nucleoporin	0006810 // transport // inferred from electronic annotation///0007129 // synapsis // inferred from mutant phenotype///0007283 // spermatogenesis // inferred from mutant phenot
NM_133757///XM_011249288///XR_388542///XR_			0.00139282	1.12	1.28	**1.31**	***Pgs1***	phosphatidylglycerophosphate synthase 1	0006629 // lipid metabolic process // inferred from electronic annotation///0006655 // phosphatidylglycerol biosynthetic process // inferred from sequence or structural similarity///(
NM_001001491			0.00662347	1.05	1.06	**1.31**	***Tpm4***	tropomyosin 4	0001649 // osteoblast differentiation // inferred from electronic annotation
NM_001136079///NM	0.00170816	0.001765052		**1.42**	**1.42**	1.31	***Ptger4***	prostaglandin E receptor 4 (subtype EP4)	0001934 // positive regulation of protein phosphorylation // not recorded///0002792 // negative regulation of peptide secretion // not recorded///0006955 // immune response // in
NM_030724///XM_006497039///XM_006497040//			9.86E-04	1.05	**1.13**	**1.31**	***Uck2***	uridine-cytidine kinase 2	0006206 // pyrimidine nucleobase metabolic process // not recorded///0006222 // UMP biosynthetic process // ---///0006238 // CMP salvage // inferred from electronic annotation/
NM_145403		9.87E-04		1.22	**1.37**	1.31	***Tmprss4***	transmembrane protease, serine 4	0006508 // proteolysis // inferred from electronic annotation///0006898 // receptor-mediated endocytosis // inferred from electronic annotation
NM_025567		0.007171161		**1.10**	**1.26**	1.31	***Cyc1***	cytochrome c-1	0006810 // transport // inferred from electronic annotation///0033762 // response to glucagon // not recorded///0055114 // oxidation-reduction process // inferred from electronic
NM_008871	2.83E-04	0.005363897		**1.62**	**1.43**	1.30	***Serpine1***	serine (or cysteine) peptidase inhibitor, clade E, member 1	0001300 // chronological cell aging // inferred from electronic annotation///0001525 // angiogenesis // inferred from electronic annotation///0001890 // placenta development // in
NM_177613///XM_006513482///XM_006513483			0.00954752	1.06	1.24	**1.30**	***Cdc34***	cell division cycle 34	0000209 // protein polyubiquitination // not recorded///0006513 // protein monoubiquitination // inferred from sequence or structural similarity///0007049 // cell cycle // inferred fr
NM_010721			0.00505408	1.03	1.08	**1.30**	***Lmnb1***	lamin B1	0031662 // positive regulation of cyclin-dependent protein serine/threonine kinase activity involved in G2/M transition of mitotic cell cycle // non-traceable author statement///00463
NM_013538///XM_006505564		6.06E-04	0.00275997	1.06	**1.28**	**1.30**	***Cdca3***	cell division cycle associated 3	0007049 // cell cycle // inferred from electronic annotation///0007067 // mitotic nuclear division // inferred from electronic annotation///0016567 // protein ubiquitination // inferre
NM_009396///XM_006515792///XM		2.94E-04	0.00157813	1.10	**1.34**	**1.30**	***Tnfaip2***	tumor necrosis factor, alpha-induced protein 2	0001525 // angiogenesis // inferred from electronic annotation///0006887 // exocytosis // not recorded///0007275 // multicellular organismal development // inferred from electron
NM_001312649///NM_007801///XM		4.59E-04	0.00267766	-1.06	**1.45**	**1.30**	***Ctsh***	cathepsin H	0001656 // metanephros development // inferred from sequence or structural similarity///0001913 // T cell mediated cytotoxicity // inferred from genetic interaction///0002250 // a
NM_019755///XM_011239878///XM		4.38E-04	0.00210417	1.05	**1.38**	**1.30**	***Gm13669///Plp2***	predicted gene 13669///proteolipid protein 2	
NM_018820///XM_00	3.93E-04	0.007304909		**1.31**	**1.46**	1.30	***Sertad1***	SERTA domain containing 1	0006351 // transcription, DNA-templated // inferred from electronic annotation///0006355 // regulation of transcription, DNA-templated // inferred from electronic annotation///00
NM_053075		0.004815442		1.04	**1.26**	1.30	***Rheb***	Ras homolog enriched in brain	0007165 // signal transduction // inferred from electronic annotation///0007264 // small GTPase mediated signal transduction // not recorded///0032006 // regulation of TOR signali
NM_011519///XM_006515045///XM_006515046//			0.00327691	-1.02	1.12	**1.30**	***Sdc1***	syndecan 1	0001657 // ureteric bud development // inferred from electronic annotation///0006954 // inflammatory response // not recorded///0009636 // response to toxic substance // inferre
NM_130889///XM_011250089		0.005539734	0.00322038	1.03	**1.21**	**1.30**	***Anp32b***	acidic (leucine-rich) nuclear phosphoprotein 32 family, member B	0000082 // G1/S transition of mitotic cell cycle // not recorded///0001944 // vasculature development // inferred from mutant phenotype///0006334 // nucleosome assembly // infe
NM_025550		0.007823732		1.08	**1.21**	1.30	***Psmd6***	proteasome (prosome, macropain) 26S subunit, non-ATPase, 6	0043161 // proteasome-mediated ubiquitin-dependent protein catabolic process // not recorded
NM_019502///XM_006507374///XM_006508382//			0.0093123	1.12	1.38	**1.30**	***Dnhd1///Timm10b***	dynein heavy chain domain 1///translocase of inner mitochondrial	0006626 // protein targeting to mitochondrion // not recorded///0006810 // transport // inferred from electronic annotation///0007018 // microtubule-based movement // inferred
NM_011313		0.005488276		1.02	**1.32**	1.29	***S100a6***	S100 calcium binding protein A6 (calcyclin)	0034220 // ion transmembrane transport // not recorded
NM_001314054///NM_031168			0.00949267	1.14	1.25	**1.29**	***Il6***	interleukin 6	0001781 // neutrophil apoptotic process // inferred from direct assay///0001781 // neutrophil apoptotic process // inferred from mutant phenotype///0001781 // neutrophil apoptot
NM_016966///NR_076393		0.004839626	0.00411586	1.00	**1.34**	**1.29**	***Gm6756///Phgdh***	3-phosphoglycerate dehydrogenase pseudogene///3-phosphoglyc	0006541 // glutamine metabolic process // inferred from mutant phenotype///0006544 // glycine metabolic process // inferred from mutant phenotype///0006563 // L-serine metab

RefSeq Transcript ID	p (24h n=460)	p (48h n=657)	p (72h n=805)	FC 24h	FC 48h	FC 72h	Gene Symbol	Gene Title	Gene Ontology Biological Process
NM_013594///XM_00	0.00171644			1.31	1.18	1.29	***Mbd1***	methyl-CpG binding domain protein 1	0000122 // negative regulation of transcription from RNA polymerase II promoter // not recorded///0006346 // methylation-dependent chromatin silencing // not recorded///00063
NM_001042513///NM	6.29E-04	0.008700395	0.00136108	1.27	1.42	1.29	***Txnrd1***	thioredoxin reductase 1	0001707 // mesoderm formation // inferred from mutant phenotype///0006979 // response to oxidative stress // not recorded///0007369 // gastrulation // inferred from mutant ph
NM_022325			0.00289558	-1.02	1.22	1.29	***Ctsz***	cathepsin Z	0006508 // proteolysis // inferred from electronic annotation///0051603 // proteolysis involved in cellular protein catabolic process // not recorded///0060441 // epithelial tube bran
NM_001204875///NM_023871			0.00252581	1.12	1.11	1.29	***Set***	SET nuclear oncogene	0006334 // nucleosome assembly // inferred from electronic annotation///0043524 // negative regulation of neuron apoptotic process // not recorded///0045892 // negative regulat
NM_007825///XM_006535383///XM		1.68E-04		1.21	1.36	1.29	***Cyp7b1***	cytochrome P450, family 7, subfamily b, polypeptide 1	0006629 // lipid metabolic process // inferred from electronic annotation///0006699 // bile acid biosynthetic process // inferred from direct assay///0007586 // digestion // traceable
NM_027519///XM_00	0.00355944	0.002369367		1.39	1.41	1.29	***Medag***	mesenteric estrogen dependent adipogenesis	0045600 // positive regulation of fat cell differentiation // inferred from mutant phenotype
NM_029595			0.00584465	1.03	1.25	1.29	***Pbp2***	phosphatidylethanolamine binding protein 2	0010033 // response to organic substance // inferred from electronic annotation///0010243 // response to organonitrogen compound // inferred from electronic annotation///00104
NM_001013368///XM_006540553///XM_00654055			0.00422818	1.10	1.19	1.29	***E2f8***	E2F transcription factor 8	0000122 // negative regulation of transcription from RNA polymerase II promoter // inferred from mutant phenotype///0000122 // negative regulation of transcription from RNA poly
NM_010253			0.0032902	1.05	1.21	1.29	***Gal***	galanin	0006954 // inflammatory response // not recorded///0007218 // neuropeptide signaling pathway // inferred from mutant phenotype///0007399 // nervous system development // in
NM_001291348///NM_001291349//		0.005597941		1.15	1.28	1.29	***Anapc15***	anaphase prompoting complex C subunit 15	0007049 // cell cycle // inferred from electronic annotation///0007067 // mitotic nuclear division // inferred from electronic annotation///0051301 // cell division // inferred from ele
NM_001198933///NM	0.00813009		0.00742388	1.15	1.33	1.29	***Me1***	malic enzyme 1, NADP(+)-dependent, cytosolic	0006108 // malate metabolic process // not recorded///0006108 // malate metabolic process // inferred from sequence or structural similarity///0009725 // response to hormone //
NM_024184		0.008237724	0.00871963	-1.06	1.22	1.29	***Asf1b***	anti-silencing function 1B histone chaperone	0006333 // chromatin assembly or disassembly // inferred from electronic annotation///0006334 // nucleosome assembly // inferred from direct assay///0006335 // DNA replication
NM_008239	0.00535609	0.007140858		1.23	1.26	1.29	***Foxq1***	forkhead box Q1	0006351 // transcription, DNA-templated // inferred from electronic annotation///0006355 // regulation of transcription, DNA-templated // inferred from electronic annotation///00
NM_001033313			0.00138672	1.06	1.24	1.29	***Pdap1***	PDGFA associated protein 1	0048008 // platelet-derived growth factor receptor signaling pathway // not recorded
NM_177638///XM_006524163///XM_006524164//			0.00333495	1.10	1.23	1.28	***Crb3***	crumbs family member 3	0045198 // establishment of epithelial cell apical/basal polarity // not recorded///0045216 // cell-cell junction organization // not recorded///0072659 // protein localization to plasm
NM_133771///XM_006525109///XM		0.004369582	0.00518366	1.13	1.26	1.28	***Memo1***	mediator of cell motility 1	0032886 // regulation of microtubule-based process // not recorded
NM_007991///XM_00	0.00742847		0.00102621	1.17	1.23	1.28	***Fbl///LOC102643269***	fibrillarin///rRNA 2'-O-methyltransferase fibrillarin	0000494 // box C/D snoRNA 3'-end processing // not recorded///0001649 // osteoblast differentiation // not recorded///0006364 // rRNA processing // inferred from electronic anno
NM_024214///XR_878878///XR_878		0.002247344		1.05	1.28	1.28	***Tomm20***	translocase of outer mitochondrial membrane 20 homolog (yeast)	0006605 // protein targeting // inferred from electronic annotation///0006626 // protein targeting to mitochondrion // not recorded///0006810 // transport // inferred from electron
NM_007574		0.001288241		-1.04	1.29	1.28	***C1qc***	complement component 1, q subcomponent, C chain	0002376 // immune system process // inferred from electronic annotation///0006958 // complement activation, classical pathway // inferred from electronic annotation///0030853 /
NM_008062///NM_01	0.00653897	0.00191245	0.00917291	1.15	1.45	1.28	***G6pdx///G6pd2***	glucose-6-phosphate dehydrogenase X-linked///glucose-6-phospha	0001816 // cytokine production // inferred from mutant phenotype///0001998 // angiotensin mediated vasoconstriction involved in regulation of systemic arterial blood pressure // i
NM_001109684///NM_001109685///NM_144531			0.00747339	1.21	1.29	1.28	***Kazn***	kazrin, periplakin interacting protein	0031424 // keratinization // inferred from electronic annotation
NM_001135151///NM	8.03E-04	7.54E-04	9.73E-04	1.29	1.28	1.28	***Slc39a14***	solute carrier family 39 (zinc transporter), member 14	0006810 // transport // inferred from electronic annotation///0006811 // ion transport // inferred from electronic annotation///0006826 // iron ion transport // inferred from direct
NM_009112///XM_011251865		0.003748216		1.01	1.30	1.28	***S100a10///LOC105242499***	S100 calcium binding protein A10 (calpactin)///protein S100-A10-li	0001765 // membrane raft assembly // not recorded///0006900 // membrane budding // not recorded///0043547 // positive regulation of GTPase activity // not recorded///005109
NM_007838			0.00986295	1.01	1.06	1.28	***Ddost***	dolichyl-di-phosphooligosaccharide-protein glycotransferase	0006486 // protein glycosylation // inferred from sequence or structural similarity///0006486 // protein glycosylation // inferred from electronic annotation///0006487 // protein N-li
NM_029639		3.60E-04	0.00107309	1.00	1.34	1.28	***Plet1***	placenta expressed transcript 1	0001953 // negative regulation of cell-matrix adhesion // inferred from mutant phenotype///0030154 // cell differentiation // inferred from electronic annotation///0030335 // positi
NM_029478///XM_00	0.00115548			1.36	1.31	1.27	***Vmp1***	vacuole membrane protein 1	0006887 // exocytosis // not recorded///0006914 // autophagy // inferred from electronic annotation///0007029 // endoplasmic reticulum organization // not recorded///0007030 /
NM_175451			0.0014105	1.06	1.15	1.27	***Ckap4***	cytoskeleton-associated protein 4	
NM_008021///NM_027697			0.00156567	1.05	1.15	1.27	***Foxm1///4933413G19Rik***	forkhead box M1///RIKEN cDNA 4933413G19 gene	0000086 // G2/M transition of mitotic cell cycle // not recorded///0000122 // negative regulation of transcription from RNA polymerase II promoter // not recorded///0000302 // res
NM_013898	0.00472442	0.00271019	0.00753921	1.22	1.37	1.27	***Timm8a1***	translocase of inner mitochondrial membrane 8A1	0006626 // protein targeting to mitochondrion // not recorded///0006810 // transport // inferred from electronic annotation///0015031 // protein transport // inferred from electron
NR_024067		0.003965239		1.05	1.37	1.27	***Snhg6***	small nucleolar RNA host gene 6	
NM_001159866///NM_019562///XM		0.004944335		1.06	1.38	1.27	***Uchl5***	ubiquitin carboxyl-terminal esterase L5	0006281 // DNA repair // inferred from electronic annotation///0006310 // DNA recombination // inferred from electronic annotation///0006351 // transcription, DNA-templated // i
NM_023418			0.00500534	1.06	1.21	1.27	***Pgam1***	phosphoglycerate mutase 1	0006094 // gluconeogenesis // ---///0006096 // glycolytic process // not recorded///0006110 // regulation of glycolytic process // not recorded///0006470 // protein dephosphorylati
NM_011655		0.005062215		-1.01	1.25	1.27	***Tubb5***	tubulin, beta 5 class I	0006457 // protein folding // traceable author statement///0007017 // microtubule-based process // inferred from direct assay///0007017 // microtubule-based process // non-trace
NM_001284328///NM_001311113//		0.005298061		1.23	1.48	1.26	***Syncrip***	synaptotagmin binding, cytoplasmic RNA interacting protein	0001649 // osteoblast differentiation // not recorded///0006397 // mRNA processing // inferred from electronic annotation///0008380 // RNA splicing // inferred from electronic ann
NM_023044		4.80E-05		1.01	1.47	1.26	***Slc15a3***	solute carrier family 15, member 3	0006810 // transport // inferred from electronic annotation///0006857 // oligopeptide transport // inferred from electronic annotation///0015031 // protein transport // inferred fro
NM_025380	0.00187606			1.50	1.44	1.26	***Eef1e1***	eukaryotic translation elongation factor 1 epsilon 1	0006281 // DNA repair // non-traceable author statement///0006412 // translation // inferred from electronic annotation///0006414 // translational elongation // not recorded///00
NM_021456	1.89E-05			1.41	1.27	1.26	***Ces1g***	carboxylesterase 1G	0008152 // metabolic process // inferred from electronic annotation///0010468 // regulation of gene expression // inferred from mutant phenotype///0016042 // lipid catabolic proc
NM_009191///XM_006507488///XM_006507489//			0.00354966	1.11	1.08	1.26	***Clpb***	ClpB caseinolytic peptidase B	0008152 // metabolic process // inferred from electronic annotation///0034605 // cellular response to heat // not recorded
NM_010902///NR_13	0.00234944			1.30	1.28	1.26	***Nfe2l2***	nuclear factor, erythroid derived 2, like 2	0006351 // transcription, DNA-templated // inferred from electronic annotation///0006355 // regulation of transcription, DNA-templated // inferred from direct assay///0006366 // t
NM_008828	0.00827432			1.23	1.36	1.26	***Pgk1***	phosphoglycerate kinase 1	0005975 // carbohydrate metabolic process // not recorded///0006094 // gluconeogenesis // not recorded///0006096 // glycolytic process // inferred from direct assay///0006096 //
NM_001040682///NM_053155///NR_104435///NR_			0.00592363	1.07	1.12	1.26	***Clmn***	calmin	0008285 // negative regulation of cell proliferation // inferred from direct assay///0031175 // neuron projection development // inferred from mutant phenotype
NM_001291439///NM	9.95E-04	4.98E-04		1.28	1.87	1.26	***Hmgcs1***	3-hydroxy-3-methylglutaryl-Coenzyme A synthase 1	0001889 // liver development // inferred from electronic annotation///0006629 // lipid metabolic process // inferred from electronic annotation///0006694 // steroid biosynthetic pr
NM_007386		0.006665054		1.12	1.29	1.26	***Aco1***	aconitase 1	0006099 // tricarboxylic acid cycle // inferred from electronic annotation///0006101 // citrate metabolic process // not recorded///0006417 // regulation of translation // inferred fro
NM_007633			0.0034679	1.08	1.19	1.26	***Ccne1***	cyclin E1	0000082 // G1/S transition of mitotic cell cycle // inferred from electronic annotation///0001547 // antral ovarian follicle growth // inferred from electronic annotation///0001889 //
NM_009569///XM_00	0.00714783			-1.21	1.06	1.26	***Zfpm1***	zinc finger protein, multitype 1	0000122 // negative regulation of transcription from RNA polymerase II promoter // inferred from direct assay///0000122 // negative regulation of transcription from RNA polymeras
NM_009773///XM_006498622			0.00485824	-1.00	1.21	1.26	***Bub1b***	budding uninhibited by benzimidazoles 1 homolog, beta (S. cerevis	0006468 // protein phosphorylation // inferred from electronic annotation///0006915 // apoptotic process // inferred from electronic annotation///0007049 // cell cycle // inferred f
NM_133225			0.00571514	1.01	1.04	1.26	***Acbd3***	acyl-Coenzyme A binding domain containing 3	0006629 // lipid metabolic process // inferred from electronic annotation///0006694 // steroid biosynthetic process // inferred from electronic annotation///0006810 // transport // i
NM_001033293///XR_374072///XR_374073///XR_3			0.00147232	1.04	1.19	1.26	***Uap1l1***	UDP-N-acteylglucosamine pyrophosphorylase 1-like 1	0006048 // UDP-N-acetylglucosamine biosynthetic process // ---///0008152 // metabolic process // inferred from electronic annotation
NM_001242368///NM_007972		0.007001603		1.10	1.21	1.26	***F10***	coagulation factor X	0006508 // proteolysis // inferred from electronic annotation///0007596 // blood coagulation // not recorded///0007599 // hemostasis // inferred from electronic annotation///003
NM_001252578///NM_001252579///NM_028072//			0.00578571	-1.08	1.17	1.25	***Sulf2***	sulfatase 2	0001822 // kidney development // inferred from genetic interaction///0002063 // chondrocyte development // inferred from mutant phenotype///0003094 // glomerular filtration //
NM_026862///XM_011250688///XM		6.07E-04		1.20	1.35	1.25	***Cd177***	CD177 antigen	
NM_001313923///NM	0.00142853			1.16	1.16	1.25	***Actg1***	actin, gamma, cytoplasmic 1	0001895 // retina homeostasis // inferred from electronic annotation///0045214 // sarcomere organization // inferred from direct assay///0051592 // response to calcium ion // infe
NM_007634///XM_006523554			0.00964926	1.02	1.08	1.25	***Ccnf***	cyclin F	0000320 // re-entry into mitotic cell cycle // inferred from mutant phenotype///0001890 // placenta development // inferred from mutant phenotype///0007049 // cell cycle // inferr
NM_021274			0.0097251	1.06	1.06	1.25	***Cxcl10***	chemokine (C-X-C motif) ligand 10	0002690 // positive regulation of leukocyte chemotaxis // not recorded///0006935 // chemotaxis // not recorded///0006954 // inflammatory response // not recorded///0006955 //
NM_001080129///NM_001080130///NM_0010801			0.00962911	-1.12	1.08	1.25	***Tmpo***	thymopoietin	0006355 // regulation of transcription, DNA-templated // inferred from direct assay
NM_001165939///NM	0.00789198	0.004358608		1.24	1.35	1.25	***Tshb***	thyroid stimulating hormone, beta subunit	0033189 // response to vitamin A // inferred from electronic annotation///0043627 // response to estrogen // inferred from electronic annotation///0051592 // response to calcium i
NM_026894///XM_006506552///XM		0.00584872		1.06	1.24	1.25	***Tamm41***	TAM41, mitochondrial translocator assembly and maintenance pr	0006629 // lipid metabolic process // inferred from electronic annotation///0008654 // phospholipid biosynthetic process // inferred from electronic annotation///0016024 // CDP-di
NM_026053///XM_011246581///XM		0.009550269	0.00996635	1.16	1.20	1.25	***Gemin6***	gem (nuclear organelle) associated protein 6	0000245 // spliceosomal complex assembly // inferred from electronic annotation///0000387 // spliceosomal snRNP assembly // not recorded///0006397 // mRNA processing // infe
NM_001306205///NM_023556///XM_006530183//			0.00911818	1.06	1.14	1.25	***Mvk***	mevalonate kinase	0006629 // lipid metabolic process // inferred from electronic annotation///0006694 // steroid biosynthetic process // inferred from electronic annotation///0006695 // cholesterol b
NM_009791///XM_006529108		0.006524405		1.03	1.25	1.25	***Aspm***	asp (abnormal spindle)-like, microcephaly associated (Drosophila)	0001764 // neuron migration // inferred from genetic interaction///0001764 // neuron migration // inferred from mutant phenotype///0002052 // positive regulation of neuroblast p
NM_019980///XM_006522427///XR_		0.005401038		1.16	1.29	1.25	***Litaf///Gm9861***	LPS-induced TN factor///predicted gene 9861	0001817 // regulation of cytokine production // inferred from mutant phenotype///0006351 // transcription, DNA-templated // inferred from electronic annotation///0006355 // reg
NM_001290706///NM_025452///NM		0.006466657		1.10	1.28	1.25	***Tmem54***	transmembrane protein 54	
NM_001026211///NM_001286972//		0.005629477		1.07	1.33	1.25	***Dnajc19///Dnajc19-ps***	DnaJ (Hsp40) homolog, subfamily C, member 19///DnaJ (Hsp40) h	0006810 // transport // inferred from electronic annotation///0007601 // visual perception // not recorded///0015031 // protein transport // inferred from electronic annotation///0
NM_021334///XR_378219			0.00897249	-1.04	1.10	1.25	***Itgax***	integrin alpha X	0007155 // cell adhesion // inferred from electronic annotation///0007229 // integrin-mediated signaling pathway // inferred from electronic annotation///0034113 // heterotypic ce
NM_001042534///NM_001271395//		3.17E-05		-1.04	1.55	1.24	***Capg***	capping protein (actin filament), gelsolin-like	0030031 // cell projection assembly // inferred from direct assay///0051016 // barbed-end actin filament capping // inferred from electronic annotation///0051693 // actin filament c
NM_139149///XM_006507759///XM_006507760			0.00616388	1.14	1.25	1.24	***Fus***	fused in sarcoma	0071277 // cellular response to calcium ion // not recorded///1903506 // regulation of nucleic acid-templated transcription // inferred from electronic annotation
NM_172845		0.00498705		1.15	1.28	1.24	***Adamts4***	a disintegrin-like and metallopeptidase (reprolysin type) with thron	0006508 // proteolysis // inferred from direct assay///0042742 // defense response to bacterium // inferred from genetic interaction
NM_001311101///NM_026467///XM_006511383			0.00202505	1.04	1.14	1.24	***Rps27l***	ribosomal protein S27-like	0000028 // ribosomal small subunit assembly // ---///0006281 // DNA repair // not recorded///0006412 // translation // not recorded///0006919 // activation of cysteine-type endop
NM_013470		0.007438398	0.00485435	1.06	1.25	1.24	***Anxa3***	annexin A3	0006909 // phagocytosis // not recorded///0010595 // positive regulation of endothelial cell migration // not recorded///0021766 // hippocampus development // inferred from elec
NM_025434			0.00607998	1.11	1.19	1.24	***Mrps28***	mitochondrial ribosomal protein S28	
NM_021526			0.00564625	1.08	1.19	1.24	***Psmd14***	proteasome (prosome, macropain) 26S subunit, non-ATPase, 14	0000724 // double-strand break repair via homologous recombination // not recorded///0006281 // DNA repair // inferred from electronic annotation///0006303 // double-strand br
NM_153576	0.00900769	9.15E-04		1.29	1.40	1.24	***Cxcl17***	chemokine (C-X-C motif) ligand 17	0001525 // angiogenesis // inferred from electronic annotation///0006935 // chemotaxis // inferred from electronic annotation///0007275 // multicellular organismal development /
NM_153570			0.00282539	1.12	1.22	1.24	***Noc4l***	nucleolar complex associated 4 homolog (S. cerevisiae)	0042254 // ribosome biogenesis // inferred from electronic annotation
NM_001310595///NM_001310596///NM_011184//			0.00255683	1.09	1.12	1.24	***Psma3***	proteasome (prosome, macropain) subunit, alpha type 3	0006508 // proteolysis // inferred from electronic annotation///0006511 // ubiquitin-dependent protein catabolic process // inferred from electronic annotation///0010499 // protea
NM_001164671///NM	0.00660579			1.45	-1.02	1.24	***Dnaja1***	DnaJ (Hsp40) homolog, subfamily A, member 1	0006457 // protein folding // inferred from electronic annotation///0007283 // spermatogenesis // inferred from mutant phenotype///0009408 // response to heat // inferred from e
NM_080457///NM_18	0.00167203	0.007025064		1.43	1.24	1.24	***Muc4***	mucin 4	0001953 // negative regulation of cell-matrix adhesion // not recorded///0002244 // hematopoietic progenitor cell differentiation // inferred from mutant phenotype///0002853 // ne
NM_001310640///NM	9.76E-04	0.003472649		1.43	1.95	1.24	***Pmvk***	phosphomevalonate kinase	0006629 // lipid metabolic process // inferred from electronic annotation///0006694 // steroid biosynthetic process // inferred from electronic annotation///0006695 // cholesterol b
NM_022331///XM_00	1.17E-04			1.29	1.11	1.24	***Herpud1***	homocysteine-inducible, endoplasmic reticulum stress-inducible, u	0006511 // ubiquitin-dependent protein catabolic process // inferred from direct assay///0006511 // ubiquitin-dependent protein catabolic process // inferred from mutant phenotyp
NM_001289428///NM	5.38E-04			-1.27	-1.00	1.24	***Pira1///Pira2///Pira6///Gm106***	paired-Ig-like receptor A1///paired-Ig-like receptor A2///paired-Ig-	0001782 // B cell homeostasis // inferred from mutant phenotype///0002250 // adaptive immune response // inferred from electronic annotation///0002376 // immune system proc
NM_001166589///NM_001166590//		0.002258092	0.00355889	1.10	1.26	1.24	***Eif5a***	eukaryotic translation initiation factor 5A	0006406 // mRNA export from nucleus // not recorded///0006412 // translation // inferred from electronic annotation///0006414 // translational elongation // inferred from electron
NM_001252658///NM	4.27E-04	0.007717057		1.24	1.40	1.24	***Ldlr***	low density lipoprotein receptor	0006629 // lipid metabolic process // inferred from genetic interaction///0006810 // transport // inferred from electronic annotation///0006869 // lipid transport // inferred from ele
NM_172733		0.005572854	0.00386057	1.11	1.25	1.23	***Dera***	2-deoxyribose-5-phosphate aldolase homolog (C. elegans)	0009264 // deoxyribonucleotide catabolic process // not recorded///0016052 // carbohydrate catabolic process // not recorded///0046121 // deoxyribonucleoside catabolic process
NM_010851	0.00178415	7.19E-04	0.00242389	1.29	1.29	1.23	***Myd88***	myeloid differentiation primary response gene 88	0002238 // response to molecule of fungal origin // inferred from mutant phenotype///0002376 // immune system process // inferred from electronic annotation///0002755 // MyD8
NM_001253768///NM_001253769//		0.006325577	0.00624043	1.00	1.23	1.23	***Ssx2ip***	synovial sarcoma, X breakpoint 2 interacting protein	0007155 // cell adhesion // inferred from electronic annotation///0030030 // cell projection organization // inferred from electronic annotation///0035020 // regulation of Rac protei
NM_173363///NM_178041///XM_006542837			0.00901469	1.11	1.12	1.23	***Eif5///LOC102641436***	eukaryotic translation initiation factor 5	0006412 // translation // inferred from electronic annotation///0006413 // translational initiation // inferred from electronic annotation///0006446 // regulation of translational initia
NM_001025388///NM_023119///XM		7.90E-04		1.04	1.48	1.23	***Eno1///Eno1b***	enolase 1, alpha non-neuron///enolase 1B, retrotransposed	0001701 // in utero embryonic development // inferred from mutant phenotype///0006096 // glycolytic process // inferred from sequence or structural similarity///0051099 // positi

RefSeq Transcript ID	p (24h n=460)	p (48h n=657)	p (72h n=805)	FC 24h	FC 48h	FC 72h	Gene Symbol	Gene Title	Gene Ontology Biological Process
NM_027352///NR_027343///XM_006500155			0.00214126	1.01	1.14	1.23	Gorasp2	golgi reassembly stacking protein 2	0006996 // organelle organization // not recorded///0007030 // Golgi organization // not recorded
NM_001025561///NM_001025562///NM_019700//			0.00438658	1.06	1.11	1.23	Pus1	pseudouridine synthase 1	0001522 // pseudouridine synthesis // inferred from electronic annotation///0008033 // tRNA processing // inferred from electronic annotation///0009451 // RNA modification // infe
NM_029397///NM_170598		0.006745453		1.19	1.47	1.23	Rbm12	RNA binding motif protein 12	0006351 // transcription, DNA-templated // inferred from electronic annotation///0006355 // regulation of transcription, DNA-templated // inferred from electronic annotation///000
NM_008360///XM_006510024///XM		0.00593709		1.08	1.27	1.23	Il18	interleukin 18	0000165 // MAPK cascade // not recorded///0001525 // angiogenesis // not recorded///0001525 // angiogenesis // inferred from sequence or structural similarity///0001666 // respo
NM_153547///NM_17	3.08E-04	0.004201335	0.00802193	1.40	1.31	1.23	Gnl3	guanine nucleotide binding protein-like 3 (nucleolar)	0008283 // cell proliferation // not recorded///0042127 // regulation of cell proliferation // not recorded///0042254 // ribosome biogenesis // not recorded///1902895 // positive reg
NM_023142		0.004574685	0.00490536	1.03	1.36	1.22	Arpc1b	actin related protein 2/3 complex, subunit 1B	0030833 // regulation of actin filament polymerization // inferred from electronic annotation///0034314 // Arp2/3 complex-mediated actin nucleation // not recorded
NM_013562	4.82E-04	0.006719883		1.31	1.25	1.22	Ifrd1	interferon-related developmental regulator 1	0007275 // multicellular organismal development // inferred from electronic annotation///0014706 // striated muscle tissue development // inferred from mutant phenotype///0030
NM_001045863///NM_001045864///NM_138580//			0.00221544	1.12	1.18	1.22	Nelfe	negative elongation factor complex member E, Rdbp	0000122 // negative regulation of transcription from RNA polymerase II promoter // inferred from electronic annotation///0006351 // transcription, DNA-templated // inferred from e
NM_009112///XM_011251865		0.002119359	0.00867136	1.00	1.31	1.22	LOC105242499///S100a10	protein S100-A10-like///S100 calcium binding protein A10 (calpact	0001765 // membrane raft assembly // not recorded///0006900 // membrane budding // not recorded///0043547 // positive regulation of GTPase activity // not recorded///005109
NM_133902///XM_011248202///XM_011248203			0.00987496	1.04	1.07	1.22	Sdsl	serine dehydratase-like	0006565 // L-serine catabolic process // not recorded///0006567 // threonine catabolic process // not recorded
NM_138584///XM_006511222		0.008324645		1.03	1.19	1.22	Spg21	spastic paraplegia 21 homolog (human)	
NM_025798///XM_006512823///XM		0.005594041	0.00595956	1.00	1.23	1.22	Hint3	histidine triad nucleotide binding protein 3	0008152 // metabolic process // inferred from electronic annotation
NM_001162946///NM	0.00282403			1.19	1.49	1.22	Pcx	pyruvate carboxylase	0006090 // pyruvate metabolic process // not recorded///0006094 // gluconeogenesis // not recorded///0006107 // oxaloacetate metabolic process // not recorded///0006629 // lip
NM_026030			0.00327023	1.12	1.14	1.22	Eif2s2	eukaryotic translation initiation factor 2, subunit 2 (beta)	0001701 // in utero embryonic development // inferred from mutant phenotype///0002176 // male germ cell proliferation // inferred from mutant phenotype///0006412 // translatio
NM_026753			0.00519836	-1.03	1.20	1.22	Fam96b	family with sequence similarity 96, member B	0007059 // chromosome segregation // not recorded///0016226 // iron-sulfur cluster assembly // not recorded
NM_009137	0.00115586	0.00277881	0.00683134	1.31	1.24	1.22	Ccl22	chemokine (C-C motif) ligand 22	0002548 // monocyte chemotaxis // ---///0006935 // chemotaxis // inferred from electronic annotation///0006954 // inflammatory response // inferred from electronic annotation//
NM_001164567///NM_011700///XM		0.009423475		1.17	1.25	1.22	Vill	villin-like	0007010 // cytoskeleton organization // inferred from electronic annotation///0051693 // actin filament capping // inferred from electronic annotation
NM_028315///XM_006519601///XM_006519602//			0.00812882	1.13	1.16	1.22	Dis3	DIS3 mitotic control homolog (S. cerevisiae)	0006364 // rRNA processing // not recorded///0006402 // mRNA catabolic process // not recorded///0016075 // rRNA catabolic process // not recorded///0043547 // positive regula
NM_025301		0.008490994	0.00326601	1.05	1.28	1.22	Mrpl17	mitochondrial ribosomal protein L17	0000002 // mitochondrial genome maintenance // traceable author statement///0006412 // translation // not recorded
NM_001304719///NM_009725///XM		0.008715484		1.03	1.27	1.22	Atp5f1	ATP synthase, H+ transporting, mitochondrial F0 complex, subunit	0006810 // transport // inferred from electronic annotation///0006811 // ion transport // inferred from electronic annotation///0015986 // ATP synthesis coupled proton transport /
NM_007984			0.00769135	-1.04	1.05	1.22	Fscn1	fascin homolog 1, actin bundling protein (Strongylocentrotus purpu	0007015 // actin filament organization // inferred from electronic annotation///0007043 // cell-cell junction assembly // not recorded///0010592 // positive regulation of lamellipodiu
NM_033079///XM_006505368///XM_006505369//			0.00829851	1.03	1.09	1.22	M1ap	meiosis 1 associated protein	0006396 // RNA processing // non-traceable author statement///0007283 // spermatogenesis // inferred from mutant phenotype///0007292 // female gamete generation // inferred
NM_001025388///NM_023119///XM		0.001249618		1.04	1.48	1.22	Eno1///Eno1b	enolase 1, alpha non-neuron///enolase 1B, retrotransposed	0001701 // in utero embryonic development // inferred from mutant phenotype///0006096 // glycolytic process // inferred from sequence or structural similarity///0051099 // positi
NM_010235		0.007389488		1.16	1.24	1.22	Fosl1	fos-like antigen 1	0001701 // in utero embryonic development // inferred from mutant phenotype///0006355 // regulation of transcription, DNA-templated // inferred from electronic annotation///00
NM_026159			0.0086093	1.04	1.24	1.21	Retsat	retinol saturase (all trans retinol 13,14 reductase)	0042572 // retinol metabolic process // inferred from direct assay///0055114 // oxidation-reduction process // inferred from direct assay
NM_011333	0.00835736	4.44E-04		1.20	1.36	1.21	Ccl2	chemokine (C-C motif) ligand 2	0000165 // MAPK cascade // inferred from electronic annotation///0001525 // angiogenesis // traceable author statement///0001666 // response to hypoxia // inferred from electro
NM_022654///XM_006536218///XM_006536219//			0.00499119	1.08	-1.01	1.21	Pidd1	p53 induced death domain protein 1	0006915 // apoptotic process // inferred from electronic annotation///0006919 // activation of cysteine-type endopeptidase activity involved in apoptotic process // inferred from ge
NM_025595		0.001052343	0.00818493	1.13	1.32	1.21	Mrpl51	mitochondrial ribosomal protein L51	0006412 // translation // inferred from direct assay///0032543 // mitochondrial translation // inferred from sequence or structural similarity
NM_001310723///NM_001310725///NM_0013107			0.00357863	1.05	1.12	1.21	Setd8	SET domain containing (lysine methyltransferase) 8	0000122 // negative regulation of transcription from RNA polymerase II promoter // not recorded///0006351 // transcription, DNA-templated // inferred from electronic annotation/
NM_026129		0.009909589	0.00872861	1.08	1.19	1.21	Erp29	endoplasmic reticulum protein 29	0000187 // activation of MAPK activity // not recorded///0001934 // positive regulation of protein phosphorylation // not recorded///0006457 // protein folding // not recorded///00
NM_009338///NM_153151		0.004299975		1.14	1.26	1.21	Acat2///Acat3	acetyl-Coenzyme A acetyltransferase 2///acetyl-Coenzyme A acety	0008152 // metabolic process // inferred from electronic annotation///0045797 // positive regulation of intestinal cholesterol absorption // inferred from direct assay
NM_009253///XM_00	0.00116102	3.71E-05		1.28	1.78	1.21	Serpina3m	serine (or cysteine) peptidase inhibitor, clade A, member 3M	0010466 // negative regulation of peptidase activity // inferred from electronic annotation///0010951 // negative regulation of endopeptidase activity // not recorded///0034097 // r
NM_001310618///NM_001310619//		0.009811147		1.02	1.19	1.21	Ube2k	ubiquitin-conjugating enzyme E2K	0000209 // protein polyubiquitination // not recorded///0008152 // metabolic process // inferred from electronic annotation///0010800 // positive regulation of peptidyl-threonine p
NM_001172100///NM_001172101//		3.73E-04		1.09	1.36	1.21	Rnh1	ribonuclease/angiogenin inhibitor 1	0043086 // negative regulation of catalytic activity // not recorded///0045765 // regulation of angiogenesis // not recorded
NM_001038703///NM_030258///XM		0.008231633		1.01	1.23	1.20	Gpr146	G protein-coupled receptor 146	0007165 // signal transduction // inferred from electronic annotation///0007186 // G-protein coupled receptor signaling pathway // inferred from electronic annotation
NM_016966///NR_033590///NR_07		0.006945512	0.00353562	-1.01	1.23	1.20	Gm8096///Phgdh///Gm6756	3-phosphoglycerate dehydrogenase pseudogene///3-phosphoglyc	0006541 // glutamine metabolic process // inferred from mutant phenotype///0006544 // glycine metabolic process // inferred from mutant phenotype///0006563 // L-serine metab
NM_009338///NM_15	0.00645456	4.86E-04	0.00965004	1.17	1.36	1.20	Acat2///Acat3	acetyl-Coenzyme A acetyltransferase 2///acetyl-Coenzyme A acety	0008152 // metabolic process // inferred from electronic annotation///0045797 // positive regulation of intestinal cholesterol absorption // inferred from direct assay
NM_001277256///NM	0.00650293			1.29	1.26	1.20	Spred1	sprouty protein with EVH-1 domain 1, related sequence	0000188 // inactivation of MAPK activity // inferred from mutant phenotype///0007275 // multicellular organismal development // inferred from electronic annotation///0009966 //
NM_053074			0.00948157	1.07	1.23	1.20	Nup62	nucleoporin 62	0006351 // transcription, DNA-templated // not recorded///0006606 // protein import into nucleus // not recorded///0006810 // transport // inferred from electronic annotation///0
NM_133888			0.00918714	1.14	1.19	1.20	Smpdl3b	sphingomyelin phosphodiesterase, acid-like 3B	0006685 // sphingomyelin catabolic process // inferred from electronic annotation///0008152 // metabolic process // inferred from electronic annotation
NM_029271		0.003213113		1.08	1.24	1.20	Mrpl32	mitochondrial ribosomal protein L32	0006412 // translation // not recorded
NM_011132		0.008494997		1.08	1.19	1.20	Pole	polymerase (DNA directed), epsilon	0000082 // G1/S transition of mitotic cell cycle // not recorded///0000731 // DNA synthesis involved in DNA repair // not recorded///0001701 // in utero embryonic development // i
NM_001081274///NM_025801///XM		0.006841025	0.00577521	1.10	1.38	1.20	Pgd	phosphogluconate dehydrogenase	0005975 // carbohydrate metabolic process // not recorded///0006098 // pentose-phosphate shunt // not recorded///0006739 // NADP metabolic process // not recorded///000905
NM_013899	0.0028944	0.002443797	0.00938679	1.29	1.29	1.19	Timm10	translocase of inner mitochondrial membrane 10	0006810 // transport // inferred from electronic annotation///0015031 // protein transport // inferred from electronic annotation///0045039 // protein import into mitochondrial inn
NM_001253751///NM	5.24E-04	0.002388096		1.29	1.45	1.19	Fdps	farnesyl diphosphate synthetase	0006629 // lipid metabolic process // inferred from electronic annotation///0006694 // steroid biosynthetic process // inferred from electronic annotation///0006695 // cholesterol b
NM_008160		0.005537277		1.06	1.42	1.19	Gpx1	glutathione peroxidase 1	0000302 // response to reactive oxygen species // inferred from mutant phenotype///0001659 // temperature homeostasis // inferred from genetic interaction///0001885 // endothe
NM_026033			0.00872779	-1.08	1.05	1.19	Gatad1	GATA zinc finger domain containing 1	0006355 // regulation of transcription, DNA-templated // inferred from electronic annotation
NM_001083904///NM	0.00806166	0.003050153		1.17	1.27	1.19	Fetub	fetuin beta	0007338 // single fertilization // inferred from mutant phenotype///0007339 // binding of sperm to zona pellucida // inferred from mutant phenotype///0010466 // negative regulatio
NM_009076///XR_880074			0.00623787	-1.01	1.15	1.19	LOC102635048///Rpl12///Rpl12-	60S ribosomal protein L12 pseudogene///ribosomal protein L12///	0000027 // ribosomal large subunit assembly // ---///0006412 // translation // inferred from electronic annotation
NM_001039701///NM_001159562//		0.001424126		1.03	1.30	1.19	Il1rn	interleukin 1 receptor antagonist	0001660 // fever generation // not recorded///0001960 // negative regulation of cytokine-mediated signaling pathway // not recorded///0002437 // inflammatory response to antige
NM_001159421///NM_001159422//		0.001059736		1.11	1.25	1.19	Ccdc58	coiled-coil domain containing 58	
NM_001289591///NM	3.07E-04			1.30	1.25	1.19	Tmem173	transmembrane protein 173	0002218 // activation of innate immune response // inferred from mutant phenotype///0002218 // activation of innate immune response // not recorded///0002230 // positive regul
NM_133653///XM_00	1.59E-04			1.30	1.19	1.19	Mat1a	methionine adenosyltransferase I, alpha	0006556 // S-adenosylmethionine biosynthetic process // not recorded///0006730 // one-carbon metabolic process // inferred from electronic annotation///0051260 // protein hom
NM_001290810///NM_023536///XM_006539162			0.00914696	1.14	1.12	1.19	Mrto4	mRNA turnover 4, homolog (S. cerevisiae)	0042254 // ribosome biogenesis // inferred from electronic annotation
NM_009533///XM_00	0.00776811			1.21	1.21	1.19	Xrcc5	X-ray repair complementing defective repair in Chinese hamster ce	0000237 // leptotene // inferred from mutant phenotype///0000723 // telomere maintenance // not recorded///0006281 // DNA repair // inferred from electronic annotation///000
NM_011113///XM_00	4.64E-04		0.00585921	1.24	1.60	1.19	Plaur	plasminogen activator, urokinase receptor	0001934 // positive regulation of protein phosphorylation // not recorded///0010628 // positive regulation of gene expression // not recorded///0016310 // phosphorylation // inferr
NM_001111058///NM_021293///XM_006540590//			0.00515563	1.03	1.13	1.19	Cd33	CD33 antigen	0007155 // cell adhesion // inferred from electronic annotation
NM_001111099///NM	3.52E-04			1.46	1.18	1.18	Cdkn1a	cyclin-dependent kinase inhibitor 1A (P21)	0000079 // regulation of cyclin-dependent protein serine/threonine kinase activity // inferred from mutant phenotype///0000082 // G1/S transition of mitotic cell cycle // not recorde
NM_026578///XM_01	0.00860914			1.16	1.13	1.18	Gar1	GAR1 ribonucleoprotein homolog (yeast)	0001522 // pseudouridine synthesis // inferred from electronic annotation///0006364 // rRNA processing // inferred from electronic annotation///0042254 // ribosome biogenesis //
NM_007422///XM_006496623		0.00722469		1.11	1.23	1.18	Adss	adenylosuccinate synthetase, non muscle	0006164 // purine nucleotide biosynthetic process // inferred from direct assay///0006167 // AMP biosynthetic process // inferred from direct assay///0006167 // AMP biosynthetic p
NM_001172146///NM	0.00967711			1.14	1.29	1.18	Aimp2	aminoacyl tRNA synthetase complex-interacting multifunctional pr	0006412 // translation // inferred from electronic annotation///0006915 // apoptotic process // inferred from electronic annotation///0007275 // multicellular organismal developme
NM_025878///XM_00	0.00136003	0.00710923		1.24	1.23	1.18	Mrps18b	mitochondrial ribosomal protein S18B	0006412 // translation // inferred from electronic annotation///0032543 // mitochondrial translation // inferred from sequence or structural similarity
NM_001166672///NM	6.62E-04			-1.30	-1.07	1.18	Gm10693///Gm14548///Lilra6//	predicted pseudogene 10693///predicted gene 14548///leukocyte	0001782 // B cell homeostasis // inferred from mutant phenotype///0002250 // adaptive immune response // inferred from electronic annotation///0002376 // immune system proc
NM_011331///XM_01	0.00829025			1.24	1.18	1.18	Ccl12///LOC105242482	chemokine (C-C motif) ligand 12///C-C motif chemokine 12	0000165 // MAPK cascade // not recorded///0001525 // angiogenesis // inferred from direct assay///0002548 // monocyte chemotaxis // inferred from direct assay///0002548 // mo
NM_009174	0.00268195	0.006479654		1.28	1.28	1.18	Siah2	seven in absentia 2	0006511 // ubiquitin-dependent protein catabolic process // inferred from electronic annotation///0006915 // apoptotic process // not recorded///0007049 // cell cycle // inferred fr
NM_008446///XM_006527855///XM		0.008590994		1.03	1.19	1.18	Kif4	kinesin family member 4	0000281 // mitotic cytokinesis // not recorded///0007018 // microtubule-based movement // not recorded///0008152 // metabolic process // inferred from electronic annotation///
NM_027198		0.009409183		1.11	1.19	1.18	Zswim7	zinc finger SWIM-type containing 7	0000724 // double-strand break repair via homologous recombination // not recorded///0006281 // DNA repair // inferred from electronic annotation///0006310 // DNA recombinat
NM_023058///XM_006524310///XM_006524311			0.00785748	-1.01	1.05	1.18	Pkmyt1	protein kinase, membrane associated tyrosine/threonine 1	0006468 // protein phosphorylation // inferred from electronic annotation///0007049 // cell cycle // inferred from electronic annotation///0010923 // negative regulation of phospha
NM_178614			0.00855916	1.06	1.30	1.18	Samm50	sorting and assembly machinery component 50 homolog (S. cerevi	0033108 // mitochondrial respiratory chain complex assembly // not recorded///0042407 // cristae formation // not recorded///0045040 // protein import into mitochondrial outer
NM_001253883///NM_011099///XM		0.001558087		1.07	1.36	1.18	Pkm	pyruvate kinase, muscle	0001889 // liver development // not recorded///0006006 // glucose metabolic process // not recorded///0006096 // glycolytic process // inferred from direct assay///0006096 // glyc
NM_001127177///NM	0.00354967	0.002393525		1.28	1.33	1.17	Ptpn2	protein tyrosine phosphatase, non-receptor type 2	0006470 // protein dephosphorylation // inferred from mutant phenotype///0008285 // negative regulation of cell proliferation // not recorded///0008286 // insulin receptor signalin
NM_007695///XM_00	0.00300145			1.27	1.11	1.17	Chil1	chitinase-like 1	0005975 // carbohydrate metabolic process // inferred from electronic annotation///0006032 // chitin catabolic process // inferred from electronic annotation///0006915 // apoptot
NM_008518	0.00122756			-1.27	-1.01	1.17	Ltb	lymphotoxin B	0006955 // immune response // inferred from electronic annotation///0010467 // gene expression // inferred from mutant phenotype///0043588 // skin development // inferred fro
NM_009388	0.00995543	0.005821056		1.15	1.39	1.17	Tkt	transketolase	0006098 // pentose-phosphate shunt // not recorded///0008152 // metabolic process // inferred from electronic annotation///0009052 // pentose-phosphate shunt, non-oxidative b
NM_025305		0.004830288		1.13	1.20	1.17	Mrps7	mitchondrial ribosomal protein S7	0000028 // ribosomal small subunit assembly // ---///0006412 // translation // not recorded///0032543 // mitochondrial translation // inferred from sequence or structural similarity
NM_016859	0.00461352			1.18	1.15	1.17	Bysl	bystin-like	0000462 // maturation of SSU-rRNA from tricistronic rRNA transcript (SSU-rRNA, 5.8S rRNA, LSU-rRNA) // inferred from mutant phenotype///0001701 // in utero embryonic developn
NM_027168///XM_00	0.0064674	0.005051289		1.18	1.20	1.17	Hddc2	HD domain containing 2	
NM_001289726///NM_008084///XM		5.92E-05		1.09	1.47	1.17	Gapdh///3000002C10Rik///Gap	glyceraldehyde-3-phosphate dehydrogenase///glyceraldehyde-3-p	0000226 // microtubule cytoskeleton organization // not recorded///0005975 // carbohydrate metabolic process // not recorded///0006006 // glucose metabolic process // inferred
NM_016776	0.00291894		0.00724114	1.18	1.16	1.17	Mybbp1a	MYB binding protein (P160) 1a	0001649 // osteoblast differentiation // not recorded///0006351 // transcription, DNA-templated // inferred from electronic annotation///0006355 // regulation of transcription, DNA
NM_001199060///NM	0.00460543	0.002391093		1.19	1.28	1.17	Wdr12	WD repeat domain 12	0000463 // maturation of LSU-rRNA from tricistronic rRNA transcript (SSU-rRNA, 5.8S rRNA, LSU-rRNA) // not recorded///0000466 // maturation of 5.8S rRNA from tricistronic rRNA t
NM_025606		0.001050849		1.15	1.36	1.17	Mrpl16	mitochondrial ribosomal protein L16	0006412 // translation // not recorded///0032543 // mitochondrial translation // not recorded
NM_013820	0.00660609			1.20	1.21	1.17	Hk2	hexokinase 2	0001678 // cellular glucose homeostasis // not recorded///0002931 // response to ischemia // not recorded///0005975 // carbohydrate metabolic process // inferred from electronic
NM_053108///XM_00	3.42E-04			1.33	1.36	1.17	Glrx	glutaredoxin	0006810 // transport // inferred from electronic annotation///0032024 // positive regulation of insulin secretion // not recorded///0045454 // cell redox homeostasis // inferred from
NM_030210		0.006290488		1.10	1.28	1.17	Aacs	acetoacetyl-CoA synthetase	0001889 // liver development // not recorded///0006629 // lipid metabolic process // inferred from electronic annotation///0006631 // fatty acid metabolic process // inferred from
NM_026454///XM_00	0.00903872	0.005571217		1.17	1.22	1.17	Ube2f	ubiquitin-conjugating enzyme E2F (putative)	0016567 // protein ubiquitination // ---///0045116 // protein neddylation // inferred from mutant phenotype///0045116 // protein neddylation // not recorded
NM_010139///XM_00	4.63E-04	0.006126615		1.26	1.41	1.17	Epha2	Eph receptor A2	0001501 // skeletal system development // inferred from mutant phenotype///0001525 // angiogenesis // inferred from electronic annotation///0001568 // blood vessel developmer

RefSeq Transcript ID	p (24h n=460)	p (48h n=657)	p (72h n=805)	FC 24h	FC 48h	FC 72h	Gene Symbol	Gene Title	Gene Ontology Biological Process
NM_001098271///NM	0.00154031	0.007274224	0.00931412	1.18	1.24	1.17	Tmem176a	transmembrane protein 176A	2001199 // negative regulation of dendritic cell differentiation // inferred from mutant phenotype
NM_026305///XR_865041			0.00785083	1.02	1.25	1.17	Tceb2///Gm9238	transcription elongation factor B (SIII), polypeptide 2///transcriptio	0006351 // transcription, DNA-templated // inferred from electronic annotation///0006355 // regulation of transcription, DNA-templated // inferred from electronic annotation///000
NM_145832///NM_211358		0.001707742		1.11	1.23	1.16	Slc35c1	solute carrier family 35, member C1	0002230 // positive regulation of defense response to virus by host // not recorded///0006810 // transport // inferred from electronic annotation///0008643 // carbohydrate transpo
NM_001289726///NM_008084///NR		7.18E-04		1.11	1.42	1.16	3000002C10Rik///Gapdh///Gapı	glyceraldehyde-3-phosphate dehydrogenase pseudogene///glycera	0000226 // microtubule cytoskeleton organization // not recorded///0005975 // carbohydrate metabolic process // not recorded///0006006 // glucose metabolic process // inferred
NM_007498	0.00342608			1.20	1.34	1.16	Atf3	activating transcription factor 3	0000122 // negative regulation of transcription from RNA polymerase II promoter // inferred from direct assay///0000122 // negative regulation of transcription from RNA polymeras
NM_001289726///NM_008084///NR		8.65E-04		1.11	1.42	1.16	3000002C10Rik///Gapdh///Gapı	glyceraldehyde-3-phosphate dehydrogenase pseudogene///glycera	0000226 // microtubule cytoskeleton organization // not recorded///0005975 // carbohydrate metabolic process // not recorded///0006006 // glucose metabolic process // inferred
NM_023243///XM_006517324///XM		0.004300398		1.05	1.24	1.16	Ccnh	cyclin H	0000079 // regulation of cyclin-dependent protein serine/threonine kinase activity // inferred from electronic annotation///0006351 // transcription, DNA-templated // inferred from
NM_008894///XM_006514587		0.009997986		1.12	1.31	1.16	Pold2	polymerase (DNA directed), delta 2, regulatory subunit	0006260 // DNA replication // not recorded///0006261 // DNA-dependent DNA replication // not recorded///0071897 // DNA biosynthetic process // not recorded
NM_018780			0.00971082	1.04	1.05	1.16	Sfrp5	secreted frizzled-related sequence protein 5	0007275 // multicellular organismal development // inferred from electronic annotation///0008285 // negative regulation of cell proliferation // not recorded///0016055 // Wnt signa
NM_001081549///NM	0.00474643			1.18	1.25	1.16	Rcan1	regulator of calcineurin 1	0002931 // response to ischemia // inferred from mutant phenotype///0006950 // response to stress // inferred from genetic interaction///0006950 // response to stress // inferred f
NM_146006///XM_006513283///XM		0.005604337		1.09	1.27	1.15	Lss	lanosterol synthase	0006629 // lipid metabolic process // inferred from electronic annotation///0006694 // steroid biosynthetic process // inferred from electronic annotation///0006695 // cholesterol b
NM_007451		0.00270058		1.07	1.32	1.15	Slc25a5	solute carrier family 25 (mitochondrial carrier, adenine nucleotide	0006810 // transport // inferred from electronic annotation///0007059 // chromosome segregation // inferred from electronic annotation///0008284 // positive regulation of cell pro
NM_026396	0.00830519			1.27	1.13	1.15	Brix1	BRX1, biogenesis of ribosomes, homolog (S. cerevisiae)	0000027 // ribosomal large subunit assembly // not recorded///0042254 // ribosome biogenesis // inferred from electronic annotation
NM_001164604///NM_026746///XM		0.002682524		1.08	1.27	1.15	Nsmce2	non-SMC element 2 homolog (MMS21, S. cerevisiae)	0000722 // telomere maintenance via recombination // not recorded///0000724 // double-strand break repair via homologous recombination // not recorded///0006281 // DNA rep
NM_010118///XM_00	0.00419909	0.002001323		1.25	1.22	1.15	Egr2	early growth response 2	0006351 // transcription, DNA-templated // inferred from electronic annotation///0006355 // regulation of transcription, DNA-templated // inferred from mutant phenotype///00063
NM_007539	8.90E-04			1.27	1.13	1.15	Bdkrb1	bradykinin receptor, beta 1	0001933 // negative regulation of protein phosphorylation // not recorded///0002687 // positive regulation of leukocyte migration // not recorded///0006950 // response to stress //
NM_024478		0.003792772		1.01	1.24	1.15	Grpel1	GrpE-like 1, mitochondrial	0006457 // protein folding // inferred from electronic annotation///0050790 // regulation of catalytic activity // inferred from electronic annotation
NM_001130416///NM_010413///XM		0.008077991		1.20	1.23	1.14	Hdac6	histone deacetylase 6	0000209 // protein polyubiquitination // inferred from direct assay///0006351 // transcription, DNA-templated // inferred from electronic annotation///0006355 // regulation of trans
NM_025531///XM_00	0.00616997	0.009307507		1.20	1.22	1.14	Slmo2	slowmo homolog 2 (Drosophila)	0015914 // phospholipid transport // not recorded
NM_001159738///NM	3.46E-04	0.001178051		1.37	1.28	1.14	Ccl20	chemokine (C-C motif) ligand 20	0002548 // monocyte chemotaxis // ---///0006935 // chemotaxis // not recorded///0006954 // inflammatory response // inferred from electronic annotation///0006955 // immune r
NM_029571	0.00164614			1.21	1.22	1.14	Kti12	KTI12 homolog, chromatin associated (S. cerevisiae)	
NM_001008700///NM_010557///XM		0.004140298		1.13	1.24	1.14	Il4ra	interleukin 4 receptor, alpha	0002376 // immune system process // inferred from electronic annotation///0002532 // production of molecular mediator involved in inflammatory response // inferred from electro
NM_009443///NM_0C	8.16E-04			1.21	1.12	1.13	Tgoln1///Tgoln2	trans-golgi network protein///trans-golgi network protein 2	0006895 // Golgi to endosome transport // not recorded
NM_181470///XM_00	0.0019388			1.23	1.17	1.13	Ltv1	LTV1 homolog (S. cerevisiae)	
NM_001287180///NM	2.44E-04	0.004517567		1.37	1.28	1.13	Atf4	activating transcription factor 4	0006094 // gluconeogenesis // inferred from direct assay///0006351 // transcription, DNA-templated // inferred from electronic annotation///0006355 // regulation of transcription,
NM_001012766///NM	0.00113065			-1.32	1.44	1.13	Ear-ps2///Ear1///Ear12///Ear2/,	eosinophil-associated, ribonuclease A family, pseudogene 2///eosi	0006935 // chemotaxis // inferred from direct assay///0008152 // metabolic process // inferred from electronic annotation///0090305 // nucleic acid phosphodiester bond hydrolysis
NM_153419///XM_00	0.00569031			1.16	1.18	1.13	Grwd1	glutamate-rich WD repeat containing 1	
NM_001033711///NM	0.00830332			-1.25	1.07	1.12	Evi2a	ecotropic viral integration site 2a	
NM_010792///XM_00	0.00430018			1.28	1.10	1.12	Mettl1	methyltransferase like 1	0006400 // tRNA modification // not recorded///0008033 // tRNA processing // inferred from electronic annotation///0030488 // tRNA methylation // not recorded///0032259 // me
NM_001290984///NM	0.00245039			-1.19	-1.03	1.12	Asnsd1	asparagine synthetase domain containing 1	0006529 // asparagine biosynthetic process // not recorded///0006541 // glutamine metabolic process // not recorded///0008152 // metabolic process // inferred from electronic an
NM_179203///XM_00	0.00281832			1.21	1.16	1.11	Atad3a	ATPase family, AAA domain containing 3A	0016049 // cell growth // not recorded///0043066 // negative regulation of apoptotic process // not recorded
NM_008617		0.00869331		1.02	1.22	1.10	Mdh2	malate dehydrogenase 2, NAD (mitochondrial)	0005975 // carbohydrate metabolic process // inferred from electronic annotation///0006099 // tricarboxylic acid cycle // not recorded///0006107 // oxaloacetate metabolic process
NM_008303		0.002897397		-1.13	-1.46	1.10	Hspe1	heat shock protein 1 (chaperonin 10)	0001649 // osteoblast differentiation // not recorded///0006457 // protein folding // inferred from electronic annotation
NM_001163704///NM_001163705//		0.007315361		1.02	1.20	1.10	Fbxo6	F-box protein 6	0006281 // DNA repair // inferred from electronic annotation///0006516 // glycoprotein catabolic process // inferred from direct assay///0006974 // cellular response to DNA damag
NM_013599///XM_00	0.00221447			-1.22	-1.06	1.10	Mmp9	matrix metallopeptidase 9	0001501 // skeletal system development // inferred from genetic interaction///0001501 // skeletal system development // inferred from mutant phenotype///0001503 // ossification
NM_054098	1.70E-04			1.32	1.13	1.10	Steap4	STEAP family member 4	0006810 // transport // inferred from electronic annotation///0006811 // ion transport // inferred from electronic annotation///0015677 // copper ion import // inferred from direct
NM_001164359///NM	0.0034719			1.20	1.02	1.10	Erlin1	ER lipid raft associated 1	0006629 // lipid metabolic process // inferred from electronic annotation///0008202 // steroid metabolic process // inferred from electronic annotation///0008203 // cholesterol me
NM_025593///NM_02	0.00670775			1.20	1.25	1.10	Polr2l///Usp50	polymerase (RNA) II (DNA directed) polypeptide L///ubiquitin speci	0006351 // transcription, DNA-templated // inferred from electronic annotation///0006355 // regulation of transcription, DNA-templated // not recorded///0006360 // transcription f
NM_133955	0.00424521	0.004243274		1.31	1.25	1.10	Rhou	ras homolog gene family, member U	0000082 // G1/S transition of mitotic cell cycle // inferred from direct assay///0007010 // cytoskeleton organization // not recorded///0007264 // small GTPase mediated signal trans
NM_145632///XM_00	0.00224016			1.18	1.14	1.10	Polr2h	polymerase (RNA) II (DNA directed) polypeptide H	0006351 // transcription, DNA-templated // inferred from electronic annotation///0006360 // transcription from RNA polymerase I promoter // ---///0006366 // transcription from RI
NM_054045///NM_013548///NM_0:		0.007545842		-1.24	-1.32	1.09	Hist2h3c2///Hist1h3a///Hist1h3i	histone cluster 2, H3c2///histone cluster 1, H3a///histone cluster 1	0000183 // chromatin silencing at rDNA // traceable author statement///0002230 // positive regulation of defense response to virus by host // not recorded///0006334 // nucleosom
NM_025402		0.007948481		1.02	1.19	1.09	Ogfod3	2-oxoglutarate and iron-dependent oxygenase domain containing :	0055114 // oxidation-reduction process // inferred from electronic annotation
NM_016863///XM_006514974		0.006807548		-1.02	1.25	1.09	Fkbp1b	FK506 binding protein 1b	0000413 // protein peptidyl-prolyl isomerization // not recorded///0002027 // regulation of heart rate // inferred from mutant phenotype///0006457 // protein folding // inferred fro
NM_007679	3.66E-05			1.45	1.23	1.09	Cebpd	CCAAT/enhancer binding protein (C/EBP), delta	0002244 // hematopoietic progenitor cell differentiation // inferred from genetic interaction///0006351 // transcription, DNA-templated // inferred from electronic annotation///000
NM_001033305		0.009883953		1.01	1.32	1.09	Ndufb6	NADH dehydrogenase (ubiquinone) 1 beta subcomplex, 6	0006810 // transport // inferred from electronic annotation///0055114 // oxidation-reduction process // inferred from electronic annotation
NM_001083912///NM	0.00865958			1.15	1.16	1.09	Plekhg2	pleckstrin homology domain containing, family G (with RhoGef dor	0035023 // regulation of Rho protein signal transduction // inferred from electronic annotation///0043547 // positive regulation of GTPase activity // inferred from electronic annotat
NM_001304956///NM_008690///XM		0.005772485		1.01	1.20	1.09	Nfkbie	nuclear factor of kappa light polypeptide gene enhancer in B cells ii	0042942 // D-serine transport // not recorded///0045892 // negative regulation of transcription, DNA-templated // inferred from reviewed computational analysis
NM_008215///XM_01	0.00508407			1.17	1.16	1.08	Has1	hyaluronan synthase1	0007155 // cell adhesion // not recorded///0010764 // negative regulation of fibroblast migration // not recorded///0030213 // hyaluronan biosynthetic process // inferred from dire
NM_021362///XM_00	0.00476557			1.24	1.17	1.08	Pappa	pregnancy-associated plasma protein A	0006508 // proteolysis // inferred from electronic annotation///0030154 // cell differentiation // inferred from electronic annotation///0051384 // response to glucocorticoid // not r
NM_007408///XM_00	0.00409489			1.25	1.33	1.08	Plin2	perilipin 2	0014070 // response to organic cyclic compound // inferred from electronic annotation///0015909 // long-chain fatty acid transport // inferred from direct assay///0019915 // lipid st
NM_022886///XM_00	0.00591289	0.003103077		1.46	1.71	1.08	Scel	sciellin	0008544 // epidermis development // inferred from direct assay///0009790 // embryo development // inferred from direct assay///0030216 // keratinocyte differentiation // not rec
NM_008255///XM_006517530///XM		0.005009947		1.10	1.22	1.08	Hmgcr	3-hydroxy-3-methylglutaryl-Coenzyme A reductase	0006629 // lipid metabolic process // inferred from electronic annotation///0006694 // steroid biosynthetic process // inferred from electronic annotation///0006695 // cholesterol b
NM_001033499///NM_012009		0.008940532		1.01	1.18	1.07	Sh2d1b1///Sh2d1b2	SH2 domain containing 1B1///SH2 domain containing 1B2	0002366 // leukocyte activation involved in immune response // inferred from direct assay///0002717 // positive regulation of natural killer cell mediated immunity // inferred from m
NM_001301374///NM	4.38E-04			-1.38	1.08	1.07	Coro1a	coronin, actin binding protein 1A	0001845 // phagolysosome assembly // not recorded///0006816 // calcium ion transport // inferred from mutant phenotype///0006909 // phagocytosis // not recorded///0007015 /
NM_011528		0.008177246		1.09	1.37	1.07	Taldo1	transaldolase 1	0005975 // carbohydrate metabolic process // inferred from electronic annotation///0006002 // fructose 6-phosphate metabolic process // not recorded///0006098 // pentose-phos
NM_007894///NM_0C	0.00146783			-1.28	1.41	1.06	Ear1///Ear-ps2///Ear12///Ear2/,	eosinophil-associated, ribonuclease A family, member 1///eosinop	0008152 // metabolic process // inferred from electronic annotation///0090305 // nucleic acid phosphodiester bond hydrolysis // ---///0090305 // nucleic acid phosphodiester bond h
NM_007672///XM_01	6.07E-04			1.26	1.03	1.06	Cdr2	cerebellar degeneration-related 2	
NM_001278226///NM	0.00912827			-1.11	-1.07	1.06	Dmc1	DMC1 dosage suppressor of mck1 homolog, meiosis-specific homc	0000730 // DNA recombinase assembly // not recorded///0001541 // ovarian follicle development // inferred from mutant phenotype///0001556 // oocyte maturation // inferred fro
NM_053169///XM_006534580		0.002140378		1.13	1.31	1.06	Trim16	tripartite motif-containing 16	0043966 // histone H3 acetylation // not recorded///0043967 // histone H4 acetylation // not recorded///0045618 // positive regulation of keratinocyte differentiation // not recorde
NM_001039175///NM_001039176//		0.004458685		1.09	1.40	1.06	Elovl1	elongation of very long chain fatty acids (FEN1/Elo2, SUR4/Elo3, ye	0006629 // lipid metabolic process // inferred from electronic annotation///0006631 // fatty acid metabolic process // inferred from electronic annotation///0006633 // fatty acid bio
NM_001289150///NM	0.00710017			-1.19	-1.22	1.05	Fam110a	family with sequence similarity 110, member A	
NM_133681///XM_00	0.00967874			1.24	1.09	1.05	Tspan1	tetraspanin 1	0007166 // cell surface receptor signaling pathway // not recorded///0008283 // cell proliferation // not recorded///0016477 // cell migration // not recorded///0045807 // positive r
NM_001302116///NM	0.00917965			-1.16	1.02	1.05	Usp3	ubiquitin specific peptidase 3	0000122 // negative regulation of transcription from RNA polymerase II promoter // not recorded///0000278 // mitotic cell cycle // not recorded///0006281 // DNA repair // not reco
NM_175121///XM_00	5.62E-04			1.34	1.27	1.05	Slc38a2	solute carrier family 38, member 2	0003333 // amino acid transmembrane transport // inferred from direct assay///0006810 // transport // inferred from electronic annotation///0006811 // ion transport // inferred fr
NM_010880///XM_006529238		0.008975337		1.08	1.26	1.05	Ncl	nucleolin	0001525 // angiogenesis // not recorded///0006897 // endocytosis // not recorded///0007283 // spermatogenesis // inferred from electronic annotation///0032760 // positive regula
NM_133689///XM_00	0.00443535			1.24	1.12	1.05	Ppp1r32	protein phosphatase 1, regulatory subunit 32	
NM_001253822///NM	0.00369158			-1.26	-1.12	1.04	Irx3	Iroquois related homeobox 3	0001656 // metanephros development // inferred from expression pattern///0001822 // kidney development // not recorded///0006351 // transcription, DNA-templated // inferred f
NM_010381///XM_00	0.00811234			1.12	1.04	1.04	H2-Ea-ps	histocompatibility 2, class II antigen E alpha, pseudogene	0002376 // immune system process // inferred from electronic annotation///0002504 // antigen processing and presentation of peptide or polysaccharide antigen via MHC class II // i
NM_025727///XM_01	0.00659906			-1.18	-1.16	1.04	Klhl10	kelch-like 10	0000902 // cell morphogenesis // inferred from mutant phenotype///0007283 // spermatogenesis // inferred from electronic annotation///0007286 // spermatid development // infe
NM_011574///XM_00	0.00127548	0.001437307		1.18	1.24	1.04	Cirh1a	cirrhosis, autosomal recessive 1A (human)	0006351 // transcription, DNA-templated // inferred from electronic annotation///0006355 // regulation of transcription, DNA-templated // not recorded///0006364 // rRNA processi
NM_001303121///NM	0.00737425			-1.15	-1.13	1.04	Mycl	v-myc myelocytomatosis viral oncogene homolog, lung carcinoma	0006355 // regulation of transcription, DNA-templated // inferred from electronic annotation///0045607 // regulation of auditory receptor cell differentiation // inferred from genetic
NM_020510	0.0027706			-1.24	-1.04	1.04	Fzd2	frizzled homolog 2 (Drosophila)	0003149 // membranous septum morphogenesis // inferred from genetic interaction///0003150 // muscular septum morphogenesis // inferred from genetic interaction///0003151 /,
NM_001042611///NM	8.75E-04			1.26	1.19	1.04	Cp	ceruloplasmin	0006810 // transport // inferred from electronic annotation///0006811 // ion transport // inferred from electronic annotation///0006825 // copper ion transport // inferred from elec
NM_023579///XM_00	0.00659966			1.14	1.10	1.04	Ipo5	importin 5	0000059 // protein import into nucleus, docking // not recorded///0000060 // protein import into nucleus, translocation // not recorded///0006607 // NLS-bearing protein import int
NM_011345///XM_006496715		0.003151331		1.15	1.25	1.04	Sele	selectin, endothelial cell	0002092 // positive regulation of receptor internalization // not recorded///0002687 // positive regulation of leukocyte migration // inferred from genetic interaction///0006954 // in
NM_134072///XM_011244251		0.005143093		1.36	2.27	1.04	Akr1c14	aldo-keto reductase family 1, member C14	0021766 // hippocampus development // inferred from electronic annotation///0055114 // oxidation-reduction process // inferred from direct assay
NM_029035///XM_00	0.00305382			1.17	1.03	1.04	Spsb1	splA/ryanodine receptor domain and SOCS box containing 1	0016567 // protein ubiquitination // inferred from electronic annotation///0035556 // intracellular signal transduction // inferred from electronic annotation
NM_010496	0.00252012			-1.30	-1.01	1.03	Id2	inhibitor of DNA binding 2	0000122 // negative regulation of transcription from RNA polymerase II promoter // inferred from direct assay///0000122 // negative regulation of transcription from RNA polymeras
NM_020493///XM_00	0.0019873			1.27	1.24	1.03	Srf	serum response factor	0001569 // patterning of blood vessels // inferred from mutant phenotype///0001666 // response to hypoxia // inferred from electronic annotation///0001701 // in utero embryonic
NM_175158	0.00156376			1.17	-1.01	1.02	Utp20	UTP20, small subunit (SSU) processome component, homolog (yea	0006364 // rRNA processing // not recorded
NM_001039129///NM_001165971//		0.006765448		-1.08	1.21	1.02	Gm10052///Gm5643///Gm5803,	heterogeneous nuclear ribonucleoprotein A1 pseudogene///predic	0000380 // alternative mRNA splicing, via spliceosome // inferred from direct assay///0006397 // mRNA processing // inferred from electronic annotation///0006810 // transport // ir
NM_001242419///NM	0.00349892			1.25	1.11	1.02	Susd6	sushi domain containing 6	0006974 // cellular response to DNA damage stimulus // not recorded///0008219 // cell death // not recorded
NM_001163161///NM	0.00799863			-1.21	-1.13	1.01	Clec4d	C-type lectin domain family 4, member d	0002250 // adaptive immune response // inferred from mutant phenotype///0002292 // T cell differentiation involved in immune response // inferred from mutant phenotype///0002
NM_008331		0.007701153		-1.21	-1.40	1.01	Ifit1	interferon-induced protein with tetratricopeptide repeats 1	0002376 // immune system process // inferred from electronic annotation///0009615 // response to virus // inferred from mutant phenotype///0035457 // cellular response to interf
NM_013462///XM_00	0.00146379			-1.18	1.08	1.01	Adrb3	adrenergic receptor, beta 3	0002024 // diet induced thermogenesis // inferred from genetic interaction///0002025 // vasodilation by norepinephrine-epinephrine involved in regulation of systemic arterial blood
NM_001289716///NM	0.00114527			1.35	1.26	1.01	Bcl2l1	BCL2-like 1	0000910 // cytokinesis // not recorded///0001541 // ovarian follicle development // inferred from genetic interaction///0001541 // ovarian follicle development // inferred from mut

RefSeq Transcript ID	p (24h n=460)	p (48h n=657)	p (72h n=805)	FC 24h	FC 48h	FC 72h	Gene Symbol	Gene Title	Gene Ontology Biological Process
NM_001163290///NM_023341///XM		0.008151176		-1.04	-1.19	1.01	***Adck3***	aarF domain containing kinase 3	0006744 // ubiquinone biosynthetic process // not recorded///0016310 // phosphorylation // not recorded
NM_009008	0.00674332			-1.32	1.06	-1.01	***Rac2***	RAS-related C3 botulinum substrate 2	0006935 // chemotaxis // inferred from direct assay///0007015 // actin filament organization // not recorded///0007186 // G-protein coupled receptor signaling pathway // inferred f
NM_016974///XM_00	1.81E-04			-1.94	-1.29	-1.02	***Dbp***	D site albumin promoter binding protein	0001889 // liver development // inferred from electronic annotation///0006351 // transcription, DNA-templated // inferred from electronic annotation///0006355 // regulation of tra
NM_013559///XM_006504914///XM		0.004626031		1.03	-1.90	-1.02	***Hsph1***	heat shock 105kDa/110kDa protein 1	0006986 // response to unfolded protein // traceable author statement///0043524 // negative regulation of neuron apoptotic process // traceable author statement///0045345 // po
NM_013560		0.004077725		-1.54	-1.96	-1.05	***Hspb1***	heat shock protein 1	0001895 // retina homeostasis // inferred from electronic annotation///0006469 // negative regulation of protein kinase activity // inferred from direct assay///0006986 // response t
NM_145459	3.63E-04			-1.32	-1.04	-1.05	***Zfp503***	zinc finger protein 503	0006351 // transcription, DNA-templated // inferred from electronic annotation///0006355 // regulation of transcription, DNA-templated // inferred from electronic annotation///00(
NM_029809		0.009228555		-1.05	-1.21	-1.05	***Rnf225***	ring finger protein 225	
NM_145562		0.008691156		1.02	-1.20	-1.05	***Parm1***	prostate androgen-regulated mucin-like protein 1	0051973 // positive regulation of telomerase activity // not recorded
NM_001097979///NM	0.00617165	0.009847457		-1.44	-1.48	-1.06	***Hist1h2bc///Hist1h2be///Hist1h***	histone cluster 1, H2bc///histone cluster 1, H2be///histone cluster	0002227 // innate immune response in mucosa // not recorded///0006334 // nucleosome assembly // not recorded///0019731 // antibacterial humoral response // not recorded///0
NM_001302338///NM	0.00452163			-1.17	-1.11	-1.06	***Nfe2***	nuclear factor, erythroid derived 2	0006351 // transcription, DNA-templated // inferred from electronic annotation///0006355 // regulation of transcription, DNA-templated // inferred from electronic annotation///00(
NM_001252651///NM	9.84E-04			-1.25	-1.13	-1.06	***Csf3r***	colony stimulating factor 3 receptor (granulocyte)	0007155 // cell adhesion // inferred from electronic annotation///0019221 // cytokine-mediated signaling pathway // inferred from electronic annotation///0030593 // neutrophil ch
NM_177725	0.00192245			1.17	1.02	-1.06	***Lrrc8a***	leucine rich repeat containing 8A	0002329 // pre-B cell differentiation // inferred from mutant phenotype///0006810 // transport // inferred from electronic annotation///0006811 // ion transport // inferred from ele
NM_001003817	0.00473034			-1.15	-1.13	-1.06	***Erbb2***	v-erb-b2 erythroblastic leukemia viral oncogene homolog 2, neuro	0001889 // liver development // inferred from electronic annotation///0001934 // positive regulation of protein phosphorylation // inferred from mutant phenotype///0006351 // tra
	0.00104963			-1.21	1.18	-1.07	***Igk***	immunoglobulin kappa chain complex	0006898 // receptor-mediated endocytosis // ---///0006910 // phagocytosis, recognition // ---///0006911 // phagocytosis, engulfment // ---///0006958 // complement activation, clas
NM_026056///XM_006516946		0.004122173		-1.07	-1.28	-1.07	***Cap2***	CAP, adenylate cyclase-associated protein, 2 (yeast)	0000902 // cell morphogenesis // inferred from electronic annotation///0007010 // cytoskeleton organization // inferred from electronic annotation
NM_009621	0.00628537			1.34	-1.03	-1.07	***Adamts1***	a disintegrin-like and metallopeptidase (reprolysin type) with thron	0001542 // ovulation from ovarian follicle // inferred from mutant phenotype///0001822 // kidney development // inferred from mutant phenotype///0006508 // proteolysis // infer
NM_008610///XM_006530751		0.009760939		-1.11	-1.27	-1.08	***Mmp2***	matrix metallopeptidase 2	0001525 // angiogenesis // inferred from electronic annotation///0001541 // ovarian follicle development // not recorded///0001666 // response to hypoxia // inferred from direct as
NM_001160215///NM	0.00949219	0.009798125		-1.14	-1.18	-1.08	***Fcrla***	Fc receptor-like A	0030154 // cell differentiation // inferred from electronic annotation
NM_031172///NM_17	0.00359042			-1.25	-1.07	-1.08	***Hist3h2a///Trim17***	histone cluster 3, H2a///tripartite motif-containing 17	0006325 // chromatin organization // not recorded///0006337 // nucleosome disassembly // not recorded///0006342 // chromatin silencing // not recorded///0006914 // autophagy
NM_025828		0.009223028		-1.13	-1.22	-1.08	***Lman2***	lectin, mannose-binding 2	0006810 // transport // inferred from electronic annotation///0006890 // retrograde vesicle-mediated transport, Golgi to ER // not recorded///0015031 // protein transport // inferre
NM_054094///XM_00	0.00566332			1.28	1.02	-1.08	***Acsm1***	acyl-CoA synthetase medium-chain family member 1	0006629 // lipid metabolic process // inferred from electronic annotation///0006631 // fatty acid metabolic process // inferred from electronic annotation///0006633 // fatty acid bio
NM_008584	0.00111136			-1.28	-1.19	-1.09	***Meox2***	mesenchyme homeobox 2	0001525 // angiogenesis // inferred from mutant phenotype///0001757 // somite specification // inferred from genetic interaction///0006351 // transcription, DNA-templated // infer
NM_001316360///NM	6.01E-04			-1.31	1.02	-1.09	***Bmp4***	bone morphogenetic protein 4	0000122 // negative regulation of transcription from RNA polymerase II promoter // inferred from direct assay///0000122 // negative regulation of transcription from RNA polymeras
NM_001013392///NM	0.00130643			-1.20	-1.11	-1.09	***Rreb1***	ras responsive element binding protein 1	0000122 // negative regulation of transcription from RNA polymerase II promoter // inferred from mutant phenotype///0000122 // negative regulation of transcription from RNA poly
NM_001005846///NM	0.004561			-1.23	-1.15	-1.09	***Mcoln2***	mucolipin 2	0006810 // transport // inferred from electronic annotation///0006811 // ion transport // inferred from electronic annotation
NM_001111043///NM_001111044//		4.20E-04		-1.24	-1.65	-1.09	***Serpinh1***	serine (or cysteine) peptidase inhibitor, clade H, member 1	0003433 // chondrocyte development involved in endochondral bone morphogenesis // inferred from mutant phenotype///0010951 // negative regulation of endopeptidase activity ,
NM_008788///XM_006504558		0.004281425		-1.07	-1.28	-1.10	***Pcolce***	procollagen C-endopeptidase enhancer protein	0006508 // proteolysis // inferred from direct assay///0010952 // positive regulation of peptidase activity // not recorded
NM_019564		0.001425422		-1.19	-1.30	-1.10	***Htra1***	HtrA serine peptidase 1	0001558 // regulation of cell growth // inferred from electronic annotation///0001890 // placenta development // inferred from mutant phenotype///0006508 // proteolysis // inferr
NM_010882		0.009879425		-1.04	-1.27	-1.10	***Ndn***	necdin	0001764 // neuron migration // inferred from mutant phenotype///0003016 // respiratory system process // inferred from mutant phenotype///0006351 // transcription, DNA-templ
NM_028044		0.005925631		-1.01	-1.21	-1.10	***Cnn3***	calponin 3, acidic	0030855 // epithelial cell differentiation // inferred from electronic annotation///0031032 // actomyosin structure organization // inferred from electronic annotation///0032780 // n
NM_015786	3.87E-04			-1.38	-1.08	-1.10	***Hist1h1c***	histone cluster 1, H1c	0000122 // negative regulation of transcription from RNA polymerase II promoter // inferred from genetic interaction///0006334 // nucleosome assembly // inferred from electronic a
NM_007464///XM_00	0.00106683			1.25	1.05	-1.11	***Birc3***	baculoviral IAP repeat-containing 3	0006915 // apoptotic process // inferred from electronic annotation///0016567 // protein ubiquitination // not recorded///0031398 // positive regulation of protein ubiquitination //
NM_022319///XM_006511334		0.007695761		-1.05	-1.19	-1.11	***Clstn2***	calsyntenin 2	0007155 // cell adhesion // inferred from electronic annotation///0007156 // homophilic cell adhesion via plasma membrane adhesion molecules // inferred from electronic annotati
NM_009789		0.009267952		-1.07	-1.26	-1.11	***S100g***	S100 calcium binding protein G	
NM_001290486///NM_010336///NM		0.003096117		1.03	-1.22	-1.11	***Lpar1***	lysophosphatidic acid receptor 1	0000187 // activation of MAPK activity // inferred from direct assay///0007165 // signal transduction // inferred from electronic annotation///0007186 // G-protein coupled receptor
NM_007735///XM_01	0.00484402	0.002351095		-1.24	-1.27	-1.11	***Col4a4***	collagen, type IV, alpha 4	0032836 // glomerular basement membrane development // inferred from mutant phenotype///0032836 // glomerular basement membrane development // not recorded
NM_008730		0.003843463		-1.13	-1.25	-1.11	***Nptx1***	neuronal pentraxin 1	0006839 // mitochondrial transport // not recorded///0035865 // cellular response to potassium ion // not recorded///0043653 // mitochondrial fragmentation involved in apoptotic
NM_001008542///NM	0.0081936			-1.20	-1.03	-1.11	***Mxi1***	MAX interactor 1, dimerization protein	0000122 // negative regulation of transcription from RNA polymerase II promoter // inferred from direct assay///0006351 // transcription, DNA-templated // inferred from electronic
	8.43E-05			-1.37	-1.26	-1.12	***Ighd***	immunoglobulin heavy constant delta	0016064 // immunoglobulin mediated immune response // inferred from mutant phenotype///0016446 // somatic hypermutation of immunoglobulin genes // inferred from mutant p
NM_010104		0.008150942		-1.12	-1.43	-1.12	***Edn1***	endothelin 1	0000122 // negative regulation of transcription from RNA polymerase II promoter // not recorded///0001501 // skeletal system development // inferred from mutant phenotype///00
NM_020581	0.00779299			1.29	1.02	-1.12	***Angptl4***	angiopoietin-like 4	0001525 // angiogenesis // inferred from electronic annotation///0001666 // response to hypoxia // inferred from electronic annotation///0009267 // cellular response to starvation ,
NM_053195///XM_006500452		0.008679129		-1.05	-1.18	-1.12	***Slc24a3***	solute carrier family 24 (sodium/potassium/calcium exchanger), m	0006810 // transport // inferred from electronic annotation///0006811 // ion transport // inferred from electronic annotation///0006812 // cation transport // not recorded///00068
NM_020495///NM_178235///XM_00		0.007755761		-1.09	-1.19	-1.12	***Slco1b2***	solute carrier organic anion transporter family, member 1b2	0001889 // liver development // inferred from electronic annotation///0006810 // transport // inferred from electronic annotation///0006811 // ion transport // inferred from electr
NM_008626///XM_006532406		0.008439485		-1.08	-1.37	-1.12	***Mrc2***	mannose receptor, C type 2	0001649 // osteoblast differentiation // not recorded///0006897 // endocytosis // inferred from electronic annotation///0030574 // collagen catabolic process // not recorded
NM_001285812///NM	0.00613731			-1.16	-1.08	-1.13	***Tmem53***	transmembrane protein 53	
NM_145434	2.70E-05	0.005097197		-1.80	-1.38	-1.13	***Nr1d1***	nuclear receptor subfamily 1, group D, member 1	0000122 // negative regulation of transcription from RNA polymerase II promoter // not recorded///0005978 // glycogen biosynthetic process // inferred from mutant phenotype///0
NM_013712///XM_011247596///XM		0.008133078		-1.05	-1.21	-1.13	***Itgb1bp2***	integrin beta 1 binding protein 2	
NM_001033257///NM_001195065//		0.007819721		1.00	-1.25	-1.13	***Phactr2***	phosphatase and actin regulator 2	0043086 // negative regulation of catalytic activity // inferred from electronic annotation
NM_015792///XM_006497505///XM		0.007663231		-1.12	-1.18	-1.13	***Fbxo18***	F-box protein 18	0000724 // double-strand break repair via homologous recombination // inferred from sequence or structural similarity///0000737 // DNA catabolic process, endonucleolytic // not re
NM_019419	0.0097243			-1.16	-1.21	-1.13	***Arl6ip1***	ADP-ribosylation factor-like 6 interacting protein 1	0006613 // cotranslational protein targeting to membrane // inferred from direct assay
NM_172448///XM_00	0.00818185			-1.21	-1.11	-1.13	***Rnf43***	ring finger protein 43	0016055 // Wnt signaling pathway // inferred from electronic annotation///0016567 // protein ubiquitination // not recorded///0030178 // negative regulation of Wnt signaling pathw
NR_028264		0.002430072		-1.21	-1.33	-1.13	***Dleu2***	deleted in lymphocytic leukemia, 2	0010977 // negative regulation of neuron projection development // inferred from genetic interaction///0014043 // negative regulation of neuron maturation // inferred from genetic
NM_027416	0.00471647			1.17	-1.03	-1.13	***Calml3***	calmodulin-like 3	
NM_001099308///NM_001099349//		0.006185873		-1.04	-1.31	-1.13	***Gm11007///Gm14308///Gm143***	predicted gene 11007///predicted gene 14308///predicted gene 14	0006355 // regulation of transcription, DNA-templated // inferred from electronic annotation
NM_001042605///NM	0.00686569			-1.28	-1.22	-1.13	***Cd74***	CD74 antigen (invariant polypeptide of major histocompatibility co	0000187 // activation of MAPK activity // not recorded///0001516 // prostaglandin biosynthetic process // not recorded///0001961 // positive regulation of cytokine-mediated signali
NM_013912		0.006826226		-1.05	-1.24	-1.13	***Apln***	apelin	0002026 // regulation of the force of heart contraction // not recorded///0007165 // signal transduction // inferred from electronic annotation///0007186 // G-protein coupled recep
NM_029436///XM_00	0.00648036			-1.18	-1.11	-1.14	***Klhl24***	kelch-like 24	0016567 // protein ubiquitination // not recorded///2000312 // regulation of kainate selective glutamate receptor activity // inferred from electronic annotation
NM_010217	0.00849896			1.35	1.03	-1.14	***Ctgf***	connective tissue growth factor	0001502 // cartilage condensation // inferred from direct assay///0001503 // ossification // inferred from mutant phenotype///0001525 // angiogenesis // inferred from direct assay/
NM_001122892///NM	0.00254945	0.001494276		-1.18	-1.30	-1.14	***Fyn***	Fyn proto-oncogene	0001764 // neuron migration // inferred from genetic interaction///0001764 // neuron migration // inferred from mutant phenotype///0002250 // adaptive immune response // infer
NM_001276383///NM_153060///XM		5.97E-04		-1.11	-1.37	-1.14	***Spns2***	spinster homolog 2	0001782 // B cell homeostasis // inferred from mutant phenotype///0002260 // lymphocyte homeostasis // inferred from mutant phenotype///0002920 // regulation of humoral imm
NM_011948///XM_006523329		0.0091936		-1.10	-1.18	-1.14	***Map3k4***	mitogen-activated protein kinase kinase kinase 4	0000165 // MAPK cascade // not recorded///0000186 // activation of MAPKK activity // not recorded///0000187 // activation of MAPK activity // not recorded///0001890 // placenta
NM_172398///XM_00	0.00854638			-1.19	-1.10	-1.14	***Akr1b10***	aldo-keto reductase family 1, member B10 (aldose reductase)	0055114 // oxidation-reduction process // inferred from electronic annotation
NM_013737///XM_00	0.00645464			-1.14	-1.04	-1.14	***Pla2g7***	phospholipase A2, group VII (platelet-activating factor acetylhydrol	0006629 // lipid metabolic process // inferred from electronic annotation///0006954 // inflammatory response // traceable author statement///0016042 // lipid catabolic process // i
NM_010391///XM_01	0.0016031			-1.24	-1.18	-1.14	***H2-Q10***	histocompatibility 2, Q region locus 10	0002376 // immune system process // inferred from electronic annotation///0002474 // antigen processing and presentation of peptide antigen via MHC class I // not recorded///000
NM_021534	0.00343374			-1.19	-1.13	-1.14	***Pxmp4***	peroxisomal membrane protein 4	
NM_007903		0.004087877		-1.05	-1.22	-1.14	***Edn3***	endothelin 3	0001755 // neural crest cell migration // inferred from mutant phenotype///0002690 // positive regulation of leukocyte chemotaxis // not recorded///0003100 // regulation of system
NM_001163640///NM	0.00511251			1.21	-1.06	-1.14	***Chn2***	chimerin 2	0007165 // signal transduction // inferred from electronic annotation///0035556 // intracellular signal transduction // inferred from electronic annotation///0043547 // positive regul
NM_001122899///NM_010704///NM		0.009543374		-1.10	-1.28	-1.15	***Lepr***	leptin receptor	0001525 // angiogenesis // not recorded///0007165 // signal transduction // traceable author statement///0007186 // G-protein coupled receptor signaling pathway // not recorded/
NM_001281852///NM	3.36E-04			-2.85	-1.69	-1.15	***S100a9***	S100 calcium binding protein A9 (calgranulin B)	0002376 // immune system process // inferred from electronic annotation///0002523 // leukocyte migration involved in inflammatory response // not recorded///0002544 // chronic
NM_008520///XM_00	0.00353953	0.003174162		-1.22	-1.29	-1.15	***Ltbp3***	latent transforming growth factor beta binding protein 3	0001501 // skeletal system development // inferred from mutant phenotype///0007179 // transforming growth factor beta receptor signaling pathway // inferred from mutant phenc
NM_025785///XM_00	0.00447636			-1.16	-1.12	-1.15	***Fbxo25***	F-box protein 25	0016567 // protein ubiquitination // inferred from direct assay
NM_133351///XM_00	0.00308314			-1.25	-1.08	-1.15	***Prss8***	protease, serine 8 (prostasin)	0001942 // hair follicle development // inferred from mutant phenotype///0006508 // proteolysis // inferred from electronic annotation///0010765 // positive regulation of sodium ic
NM_001048054///NM	1.99E-04			1.28	1.13	-1.15	***Dusp16***	dual specificity phosphatase 16	0000188 // inactivation of MAPK activity // inferred from direct assay///0006470 // protein dephosphorylation // not recorded///0016310 // phosphorylation // inferred from electro
NM_012026///XM_00	0.00298236			-1.22	-1.19	-1.15	***Arhgef28***	Rho guanine nucleotide exchange factor (GEF) 28	0021955 // central nervous system neuron axonogenesis // inferred from mutant phenotype///0030154 // cell differentiation // inferred from electronic annotation///0035023 // reg
NM_001159329///NM_025332///XM		0.00630718		-1.15	-1.27	-1.15	***Gtpbp8***	GTP-binding protein 8 (putative)	0000917 // barrier septum assembly // inferred from electronic annotation
NM_152895///XM_01	0.00344807			-1.18	-1.22	-1.16	***Kdm5b***	lysine (K)-specific demethylase 5B	0006351 // transcription, DNA-templated // inferred from electronic annotation///0006355 // regulation of transcription, DNA-templated // inferred from electronic annotation///00(
NM_001164059///NM	0.009107			-1.15	-1.16	-1.16	***Sell***	selectin, lymphocyte	0007155 // cell adhesion // inferred from electronic annotation///0033198 // response to ATP // inferred from direct assay///0034201 // response to oleic acid // inferred from electr
NM_025786	0.00187184			-1.30	-1.24	-1.16	***Rnf186***	ring finger protein 186	
NM_001201413///NM_001201414//		0.001603473		-1.10	-1.29	-1.16	***Apbb2***	amyloid beta (A4) precursor protein-binding, family B, member 2	0001764 // neuron migration // inferred from genetic interaction///0006355 // regulation of transcription, DNA-templated // inferred from direct assay///0007050 // cell cycle arrest
NM_001025431///NM	0.00906965			-1.24	-1.22	-1.16	***Btbd3***	BTB (POZ) domain containing 3	0007399 // nervous system development // inferred from electronic annotation///0021987 // cerebral cortex development // inferred from mutant phenotype///0048813 // dendrite
NM_023821///NM_028225///XM_01		0.001219075		-1.02	-1.30	-1.16	***Cmya5***	cardiomyopathy associated 5	0014733 // regulation of skeletal muscle adaptation // inferred from genetic interaction///0032513 // negative regulation of protein phosphatase type 2B activity // inferred from dire
NM_001083917///NM_001286614//		0.009017699		1.10	-1.52	-1.16	***Scn3b***	sodium channel, voltage-gated, type III, beta	0006810 // transport // inferred from electronic annotation///0006811 // ion transport // inferred from electronic annotation///0006814 // sodium ion transport // not recorded///0
NM_026162///XM_006497531		0.003870625		1.01	-1.25	-1.17	***Plxdc2***	plexin domain containing 2	
NM_009180///XR_388	0.00553054	0.006166431		-1.26	-1.32	-1.17	***St6galnac2***	ST6 (alpha-N-acetyl-neuraminyl-2,3-beta-galactosyl-1,3)-N-acetylg	0001574 // ganglioside biosynthetic process // not recorded///0006486 // protein glycosylation // inferred from electronic annotation///0009311 // oligosaccharide metabolic proces
NM_001025391///NM_015752		0.008423599		-1.09	-1.19	-1.17	***Sufu***	suppressor of fused homolog (Drosophila)	0000122 // negative regulation of transcription from RNA polymerase II promoter // inferred from direct assay///0000122 // negative regulation of transcription from RNA polymeras
NM_011729///XM_006495890		0.009094499		-1.01	-1.23	-1.17	***Ercc5***	excision repair cross-complementing rodent repair deficiency, com	0006281 // DNA repair // inferred from mutant phenotype///0006283 // transcription-coupled nucleotide-excision repair // not recorded///0006289 // nucleotide-excision repair // ir

RefSeq Transcript ID	p (24h n=460)	p (48h n=657)	p (72h n=805)	FC 24h	FC 48h	FC 72h	Gene Symbol	Gene Title	Gene Ontology Biological Process
NM_001081309///NM	0.00589131			**-1.15**	-1.12	-1.17	Pik3r4	phosphatidylinositol 3 kinase, regulatory subunit, polypeptide 4, p1	0006468 // protein phosphorylation // not recorded///0006623 // protein targeting to vacuole // not recorded///0016236 // macroautophagy // not recorded///0016310 // phospho
NM_001304475///NM_145446///NM_175337///XM			0.00982835	-1.10	-1.06	**-1.17**	Mlh3	mutL homolog 3 (E coli)	0006298 // mismatch repair // inferred from genetic interaction///0006298 // mismatch repair // inferred from mutant phenotype///0006974 // cellular response to DNA damage stir
NM_001033127///NM_001111017///NM_177311//			0.00674644	1.04	-1.04	**-1.17**	Serac1	serine active site containing 1	0006629 // lipid metabolic process // inferred from electronic annotation///0008654 // phospholipid biosynthetic process // inferred from electronic annotation///0030198 // extrace
NM_016917///XM_00	0.0081068			**-1.15**	-1.10	-1.17	Slc40a1	solute carrier family 40 (iron-regulated transporter), member 1	0002260 // lymphocyte homeostasis // inferred from mutant phenotype///0003158 // endothelium development // inferred from mutant phenotype///0006810 // transport // inferr
NM_008729///XM_006520032///XM		0.007134052		-1.03	**-1.23**	-1.17	Ctnnd2	catenin (cadherin associated protein), delta 2	0001763 // morphogenesis of a branching structure // inferred from direct assay///0006351 // transcription, DNA-templated // inferred from electronic annotation///0006355 // regu
NM_011812///XM_006515864///XM		3.36E-04		-1.13	**-1.44**	-1.17	Fbln5	fibulin 5	0001558 // regulation of cell growth // inferred from electronic annotation///0007155 // cell adhesion // inferred from electronic annotation///0030198 // extracellular matrix organi
NM_001114385///NM_031258///XM_006529001//			0.00810582	-1.04	1.01	**-1.17**	Chrdl1	chordin-like 1	0001503 // ossification // inferred from electronic annotation///0001654 // eye development // not recorded///0001709 // cell fate determination // not recorded///0007275 // mul
NM_133858///NM_1S	0.00886008			**-1.15**	-1.11	-1.17	Fam63a	family with sequence similarity 63, member A	
NM_001290745///NM_016965///XM_006499881//			0.00861665	-1.07	-1.13	**-1.17**	Nckap1	NCK-associated protein 1	0001701 // in utero embryonic development // inferred from mutant phenotype///0001756 // somitogenesis // inferred from mutant phenotype///0001843 // neural tube closure //
NM_001146275///NM	0.00288293			**-1.69**	-1.31	-1.17	Iigp1	interferon inducible GTPase 1	0002376 // immune system process // inferred from electronic annotation///0008152 // metabolic process // inferred from electronic annotation///0010506 // regulation of autopha
NM_011657///XM_006505909///XR_		0.008048018	0.0065621	-1.06	**-1.17**	**-1.17**	Tulp3	tubby-like protein 3	0001841 // neural tube formation // inferred from mutant phenotype///0001843 // neural tube closure // inferred from mutant phenotype///0007275 // multicellular organismal dev
NM_015732///XM_00	0.00927946			**-1.18**	-1.17	-1.17	Axin2	axin2	0001756 // somitogenesis // inferred from direct assay///0001934 // positive regulation of protein phosphorylation // not recorded///0001957 // intramembranous ossification // infe
NM_001081300///XM	0.00730591			**-1.15**	-1.19	-1.18	Tshz1	teashirt zinc finger family member 1	0006351 // transcription, DNA-templated // inferred from electronic annotation///0006355 // regulation of transcription, DNA-templated // inferred from electronic annotation///000
NM_053185///XM_011247883			0.00667963	-1.02	-1.15	**-1.18**	Col4a6	collagen, type IV, alpha 6	0030198 // extracellular matrix organization // inferred from direct assay///0071230 // cellular response to amino acid stimulus // inferred from direct assay
NM_010453	5.74E-05	0.001072674		**-1.48**	**-1.43**	-1.18	Hoxa5	homeobox A5	0001501 // skeletal system development // inferred from mutant phenotype///0002009 // morphogenesis of an epithelium // inferred from mutant phenotype///0003016 // respirato
NM_001079824///XM	0.00457865	0.006742058		**-1.18**	**-1.22**	-1.18	Hnrnph3	heterogeneous nuclear ribonucleoprotein H3	0030855 // epithelial cell differentiation // inferred from electronic annotation
NM_001177773///NM_001177774///NM_080428//			0.00759114	-1.05	-1.13	**-1.18**	Fbxw7	F-box and WD-40 domain protein 7	0001570 // vasculogenesis // inferred from mutant phenotype///0001944 // vasculature development // traceable author statement///0006355 // regulation of transcription, DNA-te
NM_007792			0.00960092	1.01	1.11	**-1.18**	Csrp2	cysteine and glycine-rich protein 2	0007275 // multicellular organismal development // inferred from electronic annotation///0030154 // cell differentiation // inferred from electronic annotation///0045445 // myobla
NM_029738///XM_006522701///XM_006522702//			0.00654555	-1.02	-1.15	**-1.19**	Cluap1	clusterin associated protein 1	0001843 // neural tube closure // inferred from mutant phenotype///0001947 // heart looping // inferred from mutant phenotype///0007224 // smoothened signaling pathway // infe
NM_001313939///NM	3.58E-04			**-1.41**	-1.27	-1.19	Cd79b	CD79B antigen	0002250 // adaptive immune response // inferred from electronic annotation///0002376 // immune system process // inferred from electronic annotation///0007166 // cell surface r
NM_010706///XM_006539566			0.00960752	1.01	-1.10	**-1.19**	Lgals4	lectin, galactose binding, soluble 4	
NM_001162532		5.10E-04		-1.17	**-1.45**	-1.19	Fam174b	family with sequence similarity 174, member B	
NM_011748///NM_178733///XM_006539948///XM			0.00608039	-1.06	-1.12	**-1.19**	Zfp14	zinc finger protein 14	0006355 // regulation of transcription, DNA-templated // not recorded
NM_001306088///NM	0.00939547			**-1.15**	-1.08	-1.19	Dnmbp	dynamin binding protein	0035023 // regulation of Rho protein signal transduction // inferred from electronic annotation///0035556 // intracellular signal transduction // inferred from electronic annotation///
NM_001177778///NM_001177779//		0.009131226		-1.12	**-1.18**	-1.19	Dlg3	discs, large homolog 3 (Drosophila)	0001736 // establishment of planar polarity // inferred from mutant phenotype///0007268 // synaptic transmission // not recorded///0007399 // nervous system development // not
NM_021719			0.00454522	-1.06	-1.17	**-1.19**	Cldn15	claudin 15	0006810 // transport // inferred from electronic annotation///0006811 // ion transport // inferred from electronic annotation///0016338 // calcium-independent cell-cell adhesion vi
NM_001085515///XM	0.0037944			**-1.22**	-1.19	-1.19	AI464131	expressed sequence AI464131	0005975 // carbohydrate metabolic process // inferred from electronic annotation///0008152 // metabolic process // inferred from electronic annotation///0043568 // positive regul
NM_025347///NM_02	0.00966351			**-1.21**	-1.09	-1.19	Ypel3	yippee-like 3 (Drosophila)	0006915 // apoptotic process // inferred from electronic annotation///2000774 // positive regulation of cellular senescence // not recorded
NM_001136076///NM_011031///XM		0.001857876		-1.22	**-1.37**	-1.19	P4ha2	procollagen-proline, 2-oxoglutarate 4-dioxygenase (proline 4-hydro	0018401 // peptidyl-proline hydroxylation to 4-hydroxy-L-proline // inferred from direct assay///0055114 // oxidation-reduction process // inferred from electronic annotation
NM_133238	0.00948798	0.006270198		**-1.18**	**-1.43**	-1.19	Cd209a	CD209a antigen	0006897 // endocytosis // inferred from electronic annotation///0010468 // regulation of gene expression // not recorded///0030193 // regulation of blood coagulation // not record
NM_010225	0.00195853	0.004991675		**-1.22**	**-1.23**	-1.19	Foxf2	forkhead box F2	0006351 // transcription, DNA-templated // inferred from electronic annotation///0006355 // regulation of transcription, DNA-templated // inferred from electronic annotation///000
NM_001285446///NM_009823///NM_172860///XM			0.00982678	-1.00	-1.15	**-1.19**	Cbfa2t2	core-binding factor, runt domain, alpha subunit 2, translocated to,	0006351 // transcription, DNA-templated // inferred from electronic annotation///0006355 // regulation of transcription, DNA-templated // inferred from electronic annotation///001
NM_033373	0.00622604			**-1.17**	-1.12	-1.19	Krt23	keratin 23	
NM_001109988///NM_001109989//		0.003853193		-1.15	**-1.49**	-1.19	Nrep	neuronal regeneration related protein	0017015 // regulation of transforming growth factor beta receptor signaling pathway // inferred from direct assay///0031103 // axon regeneration // inferred from electronic annotat
NM_020608///XM_006524699///XM_006524702//			0.00983081	-1.02	-1.17	**-1.19**	Cramp1l	Crm, cramped-like (Drosophila)	
NM_008984///XM_006523864///XM		0.002538469		-1.04	**-1.29**	-1.19	Ptprm	protein tyrosine phosphatase, receptor type, M	0001937 // negative regulation of endothelial cell proliferation // not recorded///0006470 // protein dephosphorylation // not recorded///0007155 // cell adhesion // inferred from e
NM_022814			0.0088749	1.05	-1.01	**-1.19**	Svep1	sushi, von Willebrand factor type A, EGF and pentraxin domain con	0007155 // cell adhesion // inferred from electronic annotation
NM_013906			0.00771691	-1.03	-1.04	**-1.19**	Adamts8	a disintegrin-like and metallopeptidase (reprolysin type) with thron	0006508 // proteolysis // inferred from electronic annotation
NM_007655	0.00117669			**-1.50**	-1.32	-1.19	Cd79a	CD79A antigen (immunoglobulin-associated alpha)	0002250 // adaptive immune response // inferred from electronic annotation///0002376 // immune system process // inferred from electronic annotation///0007166 // cell surface r
NM_001167884///NM_001167885///NM_0011678			0.00577843	-1.05	-1.12	**-1.19**	Suv420h1	suppressor of variegation 4-20 homolog 1 (Drosophila)	0006351 // transcription, DNA-templated // inferred from electronic annotation///0006355 // regulation of transcription, DNA-templated // inferred from electronic annotation///000
NM_011020///XM_006500766///XM		0.002094896		-1.23	**-1.78**	-1.20	Hspa4l	heat shock protein 4 like	0006457 // protein folding // not recorded///0006986 // response to unfolded protein // not recorded
NM_009061		0.009439616		-1.12	**-1.32**	-1.20	Rgs2	regulator of G-protein signaling 2	0006417 // regulation of translation // inferred from electronic annotation///0007049 // cell cycle // inferred from electronic annotation///0007186 // G-protein coupled receptor sig
NM_001285793///NM_019804///XM		0.009043271		-1.10	**-1.35**	-1.20	B4galt4	UDP-Gal:betaGlcNAc beta 1,4-galactosyltransferase, polypeptide 4	0005975 // carbohydrate metabolic process // inferred from electronic annotation///0006486 // protein glycosylation // inferred from electronic annotation
NM_007533///XM_006539486///XR_881644			0.00936886	-1.15	1.04	**-1.20**	Bckdha	branched chain ketoacid dehydrogenase E1, alpha polypeptide	0007584 // response to nutrient // inferred from electronic annotation///0008152 // metabolic process // inferred from electronic annotation///0009083 // branched-chain amino ac
XM_357640///XM_99	4.71E-05			**-1.46**	1.03	-1.20	Igk-V28///Igkc///Igkj1///Igkv1-1	immunoglobulin kappa chain variable 28 (V28)///immunoglobulin	0006898 // receptor-mediated endocytosis // not recorded///0006910 // phagocytosis, recognition // not recorded///0006911 // phagocytosis, engulfment // not recorded///00695
NM_008256			0.00476513	-1.12	-1.02	**-1.20**	Hmgcs2	3-hydroxy-3-methylglutaryl-Coenzyme A synthase 2	0001822 // kidney development // inferred from electronic annotation///0001889 // liver development // inferred from electronic annotation///0006629 // lipid metabolic process //
NM_023799///XM_01	0.00745062			**-1.16**	-1.34	-1.20	Mgea5	meningioma expressed antigen 5 (hyaluronidase)	0006044 // N-acetylglucosamine metabolic process // not recorded///0006517 // protein deglycosylation // not recorded///0006612 // protein targeting to membrane // not recorde
NM_013905			0.00437325	-1.03	1.11	**-1.20**	Heyl	hairy/enhancer-of-split related with YRPW motif-like	0003151 // outflow tract morphogenesis // inferred from genetic interaction///0003181 // atrioventricular valve morphogenesis // inferred from genetic interaction///0003184 // pul
	0.00366991			**-1.44**	-1.01	-1.20	Iglv1///Iglc1	immunoglobulin lambda variable 1///immunoglobulin lambda constant 1	
NM_001302559///NM	0.00129079	0.008755075		**-1.45**	**-1.43**	-1.20	Slfn4	schlafen 4	
NM_148938			0.00913573	-1.08	-1.14	**-1.20**	Slc1a3	solute carrier family 1 (glial high affinity glutamate transporter), me	0002230 // positive regulation of defense response to virus by host // not recorded///0006536 // glutamate metabolic process // inferred from mutant phenotype///0006537 // gluta
NM_001127351///NM_001177804///NM_022433//			0.00605833	-1.00	-1.09	**-1.20**	Sirt3	sirtuin 3	0006476 // protein deacetylation // inferred from direct assay///0006476 // protein deacetylation // not recorded///0007568 // aging // inferred from electronic annotation///00090
NM_001285917///NM_029653///NM		0.005758764		-1.12	**-1.24**	-1.20	Dapk1	death associated protein kinase 1	0006417 // regulation of translation // inferred from electronic annotation///0006468 // protein phosphorylation // inferred from direct assay///0006468 // protein phosphorylation ,
NM_019819///XM_00	0.0038857			**-1.21**	1.07	-1.20	Dusp14	dual specificity phosphatase 14	0000188 // inactivation of MAPK activity // inferred from electronic annotation///0006470 // protein dephosphorylation // inferred from electronic annotation///0016311 // dephosp
NM_018883///XM_006533760///XM		0.002545798		-1.26	**-1.30**	-1.20	Camkk1	calcium/calmodulin-dependent protein kinase kinase 1, alpha	0006468 // protein phosphorylation // inferred from electronic annotation///0016310 // phosphorylation // inferred from electronic annotation///0032147 // activation of protein ki
NM_001102455///NM_001102456//		0.006516694		-1.08	**-1.25**	-1.20	Aplp2	amyloid beta (A4) precursor-like protein 2	0001967 // suckling behavior // inferred from genetic interaction///0006878 // cellular copper ion homeostasis // inferred from mutant phenotype///0007176 // regulation of epiderr
NM_001310659///NM_008342///XM		0.007797964		-1.09	**-1.27**	-1.20	Igfbp2	insulin-like growth factor binding protein 2	0001558 // regulation of cell growth // inferred from electronic annotation///0007165 // signal transduction // inferred from electronic annotation///0007565 // female pregnancy //
NM_029869///NM_133906///XM_0C		0.004646215		-1.05	**-1.28**	-1.20	Zkscan1	zinc finger with KRAB and SCAN domains 1	0006351 // transcription, DNA-templated // inferred from electronic annotation///0006355 // regulation of transcription, DNA-templated // not recorded
NM_001162973///NM_001162974///NM_027053//			0.00608147	1.00	-1.16	**-1.20**	Lrrc51	leucine rich repeat containing 51	
NM_010062			0.00931373	-1.11	-1.10	**-1.21**	Dnase2a	deoxyribonuclease II alpha	0000737 // DNA catabolic process, endonucleolytic // inferred from mutant phenotype///0006259 // DNA metabolic process // inferred from electronic annotation///0006308 // DNA
NM_011782///XM_011246127			0.00749509	-1.11	-1.14	**-1.21**	Adamts5	a disintegrin-like and metallopeptidase (reprolysin type) with thron	0006508 // proteolysis // inferred from electronic annotation///0042742 // defense response to bacterium // inferred from genetic interaction
NM_025510///XM_006533940			0.00698212	-1.07	-1.14	**-1.21**	Adprm	ADP-ribose/CDP-alcohol diphosphatase, manganese dependent	0008152 // metabolic process // inferred from electronic annotation
NM_001170537///NM	0.00910976		0.00994408	**-1.14**	-1.27	**-1.21**	Mef2c	myocyte enhancer factor 2C	0000122 // negative regulation of transcription from RNA polymerase II promoter // inferred from genetic interaction///0000122 // negative regulation of transcription from RNA poly
NM_001037098///NM_026495///XM_006498259//			0.00475203	-1.03	-1.04	**-1.21**	Nacc2	nucleus accumbens associated 2, BEN and BTB (POZ) domain conta	0008285 // negative regulation of cell proliferation // not recorded///0010608 // posttranscriptional regulation of gene expression // not recorded///0016575 // histone deacetylatior
NM_174847///XM_006522976///XM		0.002111156		-1.02	**-1.29**	-1.21	C2cd2	C2 calcium-dependent domain containing 2	
NM_010382		0.002069863		-1.25	**-1.28**	-1.21	H2-Eb1	histocompatibility 2, class II antigen E beta	0002376 // immune system process // inferred from electronic annotation///0002504 // antigen processing and presentation of peptide or polysaccharide antigen via MHC class II // i
NM_001285928///NM_001285929//		0.008207412		-1.06	**-1.17**	-1.21	Ubxn10	UBX domain protein 10	
NM_008091///XM_00	0.00129949			**-1.28**	-1.03	-1.21	Gata3	GATA binding protein 3	0000122 // negative regulation of transcription from RNA polymerase II promoter // inferred from direct assay///0000902 // cell morphogenesis // inferred from mutant phenotype///
NM_001171147///NM_009534///XM		0.008853333		-1.01	**-1.22**	-1.21	Yap1	yes-associated protein 1	0000902 // cell morphogenesis // inferred from mutant phenotype///0001570 // vasculogenesis // inferred from mutant phenotype///0003143 // embryonic heart tube morphogene
NM_177775///XM_006511221///XM		0.002958888	0.00655575	-1.14	**-1.24**	**-1.21**	Esyt3	extended synaptotagmin-like protein 3	0006810 // transport // inferred from electronic annotation///0006869 // lipid transport // inferred from electronic annotation
NM_009210///NM_14	0.00718989		0.00623551	**-1.20**	-1.15	**-1.21**	Hltf	helicase-like transcription factor	0000209 // protein polyubiquitination // inferred from electronic annotation///0006301 // postreplication repair // inferred from electronic annotation///0006351 // transcription, DI
NM_028306///XM_00	0.00314892	1.73E-04		**-1.39**	**-1.61**	-1.21	Hspa12b	heat shock protein 12B	
NM_001081225///NM	0.00284439			**1.16**	1.02	-1.21	Fam178a	family with sequence similarity 178, member A	0006281 // DNA repair // inferred from electronic annotation///0006974 // cellular response to DNA damage stimulus // not recorded///0031334 // positive regulation of protein con
NM_001013373///XM_006510132//		0.007966448	0.00191935	-1.08	**-1.18**	**-1.21**	Tmprss13	transmembrane protease, serine 13	0006508 // proteolysis // inferred from electronic annotation///0006898 // receptor-mediated endocytosis // inferred from electronic annotation///0061436 // establishment of skin
NM_015776///XM_006506364		0.008446261		-1.13	**-1.32**	-1.21	Mfap5	microfibrillar associated protein 5	0043206 // extracellular fibril organization // inferred from direct assay///0060216 // definitive hemopoiesis // inferred from mutant phenotype
NM_022004			0.00491471	-1.01	-1.17	**-1.22**	Fxyd6	FXYD domain-containing ion transport regulator 6	0006810 // transport // inferred from electronic annotation///0006811 // ion transport // inferred from electronic annotation///0034220 // ion transmembrane transport // inferred
NM_001311139///NM_021494///XM_006507463			0.00869376	-1.10	1.03	**-1.22**	Dennd5a	DENN/MADD domain containing 5A	0042147 // retrograde transport, endosome to Golgi // not recorded///0043547 // positive regulation of GTPase activity // not recorded///0050982 // detection of mechanical stimul
NM_019631///XM_006522395///XM_006522396			0.0055685	1.01	-1.17	**-1.22**	Tmem45a	transmembrane protein 45a	
NM_001081357///NM_001290345///XM_0065241			0.00810031	1.03	-1.09	**-1.22**	Map4k3	mitogen-activated protein kinase kinase kinase kinase 3	0000165 // MAPK cascade // not recorded///0000185 // activation of MAPKKK activity // ---///0000185 // activation of MAPKKK activity // inferred from electronic annotation///0006
NM_001163029///NM	0.00495731			**-1.35**	-1.23	-1.22	Adgre5	adhesion G protein-coupled receptor E5	0007155 // cell adhesion // inferred from electronic annotation///0007165 // signal transduction // inferred from electronic annotation///0007166 // cell surface receptor signaling pa
NM_026793			0.00459823	-1.19	1.04	**-1.22**	Myct1	myc target 1	
NM_010180///XM_006520437		0.002711203	0.0080773	-1.12	**-1.27**	**-1.22**	Fbln1	fibulin 1	0001933 // negative regulation of protein phosphorylation // not recorded///0007162 // negative regulation of cell adhesion // not recorded///0007566 // embryo implantation // inf
NM_001168392///NM	0.00367898			**-1.22**	-1.14	-1.22	Ramp1	receptor (calcitonin) activity modifying protein 1	0001525 // angiogenesis // not recorded///0006810 // transport // inferred from electronic annotation///0006816 // calcium ion transport // not recorded///0006886 // intracellular
NM_001130187///NM_031878///XM_006534552			0.00681193	-1.08	-1.11	**-1.22**	Smarcd2	SWI/SNF related, matrix associated, actin dependent regulator of c	0006337 // nucleosome disassembly // not recorded///0006338 // chromatin remodeling // not recorded///0006351 // transcription, DNA-templated // inferred from electronic anno
NM_019588///XM_006527394///XM		0.006063055		1.07	**-1.30**	-1.22	Plce1	phospholipase C, epsilon 1	0000187 // activation of MAPK activity // not recorded///0006629 // lipid metabolic process // inferred from electronic annotation///0007165 // signal transduction // inferred from e
NM_011594		0.004286218	0.00667731	-1.14	**-1.22**	**-1.22**	Timp2	tissue inhibitor of metalloproteinase 2	0007417 // central nervous system development // inferred from electronic annotation///0007568 // aging // inferred from electronic annotation///0008285 // negative regulation of

RefSeq Transcript ID	p (24h n=460)	p (48h n=657)	p (72h n=805)	FC 24h	FC 48h	FC 72h	Gene Symbol	Gene Title	Gene Ontology Biological Process
NM_024253	0.00251151			-1.26	-1.34	-1.22	Nkg7	natural killer cell group 7 sequence	
NM_024263///XM_006539235///XM		8.38E-04		-1.21	-1.40	-1.22	Mxra8	matrix-remodelling associated 8	0060857 // establishment of glial blood-brain barrier // inferred from expression pattern
NM_145426///NM_180599///XM_0		0.007533327		-1.09	-1.19	-1.22	Mfap3	microfibrillar-associated protein 3	
NM_001282006///NM_001282007///NM_011569//			0.00374456	1.06	-1.07	-1.23	Tekt1	tektin 1	
NM_001289916///NM_001289917///NM_013646//			0.00895491	-1.06	-1.10	-1.23	Rora	RAR-related orphan receptor alpha	0001525 // angiogenesis // not recorded///0006351 // transcription, DNA-templated // inferred from electronic annotation///0006355 // regulation of transcription, DNA-templated /
NM_013587			0.00418303	-1.10	-1.13	-1.23	Lrpap1	low density lipoprotein receptor-related protein associated protein	0010916 // negative regulation of very-low-density lipoprotein particle clearance // not recorded///0032091 // negative regulation of protein binding // not recorded///1900116 // ex
NM_010346///XM_00	0.00548415		0.00863723	-1.18	-1.10	-1.23	Grb7	growth factor receptor bound protein 7	0007165 // signal transduction // traceable author statement///0009967 // positive regulation of signal transduction // traceable author statement///0017148 // negative regulation
NM_018869///XM_006526697///XM_011247149			0.00935319	-1.16	-1.18	-1.23	Grk5	G protein-coupled receptor kinase 5	0002029 // desensitization of G-protein coupled receptor protein signaling pathway // not recorded///0006468 // protein phosphorylation // inferred from electronic annotation///00
NM_001289429///NM_001289430///NM_0012894			0.00960168	1.06	-1.05	-1.23	Cipc	CLOCK interacting protein, circadian	0006351 // transcription, DNA-templated // inferred from electronic annotation///0006355 // regulation of transcription, DNA-templated // inferred from electronic annotation///00
NM_146222///XM_006510230///XM_006510231//			0.00231715	1.00	-1.23	-1.23	Msantd2	Myb/SANT-like DNA-binding domain containing 2	
NM_008817			0.00883128	-1.14	-1.09	-1.23	Peg3	paternally expressed 3	0000122 // negative regulation of transcription from RNA polymerase II promoter // inferred from mutant phenotype///0006915 // apoptotic process // inferred from electronic anno
NM_183195		0.004056494	0.00778976	-1.14	-1.20	-1.23	Marveld1	MARVEL (membrane-associating) domain containing 1	0007049 // cell cycle // inferred from electronic annotation
NM_144892///XM_006499369///XM		0.001601422	0.00867335	-1.13	-1.31	-1.23	Ncoa5	nuclear receptor coactivator 5	0006351 // transcription, DNA-templated // inferred from electronic annotation///0006355 // regulation of transcription, DNA-templated // inferred from electronic annotation///00
NM_001045489///NM_008594			0.00502676	-1.06	-1.08	-1.23	Mfge8	milk fat globule-EGF factor 8 protein	0001525 // angiogenesis // inferred from electronic annotation///0006910 // phagocytosis, recognition // inferred from direct assay///0006911 // phagocytosis, engulfment // inferre
NM_001081098///XM	0.00465372	0.007951287	0.00725604	-1.17	-1.19	-1.23	Zfp362	zinc finger protein 362	
NM_001289603///NM_033476///XM_006520738//			0.0094712	-1.08	-1.17	-1.23	Tfcp2	transcription factor CP2	0006351 // transcription, DNA-templated // inferred from electronic annotation///0006355 // regulation of transcription, DNA-templated // inferred from electronic annotation///00
NM_010945///XM_006537662///XM_006537663//			0.00387715	-1.07	-1.14	-1.23	Nsmaf	neutral sphingomyelinase (N-SMase) activation associated factor	0016197 // endosomal transport // not recorded///0043065 // positive regulation of apoptotic process // not recorded///0043085 // positive regulation of catalytic activity // not rec
NM_001008550///XM_006515647///XM_00651564			0.00900852	-1.15	-1.16	-1.23	Zfyve26	zinc finger, FYVE domain containing 26	0000724 // double-strand break repair via homologous recombination // not recorded///0000910 // cytokinesis // not recorded///0006281 // DNA repair // inferred from electronic a
NM_026385		0.008917855		-1.11	-1.18	-1.24	Pllp	plasma membrane proteolipid	0006810 // transport // inferred from electronic annotation///0006811 // ion transport // not recorded///0009611 // response to wounding // inferred from electronic annotation///
NM_011372///XM_006501219///XM_006501220//			0.00396715	-1.01	-1.18	-1.24	St6galnac3	ST6 (alpha-N-acetyl-neuraminyl-2,3-beta-galactosyl-1,3)-N-acetylg	0001574 // ganglioside biosynthetic process // not recorded///0006486 // protein glycosylation // not recorded///0006677 // glycosylceramide metabolic process // not recorded///
NM_019439///XM_006524632///XM_006524633//			0.0085311	-1.05	-1.16	-1.24	Gabbr1	gamma-aminobutyric acid (GABA) B receptor, 1	0001649 // osteoblast differentiation // not recorded///0007165 // signal transduction // inferred from electronic annotation///0007186 // G-protein coupled receptor signaling pathw
NM_007705///XM_006513164///XM		0.008986259	0.00740127	1.13	1.28	-1.24	Cirbp	cold inducible RNA binding protein	0009409 // response to cold // inferred from electronic annotation///0009411 // response to UV // not recorded///0017148 // negative regulation of translation // inferred from dire
NM_033560///XM_011242230			0.00439555	-1.05	-1.20	-1.24	Vps37a	vacuolar protein sorting 37A (yeast)	0006810 // transport // inferred from electronic annotation///0015031 // protein transport // inferred from electronic annotation///0043162 // ubiquitin-dependent protein catabol
NM_010153	9.13E-04			-1.23	-1.14	-1.24	Erbb3	v-erb-b2 erythroblastic leukemia viral oncogene homolog 3 (avian)	0003197 // endocardial cushion development // inferred from mutant phenotype///0006468 // protein phosphorylation // inferred from electronic annotation///0007162 // negative
NM_025826			0.00694613	-1.07	-1.18	-1.24	Acadsb	acyl-Coenzyme A dehydrogenase, short/branched chain	0006629 // lipid metabolic process // inferred from electronic annotation///0006631 // fatty acid metabolic process // inferred from electronic annotation///0006637 // acyl-CoA me
NM_026388///XM_006539129///XR_		0.002427733		-1.15	-1.22	-1.24	Tprgl	transformation related protein 63 regulated like	
NM_028626///XM_006541223			0.00753021	-1.03	-1.19	-1.24	Mcee	methylmalonyl CoA epimerase	0046491 // L-methylmalonyl-CoA metabolic process // not recorded
NM_023143			0.00757755	-1.01	1.00	-1.24	C1ra	complement component 1, r subcomponent A	0002376 // immune system process // inferred from electronic annotation///0006508 // proteolysis // inferred from electronic annotation///0006955 // immune response // ---///00
NM_153538///XM_006517207///XM_006517208//			0.00838378	-1.08	-1.15	-1.24	Zcchc6	zinc finger, CCHC domain containing 6	0031123 // RNA 3'-end processing // not recorded///0071044 // histone mRNA catabolic process // inferred from sequence or structural similarity
NM_172280///XM_006507615///XM_006507616//			0.00266354	-1.06	-1.18	-1.24	Emsy	EMSY, BRCA2-interacting transcriptional repressor	0006281 // DNA repair // inferred from electronic annotation///0006351 // transcription, DNA-templated // inferred from electronic annotation///0006355 // regulation of transcript
NM_007426			0.00497664	-1.05	-1.13	-1.24	Angpt2	angiopoietin 2	0001525 // angiogenesis // inferred from genetic interaction///0001666 // response to hypoxia // inferred from electronic annotation///0001974 // blood vessel remodeling // tracea
NM_013512///XM_006525627///XM		0.003142807		-1.15	-1.27	-1.24	Epb41l4a	erythrocyte membrane protein band 4.1 like 4a	
NM_146013///XM_006514437///XM_006514438//			0.00843237	-1.03	-1.06	-1.24	Sec14l4	SEC14-like lipid binding 4	0006810 // transport // inferred from electronic annotation
NM_001205330///NM_001205331//		0.00606908	0.00546246	-1.12	-1.19	-1.24	Map4	microtubule-associated protein 4	0007052 // mitotic spindle organization // inferred from direct assay///0007052 // mitotic spindle organization // not recorded///0031175 // neuron projection development // not re
NM_016854		0.006256183	0.00695645	-1.08	-1.22	-1.24	Ppp1r3c	protein phosphatase 1, regulatory (inhibitor) subunit 3C	0005975 // carbohydrate metabolic process // inferred from electronic annotation///0005977 // glycogen metabolic process // inferred from direct assay///0005978 // glycogen bios
NM_001080742///NM_016872///XM_011241406//			0.00876302	-1.04	-1.02	-1.24	Vamp5	vesicle-associated membrane protein 5	0006887 // exocytosis // not recorded///0006906 // vesicle fusion // not recorded///0007275 // multicellular organismal development // inferred from electronic annotation///0007
NM_001276481///NM_001276482//		0.008302947	0.00782754	-1.12	-1.20	-1.24	Dag1	dystroglycan 1	0002011 // morphogenesis of an epithelial sheet // inferred from mutant phenotype///0006509 // membrane protein ectodomain proteolysis // not recorded///0006607 // NLS-beari
NM_029832///XM_006500390///XM_006500391			0.00556754	-1.12	1.06	-1.25	Ddrgk1	DDRGK domain containing 1	0033146 // regulation of intracellular estrogen receptor signaling pathway // not recorded///0034976 // response to endoplasmic reticulum stress // inferred from mutant phenotype,
NM_001163548///NM_011182///XM_011240972//			0.00844713	-1.05	-1.17	-1.25	Cyth3	cytohesin 3	0016192 // vesicle-mediated transport // ---///0032012 // regulation of ARF protein signal transduction // inferred from electronic annotation///0043547 // positive regulation of GTP
NM_080575		0.007175456		-1.11	-1.29	-1.25	Acss1	acyl-CoA synthetase short-chain family member 1	0006085 // acetyl-CoA biosynthetic process // inferred from direct assay///0006085 // acetyl-CoA biosynthetic process // not recorded///0008152 // metabolic process // inferred fro
NM_183276	0.00187781			-1.26	-1.14	-1.25	Nbeal2	neurobeachin-like 2	0007596 // blood coagulation // inferred from mutant phenotype///0016197 // endosomal transport // not recorded///0030220 // platelet formation // not recorded///0035855 // n
NM_001306184///NM	0.00330959	0.009030973		-1.27	-1.25	-1.25	Mmp11	matrix metallopeptidase 11	0006508 // proteolysis // inferred from electronic annotation///0007275 // multicellular organismal development // inferred from electronic annotation///0030198 // extracellular m
NM_175484///XM_006511094///XM		0.008865466		-1.22	-1.27	-1.25	Coro2b	coronin, actin binding protein, 2B	0030036 // actin cytoskeleton organization // not recorded
NM_175155///XM_00	0.00465316	0.003999708		-1.26	-1.34	-1.25	Sash1	SAM and SH3 domain containing 1	0000209 // protein polyubiquitination // not recorded///0010595 // positive regulation of endothelial cell migration // not recorded///0031666 // positive regulation of lipopolysacch
NM_001040085///NM_001040086///NM_0010400			0.00424702	1.03	-1.11	-1.25	Sytl2	synaptotagmin-like 2	0006886 // intracellular protein transport // inferred from electronic annotation///0006887 // exocytosis // inferred from direct assay///0006887 // exocytosis // not recorded///000
NM_025654///XM_006533945///XM_006533946//			0.00859566	-1.09	-1.20	-1.25	Rdm1	RAD52 motif 1	0006281 // DNA repair // inferred from electronic annotation///0006310 // DNA recombination // inferred from electronic annotation
NM_025781///XM_006531291			0.00662335	-1.05	-1.28	-1.25	Tmem170	transmembrane protein 170	
NM_017399	0.00361476			-1.43	-1.07	-1.25	Fabp1	fatty acid binding protein 1, liver	0002230 // positive regulation of defense response to virus by host // not recorded///0006810 // transport // inferred from electronic annotation///0008284 // positive regulation of
NM_008675///XM_006538625///XM		0.001680635		-1.08	-1.23	-1.25	Nbl1	neuroblastoma, suppression of tumorigenicity 1	0007399 // nervous system development // inferred from mutant phenotype///0030514 // negative regulation of BMP signaling pathway // inferred from direct assay///0035582 // se
NM_001302947///NM_001302950///NM_0013029			0.00343301	-1.05	-1.10	-1.25	Tle4	transducin-like enhancer of split 4, homolog of Drosophila E(spl)	0000122 // negative regulation of transcription from RNA polymerase II promoter // inferred from direct assay///0006351 // transcription, DNA-templated // inferred from electronic
NM_001311156///NM_133986///XM_006511620			0.00430669	-1.11	-1.08	-1.25	Tcta	T cell leukemia translocation altered gene	
NM_173368///NR_126509///XM_00		0.004666904	0.00675473	-1.04	-1.23	-1.25	Chd6	chromodomain helicase DNA binding protein 6	0006351 // transcription, DNA-templated // inferred from electronic annotation///0006355 // regulation of transcription, DNA-templated // inferred from electronic annotation///00
NM_009497			0.00233021	-1.06	-1.15	-1.25	Vamp2	vesicle-associated membrane protein 2	0006461 // protein complex assembly // not recorded///0006906 // vesicle fusion // not recorded///0009749 // response to glucose // not recorded///0015031 // protein transport /
NM_001163470///NM_172275///XM_006530300//			0.00554172	-1.06	-1.08	-1.25	Trafd1	TRAF type zinc finger domain containing 1	0045824 // negative regulation of innate immune response // inferred from mutant phenotype
NM_009676///XM_006495630		0.003018099		1.01	-1.37	-1.25	Aox1	aldehyde oxidase 1	0055114 // oxidation-reduction process // inferred from electronic annotation
NM_001271356///NM_001271357///NM_146018//			0.00828606	-1.08	-1.07	-1.25	Flcn	folliculin	0000122 // negative regulation of transcription from RNA polymerase II promoter // inferred from mutant phenotype///0000122 // negative regulation of transcription from RNA pol
NM_011136	0.0010147			-1.22	-1.22	-1.25	Pou2af1	POU domain, class 2, associating factor 1	0006351 // transcription, DNA-templated // inferred from electronic annotation///0006355 // regulation of transcription, DNA-templated // inferred from electronic annotation
NM_146168///XM_006505951///XM_006505952			0.00414492	-1.12	-1.21	-1.26	Vopp1	vesicular, overexpressed in cancer, prosurvival protein 1	0006351 // transcription, DNA-templated // inferred from electronic annotation///0006355 // regulation of transcription, DNA-templated // inferred from electronic annotation///00
NM_173038///XM_006510393///XM_006510394//			0.00326383	-1.07	-1.20	-1.26	Tbcel	tubulin folding cofactor E-like	
NM_133791			0.00483605	-1.07	-1.10	-1.26	Wwc2	WW, C2 and coiled-coil domain containing 2	0000122 // negative regulation of transcription from RNA polymerase II promoter // not recorded///0035331 // negative regulation of hippo signaling // not recorded///0046621 // n
NM_145569///XM_01	0.0062716		0.00773209	1.15	1.01	-1.26	Mat2a	methionine adenosyltransferase II, alpha	0006556 // S-adenosylmethionine biosynthetic process // inferred from direct assay///0006556 // S-adenosylmethionine biosynthetic process // not recorded///0006730 // one-carb
NM_008008///XM_01	0.00209192	0.001924714		-1.20	-1.25	-1.26	Fgf7	fibroblast growth factor 7	0001541 // ovarian follicle development // not recorded///0002230 // positive regulation of defense response to virus by host // not recorded///0006656 // phosphatidylcholine bios
NM_011403			0.00424793	-1.22	-1.16	-1.26	Slc4a1	solute carrier family 4 (anion exchanger), member 1	0006810 // transport // inferred from electronic annotation///0006811 // ion transport // inferred from electronic annotation///0006820 // anion transport // not recorded///0006
NM_144942///XM_006520999///XM_006521000//			0.00315991	-1.12	-1.06	-1.26	Csad	cysteine sulfinic acid decarboxylase	0019452 // L-cysteine catabolic process to taurine // traceable author statement///0019530 // taurine metabolic process // traceable author statement///0019752 // carboxylic acid
NM_001287388///NM_011527///XM		0.001406864	0.00318647	-1.08	-1.26	-1.26	Tal1	T cell acute lymphocytic leukemia 1	0000122 // negative regulation of transcription from RNA polymerase II promoter // inferred from genetic interaction///0001525 // angiogenesis // inferred from mutant phenotype/
NM_146240///XM_00	0.00431234		0.00574079	-1.18	-1.19	-1.26	Rassf9	Ras association (RalGDS/AF-6) domain family (N-terminal) membe	0007165 // signal transduction // inferred from electronic annotation///0046907 // intracellular transport // not recorded
NM_019995///XM_006501801///XM_006501802//			0.00901137	-1.13	-1.15	-1.26	Supt20	suppressor of Ty 20	0007275 // multicellular organismal development // inferred from electronic annotation///0007369 // gastrulation // inferred from electronic annotation///0035948 // positive regula
NM_001177629///NM_010345///XM		0.003401647		-1.08	-1.31	-1.26	Grb10	growth factor receptor bound protein 10	0007165 // signal transduction // traceable author statement///0008286 // insulin receptor signaling pathway // not recorded///0030178 // negative regulation of Wnt signaling path
NM_001163592///NM_173390///XM		0.006673874	0.00267564	-1.11	-1.21	-1.26	Nhsl1	NHS-like 1	
NM_025812///XM_006511368			0.00901358	-1.09	-1.16	-1.26	Hmg20a	high mobility group 20A	0000122 // negative regulation of transcription from RNA polymerase II promoter // inferred from mutant phenotype///0006338 // chromatin remodeling // not recorded///0006351
NM_001252614///NM_001252615///NM_0012526			0.00254787	-1.08	-1.18	-1.26	Fut8	fucosyltransferase 8	0006486 // protein glycosylation // inferred from electronic annotation///0006487 // protein N-linked glycosylation // not recorded///0006491 // N-glycan processing // inferred from
NM_001164198///NM_001164199///NM_0011642			0.00447339	-1.05	-1.18	-1.26	Prkacb	protein kinase, cAMP dependent, catalytic, beta	0001843 // neural tube closure // inferred from genetic interaction///0006468 // protein phosphorylation // inferred from direct assay///0006468 // protein phosphorylation // not re
NM_001159573///NM_001159574//		0.008265253	0.00349866	-1.08	-1.27	-1.26	Fip1l1	FIP1 like 1 (S. cerevisiae)	0006378 // mRNA polyadenylation // inferred by curator///0006397 // mRNA processing // inferred from electronic annotation
NM_001145813///NM_010125///XM_006498704//			0.00560126	-1.10	-1.12	-1.26	Elf5	E74-like factor 5	0001712 // ectodermal cell fate commitment // inferred from genetic interaction///0006351 // transcription, DNA-templated // inferred from electronic annotation///0006355 // reg
NM_153789	0.00417518			-1.27	-1.04	-1.26	Mylip	myosin regulatory light chain interacting protein	0006928 // movement of cell or subcellular component // not recorded///0007399 // nervous system development // not recorded///0010989 // negative regulation of low-density li
NM_025768///XM_00	0.00259014		0.00882521	-1.25	-1.18	-1.26	Grtp1	GH regulated TBC protein 1	0043547 // positive regulation of GTPase activity // inferred from electronic annotation
NM_001037937///NM_145470			0.00281473	-1.13	-1.13	-1.27	Deptor	DEP domain containing MTOR-interacting protein	0006469 // negative regulation of protein kinase activity // not recorded///0032007 // negative regulation of TOR signaling // not recorded///0035556 // intracellular signal transducti
NM_001010928///NM_201637///XM		0.007571417	0.0049451	-1.07	-1.22	-1.27	Chd8	chromodomain helicase DNA binding protein 8	0000122 // negative regulation of transcription from RNA polymerase II promoter // not recorded///0001701 // in utero embryonic development // inferred from mutant phenotype/
NM_026752///XM_00	0.00106352			-1.32	-1.17	-1.27	Zfyve21	zinc finger, FYVE domain containing 21	
NM_019412///NM_198048///XM_006539666			0.00932506	-1.11	-1.19	-1.27	Prx	periaxin	0019226 // transmission of nerve impulse // inferred from mutant phenotype///0019233 // sensory perception of pain // inferred from mutant phenotype///0032287 // peripheral ne
NM_007970///XM_006532178///XM_011248717			0.00221303	-1.11	-1.18	-1.27	Ezh1	enhancer of zeste 1 polycomb repressive complex 2 subunit	0000122 // negative regulation of transcription from RNA polymerase II promoter // inferred from mutant phenotype///0006351 // transcription, DNA-templated // inferred from ele
NM_001082552///NM_009277		0.008982391		-1.13	-1.22	-1.27	Trim21	tripartite motif-containing 21	0000209 // protein polyubiquitination // not recorded///0006513 // protein monoubiquitination // not recorded///0007049 // cell cycle // inferred from electronic annotation///001
NM_028881///XM_006502152///XM_006502153//			0.00344263	-1.05	-1.07	-1.27	Crtc2	CREB regulated transcription coactivator 2	0006094 // gluconeogenesis // inferred from direct assay///0006351 // transcription, DNA-templated // inferred from electronic annotation///0006355 // regulation of transcription,
NM_008173///XM_00	0.00475808			-1.21	-1.30	-1.27	Nr3c1	nuclear receptor subfamily 3, group C, member 1	0006111 // regulation of gluconeogenesis // inferred from mutant phenotype///0006338 // chromatin remodeling // not recorded///0006351 // transcription, DNA-templated // infer
NM_013589///XM_006515501///XM_006515502//			0.00879561	1.03	-1.06	-1.27	Ltbp2	latent transforming growth factor beta binding protein 2	0043206 // extracellular fibril organization // inferred from mutant phenotype///0043206 // extracellular fibril organization // not recorded
NM_011104///XM_006523832///XM_006523833			0.00888692	-1.09	-1.18	-1.27	Prkce	protein kinase C, epsilon	0002281 // macrophage activation involved in immune response // inferred from mutant phenotype///0002376 // immune system process // inferred from electronic annotation///0

RefSeq Transcript ID	p (24h n=460)	p (48h n=657)	p (72h n=805)	FC 24h	FC 48h	FC 72h	Gene Symbol	Gene Title	Gene Ontology Biological Process
NM_009811			0.00465104	-1.11	-1.14	**-1.27**	***Casp6***	caspase 6	0002525 // acute inflammatory response to non-antigenic stimulus // not recorded///0006508 // proteolysis // not recorded///0006508 // proteolysis // inferred from reviewed comp
NM_001290740///NM_011723///NM_030560///NN			0.00687366	-1.02	-1.08	**-1.27**	***Cwc22///Xdh***	CWC22 spliceosome-associated protein homolog (S. cerevisiae)///:	0000398 // mRNA splicing, via spliceosome // not recorded///0001933 // negative regulation of protein phosphorylation // not recorded///0001937 // negative regulation of endothe
NM_177727///XM_006500617///XM_006500618//,			0.00868671	-1.12	-1.26	**-1.27**	***Lsm14b***	LSM14 homolog B (SCD6, S. cerevisiae)	0006417 // regulation of translation // inferred from electronic annotation///0007275 // multicellular organismal development // inferred from electronic annotation
NM_008268	0.00951198	0.004995458	0.00739705	**-1.20**	**-1.24**	**-1.27**	***Hoxb5***	homeobox B5	0006351 // transcription, DNA-templated // inferred from electronic annotation///0006355 // regulation of transcription, DNA-templated // inferred from direct assay///0006366 // t
NM_001110009///NM	0.00180989			**-1.27**	-1.06	-1.27	***Apoc1***	apolipoprotein C-I	0006641 // triglyceride metabolic process // inferred from mutant phenotype///0006810 // transport // inferred from electronic annotation///0006869 // lipid transport // inferred fr
NM_008609			0.00644142	-1.11	-1.27	**-1.27**	***Mmp15***	matrix metallopeptidase 15	0006508 // proteolysis // inferred from electronic annotation///0032355 // response to estradiol // inferred from electronic annotation///0035987 // endodermal cell differentiation ,
NM_025638///XM_006533944			0.00152505	-1.08	-1.13	**-1.27**	***Gdpd1***	glycerophosphodiester phosphodiesterase domain containing 1	0006071 // glycerol metabolic process // inferred from electronic annotation///0006629 // lipid metabolic process // inferred from electronic annotation
NM_027494///XM_006530457///XM_011248240			0.00447972	-1.08	-1.12	**-1.28**	***Zcchc8***	zinc finger, CCHC domain containing 8	0006397 // mRNA processing // inferred from electronic annotation///0008380 // RNA splicing // inferred from electronic annotation
NM_001289782///NM	0.00497211	2.11E-04		**-1.21**	**-1.38**	-1.28	***Cryab***	crystallin, alpha B	0001666 // response to hypoxia // inferred from mutant phenotype///0002088 // lens development in camera-type eye // inferred from genetic interaction///0006457 // protein fold
NM_153198///NM_17	0.00377578			**-1.35**	-1.27	-1.28	***Hbp1***	high mobility group box transcription factor 1	0006351 // transcription, DNA-templated // inferred from electronic annotation///0006355 // regulation of transcription, DNA-templated // inferred from electronic annotation///00:
NM_001161763///NM_001161765///NM_010232//			0.00104899	-1.06	-1.11	**-1.28**	***Fmo5***	flavin containing monooxygenase 5	0017144 // drug metabolic process // not recorded///0055114 // oxidation-reduction process // not recorded
NM_001166597///NM	0.0052117		0.00395366	**-1.20**	-1.23	**-1.28**	***Dgkz***	diacylglycerol kinase zeta	0007205 // protein kinase C-activating G-protein coupled receptor signaling pathway // inferred from electronic annotation///0016310 // phosphorylation // not recorded///0031571
NM_001311122///NM	0.00602292		0.00578565	**-1.22**	-1.12	**-1.28**	***Lrrc16a***	leucine rich repeat containing 16A	0016477 // cell migration // not recorded///0030032 // lamellipodium assembly // not recorded///0030335 // positive regulation of cell migration // inferred from mutant phenotype
NM_011902			0.00161931	-1.03	-1.03	**-1.28**	***Tekt2***	tektin 2	0030030 // cell projection organization // inferred from electronic annotation///0030317 // sperm motility // inferred from mutant phenotype///0036159 // inner dynein arm assemb
NM_001025432///XM_006521751//,		5.33E-04	0.00129831	-1.04	**-1.34**	**-1.28**	***Crebbp***	CREB binding protein	0000122 // negative regulation of transcription from RNA polymerase II promoter // not recorded///0006351 // transcription, DNA-templated // inferred from electronic annotation/,
NM_008858///XM_006515586///XM		8.40E-05	0.00187625	-1.08	**-1.50**	**-1.28**	***Prkd1***	protein kinase D1	0001525 // angiogenesis // inferred from electronic annotation///0001938 // positive regulation of endothelial cell proliferation // not recorded///0002376 // immune system process
NM_001252653///NM_007899///XM_006500984//,			0.00565337	-1.09	-1.06	**-1.28**	***Ecm1***	extracellular matrix protein 1	0001503 // ossification // inferred from electronic annotation///0001525 // angiogenesis // inferred from electronic annotation///0001938 // positive regulation of endothelial cell pr
NM_001099319///NM_001285885//		0.006968951		-1.15	**-1.34**	-1.28	***Gm12942///Zmym6***	predicted gene 12942///zinc finger, MYM-type 6	0007010 // cytoskeleton organization // not recorded///0022604 // regulation of cell morphogenesis // not recorded
NM_001163472///NM_019823			0.00457534	-1.08	1.11	**-1.28**	***Cyp2d22***	cytochrome P450, family 2, subfamily d, polypeptide 22	0006805 // xenobiotic metabolic process // not recorded///0007565 // female pregnancy // inferred from electronic annotation///0010033 // response to organic substance // inferr
NM_025933			0.00921985	-1.02	1.04	**-1.28**	***Higd2a***	HIG1 domain family, member 2A	0006810 // transport // inferred from electronic annotation///0043066 // negative regulation of apoptotic process // not recorded///0055114 // oxidation-reduction process // inferr
NM_009780///XM_006523531///XM_006523532//,			0.00130037	-1.03	1.07	**-1.29**	***C4b///LOC102643089***	complement component 4B (Chido blood group)///complement C	0002376 // immune system process // inferred from electronic annotation///0006954 // inflammatory response // inferred from electronic annotation///0006956 // complement act
NM_001313757///NM_007397///XM_006511925//,			0.00400254	-1.07	-1.22	**-1.29**	***Acvr2b***	activin receptor IIB	0000122 // negative regulation of transcription from RNA polymerase II promoter // not recorded///0001501 // skeletal system development // inferred from genetic interaction///00
NM_001291903///NM_009623///XM_006520319			0.00774957	1.02	-1.09	**-1.29**	***Adcy8***	adenylate cyclase 8	0006171 // cAMP biosynthetic process // not recorded///0007189 // adenylate cyclase-activating G-protein coupled receptor signaling pathway // not recorded///0007193 // adenyla
NM_007881			7.89E-04	-1.03	-1.01	**-1.29**	***Atn1***	atrophin 1	0000122 // negative regulation of transcription from RNA polymerase II promoter // inferred from direct assay///0000122 // negative regulation of transcription from RNA polymeras
NM_011401///XM_006505822///XM		0.005625647	0.0021223	-1.15	**-1.26**	**-1.29**	***Slc2a3***	solute carrier family 2 (facilitated glucose transporter), member 3	0006810 // transport // inferred from electronic annotation///0008643 // carbohydrate transport // inferred from electronic annotation///0015758 // glucose transport // inferred fr
NM_001044751///NM_008288///XM_006497227//,			0.00261837	-1.04	-1.11	**-1.29**	***Hsd11b1***	hydroxysteroid 11-beta dehydrogenase 1	0006629 // lipid metabolic process // inferred from electronic annotation///0006704 // glucocorticoid biosynthetic process // not recorded///0006713 // glucocorticoid catabolic pro
NM_173744///XM_00	0.00198241		0.00917043	**-1.20**	-1.09	**-1.29**	***Tdrp***	testis development related protein	0007283 // spermatogenesis // not recorded
NM_133237			5.95E-04	-1.16	-1.30	**-1.29**	***Apcdd1***	adenomatosis polyposis coli down-regulated 1	0001942 // hair follicle development // not recorded///0016055 // Wnt signaling pathway // inferred from electronic annotation///0030178 // negative regulation of Wnt signaling pa
NM_133898///XM_00	6.10E-04	0.003046447	0.00249839	**-1.28**	**-1.34**	**-1.29**	***N4bp2l1***	NEDD4 binding protein 2-like 1	
NM_001190448///NM_016672///XM		0.001337507	0.00109191	-1.16	**-1.40**	**-1.29**	***Ddc***	dopa decarboxylase	0006520 // cellular amino acid metabolic process // inferred from electronic annotation///0007623 // circadian rhythm // inferred from electronic annotation///0009636 // response
NM_144837///XM_011244517///XR_382437			0.00165726	-1.05	-1.15	**-1.29**	***Ice1***	interactor of little elongation complex ELL subunit 1	0006351 // transcription, DNA-templated // inferred from electronic annotation///0006355 // regulation of transcription, DNA-templated // inferred from electronic annotation///00:
NM_145394		0.007113169	0.00165529	-1.18	**-1.23**	**-1.29**	***Slc44a3***	solute carrier family 44, member 3	
NM_013650	2.29E-04			**-2.98**	-1.81	-1.29	***S100a8***	S100 calcium binding protein A8 (calgranulin A)	0002376 // immune system process // inferred from electronic annotation///0002523 // leukocyte migration involved in inflammatory response // not recorded///0002526 // acute i
NM_029933///XM_00	0.00409769	0.002907983		**-1.20**	**-1.26**	-1.30	***Bcl9***	B cell CLL/lymphoma 9	0014908 // myotube differentiation involved in skeletal muscle regeneration // inferred from genetic interaction///0016055 // Wnt signaling pathway // inferred from electronic anno
NM_009626///XM_006500919///XM_006500920//,			0.00732456	1.01	-1.09	**-1.30**	***Adh7***	alcohol dehydrogenase 7 (class IV), mu or sigma polypeptide	0001523 // retinoid metabolic process // not recorded///0006067 // ethanol metabolic process // not recorded///0006068 // ethanol catabolic process // inferred from mutant phen
NM_133947///XM_00	0.00532344		0.00252134	**-1.17**	-1.10	**-1.30**	***Numa1***	nuclear mitotic apparatus protein 1	0000132 // establishment of mitotic spindle orientation // inferred from mutant phenotype///0051321 // meiotic cell cycle // inferred from electronic annotation///0060487 // lung e
NM_001162494///NM_008056///XM_006520438//,			0.00458652	-1.11	-1.22	**-1.30**	***Fzd6***	frizzled homolog 6 (Drosophila)	0001736 // establishment of planar polarity // inferred from genetic interaction///0001843 // neural tube closure // inferred from genetic interaction///0001942 // hair follicle develo
NM_080846///XM_006534395///XM_006534396//,			0.00153224	1.01	-1.17	**-1.30**	***Higd1b***	HIG1 domain family, member 1B	
NM_011894///XM_006519013///XM_006519015			0.00185491	-1.10	-1.21	**-1.30**	***Sh3bp5***	SH3-domain binding protein 5 (BTK-associated)	0006469 // negative regulation of protein kinase activity // inferred from direct assay///0035556 // intracellular signal transduction // not recorded///0061099 // negative regulation (
NM_001045525///XM_011249073			0.00533577	1.03	-1.10	**-1.30**	***Cyb5d1***	cytochrome b5 domain containing 1	
NM_030203	0.00220143		0.00423348	**-1.31**	-1.18	**-1.30**	***Tspyl4***	TSPY-like 4	0006334 // nucleosome assembly // inferred from electronic annotation
NM_001033272///NM	0.00765177		0.00329473	**-1.20**	-1.25	**-1.30**	***Spata13***	spermatogenesis associated 13	0016477 // cell migration // not recorded///0030032 // lamellipodium assembly // not recorded///0030334 // regulation of cell migration // not recorded///0035023 // regulation of
NM_008587///XM_006498860			0.00773283	1.12	-1.18	**-1.30**	***Mertk***	c-mer proto-oncogene tyrosine kinase	0001779 // natural killer cell differentiation // inferred from genetic interaction///0006468 // protein phosphorylation // not recorded///0006909 // phagocytosis // not recorded///0(
NM_001314034///NM_001314035///NM_0013140:			0.0055816	-1.04	-1.15	**-1.30**	***Dmd***	dystrophin, muscular dystrophy	0001954 // positive regulation of cell-matrix adhesion // inferred from mutant phenotype///0002027 // regulation of heart rate // inferred from mutant phenotype///0002027 // regu
NM_001197321///NM	3.25E-04	4.51E-04	0.00982258	**-1.28**	**-1.41**	**-1.30**	***Foxp1***	forkhead box P1	0000122 // negative regulation of transcription from RNA polymerase II promoter // inferred from direct assay///0000122 // negative regulation of transcription from RNA polymeras
NM_018884///XM_00	0.00525579	1.98E-04	0.00232707	**-1.23**	**-1.46**	**-1.30**	***Pdzrn3***	PDZ domain containing RING finger 3	0007528 // neuromuscular junction development // inferred from mutant phenotype///0016567 // protein ubiquitination // inferred from direct assay
NM_001159529///NM	0.00981256		0.00489347	**-1.22**	-1.13	**-1.30**	***Cox7a2l***	cytochrome c oxidase subunit VIIa polypeptide 2-like	0002082 // regulation of oxidative phosphorylation // inferred from mutant phenotype///0097250 // mitochondrial respiratory chain supercomplex assembly // inferred from direct a
NM_177242			0.00251847	-1.01	-1.06	**-1.30**	***Pptc7***	PTC7 protein phosphatase homolog (S. cerevisiae)	0006470 // protein dephosphorylation // inferred from electronic annotation///0008152 // metabolic process // inferred from electronic annotation
NM_133218///XM_006530055			0.00467643	1.03	-1.17	**-1.30**	***Zfp704***	zinc finger protein 704	
NM_001243916///NM	0.00393332			**-1.16**	-1.31	-1.30	***Trim34a///Trim34b***	tripartite motif-containing 34A///tripartite motif-containing 34B	0051607 // defense response to virus // inferred from electronic annotation
NM_054041///XM_006506564			0.00414976	-1.02	-1.27	**-1.30**	***Antxr1***	anthrax toxin receptor 1	0007165 // signal transduction // not recorded///0022414 // reproductive process // inferred from mutant phenotype///0031532 // actin cytoskeleton reorganization // not recordec
NM_178626///XM_006534320			0.00891308	-1.02	-1.18	**-1.30**	***Cdc42se2***	CDC42 small effector 2	0006909 // phagocytosis // inferred from electronic annotation///0007165 // signal transduction // not recorded///0008360 // regulation of cell shape // inferred from electronic ann
NM_026347///XM_006515175///XM_006515176//,			0.0036121	-1.20	-1.04	**-1.30**	***Iah1***	isoamyl acetate-hydrolyzing esterase 1 homolog (S. cerevisiae)	0006629 // lipid metabolic process // inferred from electronic annotation///0016042 // lipid catabolic process // inferred from electronic annotation
NM_026141///XM_006512832///XR_380072			0.00169483	-1.06	-1.15	**-1.30**	***Ppil4***	peptidylprolyl isomerase (cyclophilin)-like 4	0000413 // protein peptidyl-prolyl isomerization // not recorded///0006457 // protein folding // inferred from electronic annotation///1901407 // regulation of phosphorylation of RN
NM_001039184///XM_006529900///XM_00652990			0.00905664	1.00	-1.09	**-1.30**	***Cep350***	centrosomal protein 350	0034453 // microtubule anchoring // inferred from electronic annotation
NM_007818		0.00607943		-1.30	**-1.42**	-1.31	***Cyp3a11***	cytochrome P450, family 3, subfamily a, polypeptide 11	0055114 // oxidation-reduction process // inferred from electronic annotation
NM_001123327///XM_006500458///XM_00650045			0.00109792	-1.03	-1.24	**-1.31**	***Qser1***	glutamine and serine rich 1	
NM_009672///XM_006510778///XM		0.005097781	0.00801359	-1.12	**-1.31**	**-1.31**	***Anp32a***	acidic (leucine-rich) nuclear phosphoprotein 32 family, member A	0006351 // transcription, DNA-templated // inferred from electronic annotation///0006355 // regulation of transcription, DNA-templated // inferred from electronic annotation
NM_133985///NR_045693///XM_006511919///XM_			0.00540533	-1.04	-1.11	**-1.31**	***Oxsr1***	oxidative-stress responsive 1	0006468 // protein phosphorylation // not recorded///0006979 // response to oxidative stress // inferred from direct assay///0007346 // regulation of mitotic cell cycle // not recorde
NM_176837///XM_006512885///XM_011243213//,			0.00510432	1.04	-1.15	**-1.31**	***Arhgap18***	Rho GTPase activating protein 18	0007165 // signal transduction // inferred from electronic annotation///0007264 // small GTPase mediated signal transduction // not recorded///0008360 // regulation of cell shape /,
NM_001301298///NM	0.00865401	0.003944993	0.00187503	**-1.17**	**-1.24**	**-1.31**	***Gab1***	growth factor receptor bound protein 2-associated protein 1	0000187 // activation of MAPK activity // inferred from direct assay///0006979 // response to oxidative stress // inferred from direct assay///0007165 // signal transduction // inferre
NM_031392			0.00143825	-1.09	-1.05	**-1.31**	***Wdr6***	WD repeat domain 6	0007049 // cell cycle // inferred from electronic annotation///0007050 // cell cycle arrest // not recorded///0008285 // negative regulation of cell proliferation // not recorded///001(
NM_001293757///NM_001293758///NM_001293':			0.0030867	-1.06	-1.05	**-1.31**	***Dctn6***	dynactin 6	0007005 // mitochondrion organization // non-traceable author statement///0007052 // mitotic spindle organization // inferred from direct assay///0008610 // lipid biosynthetic pro
NM_145138///XM_006515708			0.00229541	-1.10	-1.17	**-1.31**	***Nek9***	NIMA (never in mitosis gene a)-related expressed kinase 9	0006468 // protein phosphorylation // not recorded///0007049 // cell cycle // inferred from electronic annotation///0007067 // mitotic nuclear division // inferred from electronic an
NM_001039530///XM_006522392///XM_00652239			0.0020614	-1.13	-1.13	**-1.31**	***Parp14***	poly (ADP-ribose) polymerase family, member 14	0006351 // transcription, DNA-templated // inferred from electronic annotation///0006355 // regulation of transcription, DNA-templated // inferred from electronic annotation///00(
NM_001081069			0.00255107	-1.14	-1.23	**-1.31**	***Rgs11***	regulator of G-protein signaling 11	0007186 // G-protein coupled receptor signaling pathway // traceable author statement///0009968 // negative regulation of signal transduction // inferred from electronic annotatior
NM_144551///XM_006515054///XM_006515055			0.00231598	-1.01	-1.21	**-1.31**	***Trib2***	tribbles homolog 2 (Drosophila)	0006468 // protein phosphorylation // inferred from electronic annotation///0032436 // positive regulation of proteasomal ubiquitin-dependent protein catabolic process // inferred
NM_001109690///NM_001109691///NM_138755//			0.0026076	-1.11	-1.12	**-1.31**	***Phf21a***	PHD finger protein 21A	0000122 // negative regulation of transcription from RNA polymerase II promoter // not recorded///0001967 // suckling behavior // inferred from mutant phenotype///0006351 // tr
NM_026639		6.38E-04	9.62E-04	-1.10	**-1.32**	**-1.31**	***Art4***	ADP-ribosyltransferase 4	0006471 // protein ADP-ribosylation // inferred from electronic annotation
NM_026116///XM_006531303///XM_006531304			6.10E-04	-1.01	-1.16	**-1.31**	***Bbs2***	Bardet-Biedl syndrome 2 (human)	0006810 // transport // inferred from electronic annotation///0007288 // sperm axoneme assembly // inferred from mutant phenotype///0008104 // protein localization // inferred f
NM_133911		0.002716289	0.002127	-1.10	**-1.33**	**-1.31**	***Adgra3***	adhesion G protein-coupled receptor A3	0007165 // signal transduction // inferred from electronic annotation///0007166 // cell surface receptor signaling pathway // inferred from electronic annotation///0007186 // G-prot
NM_029415///XM_006535278		0.009455711		1.00	**-1.23**	-1.31	***Slc10a6***	solute carrier family 10 (sodium/bile acid cotransporter family), me	0006810 // transport // inferred from electronic annotation///0006811 // ion transport // inferred from electronic annotation///0006814 // sodium ion transport // inferred from ele
NM_010617///XM_006516861///XM		0.002084302		-1.10	**-1.31**	-1.31	***Kif13a***	kinesin family member 13A	0000910 // cytokinesis // not recorded///0006810 // transport // inferred from electronic annotation///0006886 // intracellular protein transport // not recorded///0007018 // micro
NM_013630///XM_006523834///XM_006523835//,			0.00827088	-1.06	-1.12	**-1.31**	***Pkd1***	polycystic kidney disease 1 homolog	0001502 // cartilage condensation // inferred from mutant phenotype///0001568 // blood vessel development // inferred from mutant phenotype///0001701 // in utero embryonic d
NM_133917///NM_177582///XM_006530239			0.00785371	1.14	-1.05	**-1.32**	***Mlxip***	MLX interacting protein	0006351 // transcription, DNA-templated // inferred from electronic annotation///0006355 // regulation of transcription, DNA-templated // inferred from electronic annotation///00(
NM_001042752///NM_008684///XM_006510842//,			0.00289315	-1.03	-1.14	**-1.32**	***Neo1***	neogenin	0006355 // regulation of transcription, DNA-templated // inferred from direct assay///0007155 // cell adhesion // inferred from electronic annotation///0007520 // myoblast fusion /,
NM_001285852///NM_001285859///NM_0012858			0.00281991	-1.03	-1.15	**-1.32**	***Sgip1***	SH3-domain GRB2-like (endophilin) interacting protein 1	0002021 // response to dietary excess // inferred from sequence or structural similarity///0006897 // endocytosis // inferred from electronic annotation///0048260 // positive regulat
NM_146162///XM_006530287			0.00253994	-1.10	-1.08	**-1.32**	***Tmem119***	transmembrane protein 119	0001503 // ossification // inferred from electronic annotation///0001649 // osteoblast differentiation // inferred from direct assay///0001649 // osteoblast differentiation // inferred
NM_153533///XM_006520691///XM_006520692//,			0.00401052	-1.04	-1.04	**-1.32**	***Tns2***	tensin 2	0001822 // kidney development // inferred from mutant phenotype///0006470 // protein dephosphorylation // inferred from electronic annotation///0008285 // negative regulation
NM_028775		0.007608865	0.00415165	-1.19	**-1.30**	**-1.32**	***Cyp2s1***	cytochrome P450, family 2, subfamily s, polypeptide 1	0006805 // xenobiotic metabolic process // not recorded///0019373 // epoxygenase P450 pathway // not recorded///0042738 // exogenous drug catabolic process // not recorded//
NM_030697///XM_006525175///XM		0.00974358	0.006438	-1.17	**-1.25**	**-1.32**	***Kank3***	KN motif and ankyrin repeat domains 3	0051497 // negative regulation of stress fiber assembly // not recorded
NM_001271538///NM	0.00232998		0.0099698	**-1.29**	-1.17	**-1.32**	***Myh14***	myosin, heavy polypeptide 14	0003009 // skeletal muscle contraction // not recorded///0007519 // skeletal muscle tissue development // not recorded///0007605 // sensory perception of sound // not recorded//
NM_009378		0.003984945	0.00439428	-1.13	**-1.29**	**-1.32**	***Thbd***	thrombomodulin	0007165 // signal transduction // inferred from electronic annotation///0007565 // female pregnancy // inferred from mutant phenotype///0007596 // blood coagulation // traceable
NM_019826///XM_006499925			0.00356047	-1.09	-1.12	**-1.32**	***Ivd***	isovaleryl coenzyme A dehydrogenase	0006552 // leucine catabolic process // not recorded///0008152 // metabolic process // inferred from electronic annotation///0033539 // fatty acid beta-oxidation using acyl-CoA del
NM_015814///XM_006508013///XM		2.74E-04		-1.23	**-1.55**	-1.32	***Dkk3***	dickkopf homolog 3 (Xenopus laevis)	0007275 // multicellular organismal development // inferred from electronic annotation///0016055 // Wnt signaling pathway // inferred from electronic annotation///0030178 // neg
NM_001113569///NM_009295		0.004606957	0.00209799	-1.10	**-1.21**	**-1.32**	***Stxbp1***	syntaxin binding protein 1	0002576 // platelet degranulation // not recorded///0006810 // transport // inferred from electronic annotation///0006887 // exocytosis // inferred from mutant phenotype///0006!

RefSeq Transcript ID	p (24h n=460)	p (48h n=657)	p (72h n=805)	FC 24h	FC 48h	FC 72h	Gene Symbol	Gene Title	Gene Ontology Biological Process
NM_175518///XM_006537925///XM_011250037///			0.00170902	-1.08	-1.14	**-1.32**	***Tmem245***	transmembrane protein 245	
NM_197999///XM_006531391///XM_006531392///			4.12E-04	-1.05	-1.32	**-1.32**	***Ces2g***	carboxylesterase 2G	0008152 // metabolic process // inferred from electronic annotation
NM_001013414///NM_001291118///NM_0012911			0.00465085	-1.04	-1.18	**-1.32**	***Neurl4***	neuralized E3 ubiquitin protein ligase 4	
NM_001077361///NM_001077362///NM_0012878			0.00229826	-1.10	-1.20	**-1.32**	***Fhl1***	four and a half LIM domains 1	0003254 // regulation of membrane depolarization // not recorded///0007275 // multicellular organismal development // inferred from electronic annotation///0010972 // negative
NM_177673///XM_006538825			5.40E-04	1.02	-1.12	**-1.32**	***Cep104***	centrosomal protein 104	
NM_011663		0.002148202		-1.11	**-1.25**	-1.32	***Zrsr1***	zinc finger (CCCH type), RNA binding motif and serine/arginine rich 1	
NM_175309			0.00509452	-1.05	-1.03	**-1.32**	***Upk3b***	uroplakin 3B	0010629 // negative regulation of gene expression // not recorded///0046325 // negative regulation of glucose import // inferred from mutant phenotype
NM_010474	0.00344019		0.00668088	**-1.28**	1.15	**-1.32**	***Hs3st1***	heparan sulfate (glucosamine) 3-O-sulfotransferase 1	0008152 // metabolic process // inferred from electronic annotation
NM_031257///XM_006509227///XM		8.86E-04		-1.02	**-1.34**	-1.33	***Plekha2***	pleckstrin homology domain-containing, family A (phosphoinositid	0001954 // positive regulation of cell-matrix adhesion // not recorded
NM_025290		0.003556988		1.02	**-1.26**	-1.33	***Rsph1***	radial spoke head 1 homolog (Chlamydomonas)	0035082 // axoneme assembly // not recorded///0051321 // meiotic cell cycle // inferred from electronic annotation
NM_001199059///NM_019983///XM_006504446///			0.00801718	-1.06	-1.07	**-1.33**	***Rabgef1***	RAB guanine nucleotide exchange factor (GEF) 1	0001933 // negative regulation of protein phosphorylation // inferred from mutant phenotype///0002686 // negative regulation of leukocyte migration // inferred from mutant phenc
NM_199029///XM_01	0.00941378		0.00845424	**-1.17**	-1.15	**-1.33**	***Zfp395***	zinc finger protein 395	0006357 // regulation of transcription from RNA polymerase II promoter // not recorded
NM_001113333///NM	0.00837567	0.001752195	0.00262895	**-1.21**	**-1.38**	**-1.33**	***Cry2***	cryptochrome 2 (photolyase-like)	0000122 // negative regulation of transcription from RNA polymerase II promoter // not recorded///0006281 // DNA repair // inferred from electronic annotation///0006351 // trans
NM_053200			0.00404769	-1.07	-1.02	**-1.33**	***Ces1d***	carboxylesterase 1D	0006629 // lipid metabolic process // inferred from electronic annotation///0006637 // acyl-CoA metabolic process // traceable author statement///0009636 // response to toxic subs
NM_001037136///NM_178119			0.00357796	-1.08	-1.12	**-1.33**	***Agap1***	ArfGAP with GTPase domain, ankyrin repeat and PH domain 1	0006810 // transport // inferred from electronic annotation///0007165 // signal transduction // inferred from electronic annotation///0007264 // small GTPase mediated signal transc
NM_001305284///NM_001305286///		0.004262139		-1.06	**-1.37**	-1.33	***Mpdz***	multiple PDZ domain protein	0007155 // cell adhesion // inferred from direct assay///0042552 // myelination // not recorded
NM_001159367///NM	0.00217006			**1.28**	-1.10	-1.33	***Per1***	period circadian clock 1	0000122 // negative regulation of transcription from RNA polymerase II promoter // inferred from direct assay///0000122 // negative regulation of transcription from RNA polymeras
NM_001277970///NM	0.00481572	6.88E-04		**-1.23**	**-1.53**	-1.33	***Itga6***	integrin alpha 6	0007155 // cell adhesion // inferred from electronic annotation///0007160 // cell-matrix adhesion // inferred from mutant phenotype///0007229 // integrin-mediated signaling pathw
NM_001040187///NM_152806///NM	0.008353866		0.0039879	-1.07	**-1.26**	**-1.33**	***Ddx17***	DEAD (Asp-Glu-Ala-Asp) box polypeptide 17	0006351 // transcription, DNA-templated // inferred from electronic annotation///0006355 // regulation of transcription, DNA-templated // inferred from electronic annotation///000
NM_001005740///NM_001005748///NM_0013026			0.0024098	-1.04	-1.23	**-1.33**	***Phactr1***	phosphatase and actin regulator 1	0031032 // actomyosin structure organization // inferred from mutant phenotype///0031032 // actomyosin structure organization // not recorded///0031532 // actin cytoskeleton re
NM_009829///XM_006505459///XM_006505460			0.00369307	-1.21	-1.21	**-1.34**	***Ccnd2***	cyclin D2	0000082 // G1/S transition of mitotic cell cycle // not recorded///0001934 // positive regulation of protein phosphorylation // not recorded///0007049 // cell cycle // inferred from ele
NM_001309390///NM_028783///XM_006510644///			0.00791583	-1.08	-1.14	**-1.34**	***Robo4***	roundabout homolog 4 (Drosophila)	0001525 // angiogenesis // not recorded///0007275 // multicellular organismal development // inferred from electronic annotation///0030154 // cell differentiation // inferred from e
NM_016737		0.009858104		-1.11	**-1.56**	-1.34	***Stip1***	stress-induced phosphoprotein 1	
NM_145584	0.0020224		0.00318655	**-1.19**	-1.11	**-1.34**	***Spon1***	spondin 1, (f-spondin) extracellular matrix protein	0007155 // cell adhesion // inferred from electronic annotation
NM_001081149///XM_006509112///XM_00650911			0.00199466	-1.05	-1.19	**-1.34**	***Kat6a***	K(lysine) acetyltransferase 6A	0003007 // heart morphogenesis // inferred from genetic interaction///0003007 // heart morphogenesis // inferred from mutant phenotype///0006334 // nucleosome assembly // in
NM_001291910///NM_009624///XM		0.007432137	0.00136432	-1.03	**-1.20**	**-1.34**	***Adcy9***	adenylate cyclase 9	0006171 // cAMP biosynthetic process // inferred from direct assay///0006171 // cAMP biosynthetic process // not recorded///0006171 // cAMP biosynthetic process // traceable au
NM_001311123///NM_011643///XM_006511053///			0.00129362	-1.06	-1.15	**-1.34**	***Trpc1***	transient receptor potential cation channel, subfamily C, member 1	0006810 // transport // inferred from electronic annotation///0006811 // ion transport // inferred from electronic annotation///0006816 // calcium ion transport // inferred from ele
NM_139300///XM_006521702///XM		0.00606755	0.0050602	-1.04	**-1.36**	**-1.34**	***Mylk***	myosin, light polypeptide kinase	0002230 // positive regulation of defense response to virus by host // not recorded///0006468 // protein phosphorylation // inferred from electronic annotation///0006939 // smooth
NM_020590			0.00696564	-1.15	-1.09	**-1.34**	***Gabarapl1***	gamma-aminobutyric acid (GABA) A receptor-associated protein-lil	0000045 // autophagosome assembly // not recorded///0000422 // mitophagy // not recorded///0006914 // autophagy // inferred from electronic annotation///0044804 // nucleop
NM_001025566///NM_001271496///NM_013490///			8.30E-04	1.02	-1.07	**-1.34**	***Chka***	choline kinase alpha	0006580 // ethanolamine metabolic process // not recorded///0006629 // lipid metabolic process // inferred from electronic annotation///0006646 // phosphatidylethanolamine bios
NM_001013370///NM_001162908		9.25E-04	0.00861679	-1.08	**-1.33**	**-1.34**	***Sesn1***	sestrin 1	0051896 // regulation of protein kinase B signaling // inferred from direct assay///1901031 // regulation of response to reactive oxygen species // inferred from electronic annotation
NM_001293701///NM_027241			4.35E-04	-1.07	-1.09	**-1.35**	***Polr3gl***	polymerase (RNA) III (DNA directed) polypeptide G like	0006359 // regulation of transcription from RNA polymerase III promoter // inferred from electronic annotation///0006384 // transcription initiation from RNA polymerase III promot
NM_001253885///NM	0.00497973		0.00231874	**-1.23**	-1.15	**-1.35**	***Apbb1***	amyloid beta (A4) precursor protein-binding, family B, member 1	0001764 // neuron migration // inferred from genetic interaction///0006302 // double-strand break repair // inferred from mutant phenotype///0006302 // double-strand break repa
NM_021472///NM_201239			8.11E-04	-1.14	-1.22	**-1.35**	***Rnase4***	ribonuclease, RNase A family 4	0001525 // angiogenesis // inferred from electronic annotation///0001525 // angiogenesis // ---///0001525 // angiogenesis // inferred from sequence or structural similarity///00016
NM_145586///XM_006507682			0.00200493	-1.07	-1.12	**-1.35**	***Tmem159***	transmembrane protein 159	
NM_007409		0.001428181		-1.17	**-1.27**	-1.35	***Adh1***	alcohol dehydrogenase 1 (class I)	0001523 // retinoid metabolic process // inferred from mutant phenotype///0006068 // ethanol catabolic process // inferred from direct assay///0006068 // ethanol catabolic proces
NM_133667///XM_006532457			0.00255569	-1.15	-1.10	**-1.35**	***Pdk2***	pyruvate dehydrogenase kinase, isoenzyme 2	0005975 // carbohydrate metabolic process // inferred from electronic annotation///0006006 // glucose metabolic process // inferred from electronic annotation///0006111 // regul
NM_007382			0.0035196	-1.09	-1.05	**-1.35**	***Acadm***	acyl-Coenzyme A dehydrogenase, medium chain	0001889 // liver development // inferred from mutant phenotype///0005978 // glycogen biosynthetic process // inferred from mutant phenotype///0006082 // organic acid metaboli
NM_001033460		0.003626539	8.32E-04	-1.03	**-1.22**	**-1.35**	***Drc1***	dynein regulatory complex subunit 1	0007368 // determination of left/right symmetry // inferred from mutant phenotype///0007507 // heart development // inferred from mutant phenotype///0060285 // cilium-depen
NM_001193271///NM	3.77E-05	0.001450011	6.43E-04	**-1.43**	**-1.30**	**-1.35**	***Meis1***	Meis homeobox 1	0001525 // angiogenesis // inferred from mutant phenotype///0002089 // lens morphogenesis in camera-type eye // inferred from mutant phenotype///0006351 // transcription, DN
NM_011723///XM_011246381///NM_001290740///			0.00409524	-1.09	-1.03	**-1.35**	***Xdh///Cwc22***	xanthine dehydrogenase///CWC22 spliceosome-associated proteir	0001933 // negative regulation of protein phosphorylation // not recorded///0001937 // negative regulation of endothelial cell proliferation // not recorded///0006919 // activation c
NM_001081237///XM	0.00492063	0.007821989	0.00169184	**-1.19**	**-1.31**	**-1.35**	***Klhl42***	kelch-like 42	0000209 // protein polyubiquitination // not recorded///0007049 // cell cycle // inferred from electronic annotation///0007067 // mitotic nuclear division // inferred from electronic a
NM_138590///NM_177027///XM_006537984///XM			2.63E-04	-1.01	-1.16	**-1.35**	***Zcchc7***	zinc finger, CCHC domain containing 7	
NM_021718	0.00304273		0.00394075	**-1.31**	-1.58	**-1.35**	***Ms4a4b***	membrane-spanning 4-domains, subfamily A, member 4B	
NM_001290817///NM_009242		5.14E-04	3.54E-04	-1.15	**-1.29**	**-1.36**	***Sparc***	secreted acidic cysteine rich glycoprotein	0001503 // ossification // inferred from electronic annotation///0001937 // negative regulation of endothelial cell proliferation // not recorded///0007165 // signal transduction // inf
NM_017376///NM_153484///XM_006520744///XM			0.00724866	-1.18	-1.12	**-1.36**	***Tef***	thyrotroph embryonic factor	0006351 // transcription, DNA-templated // inferred from electronic annotation///0006355 // regulation of transcription, DNA-templated // inferred from electronic annotation///000
NM_007817			0.004945	-1.03	-1.19	**-1.36**	***Cyp2f2***	cytochrome P450, family 2, subfamily f, polypeptide 2	0006805 // xenobiotic metabolic process // not recorded///0009636 // response to toxic substance // inferred from mutant phenotype///0018931 // naphthalene metabolic process /
NM_001199141///NM_144516///XM_006516491///			4.14E-04	-1.09	-1.07	**-1.36**	***Zmynd11***	zinc finger, MYND domain containing 11	0000122 // negative regulation of transcription from RNA polymerase II promoter // not recorded///0006351 // transcription, DNA-templated // inferred from electronic annotation//
NM_008304			0.00736489	-1.08	-1.29	**-1.36**	***Sdc2***	syndecan 2	0007399 // nervous system development // inferred from electronic annotation///0016477 // cell migration // not recorded///0030154 // cell differentiation // inferred from electror
NM_009284	0.0073247			**-1.16**	-1.30	-1.36	***Stat6***	signal transducer and activator of transcription 6	0000122 // negative regulation of transcription from RNA polymerase II promoter // inferred from direct assay///0000122 // negative regulation of transcription from RNA polymeras
NM_012042///XM_00	0.00569502		0.00546097	**-1.22**	-1.19	**-1.36**	***Cul1***	cullin 1	0006511 // ubiquitin-dependent protein catabolic process // inferred by curator///0006513 // protein monoubiquitination // inferred from genetic interaction///0006915 // apoptotic
NM_028763			0.00680922	-1.09	-1.10	**-1.36**	***Cbx6***	chromobox 6	0000122 // negative regulation of transcription from RNA polymerase II promoter // not recorded///0006351 // transcription, DNA-templated // inferred from electronic annotation//
NM_001081362///XM_006504781///XM_00650478			0.00219987	-1.03	-1.09	**-1.37**	***Trrap***	transformation/transcription domain-associated protein	0006281 // DNA repair // not recorded///0006351 // transcription, DNA-templated // inferred from electronic annotation///0006355 // regulation of transcription, DNA-templated //
NM_001286468///NM	0.00212396	0.003012689	0.001005	**-1.41**	**-1.40**	**-1.37**	***Arhgap24***	Rho GTPase activating protein 24	0001525 // angiogenesis // inferred from electronic annotation///0007165 // signal transduction // inferred from electronic annotation///0007275 // multicellular organismal develop
NM_001163557///NM	0.00322478	0.002404311	0.00169824	**-1.29**	**-1.31**	**-1.37**	***Ppfibp2***	PTPRF interacting protein, binding protein 2 (liprin beta 2)	
NM_023186		0.00887344		-1.31	**-1.51**	-1.37	***Chia1***	chitinase, acidic 1	0000272 // polysaccharide catabolic process // inferred from electronic annotation///0002376 // immune system process // inferred from electronic annotation///0002532 // produc
NM_001206367///NM_001206368///NM_0012063			9.21E-04	-1.07	-1.11	**-1.37**	***Gsn***	gelsolin	0006911 // phagocytosis, engulfment // inferred from mutant phenotype///0006915 // apoptotic process // not recorded///0007568 // aging // inferred from electronic annotation//
NM_001113213///NM_001113214///NM_0011776			0.00906863	-1.11	-1.12	**-1.37**	***Odf2***	outer dense fiber of sperm tails 2	0007275 // multicellular organismal development // inferred from electronic annotation///0007283 // spermatogenesis // inferred from electronic annotation///0007286 // spermati
NM_008481///XM_006512566///XM_011243134///			9.78E-04	-1.11	-1.18	**-1.37**	***Lama2***	laminin, alpha 2	0007155 // cell adhesion // inferred from electronic annotation///0007411 // axon guidance // inferred from genetic interaction///0022011 // myelination in peripheral nervous syste
NM_001033481///XM_006526927///		6.35E-04	6.57E-04	-1.13	**-1.32**	**-1.37**	***Myrf***	myelin regulatory factor	0006351 // transcription, DNA-templated // inferred from electronic annotation///0006355 // regulation of transcription, DNA-templated // inferred from electronic annotation///000
NM_175692			0.00147914	-1.05	-1.16	**-1.37**	***Snhg11***	small nucleolar RNA host gene 11	
NM_001304744///NM_007714///XM_006532111///			0.00622726	1.00	-1.19	**-1.37**	***Clk4***	CDC like kinase 4	0006468 // protein phosphorylation // inferred from electronic annotation///0016310 // phosphorylation // inferred from electronic annotation///0018108 // peptidyl-tyrosine phosp
NM_001040111///NM_001040112///NM_027180//			0.00348318	-1.09	-1.11	**-1.37**	***Arap1***	ArfGAP with RhoGAP domain, ankyrin repeat and PH domain 1	0001921 // positive regulation of receptor recycling // not recorded///0007165 // signal transduction // inferred from electronic annotation///0007179 // transforming growth factor
NM_197986///XM_006506540///XM		0.003969641	0.00567491	-1.40	**-1.33**	**-1.37**	***Tmem140***	transmembrane protein 140	
NM_001253755///NM_172605///XM_006518909///			0.00656467	-1.13	-1.25	**-1.37**	***Tdrd3***	tudor domain containing 3	0006397 // mRNA processing // inferred from electronic annotation///0016568 // chromatin modification // inferred from electronic annotation///1903506 // regulation of nucleic ac
NM_001243132///NM	5.20E-04		0.00107842	**-1.31**	-1.25	**-1.37**	***Tspan2***	tetraspanin 2	0006954 // inflammatory response // inferred from genetic interaction///0007166 // cell surface receptor signaling pathway // not recorded///0007420 // brain development // inferr
NM_023402		0.001264852	0.00826063	-1.09	**-1.32**	**-1.38**	***Myl12b***	myosin, light chain 12B, regulatory	0008360 // regulation of cell shape // inferred from mutant phenotype///0070527 // platelet aggregation // not recorded
NM_001163748///NM_008804///XM		0.008764171	0.00108919	-1.20	**-1.21**	**-1.38**	***Pde9a***	phosphodiesterase 9A	0007165 // signal transduction // inferred from electronic annotation///0010613 // positive regulation of cardiac muscle hypertrophy // inferred from mutant phenotype///0046068 /
NM_001083929///NM_008161			0.00114975	-1.10	1.03	**-1.38**	***Gpx3***	glutathione peroxidase 3	0006749 // glutathione metabolic process // not recorded///0006979 // response to oxidative stress // inferred from electronic annotation///0042744 // hydrogen peroxide catabolic
NM_009349			0.00154834	-1.05	1.01	**-1.38**	***Inmt***	indolethylamine N-methyltransferase	0009308 // amine metabolic process // not recorded///0009636 // response to toxic substance // inferred from electronic annotation///0032259 // methylation // not recorded
NM_001145799///NM_001145801///		0.006648803	0.00180809	-1.06	**-1.54**	**-1.38**	***Ctla2a///Ctla2b***	cytotoxic T lymphocyte-associated protein 2 alpha///cytotoxic T ly	0010951 // negative regulation of endopeptidase activity // traceable author statement///0010955 // negative regulation of protein processing // inferred from mutant phenotype///0
NM_001083119///NM_011214///XM_006538666///			0.00360404	-1.04	-1.03	**-1.38**	***Ptpru***	protein tyrosine phosphatase, receptor type, U	0006470 // protein dephosphorylation // not recorded///0007155 // cell adhesion // inferred from electronic annotation///0008285 // negative regulation of cell proliferation // not r
NM_001044386///NM_011768///XM_006527965///			7.14E-04	1.02	-1.20	**-1.38**	***Zfx***	zinc finger protein X-linked	0001541 // ovarian follicle development // inferred from mutant phenotype///0006351 // transcription, DNA-templated // inferred from electronic annotation///0006355 // regulatic
NM_008613///XM_006510824///XM		0.004769136		-1.05	**-1.35**	-1.38	***Mns1***	meiosis-specific nuclear structural protein 1	0007283 // spermatogenesis // inferred from mutant phenotype///0044782 // cilium organization // inferred from mutant phenotype///0045724 // positive regulation of cilium asser
NM_008458///XM_011244002///XM_011244003			5.65E-04	-1.09	-1.17	**-1.38**	***Serpina3c***	serine (or cysteine) peptidase inhibitor, clade A, member 3C	0010466 // negative regulation of peptidase activity // inferred from electronic annotation///0010951 // negative regulation of endopeptidase activity // not recorded///0034097 // re
NM_001033301///NM	5.53E-04	0.001103284		**-1.25**	**-1.35**	-1.38	***Fhdc1***	FH2 domain containing 1	
NM_001099348///NM_001100596///NM_0011634			2.71E-04	-1.05	-1.08	**-1.38**	***Gm13305///Gm2002///Il11ra1//***	predicted gene 13305///predicted gene 2002///interleukin 11 rece	0001779 // natural killer cell differentiation // inferred from mutant phenotype///0001890 // placenta development // inferred from mutant phenotype///0008284 // positive regulati
NM_029614///XM_006508299///XM_006508302///			0.00179056	-1.21	-1.02	**-1.38**	***Prss23***	protease, serine 23	0006508 // proteolysis // inferred from electronic annotation
NM_009868///XM_006530630		4.94E-04	2.82E-04	-1.11	**-1.36**	**-1.38**	***Cdh5***	cadherin 5	0001955 // blood vessel maturation // inferred from mutant phenotype///0007043 // cell-cell junction assembly // not recorded///0007155 // cell adhesion // inferred from electroni
NM_001311107///NM_001311108///		0.005453472	0.00434354	-1.10	**-1.27**	**-1.39**	***Tmcc2***	transmembrane and coiled-coil domains 2	
NM_018755///NM_176073			8.17E-04	-1.10	-1.31	**-1.39**	***Cpq***	carboxypeptidase Q	0006508 // proteolysis // not recorded///0006590 // thyroid hormone generation // not recorded///0042246 // tissue regeneration // inferred from sequence or structural similarity/
NM_001289686///NM	0.00491132		0.00135194	**-1.28**	-1.26	**-1.39**	***Arhgef3***	Rho guanine nucleotide exchange factor (GEF) 3	0035023 // regulation of Rho protein signal transduction // inferred from electronic annotation///0035556 // intracellular signal transduction // inferred from electronic annotation///
NM_023794///XM_00	0.00132298			**-1.49**	-1.22	-1.39	***Etv5***	ets variant 5	0006355 // regulation of transcription, DNA-templated // inferred from electronic annotation///0006357 // regulation of transcription from RNA polymerase II promoter // not recorc
NM_172530		0.003634006		-1.23	**-1.44**	-1.39	***She***	src homology 2 domain-containing transforming protein E	
NM_001033759///NM_031997///XM_006527486///			0.00546646	1.09	-1.38	**-1.39**	***Tmem2***	transmembrane protein 2	0007275 // multicellular organismal development // inferred from electronic annotation
NM_144799///XM_006506225			0.00216514	-1.01	-1.15	**-1.39**	***Lmcd1***	LIM and cysteine-rich domains 1	0000122 // negative regulation of transcription from RNA polymerase II promoter // inferred from genetic interaction///0002230 // positive regulation of defense response to virus by

RefSeq Transcript ID	p (24h n=460)	p (48h n=657)	p (72h n=805)	FC 24h	FC 48h	FC 72h	Gene Symbol	Gene Title	Gene Ontology Biological Process
NM_001286728///NM	3.37E-04		0.00321229	-1.32	-1.22	-1.39	Glcci1	glucocorticoid induced transcript 1	
NM_011546///XM_006525767///XM_006525769//,			0.00624649	1.06	-1.14	-1.39	Zeb1	zinc finger E-box binding homeobox 1	0000122 // negative regulation of transcription from RNA polymerase II promoter // inferred from mutant phenotype///0000122 // negative regulation of transcription from RNA poly
NM_001013786///XM_006516710///XM_00651671			0.00163829	-1.05	-1.12	-1.39	Zscan26	zinc finger and SCAN domain containing 26	0006351 // transcription, DNA-templated // inferred from electronic annotation///0006355 // regulation of transcription, DNA-templated // not recorded
NM_145220///XM_006513508///XM_006513509//,			0.00470725	1.04	1.00	-1.39	Appl2	adaptor protein, phosphotyrosine interaction, PH domain and leuc	0007049 // cell cycle // inferred from electronic annotation///0008283 // cell proliferation // not recorded
NM_019427///XM_00	0.00872323		6.86E-04	-1.15	-1.18	-1.39	Epb41l4b	erythrocyte membrane protein band 4.1 like 4b	0010628 // positive regulation of gene expression // not recorded///0031032 // actomyosin structure organization // not recorded///0042060 // wound healing // not recorded///004
NM_010197///XM_006525641///XM		0.008898494	4.76E-04	-1.16	-1.29	-1.39	Fgf1	fibroblast growth factor 1	0001525 // angiogenesis // inferred from electronic annotation///0001759 // organ induction // inferred from direct assay///0001934 // positive regulation of protein phosphorylatio
NM_011416///NM_026003///XM_0(		0.00559034		-1.01	-1.30	-1.39	Smarca2	SWI/SNF related, matrix associated, actin dependent regulator of c	0006325 // chromatin organization // traceable author statement///0006334 // nucleosome assembly // traceable author statement///0006338 // chromatin remodeling // inferred f
NM_001311148///NM_021605///XM_006529770			0.00665594	-1.02	-1.08	-1.39	Nek7	NIMA (never in mitosis gene a)-related expressed kinase 7	0006468 // protein phosphorylation // not recorded///0016310 // phosphorylation // inferred from electronic annotation
NM_133871///XM_006502485///XM_006502486			0.00827549	-1.01	-1.56	-1.39	Ifi44	interferon-induced protein 44	
NM_001289490///NM_001289491///NM_207219//			0.00186866	-1.13	-1.25	-1.39	Oard1	O-acyl-ADP-ribose deacylase 1	0042278 // purine nucleoside metabolic process // not recorded
NM_013785			0.00107573	-1.15	-1.11	-1.40	Ip6k1	inositol hexaphosphate kinase 1	0006020 // inositol metabolic process // not recorded///0016310 // phosphorylation // inferred from electronic annotation///0032958 // inositol phosphate biosynthetic process // n
NM_212457			0.0011152	-1.15	-1.08	-1.40	Bex4	brain expressed gene 4	
NM_133738		0.00948514	6.03E-04	1.00	-1.49	-1.40	Antxr2	anthrax toxin receptor 2	0022414 // reproductive process // inferred from mutant phenotype///1901998 // toxin transport // inferred from mutant phenotype
NM_145492///XM_006525819///XM_006525820//,			0.00306098	1.02	-1.10	-1.40	Zfp521	zinc finger protein 521	0006351 // transcription, DNA-templated // inferred from electronic annotation///0006355 // regulation of transcription, DNA-templated // inferred from electronic annotation///00(
NM_173781///XM_006511736///XM_006511737			0.00656108	-1.14	-1.09	-1.40	Rab6b	RAB6B, member RAS oncogene family	0006810 // transport // inferred from electronic annotation///0006886 // intracellular protein transport // not recorded///0006890 // retrograde vesicle-mediated transport, Golgi to
NM_001310837///NM_010656///XM		0.003679679	7.98E-04	-1.11	-1.37	-1.40	Sspn	sarcospan	
NM_001199136///NM_001199137//		0.0040695	5.03E-04	-1.14	-1.29	-1.40	Macf1	microtubule-actin crosslinking factor 1	0001707 // mesoderm formation // inferred from mutant phenotype///0006620 // posttranslational protein targeting to membrane // inferred from mutant phenotype///0006928 //
NM_021883///XM_006537772///XM_006537773			1.64E-04	-1.16	-1.23	-1.40	Tmod1	tropomodulin 1	0006936 // muscle contraction // inferred from mutant phenotype///0007015 // actin filament organization // not recorded///0008344 // adult locomotory behavior // inferred from
NM_133829///NM_178081///XM_006496374///XM			0.0041381	-1.11	-1.29	-1.41	Mfsd6	major facilitator superfamily domain containing 6	0002250 // adaptive immune response // inferred from electronic annotation///0002376 // immune system process // inferred from electronic annotation///0055085 // transmembr
NM_001005510			0.0037105	-1.15	-1.08	-1.41	Syne2	spectrin repeat containing, nuclear envelope 2	0006998 // nuclear envelope organization // inferred from mutant phenotype///0007097 // nuclear migration // not recorded///0007163 // establishment or maintenance of cell pola
NM_001145799///NM_007796///NM_001145801//			0.0034878	-1.06	-1.50	-1.41	Ctla2a///Ctla2b	cytotoxic T lymphocyte-associated protein 2 alpha///cytotoxic T ly	0010955 // negative regulation of protein processing // inferred from mutant phenotype///0045589 // regulation of regulatory T cell differentiation // inferred from mutant phenotyp
NM_138756///XM_006510870///XM_006510871//,			0.00397328	-1.16	-1.29	-1.41	Slc25a36	solute carrier family 25, member 36	0000002 // mitochondrial genome maintenance // not recorded///0006810 // transport // inferred from electronic annotation///0006864 // pyrimidine nucleotide transport // not re
NM_146126			0.0041585	-1.13	-1.17	-1.41	Sord	sorbitol dehydrogenase	0006060 // sorbitol metabolic process // inferred from direct assay///0006062 // sorbitol catabolic process // not recorded///0006970 // response to osmotic stress // inferred from e
NM_001081188///XM_006512207///XM_00651220			0.00883526	-1.12	-1.24	-1.41	Exosc7	exosome component 7	0000467 // exonucleolytic trimming to generate mature 3'-end of 5.8S rRNA from tricistronic rRNA transcript (SSU-rRNA, 5.8S rRNA, LSU-rRNA) // not recorded///0006364 // rRNA pro
NM_016678///XM_006538086			0.00583015	-1.04	-1.15	-1.41	Reck	reversion-inducing-cysteine-rich protein with kazal motifs	0001955 // blood vessel maturation // inferred from genetic interaction///0001955 // blood vessel maturation // inferred from mutant phenotype///0007566 // embryo implantation
NM_025817	0.00135585	3.71E-04	0.00533326	-1.36	-1.52	-1.41	Tril	TLR4 interactor with leucine-rich repeats	0002376 // immune system process // inferred from electronic annotation///0002718 // regulation of cytokine production involved in immune response // not recorded///0006954 /,
NM_017394///XM_006540196///XM		0.001890356	0.0023255	-1.13	-1.34	-1.42	Slc7a10	solute carrier family 7 (cationic amino acid transporter, y+ system)	0003333 // amino acid transmembrane transport // not recorded///0003333 // amino acid transmembrane transport // inferred from electronic annotation///0006810 // transport /,
NM_011542///XM_006538710///XM_006538711//,			0.00128194	-1.09	-1.10	-1.42	Tcea3	transcription elongation factor A (SII), 3	0006351 // transcription, DNA-templated // inferred from electronic annotation///0006355 // regulation of transcription, DNA-templated // inferred from electronic annotation///00(
NM_001168620///NM_032003///XM		0.006367763		-1.19	-1.36	-1.42	Enpp5	ectonucleotide pyrophosphatase/phosphodiesterase 5	0007154 // cell communication // not recorded///0008152 // metabolic process // inferred from electronic annotation///0009166 // nucleotide catabolic process // not recorded
NM_001199187///NM_026937///XM		0.008646874		-1.06	-1.25	-1.42	Ascc1	activating signal cointegrator 1 complex subunit 1	0006351 // transcription, DNA-templated // inferred from electronic annotation///0006355 // regulation of transcription, DNA-templated // not recorded
NM_027144///XM_006510586///XM_011242597//,			3.40E-04	-1.08	-1.10	-1.42	Arhgef12	Rho guanine nucleotide exchange factor (GEF) 12	0007186 // G-protein coupled receptor signaling pathway // not recorded///0035023 // regulation of Rho protein signal transduction // inferred from electronic annotation///00355(
NM_001271407///NM_001271408///NM_0012714(			0.0070907	-1.14	-1.19	-1.42	Sorbs3	sorbin and SH3 domain containing 3	0000122 // negative regulation of transcription from RNA polymerase II promoter // inferred from genetic interaction///0000122 // negative regulation of transcription from RNA poly
NM_001112725///NM	6.26E-04			1.31	1.01	-1.42	Aldh3a1	aldehyde dehydrogenase family 3, subfamily A1	0001666 // response to hypoxia // not recorded///0006081 // cellular aldehyde metabolic process // inferred by curator///0006081 // cellular aldehyde metabolic process // not reco
NM_001164155///NM_013869		0.00166157	0.0015411	-1.16	-1.32	-1.42	Tnfrsf19	tumor necrosis factor receptor superfamily, member 19	0001942 // hair follicle development // inferred from genetic interaction///0043123 // positive regulation of I-kappaB kinase/NF-kappaB signaling // inferred from direct assay
NM_001004468///NM_021314///NM		0.003823771	8.26E-04	-1.15	-1.35	-1.42	Tacc2	transforming, acidic coiled-coil containing protein 2	0000226 // microtubule cytoskeleton organization // inferred from mutant phenotype///0008283 // cell proliferation // inferred from mutant phenotype///0021987 // cerebral corte:
NM_009531			0.00212137	-1.22	-1.23	-1.42	Xpc	xeroderma pigmentosum, complementation group C	0000715 // nucleotide-excision repair, DNA damage recognition // not recorded///0006281 // DNA repair // inferred from mutant phenotype///0006289 // nucleotide-excision repair
NM_007823			2.45E-04	-1.01	-1.02	-1.43	Cyp4b1	cytochrome P450, family 4, subfamily b, polypeptide 1	0006725 // cellular aromatic compound metabolic process // not recorded///0018879 // biphenyl metabolic process // not recorded///0018917 // fluorene metabolic process // not r
NM_009776///XM_006498623			0.001715	-1.11	-1.14	-1.43	Serping1	serine (or cysteine) peptidase inhibitor, clade G, member 1	0001869 // negative regulation of complement activation, lectin pathway // not recorded///0002376 // immune system process // inferred from electronic annotation///0006958 // c
NM_001159632///NM_133699///XM_006515194//,			2.11E-04	-1.05	-1.05	-1.43	Atp6v1c2	ATPase, H+ transporting, lysosomal V1 subunit C2	0006810 // transport // inferred from electronic annotation///0006811 // ion transport // inferred from electronic annotation///0015991 // ATP hydrolysis coupled proton transport ,
NM_001081039///NM_001128307///NM_0011283(			5.16E-04	-1.17	-1.17	-1.43	Dock9	dedicator of cytokinesis 9	0007264 // small GTPase mediated signal transduction // inferred from electronic annotation///0043547 // positive regulation of GTPase activity // inferred from electronic annotatio
NM_001253752///NM_001253753///NM_144842//			0.00325849	-1.10	-1.12	-1.43	Zmym5	zinc finger, MYM-type 5	0006351 // transcription, DNA-templated // inferred from electronic annotation///0006355 // regulation of transcription, DNA-templated // inferred from electronic annotation///00(
NM_009546			0.00162946	-1.08	-1.22	-1.43	Trim25	tripartite motif-containing 25	0002376 // immune system process // inferred from electronic annotation///0006513 // protein monoubiquitination // not recorded///0016567 // protein ubiquitination // not recor
NM_027707///NM_201232///XM_006519994///XM			0.00155424	-1.00	-1.14	-1.44	Nipbl	Nipped-B homolog (Drosophila)	0000122 // negative regulation of transcription from RNA polymerase II promoter // not recorded///0003007 // heart morphogenesis // not recorded///0003151 // outflow tract mor
NM_001145959///NM	0.00203915		5.24E-04	-1.25	-1.04	-1.44	Ndrg2	N-myc downstream regulated gene 2	0001818 // negative regulation of cytokine production // inferred from mutant phenotype///0007165 // signal transduction // not recorded///0007275 // multicellular organismal dev
NM_001166067///XM_006505963//,		3.96E-04	7.77E-04	-1.09	-1.31	-1.44	Slc4a5	solute carrier family 4, sodium bicarbonate cotransporter, membe	0002064 // epithelial cell development // inferred from mutant phenotype///0003014 // renal system process // inferred from mutant phenotype///0003073 // regulation of systemic
NM_007548///XM_006512502///XM_006512503//,			0.00196813	-1.17	-1.27	-1.44	Prdm1	PR domain containing 1, with ZNF domain	0000122 // negative regulation of transcription from RNA polymerase II promoter // inferred from direct assay///0000122 // negative regulation of transcription from RNA polymeras
NM_026780			0.00177863	-1.11	-1.09	-1.44	Syf2	SYF2 homolog, RNA splicing factor (S. cerevisiae)	0000398 // mRNA splicing, via spliceosome // not recorded///0001701 // in utero embryonic development // inferred from mutant phenotype///0006397 // mRNA processing // infer
NM_001004359///NM_001005385//		1.95E-04	0.00467485	-1.18	-1.69	-1.44	Gprasp1	G protein-coupled receptor associated sorting protein 1	0008333 // endosome to lysosome transport // not recorded///1990172 // G-protein coupled receptor catabolic process // not recorded
NM_030235///XM_006506790			0.00718086	1.00	-1.13	-1.45	Avl9	AVL9 homolog (S. cerevisiase)	0016477 // cell migration // not recorded
NM_019503///NM_052991///NM_052992///NM_1!			0.00394081	-1.10	-1.15	-1.45	Fxyd1	FXYD domain-containing ion transport regulator 1	0006810 // transport // inferred from electronic annotation///0006811 // ion transport // inferred from electronic annotation///0006821 // chloride transport // inferred from electro
NM_001243064///NM_007616///XM_006504974//,			8.83E-04	-1.04	-1.20	-1.45	Cav1	caveolin 1, caveolae protein	0000122 // negative regulation of transcription from RNA polymerase II promoter // inferred from direct assay///0000165 // MAPK cascade // inferred from mutant phenotype///000
NM_009978///XM_006498658///XM		0.007933252		1.01	-1.22	-1.45	Cst8	cystatin 8 (cystatin-related epididymal spermatogenic)	0010466 // negative regulation of peptidase activity // inferred from electronic annotation///0010951 // negative regulation of endopeptidase activity // inferred from electronic annc
NM_027852///XM_006506623///XM		0.002332313	7.50E-04	-1.14	-1.33	-1.45	Rarres2	retinoic acid receptor responder (tazarotene induced) 2	0001523 // retinoid metabolic process // not recorded///0001701 // in utero embryonic development // inferred from electronic annotation///0001934 // positive regulation of prote
NM_001097617///NM_144938///XM_006506365//,			0.00734369	-1.04	-1.22	-1.45	C1s1	complement component 1, s subcomponent 1	0002376 // immune system process // inferred from electronic annotation///0006508 // proteolysis // inferred from electronic annotation///0006956 // complement activation // inf
NM_181390			0.00511664	1.03	-1.17	-1.46	Mustn1	musculoskeletal, embryonic nuclear protein 1	0002062 // chondrocyte differentiation // inferred from electronic annotation///0030326 // embryonic limb morphogenesis // inferred from electronic annotation///0035988 // chor
NM_001160319			0.002522	-1.13	-1.17	-1.46	Ubr4	ubiquitin protein ligase E3 component n-recognin 4	0016567 // protein ubiquitination // inferred from electronic annotation///0042787 // protein ubiquitination involved in ubiquitin-dependent protein catabolic process // not recordec
NM_001161731///NM_007447			0.00190745	-1.12	-1.45	-1.46	Ang	angiogenin, ribonuclease, RNase A family, 5	0001525 // angiogenesis // not recorded///0001666 // response to hypoxia // not recorded///0001878 // response to yeast // inferred from direct assay///0001878 // response to ye
NM_020259///XM_00	0.00113622	0.001779717	0.00291367	-1.27	-1.38	-1.46	Hhip	Hedgehog-interacting protein	0005975 // carbohydrate metabolic process // inferred from electronic annotation///0007165 // signal transduction // inferred from direct assay///0007224 // smoothened signaling
NM_028127///XM_006515977///XM_006515978//,			0.00114941	-1.05	-1.13	-1.46	Frmd6	FERM domain containing 6	0003383 // apical constriction // inferred from genetic interaction///0032970 // regulation of actin filament-based process // inferred from genetic interaction///0034613 // cellular p
NM_001161775///NM_013607///XM_006521843//,			0.00784953	-1.01	-1.22	-1.46	Myh11	myosin, heavy polypeptide 11, smooth muscle	0006939 // smooth muscle contraction // inferred from direct assay///0006939 // smooth muscle contraction // inferred from mutant phenotype///0008152 // metabolic process // i
NM_133187///XM_006501984///XM_006501985			0.00565879	-1.05	-1.15	-1.47	Fam198b	family with sequence similarity 198, member B	
NM_011545	0.00454961			-1.50	-1.35	-1.47	Tcf21	transcription factor 21	0000122 // negative regulation of transcription from RNA polymerase II promoter // inferred from direct assay///0000122 // negative regulation of transcription from RNA polymeras
NM_025285			0.00852678	-1.21	-1.42	-1.47	Stmn2	stathmin-like 2	0007019 // microtubule depolymerization // not recorded///0007026 // negative regulation of microtubule depolymerization // not recorded///0010976 // positive regulation of neu
NM_011076///XM_006503555///XM_006503556			5.48E-04	-1.03	-1.10	-1.47	Abcb1a	ATP-binding cassette, sub-family B (MDR/TAP), member 1A	0000086 // G2/M transition of mitotic cell cycle // not recorded///0006810 // transport // inferred from electronic annotation///0006855 // drug transmembrane transport // not rec
NM_001131054///NM_013917///XM_006533515//,			0.00309395	-1.01	-1.15	-1.47	Pttg1	pituitary tumor-transforming gene 1	0001558 // regulation of cell growth // not recorded///0006281 // DNA repair // inferred from electronic annotation///0006355 // regulation of transcription, DNA-templated // not r
NM_001193666///NM_001193667///NM_0011936(			0.00298848	-1.12	-1.16	-1.48	Ccl21a///Ccl21b///Ccl21c///Gm1	chemokine (C-C motif) ligand 21A (serine)///chemokine (C-C motif	0001768 // establishment of T cell polarity // inferred from sequence or structural similarity///0001771 // immunological synapse formation // inferred from direct assay///0001954 /
NM_011125///XM_006498952			0.0073195	-1.16	-1.18	-1.48	Pltp	phospholipid transfer protein	0006629 // lipid metabolic process // inferred from electronic annotation///0006810 // transport // inferred from electronic annotation///0006869 // lipid transport // inferred from e
NM_010570			0.00392377	-1.08	-1.23	-1.48	Irs1	insulin receptor substrate 1	0002053 // positive regulation of mesenchymal cell proliferation // inferred from genetic interaction///0007165 // signal transduction // inferred from electronic annotation///00082ɛ
NM_001110211///NM_013472		0.006801735		-1.15	-1.18	-1.48	Anxa6	annexin A6	0006816 // calcium ion transport // inferred from mutant phenotype///0006937 // regulation of muscle contraction // inferred from mutant phenotype///0034220 // ion transmemb
NM_010681		0.004714686	6.19E-04	-1.11	-1.25	-1.48	Lama4	laminin, alpha 4	0001568 // blood vessel development // inferred from mutant phenotype///0007155 // cell adhesion // inferred from electronic annotation///0030155 // regulation of cell adhesion /
NM_001033208			5.25E-04	-1.16	-1.16	-1.48	Myzap	myocardial zonula adherens protein	0035556 // intracellular signal transduction // inferred from direct assay
NM_011784	0.00128085	0.005279274		-1.28	-1.65	-1.48	Aplnr	apelin receptor	0007165 // signal transduction // inferred from electronic annotation///0007186 // G-protein coupled receptor signaling pathway // inferred from electronic annotation///0007275 //
NM_009749			0.00369415	-1.09	-1.11	-1.49	Bex2	brain expressed X-linked 2	0006915 // apoptotic process // inferred from electronic annotation///0007049 // cell cycle // inferred from electronic annotation///0045944 // positive regulation of transcription fr
NM_054077///XM_006529083///XM		0.007509701		-1.18	-1.39	-1.50	Prelp	proline arginine-rich end leucine-rich repeat	0007409 // axonogenesis // ---///0007569 // cell aging // inferred from direct assay///0018146 // keratan sulfate biosynthetic process // not recorded///0042340 // keratan sulfate ca
NM_001077398///NM_001286348///NM_010698//			3.89E-04	-1.10	-1.35	-1.50	Ldb2	LIM domain binding 2	0001942 // hair follicle development // inferred from genetic interaction///0006357 // regulation of transcription from RNA polymerase II promoter // inferred from electronic annota
NM_001282000///NM	0.00703129		0.00360804	-1.29	-1.36	-1.50	Rbl2	retinoblastoma-like 2	0006351 // transcription, DNA-templated // inferred from electronic annotation///0006355 // regulation of transcription, DNA-templated // inferred from electronic annotation///00(
NM_008580///XM_00	0.00785419			-1.19	-1.25	-1.51	Map3k5	mitogen-activated protein kinase kinase kinase 5	0000165 // MAPK cascade // not recorded///0000186 // activation of MAPKK activity // not recorded///0000187 // activation of MAPK activity // inferred from direct assay///000193
NM_009150			0.00485919	-1.02	1.13	-1.51	Selenbp1	selenium binding protein 1	0006810 // transport // inferred from electronic annotation///0015031 // protein transport // inferred from electronic annotation///0050873 // brown fat cell differentiation // inferr
NM_001004150///NM	0.0045804	0.004777315	0.00600456	-1.22	-1.40	-1.51	A4galt	alpha 1,4-galactosyltransferase	0001576 // globoside biosynthetic process // inferred from direct assay///0001576 // globoside biosynthetic process // inferred from mutant phenotype///0006486 // protein glycosy
NM_010370	0.0038162	0.007069772		-1.50	-1.64	-1.51	Gzma	granzyme A	0006508 // proteolysis // inferred from electronic annotation///0006915 // apoptotic process // inferred from electronic annotation///0019835 // cytolysis // inferred from electronic
NM_053247		0.005769849	0.00175873	-1.36	-1.35	-1.51	Lyve1	lymphatic vessel endothelial hyaluronan receptor 1	0006027 // glycosaminoglycan catabolic process // inferred from direct assay///0006810 // transport // inferred from electronic annotation///0007155 // cell adhesion // inferred fro
NM_001113325///NM_001252403///NM_008165//			1.22E-04	-1.03	-1.10	-1.52	Gria1	glutamate receptor, ionotropic, AMPA1 (alpha 1)	0001919 // regulation of receptor recycling // not recorded///0006810 // transport // inferred from electronic annotation///0006811 // ion transport // inferred from electronic anno
NM_011693			0.00320531	-1.08	-1.43	-1.52	Vcam1	vascular cell adhesion molecule 1	0007155 // cell adhesion // inferred from direct assay///0007155 // cell adhesion // not recorded///0007157 // heterophilic cell-cell adhesion via plasma membrane cell adhesion mol
NM_001083312///NM	0.00410791			-1.45	-1.47	-1.52	Gbp7	guanylate binding protein 7	0008152 // metabolic process // inferred from electronic annotation///0042832 // defense response to protozoan // inferred from direct assay///0042832 // defense response to pro
NM_027045///NM_028407///XM_006519603///XM			0.0027581	-1.03	-1.30	-1.52	Ccser2	coiled-coil serine rich 2	0001578 // microtubule bundle formation // inferred from direct assay
NM_173788///XM_00	0.00333721	0.003112879	3.50E-04	-1.23	-1.28	-1.52	Npr2	natriuretic peptide receptor 2	0001503 // ossification // inferred from electronic annotation///0006182 // cGMP biosynthetic process // inferred from direct assay///0006182 // cGMP biosynthetic process // not re

RefSeq Transcript ID	p (24h n=460)	p (48h n=657)	p (72h n=805)	FC 24h	FC 48h	FC 72h	Gene Symbol	Gene Title	Gene Ontology Biological Process
NM_008306///XM_006525667///XM		9.89E-04	1.76E-04	-1.24	-1.54	-1.52	*Ndst1*	N-deacetylase/N-sulfotransferase (heparan glucosaminyl) 1	0000165 // MAPK cascade // inferred from mutant phenotype///0000271 // polysaccharide biosynthetic process // inferred from mutant phenotype///0003279 // cardiac septum dev
NM_138741			0.00143162	-1.06	-1.17	-1.52	*Sdpr*	serum deprivation response	0045944 // positive regulation of transcription from RNA polymerase II promoter // not recorded
NM_001290541///NM_001290543//		0.005963474	3.52E-04	-1.12	-1.59	-1.52	*Acer2*	alkaline ceramidase 2	0001953 // negative regulation of cell-matrix adhesion // not recorded///0006629 // lipid metabolic process // inferred from electronic annotation///0006672 // ceramide metabolic
NM_001302255///NM_001302257//		0.004129136		-1.02	-1.30	-1.52	*Pmp22*	peripheral myelin protein 22	0007049 // cell cycle // inferred from electronic annotation///0007050 // cell cycle arrest // inferred from electronic annotation///0008219 // cell death // not recorded///0008285 //
NM_021398///XM_00	0.00951896		0.00471223	-1.25	-1.13	-1.53	*Slc43a3*	solute carrier family 43, member 3	0055085 // transmembrane transport // inferred from electronic annotation
NM_026433///XM_00	0.00711232	0.001134347	0.00154138	-1.38	-1.51	-1.53	*Tmem100*	transmembrane protein 100	0001525 // angiogenesis // inferred from mutant phenotype///0001570 // vasculogenesis // inferred from mutant phenotype///0001701 // in utero embryonic development // inferre
NM_019707///XM_006530627///XM		3.36E-04	3.62E-05	-1.13	-1.36	-1.53	*Cdh13*	cadherin 13	0000278 // mitotic cell cycle // inferred from electronic annotation///0001558 // regulation of cell growth // inferred from electronic annotation///0001938 // positive regulation of e
NM_001081337///XM_006531024//		0.006770804	0.00196578	-1.08	-1.28	-1.53	*Sipa1l2*	signal-induced proliferation-associated 1 like 2	0043547 // positive regulation of GTPase activity // inferred from electronic annotation///0051056 // regulation of small GTPase mediated signal transduction // inferred from electro
NM_001109661///NM_007521///XM_006537566//			0.00289919	-1.15	-1.30	-1.54	*Bach2*	BTB and CNC homology 2	0000122 // negative regulation of transcription from RNA polymerase II promoter // inferred from direct assay///0006351 // transcription, DNA-templated // inferred from electronic
NM_001029836///NM_001287101//		0.007688779	0.00403771	-1.22	-1.48	-1.54	*Npnt*	nephronectin	0001657 // ureteric bud development // inferred from mutant phenotype///0001658 // branching involved in ureteric bud morphogenesis // inferred from mutant phenotype///0007
NM_025809			0.00619041	-1.35	-1.12	-1.54	*Clec14a*	C-type lectin domain family 14, member a	
NM_001025613///NM	0.00480264	0.004081433	1.98E-04	-1.35	-1.42	-1.54	*Otud7b*	OTU domain containing 7B	0000122 // negative regulation of transcription from RNA polymerase II promoter // inferred from direct assay///0000122 // negative regulation of transcription from RNA polymeras
NM_019740///XM_006512806		0.004526118	1.94E-04	1.02	-1.37	-1.54	*Foxo3*	forkhead box O3	0000122 // negative regulation of transcription from RNA polymerase II promoter // not recorded///0001542 // ovulation from ovarian follicle // inferred from mutant phenotype///0
NM_001042634///NM_009905///NR_027853///NR_			0.00685503	-1.05	-1.30	-1.54	*Clk1*	CDC-like kinase 1	0006468 // protein phosphorylation // inferred from electronic annotation///0016310 // phosphorylation // inferred from electronic annotation///0018105 // peptidyl-serine phosph
NM_010680///XM_00	0.00178259	3.29E-04	0.00205006	-1.33	-1.65	-1.54	*Lama3*	laminin, alpha 3	0007155 // cell adhesion // inferred from electronic annotation///0016337 // single organismal cell-cell adhesion // not recorded///0030155 // regulation of cell adhesion // inferred f
NM_010231///XM_006496662///XM_006496663			6.95E-04	-1.05	-1.16	-1.55	*Fmo1*	flavin containing monooxygenase 1	0006082 // organic acid metabolic process // not recorded///0006970 // response to osmotic stress // inferred from electronic annotation///0009404 // toxin metabolic process // no
NM_010612///XM_011240817			0.00352878	-1.03	-1.24	-1.55	*Kdr*	kinase insert domain protein receptor	0001525 // angiogenesis // inferred from electronic annotation///0001541 // ovarian follicle development // inferred from mutant phenotype///0001570 // vasculogenesis // inferred
NM_001142916///NM_011961		0.002469924	5.45E-05	-1.08	-1.55	-1.55	*Plod2*	procollagen lysine, 2-oxoglutarate 5-dioxygenase 2	0001666 // response to hypoxia // inferred from electronic annotation///0032870 // cellular response to hormone stimulus // inferred from electronic annotation///0055114 // oxida
NM_001285833///NM_001285835///NM_015760//			0.00468835	-1.28	-1.17	-1.56	*Nox4*	NADPH oxidase 4	0000902 // cell morphogenesis // inferred from mutant phenotype///0001666 // response to hypoxia // inferred from electronic annotation///0006801 // superoxide metabolic proce
NM_024288///XM_00	0.00249365		8.01E-05	-1.21	-1.34	-1.56	*Rmnd5a*	required for meiotic nuclear division 5 homolog A (S. cerevisiae)	
NM_181414///XR_38	0.00975831			-1.30	-1.30	-1.57	*Pik3c3*	phosphoinositide-3-kinase, class 3	0000045 // autophagosome assembly // not recorded///0000910 // cytokinesis // not recorded///0006468 // protein phosphorylation // inferred from mutant phenotype///0006897
NM_172479		6.31E-04	0.00349072	-1.04	-1.81	-1.57	*Slc38a5*	solute carrier family 38, member 5	0003333 // amino acid transmembrane transport // not recorded///0015804 // neutral amino acid transport // not recorded///0015816 // glycine transport // not recorded
NM_018738	0.00516403			-1.66	-1.55	-1.58	*Igtp*	interferon gamma induced GTPase	0035458 // cellular response to interferon-beta // inferred from direct assay
NM_133670///XM_006507538///XM_011241719//			0.0013462	-1.08	1.20	-1.59	*Sult1a1*	sulfotransferase family 1A, phenol-preferring, member 1	0006584 // catecholamine metabolic process // not recorded///0006629 // lipid metabolic process // inferred from electronic annotation///0006805 // xenobiotic metabolic process
NM_001122733///NM	4.47E-04	0.003857236		-1.39	-1.49	-1.59	*Kit*	kit oncogene	0000187 // activation of MAPK activity // not recorded///0001541 // ovarian follicle development // inferred from mutant phenotype///0002318 // myeloid progenitor cell differentia
NM_001081433///XM_006496070///XM_00649607			5.05E-04	-1.09	-1.24	-1.59	*Ankrd44*	ankyrin repeat domain 44	
NM_001033217///XM	0.00991441		0.00119568	-1.38	-1.29	-1.60	*Prickle1*	prickle homolog 1 (Drosophila)	0001843 // neural tube closure // not recorded///0006606 // protein import into nucleus // not recorded///0031398 // positive regulation of protein ubiquitination // not recorded///
NM_052994///XM_006514347			4.21E-05	-1.16	-1.15	-1.60	*Spock2*	sparc/osteonectin, cwcv and kazal-like domains proteoglycan 2	0007165 // signal transduction // inferred from electronic annotation///0010811 // positive regulation of cell-substrate adhesion // inferred from direct assay///0019800 // peptide cr
NM_178405			0.00288794	-1.22	-1.27	-1.60	*Atp1a2*	ATPase, Na+/K+ transporting, alpha 2 polypeptide	0001504 // neurotransmitter uptake // inferred from mutant phenotype///0002026 // regulation of the force of heart contraction // inferred from mutant phenotype///0002087 // re
NM_001001980///NM_001256122///XM_00650417			6.47E-04	-1.15	-1.32	-1.61	*Limch1*	LIM and calponin homology domains 1	0031032 // actomyosin structure organization // inferred from electronic annotation
NM_001002900///NM	7.10E-04	0.002200764	0.00108225	-1.43	-1.32	-1.61	*Higd1c///Methig1///Mettl7a1//*	HIG1 domain family, member 1C///methyltransferase hypoxia indu	0008152 // metabolic process // inferred from electronic annotation///0032259 // methylation // not recorded
NM_153145///XM_006533061///XM		0.005519043	0.00152682	-1.16	-1.42	-1.62	*Abca8a*	ATP-binding cassette, sub-family A (ABC1), member 8a	0006810 // transport // inferred from electronic annotation///0006869 // lipid transport // not recorded///0008152 // metabolic process // inferred from electronic annotation///005
NM_001172424///NM	0.00445186		1.41E-04	-1.22	-1.23	-1.63	*Dhrs3*	dehydrogenase/reductase (SDR family) member 3	0001523 // retinoid metabolic process // inferred from mutant phenotype///0003151 // outflow tract morphogenesis // inferred from mutant phenotype///0008152 // metabolic pro
NM_010260	2.75E-04	0.005782543	7.16E-05	-1.49	-1.65	-1.63	*Gbp2*	guanylate binding protein 2	0008152 // metabolic process // inferred from electronic annotation///0035458 // cellular response to interferon-beta // inferred from direct assay///0042832 // defense response to
NM_010358///XM_006501017		0.006048861	0.00528406	-1.03	-1.22	-1.63	*Gstm1*	glutathione S-transferase, mu 1	0006749 // glutathione metabolic process // not recorded///0008152 // metabolic process // inferred from electronic annotation///0035690 // cellular response to drug // inferred fr
NM_019440	0.00668344			-1.77	-1.51	-1.63	*Irgm2*	immunity-related GTPase family M member 2	0034341 // response to interferon-gamma // inferred from direct assay///0034341 // response to interferon-gamma // inferred from mutant phenotype///0042742 // defense respor
NM_001143689///NM_023124///NM_207648///XM			0.00733648	-1.46	-1.23	-1.64	*H2-Q4///H2-Q6///H2-Q8///LOC6*	histocompatibility 2, Q region locus 4///histocompatibility 2, Q reg	0002474 // antigen processing and presentation of peptide antigen via MHC class I // not recorded///0006955 // immune response // inferred from electronic annotation///0019882 /
NM_001308489///NM_010216///XM_006528700//			0.00197627	-1.04	-1.23	-1.64	*Figf*	c-fos induced growth factor	0001525 // angiogenesis // inferred from electronic annotation///0001666 // response to hypoxia // inferred from electronic annotation///0007275 // multicellular organismal develo
NM_001316728///NM	0.00180348	0.002253299		-1.28	-1.26	-1.64	*Rnf167*	ring finger protein 167	0000209 // protein polyubiquitination // not recorded///0016567 // protein ubiquitination // inferred from electronic annotation///0045786 // negative regulation of cell cycle // not
NM_177231///NM_178220///XM_006507198///XM			0.00531968	-1.21	-1.11	-1.65	*Arrb1*	arrestin, beta 1	0000187 // activation of MAPK activity // not recorded///0001933 // negative regulation of protein phosphorylation // not recorded///0001934 // positive regulation of protein phosp
NM_001284392///NM_001284394//		0.007575303	0.00727098	-1.13	-1.35	-1.66	*4-Sep*	septin 4	0007049 // cell cycle // inferred from electronic annotation///0007286 // spermatid development // inferred from mutant phenotype///0007420 // brain development // inferred fro
NM_001081346///NM_133244///XM_006513290//			0.00125755	-1.15	-1.22	-1.67	*Rtkn2*	rhotekin 2	0008284 // positive regulation of cell proliferation // inferred from direct assay///0030097 // hemopoiesis // inferred from direct assay
NM_001312918///NM_010145		1.21E-04	0.00408213	-1.13	-1.47	-1.67	*Ephx1*	epoxide hydrolase 1, microsomal	0006725 // cellular aromatic compound metabolic process // inferred from mutant phenotype///0009636 // response to toxic substance // inferred from electronic annotation///001
NM_009421///XM_00	8.30E-04	0.008350425	6.59E-04	-1.56	-1.45	-1.68	*Traf1*	TNF receptor-associated factor 1	0006915 // apoptotic process // inferred from electronic annotation///0007165 // signal transduction // inferred from electronic annotation///0042981 // regulation of apoptotic pro
NM_001161420///NM_013703///XM		0.004403921	0.00318838	-1.21	-1.36	-1.68	*Vldlr*	very low density lipoprotein receptor	0000122 // negative regulation of transcription from RNA polymerase II promoter // inferred from mutant phenotype///0001666 // response to hypoxia // inferred from electronic an
NM_019919///NM_206958///XM_006524348///XM			0.00751087	1.13	-1.21	-1.68	*Ltbp1*	latent transforming growth factor beta binding protein 1	0003281 // ventricular septum development // inferred from mutant phenotype///0007179 // transforming growth factor beta receptor signaling pathway // traceable author statem
NM_001136059///NM_009992///XM		1.94E-04		-1.02	-2.58	-1.70	*Cyp1a1*	cytochrome P450, family 1, subfamily a, polypeptide 1	0001666 // response to hypoxia // inferred from electronic annotation///0001889 // liver development // inferred from electronic annotation///0006778 // porphyrin-containing com
NM_023785		0.002648783		-1.10	-1.74	-1.70	*Ppbp*	pro-platelet basic protein	0002523 // leukocyte migration involved in inflammatory response // inferred from mutant phenotype///0002690 // positive regulation of leukocyte chemotaxis // not recorded///00
NM_029568///XM_006534422///XM		0.00233738		-1.23	-1.53	-1.75	*Mfap4*	microfibrillar-associated protein 4	0007155 // cell adhesion // inferred from electronic annotation///0009650 // UV protection // not recorded///0010712 // regulation of collagen metabolic process // not recorded///
NM_029620	0.0048443	0.001332161	0.00837292	-1.35	-1.58	-1.75	*Pcolce2*	procollagen C-endopeptidase enhancer 2	0010952 // positive regulation of peptidase activity // not recorded
NM_007876	0.00390525		4.74E-05	-1.21	-1.06	-1.75	*Dpep1*	dipeptidase 1 (renal)	0006508 // proteolysis // inferred from electronic annotation///0016999 // antibiotic metabolic process // not recorded///0030336 // negative regulation of cell migration // not reco
NM_019521			1.74E-04	-1.11	-1.29	-1.75	*Gas6*	growth arrest specific 6	0001764 // neuron migration // inferred from genetic interaction///0001934 // positive regulation of protein phosphorylation // not recorded///0001961 // positive regulation of cyto
NM_008720	0.00173486	0.008053107		-1.49	-1.69	-1.75	*Npc1*	Niemann-Pick type C1	0006486 // protein glycosylation // inferred from direct assay///0006486 // protein glycosylation // not recorded///0006629 // lipid metabolic process // inferred from electronic ann
NM_008278			5.77E-04	1.05	-1.07	-1.78	*Hpgd*	hydroxyprostaglandin dehydrogenase 15 (NAD)	0006629 // lipid metabolic process // inferred from electronic annotation///0006631 // fatty acid metabolic process // inferred from electronic annotation///0006693 // prostaglandi
NM_023755///XM_00	4.04E-04	0.001339916	2.15E-05	-1.33	-1.28	-1.79	*Tfcp2l1*	transcription factor CP2-like 1	0000122 // negative regulation of transcription from RNA polymerase II promoter // inferred from direct assay///0000902 // cell morphogenesis // inferred from mutant phenotype//
NM_008986///XM_011248817		0.006829428		-1.07	-1.32	-1.82	*Ptrf*	polymerase I and transcript release factor	0006351 // transcription, DNA-templated // inferred from electronic annotation///0006353 // DNA-templated transcription, termination // inferred from electronic annotation///000
NM_011134///XM_006505011///XM		0.004530125	8.47E-05	-1.03	-1.30	-1.82	*Pon1*	paraoxonase 1	0006470 // protein dephosphorylation // ---///0006470 // protein dephosphorylation // inferred from electronic annotation///0006629 // lipid metabolic process // inferred from elec
NM_001077364///NM_010286///NR		0.001384441	0.00122274	1.13	-1.35	-1.82	*Tsc22d3*	TSC22 domain family, member 3	0000122 // negative regulation of transcription from RNA polymerase II promoter // inferred from direct assay///0006355 // regulation of transcription, DNA-templated // inferred fro
NM_013691///XM_00	7.70E-05	8.99E-06	4.66E-05	-1.65	-2.19	-1.83	*Thbs3*	thrombospondin 3	0003417 // growth plate cartilage development // inferred from genetic interaction///0003417 // growth plate cartilage development // inferred from mutant phenotype///0007155 /
NM_008030			1.09E-04	-1.03	-1.16	-1.85	*Fmo3*	flavin containing monooxygenase 3	0017144 // drug metabolic process // not recorded///0055114 // oxidation-reduction process // not recorded
NM_009654	0.00391393	0.002279399	0.00303541	-1.67	-1.83	-1.87	*Alb*	albumin	0001895 // retina homeostasis // inferred from electronic annotation///0006810 // transport // inferred from electronic annotation///0009267 // cellular response to starvation // no
NM_023617///XM_00	0.00468122		1.80E-04	-1.25	-1.15	-1.88	*Aox3*	aldehyde oxidase 3	0055114 // oxidation-reduction process // inferred from electronic annotation
NM_020052	0.00325367	3.66E-05	1.90E-04	-1.33	-1.79	-1.92	*Scube2*	signal peptide, CUB domain, EGF-like 2	
NM_009245	0.00709003	0.007495557	0.00443722	-1.73	-1.82	-1.95	*Serpina1c*	serine (or cysteine) peptidase inhibitor, clade A, member 1C	0010466 // negative regulation of peptidase activity // inferred from electronic annotation///0010951 // negative regulation of endopeptidase activity // not recorded///0034097 // re
NM_001039181///NM	0.0011588	1.43E-04	0.00108739	-1.93	-2.82	-2.10	*Npr3*	natriuretic peptide receptor 3	0001501 // skeletal system development // inferred from direct assay///0001501 // skeletal system development // inferred from mutant phenotype///0002158 // osteoclast prolifera
NM_026481///XM_006531324		0.003304487	0.00441058	-1.28	-1.59	-2.18	*Tppp3*	tubulin polymerization-promoting protein family member 3	0001578 // microtubule bundle formation // not recorded///0046785 // microtubule polymerization // inferred from electronic annotation
NM_011458	0.00583808	0.003455335	0.00709225	-2.08	-2.18	-2.22	*Serpina3k*	serine (or cysteine) peptidase inhibitor, clade A, member 3K	0010466 // negative regulation of peptidase activity // inferred from electronic annotation///0010951 // negative regulation of endopeptidase activity // not recorded///0034097 // re
NM_008343///XM_01	0.00213245	6.28E-08	1.21E-05	-1.68	-3.30	-3.86	*Igfbp3*	insulin-like growth factor binding protein 3	0001558 // regulation of cell growth // inferred from genetic interaction///0001649 // osteoblast differentiation // inferred from expression pattern///0001933 // negative regulation
NM_001045550///NM	8.88E-04	1.92E-04	8.61E-05	-6.62	-11.08	-23.14	*Gm2083///Gm21320///Mup1//*	major urinary protein LOC100048885///predicted gene, 21320///r	0006112 // energy reserve metabolic process // inferred from sequence or structural similarity///0006112 // energy reserve metabolic process // inferred from direct assay///00068

Table S2. Top 50 chemical drugs among upstream modulators of O3-responsive transcriptome (24-72 hr) suggestive to be therapeutic targets for O3 toxicity.

Â© 2000-2017 QIAGEN. All rights reserved.

Upstream Regulator	Molecule Type	Predicted Activation State	Activation z-score	p-value of overlap	Target molecules in dataset
**calcitriol**	chemical drug	Inhibited	-4.214	1.60E-24	ABCB1,ACADSB,Acp5,ADCY9,ALB,ANGPT2,ANLN,AREG,ARHGEF3,BCL2L1,BCL3,BIRC5,BUB1B,CCL20,CCNA2,CCNE1,CD14,CDC20,CDC6,CDCA5,CDK1,CDKN1A,CDT1,CEBPB,CEBPD,CENPA,CENPQ,CES1,CHTF18,CTSK,CXCL10,CYP1A1,CYR61,DCTPP1,DUSP10,ECT2,EDN1,ERBB2,ERBB3,ETV5,F3,FASN,FDPS,FDX1,FIGNL1,FN1,GADD45G,GATA3,GLRX,GRK5,GSR,HBEGF,HK2,Hmgn2 (includes others),HPGD,ID2,IGFBP3,IL18,IL1B,IL1RN,IL6,INCENP,ITGAX,KIF20A,KIF23,KPNA2,KRT23,LCN2,LGALS1,LIG1,LPAR1,LTBP1,MAD2L1,MAL,MCM2,MCM3,MCM5,MCM7,MELK,MYC,MYD88,NCAPD2,NDC1,NOX4,NRM,NUSAP1,PBK,PC,PCNA,PDE9A,POLE,POLE2,PRC1,PTGS2,PTRF,RAB32,RACGAP1,RAD51,RRM2,S100A8,S100A9,S100G,SDSL,SERPINA1,SLC16A1,SLC25A36,SOCS3,SPP1,STMN1,TCF19,THBD,THBS1,TK1,TLR2,TMEM100,TPX2
**dexamethasone**	chemical drug		-1.744	1.65E-22	AACS,ABCB1,ACADM,ACOT7,Acp5,ACTG1,ADAMTS1,ADRB3,Akr1c14,ALB,ANGPTL4,ANXA1,APLN,AREG,ARL4C,ARRB1,ATF4,ATN1,AXIN2,B4GALT4,BCKDHA,BCL2L1,BHLHE40,BIRC3,BIRC5,C8orf4,CASP6,CAV1,CCL17,Ccl2,CCL20,CCNA2,CCND2,CCNE1,CCR1,CD74,CDKN1A,CEBPB,CEBPD,CES1,CHI3L1,CLEC7A,CNN3,CP,CRYAB,CTGF,CTSK,CUL1,CXCL10,CXCL14,CYP1A1,CYP2S1,CYP3A5,CYP51A1,CYR61,DAPK1,DCTN6,DUSP10,DUSP16,DUT,ECT2,EDN1,EGR2,EIF1AX,ELOVL1,EPB41L4B,EREG,ETV5,F3,FASN,FBLN1,FDX1,FGF7,FMO1,FN1,FOSL1,FOXO3,FOXQ1,FST,GAB1,GAL,GAS6,GATA3,GBP2,GCLC,GJA1,GPX1,GRK5,GSTM5,GZMA,HBEGF,HERPUD1,HLA-A,HMMR,HNRNPAB,HOMER2,HSD11B1,HSPH1,ID2,IFIT1B,IFRD1,IGFBP2,IGFBP3,IL18,IL1B,IL1RN,IL33,IL4R,IL6,ILF2,IRS1,IRS2,IVD,JUNB,KDR,KIF4A,KIT,KLHL24,KPNA2,KRT7,KRT8,LAMA2,LAMA3,LAMA4,LCN2,LDHA,LDLR,LGALS1,LGALS3,LPAR1,LRRC8A,LSS,LTB,LTBP2,LYVE1,MAP3K5,MARCO,MAT1A,MAT2A,MATN4,ME1,MFAP5,MFGE8,MIF,MLXIP,MMP12,MMP14,MMP2,MMP9,MS4A1,Mt1,Mt2,MVK,MYC,N4BP2L1,NFIL3,NPR2,NPR3,NR3C1,NREP,NUF2,P2RY2,P4HA2,PC,PDK2,PER1,PHLDA1,PLOD2,POU2AF1,PPA1,Ppbp,PRELP,PRSS8,PTGES,PTGS1,PTGS2,RACGAP1,RASD1,RBL2,RCAN1,RGS2,RORA,S100A8,S100A9,S100G,Saa3,SCD,SDPR,SELE,SELL,SERPINA1,SERPINA3,SERPINE1,SESN1,SLC25A5,SLC2A3,SLC40A1,SLC4A1,SMARCD2,SOCS3,SOD2,SPARC,SPOCK2,SPP1,SRF,SRM,SULF2,Sult1a1,TFPI2,TFRC,TGFBI,THBD,THBS1,TIMP1,TIMP2,TK1,TLR2,TMPRSS4,TNC,TNFAIP2,TNFRSF19,Tnfsf9,TOP2A,TRAF1,TRRAP,TSC22D3,TSPAN2,TSPYL4,UBE2C,VCAM1,VMP1,XRCC5,ZFPM1,ZNF704
**dextran sulfate**	chemical drug		0.77	9.83E-19	ADAMTS1,ADORA2B,ANLN,AURKA,BIRC5,BUB1,BUB1B,Ccl2,CCNA2,CCNF,CD14,CD74,CDC20,CDCA3,CDCA5,CDK1,CHTF18,CKS1B,CTGF,E2F8,ERCC6L,FGF1,FIGNL1,GSTM5,IFIT1B,IL18,IL1B,IL6,MCM3,MCM5,MCM6,MKI67,MMP14,MMP2,MMP9,MNS1,MYC,MYLK,NCAPD2,NDST1,NUSAP1,OSMR,PCNA,PLK1,POLE,PRC1,PTGS2,RACGAP1,RAD51,RRM2,S100A8,S100A9,Saa3,SERPINE1,SOCS3,SPC25,STMN1,THBS1,TIMP2,TOP2A,TPX2,UBE2C,UHRF1,VCAM1
**PD98059**	chemical - kinase inhibitor	Inhibited	-3.868	1.11E-17	ACOT7,ANGPT2,ANXA1,AREG,ARRB1,ATF3,ATF4,BCL2L1,BIRC5,CAV1,Ccl2,CCL20,CCNA2,CCND2,CCNE1,CCR1,CD14,CD74,CDKN1A,CEBPB,CEBPD,CLU,CTGF,CTSH,CTSK,CXCL10,CYP1A1,DHRS3,DNMT1,EDN1,EGR2,ERBB2,F3,FASN,FN1,FOSL1,FPR2,GJA1,GRIA1,HAS1,HBEGF,HNRNPAB,HPGD,IGFBP3,IL1B,IL1RN,IL6,ITGAX,ITGB1BP2,ITGB2,JUNB,KRT19,KRT8,LAMA3,LDHA,LDLR,MAT2A,MIF,MKI67,MMP12,MMP14,MMP2,MMP9,MYC,MYH11,NPC1,OSMR,PC,PCNA,PER1,PHLDA1,PLAUR,PTGES,PTGS2,RAB20,RECK,RETSAT,RNASE4,RRM2,S100A4,S100A8,SDC1,SELE,SELENBP1,SELL,SERPINA1,SERPINE1,SLC38A2,SNRPD1,SOCS3,SPP1,SPRR1A,SQLE,ST6GALNAC2,THBS1,TIMP1,TIMP2,TLR2,TOMM40,TOP2A,TRAF1,TSC22D3,VCAM1
**doxorubicin**	chemical drug		-0.398	5.60E-16	ABCB1,ADAMTS1,ADORA2B,ALB,ANXA1,AREG,ATF3,AURKA,AURKB,BCL2L1,BHLHE40,BIRC3,BIRC5,BMP4,BUB1B,Ccl2,CCNB2,CD14,CDK1,CDKN1A,CENPA,CKAP4,CLDN4,CLU,CP,CTGF,CYR61,DNMT1,EDN1,EIF5A,ELF3,ERBB2,F3,FBLN1,FOXO3,GCLC,GPX2,GSR,H2AFX,HBEGF,ID2,IL1B,KIF20A,KRT18,KRT8,LCN2,LGALS3,MAD2L1,MAP4,MCM3,MEF2C,MMP2,MYC,NFE2L2,NOX4,PCNA,PIDD1,PLK1,PRC1,PTGS2,PTTG1,Pvr,RAC2,RAD51,RBL2,RRM2,SCN3B,SDC2,SESN1,SFN,SIRT3,SOD2,SPP1,STMN1,TIMP1,TK1,TNFRSF12A,TOP2A,TUBB
**LY294002**	chemical - kinase inhibitor	Inhibited	-3.879	4.22E-15	ABCB1,ADAMTS1,ADH1C,ATF3,ATF4,AURKA,BCL2L1,BHLHE40,BIRC3,BIRC5,BUB1B,Ccl2,CCNA2,CCND2,CCNE1,CCR1,CD14,CD74,CDKN1A,CEBPB,CKS2,CLDN4,CP,CTGF,CXCL10,CYR61,EGR2,EIF1AX,ERBB3,EREG,F3,FASN,FGF7,FN1,FOSL1,GCLC,GJA1,GRB10,GSR,HBEGF,HERPUD1,HMGB2,HMGCR,HMGCS1,IGFBP2,IGFBP3,IL1B,IL1RN,IL6,IRS1,IRS2,JUNB,KLF10,LCN2,MAT2A,ME1,MEF2C,MMP14,MMP2,MMP9,MPDZ,MYC,MYH11,NFE2L2,NPTX1,PCNA,PGK1,PHLDA1,PTGS2,PTPN2,PTPRU,RBL2,RFC4,RGS2,SCD,SERPINE1,SFN,SLCO1A2,SOCS3,SPP1,SQLE,SRF,TFPI2,THBD,THBS1,TLR2,TOP2A,TRIB2,UBE2C,VCAM1,ZEB1
**trichostatin A**	chemical drug		-0.638	1.50E-14	ABCB1,ADAMTS1,ADAMTS5,ANXA6,ARL6IP1,AURKB,BCL2L1,BMP4,CAP2,Ccl2,CCNA2,CCNB2,CCND2,CD14,CD79B,CDC6,CDK1,CDKN1A,CKS1B,CLU,CREBBP,CSF3R,CTPS1,CXCL10,CYP1A1,CYP3A5,CYP4B1,DAG1,DNMT1,DUSP16,ELF3,ERBB2,F3,FASN,FBLN1,FOXF2,FUT8,GADD45G,GATA3,GCLC,GSN,GSTA3,HLA-DRA,HMGCR,HMGCS1,HMGCS2,ID2,IGFBP3,IL1B,IL6,KDR,KIF20A,KIT,LAMA2,LAMA3,LDLR,LEPR,MAP4,MAT1A,MAT2A,MCM3,MCM6,MGST3,MMP9,MVK,MYC,MYH14,NDRG2,NPC1,PCNA,PLCE1,PLK1,PRC1,PRDM1,PRSS23,PTGS1,PTGS2,RECK,Retnla,SDC1,SDC2,SELE,SIAH2,SRF,STMN1,STMN2,TFPI2,TGM1,THBS1,TIMP1,TIMP2,TK1,TLR2,TMEM245,TMPO,TNFAIP2,TOP2A,VCAM1,ZEB1
**bleomycin**	chemical drug	Activated	4.062	4.07E-14	ANGPT2,BCL2L1,CCL17,Ccl2,CCL22,CCR1,CDKN1A,CTGF,CTSK,CTSS,CXCL10,FABP1,FN1,FOSL1,FZD6,H2AFX,HBEGF,IL18,IL1B,IL4R,IL6,LMNB1,MAP4,MIF,MMP12,MMP14,MMP2,MMP9,MYC,MYCT1,NDN,PCNA,PTTG1,Retnla,S100A4,Saa3,SERPINE1,SPP1,TIMP1,TIMP2,TLR2,UBE2C,UBE2T,UHRF1,WISP1
**paclitaxel**	chemical drug		0.857	7.45E-13	ABCB1,ADORA2B,APLNR,AREG,ATF3,ATF4,BCL2L1,BHLHE40,BIRC3,BIRC5,CAV1,CDC20,CDKN1A,CLU,CRY2,CTGF,CXCL10,CYP1A1,CYP3A5,DNMT1,DUSP10,EEF1E1,EPHA2,EREG,ETV5,FMO3,FOSL1,FOXO3,H2AFX,HBEGF,HLA-A,IGFBP3,IL1B,IL6,ITGA6,KIF20A,MMP9,MYC,NREP,OSMR,PCNA,PHLDA1,PLAUR,PRELP,PTGS2,RAC2,S100A9,SLC2A3,SOD2,SPTLC2,SRF,STMN1,TFPI2,THBD,THBS1,TRAF1,TULP3,UHRF1
**ritonavir**	chemical drug		-1.114	2.17E-12	ABCB1,AXIN2,BCL2L1,BIRC5,CCND2,CRYAB,CYP1A1,CYP3A5,DHCR7,ECT2,FDPS,GBP2,HMGCR,HMMR,IFRD1,IL1B,IL6,LAMA3,LDLR,ME1,MMP9,MSMO1,MVK,MYC,NUF2,RACGAP1,SCD,SELE,SQLE,TMEM140,TMPRSS4,TSC22D3,VCAM1,XRCC5
**simvastatin**	chemical drug		-1.964	3.19E-12	ADAMTS1,ANGPT2,Anp32a,ANXA1,ANXA3,BCL2L1,BIRC5,CCL20,CCND2,CD14,CDKN1A,CTGF,CYR61,EDN1,F3,FABP1,GJA1,HIST1H1C,HMGCR,HS3ST1,IGFBP3,IL18,IL1B,IL6,ITGB2,KIT,LCN2,LDLR,MKI67,MMP2,MMP9,MPP6,MYC,PON1,PTGS2,RHOU,S100A6,SELE,SERPINE1,SOCS3,SPP1,STAT6,THBD,TIMM10,TIMP1,TLR2,VCAM1
**mibolerone**	chemical drug	Activated	4.11	3.48E-12	ALDH3A1,CD74,CDK1,CLTB,CLU,CYP51A1,DHRS3,EIF2S1,ERP29,F3,FDPS,FHL1,GADD45G,HMGCS1,IGFBP3,INCENP,ISYNA1,KLF10,LDLR,LRG1,LSS,MAL,MAT2A,MYC,NME1,NME2,PCNA,PGK1,PRELP,SERP1,SLC25A5,SQLE,SRM,STMN1,TGFBI,TK1,TOP2A,UCHL5
**gefitinib**	chemical drug		0.715	3.78E-12	ADAMTS1,AREG,AURKA,AURKB,BCL2L1,BIRC5,BUB1,Ccl2,CCNE1,CCNF,CDC6,CEBPB,CEBPD,CYP2F1,ECT2,FOSL1,FSCN1,GJA1,HSPB1,KIF23,KIT,KRT19,MCM3,MKI67,MMP11,MMP15,NOX4,OSMR,PCNA,PLAUR,PRC1,PTGS2,PTTG1,S100A14,S100A9,SLC2A3,Sult1a1,TIMP2,TLR2,TOP2A,TRAF1,VILL
**mifepristone**	chemical drug		1.135	5.37E-12	ABCB1,ANXA1,AREG,ATF3,BCL2L1,CAV1,Ccl2,CD14,CD74,CDKN1A,CEBPB,CEBPD,CHI3L1,CLU,CTGF,CYP3A5,DKK3,DNAJC2,EGR2,EPCAM,F3,FST,GAL,GAS6,GJA1,HBEGF,HPGD,HSD11B1,IGFBP3,IL1B,IL4R,IL6,IRS2,JUNB,KIT,LAMA2,LDLR,MAD2L1,MMP9,Mt1,MYC,NDN,NFE2L2,NME2,NOX4,NR3C1,PC,PCNA,PTGS1,PTGS2,RGS2,S100A6,S100G,Saa3,SERPINE1,SERPING1,SLCO1B3,SPP1,SRM,TK1
**U0126**	chemical - kinase inhibitor	Inhibited	-2.22	8.65E-12	ABCB1,ADAMTS4,ADORA2B,ANGPTL4,APLNR,AREG,ATF3,ATF4,AXIN2,BCL2L1,BHLHE40,BIRC3,BIRC5,CAV1,CCL17,Ccl2,CCND2,CCNE1,CDH5,CDKN1A,CEBPB,CEBPD,CLDN4,CRY2,CTGF,CXCL10,CYP1A1,DBP,DUSP10,EDN1,EEF1E1,EIF1AX,EPHA2,EREG,ETV5,F3,FMO3,FN1,FOSL1,GCLC,GJA1,GRIA1,GSN,HBEGF,HERPUD1,HPGD,IL1B,IL6,ITGA6,JUNB,KDR,KLF10,LDLR,MAT2A,MMP12,MMP2,MMP9,MYC,NREP,OSMR,PER1,PHLDA1,PLAUR,PRELP,PTGS2,RAC2,S100A9,SERPINE1,SLC2A3,SOCS3,SPP1,SPRR1A,TFPI2,THBS1,TIMP2,TLR2,TRAF1,TSC22D3,TULP3,VCAM1,WISP1
**decitabine**	chemical drug		-0.508	1.25E-11	ABCB1,ANGPTL4,BIRC3,BMP4,CAV1,Ccl2,CCNA2,CCND2,CD79B,CDH13,CDK1,CDKN1A,CHI3L1,CLU,CTGF,CXCL10,CYP1A1,CYP3A5,CYP4B1,DAPK1,DNMT1,FN1,FOXF2,GADD45G,GLRX,GPX1,GSN,HBEGF,HK2,HMGCS2,HMMR,HPGD,HSPA1A/HSPA1B,IGFBP2,IGFBP3,IL1B,IL6,JUNB,KRT18,KRT7,KRT8,LAMA2,LAMA3,LDLR,LEPR,LGALS3,MAT1A,MGST3,MKI67,MMP14,MMP2,MMP9,MYC,NME1,NPTX1,NR3C1,PCNA,PDE9A,PLCE1,PTGS2,RECK,RNASE4,RRAD,SELL,SERPINH1,SFN,SLC2A3,SLCO1B3,SMARCA2,SOCS3,SPARC,SPP1,TFPI2,TFRC,TGFBI,THBD,THBS1,TIMP2,TOP2A
**dihydrotestosterone**	chemical - endogenous mammalian		1.248	3.96E-11	Acp5,ADAMTS1,ADH1C,ADRB3,AREG,AXIN2,BCL2L1,BUB1B,CCNA2,CCND2,CCNE1,CD74,CDC20,CDC6,CDKN1A,CHI3L1,CLU,CST8,CTGF,CYP4B1,DCTPP1,E2F8,EPB41L4B,ERBB2,FASN,FGF7,FN1,Foxp1,FOXQ1,FST,FZD6,GAS6,GPX3,GRWD1,GSN,HK2,HLA-A,HMGCR,HOMER2,HOXA5,HPGD,HSPA1A/HSPA1B,HSPB1,ID2,IGFBP2,IGFBP3,IL6,IRS2,KRT19,LDHA,LDLR,LGALS1,LTBP1,MAFF,MCM5,ME1,MMP2,Mt1,MYC,MYLK,NR3C1,PC,PDE9A,PER1,PFN1,PHF21A,PHLDA1,PTGER4,PTGS2,RARRES2,RASSF9,SCD,SELENBP1,SLC1A3,SLC2A3,SPARC,SPP1,SQLE,STXBP1,TACC2,TALDO1,THBS1,TKT,VCAM1,VLDLR,ZEB1
**3-deoxy-2-octulosonic acid(2)-[illegible]**	chemical - endogenous non-[illegible]		1.387	4.09E-11	Ccl2,CH25H,CXCL10,DHCR7,HMGCR,IFIT1B,IL1B,IL6,LSS,PTGES,PTGS1,PTGS2,SQLE
**PPARA**	ligand-dependent nuclear receptor		-0.867	4.96E-11	ACADM,ADRB3,ANGPTL4,AURKA,BCL2L1,C1R,C1S,Ccl2,CCNA2,CCNE1,CDK1,CDKN1A,CEBPB,CEBPD,CHKA,CSAD,CYP1A1,CYP51A1,CYP7B1,DHCR7,EDN1,FABP1,FASN,FDPS,H2AFX,HIST1H1C,HMGCR,HMGCS1,HMGCS2,Hmgn2 (includes others),IGFBP2,IGFBP3,IL1RN,IL6,KIF20A,KIF4A,KRT23,KRT8,LGALS4,LSS,MAD2L1,MAT2A,MGST3,MKI67,MMP9,MSMO1,MVD,MVK,MYC,NFE2L2,NR1D1,PC,PCNA,PHLDA1,PLA2G7,PLK1,PLTP,PMVK,PON1,PPA1,PRC1,PTGS2,RETSAT,RNF186,SCD,SELENBP1,SORD,SQLE,SRM,TALDO1,TLR2,TOP2A,UCHL5,UQCRC1,VCAM1

Upstream Regulator	Molecule Type	Predicted Activation State	Activation z-score	p-value of overlap	Target molecules in dataset
**phorbol myristate acetate**	chemical drug	Activated	2.671	5.28E-11	ABCB1,ACO1,ADAMTS1,ADAMTS4,ADRB3,AKR1B10,ALB,ANGPT2,ANGPTL4,ANXA1,AREG,AURKA,AURKB,BCL2L1,BIRC3,BIRC5,CAPG,CAV1,Ccl2,CCL20,CCNA2,CCND2,CCNE1,CCNH,CD14,CD68,CDK1,CDKN1A,CES1,CH25H,CMPK1,CREBBP,CTGF,CXCL10,CYP1A1,CYR61,DBP,DNMT1,ECM1,EGR2,EIF5A,EPHA2,ERBB2,ERCC6L,F3,FCER1G,FDX1,FGF7,FOSL1,FST,GAL,GATA3,GCLC,GJA1,GRK5,HAS1,HBEGF,HLA-DRA,HOXA5,HSD11B1,ID2,IGFBP2,IGFBP3,IL18,IL1B,IL1RN,IL6,ITGAX,ITGB2,JUNB,KIT,LCN2,LDLR,LEPR,LTB,LYVE1,MMP11,MMP12,MMP14,MMP2,MMP9,MRPL12,MYC,NFIL3,NFKBIE,NME2,NPR3,P2RY2,PCNA,PER1,PKM,PLAUR,PON1,POU2AF1,PRKCE,PRKD1,PTGER4,PTGES,PTGS1,PTGS2,RANBP1,RCAN1,Retnla,RGS2,S100A14,S100A8,S100A9,SDC1,SELE,SELL,SERPINA1,SERPINA3,SERPINE1,SLC16A1,SOCS3,SOD2,SPARC,SPP1,SPRR1A,TEF,TEKT1,TFPI2,TGM1,THBS1,TIMP1,TK1,TLR2,TNFRSF12A,TRAF1,TRPC1,TSC22D3,TSHB,VCAM1,VLDLR,ZEB1
**fulvestrant**	chemical drug	Inhibited	-2.716	6.31E-11	ADCY9,AREG,ATF4,AURKA,BIRC5,BMP4,CAV1,Ccl2,CCNA2,CCNE1,CD74,CDC20,CDK1,CDKN1A,CENPF,CHTF18,CKS1B,CLK1,CXCL10,CYR61,ERBB3,GAL,GJA1,H2AFX,ID2,IL1B,IRS1,KLF10,LDLR,LEPR,MCM2,MCM3,MCM5,MCM7,MYC,NME1,PCNA,POLE,PRSS23,PTGS2,PTTG1,RBL2,RFC5,S100G,SIAH2,SLC25A5,STMN1,TFRC,TK1,TSC22D3,UBE2C,VCAM1,ZEB1,ZFX
**indomethacin**	chemical drug		0.906	7.59E-11	ADAMTS1,ADAMTS5,ANGPT2,ANXA1,BCL2L1,BIRC3,BIRC5,Ccl2,CD14,CDKN1A,CEBPB,CLDN4,CLU,CP,DDC,ELF3,FASN,FCGR2A,FGF7,GPX2,HAS1,IL1B,IL6,KRT8,LCN2,LDLR,LGALS3,MCM3,MMP12,MMP14,MMP2,MYC,PCNA,PLAUR,Ppbp,PTGER4,PTGES,PTGS1,PTGS2,Pvr,S100A8,SDC1,SELL,SPP1,TFPI2,TIMP1,TNFRSF12A,TUBB,XRCC5
**nitrofurantoin**	chemical drug	Activated	2.152	8.19E-11	ACADM,ACTG1,ADAMTS1,ALB,BCL2L1,CASP6,CES1,CLU,CP,CTSS,CXCL10,ERBB2,FABP1,FETUB,FGF7,GPX2,GSR,HMGCR,HMGCS1,HMGCS2,IFRD1,IGFBP3,IL1B,IL6,ITIH4,KRT19,LGALS3,MMP12,MMP14,Mt1,MYC,PCNA,SDC1,SELENBP1,SERPINA1,SPP1,STMN1,TIMP1,TNFRSF12A,TUBB,VCAM1
**gentamicin**	chemical drug	Activated	4.668	8.94E-11	ACADM,ACADSB,ASNSD1,ATF3,Ccl2,CDC34,CEBPB,CHKA,CHORDC1,CIPC,CLU,CPQ,CSRP2,CXCL10,CYP1A1,DDC,DDX39A,DMD,DPEP1,FMO1,GNL3,GSTM5,H2AFX,HSPA1A/HSPA1B,HSPB1,IFRD1,IGFBP3,IL1B,JUNB,KTI12,LCN2,MCM6,MYBBP1A,MYC,NCL,NME1,NOP58,NR1D1,PC,PLLP,PSAT1,Pvr,RPS27L,S100G,SPP1,ST6GALNAC3,Sult1a1,TFRC,TIMP1,TUBB,VCAM1
**medroxyprogesterone acetate**	chemical drug	Inhibited	-2.697	2.80E-10	ANGPT2,ANLN,ASPM,BCL2L1,BIRC5,Ccl2,CCNB2,CDK1,CENPF,CXCL10,CYP1A1,CYR61,ERBB2,F3,FASN,FN1,GCH1,HSD11B1,HTRA1,IL1B,IL6,ITGB2,JUNB,MCM5,MYC,MYLK,NUSAP1,PBK,PRC1,SCD,SPC25,TFPI2,TNC
**acetaminophen**	chemical drug	Activated	2.716	4.16E-10	ABCB1,ACADM,ACO1,ACTG1,ALB,BCKDHA,Ccl2,CCR1,CDKN1A,CES1,CXCL10,CYP3A5,FABP1,GPX1,GSR,HMGCR,HMGCS1,HMGCS2,IFRD1,IGFBP3,IL18,IL1B,IL6,IRS1,KDR,ME1,Mt1,MYC,NDRG2,PCNA,PLAUR,PTGS2,Retnla,SOCS3,TUBB,VCAM1
**methylselenic acid**	chemical reagent			5.73E-10	ADAMTS1,ATF3,AURKA,BCL2L1,BIRC3,BIRC5,BMP4,BUB1B,CCNA2,CCNB2,CDC20,CDC6,CDK1,CDKN1A,CENPA,CENPF,CLU,CORO1A,DUSP10,ELF3,ENPP5,EPHX1,FN1,H1F0,HMMR,HNRNPH3,ID2,JUNB,MCM2,MCM3,MCM5,MCM6,MCM7,MKI67,MYC,NFIL3,NREP,PCNA,PEG3,PLK1,POLA1,POLE2,PTGES,RFC5,RRM2,SCD,SH3BP5,TLR2,TOP2A,ZFX
**raloxifene**	chemical drug		-0.208	6.22E-10	ADCY9,ANXA3,AREG,BCL3,BMP4,CBX6,CDKN1A,CLK1,CLU,CTSH,EDN3,ERBB2,ERBB3,FMO5,HSPA1A/HSPA1B,IFI30,IL18,IL6,KRT7,LDLR,MFAP5,MIF,MKI67,MMP2,PCNA,PPP1R3C,PRSS23,PTGS2,PTTG1,RFC4,SELENBP1,SERPINE1,SIAH2,SPP1,TFRC,THBD,TIMP1,TOP2A,ZFX
**cisplatin**	chemical drug	Activated	2.839	7.27E-10	ACADM,ADAMTS1,ALB,ANXA1,ASCC1,ATF3,ATF4,BCL2L1,BIRC3,BIRC5,BMP4,C8orf4,CASP6,CAV1,Ccl2,CCR1,CD14,CDK1,CDKN1A,CHKA,CLDN4,CLIP4,CLU,CP,CTGF,CXCL10,CXCL14,DHRS3,EDN1,ELF3,ERBB3,F3,FADS3,FASN,FBXW7,FCGR2A,FN1,FOSL1,FYN,GPX2,GRIA1,H2AFX,HEYL,HK2,HMGCR,HMGCS1,ID2,IGFBP3,IL18,IL1B,IL4R,IL6,ISYNA1,ITGA7,KRT19,LCN2,LGALS3,MAT1A,MCM3,MMP2,Mt1,MYC,NEO1,NOX4,NREP,PIDD1,PIK3C3,PLK1,PTGES,Pvr,RAD51,RPS27L,RRM2,SERPINE1,SESN1,SLC25A36,SLC2A3,SOCS3,SPP1,STMN1,THBS1,TIMP1,TLE4,TNFRSF12A,TOP2A,TUBB,UHRF1,USP3,VCAM1,XPC
**AGN194204**	chemical drug		1.306	8.40E-10	ARL4C,ATF3,AURKA,CCNA2,CDK1,CDKN1A,CHKA,CYP51A1,CYP7B1,DENND5A,DHCR7,FDPS,FDX1,Foxp1,GATA3,GBP2,GJA1,H2AFX,HIST1H1C,HK2,Hmgn2 (includes others),Igtp,IL4R,IRS2,ITGB2,KIF20A,KIF4A,LGALS1,LSS,MAD2L1,MKI67,MMP9,MYC,NSMAF,PHLDA1,PLK1,PRC1,SCD,SPP1,TIMP1,TOP2A
**tretinoin**	chemical - endogenous mammalian	Inhibited	-3.136	8.88E-10	Acp5,ADAMTS5,ALDH3A1,ANGPTL4,ANXA1,ANXA6,APCDD1,APLN,APLNR,AREG,ATF4,BCL2L1,BCL3,BHLHE40,BIRC3,BIRC5,BMP4,CALML3,CASP6,Ccl2,CCL20,CCL22,CCNA2,CCND2,CCNE1,CCR1,CD14,CD68,CD74,CDC20,CDH5,CDKN1A,CEBPB,CES1,CHKA,CHN2,CKS2,CP,CSF3R,CTGF,CTSC,CTSK,CTSS,CXCL10,CYP1A1,CYP2S1,CYP4B1,CYR61,DHRS3,DKK3,DMC1,DNMT1,EDN1,ELF3,EPCAM,EPHX1,F3,FCER1G,FN1,FOSL1,FOXO3,FST,GJA1,GPX1,GPX2,GRB10,GRIA1,GZMA,HBEGF,HDGF,HLA-A,HMGCS2,HOXA5,HOXB5,HS3ST1,HSD11B1,HSPB1,ID2,IFI44,IGFBP2,IGFBP3,IL1B,IL6,INMT,IRS1,ITGA6,ITGAX,ITGB2,IVD,KCNE4,KDR,KIF23,KIT,KRT18,KRT19,KRT7,LAMA4,LEPR,LGALS1,LRP2,MAP3K5,MEF2C,MERTK,MGST3,MMP11,MMP14,MMP9,MPP6,MS4A1,Mt1,MTHFD2,MYC,NCL,NCOA5,NFE2,NFE2L2,NME2,NR3C1,P2RY2,P4HA2,PARP14,PFN1,PGD,PHLDA1,PIK3C3,PLA2G7,PLAUR,PLCE1,PTGES,PTGS1,PTGS2,PTPRU,RARRES2,RBL2,RFC4,RTKN2,S100A8,S100A9,SCD,SELL,SERPINE1,SERPING1,SLC16A1,SLC25A36,SLC38A2,SOX4,SPARC,SPP1,STAT6,TAL1,TBX2,TFRC,TGFBI,TGM1,THBD,THBS1,TIMM8A,TIMP1,TK1,TLR2,TNC,TNFAIP2,TNFRSF19,Tnfsf9,TOP2A,TSC22D3,TSHB,TYROBP,UBE2C,USP3,VCAM1,VMP1,XRCC5
**sirolimus**	chemical drug	Inhibited	-3.212	1.56E-09	ACADM,ADSS,ATF4,ATP6V1G1,BCKDHA,BCL2L1,BCL3,BEX1,BIRC5,Ccl2,CCND2,CCNE1,CD68,CDKN1A,CEBPB,DNAJC2,EIF1AX,EIF2S1,ERBB2,ERBB3,F3,FASN,FN1,FSCN1,GATA3,GCLC,GLA,GRIA1,HMGCS2,ID2,IL1B,IL4R,IL6,IRS1,IRS2,JUNB,KDR,LCN2,LDHA,LEPR,LGALS3,MMP14,MMP2,MMP9,Mt1,MYC,MYH11,NCL,NR3C1,PCNA,PGK1,PKM,PLAUR,PSMA3,PSMD8,S100A4,SCD,SELL,SERPINE1,SFN,SLC25A5,SLC2A3,SOCS3,SQLE,STMN1,THBD,TIMP1,TMPO,TOP2A,TRIM25,UCK2,VCAM1,VMP1
**sulforafan**	chemical drug		-0.297	1.81E-09	ABCB1,AKR1B10,AOX1,BCL2L1,BIRC5,CDKN1A,DDC,DNMT1,EPHX1,GATA3,GCLC,GPX1,GPX2,GSR,GSTA3,GSTM5,IL1B,IL6,MMP9,Mt1,NFE2L2,PTGES,PTGS2,SELE,SOCS3,TALDO1,TKT,TXNRD1,VCAM1,ZEB1
**deferoxamine**	chemical drug		1.559	1.84E-09	ANGPT2,ANGPTL4,APLN,BHLHE40,CCL20,CCND2,CCNE1,CD14,CDKN1A,CP,CYP3A5,DUSP10,DUSP16,ECM1,F3,FASN,HK2,HSPA1A/HSPA1B,IGFBP2,IGFBP3,IL6,ITGB2,KIT,LAMA3,LCN2,LDHA,ME1,MMP2,MS4A6A,MYC,NFIL3,PGK1,PKM,PLAUR,SELE,SERPINE1,SLC2A3,SPP1,SULF2,TFRC,VCAM1
**epigallocatechin-gallate**	chemical drug		-0.646	1.98E-09	ANGPT2,BCL2L1,BIRC5,CASP6,CCL17,CCL22,CDKN1A,CEBPB,CTGF,CTSK,CXCL10,CYR61,DNMT1,EDN1,ERBB2,F3,FN1,FOSL1,FOXO3,GCLC,GJA1,GSR,IL18,IL1B,IL6,JUNB,KDR,LDLR,MAP3K5,MKI67,MMP2,MMP9,MYC,NFE2L2,PCNA,PRKCE,PTGS2,SERPINE1,SLC16A1,TACSTD2,TFRC,TIMP1,TRAF1,VCAM1
**SB203580**	chemical - kinase inhibitor	Inhibited	-2.391	2.83E-09	Acp5,ADAMTS4,ADAMTS8,ANXA1,ANXA3,ATF3,BDKRB1,BIRC5,BMP4,CCL17,Ccl2,CCL20,CDK1,CDKN1A,CEBPD,CTGF,CTSK,CXCL10,CYP1A1,ERP29,F3,FN1,FPR2,GBP2,GJA1,GLRX,HAS1,IGFBP3,IL18,IL1B,IL1RN,IL6,JUNB,KDR,KRT23,MAT2A,MMP12,MMP2,MMP9,MYC,MYH11,PLAUR,PTGES,PTGS2,SELE,SERPINE1,SERPING1,SOCS3,SOD2,SPARC,SPP1,TGM1,THBS1,TIMP1,TIMP2,TLR2,TRAF1,VCAM1,ZFYVE26
**lovastatin**	chemical drug		0.868	3.94E-09	ADAMTS1,ALB,ATF3,ATF4,BCL2L1,BIRC5,CCNA2,CDKN1A,CTGF,DHCR7,FASN,FDPS,GATA3,GPX3,HMGCR,HMGCS1,IL1B,IL6,LDLR,MKI67,MPP6,MVD,MVK,MYC,NOX4,PMVK,PTGS2,RARRES2,RHOU,SERPINE1
**cyclosporin A**	biologic drug		1.069	4.23E-09	ADORA2B,ANG,ANXA1,ATF3,BCL2L1,BIRC3,CAV1,CD14,CD68,CDKN1A,CLDN4,CLU,CP,CTGF,CYP3A5,EDN1,EGR2,ELF3,F3,FASN,FCGR2A,FN1,FST,FYN,GJA1,GPX2,GZMA,H2AFX,HERPUD1,HMGCR,IL1B,IL6,KDR,LAMA3,LCN2,LDLR,LGALS3,LYVE1,MCM3,MDH2,MIF,MMP14,MMP2,MMP9,MYC,MYH11,NDUFB6,NFE2,NME1,NR3C1,PCNA,PKM,PRKACB,PTGS2,RCAN1,S100A9,SCD,SELE,SELL,SERPINE1,SERPINH1,SIAH2,SMPDL3B,SPP1,TFEC,TIMP1,TNFRSF12A,TUBB,VCAM1
**methylprednisolone**	chemical drug	Activated	2.046	4.41E-09	Anp32a,ANXA1,ANXA3,C1QBP,Ccl2,CD74,CDH13,CDKN1A,CNN3,CTGF,CTSC,CTSH,CTSS,CYC1,DGKZ,DMD,EPHX1,F3,FBLN5,FBXW7,FCGR2A,FGF7,FN1,GBP2,GCH1,GCLC,GJB3,GNL3,HSPA1A/HSPA1B,HSPH1,IL1B,IL1RN,IL6,IRGM,ITGA7,IVD,KRT19,LAMA3,LDLR,LYVE1,ME1,MMP11,MMP9,Mt1,MVK,MYBBP1A,MYC,NCL,NFE2L2,NME1,NR3C1,PC,PCNA,PGAM1,PIGR,PLAUR,PLLP,PRSS8,PSAT1,RBL2,RGS2,SCEL,SDC1,SERPINA1,SERPINA3,SERPINE1,SLC16A1,SLC25A10,SLC2A3,SLC40A1,SORD,SPARC,Sult1a1,TACSTD2,TFRC,TLR2,TMEM53,TOMM20,TOP2A,TUBA1C,VCAM1,VMP1
**atorvastatin**	chemical drug		1.525	4.69E-09	ABCB1,BCL2L1,C8orf4,CAV1,Ccl2,CTGF,CYP51A1,CYR61,DHCR7,DMC1,EDN1,ELF3,F3,FDPS,GJA1,GSR,HMGCR,HMGCS1,IL6,ITGB2,JUNB,LDLR,LSS,MMP2,MMP9,MSMO1,MVD,MVK,NOX4,PMVK,PTGS2,RHEB,SELE,SERPINE1,SLCO1B3,SPP1,SQLE,TLR2,VCAM1
**allopurinol**	chemical drug	Activated	2.488	5.45E-09	ADAMTS1,ANXA1,BIRC3,CD14,CLDN4,CLU,CP,CXCL10,ELF3,FABP1,FCGR2A,GPX2,IL1B,LCN2,LGALS3,MCM3,MYC,PTGS2,Pvr,SPP1,TIMP1,TMEM173,TNFRSF12A,TUBB
**tamoxifen**	chemical drug		0.336	9.79E-09	ANG,BCL2L1,BIRC5,BMP4,CAV1,CCNA2,CDKN1A,CLU,CSAD,CYP2F1,CYP3A5,CYR61,EPHX1,ERBB2,ERBB3,ETV5,FABP1,FASN,HMGCR,HMGCS1,IGFBP3,IL1RN,IL6,IRS1,JUNB,KLHL24,LDLR,LSS,LTBP2,MAFF,MIF,MKI67,MYC,PCNA,PFN1,PRSS23,PTGS2,RBL2,S100G,SCD,SCUBE2,SELE,SERPINA3,SLC2A3,SOD2,SPP1,USP1,YPEL3
**fluticasone propionate**	chemical drug		-1.907	9.84E-09	ANGPTL4,CCL20,CCL22,CD74,CES1,CHI3L1,CXCL10,FBLN5,FSCN1,GAL,HLA-DRA,HPGD,HSD11B1,HTRA1,IL1B,IL1RN,IRS2,MERTK,MFGE8,MMP9,PCOLCE2,PHLDA1,PTGS1,RCAN1,S100A8,SERPINA1,SERPINE1,SESN1,SLC1A3,SPP1,THBS1,TIMP1,TSC22D3,ZCCHC6
**bee venom**	chemical - endogenous non-mammalian		-0.557	1.17E-08	AREG,Ccl2,CDC42EP4,CREBBP,DNAJB1,HAS1,HSPA4L,HSPB1,IFRD1,IL1B,IPO5,JUNB,KIF23,MKI67,NFIL3,NUSAP1,PRC1,PTGS2,RCAN1,RGS2,Saa3,SOCS3,THBD,TNFAIP2,Tnfsf9,TOP2A,TRAF1
**rosiglitazone**	chemical drug	Inhibited	-2.783	1.72E-08	ACADM,ADAMTS1,ADRB3,ANGPTL4,C1QC,CAV1,CCL17,Ccl2,CD68,CDKN1A,CLEC7A,CTGF,CXCL10,CYP1A1,DHRS3,F3,FABP1,FASN,FDX1,FGF1,FN1,FOSL1,GATA3,GBP2,GRIA1,HAS1,HLA-DRA,HPGD,HSD11B1,IGFBP2,IL18,IL1B,IL1RN,IL6,IRS1,IRS2,KRT18,LCN2,LRG1,MAT2A,MCM6,MCM7,MDH2,MMP9,NOX4,NR1D1,PC,PCNA,PLTP,PTGES,PTGS2,RARRES2,Retnla,RGS2,RORA,S100A8,S100A9,Saa3,SCD,SERPINE1,SOCS3,SOD2,THBS1,TIMP1,TNC,VLDLR,WISP1
**chloropromazine**	chemical drug		0.41	1.81E-08	DHCR7,FABP1,FDPS,HMGCR,HMGCS1,IL18,IL1B,LDLR,LSS,MSMO1,MVK,SCD,SERPINA3,SLC2A3,SQLE,TMEM140,TSC22D3

Upstream Regulator	Molecule Type	Predicted Activation State	Activation z-score	p-value of overlap	Target molecules in dataset
**N-acetyl-L-cysteine**	chemical drug		-1.839	1.90E-08	ABCB1,ADORA2B,ATF4,BCL2L1,BIRC5,CAV1,Ccl2,CCL20,CCND2,CDKN1A,CTGF,CXCL10,CYR61,EDN1,FASN,FOXO3,GCLC,GNL3,HBEGF,HLA-DRA,IL1B,IL6,MMP2,MMP9,MYC,MYH11,NFE2L2,PCNA,PLAUR,PPRC1,PTGS2,SELE,SERPINE1,SLC16A1,SOD2,SPP1,TNC,VCAM1

Table S3. Tumor necrosis factor receptor (TNFR)-dependent basal lung genes.

Affymetrix_GeneChip_MOE430A. Moderated t-test ($p<0.05$). Order by fold difference (FD).

Slected genes varied >1.2-fold between C57BL/6J and Tnfr-KO mice (positive-higher in KO, negative-lower in KO).

p (WT:KO)	RefSeq Tran:	FD Air	Gene Symbol	Gene Title	Gene Ontology Biological Process
0.001472	NM_001045	-22.23	***Mup1///Mup10///Mup12///Mup13///Mup14///Mup15///Mup17///***	major urinary protein LOC100048885///predicted gene, 21320///major urinar	0006112 // energy reserve metabolic process // inferred from sequence or structural similarity///0006112 ,
2.26E-08	NM_001131	-4.27	***Pttg1***	pituitary tumor-transforming gene 1	0001558 // regulation of cell growth // not recorded///0006281 // DNA repair // inferred from electronic a
1.93E-07	NM_173781	-3.10	***Rab6b***	RAB6B, member RAS oncogene family	0006810 // transport // inferred from electronic annotation///0006886 // intracellular protein transport //
0.0191618	NM_001039	-2.66	***Mup1///Mup10///Mup11///Mup12///Mup14///Mup16///Mup18///***	major urinary protein 1///major urinary protein 10///major urinary protein 11	0006112 // energy reserve metabolic process // inferred from sequence or structural similarity///0006112 ,
0.0027583	NM_013650	-2.46	***S100a8***	S100 calcium binding protein A8 (calgranulin A)	0002376 // immune system process // inferred from electronic annotation///0002523 // leukocyte migrati
2.47E-04	NM_001281	-2.40	***S100a9***	S100 calcium binding protein A9 (calgranulin B)	0002376 // immune system process // inferred from electronic annotation///0002523 // leukocyte migrati
0.0174304	NM_008725	-2.34	***Nppa***	natriuretic peptide type A	0001666 // response to hypoxia // inferred from electronic annotation///0003085 // negative regulation o
0.0081202	NM_011458	-2.22	***Serpina3k***	serine (or cysteine) peptidase inhibitor, clade A, member 3K	0010466 // negative regulation of peptidase activity // inferred from electronic annotation///0010951 // n
3.42E-05	NM_010370	-2.10	***Gzma***	granzyme A	0006508 // proteolysis // inferred from electronic annotation///0006915 // apoptotic process // inferred fr
0.0332464	NM_001039	-2.08	***Npr3***	natriuretic peptide receptor 3	0001501 // skeletal system development // inferred from direct assay///0001501 // skeletal system develo
0.0016276	NM_009654	-1.99	***Alb***	albumin	0001895 // retina homeostasis // inferred from electronic annotation///0006810 // transport // inferred fr
0.0055523	NM_009245	-1.96	***Serpina1c***	serine (or cysteine) peptidase inhibitor, clade A, member 1C	0010466 // negative regulation of peptidase activity // inferred from electronic annotation///0010951 // n
0.0053431	NM_013559	-1.89	***Hsph1***	heat shock 105kDa/110kDa protein 1	0006986 // response to unfolded protein // traceable author statement///0043524 // negative regulation (
1.40E-04	NM_013653	-1.79	***Ccl5***	chemokine (C-C motif) ligand 5	0000165 // MAPK cascade // not recorded///0002230 // positive regulation of defense response to virus by
7.16E-05	NM_001243	-1.73	***Arntl***	aryl hydrocarbon receptor nuclear translocator-like	0000060 // protein import into nucleus, translocation // inferred from direct assay///0006351 // transcript
0.0031658	NM_001136	-1.67	***Cyp1a1***	cytochrome P450, family 1, subfamily a, polypeptide 1	0001666 // response to hypoxia // inferred from electronic annotation///0001889 // liver development // i
0.0069887	NM_001316	-1.66	***P4ha1***	procollagen-proline, 2-oxoglutarate 4-dioxygenase (proline 4-hydroxylase), alp	0018401 // peptidyl-proline hydroxylation to 4-hydroxy-L-proline // inferred from direct assay///0030199 /
0.0040327	NM_011607	-1.65	***Tnc***	tenascin C	0001649 // osteoblast differentiation // not recorded///0007155 // cell adhesion // inferred from electroni
0.0166123	NM_009244	-1.60	***Serpina1b***	serine (or cysteine) preptidase inhibitor, clade A, member 1B	0001701 // in utero embryonic development // inferred from mutant phenotype///0006487 // protein N-li
0.0010145	NM_001177	-1.58	***Pde4b***	phosphodiesterase 4B, cAMP specific	0001780 // neutrophil homeostasis // inferred from mutant phenotype///0006198 // cAMP catabolic proc
3.57E-05	NM_010809	-1.56	***Mmp3***	matrix metallopeptidase 3	0006508 // proteolysis // inferred from mutant phenotype///0006508 // proteolysis // not recorded///001
5.40E-05	NM_001311	-1.55	***Nnmt***	nicotinamide N-methyltransferase	0010243 // response to organonitrogen compound // inferred from electronic annotation///0031100 // or
0.0328579	NM_016737	-1.54	***Stip1***	stress-induced phosphoprotein 1	
0.0201377	NM_053247	-1.54	***Lyve1***	lymphatic vessel endothelial hyaluronan receptor 1	0006027 // glycosaminoglycan catabolic process // inferred from direct assay///0006810 // transport // inf
6.37E-04	NM_017373	-1.53	***Nfil3***	nuclear factor, interleukin 3, regulated	0000122 // negative regulation of transcription from RNA polymerase II promoter // not recorded///00063
0.0312099	NM_011020	-1.52	***Hspa4l***	heat shock protein 4 like	0006457 // protein folding // not recorded///0006986 // response to unfolded protein // not recorded
0.0370396	NM_198415	-1.52	***Ckmt2***	creatine kinase, mitochondrial 2	0006603 // phosphocreatine metabolic process // traceable author statement///0016310 // phosphorylati
7.06E-04	NM_008343	-1.52	***Igfbp3***	insulin-like growth factor binding protein 3	0001558 // regulation of cell growth // inferred from genetic interaction///0001649 // osteoblast different
0.0321213	NM_001305	-1.51	***Apoa2***	apolipoprotein A-II	0002526 // acute inflammatory response // inferred from electronic annotation///0002740 // negative reg
0.0256944	NM_009610	-1.50	***Actg2***	actin, gamma 2, smooth muscle, enteric	0010628 // positive regulation of gene expression // inferred from sequence or structural similarity///0090
0.0017118	NM_133903	-1.50	***Spon2***	spondin 2, extracellular matrix protein	0002376 // immune system process // inferred from electronic annotation///0002448 // mast cell mediate
0.0273517	NM_008303	-1.47	***Hspe1***	heat shock protein 1 (chaperonin 10)	0001649 // osteoblast differentiation // not recorded///0006457 // protein folding // inferred from electro
0.0456736	NM_001198	-1.46	***Csrp3***	cysteine and glycine-rich protein 3	0001974 // blood vessel remodeling // inferred from electronic annotation///0002026 // regulation of the
0.030649	NM_001317	-1.45	***Fgg***	fibrinogen gamma chain	0002250 // adaptive immune response // inferred from electronic annotation///0002376 // immune syster
9.35E-04	NM_001163	-1.45	***Prom1***	prominin 1	0010842 // retina layer formation // inferred from mutant phenotype///0045494 // photoreceptor cell ma
0.006453	NM_007771	-1.45	***Cry1***	cryptochrome 1 (photolyase-like)	0000122 // negative regulation of transcription from RNA polymerase II promoter // inferred from mutant
0.0039426	NM_007818	-1.44	***Cyp3a11***	cytochrome P450, family 3, subfamily a, polypeptide 11	0055114 // oxidation-reduction process // inferred from electronic annotation
0.0058147	NM_020509	-1.44	***Retnla***	resistin like alpha	
2.80E-04	NM_008719	-1.44	***Npas2***	neuronal PAS domain protein 2	0006351 // transcription, DNA-templated // inferred from electronic annotation///0006355 // regulation o
0.0022047	NM_022324	-1.43	***Sdf2l1***	stromal cell-derived factor 2-like 1	0000032 // cell wall mannoprotein biosynthetic process // not recorded///0007275 // multicellular organis
0.0085954	NM_001111	-1.42	***Serpinh1***	serine (or cysteine) peptidase inhibitor, clade H, member 1	0003433 // chondrocyte development involved in endochondral bone morphogenesis // inferred from mut
6.48E-04	NM_017399	-1.41	***Fabp1***	fatty acid binding protein 1, liver	0002230 // positive regulation of defense response to virus by host // not recorded///0006810 // transport
0.0016451	NM_018775	-1.41	***Tbc1d8***	TBC1 domain family, member 8	0043547 // positive regulation of GTPase activity // inferred from electronic annotation
0.0141223	NM_010477	-1.41	***Hspd1***	heat shock protein 1 (chaperonin)	0001666 // response to hypoxia // inferred from electronic annotation///0002236 // detection of misfolde
0.0013471	NM_019696	-1.40	***Cpxm1***	carboxypeptidase X 1 (M14 family)	0006508 // proteolysis // inferred from direct assay///0006518 // peptide metabolic process // ---///00081
0.0453166	NM_146036	-1.40	***Ahsa1***	AHA1, activator of heat shock protein ATPase 1	0006457 // protein folding // inferred from sequence or structural similarity///0006950 // response to stre
5.12E-04	NM_024253	-1.39	***Nkg7***	natural killer cell group 7 sequence	
2.92E-05	NM_009498	-1.39	***Vamp3***	vesicle-associated membrane protein 3	0001921 // positive regulation of receptor recycling // inferred from direct assay///0006461 // protein cor
0.0010585	NM_019738	-1.39	***Nupr1***	nuclear protein transcription regulator 1	0002526 // acute inflammatory response // inferred from mutant phenotype///0006351 // transcription, D
0.0288408	NM_010480	-1.39	***Hsp90aa1***	heat shock protein 90, alpha (cytosolic), class A member 1	0001764 // neuron migration // not recorded///0006457 // protein folding // inferred from electronic ann
0.0213734	NM_008096	-1.39	***Gc***	group specific component	0006810 // transport // inferred from electronic annotation///0007565 // female pregnancy // inferred fro
0.0416337	NM_001097	-1.38	***Hist1h2bc///Hist1h2be///Hist1h2bl///Hist1h2bm///Hist1h2bp///Hist.***	histone cluster 1, H2bc///histone cluster 1, H2be///histone cluster 1, H2bl///h	0002227 // innate immune response in mucosa // not recorded///0006334 // nucleosome assembly // not
0.0135876	NM_009692	-1.37	***Apoa1***	apolipoprotein A-I	0001932 // regulation of protein phosphorylation // inferred from direct assay///0001935 // endothelial ce
6.81E-04	NM_011332	-1.37	***Ccl17***	chemokine (C-C motif) ligand 17	0002548 // monocyte chemotaxis // ---///0006935 // chemotaxis // traceable author statement///000695
0.0028375	NM_009786	-1.37	***Cacybp***	calcyclin binding protein	0007507 // heart development // inferred from electronic annotation///0007568 // aging // inferred from
0.0026462	NM_026785	-1.37	***Ube2c***	ubiquitin-conjugating enzyme E2C	0000209 // protein polyubiquitination // not recorded///0006511 // ubiquitin-dependent protein catabolic
0.0031939	NM_001290	-1.37	***Ahsa2***	AHA1, activator of heat shock protein ATPase 2	0006950 // response to stress // inferred from electronic annotation///0032781 // positive regulation of AT
8.97E-05	NM_146063	-1.36	***Krt79***	keratin 79	
0.0047312	NM_011817	-1.34	***Gadd45g***	growth arrest and DNA-damage-inducible 45 gamma	0000185 // activation of MAPKKK activity // not recorded///0000186 // activation of MAPKK activity // infe
5.69E-05	NM_026174	-1.34	***Entpd4///Gm21685***	ectonucleoside triphosphate diphosphohydrolase 4///predicted gene, 21685	0006256 // UDP catabolic process // not recorded///0008152 // metabolic process // inferred from electro
2.19E-04	NM_001270	-1.33	***Plac9a///Plac9b***	placenta specific 9a///placenta specific 9b	
0.0069225	NM_016668	-1.33	***Bhmt***	betaine-homocysteine methyltransferase	0006479 // protein methylation // not recorded///0006577 // amino-acid betaine metabolic process // not
0.0120883	NM_001285	-1.33	***Tmem33***	transmembrane protein 33	0034613 // cellular protein localization // not recorded///0051292 // nuclear pore complex assembly // no

p (WT:KO)	RefSeq Tran:	FD Air	Gene Symbol	Gene Title	Gene Ontology Biological Process
0.015553	NM_001111	-1.33	***Cdkn1a***	cyclin-dependent kinase inhibitor 1A (P21)	0000079 // regulation of cyclin-dependent protein serine/threonine kinase activity // inferred from mutant
0.0111063	NM_134110	-1.33	***Kcne2***	potassium voltage-gated channel, Isk-related subfamily, gene 2	0006810 // transport // inferred from electronic annotation///0006811 // ion transport // inferred from ele
0.0097235	NM_007638	-1.33	***Cct7***	chaperonin containing Tcp1, subunit 7 (eta)	0006457 // protein folding // inferred from electronic annotation///0007339 // binding of sperm to zona p
0.0036645	NM_013697	-1.32	***Ttr***	transthyretin	0006810 // transport // inferred from electronic annotation///0042572 // retinol metabolic process // not
0.0049989	NM_001111	-1.32	***Igf1***	insulin-like growth factor 1	0001649 // osteoblast differentiation // inferred from expression pattern///0001775 // cell activation // nc
0.028701	NM_010219	-1.32	***Fkbp4///LOC102642559***	FK506 binding protein 4///uncharacterized LOC102642559	0000413 // protein peptidyl-prolyl isomerization // not recorded///0006457 // protein folding // inferred fi
0.0193119	NM_001281	-1.32	***Ifi27l2a***	interferon, alpha-inducible protein 27 like 2A	0007568 // aging // inferred from direct assay///0009615 // response to virus // inferred from direct assay
0.003145	NM_011403	-1.32	***Slc4a1***	solute carrier family 4 (anion exchanger), member 1	0006810 // transport // inferred from electronic annotation///0006811 // ion transport // inferred from ele
0.0022191	NM_001081	-1.31	***Mki67***	antigen identified by monoclonal antibody Ki 67	0006259 // DNA metabolic process // inferred from electronic annotation///0007126 // meiotic nuclear div
1.61E-04	NM_010391	-1.31	***H2-Q10***	histocompatibility 2, Q region locus 10	0002376 // immune system process // inferred from electronic annotation///0002474 // antigen processin
0.0242281	NM_021384	-1.31	***Rsad2***	radical S-adenosyl methionine domain containing 2	0001503 // ossification // not recorded///0002376 // immune system process // inferred from electronic ar
6.87E-04	NM_009978	-1.30	***Cst8***	cystatin 8 (cystatin-related epididymal spermatogenic)	0010466 // negative regulation of peptidase activity // inferred from electronic annotation///0010951 // n
0.0100065	NM_009912	-1.30	***Ccr1***	chemokine (C-C motif) receptor 1	0006816 // calcium ion transport // not recorded///0006874 // cellular calcium ion homeostasis // not reco
0.009008	NM_027959	-1.30	***Pdia6***	protein disulfide isomerase associated 6	0006457 // protein folding // not recorded///0008152 // metabolic process // inferred from electronic ann
0.0062015	NM_001110	-1.30	***Apoc1***	apolipoprotein C-I	0006641 // triglyceride metabolic process // inferred from mutant phenotype///0006810 // transport // in
0.0108044	NM_011631	-1.29	***Hsp90b1***	heat shock protein 90, beta (Grp94), member 1	0001666 // response to hypoxia // not recorded///0006457 // protein folding // inferred from electronic ar
0.0015525	NM_145562	-1.29	***Parm1***	prostate androgen-regulated mucin-like protein 1	0051973 // positive regulation of telomerase activity // not recorded
0.0062092	NM_133981	-1.29	***Alg9***	asparagine-linked glycosylation 9 (alpha 1,2 mannosyltransferase)	0006486 // protein glycosylation // inferred from electronic annotation///0008152 // metabolic process //
0.0070554	NM_012006	-1.29	***Acot1///Acot2***	acyl-CoA thioesterase 1///acyl-CoA thioesterase 2	0001676 // long-chain fatty acid metabolic process // inferred from direct assay///0006637 // acyl-CoA me
0.0324248	NM_011082	-1.29	***Pigr***	polymeric immunoglobulin receptor	0001580 // detection of chemical stimulus involved in sensory perception of bitter taste // not recorded///
0.0099119	NM_010495	-1.28	***Id1***	inhibitor of DNA binding 1	0000122 // negative regulation of transcription from RNA polymerase II promoter // inferred from direct as
0.0085936	NM_011568	-1.28	***Alyref///Alyref2***	Aly/REF export factor///Aly/REF export factor 2	0006397 // mRNA processing // inferred from electronic annotation///0006406 // mRNA export from nucle
0.0053837	NM_001290	-1.28	***Acer2***	alkaline ceramidase 2	0001953 // negative regulation of cell-matrix adhesion // not recorded///0006629 // lipid metabolic proce:
0.0050811	NM_001077	-1.28	***Ldb2***	LIM domain binding 2	0001942 // hair follicle development // inferred from genetic interaction///0006357 // regulation of transc
0.0482885	NM_133726	-1.28	***St13***	suppression of tumorigenicity 13	0006457 // protein folding // not recorded///0051260 // protein homooligomerization // not recorded///0
0.00238	NM_013599	-1.28	***Mmp9***	matrix metallopeptidase 9	0001501 // skeletal system development // inferred from genetic interaction///0001501 // skeletal system
0.0304962	NM_008877	-1.27	***Plg***	plasminogen	0006508 // proteolysis // inferred from electronic annotation///0007596 // blood coagulation // not recor
0.0026767	NM_176848	-1.27	***Fbxo2***	F-box protein 2	0006511 // ubiquitin-dependent protein catabolic process // inferred from direct assay///0006516 // glyco
0.020154	NM_010104	-1.27	***Edn1***	endothelin 1	0000122 // negative regulation of transcription from RNA polymerase II promoter // not recorded///00015
0.0148855	NM_177301	-1.27	***Hnrnpl***	heterogeneous nuclear ribonucleoprotein L	0006397 // mRNA processing // inferred from electronic annotation///0034198 // cellular response to ami
0.0318101	NM_001110	-1.27	***Atp2a2***	ATPase, Ca++ transporting, cardiac muscle, slow twitch 2	0002026 // regulation of the force of heart contraction // inferred from genetic interaction///0006810 // tr
0.0079441	NM_030599	-1.27	***Klrb1b***	killer cell lectin-like receptor subfamily B member 1B	0045953 // negative regulation of natural killer cell mediated cytotoxicity // inferred from direct assay///0C
3.66E-04	NM_010180	-1.27	***Fbln1***	fibulin 1	0001933 // negative regulation of protein phosphorylation // not recorded///0007162 // negative regulatio
0.0053302	NM_001163	-1.27	***Clec4d***	C-type lectin domain family 4, member d	0002250 // adaptive immune response // inferred from mutant phenotype///0002292 // T cell differentiat
0.0069436	NM_031165	-1.27	***Hspa8***	heat shock protein 8	0001916 // positive regulation of T cell mediated cytotoxicity // not recorded///0006351 // transcription, D
0.0062382	NM_029620	-1.27	***Pcolce2***	procollagen C-endopeptidase enhancer 2	0010952 // positive regulation of peptidase activity // not recorded
0.0100064	NM_001190	-1.26	***Clec4n***	C-type lectin domain family 4, member n	0002250 // adaptive immune response // inferred from electronic annotation///0002376 // immune syster
0.0332937	NM_007743	-1.26	***Col1a2***	collagen, type I, alpha 2	0001501 // skeletal system development // not recorded///0001568 // blood vessel development // not re
1.33E-04	NM_145923	-1.26	***Rell1***	RELT-like 1	
4.32E-04	NM_021789	-1.26	***Trappc4***	trafficking protein particle complex 4	0006810 // transport // inferred from electronic annotation///0006888 // ER to Golgi vesicle-mediated trai
0.0035458	NM_011545	-1.26	***Tcf21***	transcription factor 21	0000122 // negative regulation of transcription from RNA polymerase II promoter // inferred from direct as
3.99E-04	NM_001289	-1.26	***Cryab***	crystallin, alpha B	0001666 // response to hypoxia // inferred from mutant phenotype///0002088 // lens development in can
0.0049088	NM_008083	-1.26	***Gap43***	growth associated protein 43	0007275 // multicellular organismal development // inferred from electronic annotation///0007399 // nen
0.0074224	NM_001271	-1.26	***Ackr3***	atypical chemokine receptor 3	0001525 // angiogenesis // inferred from electronic annotation///0001570 // vasculogenesis // inferred frc
0.0291845	NM_001163	-1.26	***Tcf19***	transcription factor 19	
0.0014895	NM_010511	-1.26	***Ifngr1***	interferon gamma receptor 1	0019221 // cytokine-mediated signaling pathway // not recorded
0.0090109	NM_009850	-1.25	***Cd3g***	CD3 antigen, gamma polypeptide	0007163 // establishment or maintenance of cell polarity // not recorded///0007166 // cell surface recepto
0.0149551	NM_001145	-1.25	***Zkscan3***	zinc finger with KRAB and SCAN domains 3	0000122 // negative regulation of transcription from RNA polymerase II promoter // not recorded///00063
0.0480251	NM_008039	-1.25	***Fpr2***	formyl peptide receptor 2	0006935 // chemotaxis // inferred from electronic annotation///0007165 // signal transduction // inferred
0.0227478	NM_001190	-1.25	***Dnajb11***	DnaJ (Hsp40) homolog, subfamily B, member 11	0006457 // protein folding // inferred from electronic annotation///0016556 // mRNA modification // infer
0.0030634	NM_027411	-1.25	***Spdl1***	spindle apparatus coiled-coil protein 1	0000132 // establishment of mitotic spindle orientation // not recorded///0007049 // cell cycle // inferred
0.0459714	NM_138953	-1.24	***Ell2***	elongation factor RNA polymerase II 2	0006351 // transcription, DNA-templated // inferred from electronic annotation///0006355 // regulation o
0.0111285	NM_001285	-1.24	***Dapk1***	death associated protein kinase 1	0006417 // regulation of translation // inferred from electronic annotation///0006468 // protein phosphor
0.0080922	NM_024263	-1.24	***Mxra8***	matrix-remodelling associated 8	0060857 // establishment of glial blood-brain barrier // inferred from expression pattern
3.25E-04	NM_007875	-1.24	***Dpagt1***	dolichyl-phosphate (UDP-N-acetylglucosamine) acetylglucosaminephosphotra	0000271 // polysaccharide biosynthetic process // ---///0006047 // UDP-N-acetylglucosamine metabolic pr
0.0014476	NM_011609	-1.24	***Tnfrsf1a***	tumor necrosis factor receptor superfamily, member 1a	0006693 // prostaglandin metabolic process // traceable author statement///0006915 // apoptotic process
0.0010342	NM_026347	-1.24	***Iah1***	isoamyl acetate-hydrolyzing esterase 1 homolog (S. cerevisiae)	0006629 // lipid metabolic process // inferred from electronic annotation///0016042 // lipid catabolic proc
0.0031938	NM_015814	-1.24	***Dkk3***	dickkopf homolog 3 (Xenopus laevis)	0007275 // multicellular organismal development // inferred from electronic annotation///0016055 // Wn
0.0154652	NM_009943	-1.24	***Cox6a2***	cytochrome c oxidase subunit VIa polypeptide 2	1902600 // hydrogen ion transmembrane transport // inferred from electronic annotation
7.62E-04	NM_011280	-1.24	***Trim10***	tripartite motif-containing 10	0030218 // erythrocyte differentiation // inferred from direct assay///0045087 // innate immune response
9.96E-04	NM_011610	-1.23	***Tnfrsf1b***	tumor necrosis factor receptor superfamily, member 1b	0006954 // inflammatory response // inferred from mutant phenotype///0006955 // immune response // i
0.0017916	NM_001243	-1.23	***Tspan2***	tetraspanin 2	0006954 // inflammatory response // inferred from genetic interaction///0007166 // cell surface receptor s
0.0033299	NM_133238	-1.23	***Cd209a***	CD209a antigen	0006897 // endocytosis // inferred from electronic annotation///0010468 // regulation of gene expression
0.0230947	NM_012042	-1.23	***Cul1***	cullin 1	0006511 // ubiquitin-dependent protein catabolic process // inferred by curator///0006513 // protein mor
0.0039434	NM_013691	-1.23	***Thbs3***	thrombospondin 3	0003417 // growth plate cartilage development // inferred from genetic interaction///0003417 // growth p
9.48E-04	NM_001034	-1.23	***Ppp2r1b***	protein phosphatase 2, regulatory subunit A, beta	0006461 // protein complex assembly // inferred from electronic annotation///0034047 // regulation of pr

p (WT:KO)	RefSeq Tran:	FD Air	Gene Symbol	Gene Title	Gene Ontology Biological Process
0.0048828	NM_001081	-1.22	Palld	palladin, cytoskeletal associated protein	0003334 // keratinocyte development // inferred from mutant phenotype///0003382 // epithelial cell mor
0.00257	NM_019707	-1.22	Cdh13	cadherin 13	0000278 // mitotic cell cycle // inferred from electronic annotation///0001558 // regulation of cell growth
0.0406531	NM_007865	-1.22	Dll1	delta-like 1 (Drosophila)	0001701 // in utero embryonic development // non-traceable author statement///0001756 // somitogene:
7.90E-04	NM_007825	-1.22	Cyp7b1	cytochrome P450, family 7, subfamily b, polypeptide 1	0006629 // lipid metabolic process // inferred from electronic annotation///0006699 // bile acid biosynthe
0.038422	NM_008720	-1.22	Npc1	Niemann-Pick type C1	0006486 // protein glycosylation // inferred from direct assay///0006486 // protein glycosylation // not rec
0.0085297	NM_025348	-1.22	Ndufa3	NADH dehydrogenase (ubiquinone) 1 alpha subcomplex, 3	0006810 // transport // inferred from electronic annotation///0055114 // oxidation-reduction process // ir
0.0079752	NM_021288	-1.22	Tyms///Tyms-ps	thymidylate synthase///thymidylate synthase, pseudogene	0006206 // pyrimidine nucleobase metabolic process // inferred from electronic annotation///0006231 //
0.0424505	NM_013542	-1.22	Gzmb	granzyme B	0001913 // T cell mediated cytotoxicity // inferred from mutant phenotype///0006508 // proteolysis // inf
0.0278048	NM_008929	-1.22	Dnajc3	DnaJ (Hsp40) homolog, subfamily C, member 3	0006469 // negative regulation of protein kinase activity // inferred from sequence or structural similarity/
0.0167853	NM_009131	-1.22	Clec11a	C-type lectin domain family 11, member a	0008284 // positive regulation of cell proliferation // not recorded
0.0069927	NM_009763	-1.22	Bst1	bone marrow stromal cell antigen 1	0008152 // metabolic process // not recorded///0030890 // positive regulation of B cell proliferation // no
0.0065881	NM_028394	-1.22	Hspb11	heat shock protein family B (small), member 11	0001501 // skeletal system development // inferred from mutant phenotype///0006810 // transport // inf
0.0022784	NM_012033	-1.22	Tinag	tubulointerstitial nephritis antigen	0006508 // proteolysis // inferred from electronic annotation///0006898 // receptor-mediated endocytosi:
0.0043699	NM_011794	-1.21	Bpnt1	bisphosphate 3'-nucleotidase 1	0006139 // nucleobase-containing compound metabolic process // not recorded///0006470 // protein dep
0.0057984	NM_009829	-1.21	Ccnd2	cyclin D2	0000082 // G1/S transition of mitotic cell cycle // not recorded///0001934 // positive regulation of protein
0.011662	NM_010369	-1.21	Gypa	glycophorin A	0007016 // cytoskeletal anchoring at plasma membrane // inferred from mutant phenotype///0047484 //
0.0352691	NM_001166	-1.21	Fkbp2	FK506 binding protein 2	0000413 // protein peptidyl-prolyl isomerization // not recorded///0006457 // protein folding // inferred fi
0.031296	NM_009627	-1.21	Adm	adrenomedullin	0001570 // vasculogenesis // not recorded///0001666 // response to hypoxia // inferred from electronic ar
0.0118508	NM_026602	-1.21	Bcas2	breast carcinoma amplified sequence 2	0000398 // mRNA splicing, via spliceosome // not recorded///0006397 // mRNA processing // inferred fron
0.0019934	NM_025319	-1.21	0610009B22Rik	RIKEN cDNA 0610009B22 gene	0006351 // transcription, DNA-templated // inferred from electronic annotation///0006355 // regulation o
0.0385818	NM_016752	-1.21	Slc35b1	solute carrier family 35, member B1	0006810 // transport // inferred from electronic annotation///0008643 // carbohydrate transport // inferr
0.0061123	NM_001252	-1.21	Csf3r	colony stimulating factor 3 receptor (granulocyte)	0007155 // cell adhesion // inferred from electronic annotation///0019221 // cytokine-mediated signaling
0.032887	NM_001305	-1.21	Apoe	apolipoprotein E	0001937 // negative regulation of endothelial cell proliferation // not recorded///0002021 // response to c
0.0259667	NM_177564	-1.21	Dhrs11	dehydrogenase/reductase (SDR family) member 11	0008152 // metabolic process // inferred from electronic annotation///0055114 // oxidation-reduction prc
0.0101478	NM_028176	-1.21	Cda	cytidine deaminase	0009972 // cytidine deamination // not recorded///0030308 // negative regulation of cell growth // not re
0.0017991	NM_001135	-1.20	Gm38718///Gm39869///Ptprj	predicted gene, 38718///predicted gene, 39869///protein tyrosine phosphata	0001570 // vasculogenesis // inferred from mutant phenotype///0001954 // positive regulation of cell-mat
0.0176624	NM_133198	-1.20	Pygl	liver glycogen phosphorylase	0005975 // carbohydrate metabolic process // inferred from electronic annotation///0005977 // glycogen
0.0018152	NM_001113	-1.20	Isg20	interferon-stimulated protein	0000738 // DNA catabolic process, exonucleolytic // not recorded///0002376 // immune system process //
0.0045163	NM_016696	-1.20	Gpc1	glypican 1	0014037 // Schwann cell differentiation // not recorded///0030200 // heparan sulfate proteoglycan catabo
0.0300747	NM_134188	-1.20	Acot2///Acot1	acyl-CoA thioesterase 2///acyl-CoA thioesterase 1	0000038 // very long-chain fatty acid metabolic process // not recorded///0001666 // response to hypoxia
0.0155165	NM_010006	-1.20	Cyp2d9	cytochrome P450, family 2, subfamily d, polypeptide 9	0006805 // xenobiotic metabolic process // not recorded///0019369 // arachidonic acid metabolic process
0.0349869	NM_018870	-1.20	Pgam2	phosphoglycerate mutase 2	0006094 // gluconeogenesis // not recorded///0006096 // glycolytic process // inferred from direct assay//
0.0253425	NM_008611	-1.20	Mmp8	matrix metallopeptidase 8	0006508 // proteolysis // not recorded///0030574 // collagen catabolic process // inferred from electronic
0.0043765	NM_001033	-1.20	Dnah1	dynein, axonemal, heavy chain 1	0003341 // cilium movement // inferred from electronic annotation///0003351 // epithelial cilium movem
0.0235566	NM_019474	-1.20	Olfr156	olfactory receptor 156	0007165 // signal transduction // inferred from electronic annotation///0007186 // G-protein coupled rece
0.0323284	NM_146167	-1.20	Gimap7	GTPase, IMAP family member 7	0008152 // metabolic process // inferred from electronic annotation
0.0397775	NM_001004	-1.20	Gprasp1	G protein-coupled receptor associated sorting protein 1	0008333 // endosome to lysosome transport // not recorded///1990172 // G-protein coupled receptor cat
0.0145098	NM_025569	-1.20	Mgst3	microsomal glutathione S-transferase 3	0010243 // response to organonitrogen compound // inferred from electronic annotation///0055114 // ox
0.0043618	NM_019791	-1.20	Maged1	melanoma antigen, family D, 1	0006357 // regulation of transcription from RNA polymerase II promoter // inferred from direct assay///00
0.0215543	NM_029614	-1.20	Prss23	protease, serine 23	0006508 // proteolysis // inferred from electronic annotation
0.0342132	NM_001308	-1.20	Alox5ap	arachidonate 5-lipoxygenase activating protein	0002540 // leukotriene production involved in inflammatory response // inferred from mutant phenotype/
0.0384087	NM_010942	-1.20	Nsg1	neuron specific gene family member 1	0001921 // positive regulation of receptor recycling // not recorded///0007212 // dopamine receptor signa
0.0061579	NM_001002	-1.20	Hspa2	heat shock protein 2	0001934 // positive regulation of protein phosphorylation // inferred from mutant phenotype///0007140 /
0.0094518	NM_001169	1.20	B3gnt2	UDP-GlcNAc:betaGal beta-1,3-N-acetylglucosaminyltransferase 2	0006486 // protein glycosylation // inferred from mutant phenotype///0007411 // axon guidance // inferre
0.002047	NM_012017	1.20	Zfp346	zinc finger protein 346	0043065 // positive regulation of apoptotic process // inferred from mutant phenotype///0072332 // intrir
0.0225771	NM_144796	1.20	Susd4	sushi domain containing 4	0030449 // regulation of complement activation // not recorded///0045957 // negative regulation of comp
0.022997	NM_001110	1.20	Lsm2	LSM2 homolog, U6 small nuclear RNA associated (S. cerevisiae)	0000244 // spliceosomal tri-snRNP complex assembly // not recorded///0000398 // mRNA splicing, via spli
0.0328204	NM_175309	1.20	Upk3b	uroplakin 3B	0010629 // negative regulation of gene expression // not recorded///0046325 // negative regulation of glu
0.0172706	NM_172645	1.20	Suco	SUN domain containing ossification factor	0001503 // ossification // inferred from electronic annotation///0007275 // multicellular organismal devel
0.0197225	NM_010361	1.20	Gstt2	glutathione S-transferase, theta 2	0006749 // glutathione metabolic process // not recorded///0055114 // oxidation-reduction process // not
0.0120097	NM_001005	1.20	Syne2	spectrin repeat containing, nuclear envelope 2	0006998 // nuclear envelope organization // inferred from mutant phenotype///0007097 // nuclear migrat
0.0027919	NM_001079	1.20	Srsf5	serine/arginine-rich splicing factor 5	0006351 // transcription, DNA-templated // inferred from electronic annotation///0006355 // regulation o
0.0019571	NM_001098	1.20	Hic1	hypermethylated in cancer 1	0000122 // negative regulation of transcription from RNA polymerase II promoter // inferred from direct a:
0.0101649	NM_007426	1.20	Angpt2	angiopoietin 2	0001525 // angiogenesis // inferred from genetic interaction///0001666 // response to hypoxia // inferred
9.43E-04	NM_133677	1.20	Zbtb11os1	zinc finger and BTB domain containing 11, opposite strand 1	
0.0123404	NM_153533	1.20	Tns2	tensin 2	0001822 // kidney development // inferred from mutant phenotype///0006470 // protein dephosphorylati
6.32E-04	NM_010593	1.20	Jup	junction plakoglobin	0002159 // desmosome assembly // inferred from mutant phenotype///0002159 // desmosome assembly
0.013172	NM_001164	1.20	Hspb2	heat shock protein 2	0007525 // somatic muscle development // inferred from genetic interaction///0009408 // response to he
0.0280921	NM_001164	1.21	Sptssb	serine palmitoyltransferase, small subunit B	0006629 // lipid metabolic process // inferred from electronic annotation///0006665 // sphingolipid metab
0.0086943	NM_133710	1.21	Ctdspl	CTD (carboxy-terminal domain, RNA polymerase II, polypeptide A) small phos	0001933 // negative regulation of protein phosphorylation // inferred from direct assay///0006470 // prot
0.0100975	NM_010798	1.21	Mif	macrophage migration inhibitory factor	0001516 // prostaglandin biosynthetic process // not recorded///0001934 // positive regulation of protein
0.0072266	NM_138683	1.21	Rspo1	R-spondin 1	0001934 // positive regulation of protein phosphorylation // not recorded///0002090 // regulation of rece
0.0197331	NM_153459	1.21	Dusp7	dual specificity phosphatase 7	0000188 // inactivation of MAPK activity // not recorded///0006470 // protein dephosphorylation // not re
0.028392	NM_001308	1.21	Porcn	porcupine homolog (Drosophila)	0006497 // protein lipidation // inferred from direct assay///0009100 // glycoprotein metabolic process //
8.86E-04	NM_008027	1.21	Flot1	flotillin 1	0001765 // membrane raft assembly // not recorded///0001819 // positive regulation of cytokine producti
0.0051718	NM_001284	1.21	Bcl2l11	BCL2-like 11 (apoptosis facilitator)	0001701 // in utero embryonic development // inferred from genetic interaction///0001776 // leukocyte h

p (WT:KO)	RefSeq Tran:	FD Air	Gene Symbol	Gene Title	Gene Ontology Biological Process
0.0040917	NM_001163	1.21	***Fbxo6***	F-box protein 6	0006281 // DNA repair // inferred from electronic annotation///0006516 // glycoprotein catabolic process
0.0011939	NM_173440	1.21	***Nrip1***	nuclear receptor interacting protein 1	0000122 // negative regulation of transcription from RNA polymerase II promoter // inferred from direct a:
0.0103284	NM_001164	1.21	***Pias2***	protein inhibitor of activated STAT 2	0006351 // transcription, DNA-templated // inferred from direct assay///0006355 // regulation of transcrip
0.0066668	NM_001111	1.21	***Ngef***	neuronal guanine nucleotide exchange factor	0007275 // multicellular organismal development // inferred from electronic annotation///0007399 // ner
7.45E-04	NM_001272	1.21	***Trim24***	tripartite motif-containing 24	0006351 // transcription, DNA-templated // inferred from electronic annotation///0006355 // regulation o
0.0022797	NM_027198	1.21	***Zswim7***	zinc finger SWIM-type containing 7	0000724 // double-strand break repair via homologous recombination // not recorded///0006281 // DNA r
0.0293327	NM_001083	1.21	***Scn3b***	sodium channel, voltage-gated, type III, beta	0006810 // transport // inferred from electronic annotation///0006811 // ion transport // inferred from el
0.0227504	NM_153789	1.22	***Mylip***	myosin regulatory light chain interacting protein	0006928 // movement of cell or subcellular component // not recorded///0007399 // nervous system deve
0.0162668	NM_138951	1.22	***Ttc36***	tetratricopeptide repeat domain 36	
0.0072588	NM_009759	1.22	***Bmx***	BMX non-receptor tyrosine kinase	0002250 // adaptive immune response // not recorded///0006468 // protein phosphorylation // inferred fi
0.0370463	NM_007974	1.22	***F2rl1***	coagulation factor II (thrombin) receptor-like 1	0002286 // T cell activation involved in immune response // inferred from direct assay///0002376 // immu
0.0153785	NM_008012	1.22	***Akr1b8***	aldo-keto reductase family 1, member B8	0016488 // farnesol catabolic process // not recorded///0044597 // daunorubicin metabolic process // not
0.0082785	NM_145403	1.22	***Tmprss4***	transmembrane protease, serine 4	0006508 // proteolysis // inferred from electronic annotation///0006898 // receptor-mediated endocytosis
0.0197822	NM_027168	1.22	***Hddc2***	HD domain containing 2	
0.0085118	NM_026058	1.22	***Cers4***	ceramide synthase 4	0006629 // lipid metabolic process // inferred from electronic annotation///0030148 // sphingolipid biosyr
6.43E-04	NM_030206	1.22	***Cygb***	cytoglobin	0001666 // response to hypoxia // inferred from electronic annotation///0006810 // transport // inferred f
0.0020733	NM_001199	1.22	***Prrc2a***	proline-rich coiled-coil 2A	
0.0011858	NM_172736	1.22	***Leng8***	leukocyte receptor cluster (LRC) member 8	
0.0173041	NM_027356	1.22	***Ufsp1***	UFM1-specific peptidase 1	0006508 // proteolysis // inferred from mutant phenotype
0.0067067	NM_001025	1.23	***Brd2***	bromodomain containing 2	0006334 // nucleosome assembly // not recorded///0006351 // transcription, DNA-templated // inferred fi
0.0075527	NM_001122	1.23	***Nab2***	Ngfi-A binding protein 2	0001958 // endochondral ossification // inferred from genetic interaction///0006351 // transcription, DNA
0.0410965	NM_009344	1.23	***Phlda1***	pleckstrin homology-like domain, family A, member 1	0006915 // apoptotic process // inferred from electronic annotation///0021879 // forebrain neuron differe
0.0465258	NM_001110	1.23	***Prg4***	proteoglycan 4 (megakaryocyte stimulating factor, articular superficial zone p	0006898 // receptor-mediated endocytosis // inferred from electronic annotation///0006955 // immune re
0.0021794	NM_033314	1.23	***Slco2a1***	solute carrier organic anion transporter family, member 2a1	0006810 // transport // inferred from electronic annotation///0006811 // ion transport // inferred from el
0.0165178	NM_153175	1.23	***Gimap6***	GTPase, IMAP family member 6	
0.0078029	NM_001289	1.23	***Per3***	period circadian clock 3	0000122 // negative regulation of transcription from RNA polymerase II promoter // inferred from direct a:
0.0044302	NM_018856	1.23	***Ccnl2***	cyclin L2	0000079 // regulation of cyclin-dependent protein serine/threonine kinase activity // inferred from electro
0.0029783	NM_019518	1.23	***Grasp***	GRP1 (general receptor for phosphoinositides 1)-associated scaffold protein	0006886 // intracellular protein transport // not recorded///0007165 // signal transduction // inferred fror
0.0027706	NM_001038	1.23	***Sertad2***	SERTA domain containing 2	0006351 // transcription, DNA-templated // inferred from electronic annotation///0006355 // regulation o
0.0058051	NM_145478	1.23	***Pim3***	proviral integration site 3	0006468 // protein phosphorylation // not recorded///0006915 // apoptotic process // inferred from elect
0.001162	NM_009201	1.24	***Slc1a5***	solute carrier family 1 (neutral amino acid transporter), member 5	0003333 // amino acid transmembrane transport // not recorded///0006810 // transport // inferred from
0.0014692	NM_001013	1.24	***Prkg1***	protein kinase, cGMP-dependent, type I	0001764 // neuron migration // inferred from mutant phenotype///0006468 // protein phosphorylation //
0.0149314	NM_001161	1.24	***Tnfrsf12a***	tumor necrosis factor receptor superfamily, member 12a	0001525 // angiogenesis // inferred from electronic annotation///0006915 // apoptotic process // inferred
0.0016175	NM_023831	1.24	***Ift46***	intraflagellar transport 46	0007224 // smoothened signaling pathway // inferred from mutant phenotype///0015031 // protein trans
0.0088693	NM_008256	1.24	***Hmgcs2***	3-hydroxy-3-methylglutaryl-Coenzyme A synthase 2	0001822 // kidney development // inferred from electronic annotation///0001889 // liver development //
0.0361806	NM_001289	1.24	***Zeb2***	zinc finger E-box binding homeobox 2	0000122 // negative regulation of transcription from RNA polymerase II promoter // inferred from mutant
0.0309031	NM_026573	1.24	***Upf3b***	UPF3 regulator of nonsense transcripts homolog B (yeast)	0000184 // nuclear-transcribed mRNA catabolic process, nonsense-mediated decay // not recorded///0006
0.0055189	NM_001289	1.24	***Tns1***	tensin 1	0007044 // cell-substrate junction assembly // inferred from mutant phenotype///0010761 // fibroblast m
0.0014352	NM_001114	1.24	***Slc16a11***	solute carrier family 16 (monocarboxylic acid transporters), member 11	0006629 // lipid metabolic process // not recorded///0006810 // transport // inferred from electronic anno
0.0078167	NM_172563	1.24	***Hlf***	hepatic leukemia factor	0006351 // transcription, DNA-templated // inferred from electronic annotation///0006355 // regulation o
0.0049926	NM_175030	1.24	***Tctex1d4***	Tctex1 domain containing 4	
0.0017945	NM_178931	1.24	***Tnfrsf14***	tumor necrosis factor receptor superfamily, member 14 (herpesvirus entry me	0002741 // positive regulation of cytokine secretion involved in immune response // inferred from mutant
4.30E-04	NM_007885	1.24	***Slc26a2***	solute carrier family 26 (sulfate transporter), member 2	0001503 // ossification // not recorded///0006810 // transport // inferred from electronic annotation///00
0.0016893	NM_007964	1.25	***Evi5***	ecotropic viral integration site 5	0007049 // cell cycle // inferred from electronic annotation///0042147 // retrograde transport, endosome
9.56E-04	NM_145828	1.25	***Xylt2***	xylosyltransferase II	0006024 // glycosaminoglycan biosynthetic process // inferred from mutant phenotype///0015012 // hepa
0.0114493	NM_145549	1.25	***Efcab7***	EF-hand calcium binding domain 7	
0.0028765	NM_001290	1.25	***Eps8l1***	EPS8-like 1	0007266 // Rho protein signal transduction // not recorded///0016601 // Rac protein signal transduction /
0.03092	NM_028136	1.25	***Dhx36***	DEAH (Asp-Glu-Ala-His) box polypeptide 36	0001503 // ossification // inferred from mutant phenotype///0006351 // transcription, DNA-templated // i
0.0276244	NM_145220	1.25	***Appl2***	adaptor protein, phosphotyrosine interaction, PH domain and leucine zipper c	0007049 // cell cycle // inferred from electronic annotation///0008283 // cell proliferation // not recorded
3.30E-04	NM_024472	1.25	***Cptp***	ceramide-1-phosphate transfer protein	0006810 // transport // inferred from electronic annotation///0006869 // lipid transport // inferred from e
0.0017087	NM_018874	1.25	***Pnliprp1***	pancreatic lipase related protein 1	0006629 // lipid metabolic process // inferred from electronic annotation
6.80E-04	NM_001161	1.25	***Gucy1b3***	guanylate cyclase 1, soluble, beta 3	0006182 // cGMP biosynthetic process // not recorded///0007165 // signal transduction // not recorded//,
7.54E-04	NM_001302	1.26	***Impdh1***	inosine 5'-phosphate dehydrogenase 1	0006164 // purine nucleotide biosynthetic process // inferred from mutant phenotype///0006177 // GMP I
0.0092268	NM_001277	1.26	***Acvrl1***	activin A receptor, type II-like 1	0001525 // angiogenesis // inferred from mutant phenotype///0001525 // angiogenesis // not recorded///
0.0150706	NM_019864	1.26	***Atr***	ataxia telangiectasia and Rad3 related	0000077 // DNA damage checkpoint // inferred from mutant phenotype///0000077 // DNA damage check
0.0017954	NM_133772	1.26	***Ssbp4***	single stranded DNA binding protein 4	
0.0015706	NM_013770	1.26	***Slc25a10***	solute carrier family 25 (mitochondrial carrier, dicarboxylate transporter), me	0006810 // transport // inferred from electronic annotation///0006817 // phosphate ion transport // not r
0.0040538	NM_017398	1.26	***Diaph2***	diaphanous related formin 2	0007015 // actin filament organization // inferred from electronic annotation///0007275 // multicellular o
0.0012928	NM_008344	1.26	***Igfbp6***	insulin-like growth factor binding protein 6	0001558 // regulation of cell growth // inferred from electronic annotation///0043567 // regulation of insu
0.0052937	NM_001174	1.26	***Camk2b***	calcium/calmodulin-dependent protein kinase II, beta	0000082 // G1/S transition of mitotic cell cycle // inferred from mutant phenotype///0002030 // inhibitory
0.0011485	NM_011066	1.26	***Per2***	period circadian clock 2	0000122 // negative regulation of transcription from RNA polymerase II promoter // inferred from direct a:
0.0015635	NM_008197	1.26	***H1f0***	H1 histone family, member 0	0006334 // nucleosome assembly // inferred from electronic annotation
0.0084722	NM_010518	1.26	***Igfbp5***	insulin-like growth factor binding protein 5	0001558 // regulation of cell growth // inferred from genetic interaction///0001649 // osteoblast different
0.0033936	NM_008416	1.26	***Junb***	jun B proto-oncogene	0000122 // negative regulation of transcription from RNA polymerase II promoter // not recorded///00015
5.01E-04	NM_001130	1.26	***Arhgef1***	Rho guanine nucleotide exchange factor (GEF) 1	0035023 // regulation of Rho protein signal transduction // inferred from electronic annotation///0038032
0.0042069	NM_010415	1.27	***Hbegf***	heparin-binding EGF-like growth factor	0001525 // angiogenesis // non-traceable author statement///0001832 // blastocyst growth // traceable a

p (WT:KO)	RefSeq Tran:	FD Air	Gene Symbol	Gene Title	Gene Ontology Biological Process
0.0391272	NM_008655	1.27	Gadd45b	growth arrest and DNA-damage-inducible 45 beta	0000185 // activation of MAPKKK activity // not recorded///0000186 // activation of MAPKK activity // infe
0.0014458	NM_025476	1.27	Rmdn1	regulator of microtubule dynamics 1	
0.0033835	NM_008055	1.27	Fzd4	frizzled homolog 4 (Drosophila)	0001553 // luteinization // inferred from mutant phenotype///0001568 // blood vessel development // inf
0.0177529	NM_025436	1.27	Msmo1	methylsterol monoxygenase 1	0006629 // lipid metabolic process // inferred from electronic annotation///0006633 // fatty acid biosynth
0.0255131	NM_001111	1.28	Zfp322a	zinc finger protein 322A	0006351 // transcription, DNA-templated // inferred from electronic annotation///0006355 // regulation o
0.0097232	NM_013833	1.28	Grp///Rax	gastrin releasing peptide///retina and anterior neural fold homeobox	0006351 // transcription, DNA-templated // inferred from electronic annotation///0006355 // regulation o
0.0027466	NM_001146	1.28	Nkx2-1	NK2 homeobox 1	0000122 // negative regulation of transcription from RNA polymerase II promoter // inferred from direct a:
0.0160011	NM_001290	1.28	Cwc22///Xdh	CWC22 spliceosome-associated protein homolog (S. cerevisiae)///xanthine de	0000398 // mRNA splicing, via spliceosome // not recorded///0001933 // negative regulation of protein ph
0.004408	NM_001168	1.28	Tmcc3	transmembrane and coiled coil domains 3	
0.044719	NM_001145	1.29	Slc15a2	solute carrier family 15 (H+/peptide transporter), member 2	0006810 // transport // inferred from electronic annotation///0006857 // oligopeptide transport // inferre
0.0067158	NM_011436	1.29	Sorl1	sortilin-related receptor, LDLR class A repeats-containing	0000042 // protein targeting to Golgi // not recorded///0006605 // protein targeting // not recorded///00
0.001724	NM_001163	1.29	Bri3	brain protein I3	
2.21E-04	NM_172505	1.29	A730008H23Rik///Hjurp///6430706D22Rik	RIKEN cDNA A730008H23 gene///Holliday junction recognition protein///RIKE	0007049 // cell cycle // inferred from electronic annotation///0007059 // chromosome segregation // not r
0.0089814	NM_009135	1.29	Scn7a	sodium channel, voltage-gated, type VII, alpha	0006810 // transport // inferred from electronic annotation///0006811 // ion transport // inferred from el
0.0189613	NM_019568	1.29	Cxcl14	chemokine (C-X-C motif) ligand 14	0006955 // immune response // inferred from electronic annotation///0045662 // negative regulation of m
0.0351907	NM_009438	1.29	Flt3l///Rpl13a	FMS-like tyrosine kinase 3 ligand///ribosomal protein L13A	0001934 // positive regulation of protein phosphorylation // inferred from genetic interaction///0006412 /
0.0029986	NM_026428	1.29	Dcxr	dicarbonyl L-xylulose reductase	0005975 // carbohydrate metabolic process // inferred from electronic annotation///0005997 // xylulose m
0.0049446	NM_001146	1.29	Pdgfrb	platelet derived growth factor receptor, beta polypeptide	0001568 // blood vessel development // inferred from expression pattern///0001701 // in utero embryoni
0.0110091	NM_021395	1.29	Hyou1	hypoxia up-regulated 1	0001666 // response to hypoxia // not recorded///0002931 // response to ischemia // inferred from direct
0.0010226	NM_007419	1.30	Adrb1	adrenergic receptor, beta 1	0001996 // positive regulation of heart rate by epinephrine-norepinephrine // inferred from mutant pheno
3.50E-04	NM_023133	1.30	Rps19///Rps19-ps1///Rps19-ps2///Rps19-ps3///Rps19-ps6///Rps19-p	ribosomal protein S19///ribosomal protein S19, pseudogene 1///ribosomal pr	0000028 // ribosomal small subunit assembly // not recorded///0000462 // maturation of SSU-rRNA from
9.47E-04	NM_001036	1.30	Atp2b2	ATPase, Ca++ transporting, plasma membrane 2	0000902 // cell morphogenesis // inferred from mutant phenotype///0006810 // transport // inferred from
0.0222483	NM_015800	1.30	Crim1	cysteine rich transmembrane BMP regulator 1 (chordin like)	0001558 // regulation of cell growth // inferred from electronic annotation///0010951 // negative regulati
0.0013962	NM_008898	1.30	Por	P450 (cytochrome) oxidoreductase	0003420 // regulation of growth plate cartilage chondrocyte proliferation // not recorded///0007584 // res
1.55E-04	NM_172505	1.30	6430706D22Rik///A730008H23Rik///Hjurp	Holliday junction recognition protein	0007049 // cell cycle // inferred from electronic annotation///0007059 // chromosome segregation // not r
0.0020454	NM_001109	1.31	Kazn	kazrin, periplakin interacting protein	0031424 // keratinization // inferred from electronic annotation
0.0054159	NM_009196	1.31	Slc16a1	solute carrier family 16 (monocarboxylic acid transporters), member 1	0006629 // lipid metabolic process // inferred from mutant phenotype///0006810 // transport // inferred f
9.29E-04	NM_001289	1.32	Pira1///Pira2///Pira6///Gm10693///Gm14548///Lilra6///Pira11///Pi	paired-Ig-like receptor A1///paired-Ig-like receptor A2///paired-Ig-like recepto	0001782 // B cell homeostasis // inferred from mutant phenotype///0002250 // adaptive immune respons
0.0156341	NM_021349	1.32	Tnfrsf13b	tumor necrosis factor receptor superfamily, member 13b	0001782 // B cell homeostasis // inferred from direct assay///0001782 // B cell homeostasis // inferred fro
0.0061959	NM_001081	1.32	Irs2	insulin receptor substrate 2	0002053 // positive regulation of mesenchymal cell proliferation // inferred from genetic interaction///000
0.0036453	NM_009911	1.32	Cxcr4	chemokine (C-X-C motif) receptor 4	0001569 // patterning of blood vessels // inferred from mutant phenotype///0001666 // response to hypo:
0.0237046	NM_001035	1.32	Akap2///Pakap	A kinase (PRKA) anchor protein 2///paralemmin A kinase anchor protein	0007015 // actin filament organization // inferred from direct assay///0007178 // transmembrane recepto
0.0390949	NM_001290	1.33	Ttc14	tetratricopeptide repeat domain 14	0001947 // heart looping // ---///0003341 // cilium movement // ---///0003356 // regulation of cilium beat
0.0101252	NM_001142	1.33	Lmo2	LIM domain only 2	0035162 // embryonic hemopoiesis // inferred from genetic interaction///0042789 // mRNA transcription f
0.0025517	NR_002847	1.34	Malat1	metastasis associated lung adenocarcinoma transcript 1 (non-coding RNA)	0000381 // regulation of alternative mRNA splicing, via spliceosome // inferred from mutant phenotype///
0.0015302	NM_001113	1.34	Ldb1	LIM domain binding 1	0000972 // transcription-dependent tethering of RNA polymerase II gene DNA at nuclear periphery // infer
0.0208486	NM_001077	1.35	Hnrnpd	heterogeneous nuclear ribonucleoprotein D	0006351 // transcription, DNA-templated // inferred from electronic annotation///0006355 // regulation o
0.0067467	NM_001164	1.35	Ankrd33b	ankyrin repeat domain 33B	
0.0090709	NM_018820	1.35	Sertad1	SERTA domain containing 1	0006351 // transcription, DNA-templated // inferred from electronic annotation///0006355 // regulation o
1.95E-04	NM_027873	1.35	Ubiad1	UbiA prenyltransferase domain containing 1	0001885 // endothelial cell development // inferred from sequence or structural similarity///0006744 // ub
0.0055772	NM_001111	1.36	Cd59a	CD59a antigen	0001775 // cell activation // ---///0001971 // negative regulation of activation of membrane attack comple
0.0028429	NM_001177	1.36	Pfkfb3	6-phosphofructo-2-kinase/fructose-2,6-biphosphatase 3	0006000 // fructose metabolic process // inferred from electronic annotation///0006003 // fructose 2,6-bi:
0.0195851	NM_009052	1.36	Bex1	brain expressed gene 1	0002052 // positive regulation of neuroblast proliferation // not recorded///0007275 // multicellular organ
6.30E-04	NM_001289	1.37	Sox17	SRY (sex determining region Y)-box 17	0000122 // negative regulation of transcription from RNA polymerase II promoter // inferred from mutant
2.78E-04	NM_021332	1.38	Glp1r	glucagon-like peptide 1 receptor	0006950 // response to stress // inferred from mutant phenotype///0007165 // signal transduction // infer
0.0017829	NM_001163	1.39	Emcn	endomucin	0001525 // angiogenesis // inferred from sequence or structural similarity///0016337 // single organismal c
0.0020934	NM_008360	1.39	Il18	interleukin 18	0000165 // MAPK cascade // not recorded///0001525 // angiogenesis // not recorded///0001525 // angiog
0.0069613	NM_013672	1.39	Sp1	trans-acting transcription factor 1	0001503 // ossification // inferred from genetic interaction///0001701 // in utero embryonic development
1.71E-04	NM_001083	1.40	Gm13139	predicted gene 13139	0002244 // hematopoietic progenitor cell differentiation // inferred from mutant phenotype///0006355 //
1.03E-04	NM_029357	1.40	Pcdh1	protocadherin 1	0007155 // cell adhesion // inferred from electronic annotation///0007156 // homophilic cell adhesion via
0.0440882	NM_007705	1.41	Cirbp	cold inducible RNA binding protein	0009409 // response to cold // inferred from mutant phenotype///0009411 // response to UV // not rec
2.70E-04	NM_001289	1.41	Pira1///Pira2///Pira6///Gm10693///Gm14548///Lilra6///Pira11///Pi	paired-Ig-like receptor A1///paired-Ig-like receptor A2///paired-Ig-like recepto	0001782 // B cell homeostasis // inferred from mutant phenotype///0002250 // adaptive immune respons
1.92E-04	NM_001111	1.41	Comt	catechol-O-methyltransferase	0006584 // catecholamine metabolic process // inferred from electronic annotation///0007565 // female p
1.31E-04	NM_011057	1.42	Pdgfb	platelet derived growth factor, B polypeptide	0001568 // blood vessel development // inferred from expression pattern///0001666 // response to hypox
0.0452824	NM_022983	1.45	Lpar3	lysophosphatidic acid receptor 3	0000187 // activation of MAPK activity // inferred from direct assay///0007165 // signal transduction // inf
0.0026965	NR_045032	1.45	Gdap10	ganglioside-induced differentiation-associated-protein 10	
2.59E-04	NM_001003	1.47	Pcdha1///Pcdha10///Pcdha11///Pcdha12///Pcdha2///Pcdha3///Pcdh	protocadherin alpha 1///protocadherin alpha 10///protocadherin alpha 11///	0007155 // cell adhesion // inferred from electronic annotation///0007155 // cell adhesion // traceable au
1.47E-04	NM_001252	1.49	Tspan4	tetraspanin 4	0006461 // protein complex assembly // not recorded///0007166 // cell surface receptor signaling pathwa
4.55E-05	NM_153068	1.51	Ehd2	EH-domain containing 2	0006897 // endocytosis // inferred from direct assay///0008152 // metabolic process // inferred from elect
1.07E-05	NM_009003	1.53	Rab4a	RAB4A, member RAS oncogene family	0006810 // transport // inferred from electronic annotation///0006886 // intracellular protein transport //
0.0335806	NM_001166	1.54	Gm15453///Rbm3	predicted gene 15453///RNA binding motif protein 3	0006412 // translation // inferred from direct assay///0006417 // regulation of translation // inferred from
0.0038689	NM_139149	1.55	Fus	fused in sarcoma	0071277 // cellular response to calcium ion // not recorded///1903506 // regulation of nucleic acid-templa
2.21E-04	NM_017376	1.55	Tef	thyrotroph embryonic factor	0006351 // transcription, DNA-templated // inferred from electronic annotation///0006355 // regulation o
0.0062066	NR_002840/	1.56	Gas5///Snord47	growth arrest specific 5///small nucleolar RNA, C/D box 47	
1.76E-05	NM_001160	1.57	Gnb1	guanine nucleotide binding protein (G protein), beta 1	0007165 // signal transduction // inferred from electronic annotation///0007186 // G-protein coupled rece
1.70E-06	NM_001111	1.60	Wdfy1	WD repeat and FYVE domain containing 1	0034141 // positive regulation of toll-like receptor 3 signaling pathway // inferred from mutant phenotype,

p (WT:KO)	RefSeq Tran:	FD Air	Gene Symbol	Gene Title	Gene Ontology Biological Process
3.15E-04	NM_001165	1.60	***Tshb***	thyroid stimulating hormone, beta subunit	0033189 // response to vitamin A // inferred from electronic annotation///0043627 // response to estroge
7.45E-04	NM_145434	1.64	***Nr1d1***	nuclear receptor subfamily 1, group D, member 1	0000122 // negative regulation of transcription from RNA polymerase II promoter // not recorded///00059
3.92E-04	NM_011122	1.65	***Plod1***	procollagen-lysine, 2-oxoglutarate 5-dioxygenase 1	0001666 // response to hypoxia // inferred from electronic annotation///0008544 // epidermis developme
0.0171234		1.67	***Ighd***	immunoglobulin heavy constant delta	0016064 // immunoglobulin mediated immune response // inferred from mutant phenotype///0016446 //
3.30E-04	NM_001003	1.68	***Pcdha1///Pcdha10///Pcdha11///Pcdha12///Pcdha2///Pcdha3///Pcdh***	protocadherin alpha 1///protocadherin alpha 10///protocadherin alpha 11///	0007155 // cell adhesion // inferred from electronic annotation///0007155 // cell adhesion // traceable au
0.0221363	NM_011584	1.79	***Nr1d2***	nuclear receptor subfamily 1, group D, member 2	0006351 // transcription, DNA-templated // inferred from electronic annotation///0006355 // regulation o
1.05E-04	XM_011238	1.88	***Gm3579///Gm40514///Gm40814///Gm40991///Gm42035///Gm421***	predicted gene 3579///predicted gene, 40514///predicted gene, 40814///pre	0015074 // DNA integration // inferred from electronic annotation///0090502 // RNA phosphodiester bonc
2.41E-05	NM_001308	1.92	***Nnt***	nicotinamide nucleotide transhydrogenase	0006740 // NADPH regeneration // not recorded///0015992 // proton transport // not recorded///004545
1.37E-07	NM_177673	1.92	***Cep104***	centrosomal protein 104	
4.55E-05	XM_011238	2.30	***Gm3579///Gm40514///Gm40814///Gm40991///Gm42035///Gm421***	predicted gene 3579///predicted gene, 40514///predicted gene, 40814///pre	0015074 // DNA integration // inferred from electronic annotation///0090502 // RNA phosphodiester bonc
1.15E-06	XM_011238	2.54	***Gm37416///Gm39743///Gm40611///Gm40814///Gm41035///Gm414***	predicted gene, 37416///predicted gene, 39743///predicted gene, 40611///p	0006278 // RNA-dependent DNA replication // inferred from electronic annotation///0008152 // metaboli
4.98E-06	NM_016974	3.66	***Dbp***	D site albumin promoter binding protein	0001889 // liver development // inferred from electronic annotation///0006351 // transcription, DNA-tem

Table S4. Tumor necrosis fector receptor (TNFR)-dependently changed lung genes by 0.3-parts per million (ppm) ozone (O_3).

Affymetrix_GeneChip_MOE430A. 2 Way ANOVA (p<0.05). Order by fold difference (FD) at 48 hr O3.

Slected genes varied at least one time point between C57BL/6J and Tnfr-KO mice (positive-higher in KO, negative-lower in KO).

RefSeq Tr	*p* 24h	*p* 48h	*p* 72h	FD Air	FD 24h	FD 48h	FD 72h	Gene Symbol	Gene Title	Gene Ontology Biological Process
NM_173781///XM_		0.027		-3.10	-3.96	-4.12	-2.90	***Rab6b***	RAB6B, member RAS oncogene family	0006810 // transport // inferred from electronic annotation///0006886 // intracellular pr
NM_001131054///		0.014	2E-04	-4.27	-4.12	-3.09	-1.84	***Pttg1***	pituitary tumor-transforming gene 1	0001558 // regulation of cell growth // not recorded///0006281 // DNA repair // inferred
NM_001045550///		0.046	0.019	-18.72	-4.81	-2.82	1.33	***Mup1///Mup10///Mup12///Mup13///Mu***	major urinary protein LOC100048885///predicted gene, 21320///major urinary protei	0006112 // energy reserve metabolic process // inferred from sequence or structural sim
NM_001143689///		0.027		-1.17	1.09	-2.08	-1.11	***H2-Q4///H2-Q6///H2-Q8///LOC68395***	histocompatibility 2, Q region locus 4///histocompatibility 2, Q region locus 6///histoc	0002474 // antigen processing and presentation of peptide antigen via MHC class I // not
NM_001198560///		0.048		-1.24	1.10	-1.95	-1.22	***H2-D4///H2-Q7///H2-Q9***	histocompatibility 2, D region locus 4///histocompatibility 2, Q region locus 7///histoc	0001916 // positive regulation of T cell mediated cytotoxicity // inferred from direct assay
NM_0136	0.0492			-1.79	-1.20	-1.95	-1.62	***Ccl5***	chemokine (C-C motif) ligand 5	0000165 // MAPK cascade // not recorded///0002230 // positive regulation of defense re
NM_0103	0.0319			-2.10	-1.27	-1.89	-1.36	***Gzma***	granzyme A	0006508 // proteolysis // inferred from electronic annotation///0006915 // apoptotic pro
NM_0173	0.0288			-1.41	-1.03	-1.88	-1.25	***Fabp1***	fatty acid binding protein 1, liver	0002230 // positive regulation of defense response to virus by host // not recorded///000
XM_0014	0.0038			1.03	1.62	-1.79	-1.31	***Ighm///Igh-V7183///Igh-VJ558///Ighg///I***	immunoglobulin heavy constant mu///immunoglobulin heavy chain (V7183 family)///i	0000187 // activation of MAPK activity // inferred from direct assay///0002455 // humor
NM_0136	0.0069			-2.46	1.98	-1.63	-2.00	***S100a8***	S100 calcium binding protein A8 (calgranulin A)	0002376 // immune system process // inferred from electronic annotation///0002523 //
NM_1460	0.0402			-1.36	-1.10	-1.63	-1.27	***Krt79***	keratin 79	
NM_0137	0.0092			-1.20	-3.52	-1.62	1.05	***Pdk4***	pyruvate dehydrogenase kinase, isoenzyme 4	0002230 // positive regulation of defense response to virus by host // not recorded///000
NM_001033301///		0.029		-1.19	-1.57	-1.60	-1.14	***Fhdc1***	FH2 domain containing 1	
NM_0010	0.013			-1.19	-1.57	-1.60	-1.14	***Fhdc1***	FH2 domain containing 1	
NM_1449	0.0264			-1.03	-1.31	-1.58	-1.12	***Ces1f***	carboxylesterase 1F	0019626 // short-chain fatty acid catabolic process // inferred from direct assay
NM_0012	0.0029			-2.40	2.34	-1.58	-2.05	***S100a9***	S100 calcium binding protein A9 (calgranulin B)	0002376 // immune system process // inferred from electronic annotation///0002523 //
NM_0228	0.0271	0.005		1.31	-1.21	-1.54	1.09	***Scel***	sciellin	**0008544 // epidermis development // inferred from direct assay///0009790 // embryo**
NM_029614///XM_006508			0.036	-1.20	-1.59	-1.52	1.04	***Prss23***	protease, serine 23	0006508 // proteolysis // inferred from electronic annotation
NM_0242	0.0127			-1.39	-1.03	-1.48	-1.30	***Nkg7***	natural killer cell group 7 sequence	
NM_0013	0.0076	0.016	0.011	-1.07	-1.36	-1.47	-1.85	***Igfbp2***	insulin-like growth factor binding protein 2	0001558 // regulation of cell growth // inferred from electronic annotation///0007165 //
NM_001271005///		0.049		-1.06	1.02	-1.46	-1.13	***C920025E04Rik///H2-T23***	RIKEN cDNA C920025E04 gene///histocompatibility 2, T region locus 23	0001916 // positive regulation of T cell mediated cytotoxicity // inferred from direct assay
	######			-1.01	1.60	-1.43	-1.12	***Igk***	immunoglobulin kappa chain complex	0006898 // receptor-mediated endocytosis // ---///0006910 // phagocytosis, recognition
NM_026880		0.011		-1.06	-1.16	-1.41	-1.07	***Pink1***	PTEN induced putative kinase 1	0000422 // mitophagy // not recorded///0001934 // positive regulation of protein phosp
NM_013462///XM_		0.034		1.04	1.05	-1.40	-1.17	***Adrb3***	adrenergic receptor, beta 3	0002024 // diet induced thermogenesis // inferred from genetic interaction///0002025 //
	######			1.04	2.09	-1.39	-1.09	***Igk-V28///Igkc///Igkj1///Igkv1-117///Igkv***	immunoglobulin kappa chain variable 28 (V28)///immunoglobulin kappa constant///in	0006898 // receptor-mediated endocytosis // not recorded///0006910 // phagocytosis, r
NM_010474		0.036		1.20	1.36	-1.38	1.15	***Hs3st1***	heparan sulfate (glucosamine) 3-O-sulfotransferase 1	0008152 // metabolic process // inferred from electronic annotation
NM_015786		0.011		1.01	-1.04	-1.37	-1.14	***Hist1h1c***	histone cluster 1, H1c	0000122 // negative regulation of transcription from RNA polymerase II promoter // infer
NM_133904///XM_		0.044		1.04	-1.03	-1.36	-1.21	***Acacb***	acetyl-Coenzyme A carboxylase beta	0006084 // acetyl-CoA metabolic process // not recorded///0006629 // lipid metabolic pr
NM_133229		0.044		-1.05	-1.18	-1.34	-1.16	***Ripply3***	ripply3 homolog (zebrafish)	0000122 // negative regulation of transcription from RNA polymerase II promoter // infer
NM_172578			0.004	-1.15	-1.07	-1.33	1.05	***Mis18bp1***	MIS18 binding protein 1	0007049 // cell cycle // inferred from electronic annotation///0007067 // mitotic nuclear
NM_0011	0.0131			-1.20	-1.01	-1.32	-1.16	***Gm38718///Gm39869///Ptprj***	predicted gene, 38718///predicted gene, 39869///protein tyrosine phosphatase, recep	0001570 // vasculogenesis // inferred from mutant phenotype///0001954 // positive regu
NM_0010	0.0145	0.019		1.05	-1.25	-1.32	-1.05	***Samd4***	sterile alpha motif domain containing 4	0006355 // regulation of transcription, DNA-templated // inferred from electronic annota
NM_001293690///		0.047		-1.02	-1.02	-1.31	-1.08	***Ido1***	indoleamine 2,3-dioxygenase 1	0002376 // immune system process // inferred from electronic annotation///0002534 //
NM_0097	0.0111	0.022	0.037	-1.07	-1.21	-1.31	1.11	***C4b///LOC102643089***	complement component 4B (Chido blood group)///complement C4-B	0002376 // immune system process // inferred from electronic annotation///0006954 //
NM_001042615///		0.023		-1.07	-1.16	-1.29	-1.14	***Htra3***	HtrA serine peptidase 3	0001558 // regulation of cell growth // inferred from electronic annotation///0006508 //
NM_001166249///		0.048		-1.06	-1.05	-1.29	-1.08	***Mgll***	monoglyceride lipase	0006629 // lipid metabolic process // inferred from electronic annotation///0006631 // fa
NM_0114	0.0388			-1.10	1.08	-1.29	-1.11	***Slc2a3***	solute carrier family 2 (facilitated glucose transporter), member 3	0006810 // transport // inferred from electronic annotation///0008643 // carbohydrate t
NM_018885///XM_		0.016		-1.00	-1.02	-1.29	-1.06	***Irx4***	Iroquois related homeobox 4 (Drosophila)	0006355 // regulation of transcription, DNA-templated // inferred from electronic annota
NM_0529	######	0.027		1.08	-1.37	-1.29	1.26	***Spock2***	sparc/osteonectin, cwcv and kazal-like domains proteoglycan 2	0007165 // signal transduction // inferred from electronic annotation///0010811 // posit
		0.044		-1.01	-1.22	-1.29	-1.11	***D2Ertd63e***	DNA segment, Chr 2, ERATO Doi 63, expressed	
NM_0135	0.0027			-1.28	1.07	-1.29	-1.46	***Mmp9***	matrix metallopeptidase 9	0001501 // skeletal system development // inferred from genetic interaction///0001501 /
NM_011430		0.046		1.05	-1.15	-1.29	1.03	***Sncg***	synuclein, gamma	0007268 // synaptic transmission // inferred from genetic interaction///0008344 // adult
NM_001163577///NM_00			0.024	-1.45	-1.62	-1.29	-1.04	***Prom1***	prominin 1	0010842 // retina layer formation // inferred from mutant phenotype///0045494 // phot
NM_0011	0.0276			-1.10	1.23	-1.29	-1.20	***Lck***	lymphocyte protein tyrosine kinase	0006468 // protein phosphorylation // inferred from direct assay///0006468 // protein p
NM_001033217///		0.016		1.22	1.22	-1.28	1.24	***Prickle1***	prickle homolog 1 (Drosophila)	0001843 // neural tube closure // not recorded///0006606 // protein import into nucleus
NM_133662		0.035		1.15	1.70	-1.27	-1.10	***Ier3***	immediate early response 3	0001562 // response to protozoan // inferred from mutant phenotype///0003085 // nega
NM_0098	0.0371			-1.09	1.23	-1.27	-1.25	***Cd8b1***	CD8 antigen, beta chain 1	0002250 // adaptive immune response // inferred from electronic annotation///0002376
NM_0102	0.0233	0.02	0.039	1.05	-1.16	-1.27	-1.13	***Gast***	gastrin	0007165 // signal transduction // not recorded///0007186 // G-protein coupled receptor
NM_010201///NM		0.005		-1.01	-1.05	-1.27	-1.12	***Fgf14***	fibroblast growth factor 14	0002230 // positive regulation of defense response to virus by host // not recorded///000
NM_0012	0.0224	0.027		1.01	-1.23	-1.27	-1.07	***Aebp1***	AE binding protein 1	0000122 // negative regulation of transcription from RNA polymerase II promoter // not r
NM_018826		0.026		1.01	1.10	-1.27	-1.14	***Irx5***	Iroquois related homeobox 5 (Drosophila)	0002027 // regulation of heart rate // inferred from mutant phenotype///0006351 // tran
NM_001301332///		0.041		1.12	1.29	-1.26	1.12	***Gdnf***	glial cell line derived neurotrophic factor	0001656 // metanephros development // inferred from genetic interaction///0001656 //
NM_010189///XM_		0.017		1.08	1.03	-1.26	1.18	***Fcgrt***	Fc receptor, IgG, alpha chain transporter	0002474 // antigen processing and presentation of peptide antigen via MHC class I // infe
NM_031176///XM_006525			0.047	-1.10	-1.11	-1.26	1.51	***Tnxb***	tenascin XB	0006629 // lipid metabolic process // inferred from mutant phenotype///0006631 // fatt
NM_0114	0.0052	0.007		1.07	-1.23	-1.26	-1.07	***Sprr2i***	small proline-rich protein 2I	0008544 // epidermis development // non-traceable author statement///0018149 // pep
NM_010102		0.045		-1.00	1.11	-1.26	-1.19	***S1pr4***	sphingosine-1-phosphate receptor 4	0003376 // sphingosine-1-phosphate signaling pathway // inferred from electronic annot
NM_010766		0.033		1.12	1.21	-1.26	-1.04	***Marco***	macrophage receptor with collagenous structure	0002376 // immune system process // inferred from electronic annotation///0006897 //
NM_175263///XM_		0.026	0.032	1.04	-1.03	-1.26	-1.16	***Notum***	notum pectinacetylesterase homolog (Drosophila)	0006507 // GPI anchor release // inferred from direct assay///0016055 // Wnt signaling p
NM_0195	######	0.011		1.09	-1.25	-1.26	1.04	***Gas6***	growth arrest specific 6	0001764 // neuron migration // inferred from genetic interaction///0001934 // positive r
NM_007440///XM_		0.02		1.11	-1.04	-1.25	-1.01	***Alox12***	arachidonate 12-lipoxygenase	0006629 // lipid metabolic process // inferred from electronic annotation///0006631 // fa
NM_011902		0.035		1.08	-1.15	-1.25	1.03	***Tekt2***	tektin 2	0030030 // cell projection organization // inferred from electronic annotation///0030317

RefSeq Tr	p 24h	p 48h	p 72h	FD Air	FD 24h	FD 48h	FD 72h	Gene Symbol	Gene Title	Gene Ontology Biological Process
NM_011345///XM_		0.045		-1.10	1.05	-1.25	-1.04	***Sele***	selectin, endothelial cell	0002092 // positive regulation of receptor internalization // not recorded///0002687 // p
NM_001045540///		0.025		-1.05	-1.02	-1.25	1.07	***Gm12185***	predicted gene 12185	
NM_0098	0.0232			-1.25	1.18	-1.25	-1.25	***Cd3g***	CD3 antigen, gamma polypeptide	0007163 // establishment or maintenance of cell polarity // not recorded///0007166 // c
NM_0085	0.0209			-1.17	-1.04	-1.25	-1.29	***Cxcl9***	chemokine (C-X-C motif) ligand 9	0002690 // positive regulation of leukocyte chemotaxis // not recorded///0006935 // che
NM_013585///XM_		0.043		1.08	1.16	-1.25	1.14	***Psmb9***	proteasome (prosome, macropain) subunit, beta type 9 (large multifunctional peptida	0002376 // immune system process // inferred from electronic annotation///0006508 //
NM_001033484///XM_006			0.021	-1.09	-1.09	-1.25	1.09	***Iqgap3***	IQ motif containing GTPase activating protein 3	0000082 // G1/S transition of mitotic cell cycle // inferred from mutant phenotype///000
NM_0532	0.0052	0.003		-1.00	-1.20	-1.25	1.18	***Ces1d***	carboxylesterase 1D	0006629 // lipid metabolic process // inferred from electronic annotation///0006637 // a
NM_172378///XM_		0.043		1.03	-1.14	-1.25	-1.14	***Slc22a22***	solute carrier family 22 (organic cation transporter), member 22	0007186 // G-protein coupled receptor signaling pathway // inferred from direct assay//
NM_0089	0.0357			1.03	-1.31	-1.24	-1.18	***Prkg2***	protein kinase, cGMP-dependent, type II	0006468 // protein phosphorylation // inferred from electronic annotation///0007623 //
NM_053266///XM_		0.026		1.05	-1.07	-1.24	-1.06	***Gtf2ird2***	GTF2I repeat domain containing 2	0006351 // transcription, DNA-templated // inferred from electronic annotation///00063
NM_023850///XM_		0.005		1.13	-1.03	-1.24	-1.06	***Chst1***	carbohydrate (keratan sulfate Gal-6) sulfotransferase 1	0005975 // carbohydrate metabolic process // inferred from electronic annotation///000
NM_0078	0.0342			-1.08	1.30	-1.24	1.10	***Ear1///Ear-ps2///Ear12///Ear2///Ear3***	eosinophil-associated, ribonuclease A family, member 1///eosinophil-associated, ribor	0008152 // metabolic process // inferred from electronic annotation///0090305 // nuclei
XM_9773	0.0287			-1.03	1.47	-1.24	-1.17	***LOC665506///Tcrb-J///Trbc1///Trbc2***	T-cell receptor beta-2 chain C region-like///T cell receptor beta, joining region///T cell receptor beta, constant region 1///T cell receptor beta, constant 2	
NM_010326///XM_		0.029		-1.04	-1.11	-1.24	-1.11	***Gp1ba***	glycoprotein 1b, alpha polypeptide	0000902 // cell morphogenesis // inferred from mutant phenotype///0007155 // cell adh
NM_009999		0.04		1.02	-1.22	-1.24	1.26	***Cyp2b10***	cytochrome P450, family 2, subfamily b, polypeptide 10	0006805 // xenobiotic metabolic process // not recorded///0008202 // steroid metabolic
NR_131197///NR_		0.032		1.05	-1.03	-1.24	-1.03	***Ggnbp2os***	gametogenetin binding protein 2, opposite strand	
NM_026514///XM_		0.003		1.03	-1.08	-1.24	-1.08	***Cdc42ep3***	CDC42 effector protein (Rho GTPase binding) 3	0007266 // Rho protein signal transduction // not recorded///0008360 // regulation of ce
NM_134110///XM_00652			0.024	-1.33	-1.34	-1.24	1.14	***Kcne2***	potassium voltage-gated channel, Isk-related subfamily, gene 2	0006810 // transport // inferred from electronic annotation///0006811 // ion transport /
NM_009994///XM_		0.037		1.04	-1.00	-1.23	-1.09	***Cyp1b1***	cytochrome P450, family 1, subfamily b, polypeptide 1	0001525 // angiogenesis // inferred from mutant phenotype///0002930 // trabecular me
NR_028574			0.034	1.03	1.04	-1.23	-1.09	***Snhg8***	small nucleolar RNA host gene 8	
NM_023168///XM_		0.008		1.00	1.03	-1.23	1.05	***Grina***	glutamate receptor, ionotropic, N-methyl D-aspartate-associated protein 1 (glutamate	0032469 // endoplasmic reticulum calcium ion homeostasis // inferred from direct assay/
NM_0011	0.0132			-1.06	1.02	-1.23	-1.07	***Tshr***	thyroid stimulating hormone receptor	0007165 // signal transduction // inferred from electronic annotation///0007186 // G-pro
NM_019431		0.003		1.04	-1.01	-1.23	-1.03	***Cacng4***	calcium channel, voltage-dependent, gamma subunit 4	0006810 // transport // inferred from electronic annotation///0006811 // ion transport /
NM_0010	0.0456	0.007		1.03	-1.14	-1.23	-1.05	***Calca***	calcitonin/calcitonin-related polypeptide, alpha	0001976 // neurological system process involved in regulation of systemic arterial blood p
NM_031377///XM_		0.008		1.04	-1.04	-1.23	-1.08	***Pramel1***	preferentially expressed antigen in melanoma-like 1	0008284 // positive regulation of cell proliferation // inferred from electronic annotation,
XM_9773	0.0133			-1.05	1.45	-1.23	-1.17	***LOC665506///Trbc2///Tcrb-J///Trbc1***	T-cell receptor beta-2 chain C region-like///T cell receptor beta, constant 2///T cell receptor beta, joining region///T cell receptor beta, constant region 1	
NM_0280	0.0151	0.007	#####	1.12	-1.09	-1.23	-1.24	***Cyp2c55***	cytochrome P450, family 2, subfamily c, polypeptide 55	0006805 // xenobiotic metabolic process // not recorded///0019369 // arachidonic acid m
NM_001253692///		0.002		-1.03	-1.01	-1.23	1.06	***Elmod3***	ELMO/CED-12 domain containing 3	
NM_0010	0.041			1.01	-1.24	-1.23	-1.18	***Sepp1***	selenoprotein P, plasma, 1	0001887 // selenium compound metabolic process // inferred from direct assay///00018
NM_172584		0.007		1.04	-1.04	-1.23	-1.04	***Itpk1***	inositol 1,3,4-triphosphate 5/6 kinase	0016310 // phosphorylation // inferred from electronic annotation///0032957 // inositol
NM_001160163///		0.034		1.13	-1.02	-1.23	-1.05	***Neu2***	neuraminidase 2	0005975 // carbohydrate metabolic process // inferred from electronic annotation///000
NM_0012	0.0127			-1.21	1.24	-1.23	-1.21	***Csf3r***	colony stimulating factor 3 receptor (granulocyte)	0007155 // cell adhesion // inferred from electronic annotation///0019221 // cytokine-m
NM_001083959///		0.028	0.018	1.07	-1.06	-1.23	-1.16	***Spo11***	SPO11 meiotic protein covalently bound to DSB	0000237 // leptotene // inferred from mutant phenotype///0000737 // DNA catabolic pr
NM_0010	0.0157			-1.12	-1.57	-1.22	1.01	***Otud7b***	OTU domain containing 7B	0000122 // negative regulation of transcription from RNA polymerase II promoter // infer
NM_0011	0.0043	0.004	0.032	1.03	-1.11	-1.22	-1.13	***Smoc1***	SPARC related modular calcium binding 1	0001654 // eye development // inferred from mutant phenotype///0001654 // eye devel
NM_008256		0.009	0.012	1.24	1.05	-1.22	-1.09	***Hmgcs2***	3-hydroxy-3-methylglutaryl-Coenzyme A synthase 2	0001822 // kidney development // inferred from electronic annotation///0001889 // live
NM_0268	0.0175	0.011		1.01	1.24	-1.22	-1.22	***Cd177***	CD177 antigen	
NM_0097	0.0184			-1.04	-1.32	-1.22	1.12	***Aspm***	asp (abnormal spindle)-like, microcephaly associated (Drosophila)	0001764 // neuron migration // inferred from genetic interaction///0001764 // neuron m
XM_9773	0.0143			-1.05	1.39	-1.22	-1.16	***LOC665506///Trbc2///Tcrb-J///Trbc1///Trb***	T-cell receptor beta-2 chain C region-like///T cell receptor beta, constant 2///T cell receptor beta, joining region///T cell receptor beta, constant region 1///T cell receptor beta,	
	0.0049			-1.15	1.71	-1.22	-1.15	***Iglv1///Iglc1***	immunoglobulin lambda variable 1///immunoglobulin lambda constant 1	
NR_003964		0.006		1.03	-1.06	-1.22	-1.14	***Tubb2a-ps2***	tubulin, beta 2a, pseudogene 2	
NM_1460	0.0038	0.008		1.06	-1.16	-1.22	-1.07	***Zdhhc14***	zinc finger, DHHC domain containing 14	0006612 // protein targeting to membrane // ---///0018230 // peptidyl-L-cysteine S-palm
NM_153067		0.049		1.01	-1.23	-1.22	-1.11	***Mrgpra3***	MAS-related GPR, member A3	0006953 // acute-phase response // inferred from electronic annotation///0007165 // sig
NM_001308501///		0.023		1.11	-1.04	-1.21	-1.22	***Gpr50***	G-protein-coupled receptor 50	0007165 // signal transduction // inferred from electronic annotation///0007166 // cell s
NM_0011	0.0485			-1.10	1.12	-1.21	-1.08	***Ccr6***	chemokine (C-C motif) receptor 6	0006935 // chemotaxis // inferred from electronic annotation///0006955 // immune resp
NM_0012	0.0408	0.015		1.07	-1.13	-1.21	-1.04	***Ebf2***	early B cell factor 2	0001709 // cell fate determination // inferred from direct assay///0006351 // transcriptio
NM_0013	0.0309	0.005	0.03	1.11	-1.04	-1.21	-1.11	***Stmn4***	stathmin-like 4	0007019 // microtubule depolymerization // not recorded///0031110 // regulation of mi
NM_001082531///		0.048		1.01	-1.07	-1.21	-1.09	***Pla2g2a***	phospholipase A2, group IIA (platelets, synovial fluid)	0006629 // lipid metabolic process // inferred from electronic annotation///0006644 // p
NM_138683///XM_		0.004		1.21	-1.05	-1.21	-1.09	***Rspo1***	R-spondin 1	0001934 // positive regulation of protein phosphorylation // not recorded///0002090 // r
NM_030720///XM_		0.017		1.03	1.12	-1.21	-1.08	***Gpr84***	G protein-coupled receptor 84	0007165 // signal transduction // inferred from electronic annotation///0007186 // G-pro
NM_1336	0.0271			1.13	-1.45	-1.21	1.06	***Sult1a1***	sulfotransferase family 1A, phenol-preferring, member 1	0006584 // catecholamine metabolic process // not recorded///0006629 // lipid metabol
NM_001013373///		0.031		-1.04	-1.12	-1.21	-1.18	***Tmprss13***	transmembrane protease, serine 13	0006508 // proteolysis // inferred from electronic annotation///0006898 // receptor-med
NM_0010	0.0433	0.04		1.03	-1.07	-1.21	-1.12	***Arsi***	arylsulfatase i	0008152 // metabolic process // inferred from electronic annotation
NM_178086		0.009	0.002	1.03	-1.10	-1.21	-1.23	***Fa2h***	fatty acid 2-hydroxylase	0001949 // sebaceous gland cell differentiation // inferred from mutant phenotype///000
NM_007574		0.02		-1.00	-1.05	-1.21	-1.05	***C1qc***	complement component 1, q subcomponent, C chain	0002376 // immune system process // inferred from electronic annotation///0006958 //
NM_0011	0.0159			1.15	-1.40	-1.21	1.01	***Sphk1***	sphingosine kinase 1	0001568 // blood vessel development // inferred from genetic interaction///0001934 // p
NM_133667///XM_		0.008		1.14	-1.00	-1.21	1.11	***Pdk2***	pyruvate dehydrogenase kinase, isoenzyme 2	0005975 // carbohydrate metabolic process // inferred from electronic annotation///000
NM_183171///XM_		0.042	0.04	1.07	-1.04	-1.21	-1.14	***Fez1***	fasciculation and elongation protein zeta 1 (zygin I)	0006810 // transport // inferred from electronic annotation///0010976 // positive regula
NM_0012	0.03			-1.09	-1.21	-1.21	-1.01	***Gsn***	gelsolin	0006911 // phagocytosis, engulfment // inferred from mutant phenotype///0006915 // a
NM_013751///XM_		0.041		-1.05	1.01	-1.21	-1.13	***Hrasls***	HRAS-like suppressor	0001558 // regulation of cell growth // traceable author statement///0006629 // lipid me
NM_145364///XM_		0.049		-1.05	1.01	-1.21	-1.14	***Akr1d1***	aldo-keto reductase family 1, member D1	0006629 // lipid metabolic process // inferred from electronic annotation///0006699 // b
NM_010180///XM_00652			0.003	-1.27	-1.22	-1.21	-1.03	***Fbln1***	fibulin 1	0001933 // negative regulation of protein phosphorylation // not recorded///0007162 //
NM_001165256///		0.003	0.046	1.16	1.07	-1.20	-1.06	***Dcaf4***	DDB1 and CUL4 associated factor 4	
NM_0012	0.0324			1.01	-1.19	-1.20	1.07	***Myh14***	myosin, heavy polypeptide 14	0003009 // skeletal muscle contraction // not recorded///0007519 // skeletal muscle tiss

RefSeq Tr	*p* 24h	*p* 48h	*p* 72h	FD Air	FD 24h	FD 48h	FD 72h	Gene Symbol	Gene Title	Gene Ontology Biological Process
		0.004		-1.04	-1.05	-1.20	-1.20	***Tcrg-C4***	T cell receptor gamma, constant 4	
NM_001033263///		0.032		1.09	-1.01	-1.20	-1.12	***Agap2***	ArfGAP with GTPase domain, ankyrin repeat and PH domain 2	0007264 // small GTPase mediated signal transduction // inferred from electronic annota
NM_0013	0.0134	0.01		1.12	-1.19	-1.20	-1.17	***Ctf1***	cardiotrophin 1	0007166 // cell surface receptor signaling pathway // not recorded///0007399 // nervous
NM_013638		0.025		1.02	1.02	-1.20	-1.05	***Prm3***	protamine 3	0007275 // multicellular organismal development // inferred from electronic annotation/
NM_0013	0.0364			-1.01	1.22	-1.20	-1.16	***Irf8***	interferon regulatory factor 8	0006351 // transcription, DNA-templated // inferred from electronic annotation///00063
NM_001286015///		0.033		1.04	-1.10	-1.20	-1.03	***Csn1s1***	casein alpha s1	0006810 // transport // inferred from electronic annotation
NM_001170638///		0.017		1.10	-1.02	-1.20	-1.02	***Slc17a1***	solute carrier family 17 (sodium phosphate), member 1	0006810 // transport // inferred from electronic annotation///0006811 // ion transport /
NM_029901		0.025		1.01	-1.05	-1.20	-1.13	***Akr1c21***	aldo-keto reductase family 1, member C21	0006629 // lipid metabolic process // inferred from electronic annotation///0006694 // s
NM_148940///XM		0.019	0.016	-1.03	-1.08	-1.20	-1.18	***Prss44***	protease, serine 44	0006508 // proteolysis // inferred from electronic annotation///0007281 // germ cell dev
NM_0137	0.0242	0.005		1.08	-1.17	-1.20	-1.04	***Slco1a1***	solute carrier organic anion transporter family, member 1a1	0006810 // transport // inferred from electronic annotation///0006811 // ion transport /
NM_0010	0.0498			-1.20	-1.47	-1.20	-1.10	***Hspa2***	heat shock protein 2	0001934 // positive regulation of protein phosphorylation // inferred from mutant phenc
NM_0097	0.0019			1.09	-1.32	-1.20	1.06	***Bnip3***	BCL2/adenovirus E1B interacting protein 3	0001666 // response to hypoxia // not recorded///0006915 // apoptotic process // not re
NM_008993///XM		0.032		-1.06	-1.12	-1.20	-1.15	***Pxmp2***	peroxisomal membrane protein 2	
NM_145077		0.011	0.019	1.10	-1.04	-1.20	-1.21	***Ucn2***	urocortin 2	0006171 // cAMP biosynthetic process // inferred from sequence or structural similarity/,
NM_009396///XM		0.005		1.03	1.09	-1.20	1.02	***Tnfaip2***	tumor necrosis factor, alpha-induced protein 2	0001525 // angiogenesis // inferred from electronic annotation///0006887 // exocytosis ,
NM_139197///XM		0.032		1.03	1.00	-1.20	-1.14	***Gbgt1***	globoside alpha-1,3-N-acetylgalactosaminyltransferase 1	0005975 // carbohydrate metabolic process // inferred from electronic annotation///000
NM_010781///XM		0.008	0.015	1.05	1.02	-1.20	-1.17	***Tpsb2***	tryptase beta 2	0006508 // proteolysis // inferred from electronic annotation///0006954 // inflammatory
NM_019485		0.045		1.05	-1.10	-1.19	-1.08	***Olfr70***	olfactory receptor 70	0007165 // signal transduction // inferred from electronic annotation///0007186 // G-pro
NM_0134	######	0.01		1.04	-1.20	-1.19	1.03	***C2***	complement component 2 (within H-2S)	0002376 // immune system process // inferred from electronic annotation///0006508 //
NM_023617///XM		0.002		1.11	1.04	-1.19	1.23	***Aox3***	aldehyde oxidase 3	0055114 // oxidation-reduction process // inferred from electronic annotation
NM_008780///XM		0.04		-1.01	-1.05	-1.19	-1.11	***Pax1***	paired box 1	0001501 // skeletal system development // inferred from genetic interaction///0001501 ,
NM_025777		0.017		1.03	1.00	-1.19	-1.13	***Duoxa2***	dual oxidase maturation factor 2	0006810 // transport // inferred from electronic annotation///0015031 // protein transpo
NM_1461	0.0226			-1.20	1.07	-1.19	-1.02	***Gimap7***	GTPase, IMAP family member 7	0008152 // metabolic process // inferred from electronic annotation
NM_145827///XM		0.021		1.01	1.01	-1.19	-1.04	***Nlrp3***	NLR family, pyrin domain containing 3	0002376 // immune system process // inferred from electronic annotation///0002674 //
NM_010951///XM		0.004		1.00	-1.11	-1.19	-1.09	***Gpr143***	G protein-coupled receptor 143	0007165 // signal transduction // inferred from electronic annotation///0007186 // G-pro
NM_032465		0.045		1.04	-1.00	-1.19	-1.15	***Cd96***	CD96 antigen	0007155 // cell adhesion // inferred from electronic annotation
NM_001310464///		0.033		1.12	-1.12	-1.19	1.01	***Reln***	reelin	0000904 // cell morphogenesis involved in differentiation // inferred from mutant pheno
NM_145229		0.011		-1.01	1.01	-1.19	-1.16	***AY074887***	cDNA sequence AY074887	0007050 // cell cycle arrest // inferred from direct assay///0008285 // negative regulation
NM_053224			0.018	-1.07	-1.14	-1.19	-1.33	***Vmn1r54***	vomeronasal 1 receptor 54	0007165 // signal transduction // inferred from electronic annotation///0007186 // G-pro
NM_0134	0.0425			1.00	-1.14	-1.19	-1.11	***Bcl2l10***	Bcl2-like 10	0006915 // apoptotic process // inferred from electronic annotation///0008625 // extrins
NM_001080809///		0.042		1.00	-1.18	-1.19	-1.15	***Cps1***	carbamoyl-phosphate synthetase 1	0000050 // urea cycle // not recorded///0000050 // urea cycle // inferred by curator///0
NM_008344		0.026		1.26	1.09	-1.19	-1.07	***Igfbp6***	insulin-like growth factor binding protein 6	0001558 // regulation of cell growth // inferred from electronic annotation///0043567 //
NM_0083	0.0207			1.26	1.09	-1.19	-1.07	***Igfbp6***	insulin-like growth factor binding protein 6	0001558 // regulation of cell growth // inferred from electronic annotation///0043567 //
NM_0010	0.0227			-1.10	1.39	-1.19	1.12	***Ear-ps2///Ear1///Ear12///Ear2///Ear3***	eosinophil-associated, ribonuclease A family, pseudogene 2///eosinophil-associated, r	0006935 // chemotaxis // inferred from direct assay///0008152 // metabolic process // ir
NM_013666///NM		0.012		-1.00	-1.02	-1.19	-1.13	***St8sia5***	ST8 alpha-N-acetyl-neuraminide alpha-2,8-sialyltransferase 5	0006486 // protein glycosylation // inferred from electronic annotation///0097503 // sial
NM_0082	0.0078			-1.06	1.34	-1.19	-1.18	***H2-Oa***	histocompatibility 2, O region alpha locus	0002376 // immune system process // inferred from electronic annotation///0002504 //
NM_001160406///		0.039		-1.04	-1.02	-1.19	-1.12	***Gfi1b***	growth factor independent 1B	0000122 // negative regulation of transcription from RNA polymerase II promoter // not
NM_013616			#####	-1.03	1.04	-1.19	-1.27	***Olfr65***	olfactory receptor 65	0007165 // signal transduction // not recorded///0007186 // G-protein coupled receptor
NM_010468		0.035		1.01	1.06	-1.19	-1.10	***Hoxd3***	homeobox D3	0006351 // transcription, DNA-templated // not recorded///0006355 // regulation of trar
NM_001282040///NM_00			0.041	1.08	1.06	-1.18	1.17	***Pmm1***	phosphomannomutase 1	0006013 // mannose metabolic process // not recorded///0008152 // metabolic process
NM_001163270///		0.027		1.06	-1.00	-1.18	-1.12	***Baiap3***	BAI1-associated protein 3	0007186 // G-protein coupled receptor signaling pathway // not recorded
NM_2015	0.0434			-1.07	-1.27	-1.18	1.05	***Lmo7***	LIM domain only 7	0016337 // single organismal cell-cell adhesion // not recorded///0023051 // regulation c
NM_1530	0.0434			1.04	-1.12	-1.18	-1.10	***Hrh4***	histamine receptor H4	0006954 // inflammatory response // not recorded///0006954 // inflammatory response
NM_0107	0.0034	0.008		1.10	-1.09	-1.18	-1.10	***Lgals4***	lectin, galactose binding, soluble 4	
NM_133922///XM		0.045		1.01	1.06	-1.18	-1.08	***Krba1***	KRAB-A domain containing 1	
NM_015732///XM		0.049		1.01	-1.09	-1.18	-1.09	***Axin2***	axin2	0001756 // somitogenesis // inferred from direct assay///0001934 // positive regulation c
NM_0011	0.0021	0.014	0.009	1.04	-1.33	-1.18	-1.22	***Ccl20***	chemokine (C-C motif) ligand 20	0002548 // monocyte chemotaxis // ---///0006935 // chemotaxis // not recorded///0006
NM_026410			0.037	-1.14	1.05	-1.18	1.04	***Cdca5***	cell division cycle associated 5	0000082 // G1/S transition of mitotic cell cycle // not recorded///0006302 // double-stra
NM_0196	0.0329			-1.01	1.40	-1.18	-1.21	***Rrad***	Ras-related associated with diabetes	0007165 // signal transduction // inferred from electronic annotation///0007264 // smal
NM_001277847///		0.045		1.09	-1.05	-1.18	-1.10	***Treh***	trehalase (brush-border membrane glycoprotein)	0005991 // trehalose metabolic process // inferred from electronic annotation///000599
NM_0011	0.0226			-1.01	-1.36	-1.18	-1.02	***Vldlr***	very low density lipoprotein receptor	0000122 // negative regulation of transcription from RNA polymerase II promoter // infer
NM_001199971///		0.017		1.04	-1.02	-1.18	-1.11	***Tac2***	tachykinin 2	0007217 // tachykinin receptor signaling pathway // inferred from electronic annotation/
NM_026680///NM		0.004		1.03	-1.08	-1.18	-1.02	***Golt1a///Kiss1***	golgi transport 1 homolog A (S. cerevisiae)///KiSS-1 metastasis-suppressor	0006629 // lipid metabolic process // not recorded///0006810 // transport // inferred fro
NM_0013	0.0492	0.034		1.02	-1.11	-1.18	-1.12	***Gpr83***	G protein-coupled receptor 83	0007165 // signal transduction // inferred from electronic annotation///0007166 // cell s
NM_011680///XM		0.01	0.022	1.06	1.01	-1.18	-1.10	***Usf2***	upstream transcription factor 2	0000432 // positive regulation of transcription from RNA polymerase II promoter by gluco
NM_001291065///		0.022		1.05	1.02	-1.18	-1.08	***Foxa2***	forkhead box A2	0000432 // positive regulation of transcription from RNA polymerase II promoter by gluco
NM_008571///XM		0.015		-1.04	-1.03	-1.18	-1.20	***Mcpt2***	mast cell protease 2	0006508 // proteolysis // inferred from electronic annotation///0016485 // protein proce
NM_011172///XM		0.027		1.13	1.04	-1.18	-1.05	***Prodh***	proline dehydrogenase	0006560 // proline metabolic process // inferred from electronic annotation///0006562 /
NM_001168498///		0.044		1.03	-1.09	-1.18	-1.09	***Ncmap***	noncompact myelin associated protein	0031643 // positive regulation of myelination // not recorded///0032290 // peripheral ne
NM_0011	0.0321			-1.27	1.01	-1.18	-1.33	***Clec4d***	C-type lectin domain family 4, member d	0002250 // adaptive immune response // inferred from mutant phenotype///0002292 //
NM_013750		0.039		1.02	-1.11	-1.18	-1.13	***Phlda3***	pleckstrin homology-like domain, family A, member 3	0006915 // apoptotic process // inferred from electronic annotation///0042771 // intrins
NM_001001892///		0.041		-1.07	1.05	-1.18	-1.08	***H2-K1///H2-D1***	histocompatibility 2, K1, K region///histocompatibility 2, D region locus 1///H-2 class I	0001916 // positive regulation of T cell mediated cytotoxicity // inferred from direct assay
NM_133831		0.046		1.10	1.14	-1.18	-1.20	***Gltscr2***	glioma tumor suppressor candidate region gene 2	
NM_0169	0.0032			-1.06	1.30	-1.18	-1.24	***Ptprcap***	protein tyrosine phosphatase, receptor type, C polypeptide-associated protein	0006468 // protein phosphorylation // inferred from direct assay///0006468 // protein p

RefSeq Tr	*p* 24h	*p* 48h	*p* 72h	FD Air	FD 24h	FD 48h	FD 72h	Gene Symbol	Gene Title	Gene Ontology Biological Process
NM_153097		0.033		1.06	-1.10	-1.18	-1.08	***Trim60***	tripartite motif-containing 60	
NM_0540	0.0317			-1.02	-1.18	-1.18	-1.07	***Pnpla3***	patatin-like phospholipase domain containing 3	0006629 // lipid metabolic process // inferred from electronic annotation///0008152 // m
NM_001031622///NM_00			0.047	1.00	-1.05	-1.18	-1.20	***Gm16367///Gm16427///Gm16513///Gm3***	predicted gene 16367///predicted gene 16427///predicted gene, Gm16513///predicte	0008284 // positive regulation of cell proliferation // inferred from electronic annotation/
NM_0099	0.0187			-1.16	-1.04	-1.18	-1.19	***Cst7***	cystatin F (leukocystatin)	0010466 // negative regulation of peptidase activity // inferred from electronic annotatio
NM_0103	######		0.02	-1.31	1.01	-1.18	-1.16	***H2-Q10***	histocompatibility 2, Q region locus 10	0002376 // immune system process // inferred from electronic annotation///0002474 //
NM_019540		0.027		1.01	-1.12	-1.18	-1.10	***Pfpl***	pore forming protein-like	
NM_153789		0.017		1.22	1.30	-1.18	1.07	***Mylip***	myosin regulatory light chain interacting protein	0006928 // movement of cell or subcellular component // not recorded///0007399 // ne
NM_009800///XM		0.044		-1.02	-1.09	-1.18	-1.10	***Car11***	carbonic anhydrase 11	
NM_001077696///		0.047		1.09	1.15	-1.18	1.13	***Hdac5***	histone deacetylase 5	0000122 // negative regulation of transcription from RNA polymerase II promoter // infer
NM_001190357///		0.034		1.04	-1.02	-1.17	-1.12	***Carkd***	carbohydrate kinase domain containing	0008152 // metabolic process // inferred from electronic annotation
NM_0205	0.0418			1.14	-1.04	-1.17	1.05	***Slc25a20***	solute carrier family 25 (mitochondrial carnitine/acylcarnitine translocase), member 2	0006810 // transport // inferred from electronic annotation
NM_021287///XM		0.049		1.04	-1.08	-1.17	-1.09	***Sptbn2***	spectrin beta, non-erythrocytic 2	0007416 // synapse assembly // inferred from mutant phenotype///0016079 // synaptic
NM_130903///XM		0.047		-1.03	-1.05	-1.17	-1.16	***Cd209c***	CD209c antigen	
NM_001252565///		#####	0.009	1.02	-1.02	-1.17	-1.14	***Oprl1***	opioid receptor-like 1	0001934 // positive regulation of protein phosphorylation // not recorded///0007165 // s
NM_0118	0.0355	0.026		1.05	-1.01	-1.17	-1.08	***3-Sep***	septin 3	0007049 // cell cycle // inferred from electronic annotation///0051301 // cell division // i
NM_0254	0.0063			1.03	-1.65	-1.17	-1.05	***Rgcc***	regulator of cell cycle	0000082 // G1/S transition of mitotic cell cycle // not recorded///0001100 // negative reg
NM_018795///XM		0.019	0.01	1.09	1.02	-1.17	-1.20	***Abcc6***	ATP-binding cassette, sub-family C (CFTR/MRP), member 6	0006810 // transport // inferred from electronic annotation///0008152 // metabolic proc
NM_0083	0.0035	0.001		1.10	-1.14	-1.17	-1.08	***Htr2b***	5-hydroxytryptamine (serotonin) receptor 2B	0001755 // neural crest cell migration // inferred from mutant phenotype///0001819 // p
NM_139299///XM_00651			0.001	-1.02	-1.07	-1.17	-1.29	***Il31ra***	interleukin 31 receptor A	0002067 // glandular epithelial cell differentiation // inferred from mutant phenotype///
NM_0011	0.0369			-1.00	1.18	-1.17	1.07	***Pparg***	peroxisome proliferator activated receptor gamma	0000122 // negative regulation of transcription from RNA polymerase II promoter // infer
NM_010630///XM		0.038		1.04	-1.01	-1.17	-1.00	***Kifc2***	kinesin family member C2	0007018 // microtubule-based movement // not recorded///0008152 // metabolic proce
NM_0074	0.0479		0.029	1.02	-1.12	-1.17	-1.17	***Aqp7***	aquaporin 7	0006810 // transport // inferred from electronic annotation///0006833 // water transpor
NM_027983			0.002	-1.03	-1.01	-1.17	-1.29	***Krt33a***	keratin 33A	
NM_0079	0.0212			1.05	-1.27	-1.17	1.12	***Ereg***	epiregulin	0000187 // activation of MAPK activity // not recorded///0001525 // angiogenesis // infe
NM_0013	0.0348			1.02	-1.15	-1.17	1.20	***Adamtsl4***	ADAMTS-like 4	0006508 // proteolysis // inferred from electronic annotation///0006915 // apoptotic pro
NM_008340///XM		0.028		-1.00	-1.03	-1.17	-1.14	***Igfals***	insulin-like growth factor binding protein, acid labile subunit	0007155 // cell adhesion // inferred from electronic annotation
NM_007699///XM_00649			0.046	-1.00	-1.04	-1.17	-1.19	***Chrm4***	cholinergic receptor, muscarinic 4	0007165 // signal transduction // inferred from electronic annotation///0007186 // G-pro
NM_001167691///		0.04		1.03	-1.03	-1.17	-1.09	***Sirt4***	sirtuin 4	0000820 // regulation of glutamine family amino acid metabolic process // inferred from
NM_001310639///		0.011		1.09	1.28	-1.17	-1.10	***Stap1***	signal transducing adaptor family member 1	0007169 // transmembrane receptor protein tyrosine kinase signaling pathway // inferre
NM_010670			0.043	1.02	1.02	-1.17	-1.05	***Krtap12-1***	keratin associated protein 12-1	
NM_011741///XM		0.001		-1.01	-1.05	-1.17	-1.12	***Zan***	zonadhesin	0007155 // cell adhesion // inferred from electronic annotation///0007339 // binding of s
NM_0092	0.0358			1.10	-1.08	-1.17	-1.17	***Sts***	steroid sulfatase	0006629 // lipid metabolic process // inferred from electronic annotation///0007565 // fe
NM_0082	0.0012			1.09	1.65	-1.17	-1.01	***Hhex***	hematopoietically expressed homeobox	0000122 // negative regulation of transcription from RNA polymerase II promoter // infer
NM_025987		0.035		1.05	1.01	-1.17	-1.09	***Ndufa6***	NADH dehydrogenase (ubiquinone) 1 alpha subcomplex, 6 (B14)	0006810 // transport // inferred from electronic annotation///0006979 // response to ox
NM_1460	0.0166			1.05	-1.21	-1.17	-1.01	***Sec14l4***	SEC14-like lipid binding 4	0006810 // transport // inferred from electronic annotation
NM_0103	0.0415			1.06	-1.07	-1.17	1.18	***Grb7***	growth factor receptor bound protein 7	0007165 // signal transduction // traceable author statement///0009967 // positive regu
NM_016863///XM		0.011		1.09	1.02	-1.17	-1.15	***Fkbp1b***	FK506 binding protein 1b	0000413 // protein peptidyl-prolyl isomerization // not recorded///0002027 // regulation
NM_001039530///		0.038		1.07	1.04	-1.17	1.13	***Parp14***	poly (ADP-ribose) polymerase family, member 14	0006351 // transcription, DNA-templated // inferred from electronic annotation///00063
NM_0098	0.0413	0.014		1.02	-1.12	-1.17	-1.16	***Entpd2***	ectonucleoside triphosphate diphosphohydrolase 2	0007186 // G-protein coupled receptor signaling pathway // inferred from direct assay//
NM_053122		0.02		1.08	-1.01	-1.17	-1.09	***Immp2l***	IMP2 inner mitochondrial membrane peptidase-like (S. cerevisiae)	0001541 // ovarian follicle development // inferred from mutant phenotype///0006465 /
NM_033037///XR_		0.033		1.11	-1.00	-1.17	-1.04	***Cdo1***	cysteine dioxygenase 1, cytosolic	0007595 // lactation // not recorded///0010243 // response to organonitrogen compour
NM_001002927///		0.042		1.07	-1.06	-1.17	-1.06	***Penk***	preproenkephalin	0001662 // behavioral fear response // inferred from mutant phenotype///0001964 // st
NM_013491///XM		0.05		1.06	-1.02	-1.17	-1.06	***Clcn1***	chloride channel, voltage-sensitive 1	0006810 // transport // inferred from electronic annotation///0006811 // ion transport /
	0.0035			-1.07	1.24	-1.17	-1.22	***Igkv3-12///Igkv3-7***	immunoglobulin kappa variable 3-12///immunoglobulin kappa variable 3-7	0006898 // receptor-mediated endocytosis // ---///0006910 // phagocytosis, recognition
NM_0097	0.0197	0.004		1.10	-1.15	-1.17	1.05	***Chic1***	cysteine-rich hydrophobic domain 1	0006810 // transport // not recorded
NM_001252662///		0.042		1.04	1.06	-1.17	-1.02	***Dmtn***	dematin actin binding protein	0006461 // protein complex assembly // inferred from direct assay///0006461 // protein
NM_145377///XM		0.01		1.04	-1.06	-1.17	-1.03	***Trim41***	tripartite motif-containing 41	0016567 // protein ubiquitination // inferred from electronic annotation
NM_133191///XM		0.037		1.03	-1.00	-1.17	1.12	***Eps8l2***	EPS8-like 2	0007266 // Rho protein signal transduction // not recorded///0016601 // Rac protein sig
NM_001310680///		0.009		1.01	-1.02	-1.17	-1.01	***Scg2***	secretogranin II	0000165 // MAPK cascade // not recorded///0000165 // MAPK cascade // inferred from
NM_001199123///		0.017		-1.00	-1.11	-1.16	-1.13	***Spc25***	SPC25, NDC80 kinetochore complex component, homolog (S. cerevisiae)	0007049 // cell cycle // inferred from electronic annotation///0007052 // mitotic spindle
NM_0273	0.0471			1.02	-1.24	-1.16	-1.08	***Iyd***	iodotyrosine deiodinase	0055114 // oxidation-reduction process // inferred from electronic annotation
NM_001122949///		0.01		1.02	-1.03	-1.16	-1.09	***Mpl***	myeloproliferative leukemia virus oncogene	0010628 // positive regulation of gene expression // inferred from mutant phenotype///
NM_008824///XM		0.034		1.04	-1.04	-1.16	-1.04	***Pfkfb1***	6-phosphofructo-2-kinase/fructose-2,6-biphosphatase 1	0006000 // fructose metabolic process // inferred from electronic annotation///0006003
NM_1768	0.026			-1.02	-1.22	-1.16	1.07	***Osbpl11***	oxysterol binding protein-like 11	0006810 // transport // inferred from electronic annotation///0006869 // lipid transport
NM_0012	0.0073			-1.14	-1.44	-1.16	-1.16	***St7l***	suppression of tumorigenicity 7-like	0030198 // extracellular matrix organization // non-traceable author statement///00303
NM_1531	0.0392			-1.01	-1.30	-1.16	1.20	***Abca8a***	ATP-binding cassette, sub-family A (ABC1), member 8a	0006810 // transport // inferred from electronic annotation///0006869 // lipid transport
NM_0269	0.0417	0.007	0.023	1.07	-1.06	-1.16	-1.14	***Zg16***	zymogen granule protein 16	0006810 // transport // inferred from electronic annotation///0015031 // protein transp
NM_031172///XM		0.037		1.03	1.02	-1.16	-1.12	***Trim17///Hist3h2a***	tripartite motif-containing 17///histone cluster 3, H2a	0006914 // autophagy // not recorded///0016567 // protein ubiquitination // inferred fro
NM_0012	0.0111			-1.11	1.01	-1.16	-1.18	***Dmc1***	DMC1 dosage suppressor of mck1 homolog, meiosis-specific homologous recombinati	0000730 // DNA recombinase assembly // not recorded///0001541 // ovarian follicle dev
NM_008154		0.028		1.04	-1.04	-1.16	-1.12	***Gpr3***	G-protein coupled receptor 3	0007165 // signal transduction // inferred from electronic annotation///0007186 // G-pro
NM_0011	0.0016	#####		1.06	-1.09	-1.16	-1.06	***Kif17***	kinesin family member 17	0006810 // transport // inferred from electronic annotation///0007017 // microtubule-ba
NM_021409		0.004		1.13	1.06	-1.16	1.11	***Pard6b***	par-6 family cell polarity regulator beta	0006461 // protein complex assembly // not recorded///0007049 // cell cycle // inferred
NM_025347///NM		0.008		1.14	1.16	-1.16	-1.07	***Ypel3***	yippee-like 3 (Drosophila)	0006915 // apoptotic process // inferred from electronic annotation///2000774 // positiv
NM_0010	0.0369			1.06	-1.12	-1.16	-1.02	***Rusc2***	RUN and SH3 domain containing 2	

RefSeq Tr	*p* 24h	*p* 48h	*p* 72h	FD Air	FD 24h	FD 48h	FD 72h	Gene Symbol	Gene Title	Gene Ontology Biological Process
NM_026419///XM_		0.042		-1.01	-1.08	-1.16	-1.17	***Cela3b***	chymotrypsin-like elastase family, member 3B	0006508 // proteolysis // not recorded///0008203 // cholesterol metabolic process // no
NM_001290561///		0.004	0.005	1.02	-1.06	-1.16	-1.19	***Plp1***	proteolipid protein (myelin) 1	0006954 // inflammatory response // inferred from genetic interaction///0007229 // inte
NM_001290680///		0.045	0.005	1.09	1.08	-1.16	-1.11	***Nsg2***	neuron specific gene family member 2	0007212 // dopamine receptor signaling pathway // inferred from electronic annotation/
XM_9773	0.0345			-1.04	1.32	-1.16	-1.17	***Tcrb-J///LOC665506///Trbc1///Trbc2***	T cell receptor beta, joining region///T-cell receptor beta-2 chain C region-like///T cell receptor beta, constant region 1///T cell receptor beta, constant 2	
NM_026818		0.03	0.035	1.12	-1.05	-1.16	-1.14	***Cilp2***	cartilage intermediate layer protein 2	
NM_0194	0.0164			1.03	-1.17	-1.16	1.03	***Hgfac***	hepatocyte growth factor activator	0006508 // proteolysis // inferred from electronic annotation
NM_1733	0.0086			-1.11	1.05	-1.16	-1.17	***Xkr6***	X Kell blood group precursor related family member 6 homolog	
NM_0218	0.0077		0.025	-1.06	-1.44	-1.16	1.09	***Tmod1***	tropomodulin 1	0006936 // muscle contraction // inferred from mutant phenotype///0007015 // actin fil
NM_001165934///		0.039		-1.02	-1.13	-1.16	-1.12	***Rgs9***	regulator of G-protein signaling 9	0007186 // G-protein coupled receptor signaling pathway // traceable author statement/
NM_0080	0.0074			1.11	-1.53	-1.16	1.39	***Fmo3***	flavin containing monooxygenase 3	0017144 // drug metabolic process // not recorded///0055114 // oxidation-reduction pro
NM_0011	0.011			1.01	-1.22	-1.16	-1.08	***Smyd1***	SET and MYND domain containing 1	0006338 // chromatin remodeling // inferred from physical interaction///0006351 // tran
NM_0134	0.0496			-1.15	1.12	-1.16	-1.23	***Cd3d***	CD3 antigen, delta polypeptide	0006461 // protein complex assembly // non-traceable author statement///0007166 // c
NM_0100	0.0421			1.03	-1.30	-1.15	-1.08	***Cyp2b9***	cytochrome P450, family 2, subfamily b, polypeptide 9	0006805 // xenobiotic metabolic process // not recorded///0019373 // epoxygenase P45
NM_0292	0.0127			1.01	-1.14	-1.15	-1.13	***Spp2***	secreted phosphoprotein 2	0006461 // protein complex assembly // not recorded///0046849 // bone remodeling //
NM_1454	0.0146	0.005	0.025	1.08	-1.06	-1.15	-1.07	***Csdc2***	cold shock domain containing C2, RNA binding	0006355 // regulation of transcription, DNA-templated // inferred from electronic annota
NM_0166	0.0148	0.021		1.18	-1.26	-1.15	1.07	***Reck***	reversion-inducing-cysteine-rich protein with kazal motifs	0001955 // blood vessel maturation // inferred from genetic interaction///0001955 // blo
XR_105122///XR_107916			0.006	1.02	-1.02	-1.15	-1.22	***Gm9885***	predicted gene 9885	
NM_0214	0.0035			1.02	-1.09	-1.15	-1.11	***Mlxipl***	MLX interacting protein-like	0000122 // negative regulation of transcription from RNA polymerase II promoter // infer
NM_021879///XM_		0.011		1.08	-1.02	-1.15	-1.09	***Oca2***	oculocutaneous albinism II	0006810 // transport // inferred from electronic annotation///0007286 // spermatid dev
NM_0167	0.0155	0.01		1.07	-1.18	-1.15	-1.01	***Pgrmc1***	progesterone receptor membrane component 1	0007411 // axon guidance // inferred from electronic annotation
NM_0277	0.0212			-1.00	-1.26	-1.15	1.10	***Nipbl***	Nipped-B homolog (Drosophila)	0000122 // negative regulation of transcription from RNA polymerase II promoter // not
NM_001163486///		0.034		1.00	-1.04	-1.15	-1.19	***Hsd17b13***	hydroxysteroid (17-beta) dehydrogenase 13	0008152 // metabolic process // inferred from electronic annotation///0046889 // positi
XR_871929///XR_		0.013		1.07	-1.03	-1.15	1.04	***Igf1os***	insulin-like growth factor 1, opposite strand	
NM_016695///XM_		0.015	0.022	1.07	1.02	-1.15	-1.13	***Mpp2***	membrane protein, palmitoylated 2 (MAGUK p55 subfamily member 2)	
NM_008212		0.045		1.06	1.04	-1.15	1.14	***Hadh***	hydroxyacyl-Coenzyme A dehydrogenase	0006629 // lipid metabolic process // inferred from electronic annotation///0006631 // f
NM_001010941///		0.047	0.027	1.01	-1.02	-1.15	-1.15	***Gpr12***	G-protein coupled receptor 12	0006874 // cellular calcium ion homeostasis // inferred from direct assay///0007165 // si
NR_02826	0.0457			1.02	-1.33	-1.15	-1.22	***Dleu2***	deleted in lymphocytic leukemia, 2	0010977 // negative regulation of neuron projection development // inferred from geneti
NM_027918///XM_00651			0.039	1.00	1.07	-1.15	-1.19	***1300017J02Rik***	RIKEN cDNA 1300017J02 gene	0043086 // negative regulation of catalytic activity // inferred from electronic annotation
NM_0076	0.0063			1.00	-1.30	-1.15	-1.14	***Serpina6***	serine (or cysteine) peptidase inhibitor, clade A, member 6	0006810 // transport // inferred from electronic annotation///0008211 // glucocorticoid
NM_011455///NM		0.043		1.05	-1.02	-1.15	-1.01	***Serpinb9e///Serpinb9f///Serpinb9g***	predicted gene 11397///serine (or cysteine) peptidase inhibitor, clade B, member 9e//	0002244 // hematopoietic progenitor cell differentiation // inferred from mutant phenot
NM_182805		0.03		1.14	1.02	-1.15	1.02	***Gpt***	glutamic pyruvic transaminase, soluble	0009058 // biosynthetic process // inferred from electronic annotation///0042853 // L-al
NM_0100	0.0297	0.016		1.07	-1.09	-1.15	-1.04	***Cyp2c38***	cytochrome P450, family 2, subfamily c, polypeptide 38	0006805 // xenobiotic metabolic process // not recorded///0019373 // epoxygenase P45
NM_001128605///		0.04		-1.04	-1.00	-1.15	-1.03	***Psen2***	presenilin 2	0000122 // negative regulation of transcription from RNA polymerase II promoter // infer
NM_001037098///NM_02			0.003	-1.05	-1.20	-1.15	1.11	***Nacc2***	nucleus accumbens associated 2, BEN and BTB (POZ) domain containing	0008285 // negative regulation of cell proliferation // not recorded///0010608 // posttra
NM_027790///XM_		0.014		1.05	-1.01	-1.15	-1.12	***Dhrs2***	dehydrogenase/reductase member 2	0008152 // metabolic process // inferred from electronic annotation///0009636 // respo
NM_0307	0.0419			-1.12	1.02	-1.15	-1.19	***Cxcr6***	chemokine (C-X-C motif) receptor 6	0006935 // chemotaxis // inferred from electronic annotation///0006954 // inflammator
NM_0112	0.0458	0.024		1.03	-1.09	-1.15	-1.12	***Pvt1***	plasmacytoma variant translocation 1	
NM_001110337///		0.011		1.06	-1.10	-1.15	1.01	***Gprc5c***	G protein-coupled receptor, family C, group 5, member C	0007165 // signal transduction // inferred from electronic annotation///0007186 // G-pro
NM_0012	0.014	0.012	0.035	1.01	-1.08	-1.15	-1.17	***Kcnma1***	potassium large conductance calcium-activated channel, subfamily M, alpha member	0001666 // response to hypoxia // inferred from direct assay///0001666 // response to h
NM_0012	0.0263	0.043		1.10	-1.08	-1.15	1.02	***Ttc28***	tetratricopeptide repeat domain 28	0007049 // cell cycle // inferred from electronic annotation///0007067 // mitotic nuclear
NM_013697			0.033	-1.32	-1.05	-1.15	-1.08	***Ttr***	transthyretin	0006810 // transport // inferred from electronic annotation///0042572 // retinol metabo
NM_009766///XM_		0.038		1.06	-1.01	-1.15	-1.08	***Brs3***	bombesin-like receptor 3	0007165 // signal transduction // inferred from electronic annotation///0007186 // G-pro
		0.018	0.019	-1.00	-1.04	-1.15	-1.28	***Gm8479***	thiopurine S-methyltransferase pseudogene	
NM_019510///XM_		0.016		1.09	1.09	-1.15	1.01	***Trpc3***	transient receptor potential cation channel, subfamily C, member 3	0006810 // transport // inferred from electronic annotation///0006811 // ion transport /
NM_001083929///NM_00			0.02	-1.08	-1.12	-1.15	1.15	***Gpx3***	glutathione peroxidase 3	0006749 // glutathione metabolic process // not recorded///0006979 // response to oxid
NM_001286396///		0.01		1.05	1.12	-1.15	-1.08	***Dao***	D-amino acid oxidase	0006551 // leucine metabolic process // inferred from mutant phenotype///0006562 // p
NM_1784	0.0157			-1.05	1.35	-1.15	-1.19	***Myo1g***	myosin IG	0002250 // adaptive immune response // inferred from electronic annotation///0002376
NM_1707	0.0381			-1.02	1.28	-1.14	1.01	***Cd300a***	CD300A antigen	0002376 // immune system process // inferred from electronic annotation///0002552 //
NM_0212	0.0283			1.02	-1.15	-1.14	-1.18	***Wnt1***	wingless-type MMTV integration site family, member 1	0000578 // embryonic axis specification // inferred from mutant phenotype///0001658 /
NM_080729///XM_00651			0.036	-1.15	-1.01	-1.14	-1.02	***Il25***	interleukin 25	0002437 // inflammatory response to antigenic stimulus // not recorded///0006954 // in
NM_0271	0.0093	0.041		1.07	-1.12	-1.14	1.15	***Arhgef12***	Rho guanine nucleotide exchange factor (GEF) 12	0007186 // G-protein coupled receptor signaling pathway // not recorded///0035023 // r
NM_001142732///		0.005		1.11	-1.03	-1.14	-1.07	***Ttll3***	tubulin tyrosine ligase-like family, member 3	0006464 // cellular protein modification process // inferred from electronic annotation//
NM_0085	0.0393			1.15	1.88	-1.14	-1.46	***Ltb***	lymphotoxin B	0006955 // immune response // inferred from electronic annotation///0010467 // gene e
NM_028621		0.008		-1.00	-1.06	-1.14	-1.02	***Krtap21-1***	keratin associated protein 21-1	0001942 // hair follicle development // inferred from mutant phenotype///0007165 // sig
NM_053227///XM_		0.021	0.031	-1.01	-1.11	-1.14	-1.14	***Vmn1r44***	vomeronasal 1 receptor 44	0007165 // signal transduction // inferred from electronic annotation///0007186 // G-pro
NM_0108	0.0276		0.044	-1.17	1.00	-1.14	-1.09	***Myoc***	myocilin	0001649 // osteoblast differentiation // inferred from direct assay///0001649 // osteobla
NM_0276	0.0176			1.06	-1.13	-1.14	-1.08	***Speer3***	spermatogenesis associated glutamate (E)-rich protein 3	
NM_020277///XM_		0.037		1.03	-1.03	-1.14	-1.20	***Trpm5***	transient receptor potential cation channel, subfamily M, member 5	0006810 // transport // inferred from electronic annotation///0006811 // ion transport /
NM_053221		0.032	0.041	1.15	-1.03	-1.14	-1.10	***Vmn1r42***	vomeronasal 1 receptor 42	0007165 // signal transduction // inferred from electronic annotation///0007186 // G-pro
NM_001111015///		0.008	0.007	1.07	-1.04	-1.14	-1.13	***Syn2***	synapsin II	0007269 // neurotransmitter secretion // inferred from genetic interaction///0007269 //
NM_0013	0.0289			-1.17	1.05	-1.14	-1.16	***Ggt1***	gamma-glutamyltransferase 1	0002682 // regulation of immune system process // inferred from mutant phenotype///0
NM_023907		0.017		1.06	-1.07	-1.14	-1.10	***Foxi1***	forkhead box I1	0006351 // transcription, DNA-templated // inferred from electronic annotation///00063
NM_0096	0.0345			-1.12	1.08	-1.14	-1.13	***Apob***	apolipoprotein B	0001701 // in utero embryonic development // inferred from mutant phenotype///00066
NM_001007570		0.012		1.03	1.03	-1.14	-1.07	***Slc25a42***	solute carrier family 25, member 42	0006810 // transport // inferred from electronic annotation///0008152 // metabolic proc

RefSeq Tr	*p* 24h	*p* 48h	*p* 72h	FD Air	FD 24h	FD 48h	FD 72h	Gene Symbol	Gene Title	Gene Ontology Biological Process
NM_027220		0.022		1.04	-1.05	-1.14	-1.12	***Prss32***	protease, serine 32	0006508 // proteolysis // inferred by curator
NM_008814		0.034		1.00	-1.08	-1.14	-1.15	***Pdx1***	pancreatic and duodenal homeobox 1	0000122 // negative regulation of transcription from RNA polymerase II promoter // infer
NM_175309		0.016		1.20	1.05	-1.14	1.18	***Upk3b***	uroplakin 3B	0010629 // negative regulation of gene expression // not recorded///0046325 // negative
NM_001313956///		0.038		1.01	-1.05	-1.14	-1.04	***Krt14///Krt17***	keratin 14///keratin 17	0002009 // morphogenesis of an epithelium // inferred from genetic interaction///00071
NM_001037930///		0.013		1.01	-1.04	-1.14	-1.10	***Gm6787***	proteasome (prosome, macropain) subunit, beta type 7 pseudogene	
NM_0012	0.0222	0.005		1.13	-1.20	-1.14	1.03	***Polr3gl***	polymerase (RNA) III (DNA directed) polypeptide G like	0006359 // regulation of transcription from RNA polymerase III promoter // inferred from
NM_008936			0.03	1.04	-1.17	-1.14	-1.15	***Prop1***	paired like homeodomain factor 1	0000122 // negative regulation of transcription from RNA polymerase II promoter // infer
NM_0136	0.0183			-1.05	1.11	-1.14	-1.12	***Irf4***	interferon regulatory factor 4	0006351 // transcription, DNA-templated // inferred from electronic annotation///00063
NM_007771///XM_01124			0.02	-1.45	-1.22	-1.14	1.00	***Cry1***	cryptochrome 1 (photolyase-like)	0000122 // negative regulation of transcription from RNA polymerase II promoter // infer
NM_0197	0.0163	0.009		1.10	-1.18	-1.14	-1.16	***Tmeff2***	transmembrane protein with EGF-like and two follistatin-like domains 2	
NM_0010	######			-1.02	-1.24	-1.14	-1.10	***Limch1***	LIM and calponin homology domains 1	0031032 // actomyosin structure organization // inferred from electronic annotation
NM_0214	0.0107	0.044		1.10	-1.07	-1.14	1.01	***Mknk2***	MAP kinase-interacting serine/threonine kinase 2	0006417 // regulation of translation // inferred from electronic annotation///0006468 //
NM_009937///NM		0.027	0.022	1.07	-1.04	-1.14	-1.15	***Colq///Hacl1///LOC102639719***	collagen-like tail subunit (single strand of homotrimer) of asymmetric acetylcholineste	0001561 // fatty acid alpha-oxidation // not recorded///0006629 // lipid metabolic proce
NM_026335		0.005		1.04	-1.03	-1.14	-1.12	***Lce1h***	late cornified envelope 1H	0008544 // epidermis development // inferred from electronic annotation///0018149 //
NM_0540	0.0162			1.14	-1.16	-1.14	-1.04	***Psg28***	pregnancy-specific glycoprotein 28	0007565 // female pregnancy // inferred from electronic annotation
NM_015730///XM		0.014		1.00	1.00	-1.14	-1.14	***Chrna4***	cholinergic receptor, nicotinic, alpha polypeptide 4	0001508 // action potential // inferred from mutant phenotype///0001666 // response t
NM_176987///XM		0.029		1.07	1.05	-1.14	-1.07	***Simc1***	SUMO-interacting motifs containing 1	
NM_009510		0.017		1.06	-1.01	-1.14	1.03	***Ezr***	ezrin	0000122 // negative regulation of transcription from RNA polymerase II promoter // not
NM_0107	0.0479			-1.07	1.01	-1.14	-1.10	***Mcpt4***	mast cell protease 4	0002002 // regulation of angiotensin levels in blood // inferred from genetic interaction//
NM_001291280///NM_02			0.012	1.01	-1.01	-1.13	-1.15	***Nmb***	neuromedin B	0007204 // positive regulation of cytosolic calcium ion concentration // not recorded///0
NM_0213	0.0147	0.042		1.09	-1.13	-1.13	-1.02	***Pappa***	pregnancy-associated plasma protein A	0006508 // proteolysis // inferred from electronic annotation///0030154 // cell differenti
NM_0011	0.0267	0.018		1.05	-1.11	-1.13	1.12	***Fmo5***	flavin containing monooxygenase 5	0017144 // drug metabolic process // not recorded///0055114 // oxidation-reduction pr
NM_001081025///		0.031		1.09	-1.11	-1.13	-1.01	***Maats1***	MYCBP-associated, testis expressed 1	
NM_0010	0.0354			1.01	1.24	-1.13	1.14	***Hmga1///Hmga1-rs1***	high mobility group AT-hook 1///high mobility group AT-hook I, related sequence 1	0006284 // base-excision repair // not recorded///0006351 // transcription, DNA-templa
NM_011718///XM		0.007	0.028	1.11	1.04	-1.13	-1.13	***Wnt10b***	wingless-type MMTV integration site family, member 10B	0000086 // G2/M transition of mitotic cell cycle // inferred from genetic interaction///00
NM_0095	0.0048	0.016		1.07	-1.21	-1.13	1.06	***Abcg1***	ATP-binding cassette, sub-family G (WHITE), member 1	0006355 // regulation of transcription, DNA-templated // inferred from mutant phenoty
NM_0088	0.0468			-1.02	-1.20	-1.13	1.08	***Pkd2***	polycystic kidney disease 2	0001658 // branching involved in ureteric bud morphogenesis // inferred from electronic
NM_1530	0.0118	0.029		1.05	-1.11	-1.13	-1.05	***Slc18a1***	solute carrier family 18 (vesicular monoamine), member 1	0006810 // transport // inferred from electronic annotation///0006836 // neurotransmit
NM_001289468///		0.01		1.04	1.02	-1.13	-1.05	***Tcl1***	T cell lymphoma breakpoint 1	0010629 // negative regulation of gene expression // inferred from mutant phenotype///
NM_0089	0.0124			1.04	-1.16	-1.13	-1.15	***Prop1***	paired like homeodomain factor 1	0000122 // negative regulation of transcription from RNA polymerase II promoter // infer
NM_0094	0.0302	0.042	0.022	1.11	-1.09	-1.13	-1.14	***Trh***	thyrotropin releasing hormone	0001666 // response to hypoxia // inferred from electronic annotation///0001692 // hist
NM_008764		0.019		1.05	1.03	-1.13	-1.05	***Tnfrsf11b***	tumor necrosis factor receptor superfamily, member 11b (osteoprotegerin)	0006915 // apoptotic process // inferred from electronic annotation///0006954 // inflam
NM_008262			0.022	-1.03	1.10	-1.13	-1.24	***Onecut1***	one cut domain, family member 1	0001889 // liver development // inferred from genetic interaction///0001952 // regulatio
NM_0011	0.001	0.028		1.05	-1.09	-1.13	-1.05	***Ralbp1***	ralA binding protein 1	0006810 // transport // not recorded///0006897 // endocytosis // not recorded///00070
NM_001048139///		0.005	0.033	1.18	1.06	-1.13	-1.05	***Bdnf***	brain derived neurotrophic factor	0001657 // ureteric bud development // inferred from direct assay///0001662 // behavio
NM_009710///XM		0.045		-1.01	-1.00	-1.13	-1.11	***Art1***	ADP-ribosyltransferase 1	0006471 // protein ADP-ribosylation // inferred from direct assay
NM_0135	0.0117			1.03	1.31	-1.13	-1.16	***Itgb7***	integrin beta 7	0003366 // cell-matrix adhesion involved in ameboidal cell migration // not recorded///0
NM_0010	0.0147			-1.20	1.04	-1.13	-1.13	***Dnah1***	dynein, axonemal, heavy chain 1	0003341 // cilium movement // inferred from electronic annotation///0003351 // epithe
NM_013803///XM		0.043		1.06	-1.08	-1.13	-1.06	***Casr***	calcium-sensing receptor	0001503 // ossification // not recorded///0006816 // calcium ion transport // not record
NM_030259		0.047		-1.01	-1.02	-1.13	-1.09	***Rilpl2***	Rab interacting lysosomal protein-like 2	0003382 // epithelial cell morphogenesis // inferred from mutant phenotype///0006810
NM_0011	0.0026	0.003	0.032	1.04	-1.04	-1.13	1.01	***Hopx***	HOP homeobox	0000122 // negative regulation of transcription from RNA polymerase II promoter // infer
NM_026447///NM		0.024		1.04	1.09	-1.13	-1.08	***Ppm1m***	protein phosphatase 1M	0006470 // protein dephosphorylation // inferred from direct assay
NM_001110780///		0.029		1.01	-1.11	-1.13	-1.09	***Syn1***	synapsin I	0007269 // neurotransmitter secretion // inferred from genetic interaction///0007269 //
NM_0118	0.026			1.02	-1.15	-1.13	-1.04	***Aadat***	aminoadipate aminotransferase	0006103 // 2-oxoglutarate metabolic process // not recorded///0006536 // glutamate m
NM_0010	0.0467			1.09	-1.00	-1.13	1.13	***Atxn7l3***	ataxin 7-like 3	0006351 // transcription, DNA-templated // inferred from electronic annotation///00063
NM_028176///XM_00653			0.023	-1.21	-1.04	-1.13	1.03	***Cda***	cytidine deaminase	0009972 // cytidine deamination // not recorded///0030308 // negative regulation of cel
NM_0107	0.0188			-1.02	1.27	-1.13	-1.09	***Lst1***	leukocyte specific transcript 1	0000902 // cell morphogenesis // inferred from electronic annotation///0002376 // imm
NM_0013	0.0398			1.01	-1.20	-1.13	-1.14	***Slc16a4***	solute carrier family 16 (monocarboxylic acid transporters), member 4	0006810 // transport // inferred from electronic annotation///0015718 // monocarboxyl
NM_0136	0.0447			1.14	-1.35	-1.13	-1.02	***Mt1***	metallothionein 1	0000032 // cell wall mannoprotein biosynthetic process // ---///0006486 // protein glyco
NM_130860///XM		0.037		1.09	1.10	-1.13	-1.08	***Cdk9***	cyclin-dependent kinase 9 (CDC2-related kinase)	0006281 // DNA repair // inferred from electronic annotation///0006282 // regulation of
NM_001291149///		0.044	0.034	1.07	-1.06	-1.13	-1.14	***Ptprt***	protein tyrosine phosphatase, receptor type, T	0006470 // protein dephosphorylation // inferred from direct assay///0006470 // protei
NM_1454	0.0191			-1.09	1.06	-1.13	-1.14	***Cd300ld***	CD300 molecule-like family member d	0002376 // immune system process // inferred from electronic annotation///0032675 //
NM_010645			0.003	1.05	-1.13	-1.13	-1.21	***Klk1b1***	kallikrein 1-related peptidase b1	0002255 // tissue kallikrein-kinin cascade // inferred from mutant phenotype///0002936
NM_0010	0.0278			-1.04	1.07	-1.13	-1.06	***Fmnl1***	formin-like 1	0006929 // substrate-dependent cell migration // inferred from mutant phenotype///00
NM_008530///XM		0.028		1.01	-1.20	-1.13	1.03	***Ly6f***	lymphocyte antigen 6 complex, locus F	
NM_0012	0.0225			-1.07	1.03	-1.13	-1.19	***Add2***	adducin 2 (beta)	0006461 // protein complex assembly // not recorded///0006811 // ion transport // not
NM_0224	0.011			-1.08	1.02	-1.13	-1.03	***Tmub1***	transmembrane and ubiquitin-like domain containing 1	0030433 // ER-associated ubiquitin-dependent protein catabolic process // not recorded
NM_0287	0.0427			1.03	-1.14	-1.13	1.09	***Cyp2s1***	cytochrome P450, family 2, subfamily s, polypeptide 1	0006805 // xenobiotic metabolic process // not recorded///0019373 // epoxygenase P45
NM_013906			#####	-1.11	-1.15	-1.13	1.12	***Adamts8***	a disintegrin-like and metallopeptidase (reprolysin type) with thrombospondin type 1	0006508 // proteolysis // inferred from electronic annotation
NM_0157	0.0352			1.03	-1.23	-1.13	-1.05	***Spag6***	sperm associated antigen 6	0007288 // sperm axoneme assembly // inferred from mutant phenotype///0021591 //
NM_013533///XM_00650			0.023	1.01	1.04	-1.13	-1.17	***Gpr162***	G protein-coupled receptor 162	0007165 // signal transduction // inferred from electronic annotation///0007186 // G-pr
NM_0168	0.0401			-1.12	1.04	-1.13	-1.15	***Sart1***	squamous cell carcinoma antigen recognized by T cells 1	0000387 // spliceosomal snRNP assembly // not recorded///0000398 // mRNA splicing, v
NM_001136056///NM_00			0.027	1.03	-1.06	-1.13	-1.16	***Cntfr***	ciliary neurotrophic factor receptor	0001967 // suckling behavior // inferred from mutant phenotype///0003360 // brainsten
NM_133359			0.043	1.01	-1.04	-1.13	-1.20	***Krtap19-9b***	keratin associated protein 19-9B	

RefSeq Tra	*p* 24h	*p* 48h	*p* 72h	FD Air	FD 24h	FD 48h	FD 72h	Gene Symbol	Gene Title	Gene Ontology Biological Process
NM_0118	0.0365			-1.06	1.10	-1.13	-1.13	***Hcst***	hematopoietic cell signal transducer	0006468 // protein phosphorylation // not recorded///0014068 // positive regulation of
NM_007818		0.039		-1.44	-1.14	-1.13	-1.16	***Cyp3a11***	cytochrome P450, family 3, subfamily a, polypeptide 11	0055114 // oxidation-reduction process // inferred from electronic annotation
NM_0242	0.0382			-1.24	-1.33	-1.12	-1.34	***Mxra8***	matrix-remodelling associated 8	0060857 // establishment of glial blood-brain barrier // inferred from expression pattern
NM_001037859///		0.04		1.01	1.03	-1.12	-1.02	***Csf1r***	colony stimulating factor 1 receptor	0001934 // positive regulation of protein phosphorylation // not recorded///0002376 // i
NM_001081445///		0.029		1.01	-1.03	-1.12	-1.07	***Ncam1***	neural cell adhesion molecule 1	0001928 // regulation of exocyst assembly // inferred from mutant phenotype///000715
NM_0012	0.0382			-1.11	-1.32	-1.12	-1.03	***Trim3***	tripartite motif-containing 3	0006810 // transport // inferred from electronic annotation///0015031 // protein transp
NM_0010	0.0196	0.036		1.12	-1.07	-1.12	-1.02	***Zfr2***	zinc finger RNA binding protein 2	0006955 // immune response // inferred from electronic annotation///0008152 // metab
NM_009043		0.012	0.019	1.07	-1.10	-1.12	-1.12	***Reg2***	regenerating islet-derived 2	0001967 // suckling behavior // inferred from mutant phenotype///0042552 // myelinati
NM_153119///XM_		0.003		1.04	1.12	-1.12	1.08	***Plekho2***	pleckstrin homology domain containing, family O member 2	
NM_028149///NM		0.032		1.03	1.03	-1.12	1.04	***Fbxl20***	F-box and leucine-rich repeat protein 20	0001662 // behavioral fear response // inferred from mutant phenotype///0016567 // pr
NM_1725	0.0116	0.011	0.011	1.08	-1.06	-1.12	-1.14	***Klhl11***	kelch-like 11	
NM_0080	0.0455	0.003		1.11	-1.00	-1.12	-1.06	***Gas1***	growth arrest specific 1	0002053 // positive regulation of mesenchymal cell proliferation // inferred from mutant
NM_011274///XM_		0.032		1.01	1.00	-1.12	1.01	***Uri1***	URI1, prefoldin-like chaperone	0000122 // negative regulation of transcription from RNA polymerase II promoter // not
NM_001252501///NM_00			0.046	-1.01	1.01	-1.12	-1.17	***Sorcs1***	sortilin-related VPS10 domain containing receptor 1	
NM_0075	0.0281			-1.12	1.17	-1.12	-1.13	***Cxcr5***	chemokine (C-X-C motif) receptor 5	0006935 // chemotaxis // inferred from electronic annotation///0006955 // immune resp
NM_0166	0.018			1.00	1.14	-1.12	1.01	***Il27ra***	interleukin 27 receptor, alpha	0002827 // positive regulation of T-helper 1 type immune response // inferred from muta
NM_012034///XM_006524			0.035	-1.05	-1.16	-1.12	-1.29	***Tpsg1***	tryptase gamma 1	0006508 // proteolysis // inferred from electronic annotation
NM_001316370///NM_00			0.038	-1.66	-1.71	-1.12	1.05	***P4ha1***	procollagen-proline, 2-oxoglutarate 4-dioxygenase (proline 4-hydroxylase), alpha 1 pol	0018401 // peptidyl-proline hydroxylation to 4-hydroxy-L-proline // inferred from direct a
NM_008878			0.048	-1.02	-1.01	-1.12	-1.19	***Serpinf2***	serine (or cysteine) peptidase inhibitor, clade F, member 2	0002034 // regulation of blood vessel size by renin-angiotensin // inferred from mutant p
NM_080448///NM		0.047		1.03	-1.10	-1.12	1.01	***Srgap3***	SLIT-ROBO Rho GTPase activating protein 3	0007165 // signal transduction // inferred from electronic annotation///0030336 // nega
NM_1447	0.0171	0.043		1.14	-1.09	-1.12	-1.13	***Wt1***	Wilms tumor 1 homolog	0000122 // negative regulation of transcription from RNA polymerase II promoter // not
NM_001146215///		0.015		1.09	1.03	-1.12	-1.10	***Msh5***	mutS homolog 5 (E. coli)	0000710 // meiotic mismatch repair // ---///0006298 // mismatch repair // inferred from
NM_001244198///		0.009		1.12	1.05	-1.12	-1.09	***Pax6***	paired box 6	0000122 // negative regulation of transcription from RNA polymerase II promoter // infer
NM_0202	0.0168			1.04	-1.14	-1.12	-1.10	***Cldn9***	claudin 9	0016338 // calcium-independent cell-cell adhesion via plasma membrane cell-adhesion m
NM_1537	0.027	0.004	0.003	1.06	-1.06	-1.12	-1.23	***Rell2***	RELT-like 2	0010811 // positive regulation of cell-substrate adhesion // inferred from direct assay
NM_1340	0.0011	0.043		1.12	-1.11	-1.12	-1.06	***Tbc1d10a***	TBC1 domain family, member 10a	0042147 // retrograde transport, endosome to Golgi // not recorded///0043547 // positi
NM_1332	0.0087	0.034		1.11	-1.24	-1.12	1.06	***Zfp704***	zinc finger protein 704	
NM_001290792///		0.036		1.01	1.06	-1.12	1.07	***Wdr45***	WD repeat domain 45	0000045 // autophagosome assembly // not recorded///0000422 // mitophagy // not rec
NM_001276676///NM_00			0.007	1.09	1.07	-1.12	-1.26	***Syt6***	synaptotagmin VI	0006906 // vesicle fusion // ---///0007340 // acrosome reaction // inferred from direct as
NM_0136	0.0232	0.018		1.00	-1.15	-1.12	-1.00	***Mt3***	metallothionein 3	0000060 // protein import into nucleus, translocation // inferred from sequence or struct
NM_001081363///XM_006			0.017	-1.11	1.00	-1.12	1.12	***Cenpf***	centromere protein F	0000278 // mitotic cell cycle // not recorded///0002724 // regulation of T cell cytokine pr
NM_030678			0.023	-1.11	-1.01	-1.12	1.01	***Gys1***	glycogen synthase 1, muscle	0005977 // glycogen metabolic process // not recorded///0005978 // glycogen biosynthe
NM_0011	0.0353			1.02	-1.24	-1.12	-1.09	***Pclo***	piccolo (presynaptic cytomatrix protein)	0007010 // cytoskeleton organization // inferred from direct assay///0007416 // synapse
NM_001139520///		0.021		1.09	1.07	-1.12	1.04	***Samhd1***	SAM domain and HD domain, 1	0002376 // immune system process // inferred from electronic annotation///0006203 //
NM_0011	0.0196			-1.00	-1.18	-1.12	1.02	***Mak***	male germ cell-associated kinase	0006351 // transcription, DNA-templated // inferred from electronic annotation///00063
NM_011850			0.016	1.03	1.05	-1.12	-1.10	***Nr0b2***	nuclear receptor subfamily 0, group B, member 2	0000122 // negative regulation of transcription from RNA polymerase II promoter // not
NM_0010	0.0262	0.017		1.02	-1.28	-1.12	1.10	***Chka***	choline kinase alpha	0006580 // ethanolamine metabolic process // not recorded///0006629 // lipid metaboli
NM_013772///XM_		0.045	0.027	1.02	-1.05	-1.12	-1.16	***LOC100041483///Tcl1b3***	protein TCL1B3///T cell leukemia/lymphoma 1B, 3	
NM_008175		0.006		1.06	1.03	-1.12	1.06	***Grn***	granulin	0001835 // blastocyst hatching // inferred from direct assay///0007566 // embryo implar
NM_0011	0.0139			-1.09	1.05	-1.12	-1.16	***Tcf7l2***	transcription factor 7 like 2, T cell specific, HMG box	0000122 // negative regulation of transcription from RNA polymerase II promoter // infer
NM_0202	0.0258			-1.18	-1.03	-1.12	-1.15	***Magea4***	melanoma antigen, family A, 4	
NM_0010	0.0348			1.02	-1.08	-1.12	-1.12	***Oxtr***	oxytocin receptor	0001967 // suckling behavior // not recorded///0001975 // response to amphetamine //
NM_1772	0.0207			-1.03	1.10	-1.12	-1.13	***Trac///Tnpo3///Traj15///Trav6-7-dv9///Tr***	T cell receptor alpha constant///transportin 3///T cell receptor alpha joining 15///T ce	0050852 // T cell receptor signaling pathway // ---///0006606 // protein import into nucle
NM_0281	0.0047			1.04	-1.15	-1.12	-1.01	***Nkd2***	naked cuticle 2 homolog (Drosophila)	0006810 // transport // inferred from electronic annotation///0006887 // exocytosis // ir
NM_011663		0.013		1.06	-1.19	-1.12	1.02	***Zrsr1***	zinc finger (CCCH type), RNA binding motif and serine/arginine rich 1	
NM_029096///NM		0.027	0.01	1.04	1.04	-1.12	-1.10	***Rita1***	RBPJ interacting and tubulin associated 1	0007219 // Notch signaling pathway // inferred from electronic annotation///0007399 //
NM_009718		0.018		1.07	-1.05	-1.12	-1.10	***Neurog2***	neurogenin 2	0001764 // neuron migration // inferred from mutant phenotype///0006351 // transcript
NM_0012	0.0412			1.05	-1.12	-1.12	1.09	***Scnn1b***	sodium channel, nonvoltage-gated 1 beta	0001666 // response to hypoxia // inferred from electronic annotation///0002028 // regu
NM_0081	0.0045		#####	1.04	-1.17	-1.12	-1.24	***Gpr33***	G protein-coupled receptor 33	0007165 // signal transduction // inferred from electronic annotation///0007186 // G-pro
NM_001315503///		0.032		1.05	-1.08	-1.12	-1.11	***Adcyap1***	adenylate cyclase activating polypeptide 1	0001541 // ovarian follicle development // not recorded///0001662 // behavioral fear re
NM_001136064///		0.05		1.05	1.04	-1.12	1.05	***Bscl2***	Berardinelli-Seip congenital lipodystrophy 2 homolog (seipin)	0006629 // lipid metabolic process // inferred from electronic annotation///0016042 // li
NM_016982///NM		0.026		1.04	-1.04	-1.12	-1.07	***Vpreb1///Vpreb2***	pre-B lymphocyte gene 1///pre-B lymphocyte gene 2	0000902 // cell morphogenesis // inferred from genetic interaction///0002377 // immun
NM_0087	0.0471			1.05	-1.20	-1.12	-1.15	***Ints6***	integrator complex subunit 6	0016180 // snRNA processing // not recorded
NM_0082	0.0125			1.08	-1.37	-1.12	1.18	***Hpgd***	hydroxyprostaglandin dehydrogenase 15 (NAD)	0006629 // lipid metabolic process // inferred from electronic annotation///0006631 // f
NM_144524///XM_		0.033		1.07	1.04	-1.12	-1.02	***Angel1***	angel homolog 1 (Drosophila)	
NM_0089	0.006			-1.03	-1.19	-1.12	1.00	***Npepps***	aminopeptidase puromycin sensitive	0006508 // proteolysis // inferred from electronic annotation///0043171 // peptide catal
NM_026261		0.012	0.016	1.11	-1.03	-1.12	-1.09	***Ubl4b***	ubiquitin-like 4B	1903955 // positive regulation of protein targeting to mitochondrion // not recorded
NM_0012	0.0252			1.01	-1.13	-1.12	-1.18	***Cdh23***	cadherin 23 (otocadherin)	0006816 // calcium ion transport // not recorded///0007155 // cell adhesion // traceable
NM_001198835///NM_00			0.035	1.02	-1.03	-1.12	-1.15	***Coch***	coagulation factor C homolog (Limulus polyphemus)	0007605 // sensory perception of sound // inferred from mutant phenotype///0008360 /
NM_0100	0.0236			1.03	-1.18	-1.12	1.06	***Dspp***	dentin sialophosphoprotein	0031214 // biomineral tissue development // inferred from electronic annotation///0071
NM_0093	0.0413	0.02	0.028	1.09	-1.09	-1.12	-1.19	***Tecta***	tectorin alpha	0007160 // cell-matrix adhesion // inferred from electronic annotation///0007605 // sen
NM_0302	0.0478			-1.01	1.09	-1.11	-1.10	***Aacs***	acetoacetyl-CoA synthetase	0001889 // liver development // not recorded///0006629 // lipid metabolic process // inf
NM_008108///XM_00650			0.022	-1.03	1.02	-1.11	-1.19	***Gdf3***	growth differentiation factor 3	0001501 // skeletal system development // not recorded///0001654 // eye development
NM_0012	0.03	0.037	0.006	1.15	1.00	-1.11	-1.16	***Nxf2***	nuclear RNA export factor 2	0006405 // RNA export from nucleus // inferred from direct assay///0006405 // RNA exp

RefSeq Tr	*p* 24h	*p* 48h	*p* 72h	FD Air	FD 24h	FD 48h	FD 72h	Gene Symbol	Gene Title	Gene Ontology Biological Process
NM_178020			0.044	-1.07	1.00	-1.11	-1.26	***Hyal3***	hyaluronoglucosaminidase 3	0005975 // carbohydrate metabolic process // inferred from electronic annotation///000
NM_023160///XM		0.043		1.01	-1.05	-1.11	-1.13	***Cml1***	camello-like 1	0001702 // gastrulation with mouth forming second // non-traceable author statement/
NM_133346			0.045	-1.01	-1.09	-1.11	-1.11	***Asb6***	ankyrin repeat and SOCS box-containing 6	0016567 // protein ubiquitination // inferred from electronic annotation///0035556 // in
NM_0169	0.0486			1.02	-1.09	-1.11	-1.14	***Prg3***	proteoglycan 3	0001694 // histamine biosynthetic process // not recorded///0006955 // immune respon
NM_0012	0.0134			1.11	-1.02	-1.11	-1.02	***Rhbdf1***	rhomboid family 1 (Drosophila)	0006508 // proteolysis // inferred from electronic annotation///0008283 // cell prolifera
NM_019450			0.05	1.03	-1.04	-1.11	-1.13	***Il1f6***	interleukin 1 family, member 6	0001819 // positive regulation of cytokine production // inferred from direct assay///000
NM_0088	0.0062			-1.11	1.29	-1.11	1.01	***Pim1***	proviral integration site 1	0006468 // protein phosphorylation // not recorded///0007346 // regulation of mitotic c
NM_031184///XM		0.036	0.024	1.08	1.08	-1.11	-1.05	***Glis2***	GLIS family zinc finger 2	0000122 // negative regulation of transcription from RNA polymerase II promoter // infe
NM_001033402///		0.022	0.008	1.12	1.00	-1.11	-1.12	***Zmiz1***	zinc finger, MIZ-type containing 1	0001570 // vasculogenesis // inferred from mutant phenotype///0001701 // in utero em
NM_030687///XM		0.029	0.04	1.08	1.00	-1.11	-1.04	***Slco1a4***	solute carrier organic anion transporter family, member 1a4	0006810 // transport // inferred from electronic annotation///0006811 // ion transport /
NM_001113529///		0.011		1.10	1.06	-1.11	1.05	***Csf1***	colony stimulating factor 1 (macrophage)	0001503 // ossification // not recorded///0001954 // positive regulation of cell-matrix ad
NM_0119	0.0397			-1.15	-1.04	-1.11	-1.14	***Vax2***	ventral anterior homeobox 2	0000122 // negative regulation of transcription from RNA polymerase II promoter // infe
NM_025886///XM		0.039		1.14	1.06	-1.11	1.00	***Rassf7***	Ras association (RalGDS/AF-6) domain family (N-terminal) member 7	0006915 // apoptotic process // inferred from electronic annotation///0007165 // signal
NM_009301			0.019	-1.07	1.01	-1.11	-1.05	***Svs5***	seminal vesicle secretory protein 5	
NM_008712///XM_00653			0.022	-1.02	-1.03	-1.11	-1.20	***Nos1***	nitric oxide synthase 1, neuronal	0001666 // response to hypoxia // inferred from electronic annotation///0002028 // regu
NM_011195		0.032		1.08	-1.11	-1.11	-1.07	***Ptcra***	pre T cell antigen receptor alpha	0070244 // negative regulation of thymocyte apoptotic process // inferred from mutant
NM_001136259///		0.023		1.04	1.04	-1.11	1.06	***Tom1***	target of myb1 homolog (chicken)	0006810 // transport // inferred from electronic annotation///0006886 // intracellular pr
NM_153112			0.049	-1.10	-1.09	-1.11	-1.23	***Cadm4***	cell adhesion molecule 4	0007155 // cell adhesion // inferred from electronic annotation///0007156 // homophilic
NM_031396///XM		0.004	0.022	1.11	-1.01	-1.11	-1.13	***Cnnm1***	cyclin M1	0006810 // transport // inferred from electronic annotation///0006811 // ion transport /
NM_001302397///		0.048		1.15	1.00	-1.11	1.05	***Phf1***	PHD finger protein 1	0006351 // transcription, DNA-templated // inferred from electronic annotation///00063
NM_013630///XM		0.011		1.11	-1.12	-1.11	1.15	***Pkd1***	polycystic kidney disease 1 homolog	0001502 // cartilage condensation // inferred from mutant phenotype///0001568 // bloo
NM_021715///XR_		0.029		1.08	1.00	-1.11	-1.13	***Chst7***	carbohydrate (N-acetylglucosamino) sulfotransferase 7	0005975 // carbohydrate metabolic process // inferred from electronic annotation///000
XR_389155///XR_393665/			0.048	1.01	1.00	-1.11	-1.18	***Myo15b***	myosin XVB	0008152 // metabolic process // inferred from electronic annotation
NM_008022			0.029	-1.05	-1.07	-1.11	-1.40	***Foxd4***	forkhead box D4	0006351 // transcription, DNA-templated // inferred from electronic annotation///00063
NM_0253	0.0375			-1.09	1.12	-1.11	-1.06	***Chchd5***	coiled-coil-helix-coiled-coil-helix domain containing 5	0009060 // aerobic respiration // not recorded///0033617 // mitochondrial respiratory c
NM_001285807///NM_00			0.04	-1.06	-1.10	-1.11	1.03	***Dtna***	dystrobrevin alpha	
NM_0012	0.049			1.08	-1.09	-1.11	-1.09	***Tet1***	tet methylcytosine dioxygenase 1	0001826 // inner cell mass cell differentiation // inferred from mutant phenotype///0006
NM_008835///NR_028271			0.021	-1.02	-1.12	-1.11	-1.18	***Phxr4***	per-hexamer repeat gene 4	
NM_001316360///		0.025		1.07	1.34	-1.11	1.10	***Bmp4***	bone morphogenetic protein 4	0000122 // negative regulation of transcription from RNA polymerase II promoter // infe
NM_0079	0.0266			1.09	-1.20	-1.11	-1.18	***Endog***	endonuclease G	0001701 // in utero embryonic development // inferred from mutant phenotype///00063
NM_024432///XM		0.034		1.12	1.02	-1.11	-1.08	***Ubxn6***	UBX domain protein 6	
NM_001008542///		0.006		1.10	1.04	-1.11	1.07	***Mxi1***	MAX interactor 1, dimerization protein	0000122 // negative regulation of transcription from RNA polymerase II promoter // infe
NM_001142335///		0.038	0.03	1.33	1.32	-1.11	-1.15	***Lmo2***	LIM domain only 2	0035162 // embryonic hemopoiesis // inferred from genetic interaction///0042789 // m
NM_144518///XM		0.043		1.14	-1.00	-1.11	-1.07	***2900011O08Rik***	RIKEN cDNA 2900011O08 gene	0007026 // negative regulation of microtubule depolymerization // inferred from mutant
NM_001042528///NM_00			0.014	-1.01	-1.06	-1.11	-1.15	***Cacna1b***	calcium channel, voltage-dependent, N type, alpha 1B subunit	0001956 // positive regulation of neurotransmitter secretion // not recorded///0006810
NM_0011	######	0.027		1.15	-1.09	-1.11	-1.00	***Abl1***	c-abl oncogene 1, non-receptor tyrosine kinase	0001922 // B-1 B cell homeostasis // inferred from mutant phenotype///0001934 // posi
NM_0313	0.0019			1.04	-1.13	-1.11	-1.02	***Tex13***	testis expressed gene 13	
NM_001301805///NM_00			0.017	1.06	1.03	-1.11	-1.22	***Hes2***	hairy and enhancer of split 2 (Drosophila)	0006351 // transcription, DNA-templated // inferred from electronic annotation///00063
NM_011040///XM_00649			0.036	-1.17	-1.09	-1.11	-1.07	***Pax8***	paired box 8	0001655 // urogenital system development // inferred from genetic interaction///00016
NM_001081306///NM_00			0.029	-1.16	-1.08	-1.11	-1.05	***Ptprz1***	protein tyrosine phosphatase, receptor type Z, polypeptide 1	0002244 // hematopoietic progenitor cell differentiation // inferred from mutant phenot
NM_007817		0.027		-1.01	-1.14	-1.11	1.18	***Cyp2f2***	cytochrome P450, family 2, subfamily f, polypeptide 2	0006805 // xenobiotic metabolic process // not recorded///0009636 // response to toxic
NM_153558///XM_00649			0.005	-1.12	-1.09	-1.11	1.03	***Obp2a***	odorant binding protein 2A	0006810 // transport // inferred from electronic annotation
NM_015779		0.045		1.00	1.01	-1.11	-1.18	***Elane***	elastase, neutrophil expressed	0000122 // negative regulation of transcription from RNA polymerase II promoter // not
NM_0012	0.0151			1.02	-1.15	-1.11	1.00	***Anks1***	ankyrin repeat and SAM domain containing 1	0006929 // substrate-dependent cell migration // inferred from direct assay///0016322 /
NM_1452	0.0479	0.02		1.25	-1.06	-1.11	1.22	***Appl2***	adaptor protein, phosphotyrosine interaction, PH domain and leucine zipper containin	0007049 // cell cycle // inferred from electronic annotation///0008283 // cell proliferatio
NM_0278	0.0113			1.02	-1.09	-1.11	-1.06	***Fam110c***	family with sequence similarity 110, member C	0030335 // positive regulation of cell migration // not recorded///0051897 // positive reg
NM_0292	0.0201			1.05	1.23	-1.11	1.03	***Rnf19b***	ring finger protein 19B	0000209 // protein polyubiquitination // not recorded///0002250 // adaptive immune re
NM_009482			0.02	1.04	-1.03	-1.11	-1.16	***Utf1***	undifferentiated embryonic cell transcription factor 1	0006351 // transcription, DNA-templated // inferred from electronic annotation///00063
NM_0079	0.0015	0.017	0.005	1.07	-1.11	-1.11	-1.18	***Etv2***	ets variant 2	0001701 // in utero embryonic development // inferred from mutant phenotype///0001
NM_138955///XM_01124			0.04	1.03	1.00	-1.11	-1.10	***Abcg4***	ATP-binding cassette, sub-family G (WHITE), member 4	0006355 // regulation of transcription, DNA-templated // inferred from mutant phenoty
NM_0011	0.0207			1.03	-1.10	-1.11	-1.10	***Rab17***	RAB17, member RAS oncogene family	0002415 // immunoglobulin transcytosis in epithelial cells mediated by polymeric immun
NM_001290574///		0.031		1.09	1.02	-1.11	-1.08	***Ppp1r3f***	protein phosphatase 1, regulatory (inhibitor) subunit 3F	0005979 // regulation of glycogen biosynthetic process // not recorded///2000465 // reg
NM_0115	0.0035			1.04	-1.10	-1.11	-1.12	***Sry***	sex determining region of Chr Y	0000122 // negative regulation of transcription from RNA polymerase II promoter // infe
NM_0104	0.0229			1.05	-1.15	-1.11	-1.11	***Hoxc6***	homeobox C6	0006351 // transcription, DNA-templated // inferred from electronic annotation///00063
NM_001311152///		0.026		1.06	1.09	-1.11	-1.00	***Rnf123***	ring finger protein 123	0016567 // protein ubiquitination // inferred from electronic annotation
NM_029658			0.016	-1.02	-1.09	-1.11	-1.18	***Fam101b***	family with sequence similarity 101, member B	0001837 // epithelial to mesenchymal transition // inferred from genetic interaction///0
NM_008003			0.017	1.03	1.01	-1.11	-1.14	***Fgf15***	fibroblast growth factor 15	0001755 // neural crest cell migration // inferred from mutant phenotype///0001934 //
NR_001463///NR_		0.002	0.013	1.03	-1.04	-1.11	-1.10	***Xist***	inactive X specific transcripts	0009048 // dosage compensation by inactivation of X chromosome // inferred from dire
NM_009253///XM_00651			0.019	-1.14	-1.09	-1.11	1.00	***Serpina3m***	serine (or cysteine) peptidase inhibitor, clade A, member 3M	0010466 // negative regulation of peptidase activity // inferred from electronic annotatio
NM_0194	0.0227			1.05	-1.20	-1.11	1.15	***Epb41l4b***	erythrocyte membrane protein band 4.1 like 4b	0010628 // positive regulation of gene expression // not recorded///0031032 // actomyo
NM_007848			0.016	-1.01	1.02	-1.10	-1.17	***Defa-rs7***	defensin, alpha, related sequence 7	0006952 // defense response // inferred from electronic annotation///0042742 // defens
NM_175244///XR_		0.036		1.08	-1.01	-1.10	1.01	***Hectd3***	HECT domain containing 3	0016567 // protein ubiquitination // inferred from electronic annotation///0016567 // pr
NM_133235///XR_373242			0.02	1.06	-1.03	-1.10	-1.20	***Khdrbs2***	KH domain containing, RNA binding, signal transduction associated 2	0006351 // transcription, DNA-templated // inferred from electronic annotation///00063
NM_0076	0.0097	0.016		1.08	-1.03	-1.10	1.26	***Cbr2***	carbonyl reductase 2	0006116 // NADH oxidation // traceable author statement///0008152 // metabolic proce

RefSeq Tra	*p* 24h	*p* 48h	*p* 72h	FD Air	FD 24h	FD 48h	FD 72h	Gene Symbol	Gene Title	Gene Ontology Biological Process
NM_0217	0.0358	0.039		-1.26	-1.07	-1.10	-1.14	***Trappc4***	trafficking protein particle complex 4	0006810 // transport // inferred from electronic annotation///0006888 // ER to Golgi ves
NM_001308327///		0.008		1.06	1.13	-1.10	-1.15	***Enpp1***	ectonucleotide pyrophosphatase/phosphodiesterase 1	0006091 // generation of precursor metabolites and energy // not recorded///0006796 /
NM_146064///XM		0.045		1.05	1.05	-1.10	1.02	***Soat2***	sterol O-acyltransferase 2	0006629 // lipid metabolic process // inferred from electronic annotation///0008202 // s
NM_0296	0.0475			-1.14	1.03	-1.10	-1.09	***Stambpl1***	STAM binding protein like 1	0006508 // proteolysis // inferred from electronic annotation
NM_198108		0.05	0.031	1.05	1.03	-1.10	-1.11	***Morn4***	MORN repeat containing 4	
NM_025716		0.048		1.05	1.06	-1.10	1.10	***Spryd4***	SPRY domain containing 4	
NM_008759		0.025	0.035	1.03	-1.10	-1.10	-1.16	***Sebox***	SEBOX homeobox	0006351 // transcription, DNA-templated // inferred from electronic annotation///00063
NM_001177594///		0.028		1.09	-1.01	-1.10	1.04	***Slc8b1***	solute carrier family 8 (sodium/lithium/calcium exchanger), member B1	0006810 // transport // inferred from electronic annotation///0006811 // ion transport /
NM_0198	0.0063			1.06	1.40	-1.10	1.36	***Dusp14***	dual specificity phosphatase 14	0000188 // inactivation of MAPK activity // inferred from electronic annotation///000647
NM_133643///XM_00651			0.007	-1.00	-1.01	-1.10	-1.22	***Edaradd***	EDAR (ectodysplasin-A receptor)-associated death domain	0001942 // hair follicle development // inferred from genetic interaction///0007165 // si
NM_0010	0.0171			1.05	-1.49	-1.10	1.33	***Rtkn2***	rhotekin 2	0008284 // positive regulation of cell proliferation // inferred from direct assay///003009
NM_010149///XM		0.003	0.025	1.10	1.05	-1.10	-1.12	***Epor***	erythropoietin receptor	0003007 // heart morphogenesis // traceable author statement///0007165 // signal trans
NM_001145876///		0.006		1.05	-1.01	-1.10	1.06	***Slc25a44***	solute carrier family 25, member 44	0006810 // transport // inferred from electronic annotation///0055085 // transmembrar
NM_054095///XM		0.045	0.002	1.06	-1.02	-1.10	-1.13	***Necab2***	N-terminal EF-hand calcium binding protein 2	0042984 // regulation of amyloid precursor protein biosynthetic process // not recorded
NM_138955///XM		0.013		1.03	-1.05	-1.10	-1.10	***Abcg4***	ATP-binding cassette, sub-family G (WHITE), member 4	0006355 // regulation of transcription, DNA-templated // inferred from mutant phenotyp
NM_0012	0.0383	0.007	0.012	1.05	-1.11	-1.10	-1.12	***Pde10a***	phosphodiesterase 10A	0006198 // cAMP catabolic process // not recorded///0007165 // signal transduction // i
NM_001285487///		0.025		1.06	1.04	-1.10	1.01	***Mknk1***	MAP kinase-interacting serine/threonine kinase 1	0006417 // regulation of translation // inferred from electronic annotation///0006446 //
NM_0010	0.0045			1.07	-1.17	-1.10	1.17	***5330417C22Rik***	RIKEN cDNA 5330417C22 gene	0006914 // autophagy // inferred from electronic annotation///0009267 // cellular respo
NM_0011	0.0081		0.043	1.09	-1.14	-1.10	-1.03	***Rapgef3***	Rap guanine nucleotide exchange factor (GEF) 3	0001525 // angiogenesis // inferred from electronic annotation///0001932 // regulation
NM_080555		0.015		1.03	-1.01	-1.10	-1.06	***Plpp3***	phospholipid phosphatase 3	0001568 // blood vessel development // inferred from mutant phenotype///0001702 // g
NM_0297	0.002			-1.16	1.13	-1.10	1.01	***Rps9***	ribosomal protein S9	0006412 // translation // not recorded///0008284 // positive regulation of cell proliferati
NM_133902///XM_01124			0.01	-1.05	1.08	-1.10	-1.29	***Sdsl***	serine dehydratase-like	0006565 // L-serine catabolic process // not recorded///0006567 // threonine catabolic p
NM_0011	0.0011	0.019		1.03	-1.10	-1.10	-1.10	***Cd160***	CD160 antigen	0007165 // signal transduction // ---///0007165 // signal transduction // inferred from el
NM_008167///XM_00650			0.007	1.00	-1.04	-1.10	-1.17	***Grid2***	glutamate receptor, ionotropic, delta 2	0006810 // transport // inferred from electronic annotation///0006811 // ion transport /
NM_0114	0.0207			1.01	-1.17	-1.10	1.08	***Sox13***	SRY (sex determining region Y)-box 13	0006351 // transcription, DNA-templated // inferred from electronic annotation///00063
NM_0256	0.0055			1.02	-1.22	-1.10	-1.13	***Mis18a***	MIS18 kinetochore protein homolog A (S. pombe)	0007049 // cell cycle // inferred from electronic annotation///0007059 // chromosome s
NM_0011	0.0106			1.05	-1.18	-1.10	-1.08	***Cbfa2t3***	core-binding factor, runt domain, alpha subunit 2, translocated to, 3 (human)	0000122 // negative regulation of transcription from RNA polymerase II promoter // infer
NM_009430			0.014	-1.01	1.04	-1.10	-1.18	***Prss2***	protease, serine 2	0006508 // proteolysis // not recorded///0007584 // response to nutrient // inferred fror
NM_0095	0.041			1.27	-1.44	-1.10	-1.13	***Plagl1***	pleiomorphic adenoma gene-like 1	0006351 // transcription, DNA-templated // inferred from electronic annotation///00063
	0.0283			-1.05	-1.19	-1.10	-1.23	***BC038268***	cDNA sequence BC038268	0001503 // ossification // inferred from electronic annotation///0001822 // kidney devel
NM_0254	0.0274			-1.01	-1.28	-1.10	-1.17	***Arl4d***	ADP-ribosylation factor-like 4D	0007264 // small GTPase mediated signal transduction // inferred from electronic annota
NM_0096	0.0444	0.013	0.017	-1.99	-1.23	-1.10	-1.08	***Alb***	albumin	0001895 // retina homeostasis // inferred from electronic annotation///0006810 // trans
NM_1453	0.024	0.041		1.07	-1.09	-1.10	-1.05	***Mrgprf***	MAS-related GPR, member F	0007165 // signal transduction // inferred from electronic annotation///0007186 // G-pro
NM_133980///XM		0.038	0.011	1.06	-1.06	-1.10	-1.16	***Slc22a13***	solute carrier family 22 (organic cation transporter), member 13	0015747 // urate transport // not recorded///0055085 // transmembrane transport // no
NM_0290	######			-1.03	-1.39	-1.10	-1.11	***Spsb1***	splA/ryanodine receptor domain and SOCS box containing 1	0016567 // protein ubiquitination // inferred from electronic annotation///0035556 // in
NM_021316///XM_00653			0.024	1.05	-1.01	-1.10	-1.14	***Cend1***	cell cycle exit and neuronal differentiation 1	0007628 // adult walking behavior // inferred from mutant phenotype///0021549 // cere
NM_144790///XM_00652			0.043	1.04	-1.09	-1.10	-1.20	***Ankrd33***	ankyrin repeat domain 33	0000122 // negative regulation of transcription from RNA polymerase II promoter // infer
XM_006504541///XM_006			0.043	1.02	-1.03	-1.10	-1.21	***Muc3***	mucin 3, intestinal	0019725 // cellular homeostasis // traceable author statement
NM_144820///XM_00651			0.036	1.02	-1.08	-1.10	-1.14	***Ccdc28a***	coiled-coil domain containing 28A	
NM_0012	0.002			1.03	-1.04	-1.10	1.22	***Ager***	advanced glycosylation end product-specific receptor	0001666 // response to hypoxia // inferred from mutant phenotype///0001933 // negati
NM_028625			0.038	1.02	-1.11	-1.10	-1.17	***Lce1a2***	late cornified envelope 1A2	0008544 // epidermis development // inferred from electronic annotation///0018149 //
NM_013822///XM_01123			0.029	-1.05	-1.01	-1.10	1.03	***Jag1***	jagged 1	0001974 // blood vessel remodeling // inferred from mutant phenotype///0002011 // mo
NM_058214///XM_00652			0.028	1.03	1.03	-1.10	-1.14	***Recql4***	RecQ protein-like 4	0000733 // DNA strand renaturation // not recorded///0001501 // skeletal system develo
NM_1455	0.027			1.13	-1.04	-1.10	-1.06	***Ccm2l***	cerebral cavernous malformation 2-like	0003209 // cardiac atrium morphogenesis // inferred from genetic interaction///0003222
NM_021452///XM		0.007		1.19	1.05	-1.10	1.09	***Kcnmb4***	potassium large conductance calcium-activated channel, subfamily M, beta member 4	0001508 // action potential // not recorded///0005513 // detection of calcium ion // not
NM_011434		0.018		-1.01	-1.11	-1.10	-1.13	***Sod1***	superoxide dismutase 1, soluble	0000187 // activation of MAPK activity // inferred from direct assay///0000302 // respon
NM_026273///XM		0.024		1.10	-1.06	-1.10	-1.07	***4930453N24Rik***	RIKEN cDNA 4930453N24 gene	0006915 // apoptotic process // inferred from electronic annotation
NM_0010	0.0355			1.06	-1.19	-1.10	-1.03	***Rgs11***	regulator of G-protein signaling 11	0007186 // G-protein coupled receptor signaling pathway // traceable author statement/
NM_0012	0.0239			-1.04	1.12	-1.10	-1.17	***Socs1***	suppressor of cytokine signaling 1	0001932 // regulation of protein phosphorylation // inferred from direct assay///000646
NM_0010	0.0264			1.27	-1.21	-1.10	1.62	***Gpr155***	G protein-coupled receptor 155	0035556 // intracellular signal transduction // inferred from electronic annotation///005
NM_0087	0.0437			1.02	-1.08	-1.10	-1.09	***Pcp4***	Purkinje cell protein 4	0006469 // negative regulation of protein kinase activity // not recorded///0010976 // po
NM_1789	0.0033			-1.08	1.50	-1.10	-1.05	***Pld4***	phospholipase D family, member 4	0002244 // hematopoietic progenitor cell differentiation // inferred from mutant phenoty
NM_011437///XM_00652			0.002	1.03	1.00	-1.09	-1.09	***Sox10***	SRY (sex determining region Y)-box 10	0001701 // in utero embryonic development // inferred from genetic interaction///0001
NM_028216			0.001	1.06	1.06	-1.09	-1.17	***Psca***	prostate stem cell antigen	
NM_026716			0.033	-1.00	1.01	-1.09	-1.11	***Sycn***	syncollin	0006887 // exocytosis // inferred from electronic annotation
NM_012013///XM_00650			0.026	-1.07	1.01	-1.09	-1.33	***Figla***	folliculogenesis specific basic helix-loop-helix	0006351 // transcription, DNA-templated // inferred from electronic annotation///00063
NM_0287	0.0144			-1.02	-1.15	-1.09	-1.17	***Slc7a13***	solute carrier family 7, (cationic amino acid transporter, y+ system) member 13	0003333 // amino acid transmembrane transport // not recorded///0006810 // transpor
NM_0296	0.0064	0.003		1.10	-1.00	-1.09	1.06	***Abhd14b***	abhydrolase domain containing 14b	0008152 // metabolic process // not recorded///0045944 // positive regulation of transc
NM_027454///NM_17321			0.024	1.07	-1.07	-1.09	-1.18	***Chrnb3***	cholinergic receptor, nicotinic, beta polypeptide 3	0006810 // transport // inferred from electronic annotation///0006811 // ion transport /
NM_019741///XM		0.037	0.003	1.07	1.01	-1.09	-1.16	***Slc2a5***	solute carrier family 2 (facilitated glucose transporter), member 5	0006810 // transport // inferred from electronic annotation///0008643 // carbohydrate t
NM_008818			0.033	-1.05	-1.02	-1.09	-1.23	***Rhox5***	reproductive homeobox 5	0000122 // negative regulation of transcription from RNA polymerase II promoter // infer
NM_001081373///		0.027	0.001	1.05	1.04	-1.09	-1.23	***Cep164***	centrosomal protein 164	0006281 // DNA repair // inferred from electronic annotation///0006974 // cellular respo
NM_1455	0.0293	0.032	0.036	1.04	-1.08	-1.09	-1.09	***Mypop***	Myb-related transcription factor, partner of profilin	0000122 // negative regulation of transcription from RNA polymerase II promoter // infer
NM_0011	0.041			1.09	-1.22	-1.09	1.13	***Trim32***	tripartite motif-containing 32	0000209 // protein polyubiquitination // not recorded///0001894 // tissue homeostasis /

RefSeq Tr	*p* 24h	*p* 48h	*p* 72h	FD Air	FD 24h	FD 48h	FD 72h	Gene Symbol	Gene Title	Gene Ontology Biological Process
NM_008410		0.031		1.01	-1.03	-1.09	-1.03	***Itm2b***	integral membrane protein 2B	0042985 // negative regulation of amyloid precursor protein biosynthetic process // not r
NM_0134	0.031			1.02	-1.25	-1.09	1.15	***Aldh1a1***	aldehyde dehydrogenase family 1, subfamily A1	0001822 // kidney development // inferred from electronic annotation///0001889 // live
NM_0093	0.0117			1.17	-1.01	-1.09	1.13	***Inmt***	indolethylamine N-methyltransferase	0009308 // amine metabolic process // not recorded///0009636 // response to toxic subs
NM_0012	0.0371	0.027		1.10	-1.09	-1.09	-1.07	***Slc4a10***	solute carrier family 4, sodium bicarbonate cotransporter-like, member 10	0006486 // protein glycosylation // not recorded///0006810 // transport // inferred from
NM_026602///XM_00650			0.01	-1.21	-1.08	-1.09	1.13	***Bcas2***	breast carcinoma amplified sequence 2	0000398 // mRNA splicing, via spliceosome // not recorded///0006397 // mRNA processi
NM_017373///XM		0.024		-1.53	-1.31	-1.09	-1.34	***Nfil3***	nuclear factor, interleukin 3, regulated	0000122 // negative regulation of transcription from RNA polymerase II promoter // not r
NM_1336	0.0187			1.07	-1.18	-1.09	-1.06	***Ppp1r32***	protein phosphatase 1, regulatory subunit 32	
NM_0010	0.0204			-1.01	-1.20	-1.09	-1.03	***Sorbs1***	sorbin and SH3 domain containing 1	0006810 // transport // inferred from electronic annotation///0007015 // actin filament
NM_0298	0.0201			-1.04	-1.19	-1.09	1.13	***Gpbp1l1***	GC-rich promoter binding protein 1-like 1	0006351 // transcription, DNA-templated // inferred from electronic annotation///00063
NM_0011	0.0433			1.02	1.34	-1.09	-1.03	***Arhgap4***	Rho GTPase activating protein 4	0007165 // signal transduction // inferred from electronic annotation///0010764 // nega
NM_0011	0.001			1.06	-1.11	-1.09	-1.06	***Syt3***	synaptotagmin III	0006906 // vesicle fusion // ---///0016079 // synaptic vesicle exocytosis // not recorded/
NM_025402		0.042		1.01	1.06	-1.09	-1.04	***Ogfod3***	2-oxoglutarate and iron-dependent oxygenase domain containing 3	0055114 // oxidation-reduction process // inferred from electronic annotation
			0.018	-1.02	-1.01	-1.09	-1.15	***D4Ertd117e***	DNA segment, Chr 4, ERATO Doi 117, expressed	
NM_145156///XM		0.014		1.05	1.12	-1.09	-1.11	***Slc25a28***	solute carrier family 25, member 28	0006810 // transport // inferred from electronic annotation///0006811 // ion transport /
NM_0103	######	0.003	0.027	1.08	-1.16	-1.09	-1.06	***H2-Ea-ps***	histocompatibility 2, class II antigen E alpha, pseudogene	0002376 // immune system process // inferred from electronic annotation///0002504 //
NM_001048190///		0.029		1.10	-1.01	-1.09	-1.02	***Agbl5***	ATP/GTP binding protein-like 5	0006508 // proteolysis // inferred from electronic annotation///0035608 // protein deglu
NM_0213	######			1.04	-1.34	-1.09	-1.14	***Klf13***	Kruppel-like factor 13	0006351 // transcription, DNA-templated // inferred from electronic annotation///00063
NM_013809		0.044		1.10	-1.05	-1.09	-1.17	***Cyp2g1***	cytochrome P450, family 2, subfamily g, polypeptide 1	0006805 // xenobiotic metabolic process // not recorded///0019373 // epoxygenase P45
NM_133185///XM_01124			0.009	1.03	1.13	-1.09	-1.11	***Rogdi***	rogdi homolog (Drosophila)	0007420 // brain development // not recorded///0008284 // positive regulation of cell pr
NM_0107	0.0044			1.03	1.80	-1.09	-1.03	***Ly6d***	lymphocyte antigen 6 complex, locus D	0030098 // lymphocyte differentiation // inferred from expression pattern///0035634 //
NM_0012	0.0315	0.024	0.009	1.11	-1.00	-1.09	-1.13	***Pla2g10***	phospholipase A2, group X	0006629 // lipid metabolic process // inferred from electronic annotation///0006644 // p
NM_0215	0.0033			-1.01	1.17	-1.09	1.12	***Chst12***	carbohydrate sulfotransferase 12	0005975 // carbohydrate metabolic process // inferred from electronic annotation///001
NM_0010	0.0268			1.01	-1.12	-1.09	-1.03	***Mrpl44***	mitochondrial ribosomal protein L44	0006396 // RNA processing // inferred from electronic annotation///0070125 // mitocho
NM_0013	0.0322			-1.11	-1.37	-1.09	-1.01	***Figf***	c-fos induced growth factor	0001525 // angiogenesis // inferred from electronic annotation///0001666 // response to
NM_033320///XM_00651			0.024	-1.06	-1.07	-1.09	1.13	***Glce***	glucuronyl C5-epimerase	0006024 // glycosaminoglycan biosynthetic process // inferred from electronic annotatio
NM_001146707///		0.001	0.037	1.05	-1.06	-1.09	-1.03	***Gm4204///Nap1l1***	nucleosome assembly protein 1-like 1 pseudogene///nucleosome assembly protein 1-l	0006334 // nucleosome assembly // inferred from electronic annotation///0014010 // Sc
NM_175133///XM		0.021		1.05	-1.02	-1.09	-1.05	***Gucd1***	guanylyl cyclase domain containing 1	
NM_0011	0.0264	0.033		1.12	-1.06	-1.09	1.21	***Dhrs3***	dehydrogenase/reductase (SDR family) member 3	0001523 // retinoid metabolic process // inferred from mutant phenotype///0003151 //
NM_029360		0.016		1.08	1.08	-1.09	-1.03	***Tm4sf5***	transmembrane 4 superfamily member 5	
NM_0294	0.0042			-1.07	-1.37	-1.09	-1.09	***Them4***	thioesterase superfamily member 4	0006629 // lipid metabolic process // inferred from electronic annotation///0006631 // f
NM_0261	0.0326		0.038	1.07	-1.13	-1.09	-1.11	***Ppp1r2-ps9***	protein phosphatase 1, regulatory (inhibitor) subunit 2, pseudogene 9	
NM_0080	0.0073			1.01	-1.29	-1.09	-1.08	***Gata2***	GATA binding protein 2	0000122 // negative regulation of transcription from RNA polymerase II promoter // infer
NM_0187	0.005			-1.04	-1.42	-1.09	1.08	***Cpq***	carboxypeptidase Q	0006508 // proteolysis // not recorded///0006590 // thyroid hormone generation // not
NM_001025599///		0.011	0.041	1.07	-1.01	-1.09	-1.03	***Trim26***	tripartite motif-containing 26	0045087 // innate immune response // not recorded///0046597 // negative regulation of
NM_010791			0.008	-1.01	-1.02	-1.09	-1.17	***Meox1***	mesenchyme homeobox 1	0001757 // somite specification // inferred from genetic interaction///0006351 // transcr
NM_011246			0.014	1.03	-1.01	-1.09	-1.13	***Rasgrp1***	RAS guanyl releasing protein 1	0001816 // cytokine production // inferred from mutant phenotype///0001934 // positiv
NM_025974///NR_110499			0.028	-1.07	-1.02	-1.09	-1.12	***Rpl14///Rpl14-ps1***	ribosomal protein L14///ribosomal protein L14, pseudogene 1	0006364 // rRNA processing // not recorded///0006412 // translation // inferred from el
NM_0010	0.0076		0.038	1.11	-1.06	-1.09	1.01	***Mink1***	misshapen-like kinase 1 (zebrafish)	0000165 // MAPK cascade // not recorded///0001952 // regulation of cell-matrix adhesi
NM_0012	0.0372			1.06	-1.06	-1.09	-1.21	***Dtx2***	deltex 2 homolog (Drosophila)	0007219 // Notch signaling pathway // inferred from direct assay///0007219 // Notch sig
NM_0113	0.0122			1.06	1.41	-1.09	-1.30	***Spi1***	spleen focus forming virus (SFFV) proviral integration oncogene	0000122 // negative regulation of transcription from RNA polymerase II promoter // infer
NM_0012	0.001			1.04	1.52	-1.08	1.04	***Lrmp***	lymphoid-restricted membrane protein	0002376 // immune system process // inferred from electronic annotation///0007338 //
NM_177242		0.043		1.04	-1.08	-1.08	1.00	***Pptc7***	PTC7 protein phosphatase homolog (S. cerevisiae)	0006470 // protein dephosphorylation // inferred from electronic annotation///0008152
NM_0106	0.0204			1.00	1.25	-1.08	1.02	***Lcp2***	lymphocyte cytosolic protein 2	0006955 // immune response // not recorded///0007169 // transmembrane receptor pro
NM_0117	0.0275			-1.11	-1.00	-1.08	-1.16	***Xcr1***	chemokine (C motif) receptor 1	0006935 // chemotaxis // inferred from direct assay///0007165 // signal transduction // i
NM_008763			0.045	-1.08	-1.20	-1.08	-1.01	***Olfr16***	olfactory receptor 16	0007165 // signal transduction // inferred from electronic annotation///0007186 // G-pro
NM_0010	0.0154			1.08	-1.13	-1.08	-1.05	***Lgr6***	leucine-rich repeat-containing G protein-coupled receptor 6	0007165 // signal transduction // inferred from electronic annotation///0007186 // G-pro
NM_031379			0.038	1.03	-1.04	-1.08	-1.15	***Tktl1***	transketolase-like 1	0008152 // metabolic process // inferred from electronic annotation
NM_016875///XM		0.033		1.11	1.02	-1.08	-1.12	***Ybx2***	Y box protein 2	0006355 // regulation of transcription, DNA-templated // inferred from electronic annota
NM_008911///XM		0.037		1.08	1.04	-1.08	-1.00	***Ppox***	protoporphyrinogen oxidase	0006779 // porphyrin-containing compound biosynthetic process // not recorded///0006
NM_009314			0.033	1.03	-1.03	-1.08	-1.11	***Tacr2***	tachykinin receptor 2	0007165 // signal transduction // inferred from electronic annotation///0007166 // cell s
NM_0084	0.0474			-1.08	1.02	-1.08	-1.12	***Krt4***	keratin 4	0007010 // cytoskeleton organization // not recorded///0009790 // embryo developmen
NM_0217	0.0358			1.06	1.23	-1.08	1.15	***Myg1***	melanocyte proliferating gene 1	0035641 // locomotory exploration behavior // inferred from mutant phenotype
NM_001177773///NM_00			0.017	-1.14	-1.20	-1.08	-1.05	***Fbxw7***	F-box and WD-40 domain protein 7	0001570 // vasculogenesis // inferred from mutant phenotype///0001944 // vasculature
NM_0274	0.0232			1.03	-1.19	-1.08	1.12	***Zcchc8***	zinc finger, CCHC domain containing 8	0006397 // mRNA processing // inferred from electronic annotation///0008380 // RNA sp
NM_1489	0.0441			-1.06	1.02	-1.08	-1.21	***Usp9y***	ubiquitin specific peptidase 9, Y chromosome	0006508 // proteolysis // inferred from electronic annotation///0006511 // ubiquitin-dep
NM_0011	0.0171		0.039	-1.00	1.10	-1.08	-1.12	***Ppfia4***	protein tyrosine phosphatase, receptor type, f polypeptide (PTPRF), interacting protein (liprin), alpha 4	
NM_001113408///		0.028		1.34	1.32	-1.08	1.13	***Ldb1***	LIM domain binding 1	0000972 // transcription-dependent tethering of RNA polymerase II gene DNA at nuclear
NM_030890///XM_00652			0.003	1.03	-1.06	-1.08	-1.14	***Prrt1***	proline-rich transmembrane protein 1	0009607 // response to biotic stimulus // inferred from electronic annotation
NM_0269	0.0273			1.05	-1.03	-1.08	1.00	***Snx15***	sorting nexin 15	0006810 // transport // inferred from electronic annotation///0007165 // signal transduc
NM_001316710///NM_01			0.039	1.01	-1.01	-1.08	-1.16	***Gprin1***	G protein-regulated inducer of neurite outgrowth 1	0031175 // neuron projection development // inferred from direct assay
NM_001276298///NM_00			0.007	1.10	1.01	-1.08	-1.15	***Avpr2***	arginine vasopressin receptor 2	0001992 // regulation of systemic arterial blood pressure by vasopressin // not recorded/
NM_0285	0.0428		0.025	-1.09	1.02	-1.08	-1.26	***1700080E11Rik***	RIKEN cDNA 1700080E11 gene	0008152 // metabolic process // inferred from electronic annotation
NM_001272024///NM_01			#####	1.03	1.01	-1.08	-1.17	***Sema6c***	sema domain, transmembrane domain (TM), and cytoplasmic domain, (semaphorin) 6	0001755 // neural crest cell migration // not recorded///0007275 // multicellular organis
NM_0110	0.0235			-1.09	1.07	-1.08	-1.13	***Otp***	orthopedia homolog (Drosophila)	0002052 // positive regulation of neuroblast proliferation // inferred from mutant pheno

RefSeq Tr	*p* 24h	*p* 48h	*p* 72h	FD Air	FD 24h	FD 48h	FD 72h	Gene Symbol	Gene Title	Gene Ontology Biological Process
NM_0012	0.0115			1.08	-1.26	-1.08	1.07	***Bcl2l1***	BCL2-like 1	0000910 // cytokinesis // not recorded///0001541 // ovarian follicle development // infe
NM_0087	0.0499			1.05	-1.41	-1.08	1.01	***Ogn***	osteoglycin	0018146 // keratan sulfate biosynthetic process // not recorded///0035385 // Roundabo
NM_0195	0.0176		0.022	1.10	-1.08	-1.08	-1.16	***Plac1***	placental specific protein 1	0001890 // placenta development // inferred from expression pattern///0001890 // plac
NM_0011	0.0024			1.21	-1.06	-1.08	1.10	***Ngef***	neuronal guanine nucleotide exchange factor	0007275 // multicellular organismal development // inferred from electronic annotation/
NM_0013	0.0485	0.041		1.02	-1.08	-1.08	-1.06	***Rab7b***	RAB7B, member RAS oncogene family	0006810 // transport // inferred from electronic annotation///0006886 // intracellular pr
NM_145746///XM_00653			0.017	1.10	-1.02	-1.08	-1.10	***Odf4***	outer dense fiber of sperm tails 4	0007275 // multicellular organismal development // inferred from electronic annotation/
NM_001293630///		0.014		1.13	1.08	-1.08	-1.01	***Slc37a4***	solute carrier family 37 (glucose-6-phosphate transporter), member 4	0001780 // neutrophil homeostasis // inferred from mutant phenotype///0001816 // cyt
NM_0012	0.0032			1.11	1.58	-1.08	1.02	***Cd37***	CD37 antigen	0002639 // positive regulation of immunoglobulin production // inferred from mutant ph
NM_0010	######			-1.02	-1.40	-1.08	1.12	***Fam178a***	family with sequence similarity 178, member A	0006281 // DNA repair // inferred from electronic annotation///0006974 // cellular respo
NM_001267625///NM_00			0.018	1.05	-1.02	-1.08	-1.17	***Dpf3***	D4, zinc and double PHD fingers, family 3	0002230 // positive regulation of defense response to virus by host // not recorded///000
NM_0202	0.0171			1.06	-1.14	-1.08	-1.18	***Klk1b27***	kallikrein 1-related peptidase b27	0002526 // acute inflammatory response // ---///0003073 // regulation of systemic arteri
NM_023910///NM		0.005		1.14	1.15	-1.08	-1.24	***Tsc22d4***	TSC22 domain family, member 4	0006351 // transcription, DNA-templated // inferred from electronic annotation///00063
NM_0105	0.0483			-1.03	1.29	-1.08	1.28	***Ifit3***	interferon-induced protein with tetratricopeptide repeats 3	0002376 // immune system process // inferred from electronic annotation///0008285 //
NM_1462	0.0251			1.04	-1.26	-1.08	1.04	***Wdr78***	WD repeat domain 78	0002244 // hematopoietic progenitor cell differentiation // inferred from mutant phenoty
NM_0012	0.0271			-1.08	1.04	-1.08	-1.26	***Wnk2***	WNK lysine deficient protein kinase 2	0006468 // protein phosphorylation // inferred from direct assay///0006468 // protein p
NM_026780		0.045		1.04	-1.03	-1.08	1.17	***Syf2***	SYF2 homolog, RNA splicing factor (S. cerevisiae)	0000398 // mRNA splicing, via spliceosome // not recorded///0001701 // in utero embry
NM_0231	0.0062			1.03	-1.10	-1.08	-1.12	***Ctrl***	chymotrypsin-like	0006508 // proteolysis // inferred from direct assay///0006508 // proteolysis // not recor
		0.021		1.19	1.04	-1.08	1.02	***D12Ertd123e***	DNA segment, Chr 12, ERATO Doi 123, expressed	
NM_013730///XM_00649			0.026	1.06	1.09	-1.08	-1.14	***Slamf1***	signaling lymphocytic activation molecule family member 1	0001779 // natural killer cell differentiation // inferred from mutant phenotype///00017
NM_028953///XM_00652			0.048	1.03	-1.02	-1.08	-1.17	***Tmc1***	transmembrane channel-like gene family 1	0006810 // transport // inferred from electronic annotation///0006811 // ion transport /
NM_013651///XM		0.047		1.13	1.07	-1.08	-1.12	***Sf3a2***	splicing factor 3a, subunit 2	0000245 // spliceosomal complex assembly // not recorded///0000375 // RNA splicing, v
NM_0119	0.0361	0.009		1.12	-1.05	-1.08	-1.14	***Sema4g***	sema domain, immunoglobulin domain (Ig), transmembrane domain (TM) and short cy	0001755 // neural crest cell migration // not recorded///0007275 // multicellular organis
NM_019975///XM		0.035		1.04	-1.02	-1.08	-1.06	***Hacl1///Colq///LOC102639719***	2-hydroxyacyl-CoA lyase 1///collagen-like tail subunit (single strand of homotrimer) of	0001561 // fatty acid alpha-oxidation // not recorded///0006629 // lipid metabolic proce
NM_0120	0.0378			1.10	-1.14	-1.08	-1.11	***Ppnr***	per-pentamer repeat gene	
NM_0010	0.0255			-1.01	1.35	-1.08	-1.10	***Hvcn1***	hydrogen voltage-gated channel 1	0006810 // transport // inferred from electronic annotation///0006811 // ion transport /
NM_1531	0.009			1.03	-1.10	-1.08	1.10	***Svil***	supervillin	0007010 // cytoskeleton organization // inferred from electronic annotation///0007519 /
NM_007745			0.038	1.03	1.05	-1.08	-1.15	***Cort***	cortistatin	0007193 // adenylate cyclase-inhibiting G-protein coupled receptor signaling pathway //
NM_008429///XM_00649			0.049	1.07	-1.02	-1.08	-1.22	***Kcnj9***	potassium inwardly-rectifying channel, subfamily J, member 9	0006810 // transport // inferred from electronic annotation///0006811 // ion transport /
NM_029938		0.028		1.09	1.01	-1.08	-1.10	***H2afv***	H2A histone family, member V	0006325 // chromatin organization // not recorded///0006342 // chromatin silencing //
NM_001037940///		0.018		1.05	-1.06	-1.08	1.05	***Dnajb6***	DnaJ (Hsp40) homolog, subfamily B, member 6	0006457 // protein folding // not recorded///0030036 // actin cytoskeleton organization
NM_001289507///NM_00			0.029	-1.02	-1.04	-1.08	-1.24	***Tfr2***	transferrin receptor 2	0006879 // cellular iron ion homeostasis // traceable author statement///0006898 // rec
NM_0266	0.0033	0.039		1.04	-1.15	-1.08	1.02	***Rbm28***	RNA binding motif protein 28	0006397 // mRNA processing // inferred from electronic annotation///0008380 // RNA sp
NM_011134///XM		0.022		1.12	-1.05	-1.08	1.29	***Pon1***	paraoxonase 1	0006470 // protein dephosphorylation // ---///0006470 // protein dephosphorylation // i
NM_025595		0.047	0.042	1.04	-1.00	-1.08	-1.08	***Mrpl51***	mitochondrial ribosomal protein L51	0006412 // translation // inferred from direct assay///0032543 // mitochondrial translati
NM_144919///XM		0.014		1.11	1.07	-1.08	-1.02	***Hdac11***	histone deacetylase 11	0006351 // transcription, DNA-templated // inferred from electronic annotation///00063
NM_015809		0.041		1.06	1.00	-1.08	-1.10	***Krtap5-4***	keratin associated protein 5-4	
NM_010030			0.005	1.02	-1.02	-1.08	-1.21	***Defb2***	defensin beta 2	0006952 // defense response // inferred from electronic annotation///0042742 // defens
NM_001164607///		0.034		1.04	1.07	-1.08	-1.10	***Maf1***	MAF1 homolog (S. cerevisiae)	0006351 // transcription, DNA-templated // inferred from electronic annotation///00063
NM_0093	0.0466			-1.05	-1.48	-1.08	1.04	***Thbd***	thrombomodulin	0007165 // signal transduction // inferred from electronic annotation///0007565 // fema
NM_145479///XM		0.02		1.03	-1.05	-1.08	-1.07	***Klhl22***	kelch-like 22	0000070 // mitotic sister chromatid segregation // not recorded///0006513 // protein mo
NM_001278581///NM_00			0.038	1.06	1.07	-1.08	-1.13	***Pomc***	pro-opiomelanocortin-alpha	0006091 // generation of precursor metabolites and energy // not recorded///0007165 /
NM_019663///XM		0.023		1.11	-1.01	-1.08	1.16	***Pias1***	protein inhibitor of activated STAT 1	0000082 // G1/S transition of mitotic cell cycle // not recorded///0000122 // negative reg
NM_148953			0.019	1.03	-1.01	-1.07	-1.14	***Asb16***	ankyrin repeat and SOCS box-containing 16	0016567 // protein ubiquitination // inferred from electronic annotation///0035556 // in
NM_0011	0.0428			1.09	-1.07	-1.07	-1.10	***Dctn1***	dynactin 1	0006810 // transport // inferred from electronic annotation///0008152 // metabolic proc
NM_019701///XM_00653			0.04	-1.01	1.03	-1.07	-1.20	***Clcnkb***	chloride channel, voltage-sensitive Kb	0006810 // transport // inferred from electronic annotation///0006811 // ion transport /
NM_145564///XM		0.008	0.046	1.12	1.03	-1.07	-1.03	***Fbxo21***	F-box protein 21	
NM_001013360///		0.014		1.02	-1.01	-1.07	-1.10	***Npcd///Nptxr***	neuronal pentraxin chromo domain///neuronal pentraxin receptor	0000122 // negative regulation of transcription from RNA polymerase II promoter // infer
NM_009620			0.038	1.05	1.00	-1.07	-1.06	***Adam4***	a disintegrin and metallopeptidase domain 4	0006508 // proteolysis // inferred from electronic annotation///0007229 // integrin-med
NM_001040136///NM_00			0.049	-1.01	-1.02	-1.07	-1.16	***Pdzd9***	PDZ domain containing 9	
XM_9935	0.0102		0.002	1.07	1.20	-1.07	-1.16	***LOC672450///Igk-V21///Igk-V28///Igk-V8/***	V(kappa) gene product///immunoglobulin kappa chain variable 21 (V21)///immunogl	0006898 // receptor-mediated endocytosis // not recorded///0006910 // phagocytosis, r
NM_009438///NM_01352			0.047	-1.01	1.04	-1.07	-1.05	***Rpl13a///Flt3l///Gm11478***	ribosomal protein L13A///FMS-like tyrosine kinase 3 ligand///60S ribosomal protein L1	0006412 // translation // inferred from electronic annotation///0006417 // regulation of
NM_025414///XM_00653			0.013	-1.09	-1.04	-1.07	1.05	***Myo19***	myosin XIX	0008152 // metabolic process // inferred from electronic annotation///0034642 // mitoc
NM_001159520///NM_00			0.002	-1.00	-1.03	-1.07	-1.18	***Pax3***	paired box 3	0000122 // negative regulation of transcription from RNA polymerase II promoter // infer
NM_0264	0.0431			-1.16	-1.07	-1.07	-1.14	***Mtfp1***	mitochondrial fission process 1	0000266 // mitochondrial fission // not recorded///0006915 // apoptotic process // infer
NM_134000///XM		0.005	0.004	1.10	1.11	-1.07	-1.08	***Traf3ip2***	TRAF3 interacting protein 2	0001783 // B cell apoptotic process // inferred from mutant phenotype///0002230 // pos
NM_0265	0.0233			1.16	-1.06	-1.07	-1.09	***Bambi***	BMP and activin membrane-bound inhibitor	0007179 // transforming growth factor beta receptor signaling pathway // not recorded/
NM_0120	0.0346			-1.04	1.08	-1.07	-1.14	***Pla2g2e***	phospholipase A2, group IIE	0006629 // lipid metabolic process // inferred from electronic annotation///0006644 // p
NM_001166642///		0.042		1.10	-1.01	-1.07	-1.01	***Bcas3***	breast carcinoma amplified sequence 3	0001525 // angiogenesis // inferred from electronic annotation///0006351 // transcriptic
NM_0011	0.034			1.02	-1.05	-1.07	-1.04	***Prph***	peripherin	0045104 // intermediate filament cytoskeleton organization // inferred from direct assay
NM_1308	0.0147	0.002		1.05	-1.03	-1.07	-1.06	***Nxph3***	neurexophilin 3	
NM_0012	0.0273			1.03	-1.14	-1.07	-1.02	***Pbx1***	pre B cell leukemia homeobox 1	0001655 // urogenital system development // inferred from mutant phenotype///00016
NM_1341	0.0311		0.048	-1.05	1.15	-1.07	1.07	***Pla1a***	phospholipase A1 member A	0006629 // lipid metabolic process // inferred from electronic annotation///0016042 // li
NM_001289729///NM_13			0.028	-1.01	-1.14	-1.07	-1.16	***Xpnpep2***	X-prolyl aminopeptidase (aminopeptidase P) 2, membrane-bound	0006508 // proteolysis // inferred from electronic annotation///0008152 // metabolic pr
NM_0010	0.0472			1.05	-1.05	-1.07	-1.24	***Ccdc102a***	coiled-coil domain containing 102A	0008152 // metabolic process // inferred from electronic annotation

RefSeq Tr	p 24h	p 48h	p 72h	FD Air	FD 24h	FD 48h	FD 72h	Gene Symbol	Gene Title	Gene Ontology Biological Process
NM_023697///XM_		0.025		1.03	-1.06	-1.07	-1.09	***Rdh14***	retinol dehydrogenase 14 (all-trans and 9-cis)	0001649 // osteoblast differentiation // not recorded///0008152 // metabolic process //
NM_0113	0.0314			1.06	-1.15	-1.07	1.26	***Scnn1a***	sodium channel, nonvoltage-gated 1 alpha	0006810 // transport // inferred from electronic annotation///0006811 // ion transport /
NM_145940		0.044		1.02	-1.03	-1.07	1.02	***Wipi1***	WD repeat domain, phosphoinositide interacting 1	0000045 // autophagosome assembly // not recorded///0000422 // mitophagy // not rec
NM_029624///NR_045520			0.031	-1.03	1.03	-1.07	1.13	***Lmf1***	lipase maturation factor 1	0006888 // ER to Golgi vesicle-mediated transport // inferred from mutant phenotype///
NM_0011	0.0117			-1.01	1.09	-1.07	-1.12	***Dbn1***	drebrin 1	0007015 // actin filament organization // inferred from direct assay///0007275 // multice
NM_0011	0.0487			-1.03	-1.32	-1.07	1.06	***Lpin1***	lipin 1	0000122 // negative regulation of transcription from RNA polymerase II promoter // infer
NM_138655			0.043	1.02	-1.01	-1.07	-1.12	***Tmc2***	transmembrane channel-like gene family 2	0006810 // transport // inferred from electronic annotation///0006811 // ion transport /
NM_144523		0.02		1.01	-1.03	-1.07	-1.22	***Zfp622***	zinc finger protein 622	0008631 // intrinsic apoptotic signaling pathway in response to oxidative stress // not rec
NM_148938			0.031	-1.08	-1.04	-1.07	1.04	***Slc1a3***	solute carrier family 1 (glial high affinity glutamate transporter), member 3	0002230 // positive regulation of defense response to virus by host // not recorded///000
NM_1455	0.0162	0.009		1.03	-1.21	-1.07	1.03	***Tmem159***	transmembrane protein 159	
NM_0111	0.0488			-1.03	1.27	-1.07	-1.04	***Pstpip1***	proline-serine-threonine phosphatase-interacting protein 1	0000910 // cytokinesis // inferred by curator///0002376 // immune system process // inf
NM_008803		0.009		1.11	-1.06	-1.07	1.11	***Pde8a***	phosphodiesterase 8A	0006198 // cAMP catabolic process // inferred from electronic annotation///0006355 //
NM_0088	0.0328			1.11	-1.06	-1.07	1.11	***Pde8a***	phosphodiesterase 8A	0006198 // cAMP catabolic process // inferred from electronic annotation///0006355 //
NM_0294	0.0159			-1.05	1.09	-1.07	-1.03	***Epsti1***	epithelial stromal interaction 1 (breast)	
NM_1528	0.025	0.003		1.11	-1.04	-1.07	1.05	***Ccdc137***	coiled-coil domain containing 137	
NM_0168	0.0478			-1.12	1.03	-1.07	-1.14	***Avpr1a***	arginine vasopressin receptor 1A	0001992 // regulation of systemic arterial blood pressure by vasopressin // inferred from
NM_011973///XM_00651			0.018	1.03	1.03	-1.07	-1.12	***Mok***	MOK protein kinase	0006468 // protein phosphorylation // inferred from electronic annotation///0016310 //
NM_024230///XM_00650			0.04	1.06	-1.08	-1.07	-1.23	***Smtnl1***	smoothelin-like 1	0014823 // response to activity // inferred from mutant phenotype///0018105 // peptid
NM_0013	0.0339			-1.03	-1.18	-1.07	1.15	***Nek7***	NIMA (never in mitosis gene a)-related expressed kinase 7	0006468 // protein phosphorylation // not recorded///0016310 // phosphorylation // inf
NM_0299	0.0435			-1.08	-1.02	-1.07	-1.10	***Sla2***	Src-like-adaptor 2	0000122 // negative regulation of transcription from RNA polymerase II promoter // not
NM_0198	0.0114			-1.03	1.20	-1.07	1.03	***Gsk3b***	glycogen synthase kinase 3 beta	0000320 // re-entry into mitotic cell cycle // inferred from direct assay///0001837 // epit
NM_001130419///		0.022	0.003	1.15	-1.03	-1.07	-1.24	***Hpca***	hippocalcin	0007420 // brain development // inferred from electronic annotation///0010518 // posit
NM_007811			0.014	-1.04	1.01	-1.07	-1.24	***Cyp26a1***	cytochrome P450, family 26, subfamily a, polypeptide 1	0007417 // central nervous system development // inferred from genetic interaction///0
NM_008899		0.047		1.04	-1.03	-1.07	-1.07	***Pou3f2***	POU domain, class 3, transcription factor 2	0006351 // transcription, DNA-templated // inferred from electronic annotation///00063
NM_030714///XM		0.045		1.15	1.02	-1.07	1.22	***Dtx3***	deltex 3 homolog (Drosophila)	0007219 // Notch signaling pathway // inferred from direct assay///0007219 // Notch sig
NM_0220	######			-1.01	1.14	-1.07	1.05	***Dusp10***	dual specificity phosphatase 10	0000188 // inactivation of MAPK activity // not recorded///0002819 // regulation of ada
NM_0264	0.0339			-1.05	1.07	-1.07	-1.01	***Coq9***	coenzyme Q9 homolog (yeast)	0006120 // mitochondrial electron transport, NADH to ubiquinone // inferred from muta
NM_1789	0.0449			-1.04	-1.36	-1.07	1.05	***Mal2***	mal, T cell differentiation protein 2	
NM_1813	0.0082		0.021	1.09	-1.10	-1.07	-1.15	***Cwh43***	cell wall biogenesis 43 C-terminal homolog (S. cerevisiae)	0006506 // GPI anchor biosynthetic process // inferred from electronic annotation
NM_1980	######			1.04	-1.08	-1.07	-1.15	***Tnrc6c***	trinucleotide repeat containing 6C	0001706 // endoderm formation // inferred from mutant phenotype///0006417 // regula
NM_001313928///NM_00			0.009	-1.02	1.01	-1.07	-1.15	***Crhr1***	corticotropin releasing hormone receptor 1	0001666 // response to hypoxia // not recorded///0007165 // signal transduction // infe
NM_0260	0.0489			1.01	-1.12	-1.07	-1.02	***Gemin6***	gem (nuclear organelle) associated protein 6	0000245 // spliceosomal complex assembly // inferred from electronic annotation///000
NM_144867///XM_00652			0.038	-1.03	-1.03	-1.07	-1.14	***Slmo1***	slowmo homolog 1 (Drosophila)	0015914 // phospholipid transport // not recorded///1903955 // positive regulation of p
NM_033146///XM		0.007	0.024	1.16	1.11	-1.07	-1.21	***Emc9***	ER membrane protein complex subunit 9	
NM_0085	0.0453			-1.01	1.06	-1.07	-1.19	***Mageb1///Mageb2///Mageb3***	melanoma antigen, family B, 1///melanoma antigen, family B, 2///melanoma antigen, family B, 3	
NM_001039509///NM_01			0.007	1.06	-1.11	-1.07	-1.12	***Pnkd***	paroxysmal nonkinesiogenic dyskinesia	0006750 // glutathione biosynthetic process // inferred from electronic annotation///001
NM_0011	0.0471		0.023	1.03	-1.07	-1.07	-1.13	***Ak4***	adenylate kinase 4	0001889 // liver development // inferred from electronic annotation///0006139 // nucle
NM_0116	0.0261			-1.04	-1.11	-1.07	-1.06	***Uba1y***	ubiquitin-activating enzyme, Chr Y	0006464 // cellular protein modification process // inferred from electronic annotation//
NM_0226	0.0035	0.008		1.06	-1.09	-1.07	-1.08	***Nisch***	nischarin	0006006 // glucose metabolic process // inferred from electronic annotation///0006915
NM_001302393///		0.039		1.04	-1.02	-1.07	1.02	***Ppt2***	palmitoyl-protein thioesterase 2	0098734 // macromolecule depalmitoylation // not recorded
NM_0013	0.0275			1.02	-1.11	-1.07	1.05	***Kifap3***	kinesin-associated protein 3	0007017 // microtubule-based process // inferred from physical interaction///0008104 /
NM_1304	0.0145			1.05	-1.14	-1.07	-1.06	***Cntnap4***	contactin associated protein-like 4	0007155 // cell adhesion // inferred from electronic annotation///0032225 // regulation
NM_0010	0.0119			-1.06	1.10	-1.07	-1.10	***Pik3cd***	phosphatidylinositol 3-kinase catalytic delta polypeptide	0001782 // B cell homeostasis // inferred from mutant phenotype///0002250 // adaptive
NM_007792			0.02	-1.02	-1.12	-1.06	1.10	***Csrp2***	cysteine and glycine-rich protein 2	0007275 // multicellular organismal development // inferred from electronic annotation/
NM_0099	0.032			-1.03	1.15	-1.06	-1.04	***Cx3cr1***	chemokine (C-X3-C motif) receptor 1	0001774 // microglial cell activation // not recorded///0002052 // positive regulation of
NM_0013	0.0033			1.02	-1.15	-1.06	1.01	***Tnfrsf23***	tumor necrosis factor receptor superfamily, member 23	1902042 // negative regulation of extrinsic apoptotic signaling pathway via death domai
NM_0242	0.035			1.02	-1.07	-1.06	1.05	***LOC102641347///Ndufc2***	NADH dehydrogenase [ubiquinone] 1 subunit C2///NADH dehydrogenase (ubiquinone	0006120 // mitochondrial electron transport, NADH to ubiquinone // inferred from elect
NM_0110	0.0314			-1.00	-1.31	-1.06	1.02	***Pex11a***	peroxisomal biogenesis factor 11 alpha	0007031 // peroxisome organization // not recorded///0007165 // signal transduction //
NM_0011	0.0477			1.17	-1.00	-1.06	-1.02	***Cbarp***	calcium channel, voltage-dependent, beta subunit associated regulatory protein	1901386 // negative regulation of voltage-gated calcium channel activity // not recorded
NM_0010	0.0339			-1.05	1.12	-1.06	-1.10	***Brdt***	bromodomain, testis-specific	0001207 // histone displacement // inferred from mutant phenotype///0006338 // chro
NM_0532	0.01	0.008		1.13	-1.02	-1.06	1.12	***Hsd17b11***	hydroxysteroid (17-beta) dehydrogenase 11	0006629 // lipid metabolic process // inferred from electronic annotation///0006694 //
NM_023816///XM_00651			0.045	1.09	-1.04	-1.06	-1.11	***Ankrd36***	ankyrin repeat domain 36	
NM_0010	0.0251	0.04		1.07	-1.14	-1.06	1.02	***Agap1***	ArfGAP with GTPase domain, ankyrin repeat and PH domain 1	0006810 // transport // inferred from electronic annotation///0007165 // signal transdu
NM_0011	0.021	0.045		1.35	-1.10	-1.06	-1.13	***Ankrd33b***	ankyrin repeat domain 33B	
NM_007845			0.034	-1.05	1.02	-1.06	-1.10	***Defa-rs10***	defensin, alpha, related sequence 10	0006952 // defense response // inferred from electronic annotation
NM_001146021///NM_02			0.016	1.02	1.03	-1.06	-1.22	***Lrrc18***	leucine rich repeat containing 18	
NM_0012	0.0427			-1.08	1.05	-1.06	-1.14	***Kcnab2***	potassium voltage-gated channel, shaker-related subfamily, beta member 2	0002244 // hematopoietic progenitor cell differentiation // inferred from genetic interac
NM_175217///XM_00650			0.032	1.03	-1.09	-1.06	-1.18	***Mmd2***	monocyte to macrophage differentiation-associated 2	0006468 // protein phosphorylation // not recorded///0019835 // cytolysis // inferred fr
NM_0013	0.0433			-1.17	1.05	-1.06	-1.05	***Tuba4a***	tubulin, alpha 4A	0007017 // microtubule-based process // inferred from electronic annotation///0008152
NM_028658		0.03	0.031	1.02	-1.06	-1.06	-1.06	***Ppp1r21***	protein phosphatase 1, regulatory subunit 21	
NM_1456	0.0285			-1.04	-1.11	-1.06	-1.15	***BC021891***	cDNA sequence BC021891	0000165 // MAPK cascade // inferred from electronic annotation///0000186 // activatio
NM_144872///XM		0.019		1.19	1.39	-1.06	1.26	***Eml3***	echinoderm microtubule associated protein like 3	
NM_0103	0.0226			1.00	-1.08	-1.06	1.22	***Gstm1***	glutathione S-transferase, mu 1	0006749 // glutathione metabolic process // not recorded///0008152 // metabolic proce
NM_0109	0.0336	0.043		1.08	-1.15	-1.06	-1.17	***Nfkbia***	nuclear factor of kappa light polypeptide gene enhancer in B cells inhibitor, alpha	0000060 // protein import into nucleus, translocation // inferred from direct assay///000

RefSeq Tr	*p* 24h	*p* 48h	*p* 72h	FD Air	FD 24h	FD 48h	FD 72h	Gene Symbol	Gene Title	Gene Ontology Biological Process
NM_013811///XM_		0.017		1.14	1.05	-1.06	-1.04	***Dnah8***	dynein, axonemal, heavy chain 8	0007018 // microtubule-based movement // inferred from electronic annotation///00081
NM_0010	0.0207			1.09	-1.14	-1.06	-1.08	***Car1***	carbonic anhydrase 1	0006730 // one-carbon metabolic process // inferred from electronic annotation
NM_0198	0.0223			1.01	-1.13	-1.06	-1.18	***Copz2***	coatomer protein complex, subunit zeta 2	0006810 // transport // inferred from electronic annotation///0015031 // protein transp
NM_0089	0.0155			1.03	-1.25	-1.06	1.33	***Ptrf***	polymerase I and transcript release factor	0006351 // transcription, DNA-templated // inferred from electronic annotation///00063
NM_1340	0.0023			1.09	-1.14	-1.06	-1.04	***Chmp7***	charged multivesicular body protein 7	0000920 // cell separation after cytokinesis // not recorded///0006810 // transport // inf
NM_0158	0.0157			1.04	-1.15	-1.06	-1.11	***Capn15***	calpain 15	0006508 // proteolysis // not recorded
NM_0013	0.0351			1.07	-1.15	-1.06	-1.04	***Zfp423***	zinc finger protein 423	0006351 // transcription, DNA-templated // inferred from electronic annotation///00063
NM_001033499///		0.015		1.06	1.09	-1.06	1.07	***Sh2d1b1///Sh2d1b2***	SH2 domain containing 1B1///SH2 domain containing 1B2	0002366 // leukocyte activation involved in immune response // inferred from direct assa
NM_009855///XM_00652			0.015	-1.05	-1.05	-1.06	-1.22	***Cd80***	CD80 antigen	0031295 // T cell costimulation // inferred from direct assay///0042102 // positive regula
NM_0013	0.0459			1.09	-1.05	-1.06	-1.03	***Slc27a3***	solute carrier family 27 (fatty acid transporter), member 3	0001676 // long-chain fatty acid metabolic process // traceable author statement///0006
NM_0220	0.0354			-1.08	1.05	-1.06	-1.09	***Ube4b***	ubiquitination factor E4B	0000209 // protein polyubiquitination // inferred from direct assay///0000209 // protein
NM_0282	0.0261	0.027		1.08	-1.06	-1.06	1.07	***Sgol1***	shugoshin-like 1 (S. pombe)	0007049 // cell cycle // inferred from electronic annotation///0007059 // chromosome s
NM_0011	0.0479			1.03	-1.08	-1.06	-1.12	***Smox***	spermine oxidase	0006598 // polyamine catabolic process // not recorded///0046208 // spermine cataboli
NM_013774///XM_00651			0.036	1.05	1.01	-1.06	-1.05	***Tcl1b4***	T cell leukemia/lymphoma 1B, 4	
NM_030262///XR_380457			0.046	-1.13	-1.12	-1.06	1.07	***Pofut2***	protein O-fucosyltransferase 2	0001707 // mesoderm formation // inferred from mutant phenotype///0005975 // carbo
NM_008474///XM_00652			0.036	-1.06	-1.06	-1.06	-1.32	***Krt84***	keratin 84	0045616 // regulation of keratinocyte differentiation // inferred from direct assay
NM_011723///XM_		#####		1.18	1.03	-1.06	1.31	***Xdh///Cwc22***	xanthine dehydrogenase///CWC22 spliceosome-associated protein homolog (S. cerevi	0001933 // negative regulation of protein phosphorylation // not recorded///0001937 //
NM_145926		0.05		1.16	1.13	-1.06	-1.16	***Mgat4b***	mannoside acetylglucosaminyltransferase 4, isoenzyme B	0005975 // carbohydrate metabolic process // inferred from electronic annotation///000
NM_1818	0.0451			-1.01	-1.12	-1.06	1.01	***Hcfc1r1***	host cell factor C1 regulator 1 (XPO1-dependent)	
NM_001038700///		0.032		1.08	1.05	-1.06	1.01	***Fnbp1***	formin binding protein 1	0006897 // endocytosis // inferred from electronic annotation///0007399 // nervous syst
NM_0076	0.0133			1.06	1.29	-1.06	-1.15	***Cdkn2b***	cyclin-dependent kinase inhibitor 2B (p15, inhibits CDK4)	0000079 // regulation of cyclin-dependent kinase protein serine/threonine kinase activity // not
NM_001204129///NM_00			0.01	1.03	-1.04	-1.06	-1.09	***C1qtnf1***	C1q and tumor necrosis factor related protein 1	0007204 // positive regulation of cytosolic calcium ion concentration // not recorded///0
NM_0087	0.0395		0.007	1.04	-1.08	-1.06	-1.18	***Dusp8***	dual specificity phosphatase 8	0000188 // inactivation of MAPK activity // inferred from electronic annotation///00064
NM_133351///XM_		0.017		1.14	1.15	-1.06	1.19	***Prss8***	protease, serine 8 (prostasin)	0001942 // hair follicle development // inferred from mutant phenotype///0006508 // pr
NM_023377			0.008	-1.02	1.10	-1.06	1.12	***Stard5***	StAR-related lipid transfer (START) domain containing 5	0006810 // transport // inferred from electronic annotation///0006869 // lipid transport
NM_022411///XM_00653			0.036	1.03	1.02	-1.06	-1.15	***Slc13a2***	solute carrier family 13 (sodium-dependent dicarboxylate transporter), member 2	0006810 // transport // inferred from electronic annotation///0006811 // ion transport /
NM_1727	0.0305	0.007		1.22	1.10	-1.06	1.05	***Leng8***	leukocyte receptor cluster (LRC) member 8	
NM_1393	0.0409	0.048		1.11	-1.02	-1.06	-1.02	***Mllt6***	myeloid/lymphoid or mixed-lineage leukemia (trithorax homolog, Drosophila); translocated to, 6	
NM_0166	0.0303	0.013		1.11	-1.03	-1.06	-1.04	***Hpcal1***	hippocalcin-like 1	0007165 // signal transduction // inferred from electronic annotation
NM_0075	0.0125	0.022		1.06	-1.05	-1.06	-1.03	***Caml***	calcium modulating ligand	0001881 // receptor recycling // inferred from mutant phenotype///0007173 // epiderma
NM_0013	0.0126			1.11	-1.08	-1.06	-1.16	***Fdx1***	ferredoxin 1	0006629 // lipid metabolic process // inferred from electronic annotation///0006694 // s
NM_0086	0.0066			1.04	-1.09	-1.06	-1.01	***Mtnr1a***	melatonin receptor 1A	0007165 // signal transduction // inferred from electronic annotation///0007166 // cell s
NM_153807///XR_		0.034		1.09	1.04	-1.06	1.12	***Acsf2***	acyl-CoA synthetase family member 2	0006629 // lipid metabolic process // inferred from electronic annotation///0006631 // f
NM_001159934///NM_14			0.024	1.12	-1.04	-1.06	-1.12	***Oas1h***	2'-5' oligoadenylate synthetase 1H	0006955 // immune response // inferred from electronic annotation///0008152 // metab
NM_133985///NR_045693			0.045	-1.08	-1.15	-1.06	1.12	***Oxsr1***	oxidative-stress responsive 1	0006468 // protein phosphorylation // not recorded///0006979 // response to oxidative
NM_0188	0.008	0.011		1.25	-1.04	-1.06	1.01	***Pnliprp1***	pancreatic lipase related protein 1	0006629 // lipid metabolic process // inferred from electronic annotation
NM_025606		0.013		1.12	1.15	-1.06	1.05	***Mrpl16***	mitochondrial ribosomal protein L16	0006412 // translation // not recorded///0032543 // mitochondrial translation // not rec
NM_133968///XM_		0.017		1.13	1.07	-1.06	-1.02	***Snapc2***	small nuclear RNA activating complex, polypeptide 2	0006351 // transcription, DNA-templated // inferred from electronic annotation///00063
NM_0011	0.0081	0.019		1.06	-1.24	-1.06	1.16	***Lpin2***	lipin 2	0006351 // transcription, DNA-templated // inferred from electronic annotation///00063
NM_1391	0.0056			-1.03	1.21	-1.06	1.05	***Adgre4***	adhesion G protein-coupled receptor E4	0007165 // signal transduction // inferred from electronic annotation///0007166 // cell s
NM_033603			0.04	1.07	1.02	-1.06	-1.13	***Amn***	amnionless	0006898 // receptor-mediated endocytosis // not recorded///0007275 // multicellular or
NM_0255	0.0168			-1.07	1.08	-1.06	-1.13	***Nudt8***	nudix (nucleoside diphosphate linked moiety X)-type motif 8	0008152 // metabolic process // inferred from electronic annotation
NM_0011	0.0066			-1.01	1.29	-1.06	1.09	***Ptk2b***	PTK2 protein tyrosine kinase 2 beta	0000165 // MAPK cascade // not recorded///0000302 // response to reactive oxygen spe
NM_001101502///		0.042		1.12	1.15	-1.06	-1.03	***Zfp703***	zinc finger protein 703	0006351 // transcription, DNA-templated // inferred from electronic annotation///00063
NM_007685			0.036	-1.01	1.09	-1.06	-1.15	***Cfc1***	cripto, FRL-1, cryptic family 1	0001889 // liver development // inferred from mutant phenotype///0001947 // heart loo
NM_001098203///		0.049	0.006	1.20	1.09	-1.06	-1.08	***Hic1***	hypermethylated in cancer 1	0000122 // negative regulation of transcription from RNA polymerase II promoter // infer
NM_0010	0.042			1.20	1.09	-1.06	-1.08	***Hic1***	hypermethylated in cancer 1	0000122 // negative regulation of transcription from RNA polymerase II promoter // infer
NM_009374///XM_00649			0.034	1.06	-1.01	-1.06	-1.13	***Tgm3***	transglutaminase 3, E polypeptide	0008544 // epidermis development // traceable author statement///0018149 // peptide
NM_0108	######	0.003		1.07	-1.09	-1.06	-1.04	***Ncoa1***	nuclear receptor coactivator 1	0000435 // positive regulation of transcription from RNA polymerase II promoter by gala
NM_0010	0.0062			1.03	-1.10	-1.06	-1.10	***Zfp518b***	zinc finger protein 518B	0006351 // transcription, DNA-templated // inferred from electronic annotation///00063
NM_001161741///NM_01			0.01	-1.11	1.04	-1.06	1.06	***Reg3d***	regenerating islet-derived 3 delta	
NM_1813	0.0053			1.02	-1.15	-1.06	-1.15	***Slc25a29***	solute carrier family 25 (mitochondrial carrier, palmitoylcarnitine transporter), membe	0006810 // transport // inferred from electronic annotation///0006844 // acyl carnitine t
NM_027411///XM_		0.028		-1.25	-1.09	-1.06	-1.03	***Spdl1***	spindle apparatus coiled-coil protein 1	0000132 // establishment of mitotic spindle orientation // not recorded///0007049 // cel
NM_001082961///		0.02		1.02	1.04	-1.06	-1.03	***Snrpn///Snurf///Gm35595***	small nuclear ribonucleoprotein N///SNRPN upstream reading frame///predicted gene	0000398 // mRNA splicing, via spliceosome // not recorded///0009725 // response to hor
NM_007405///XM_		0.017		1.06	-1.02	-1.06	1.13	***Adcy6***	adenylate cyclase 6	0003091 // renal water homeostasis // inferred from mutant phenotype///0006171 // cA
NM_0205	0.0234			1.08	1.32	-1.06	-1.05	***Fzd2***	frizzled homolog 2 (Drosophila)	0003149 // membranous septum morphogenesis // inferred from genetic interaction///0
NM_1537	0.0168			1.14	1.40	-1.06	1.03	***Fermt3***	fermitin family homolog 3 (Drosophila)	0007155 // cell adhesion // inferred from electronic annotation///0007159 // leukocyte c
NM_0213	0.0231			-1.12	1.05	-1.06	-1.06	***Vstm2b***	V-set and transmembrane domain containing 2B	
NM_008973///XM_00650			0.027	1.02	1.05	-1.06	-1.27	***Ptn***	pleiotrophin	0001503 // ossification // inferred from direct assay///0001889 // liver development // ir
NM_001024698///		0.026		-1.16	-1.08	-1.05	-1.23	***Cpa2***	carboxypeptidase A2, pancreatic	0006508 // proteolysis // inferred from electronic annotation///0007039 // protein catab
NM_009045///XM_00653			0.018	1.12	1.13	-1.05	1.06	***Rela***	v-rel reticuloendotheliosis viral oncogene homolog A (avian)	0000122 // negative regulation of transcription from RNA polymerase II promoter // not r
NM_023773///XM_		0.043	0.021	1.01	-1.02	-1.05	-1.11	***Mphosph8***	M-phase phosphoprotein 8	0006351 // transcription, DNA-templated // inferred from electronic annotation///00063
NM_0011	0.0146			1.02	-1.24	-1.05	1.04	***Chn2***	chimerin 2	0007165 // signal transduction // inferred from electronic annotation///0035556 // intra
NM_1456	0.0483		0.042	-1.02	-1.10	-1.05	-1.20	***Alox12e***	arachidonate lipoxygenase, epidermal	0006629 // lipid metabolic process // inferred from electronic annotation///0006631 // f

RefSeq Tr	p 24h	p 48h	p 72h	FD Air	FD 24h	FD 48h	FD 72h	Gene Symbol	Gene Title	Gene Ontology Biological Process
NM_0264	0.0471			-1.00	-1.19	-1.05	1.34	***Tppp3***	tubulin polymerization-promoting protein family member 3	0001578 // microtubule bundle formation // not recorded///0046785 // microtubule pol
NM_053249			0.004	1.09	-1.07	-1.05	-1.24	***Krt82***	keratin 82	
NM_0233	0.0394			-1.15	1.15	-1.05	-1.22	***Samsn1***	SAM domain, SH3 domain and nuclear localization signals, 1	0002820 // negative regulation of adaptive immune response // inferred from mutant ph
NM_020279///XM_006517			0.027	1.03	1.01	-1.05	-1.10	***Ccl28***	chemokine (C-C motif) ligand 28	0001954 // positive regulation of cell-matrix adhesion // not recorded///0006935 // cher
NM_001190466///		0.038		1.11	1.11	-1.05	1.04	***Dact1***	dapper homolog 1, antagonist of beta-catenin (xenopus)	0000122 // negative regulation of transcription from RNA polymerase II promoter // not r
NM_1818	0.0391			-1.11	1.02	-1.05	-1.13	***Tpte***	transmembrane phosphatase with tensin homology	0006470 // protein dephosphorylation // inferred from electronic annotation///0016311
NM_029798			0.032	1.07	1.01	-1.05	-1.17	***Flywch2***	FLYWCH family member 2	
NM_130886///XM_00653			0.01	-1.03	-1.02	-1.05	-1.13	***Card14***	caspase recruitment domain family, member 14	0001934 // positive regulation of protein phosphorylation // not recorded///0006915 // a
NM_024479///XM		0.031		1.07	1.05	-1.05	-1.05	***Wbscr27***	Williams Beuren syndrome chromosome region 27 (human)	0008152 // metabolic process // inferred from electronic annotation///0032259 // methy
NM_0013	0.0095			1.05	-1.14	-1.05	-1.09	***Vsx2***	visual system homeobox 2	0006351 // transcription, DNA-templated // inferred from electronic annotation///00063
NM_027203		0.042	0.01	-1.17	1.00	-1.05	-1.03	***Leng1***	leukocyte receptor cluster (LRC) member 1	
NM_001003918///		0.016		1.03	-1.04	-1.05	1.01	***Usp7***	ubiquitin specific peptidase 7	0006281 // DNA repair // inferred from electronic annotation///0006283 // transcription
NM_023312///XM_00650			0.018	1.05	-1.05	-1.05	-1.28	***Ndufa13///Yjefn3***	NADH dehydrogenase (ubiquinone) 1 alpha subcomplex, 13///YjeF N-terminal domain	0006915 // apoptotic process // inferred from electronic annotation///0030308 // negati
NM_001316699///NM_02			0.015	1.04	-1.01	-1.05	-1.19	***Mier2***	mesoderm induction early response 1, family member 2	0006351 // transcription, DNA-templated // inferred from electronic annotation///00063
NM_001163770///		0.048		1.06	1.08	-1.05	1.03	***Dxo***	decapping exoribonuclease	0006402 // mRNA catabolic process // inferred from mutant phenotype///0008152 // me
NM_0081	0.0071			-1.06	-1.57	-1.05	1.22	***Glul***	glutamate-ammonia ligase (glutamine synthetase)	0006536 // glutamate metabolic process // not recorded///0006542 // glutamine biosynt
NM_0082	0.007			1.06	1.51	-1.05	-1.26	***Map4k1***	mitogen-activated protein kinase kinase kinase kinase 1	0000165 // MAPK cascade // not recorded///0000185 // activation of MAPKKK activity //
NM_146240///XM_00651			0.005	-1.15	-1.07	-1.05	1.08	***Rassf9***	Ras association (RalGDS/AF-6) domain family (N-terminal) member 9	0007165 // signal transduction // inferred from electronic annotation///0046907 // intrac
NM_011592		0.046		1.04	1.00	-1.05	1.11	***Timm44***	translocase of inner mitochondrial membrane 44	0006810 // transport // inferred from electronic annotation///0006886 // intracellular pr
NM_0010	0.0271		0.043	1.10	-1.03	-1.05	1.39	***Fgfr1***	fibroblast growth factor receptor 1	0000122 // negative regulation of transcription from RNA polymerase II promoter // infer
NM_0011	0.039			-1.10	1.07	-1.05	-1.14	***Trp63***	transformation related protein 63	0000122 // negative regulation of transcription from RNA polymerase II promoter // infer
NM_007813///XM_00653			0.003	1.07	-1.01	-1.05	-1.13	***Cyp2b13***	cytochrome P450, family 2, subfamily b, polypeptide 13	0006805 // xenobiotic metabolic process // not recorded///0019373 // epoxygenase P45
NM_0011	0.0182			-1.07	-1.39	-1.05	-1.32	***Banp***	BTG3 associated nuclear protein	0006351 // transcription, DNA-templated // inferred from electronic annotation///00063
NM_028055///XM_00653			0.042	-1.02	1.07	-1.05	-1.30	***Btbd17***	BTB (POZ) domain containing 17	
NM_010758///XM_00653			0.026	1.08	1.08	-1.05	-1.07	***Mag***	myelin-associated glycoprotein	0007155 // cell adhesion // inferred from electronic annotation///0007399 // nervous sy
NM_0013	0.0203			-1.01	1.17	-1.05	-1.02	***Grhpr***	glyoxylate reductase/hydroxypyruvate reductase	0007588 // excretion // not recorded///0008152 // metabolic process // inferred from el
NM_1535	0.0012			1.20	-1.12	-1.05	1.20	***Tns2***	tensin 2	0001822 // kidney development // inferred from mutant phenotype///0006470 // protei
NM_0137	0.0112			-1.13	1.19	-1.05	-1.01	***LOC102642963///Rps26***	40S ribosomal protein S26-like///ribosomal protein S26	0006412 // translation // inferred from electronic annotation///0033119 // negative regu
NM_0010	0.0396			1.03	-1.22	-1.05	1.11	***Pdgfra***	platelet derived growth factor receptor, alpha polypeptide	0001553 // luteinization // inferred from genetic interaction///0001553 // luteinization /
NM_0092	0.0226			-1.02	-1.21	-1.05	-1.03	***Snta1***	syntrophin, acidic 1	0002027 // regulation of heart rate // not recorded///0003117 // regulation of vasocons
NM_018780			0.036	-1.00	1.02	-1.05	-1.18	***Sfrp5***	secreted frizzled-related sequence protein 5	0007275 // multicellular organismal development // inferred from electronic annotation/
NM_053178///XM		0.007		-1.13	-1.12	-1.05	-1.12	***Acsbg1***	acyl-CoA synthetase bubblegum family member 1	0000038 // very long-chain fatty acid metabolic process // not recorded///0000038 // ve
NM_1338	0.0072			1.02	-1.17	-1.05	1.10	***Bsdc1***	BSD domain containing 1	
NM_0012	0.0076			1.04	-1.18	-1.05	1.01	***Rora***	RAR-related orphan receptor alpha	0001525 // angiogenesis // not recorded///0006351 // transcription, DNA-templated // i
NM_0013	0.0436			1.07	-1.15	-1.05	1.10	***Trpc1***	transient receptor potential cation channel, subfamily C, member 1	0006810 // transport // inferred from electronic annotation///0006811 // ion transport /
NM_0013	0.0124			-1.03	1.13	-1.05	-1.09	***Car7***	carbonic anhydrase 7	0002230 // positive regulation of defense response to virus by host // not recorded///00
NM_001290789///NM_02			0.032	1.07	-1.03	-1.05	-1.17	***Shroom1***	shroom family member 1	0000902 // cell morphogenesis // inferred from direct assay///0051017 // actin filament
NM_008997///XM		0.022		1.14	1.08	-1.05	1.08	***Rab11b***	RAB11B, member RAS oncogene family	0001881 // receptor recycling // inferred from mutant phenotype///0006810 // transpor
NR_02832	0.0374			1.05	-1.10	-1.05	-1.01	***Appbp2os***	amyloid beta precursor protein (cytoplasmic tail) binding protein 2, opposite strand	
NM_001085348///NM_00			0.014	1.04	-1.07	-1.05	-1.20	***Rhox2a///Rhox2b///Rhox2e***	reproductive homeobox 2A///reproductive homeobox 2B///reproductive homeobox 2	0000122 // negative regulation of transcription from RNA polymerase II promoter // infer
NM_172665///XM		0.037		1.15	1.08	-1.05	-1.01	***Pdk1***	pyruvate dehydrogenase kinase, isoenzyme 1	0005975 // carbohydrate metabolic process // inferred from electronic annotation///000
NM_024264///XM		0.039		1.09	1.01	-1.05	-1.07	***Cyp27a1***	cytochrome P450, family 27, subfamily a, polypeptide 1	0008203 // cholesterol metabolic process // not recorded///0036378 // calcitriol biosynt
NM_016852///XM		0.049		-1.01	1.02	-1.05	1.18	***Wbp2***	WW domain binding protein 2	0045184 // establishment of protein localization // not recorded///0045815 // positive re
NM_0090	0.0066	0.005	0.001	1.00	-1.01	-1.05	1.01	***Rps29***	ribosomal protein S29	0006412 // translation // not recorded
NM_024190///NM_02592			0.033	-1.11	-1.01	-1.05	1.15	***2610002M06Rik///Chmp1b***	RIKEN cDNA 2610002M06 gene///charged multivesicular body protein 1B	0000920 // cell separation after cytokinesis // not recorded///0006810 // transport // inf
NM_0011	0.0487			-1.05	1.07	-1.05	-1.11	***6-Sep***	septin 6	0007049 // cell cycle // inferred from electronic annotation///0051301 // cell division // i
NM_001310766///NM_02			0.004	1.01	-1.06	-1.05	-1.13	***B3gat1***	beta-1,3-glucuronyltransferase 1 (glucuronosyltransferase P)	0005975 // carbohydrate metabolic process // not recorded///0006486 // protein glycos
NM_2072	0.0274			1.06	1.19	-1.05	-1.26	***Mrps26***	mitochondrial ribosomal protein S26	
NM_0257	0.0136			-1.05	1.09	-1.05	-1.08	***Adat2***	adenosine deaminase, tRNA-specific 2	0002100 // tRNA wobble adenosine to inosine editing // inferred from electronic annotat
NM_0010	0.0332			-1.05	1.17	-1.05	-1.08	***Lif***	leukemia inhibitory factor	0001974 // blood vessel remodeling // inferred from genetic interaction///0006955 // im
NM_0012	0.0244			1.05	-1.17	-1.05	1.07	***Birc2***	baculoviral IAP repeat-containing 2	0000209 // protein polyubiquitination // not recorded///0001666 // response to hypoxia
NM_0013	0.0016			1.01	2.38	-1.05	-1.06	***Coro1a***	coronin, actin binding protein 1A	0001845 // phagolysosome assembly // not recorded///0006816 // calcium ion transport
NM_0013	0.0166			-1.00	1.54	-1.05	-1.16	***Prkcb***	protein kinase C, beta	0001666 // response to hypoxia // inferred from direct assay///0002250 // adaptive imm
NM_172676///XM		0.023		1.10	-1.00	-1.05	1.04	***Samd10***	sterile alpha motif domain containing 10	
NM_0010	0.007			1.04	-1.25	-1.05	1.07	***Dock9***	dedicator of cytokinesis 9	0007264 // small GTPase mediated signal transduction // inferred from electronic annota
NM_001039653///NM_01			0.019	1.03	-1.05	-1.05	-1.20	***Lhx3***	LIM homeobox protein 3	0001890 // placenta development // inferred from genetic interaction///0006351 // tran
NM_0106	0.0028			-1.04	-1.70	-1.05	-1.01	***Klf9***	Kruppel-like factor 9	0006351 // transcription, DNA-templated // inferred from electronic annotation///00063
NM_145394			0.025	-1.10	-1.03	-1.05	1.11	***Slc44a3***	solute carrier family 44, member 3	
NM_0012	0.0167			-1.01	1.40	-1.05	-1.10	***Fyb***	FYN binding protein	0006955 // immune response // inferred from electronic annotation///0045576 // mast c
NM_011616			0.033	-1.00	-1.08	-1.05	-1.18	***Cd40lg***	CD40 ligand	0002839 // positive regulation of immune response to tumor cell // not recorded///0006
NM_0010	0.0018	0.011	0.011	1.07	-1.12	-1.05	-1.05	***Cmc4///Mtcp1***	C-x(9)-C motif containing 4///mature T cell proliferation 1	
NM_175667			0.022	1.07	-1.04	-1.05	-1.18	***Ankef1***	ankyrin repeat and EF-hand domain containing 1	
NM_013851///XM		0.038		1.16	1.05	-1.05	-1.08	***Abca8b***	ATP-binding cassette, sub-family A (ABC1), member 8b	0006810 // transport // not recorded///0006869 // lipid transport // not recorded///000
NM_0118	0.0198			-1.03	-1.17	-1.05	-1.04	***Mmp17***	matrix metallopeptidase 17	0006508 // proteolysis // inferred from electronic annotation

RefSeq Tr	*p* 24h	*p* 48h	*p* 72h	FD Air	FD 24h	FD 48h	FD 72h	Gene Symbol	Gene Title	Gene Ontology Biological Process
NM_0094	0.0188			1.03	-1.12	-1.05	1.14	***Tst***	thiosulfate sulfurtransferase, mitochondrial	0008152 // metabolic process // inferred from electronic annotation///0030855 // epithe
NM_0010	0.0478			-1.11	1.04	-1.05	1.05	***11-Sep***	septin 11	0007049 // cell cycle // inferred from electronic annotation///0051291 // protein hetero
NM_0086	0.0104			1.10	-1.08	-1.05	1.16	***Mmp15***	matrix metallopeptidase 15	0006508 // proteolysis // inferred from electronic annotation///0032355 // response to e
NM_007435		0.034		1.10	1.17	-1.04	1.12	***Abcd1***	ATP-binding cassette, sub-family D (ALD), member 1	0006635 // fatty acid beta-oxidation // inferred from mutant phenotype///0006635 // fa
NM_0104	0.0257			-1.06	-1.13	-1.04	-1.04	***Hexa***	hexosaminidase A	0001501 // skeletal system development // inferred from genetic interaction///0005975
NM_1445	0.032			-1.12	1.04	-1.04	-1.07	***Fcgr4***	Fc receptor, IgG, low affinity IV	0001866 // NK T cell proliferation // not recorded///0051930 // regulation of sensory per
NM_0012	0.0301			-1.05	1.07	-1.04	-1.07	***Nppb***	natriuretic peptide type B	0001666 // response to hypoxia // inferred from electronic annotation///0003085 // neg
NM_020289			0.032	1.05	1.00	-1.04	-1.22	***Olfr544***	olfactory receptor 544	0007165 // signal transduction // not recorded///0007186 // G-protein coupled receptor
NM_001285845///		0.018		1.11	1.10	-1.04	1.12	***Paqr7***	progestin and adipoQ receptor family member VII	0007275 // multicellular organismal development // inferred from electronic annotation/
NM_011121		0.032		1.10	1.03	-1.04	1.04	***Plk1***	polo-like kinase 1	0000070 // mitotic sister chromatid segregation // not recorded///0000086 // G2/M tran
NM_0010	0.0031			-1.00	1.13	-1.04	1.11	***Thoc6***	THO complex 6 homolog (Drosophila)	0006397 // mRNA processing // inferred from electronic annotation///0006406 // mRNA
NM_001162485///		0.032		1.05	1.08	-1.04	1.00	***Arrdc1***	arrestin domain containing 1	
NM_0012	0.046		0.022	1.09	1.00	-1.04	-1.06	***Ranbp3***	RAN binding protein 3	0006810 // transport // inferred from electronic annotation///0015031 // protein transp
NM_001146153///NM_01			0.038	1.01	1.03	-1.04	-1.21	***Homer3***	homer homolog 3 (Drosophila)	0007216 // G-protein coupled glutamate receptor signaling pathway // traceable author
NM_027122			0.038	1.03	-1.02	-1.04	-1.19	***Mfsd3***	major facilitator superfamily domain containing 3	0006810 // transport // not recorded///0015992 // proton transport // ---///0055085 //
NM_0101	0.0044		0.012	1.10	-1.04	-1.04	-1.07	***Ext2***	exostoses (multiple) 2	0001503 // ossification // not recorded///0001707 // mesoderm formation // inferred fr
NM_0101	0.0044			-1.09	1.15	-1.04	-1.13	***Fcgr1***	Fc receptor, IgG, high affinity I	0001788 // antibody-dependent cellular cytotoxicity // inferred from mutant phenotype/
NM_0010	0.0105			-1.01	-1.13	-1.04	1.14	***Zfp57***	zinc finger protein 57	0000122 // negative regulation of transcription from RNA polymerase II promoter // infer
NM_144913		0.021		1.10	1.08	-1.04	1.00	***Mepce***	methylphosphate capping enzyme	0000122 // negative regulation of transcription from RNA polymerase II promoter // infer
NM_0011	0.0393			1.05	-1.09	-1.04	-1.03	***Tmem184a***	transmembrane protein 184a	0006810 // transport // ---///0018992 // germ-line sex determination // inferred from mu
NM_145490///XM		0.044		1.14	1.00	-1.04	1.02	***Zfp959***	zinc finger protein 959	0006355 // regulation of transcription, DNA-templated // not recorded
NM_001003815///		0.017		1.11	1.02	-1.04	-1.02	***Epb41l1***	erythrocyte membrane protein band 4.1 like 1	0001558 // regulation of cell growth // inferred from electronic annotation///0007010 //
NM_172439///XM_00651			0.024	1.04	-1.02	-1.04	-1.04	***Inpp5j***	inositol polyphosphate 5-phosphatase J	0006470 // protein dephosphorylation // inferred from direct assay///0006470 // protein
NM_0095	0.027	0.034		1.06	-1.04	-1.04	1.03	***Vpreb3***	pre-B lymphocyte gene 3	0002377 // immunoglobulin production // ---///0006955 // immune response // ---///00
NM_028071		#####		1.04	1.12	-1.04	1.04	***Cotl1***	coactosin-like 1 (Dictyostelium)	0019370 // leukotriene biosynthetic process // inferred from electronic annotation///00
NM_012042///XM_00650			0.022	-1.23	1.03	-1.04	1.16	***Cul1***	cullin 1	0006511 // ubiquitin-dependent protein catabolic process // inferred by curator///0006
NM_1752	0.0034			1.11	-1.21	-1.04	1.00	***Arid2***	AT rich interactive domain 2 (ARID, RFX-like)	0006337 // nucleosome disassembly // not recorded///0006355 // regulation of transcri
NM_0296	0.0235			1.04	1.17	-1.04	1.10	***Plet1***	placenta expressed transcript 1	0001953 // negative regulation of cell-matrix adhesion // inferred from mutant phenotyp
NM_0012	0.0169			-1.05	1.38	-1.04	1.06	***Irx3***	Iroquois related homeobox 3	0001656 // metanephros development // inferred from expression pattern///0001822 //
NM_011910			0.025	1.09	-1.10	-1.04	-1.20	***Uts2***	urotensin 2	0001666 // response to hypoxia // inferred from electronic annotation///0003105 // neg
NM_001171024///NM_01			0.036	1.04	1.05	-1.04	-1.21	***Gm21948///Il4i1***	predicted gene, 21948///interleukin 4 induced 1	0006351 // transcription, DNA-templated // ---///0006351 // transcription, DNA-templat
NM_013581///XM_00653			0.005	1.03	1.05	-1.04	-1.06	***Cog1///LOC102641618***	component of oligomeric golgi complex 1///conserved oligomeric Golgi complex subur	0000301 // retrograde transport, vesicle recycling within Golgi // not recorded///00068
NM_018730///XM_00652			0.027	1.04	1.16	-1.04	-1.26	***Rpl36///Rpl36-ps2///Gm4604***	ribosomal protein L36///ribosomal protein L36, pseudogene 2///predicted gene 4604	0002181 // cytoplasmic translation // ---///0006412 // translation // inferred from electr
NM_1530	0.043			-1.09	1.03	-1.04	-1.14	***Tmem5***	transmembrane protein 5	0006486 // protein glycosylation // inferred from electronic annotation///0035269 // pro
NM_001287514///		0.001		1.08	1.02	-1.04	1.05	***Cebpa***	CCAAT/enhancer binding protein (C/EBP), alpha	0000050 // urea cycle // inferred from direct assay///0000122 // negative regulation of t
NM_0011	0.0063			1.18	1.05	-1.04	-1.02	***Zfp574***	zinc finger protein 574	0006351 // transcription, DNA-templated // inferred from electronic annotation///00063
NM_0213	0.0147	0.047		-1.17	1.02	-1.04	-1.04	***Cd200r1***	CD200 receptor 1	
NM_015803///XM_00651			0.017	1.05	-1.07	-1.04	-1.27	***Atp8a2***	ATPase, aminophospholipid transporter-like, class I, type 8A, member 2	0003011 // involuntary skeletal muscle contraction // inferred from mutant phenotype//
NM_001031667///		0.016		1.06	1.04	-1.04	-1.03	***Gsk3a***	glycogen synthase kinase 3 alpha	0003073 // regulation of systemic arterial blood pressure // inferred from mutant phenot
NM_144829///XM_00653			0.019	-1.04	1.03	-1.04	1.12	***Aarsd1***	alanyl-tRNA synthetase domain containing 1	0006412 // translation // inferred from electronic annotation///0006419 // alanyl-tRNA a
NM_0294	0.0298	0.03	0.01	1.09	-1.06	-1.04	-1.18	***Klf17***	Kruppel-like factor 17	0000122 // negative regulation of transcription from RNA polymerase II promoter // infer
NM_028636///XM_00651			0.014	1.10	1.00	-1.04	-1.01	***Man2c1***	mannosidase, alpha, class 2C, member 1	0005975 // carbohydrate metabolic process // inferred from electronic annotation///000
NM_0232	0.0477			1.17	1.06	-1.04	1.07	***Fastk***	Fas-activated serine/threonine kinase	0006468 // protein phosphorylation // not recorded///0006915 // apoptotic process // in
NM_153053			0.045	1.04	1.06	-1.04	-1.14	***Sf3b4***	splicing factor 3b, subunit 4	0006397 // mRNA processing // inferred from electronic annotation///0008380 // RNA sp
NM_0085	0.0418		0.048	1.14	1.49	-1.04	-1.20	***Blnk***	B cell linker	0006955 // immune response // not recorded///0007169 // transmembrane receptor pro
NM_0011	0.0164			1.03	1.30	-1.04	-1.08	***Fcrla***	Fc receptor-like A	0030154 // cell differentiation // inferred from electronic annotation
NM_0011	0.0141			-1.01	1.72	-1.04	-1.05	***Sell***	selectin, lymphocyte	0007155 // cell adhesion // inferred from electronic annotation///0033198 // response t
NM_173382			0.034	-1.01	1.02	-1.04	-1.22	***Proser1***	proline and serine rich 1	
NM_0010	0.0167			1.07	-1.17	-1.04	-1.07	***Unc13b***	unc-13 homolog B (C. elegans)	0006887 // exocytosis // inferred from electronic annotation///0007165 // signal transdu
NM_001290508///		0.027		1.07	1.09	-1.04	-1.03	***Dolpp1***	dolichyl pyrophosphate phosphatase 1	0006486 // protein glycosylation // inferred from electronic annotation///0006487 // pro
NM_153570			0.007	1.08	1.07	-1.04	-1.08	***Noc4l***	nucleolar complex associated 4 homolog (S. cerevisiae)	0042254 // ribosome biogenesis // inferred from electronic annotation
NM_026849///XM_00650			0.042	1.09	-1.05	-1.04	-1.13	***Mtmr14***	myotubularin related protein 14	0006470 // protein dephosphorylation // ---///0006470 // protein dephosphorylation // i
NM_001045520///XM_00			0.037	-1.11	-1.02	-1.04	1.13	***Clint1***	clathrin interactor 1	0006897 // endocytosis // inferred from electronic annotation///0016192 // vesicle-medi
NM_001310587///		0.006	0.033	1.10	1.04	-1.04	-1.05	***Mef2d***	myocyte enhancer factor 2D	0001649 // osteoblast differentiation // inferred from genetic interaction///0001958 // e
NM_0011	0.0412			-1.09	-1.03	-1.04	-1.05	***Gtse1***	G two S phase expressed protein 1	
NM_001243049///		0.042		1.16	1.21	-1.04	1.19	***Atp6v0a1***	ATPase, H+ transporting, lysosomal V0 subunit A1	0006810 // transport // inferred from electronic annotation///0006811 // ion transport /
NM_001307989///NM_01			0.042	1.01	1.03	-1.04	-1.19	***Nr4a3***	nuclear receptor subfamily 4, group A, member 3	0000122 // negative regulation of transcription from RNA polymerase II promoter // infer
NM_0118	0.0203			-1.05	1.11	-1.04	1.01	***Med24***	mediator complex subunit 24	0006351 // transcription, DNA-templated // inferred from electronic annotation///00063
NM_0013	0.0053		0.01	1.05	-1.12	-1.04	-1.12	***Arx***	aristaless related homeobox	0000122 // negative regulation of transcription from RNA polymerase II promoter // infer
NM_0085	######			1.06	-1.50	-1.04	1.04	***Mertk***	c-mer proto-oncogene tyrosine kinase	0001779 // natural killer cell differentiation // inferred from genetic interaction///00064
NM_033523///XM		0.023		1.05	-1.01	-1.04	1.08	***Spred2***	sprouty-related, EVH1 domain containing 2	0000188 // inactivation of MAPK activity // inferred from mutant phenotype///0007275
NM_001081277///XM_00			0.028	1.12	1.09	-1.04	-1.05	***Ak5***	adenylate kinase 5	0006139 // nucleobase-containing compound metabolic process // inferred from electro
NM_0806	0.023			1.00	-1.28	-1.04	-1.01	***Caskin2***	CASK-interacting protein 2	
NM_001205396///NM_05			0.016	1.06	1.07	-1.04	-1.20	***Gprc5d***	G protein-coupled receptor, family C, group 5, member D	0007165 // signal transduction // inferred from electronic annotation///0007186 // G-pr

RefSeq Tra	*p* 24h	*p* 48h	*p* 72h	FD Air	FD 24h	FD 48h	FD 72h	Gene Symbol	Gene Title	Gene Ontology Biological Process
NM_1832	0.0232			-1.01	-1.12	-1.04	-1.05	***Rnmtl1***	RNA methyltransferase like 1	0001510 // RNA methylation // not recorded///0006364 // rRNA processing // inferred fr
NM_0013	0.0052			1.08	1.53	-1.04	-1.16	***Ccr7***	chemokine (C-C motif) receptor 7	0001768 // establishment of T cell polarity // traceable author statement///0002408 // n
NM_0011	0.0189			1.01	1.15	-1.04	-1.02	***Ccm2***	cerebral cavernous malformation 2	0001568 // blood vessel development // inferred from mutant phenotype///0001570 // v
NM_011790///XM_		0.019		1.07	1.14	-1.04	1.01	***Arih2***	ariadne homolog 2 (Drosophila)	0000209 // protein polyubiquitination // not recorded///0006511 // ubiquitin-dependen
NM_0094	0.0447			1.02	-1.18	-1.04	-1.28	***Tpst2***	protein-tyrosine sulfotransferase 2	0006478 // peptidyl-tyrosine sulfation // inferred from mutant phenotype///0007342 // f
NM_145374///XM_		0.007		1.05	1.07	-1.04	1.05	***Mios***	missing oocyte, meiosis regulator, homolog (Drosophila)	0032008 // positive regulation of TOR signaling // not recorded///0034198 // cellular res
NM_001037801///NM_00			#####	1.09	1.03	-1.04	-1.12	***Cd6***	CD6 antigen	0001771 // immunological synapse formation // inferred from sequence or structural sim
NM_001289442///NM_01			0.021	1.01	1.07	-1.04	-1.13	***Grap2***	GRB2-related adaptor protein 2	
NM_0011	0.006			-1.30	1.14	-1.04	-1.04	***Apoc1***	apolipoprotein C-I	0006641 // triglyceride metabolic process // inferred from mutant phenotype///0006810
NM_2076	0.0124			-1.00	-1.44	-1.04	1.02	***Fndc3a***	fibronectin type III domain containing 3A	0007286 // spermatid development // inferred from mutant phenotype///0009566 // fer
NM_018730///XM_00652			0.037	1.06	1.16	-1.04	-1.23	***Rpl36///Rpl36-ps2///Gm4604***	ribosomal protein L36///ribosomal protein L36, pseudogene 2///predicted gene 4604	0002181 // cytoplasmic translation // ---///0006412 // translation // inferred from electro
NM_0011	0.0098			1.06	-1.05	-1.04	1.10	***Kat7***	K(lysine) acetyltransferase 7	0006260 // DNA replication // not recorded///0006351 // transcription, DNA-templated /
NM_175275///NM_17738			0.044	1.13	-1.03	-1.04	-1.15	***Cntln***	centlein, centrosomal protein	0010457 // centriole-centriole cohesion // not recorded///0033365 // protein localization
NM_0012	0.0107			1.09	-1.06	-1.03	1.01	***Osbpl6***	oxysterol binding protein-like 6	0006810 // transport // inferred from electronic annotation///0006869 // lipid transport
NM_0011	0.037			1.12	1.35	-1.03	1.10	***Fcrl1***	Fc receptor-like 1	
NM_0012	0.0489			-1.03	1.19	-1.03	-1.19	***Cmtm7***	CKLF-like MARVEL transmembrane domain containing 7	0002337 // B-1a B cell differentiation // inferred from mutant phenotype///0006935 // c
NM_0110	0.0491			-1.07	1.10	-1.03	-1.15	***Pitx1***	paired-like homeodomain transcription factor 1	0001501 // skeletal system development // inferred from mutant phenotype///0006351 /
NM_1789	0.0354			-1.10	-1.01	-1.03	-1.09	***Hic2***	hypermethylated in cancer 2	0006351 // transcription, DNA-templated // inferred from electronic annotation///00063
NM_0289	0.0397		0.03	1.02	-1.05	-1.03	-1.08	***Gorasp1***	golgi reassembly stacking protein 1	0006487 // protein N-linked glycosylation // inferred from mutant phenotype///0006810
NM_0254	0.0406			1.08	-1.08	-1.03	-1.03	***1700018B24Rik***	enhancer of rudimentary homolog pseudogene	0006221 // pyrimidine nucleotide biosynthetic process // inferred from electronic annota
NM_0010	0.0364			-1.20	-1.60	-1.03	-1.09	***Gprasp1***	G protein-coupled receptor associated sorting protein 1	0008333 // endosome to lysosome transport // not recorded///1990172 // G-protein cou
NM_0076	0.0065			1.08	2.18	-1.03	-1.15	***Ms4a1***	membrane-spanning 4-domains, subfamily A, member 1	0042113 // B cell activation // inferred from electronic annotation
NM_0012	0.002			-1.00	-1.26	-1.03	1.14	***Cav1***	caveolin 1, caveolae protein	0000122 // negative regulation of transcription from RNA polymerase II promoter // infer
NM_0080	0.0457			1.10	-1.01	-1.03	1.03	***Fzd8***	frizzled homolog 8 (Drosophila)	0000122 // negative regulation of transcription from RNA polymerase II promoter // infer
NM_0010	0.0342			1.16	-1.12	-1.03	1.09	***Zscan26***	zinc finger and SCAN domain containing 26	0006351 // transcription, DNA-templated // inferred from electronic annotation///00063
NM_0011	0.0457		0.011	-1.15	-1.02	-1.03	1.00	***Zfp937***	zinc finger protein 937	
NM_0198	0.0396			-1.07	1.05	-1.03	-1.13	***Abcb9***	ATP-binding cassette, sub-family B (MDR/TAP), member 9	0006810 // transport // inferred from electronic annotation///0008152 // metabolic proc
NM_0281	0.033			-1.06	1.09	-1.03	-1.03	***Cep89***	centrosomal protein 89	0030030 // cell projection organization // inferred from electronic annotation///0035058
NM_0011	0.0477			-1.33	-1.83	-1.03	-1.41	***Cdkn1a***	cyclin-dependent kinase inhibitor 1A (P21)	0000079 // regulation of cyclin-dependent protein serine/threonine kinase activity // infe
NM_0012	0.0167			-1.05	-1.41	-1.03	-1.20	***Jdp2***	Jun dimerization protein 2	0000122 // negative regulation of transcription from RNA polymerase II promoter // infer
NM_1810	0.0142			1.08	-1.09	-1.03	1.05	***Adgrl1***	adhesion G protein-coupled receptor L1	0007157 // heterophilic cell-cell adhesion via plasma membrane cell adhesion molecules
NM_001168652///		0.05	0.033	1.10	1.06	-1.03	-1.01	***Gm20605///Lrch4///Sap25***	predicted gene 20605///leucine-rich repeats and calponin homology (CH) domain con	0006351 // transcription, DNA-templated // inferred from electronic annotation///00063
NM_013581///XM_00653			0.022	1.02	1.06	-1.03	-1.05	***Cog1///LOC102641618***	component of oligomeric golgi complex 1///conserved oligomeric Golgi complex subu	0000301 // retrograde transport, vesicle recycling within Golgi // not recorded///000681
XM_357640///XM_		0.047	0.025	1.11	1.16	-1.03	-1.05	***Igk-V21///Igk-V28///Igk-V8///Igkc///Igkv***	immunoglobulin kappa chain variable 21 (V21)///immunoglobulin kappa chain variabl	0006898 // receptor-mediated endocytosis // not recorded///0006910 // phagocytosis, r
NM_001163633///NM_00			0.009	1.10	1.03	-1.03	-1.10	***Wnt7b***	wingless-type MMTV integration site family, member 7B	0001525 // angiogenesis // inferred from genetic interaction///0001525 // angiogenesis /
NM_0011	0.0042			1.07	-1.10	-1.03	-1.08	***Zfp746***	zinc finger protein 746	0000122 // negative regulation of transcription from RNA polymerase II promoter // infer
NM_053126			0.006	-1.08	-1.04	-1.03	1.01	***Pcdhb1***	protocadherin beta 1	0007155 // cell adhesion // inferred from electronic annotation///0007156 // homophilic
NM_001291235///		0.002		1.15	1.01	-1.03	-1.04	***Tlcd1***	TLC domain containing 1	
NM_026645///XM_01124			0.017	-1.10	-1.08	-1.03	-1.04	***Iqcf3***	IQ motif containing F3	
NM_0109	0.0118			1.02	1.11	-1.03	-1.09	***Ntn3///Tbc1d24***	netrin 3///TBC1 domain family, member 24	0006355 // regulation of transcription, DNA-templated // not recorded///0007409 // axo
NM_001081171///XM_00			0.045	1.04	1.05	-1.03	-1.07	***Lama5***	laminin, alpha 5	0001658 // branching involved in ureteric bud morphogenesis // inferred from mutant ph
NM_0079	0.0103			-1.10	1.12	-1.03	-1.14	***Fscn1***	fascin homolog 1, actin bundling protein (Strongylocentrotus purpuratus)	0007015 // actin filament organization // inferred from electronic annotation///0007043
NM_001145881///		0.026		1.11	1.05	-1.03	1.10	***Zfp212***	Zinc finger protein 212	0006355 // regulation of transcription, DNA-templated // inferred from electronic annota
NM_001029850///NM_00			0.037	1.06	-1.01	-1.03	-1.12	***Magi1***	membrane associated guanylate kinase, WW and PDZ domain containing 1	0070997 // neuron death // not recorded
NM_0011	0.0278			1.06	-1.11	-1.03	-1.02	***Mrps2***	mitochondrial ribosomal protein S2	0002230 // positive regulation of defense response to virus by host // not recorded///000
NM_0205	0.0057			1.06	-1.07	-1.03	-1.02	***Tas2r108***	taste receptor, type 2, member 108	0001580 // detection of chemical stimulus involved in sensory perception of bitter taste /
NM_1448	0.0261	0.012	0.04	1.16	-1.09	-1.03	-1.06	***Nrbp2***	nuclear receptor binding protein 2	0006468 // protein phosphorylation // inferred from electronic annotation///0007399 //
NM_001290437///		0.04	0.043	1.07	-1.01	-1.03	-1.03	***Inpp5e***	inositol polyphosphate-5-phosphatase E	0006470 // protein dephosphorylation // inferred from direct assay///0006470 // protein
NM_145425///XM_00651			0.03	-1.16	-1.05	-1.03	1.14	***Wdpcp***	WD repeat containing planar cell polarity effector	0001822 // kidney development // inferred from mutant phenotype///0002093 // audito
NM_001243762///NM_01			0.04	-1.02	-1.02	-1.03	-1.12	***Clcn5***	chloride channel 5	0006810 // transport // inferred from electronic annotation///0006811 // ion transport /
NM_008916///XM_		0.006		1.16	1.14	-1.03	1.15	***Inpp5k***	inositol polyphosphate 5-phosphatase K	0001701 // in utero embryonic development // inferred from mutant phenotype///0001
NM_0105	0.0031	0.041		-1.17	1.20	-1.03	-1.20	***Il1r2***	interleukin 1 receptor, type II	0019221 // cytokine-mediated signaling pathway // inferred from electronic annotation
NM_026490			0.013	-1.18	-1.03	-1.03	1.10	***Mrpl19***	mitochondrial ribosomal protein L19	0006412 // translation // inferred from sequence or structural similarity
NM_0114	0.0291			-1.08	-1.38	-1.03	1.17	***Serpinb6b***	serine (or cysteine) peptidase inhibitor, clade B, member 6b	0008406 // gonad development // non-traceable author statement///0010951 // negativ
NM_0137	0.0084			1.11	1.41	-1.03	-1.05	***Dok3***	docking protein 3	0007265 // Ras protein signal transduction // inferred from direct assay
NM_0012	0.0029	0.008		1.21	-1.01	-1.03	1.02	***Bcl2l11***	BCL2-like 11 (apoptosis facilitator)	0001701 // in utero embryonic development // inferred from genetic interaction///0001
NM_009697///NM		0.033		1.15	1.05	-1.03	1.06	***Nr2f2***	nuclear receptor subfamily 2, group F, member 2	0000122 // negative regulation of transcription from RNA polymerase II promoter // infer
NM_0194	0.016			-1.15	1.11	-1.03	1.01	***Paf1***	Paf1, RNA polymerase II associated factor, homolog (S. cerevisiae)	0000122 // negative regulation of transcription from RNA polymerase II promoter // infer
NM_0113	0.0078			1.08	-1.13	-1.03	1.00	***Slc22a5***	solute carrier family 22 (organic cation transporter), member 5	0006810 // transport // inferred from electronic annotation///0006811 // ion transport /
NM_0011	0.0178			1.03	-1.04	-1.03	-1.11	***Igfbp7***	insulin-like growth factor binding protein 7	0001558 // regulation of cell growth // inferred from electronic annotation///0007155 //
NM_0080	0.0184			-1.05	1.11	-1.03	1.07	***Ftl1///Ftl2-ps///LOC100862446///Mir692-***	ferritin light polypeptide 1///ferritin light polypeptide 2, pseudogene///ferritin light ch	0006826 // iron ion transport // inferred from electronic annotation///0006879 // cellula
NM_0082	0.0159			-1.13	1.02	-1.03	-1.15	***Foxa3***	forkhead box A3	0001678 // cellular glucose homeostasis // inferred from mutant phenotype///0006351 /
NM_008468///XM_00650			0.011	-1.05	-1.02	-1.03	1.07	***Kpna6***	karyopherin (importin) alpha 6	0006606 // protein import into nucleus // inferred from direct assay///0006607 // NLS-b
NM_0011	######			-1.02	-1.63	-1.03	1.09	***Plod2***	procollagen lysine, 2-oxoglutarate 5-dioxygenase 2	0001666 // response to hypoxia // inferred from electronic annotation///0032870 // cellu

RefSeq Tr	p 24h	p 48h	p 72h	FD Air	FD 24h	FD 48h	FD 72h	Gene Symbol	Gene Title	Gene Ontology Biological Process
NM_001113470///		0.025		1.08	1.16	-1.03	1.04	*Ctdsp2*	CTD (carboxy-terminal domain, RNA polymerase II, polypeptide A) small phosphatase	0001933 // negative regulation of protein phosphorylation // inferred from direct assay//
NM_0137	0.0072			-1.01	1.16	-1.03	-1.10	*Txnrd2*	thioredoxin reductase 2	0000305 // response to oxygen radical // traceable author statement///0007507 // heart
NM_0011	0.0167	0.03	0.008	1.24	1.05	-1.03	-1.10	*Slc16a11*	solute carrier family 16 (monocarboxylic acid transporters), member 11	0006629 // lipid metabolic process // not recorded///0006810 // transport // inferred fro
NM_001164062///		0.019		1.14	1.20	-1.03	1.10	*Stat5a*	signal transducer and activator of transcription 5A	0000255 // allantoin metabolic process // inferred from mutant phenotype///0001553 //
NM_0231	######			-1.18	1.22	-1.03	-1.03	*Arpc1b*	actin related protein 2/3 complex, subunit 1B	0030833 // regulation of actin filament polymerization // inferred from electronic annota
NM_139236///NM		0.004		1.08	1.05	-1.03	-1.01	*Nol6*	nucleolar protein family 6 (RNA-associated)	0006364 // rRNA processing // not recorded///0006409 // tRNA export from nucleus // n
NM_0532	0.0486			1.01	-1.05	-1.03	-1.02	*Erc1*	ELKS/RAB6-interacting/CAST family member 1	0006810 // transport // inferred from electronic annotation///0007252 // I-kappaB phos
NM_025476///XR_		0.007		1.27	1.15	-1.03	1.08	*Rmdn1*	regulator of microtubule dynamics 1	
NM_0090	0.0496			1.08	-1.10	-1.03	-1.03	*Rel*	reticuloendotheliosis oncogene	0000122 // negative regulation of transcription from RNA polymerase II promoter // not
NM_0236	0.0143			-1.00	1.13	-1.03	-1.04	*Sars2*	seryl-aminoacyl-tRNA synthetase 2	0006412 // translation // inferred from electronic annotation///0006418 // tRNA aminoa
NM_0080	0.0301			1.13	-1.01	-1.03	1.15	*Fzd7*	frizzled homolog 7 (Drosophila)	0006355 // regulation of transcription, DNA-templated // not recorded///0007165 // sigr
NM_001301100///NM_18			0.003	1.15	1.04	-1.03	-1.06	*Edc4*	enhancer of mRNA decapping 4	
NM_0011	0.0099			-1.03	-1.17	-1.02	1.24	*Cep55*	centrosomal protein 55	0000281 // mitotic cytokinesis // not recorded///0000920 // cell separation after cytokin
NM_001290740///		0.031		1.28	1.07	-1.02	1.24	*Cwc22///Xdh*	CWC22 spliceosome-associated protein homolog (S. cerevisiae)///xanthine dehydroge	0000398 // mRNA splicing, via spliceosome // not recorded///0001933 // negative regula
NM_013905			0.005	1.12	1.05	-1.02	1.24	*Heyl*	hairy/enhancer-of-split related with YRPW motif-like	0003151 // outflow tract morphogenesis // inferred from genetic interaction///0003181
NM_0085	0.0441		#####	1.04	-1.08	-1.02	-1.17	*Lor*	loricrin	0031424 // keratinization // inferred from electronic annotation
NM_0012	0.0201			1.03	-1.06	-1.02	-1.11	*Ngb*	neuroglobin	0006810 // transport // inferred from electronic annotation///0006915 // apoptotic proc
NM_0012	0.0031			-1.02	-1.13	-1.02	1.00	*Ncor1*	nuclear receptor co-repressor 1	0000122 // negative regulation of transcription from RNA polymerase II promoter // infer
NM_1786	0.0059			1.08	-1.10	-1.02	1.07	*Poldip3*	polymerase (DNA-directed), delta interacting protein 3	0006417 // regulation of translation // inferred from electronic annotation///0006810 //
NM_1386	0.0021			1.04	-1.03	-1.02	1.16	*Casc3*	cancer susceptibility candidate 3	0000184 // nuclear-transcribed mRNA catabolic process, nonsense-mediated decay // inf
NM_199467///XM_00653			0.043	-1.04	1.06	-1.02	1.18	*Mms22l*	MMS22-like, DNA repair protein	0000724 // double-strand break repair via homologous recombination // not recorded//
NM_0010	0.0459			1.17	-1.02	-1.02	-1.16	*Cracr2b*	calcium release activated channel regulator 2B	0002115 // store-operated calcium entry // not recorded///0034613 // cellular protein lo
NM_0010	0.0343			-1.07	1.12	-1.02	1.05	*Spryd3*	SPRY domain containing 3	
NM_011505///XM		0.025	0.022	-1.11	-1.14	-1.02	-1.04	*Stxbp4*	syntaxin binding protein 4	0006605 // protein targeting // inferred from direct assay///0006974 // cellular response
NM_0223	0.0266		0.008	1.12	-1.03	-1.02	-1.19	*Slc28a3*	solute carrier family 28 (sodium-coupled nucleoside transporter), member 3	0001895 // retina homeostasis // not recorded///0006810 // transport // inferred from e
NM_053185///XM_011247			0.009	-1.01	-1.05	-1.02	1.10	*Col4a6*	collagen, type IV, alpha 6	0030198 // extracellular matrix organization // inferred from direct assay///0071230 // c
NM_0117	0.0376			-1.03	-1.20	-1.02	1.02	*Ywhah*	tyrosine 3-monooxygenase/tryptophan 5-monooxygenase activation protein, eta poly	0002028 // regulation of sodium ion transport // not recorded///0002028 // regulation o
NM_0010	0.0283			1.02	-1.09	-1.02	-1.05	*Purg*	purine-rich element binding protein G	
NM_009160			0.014	-1.04	-1.03	-1.02	1.13	*Sftpd*	surfactant associated protein D	0002376 // immune system process // inferred from electronic annotation///0007585 //
NM_0012	0.0014			1.18	-1.25	-1.02	1.30	*Alas1*	aminolevulinic acid synthase 1	0006778 // porphyrin-containing compound metabolic process // inferred from electroni
NM_011359		0.02		-1.04	-1.05	-1.02	-1.03	*Sftpc*	surfactant associated protein C	0007585 // respiratory gaseous exchange // inferred from electronic annotation///00076
NM_001252448///NM_00			0.045	-1.03	1.03	-1.02	1.07	*Strada*	STE20-related kinase adaptor alpha	0006468 // protein phosphorylation // inferred from electronic annotation///0006611 //
NM_1387	0.0453			1.11	-1.10	-1.02	1.23	*Sdpr*	serum deprivation response	0045944 // positive regulation of transcription from RNA polymerase II promoter // not r
NM_139236///NM_13923			0.022	1.08	1.08	-1.02	-1.01	*Nol6*	nucleolar protein family 6 (RNA-associated)	0006364 // rRNA processing // not recorded///0006409 // tRNA export from nucleus // n
NM_0199	0.0434			1.03	-1.11	-1.02	-1.03	*Alg2*	asparagine-linked glycosylation 2 (alpha-1,3-mannosyltransferase)	0006486 // protein glycosylation // inferred from electronic annotation///0006488 // dol
NM_001078649///		0.049		1.09	1.14	-1.02	-1.06	*Tmem134*	transmembrane protein 134	
NM_001311156///		0.025		1.09	1.10	-1.02	1.05	*Tcta*	T cell leukemia translocation altered gene	
NM_0114	0.0239			-1.14	1.47	-1.02	-1.09	*Slfn1*	schlafen 1	0000122 // negative regulation of transcription from RNA polymerase II promoter // infer
NM_0090	0.0028			-1.10	1.80	-1.02	1.04	*Rac2*	RAS-related C3 botulinum substrate 2	0006935 // chemotaxis // inferred from direct assay///0007015 // actin filament organiz
NM_0187	0.0387			-1.05	1.10	-1.02	-1.08	*Nme6*	NME/NM23 nucleoside diphosphate kinase 6	0006165 // nucleoside diphosphate phosphorylation // inferred from electronic annotati
NM_1531	0.0348			-1.04	1.18	-1.02	-1.02	*Phospho1///Zfp652*	phosphatase, orphan 1///zinc finger protein 652	0001958 // endochondral ossification // inferred from mutant phenotype///0006351 // t
NM_0312	0.0199			1.04	1.35	-1.02	1.05	*Gimap3*	GTPase, IMAP family member 3	0033955 // mitochondrial DNA inheritance // inferred from mutant phenotype
NM_001291047///NM_00			0.047	1.03	1.01	-1.02	-1.08	*Ypel1*	yippee-like 1 (Drosophila)	
NM_1990	0.0184			1.14	-1.14	-1.02	1.00	*Stard8*	START domain containing 8	0007165 // signal transduction // inferred from electronic annotation///0043547 // posit
NM_008581		0.025		1.15	1.02	-1.02	1.04	*Mela*	melanoma antigen	0006508 // proteolysis // inferred from electronic annotation///0015074 // DNA integrat
NM_0010	0.0038	0.013	0.043	1.10	-1.04	-1.02	1.03	*Cdc42bpa*	CDC42 binding protein kinase alpha	0000226 // microtubule cytoskeleton organization // inferred from mutant phenotype///
NM_0280	0.0143			1.02	1.18	-1.02	-1.22	*Ddx54*	DEAD (Asp-Glu-Ala-Asp) box polypeptide 54	0006351 // transcription, DNA-templated // inferred from electronic annotation///00063
NM_0272	0.0361		0.012	1.10	-1.03	-1.02	-1.16	*Ppp1r35*	protein phosphatase 1, regulatory subunit 35	0010923 // negative regulation of phosphatase activity // not recorded
NM_0335	0.0084			1.08	-1.11	-1.02	1.04	*Aff4*	AF4/FMR2 family, member 4	0006351 // transcription, DNA-templated // inferred from electronic annotation///00063
NM_017401///XM_00651			0.039	1.14	1.17	-1.02	-1.05	*Polm*	polymerase (DNA directed), mu	0006281 // DNA repair // inferred from electronic annotation///0006310 // DNA recomb
NM_0231	0.0402			1.05	1.14	-1.02	-1.16	*Map2k2*	mitogen-activated protein kinase kinase 2	0000165 // MAPK cascade // not recorded///0000187 // activation of MAPK activity // tr
NM_019435///XM_00652			0.039	1.01	-1.03	-1.02	-1.04	*Ndufb11*	NADH dehydrogenase (ubiquinone) 1 beta subcomplex, 11	0006810 // transport // inferred from electronic annotation///0055114 // oxidation-redu
NM_0011	0.0257			-1.09	-1.39	-1.02	-1.02	*Grhl1*	grainyhead-like 1 (Drosophila)	0006351 // transcription, DNA-templated // inferred from electronic annotation///00063
NM_029342			0.044	1.12	1.03	-1.02	-1.12	*Nhej1*	nonhomologous end-joining factor 1	0006281 // DNA repair // inferred from electronic annotation///0006302 // double-stran
NM_0105	0.0024			1.11	-1.10	-1.02	1.00	*Il17a*	interleukin 17A	0006954 // inflammatory response // inferred from electronic annotation///0007166 // c
NM_0012	0.0024			-1.04	1.19	-1.02	-1.20	*Blvrb*	biliverdin reductase B (flavin reductase (NADPH))	0042167 // heme catabolic process // not recorded///0055114 // oxidation-reduction pro
NM_023058///XM_00652			0.048	1.02	1.01	-1.02	-1.09	*Pkmyt1*	protein kinase, membrane associated tyrosine/threonine 1	0006468 // protein phosphorylation // inferred from electronic annotation///0007049 //
NM_008106///XM_00652			0.048	1.07	1.01	-1.02	-1.12	*Opn1mw*	opsin 1 (cone pigments), medium-wave-sensitive (color blindness, deutan)	0007165 // signal transduction // inferred from electronic annotation///0007186 // G-pr
NM_0011	0.0203			1.04	-1.18	-1.02	1.06	*Sec14l1*	SEC14-like lipid binding 1	0006810 // transport // inferred from electronic annotation
NM_008168///XM_00653			0.007	1.18	-1.01	-1.02	-1.17	*Grik5*	glutamate receptor, ionotropic, kainate 5 (gamma 2)	0006621 // protein retention in ER lumen // not recorded///0006810 // transport // infer
NM_1337	0.0185	0.022		1.21	-1.02	-1.02	1.27	*Ctdspl*	CTD (carboxy-terminal domain, RNA polymerase II, polypeptide A) small phosphatase-	0001933 // negative regulation of protein phosphorylation // inferred from direct assay//
NM_1755	0.045			1.00	-1.12	-1.02	-1.02	*Wdfy2*	WD repeat and FYVE domain containing 2	0001934 // positive regulation of protein phosphorylation // not recorded///0045600 //
NM_0194	0.0167			1.05	1.31	-1.02	1.06	*Atf7ip*	activating transcription factor 7 interacting protein	0000122 // negative regulation of transcription from RNA polymerase II promoter // infer
NM_001166273///NM_01			0.012	-1.00	-1.13	-1.02	-1.22	*Cspg5*	chondroitin sulfate proteoglycan 5	0002230 // positive regulation of defense response to virus by host // not recorded///000

RefSeq Tr	p 24h	p 48h	p 72h	FD Air	FD 24h	FD 48h	FD 72h	Gene Symbol	Gene Title	Gene Ontology Biological Process
NM_009320///XM_		0.03		1.17	1.20	-1.02	1.12	*Slc6a6*	solute carrier family 6 (neurotransmitter transporter, taurine), member 6	0001762 // beta-alanine transport // inferred from direct assay///0006810 // transport /,
NM_0010	0.0407			1.03	-1.60	-1.02	1.01	*Edem3*	ER degradation enhancer, mannosidase alpha-like 3	0006491 // N-glycan processing // not recorded///0006516 // glycoprotein catabolic proc
NM_0118	0.0351			1.00	1.09	-1.02	-1.01	*Lamc3*	laminin gamma 3	0000904 // cell morphogenesis involved in differentiation // inferred from genetic interac
NM_0010	0.0023			-1.01	-1.13	-1.02	1.14	*Kat6a*	K(lysine) acetyltransferase 6A	0003007 // heart morphogenesis // inferred from genetic interaction///0003007 // heart
NM_1336	0.0378			-1.03	1.12	-1.02	1.08	*Ndufv1*	NADH dehydrogenase (ubiquinone) flavoprotein 1	0006120 // mitochondrial electron transport, NADH to ubiquinone // ---///0006810 // tra
NM_1530	0.0286			1.04	-1.10	-1.02	-1.07	*Gprc6a*	G protein-coupled receptor, family C, group 6, member A	0007165 // signal transduction // inferred from electronic annotation///0007186 // G-pro
NM_008868			0.009	1.07	1.05	-1.02	-1.15	*Pla2g2c*	phospholipase A2, group IIC	0006629 // lipid metabolic process // inferred from electronic annotation///0006644 // p
NM_0086	0.028			-1.20	1.07	-1.02	1.04	*Mmp8*	matrix metallopeptidase 8	0006508 // proteolysis // not recorded///0030574 // collagen catabolic process // inferre
NM_0085	0.0055			1.02	-1.25	-1.02	1.08	*Smad1*	SMAD family member 1	0000165 // MAPK cascade // inferred from mutant phenotype///0000165 // MAPK casca
NM_0262	0.038			1.08	-1.04	-1.02	1.01	*H2afy2///H2afy3*	H2A histone family, member Y2///H2A histone family, member Y3	0000122 // negative regulation of transcription from RNA polymerase II promoter // not r
NM_0010	0.0412			-1.07	-1.02	-1.02	-1.09	*Rpf2*	ribosome production factor 2 homolog (S. cerevisiae)	0000027 // ribosomal large subunit assembly // not recorded///0000463 // maturation o
NM_0010	0.046			1.04	-1.03	-1.02	1.05	*Strn4*	striatin, calmodulin binding protein 4	
		0.019		-1.06	-1.04	-1.02	-1.12	*C78228*	expressed sequence C78228	
NM_001130150///		0.016	0.049	1.26	1.25	-1.02	1.03	*Arhgef1*	Rho guanine nucleotide exchange factor (GEF) 1	0035023 // regulation of Rho protein signal transduction // inferred from electronic anno
NM_0276	0.0086			-1.03	-1.29	-1.02	-1.06	*Psd3*	pleckstrin and Sec7 domain containing 3	0016192 // vesicle-mediated transport // ---///0030182 // neuron differentiation // not r
NM_1389	0.0223			1.05	-1.07	-1.02	-1.05	*Wdr81*	WD repeat domain 81	0000132 // establishment of mitotic spindle orientation // not recorded///0007097 // nu
NM_1337	0.0357		0.046	-1.02	1.13	-1.02	1.06	*Ptcd1*	pentatricopeptide repeat domain 1	0008033 // tRNA processing // inferred from electronic annotation///0042780 // tRNA 3'
	0.0302		0.008	1.05	-1.06	-1.02	-1.10	*C80012*	expressed sequence C80012	
NM_001081065///XM_006			0.008	1.09	1.08	-1.02	-1.03	*Zfp707*	zinc finger protein 707	0006355 // regulation of transcription, DNA-templated // not recorded
NM_0010	0.0124			1.11	1.66	-1.02	-1.02	*Cd22*	CD22 antigen	0007155 // cell adhesion // inferred from electronic annotation///0007166 // cell surface
NM_010213///XM_00650			0.037	-1.00	1.10	-1.02	-1.28	*Fhl3*	four and a half LIM domains 3	0030036 // actin cytoskeleton organization // inferred from direct assay
NM_0010	0.0316			1.04	-1.23	-1.02	1.16	*Cep350*	centrosomal protein 350	0034453 // microtubule anchoring // inferred from electronic annotation
NM_0214	0.004	0.05		1.08	-1.12	-1.02	-1.01	*Rnase4*	ribonuclease, RNase A family 4	0001525 // angiogenesis // inferred from electronic annotation///0001525 // angiogenes
NM_018762///XM_006506			0.038	1.00	1.06	-1.02	-1.18	*Gp9*	glycoprotein 9 (platelet)	0007155 // cell adhesion // inferred from electronic annotation///0007596 // blood coag
NM_1448	######			1.08	-1.20	-1.02	1.04	*Rprd1a*	regulation of nuclear pre-mRNA domain containing 1A	0070940 // dephosphorylation of RNA polymerase II C-terminal domain // not recorded
NM_013876///XM		0.043		1.12	-1.05	-1.02	-1.12	*Rnf11*	ring finger protein 11	0006511 // ubiquitin-dependent protein catabolic process // inferred from physical intera
NM_020283///XM_00649			0.016	1.12	1.01	-1.02	-1.12	*B3galt1*	UDP-Gal:betaGlcNAc beta 1,3-galactosyltransferase, polypeptide 1	0006486 // protein glycosylation // not recorded///0009312 // oligosaccharide biosynthe
NM_0013	0.0021			1.02	-1.18	-1.02	1.22	*Pmp22*	peripheral myelin protein 22	0007049 // cell cycle // inferred from electronic annotation///0007050 // cell cycle arrest
NM_019685			0.012	-1.12	-1.06	-1.02	1.03	*Ruvbl1*	RuvB-like protein 1	0006281 // DNA repair // inferred from electronic annotation///0006310 // DNA recomb
NM_0013	0.0126			-1.04	1.19	-1.02	1.02	*Csk*	c-src tyrosine kinase	0001817 // regulation of cytokine production // not recorded///0002009 // morphogene
NM_001001297///NM_17			0.023	-1.05	1.06	-1.02	1.09	*Thnsl1*	threonine synthase-like 1 (bacterial)	0009088 // threonine biosynthetic process // not recorded
NM_0010	0.0114			1.00	-1.09	-1.01	1.08	*Hsd11b1*	hydroxysteroid 11-beta dehydrogenase 1	0006629 // lipid metabolic process // inferred from electronic annotation///0006704 // g
NM_018775///XM		0.013		-1.41	-1.41	-1.01	-1.11	*Tbc1d8*	TBC1 domain family, member 8	0043547 // positive regulation of GTPase activity // inferred from electronic annotation
NM_026585///XM_006506			0.013	-1.01	1.02	-1.01	1.13	*Fam21*	family with sequence similarity 21	0006810 // transport // inferred from electronic annotation///0015031 // protein transpo
NM_029645		0.023		1.06	-1.01	-1.01	-1.07	*Gatc*	glutamyl-tRNA(Gln) amidotransferase, subunit C	0006412 // translation // inferred from electronic annotation///0006450 // regulation of
NM_0086	0.0439			-1.02	1.41	-1.01	-1.04	*Ncf4*	neutrophil cytosolic factor 4	0043085 // positive regulation of catalytic activity // not recorded
NM_1336	0.0211			-1.00	-1.14	-1.01	1.06	*Arhgef5*	Rho guanine nucleotide exchange factor (GEF) 5	0002408 // myeloid dendritic cell chemotaxis // inferred from mutant phenotype///0030
NM_0075	0.003			-1.05	1.20	-1.01	1.00	*Bid*	BH3 interacting domain death agonist	0001836 // release of cytochrome c from mitochondria // inferred from direct assay///00
NM_0238	0.0408	0.008		-1.14	1.04	-1.01	-1.06	*Retnlb*	resistin like beta	
NM_0010	0.009			1.01	-1.24	-1.01	1.00	*Brd1*	bromodomain containing 1	0035902 // response to immobilization stress // inferred from electronic annotation///00
NM_1337	0.0456		0.035	1.13	-1.01	-1.01	-1.07	*Fam131a*	family with sequence similarity 131, member A	
NM_001205043///		0.04		1.14	1.09	-1.01	1.05	*Jarid2*	jumonji, AT rich interactive domain 2	0000122 // negative regulation of transcription from RNA polymerase II promoter // infer
NM_0099	0.0366			-1.00	1.10	-1.01	1.05	*Dpysl2*	dihydropyrimidinase-like 2	0001975 // response to amphetamine // inferred from electronic annotation///0006897
NM_008553			0.048	1.06	-1.11	-1.01	-1.15	*Ascl1*	achaete-scute complex homolog 1 (Drosophila)	0000122 // negative regulation of transcription from RNA polymerase II promoter // not r
NM_0253	0.0397			1.01	1.18	-1.01	-1.07	*Sharpin*	SHANK-associated RH domain interacting protein	0007005 // mitochondrion organization // inferred from mutant phenotype///0007420 /,
NM_0011	0.0309			1.03	-1.10	-1.01	1.02	*Aamdc*	adipogenesis associated Mth938 domain containing	0043066 // negative regulation of apoptotic process // inferred from mutant phenotype/,
NM_0012	0.0059			1.03	-1.10	-1.01	1.01	*Thsd1*	thrombospondin, type I, domain 1	0002244 // hematopoietic progenitor cell differentiation // inferred from genetic interact
NM_028354///XM_00651			0.035	1.01	1.02	-1.01	1.07	*Tdp1*	tyrosyl-DNA phosphodiesterase 1	0000012 // single strand break repair // inferred from mutant phenotype///0000012 // si
NM_026058///XM		0.011		1.22	1.18	-1.01	1.17	*Cers4*	ceramide synthase 4	0006629 // lipid metabolic process // inferred from electronic annotation///0030148 // s
NM_022031///XM_00650			0.047	1.04	1.01	-1.01	-1.14	*Hapln2*	hyaluronan and proteoglycan link protein 2	0001501 // skeletal system development // not recorded///0007155 // cell adhesion // n
NM_0013	0.0199			1.04	1.41	-1.01	-1.03	*Cyba*	cytochrome b-245, alpha polypeptide	0001938 // positive regulation of endothelial cell proliferation // not recorded///0003106
NM_001161428///		0.036	0.044	1.10	1.17	-1.01	-1.10	*Unc93b1*	unc-93 homolog B1 (C. elegans)	0002224 // toll-like receptor signaling pathway // inferred from mutant phenotype///000
NM_001313888///NM_00			0.028	-1.07	1.00	-1.01	1.04	*Il7*	interleukin 7	0002360 // T cell lineage commitment // inferred from mutant phenotype///0006955 // i
NM_0012	0.0398			1.02	-1.04	-1.01	1.20	*Kif1b*	kinesin family member 1B	0007018 // microtubule-based movement // inferred from direct assay///0007270 // neu
NM_0107	0.0283			-1.02	-1.29	-1.01	-1.15	*Mxd4*	Max dimerization protein 4	0006351 // transcription, DNA-templated // inferred from electronic annotation///00063
NM_001033175		0.031		1.12	1.07	-1.01	1.16	*Cln6*	ceroid-lipofuscinosis, neuronal 6	0001573 // ganglioside metabolic process // not recorded///0001573 // ganglioside meta
NM_0197	0.0378			1.01	-1.09	-1.01	-1.09	*Nme4*	NME/NM23 nucleoside diphosphate kinase 4	0006163 // purine nucleotide metabolic process // not recorded///0006165 // nucleoside
NM_008572///XM_00651			0.024	1.14	1.10	-1.01	-1.14	*Mcpt8*	mast cell protease 8	0006508 // proteolysis // inferred from electronic annotation///0006955 // immune resp
NM_027493///XM_00651			0.045	-1.03	1.07	-1.01	1.14	*Actr8*	ARP8 actin-related protein 8	0006281 // DNA repair // inferred from electronic annotation///0006310 // DNA recomb
NM_008052///XM_01124			0.049	1.04	1.07	-1.01	-1.18	*Dtx1*	deltex 1 homolog (Drosophila)	0007219 // Notch signaling pathway // inferred from direct assay///0007219 // Notch sig
NM_0010	0.0056			1.02	-1.12	-1.01	1.05	*Fhl1*	four and a half LIM domains 1	0003254 // regulation of membrane depolarization // not recorded///0007275 // multice
NM_0012	0.0092			1.13	1.03	-1.01	1.19	*Jund*	jun D proto-oncogene	0000122 // negative regulation of transcription from RNA polymerase II promoter // not r
NM_133823///XM_00653			0.027	1.02	-1.15	-1.01	1.26	*Mmaa*	methylmalonic aciduria (cobalamin deficiency) type A	0009236 // cobalamin biosynthetic process // inferred from electronic annotation
NM_0011	0.0057			1.01	1.32	-1.01	1.07	*Meis1*	Meis homeobox 1	0001525 // angiogenesis // inferred from mutant phenotype///0002089 // lens morphog

RefSeq Tr	*p* 24h	*p* 48h	*p* 72h	FD Air	FD 24h	FD 48h	FD 72h	Gene Symbol	Gene Title	Gene Ontology Biological Process
NM_0265	0.0382			1.24	-1.14	-1.01	1.10	***Upf3b***	UPF3 regulator of nonsense transcripts homolog B (yeast)	0000184 // nuclear-transcribed mRNA catabolic process, nonsense-mediated decay // no
NM_0010	0.0343			1.03	1.35	-1.01	1.07	***Pgd***	phosphogluconate dehydrogenase	0005975 // carbohydrate metabolic process // not recorded///0006098 // pentose-phos
NM_0010	0.0214			1.06	-1.10	-1.01	1.03	***Mecp2***	methyl CpG binding protein 2	0000122 // negative regulation of transcription from RNA polymerase II promoter // not
NM_0110	0.036			1.13	-1.08	-1.01	-1.08	***Phex***	phosphate regulating endopeptidase homolog, X-linked	0006508 // proteolysis // not recorded///0019637 // organophosphate metabolic proces
NM_010108///XM_00650			0.015	1.07	1.03	-1.01	1.15	***Efna3***	ephrin A3	0007411 // axon guidance // not recorded///0045664 // regulation of neuron differentia
NM_0011	0.012			1.09	-1.11	-1.01	1.11	***Rnf146***	ring finger protein 146	0016055 // Wnt signaling pathway // inferred from electronic annotation///0016567 // p
NM_0200	0.0249			-1.04	1.06	-1.01	-1.07	***Dhodh***	dihydroorotate dehydrogenase	0006207 // 'de novo' pyrimidine nucleobase biosynthetic process // not recorded///0006
NM_145540///NM_17887			0.046	-1.17	-1.13	-1.01	1.05	***Ints3***	integrator complex subunit 3	0006281 // DNA repair // not recorded///0006974 // cellular response to DNA damage st
NM_0100	0.0348			1.12	-1.10	-1.01	1.04	***Dapk2***	death-associated protein kinase 2	0006468 // protein phosphorylation // not recorded///0006468 // protein phosphorylati
NM_007376		0.022		-1.15	-1.07	-1.01	-1.13	***Pzp***	pregnancy zone protein	0007566 // embryo implantation // inferred from genetic interaction///0010466 // negat
NM_025912///XM		0.021		1.06	1.02	-1.01	-1.09	***Fam210b***	family with sequence similarity 210, member B	
NM_001205330///NM_00			0.042	-1.07	-1.19	-1.01	1.10	***Map4***	microtubule-associated protein 4	0007052 // mitotic spindle organization // inferred from direct assay///0007052 // mitoti
NM_1725	0.0353			1.07	-1.05	-1.01	-1.02	***Boc***	biregional cell adhesion molecule-related/down-regulated by oncogenes (Cdon) bindir	0007155 // cell adhesion // inferred from electronic annotation///0007224 // smoothene
NM_0335	######			1.09	-1.33	-1.01	1.08	***Klf7***	Kruppel-like factor 7 (ubiquitous)	0006351 // transcription, DNA-templated // inferred from electronic annotation///00063
NM_001081650///NM_00			0.023	1.09	1.18	-1.01	-1.04	***Rgs3***	regulator of G-protein signaling 3	0007165 // signal transduction // not recorded///0007186 // G-protein coupled receptor
NM_0262	0.0381			1.27	-1.10	-1.01	1.06	***Fibin***	fin bud initiation factor homolog (zebrafish)	
NM_0112	0.0264	0.021		1.14	1.02	-1.01	1.05	***Rad9a***	RAD9 homolog A	0000075 // cell cycle checkpoint // inferred from electronic annotation///0000076 // DN
NM_0011	0.0426			-1.20	-1.60	-1.01	-1.06	***Fxr1***	fragile X mental retardation gene 1, autosomal homolog	0007275 // multicellular organismal development // inferred from electronic annotation/
NM_010098///XM		0.047		-1.09	-1.03	-1.01	-1.14	***Opn3***	opsin 3	0007165 // signal transduction // inferred from electronic annotation///0007186 // G-pr
NM_0089	0.0124			1.04	-1.24	-1.01	-1.03	***Ptger2***	prostaglandin E receptor 2 (subtype EP2)	0007165 // signal transduction // inferred from electronic annotation///0007186 // G-pr
NM_001290299///NM_14			0.037	1.04	1.02	-1.01	-1.16	***Plppr2***	phospholipid phosphatase related 2	0006470 // protein dephosphorylation // inferred from electronic annotation///0006644
NM_134097			0.038	-1.02	1.07	-1.01	1.18	***Topors***	topoisomerase I binding, arginine/serine-rich	0006511 // ubiquitin-dependent protein catabolic process // not recorded///0006513 //
NM_0011	0.0328			1.16	-1.03	-1.01	1.11	***Islr***	immunoglobulin superfamily containing leucine-rich repeat	
NM_0012	0.0147			1.02	1.18	-1.01	-1.12	***Arhgap9***	Rho GTPase activating protein 9	0007165 // signal transduction // inferred from electronic annotation///0043547 // positi
NM_001305185///NM_00			0.009	1.08	-1.02	-1.01	-1.07	***Plekha7***	pleckstrin homology domain containing, family A member 7	0045218 // zonula adherens maintenance // not recorded///0090136 // epithelial cell-ce
NM_0205	0.0125			-1.02	1.07	-1.01	-1.01	***Flad1///Lenep***	RFad1, flavin adenine dinucleotide synthetase, homolog (yeast)///lens epithelial prote	0006747 // FAD biosynthetic process // inferred from electronic annotation///0007275 /
NM_026639		0.017	0.002	-1.13	-1.07	-1.01	1.16	***Art4***	ADP-ribosyltransferase 4	0006471 // protein ADP-ribosylation // inferred from electronic annotation
NM_0013	0.0226			-1.15	-1.00	-1.01	-1.03	***Ahcy///Gm4737***	S-adenosylhomocysteine hydrolase///predicted gene 4737	0002439 // chronic inflammatory response to antigenic stimulus // not recorded///0006
NM_0098	0.0298			1.12	1.70	-1.01	-1.04	***Cd19***	CD19 antigen	0006968 // cellular defense response // not recorded///0007173 // epidermal growth fa
NM_0074	0.0393			1.09	-1.45	-1.01	1.06	***Adrb2***	adrenergic receptor, beta 2	0001666 // response to hypoxia // inferred from electronic annotation///0001889 // live
NM_0206	0.0151			-1.03	-1.17	-1.01	-1.01	***Cramp1l***	Crm, cramped-like (Drosophila)	
NM_0110	0.0317			-1.09	1.05	-1.01	1.14	***Pfdn2***	prefoldin 2	0006457 // protein folding // inferred from direct assay///0051495 // positive regulation
NM_0011	0.0186			-1.04	1.25	-1.01	1.01	***Mical1***	microtubule associated monooxygenase, calponin and LIM domain containing 1	0001933 // negative regulation of protein phosphorylation // inferred from mutant phen
NM_1460	0.0061			1.05	1.31	-1.01	-1.04	***Nrros***	negative regulator of reactive oxygen species	0002376 // immune system process // inferred from electronic annotation///0006801 //
NM_0187	0.0072			1.06	-1.09	-1.01	1.10	***Atp6ap1***	ATPase, H+ transporting, lysosomal accessory protein 1	0006810 // transport // inferred from electronic annotation///0006811 // ion transport /
NM_011693			0.047	-1.19	-1.23	-1.01	1.11	***Vcam1***	vascular cell adhesion molecule 1	0007155 // cell adhesion // inferred from direct assay///0007155 // cell adhesion // not r
NM_0013	0.0162			1.02	1.25	-1.01	1.01	***Cnr2***	cannabinoid receptor 2 (macrophage)	0001975 // response to amphetamine // inferred from electronic annotation///0006954
NM_007650///XM_00652			0.015	1.06	1.16	-1.01	-1.28	***Cd5***	CD5 antigen	0006898 // receptor-mediated endocytosis // inferred from electronic annotation///0031
NM_001105066///		0.05		-1.02	1.04	-1.01	-1.04	***Vmn2r34///Vmn2r42///Vmn2r45***	vomeronasal 2, receptor 34///vomeronasal 2, receptor 42///vomeronasal 2, receptor	0007165 // signal transduction // inferred from electronic annotation///0007186 // G-pr
NM_001004150///NM_00			0.04	-1.18	-1.13	-1.01	1.27	***A4galt***	alpha 1,4-galactosyltransferase	0001576 // globoside biosynthetic process // inferred from direct assay///0001576 // glo
NM_0011	0.0011			1.15	-1.24	-1.01	1.12	***Slc4a5***	solute carrier family 4, sodium bicarbonate cotransporter, member 5	0002064 // epithelial cell development // inferred from mutant phenotype///0003014 //
NM_0091	0.0478			-1.09	1.21	-1.01	-1.06	***Selplg***	selectin, platelet (p-selectin) ligand	0007155 // cell adhesion // inferred from electronic annotation///0050901 // leukocyte t
NM_008962///XR_383147			0.006	1.00	1.01	-1.01	-1.18	***Ptgdr***	prostaglandin D receptor	0006954 // inflammatory response // inferred from mutant phenotype///0007165 // sign
NM_001033231///XM_011			0.006	1.09	1.02	-1.01	-1.11	***Fam195b***	family with sequence similarity 195, member B	0010717 // regulation of epithelial to mesenchymal transition // not recorded
NM_009630///XM_00651			0.024	1.01	1.08	-1.01	-1.11	***Adora2a***	adenosine A2a receptor	0001963 // synaptic transmission, dopaminergic // inferred from genetic interaction///00
NM_0011	0.0082	0.035		1.17	-1.02	-1.01	1.04	***Tmem80***	transmembrane protein 80	
NM_001146023///NM_02			0.018	1.13	1.16	-1.00	-1.02	***Fam98c***	family with sequence similarity 98, member C	
NM_1393	0.0136			1.05	-1.14	-1.00	1.08	***Vasn***	vasorin	0010719 // negative regulation of epithelial to mesenchymal transition // not recorded//
NM_016854		0.049		-1.18	-1.08	-1.00	-1.09	***Ppp1r3c***	protein phosphatase 1, regulatory (inhibitor) subunit 3C	0005975 // carbohydrate metabolic process // inferred from electronic annotation///000
NM_001291442///		0.024		1.05	1.00	-1.00	1.14	***Micu1***	mitochondrial calcium uptake 1	0006810 // transport // inferred from electronic annotation///0006811 // ion transport /
NM_0011	0.0211			1.09	-1.06	-1.00	1.04	***Enpp2***	ectonucleotide pyrophosphatase/phosphodiesterase 2	0001953 // negative regulation of cell-matrix adhesion // not recorded///0006629 // lipi
NM_001047158///NM_19			0.026	1.04	1.01	-1.00	-1.06	***Uqcr10***	ubiquinol-cytochrome c reductase, complex III subunit X	0006122 // mitochondrial electron transport, ubiquinol to cytochrome c // inferred from
NM_0199	0.0288			1.06	-1.14	-1.00	-1.13	***Zfp235***	zinc finger protein 235	0006355 // regulation of transcription, DNA-templated // inferred from electronic annota
NM_0214	0.0191			1.14	-1.17	-1.00	-1.17	***Shank3***	SH3/ankyrin domain gene 3	0000165 // MAPK cascade // inferred from genetic interaction///0001838 // embryonic e
NM_0010	0.0404			-1.94	-5.56	-1.00	-1.16	***Zbtb16***	zinc finger and BTB domain containing 16	0000122 // negative regulation of transcription from RNA polymerase II promoter // not
NM_130892///XR_		0.019		-1.06	1.02	-1.00	-1.01	***Rtn4ip1***	reticulon 4 interacting protein 1	0055114 // oxidation-reduction process // inferred from electronic annotation
NM_0010	######	0.039		1.10	-1.13	-1.00	-1.07	***Zbtb2***	zinc finger and BTB domain containing 2	
NM_0013	0.0316			1.20	-1.08	-1.00	1.01	***Sprydt7***	SPRY domain containing 7	
NM_023463			0.008	1.02	1.09	-1.00	1.21	***Ly6g6c***	lymphocyte antigen 6 complex, locus G6C	
NM_0213	0.0272		0.027	1.17	-1.06	-1.00	-1.19	***Slc26a3***	solute carrier family 26, member 3	0006810 // transport // inferred from electronic annotation///0006821 // chloride transp
NM_0091	######			1.00	-1.13	-1.00	1.00	***Slc16a2***	solute carrier family 16 (monocarboxylic acid transporters), member 2	0006810 // transport // inferred from electronic annotation///0009914 // hormone trans
NM_025963///XM_01124			0.011	1.03	1.13	-1.00	-1.04	***Rps10///Rps10-ps2***	ribosomal protein S10///ribosomal protein S10, pseudogene 2	0000028 // ribosomal small subunit assembly // ---
NM_0010	0.009			1.10	-1.12	-1.00	1.06	***Syne1***	spectrin repeat containing, nuclear envelope 1	0007030 // Golgi organization // not recorded///0040023 // establishment of nucleus loc
NM_0013	0.0139			1.06	-1.23	-1.00	1.03	***Tpk1***	thiamine pyrophosphokinase	0006772 // thiamine metabolic process // inferred from direct assay///0009229 // thiami

RefSeq Tr	p 24h	p 48h	p 72h	FD Air	FD 24h	FD 48h	FD 72h	Gene Symbol	Gene Title	Gene Ontology Biological Process
NM_001172112///		0.024	0.035	-1.12	-1.01	-1.00	1.08	***Dhrs7b***	dehydrogenase/reductase (SDR family) member 7B	0008152 // metabolic process // inferred from electronic annotation///0055114 // oxidat
NM_0010	0.0394			-1.05	1.07	1.00	-1.13	***Tnfrsf9***	tumor necrosis factor receptor superfamily, member 9	0006954 // inflammatory response // not recorded///0006955 // immune response // nc
NM_0086	0.0471			-1.07	1.07	1.00	-1.11	***Msh2***	mutS homolog 2 (E. coli)	0000710 // meiotic mismatch repair // not recorded///0001701 // in utero embryonic de
NM_0011	0.045			1.00	1.26	1.00	1.03	***Sh3bp2***	SH3-domain binding protein 2	
NM_0011	0.0035			-1.05	1.12	1.00	1.10	***Ptpra***	protein tyrosine phosphatase, receptor type, A	0006468 // protein phosphorylation // inferred from direct assay///0006470 // protein d
NM_0194	0.0209			1.03	1.49	1.00	1.15	***Tmem37***	transmembrane protein 37	0006810 // transport // inferred from electronic annotation///0006811 // ion transport /
NM_0011	0.0131		0.003	1.22	1.09	1.00	1.04	***Prrc2a***	proline-rich coiled-coil 2A	
NM_0166	0.0374			1.01	1.14	1.00	-1.01	***Cldn2***	claudin 2	0016338 // calcium-independent cell-cell adhesion via plasma membrane cell-adhesion n
NM_1722	0.0203			1.05	1.31	1.00	-1.01	***Plcg2***	phospholipase C, gamma 2	0002092 // positive regulation of receptor internalization // inferred from mutant phenot
NM_0074	0.0357			-1.08	1.02	1.00	-1.14	***Adra2a***	adrenergic receptor, alpha 2a	0001819 // positive regulation of cytokine production // not recorded///0002526 // acut
NM_0117	0.045			-1.08	1.19	1.00	-1.23	***Wnt9b***	wingless-type MMTV integration site family, member 9B	0001658 // branching involved in ureteric bud morphogenesis // inferred from genetic in
NM_001045525///XM_011			0.044	1.00	1.04	1.00	1.17	***Cyb5d1***	cytochrome b5 domain containing 1	
NM_1454	0.0134			-1.01	1.14	1.00	1.09	***Stk24***	serine/threonine kinase 24	0006468 // protein phosphorylation // not recorded///0006915 // apoptotic process // ir
NM_024288///XM_00650			0.013	1.02	-1.04	1.00	1.25	***Rmnd5a***	required for meiotic nuclear division 5 homolog A (S. cerevisiae)	
NM_146168///XM_00650			0.021	-1.14	-1.23	1.00	1.08	***Vopp1***	vesicular, overexpressed in cancer, prosurvival protein 1	0006351 // transcription, DNA-templated // inferred from electronic annotation///00063
NM_175324			0.004	1.01	-1.04	1.00	1.18	***Acad11***	acyl-Coenzyme A dehydrogenase family, member 11	0008152 // metabolic process // inferred from electronic annotation///0033539 // fatty a
NM_0010	0.0478			-1.12	1.03	1.00	-1.01	***St3gal5***	ST3 beta-galactoside alpha-2,3-sialyltransferase 5	0001574 // ganglioside biosynthetic process // not recorded///0006486 // protein glycos
NM_1725	0.042			-1.02	1.14	1.00	1.03	***Rufy1***	RUN and FYVE domain containing 1	0006810 // transport // inferred from electronic annotation///0006897 // endocytosis //
NM_025914///XM_00651			0.031	-1.05	-1.14	1.00	1.06	***Actr6***	ARP6 actin-related protein 6	0006338 // chromatin remodeling // inferred from electronic annotation
NM_0074	0.0027			-1.08	1.15	1.00	-1.01	***Adora2b***	adenosine A2b receptor	0001973 // adenosine receptor signaling pathway // inferred from electronic annotation/
NM_001033908///		0.045	0.03	1.14	1.00	1.00	-1.08	***Med22***	mediator complex subunit 22	0006351 // transcription, DNA-templated // inferred from electronic annotation///00063
NM_0010	######			-1.04	1.30	1.00	1.20	***Eno1///Eno1b***	enolase 1, alpha non-neuron///enolase 1B, retrotransposed	0001701 // in utero embryonic development // inferred from mutant phenotype///00060
NM_0010	0.0387			1.07	-1.08	1.00	1.07	***Adam28***	a disintegrin and metallopeptidase domain 28	0006508 // proteolysis // inferred from electronic annotation///0007229 // integrin-med
NM_134133///XM		0.015		-1.17	-1.23	1.00	-1.03	***Smim3***	small integral membrane protein 3	
NM_0012	0.0014			-1.02	1.31	1.00	-1.14	***B4galnt1***	beta-1,4-N-acetyl-galactosaminyl transferase 1	0001574 // ganglioside biosynthetic process // inferred from genetic interaction///00015
NM_1339	0.0025			1.01	1.27	1.00	-1.13	***Chpf2***	chondroitin polymerizing factor 2	0008152 // metabolic process // inferred from electronic annotation
NM_027909///XM_00651			0.034	1.05	1.06	1.00	-1.00	***C2cd2l***	C2 calcium-dependent domain containing 2-like	0035774 // positive regulation of insulin secretion involved in cellular response to glucose
NM_0198	0.0207			-1.02	-1.24	1.00	-1.08	***Gkap1***	G kinase anchoring protein 1	0007165 // signal transduction // inferred from direct assay///0007199 // G-protein coup
NM_025989///XM		0.03		-1.17	-1.11	1.00	1.06	***Gp2***	glycoprotein 2 (zymogen granule membrane)	0002412 // antigen transcytosis by M cells in mucosal-associated lymphoid tissue // infer
NM_008598///XM_00650			0.023	1.05	1.00	1.00	-1.18	***Mgmt***	O-6-methylguanine-DNA methyltransferase	0006281 // DNA repair // inferred from mutant phenotype///0006307 // DNA dealkylatic
NM_031406///XM_00650			0.017	1.12	1.06	1.00	-1.03	***Slc12a9***	solute carrier family 12 (potassium/chloride transporters), member 9	0006810 // transport // inferred from electronic annotation///0006811 // ion transport /
NM_0260	0.0079			1.04	1.18	1.00	1.11	***Ikbkap***	inhibitor of kappa light polypeptide enhancer in B cells, kinase complex-associated pro	0006351 // transcription, DNA-templated // inferred from electronic annotation///00063
NM_0075	0.0087			1.02	1.19	1.00	-1.15	***Phb2***	prohibitin 2	0000060 // protein import into nucleus, translocation // not recorded///0006351 // trans
NM_0532	0.0446			-1.10	1.11	1.00	-1.06	***Myo1f***	myosin IF	0002446 // neutrophil mediated immunity // inferred from mutant phenotype///00071
NM_0012	0.0207		0.048	1.07	-1.29	1.00	1.33	***Cipc***	CLOCK interacting protein, circadian	0006351 // transcription, DNA-templated // inferred from electronic annotation///00063
NM_0090	0.0324			-1.07	1.06	1.00	1.04	***Rasgrf2***	RAS protein-specific guanine nucleotide-releasing factor 2	0007264 // small GTPase mediated signal transduction // inferred from electronic annota
NM_145416			0.016	1.07	-1.04	1.00	-1.09	***Kri1***	KRI1 homolog (S. cerevisiae)	
NM_0010	0.0223			1.07	-1.00	1.00	1.02	***Atxn7l3b***	ataxin 7-like 3B	
NM_001185083///NM_00			0.022	1.07	1.02	1.00	-1.09	***Ins2***	insulin II	0000122 // negative regulation of transcription from RNA polymerase II promoter // infer
NM_0013	0.0451	0.002		1.09	-1.02	1.00	1.02	***Tmub2***	transmembrane and ubiquitin-like domain containing 2	
NM_001081339///		0.034		-1.11	-1.15	1.00	-1.19	***Xirp1***	xin actin-binding repeat containing 1	0003007 // heart morphogenesis // not recorded///0007219 // Notch signaling pathway
NM_0011	0.003			1.11	-1.33	1.00	-1.04	***Gjc1***	gap junction protein, gamma 1	0001570 // vasculogenesis // inferred from mutant phenotype///0007154 // cell commu
NM_0011	0.0038			-1.05	1.18	1.01	-1.14	***Psmg4***	proteasome (prosome, macropain) assembly chaperone 4	0043248 // proteasome assembly // inferred from electronic annotation
NM_001290011///NM_00			0.047	1.06	-1.04	1.01	-1.10	***Pemt***	phosphatidylethanolamine N-methyltransferase	0006629 // lipid metabolic process // inferred from electronic annotation///0006644 // p
NM_1988	0.0321			1.12	-1.04	1.01	-1.07	***Scx***	scleraxis	0001707 // mesoderm formation // inferred from mutant phenotype///0001894 // tissue
NM_001122958///		0.014		-1.12	-1.04	1.01	-1.01	***Rad54l***	RAD54 like (S. cerevisiae)	0000724 // double-strand break repair via homologous recombination // inferred from g
NM_0012	0.0226	0.04		-1.07	1.03	1.01	-1.08	***D16Ertd472e***	DNA segment, Chr 16, ERATO Doi 472, expressed	
NM_0013	0.0417			-1.01	1.11	1.01	1.10	***Psmf1***	proteasome (prosome, macropain) inhibitor subunit 1	0006511 // ubiquitin-dependent protein catabolic process // not recorded///1901799 //
NM_177876			0.047	1.03	1.12	1.01	-1.19	***Vps37b***	vacuolar protein sorting 37B (yeast)	0006810 // transport // inferred from electronic annotation///0015031 // protein transp
NM_0013	0.0373			1.01	1.32	1.01	-1.13	***Unc119***	unc-119 homolog (C. elegans)	0006810 // transport // inferred from electronic annotation///0006897 // endocytosis //
NM_001164082///NM_02			0.033	-1.01	1.06	1.01	1.01	***Polr3d***	polymerase (RNA) III (DNA directed) polypeptide D	0002376 // immune system process // inferred from electronic annotation///0006351 //
NM_0011	0.0089			-1.16	1.11	1.01	-1.11	***Lmo4***	LIM domain only 4	0001843 // neural tube closure // inferred from mutant phenotype///0003281 // ventricu
NM_1454	0.0181			-1.01	1.09	1.01	-1.02	***Heatr6***	HEAT repeat containing 6	
NM_0011	######			1.02	-1.24	1.01	1.10	***Tsc22d1***	TSC22 domain family, member 1	0006351 // transcription, DNA-templated // inferred from electronic annotation///00063
NM_009076///XR_880074			0.007	1.02	1.22	1.01	-1.15	***LOC102635048///Rpl12///Rpl12-ps1***	60S ribosomal protein L12 pseudogene///ribosomal protein L12///ribosomal protein L	0000027 // ribosomal large subunit assembly // ---///0006412 // translation // inferred fr
NM_0195	0.0295			1.01	-1.27	1.01	1.00	***Plce1***	phospholipase C, epsilon 1	0000187 // activation of MAPK activity // not recorded///0006629 // lipid metabolic proc
NM_0011	0.0441			1.13	-1.02	1.01	1.05	***Sprtn***	SprT-like N-terminal domain	0006281 // DNA repair // inferred from electronic annotation///0006974 // cellular respo
NM_0010	0.0376			1.01	-1.12	1.01	1.09	***Myrf***	myelin regulatory factor	0006351 // transcription, DNA-templated // inferred from electronic annotation///00063
NM_025857///XM_00651			0.006	-1.02	1.02	1.01	1.11	***Aagab***	alpha- and gamma-adaptin binding protein	0006810 // transport // inferred from electronic annotation///0015031 // protein transp
NM_0540	0.0438	0.047		1.14	-1.03	1.01	1.13	***Pip4k2c***	phosphatidylinositol-5-phosphate 4-kinase, type II, gamma	0010506 // regulation of autophagy // not recorded///0016310 // phosphorylation // inf
NM_025465///XM		0.013	0.036	-1.12	1.01	1.01	-1.02	***Tma16***	translation machinery associated 16 homolog (S. cerevisiae)	
NM_0111	0.0356			-1.01	1.08	1.01	1.17	***Pa2g4***	proliferation-associated 2G4	0006351 // transcription, DNA-templated // inferred from electronic annotation///00063
NM_030168///XM		0.016		-1.06	-1.01	1.01	1.01	***Rictor***	RPTOR independent companion of MTOR, complex 2	0001932 // regulation of protein phosphorylation // inferred from mutant phenotype///0
NM_0306	0.0329			1.07	-1.26	1.01	1.02	***Kank3***	KN motif and ankyrin repeat domains 3	0051497 // negative regulation of stress fiber assembly // not recorded

RefSeq Tr	*p* 24h	*p* 48h	*p* 72h	FD Air	FD 24h	FD 48h	FD 72h	Gene Symbol	Gene Title	Gene Ontology Biological Process
NM_0011	0.0408			1.02	1.24	1.01	-1.15	***Sema4a***	sema domain, immunoglobulin domain (Ig), transmembrane domain (TM) and short c	0001525 // angiogenesis // inferred from electronic annotation///0001755 // neural cres
NM_0013	0.0398			1.15	-1.08	1.01	-1.06	***Slc26a1***	solute carrier family 26 (sulfate transporter), member 1	0006810 // transport // inferred from electronic annotation///0006811 // ion transport /
NM_0235	0.0484			1.14	-1.07	1.01	-1.03	***Arid5b***	AT rich interactive domain 5B (MRF1-like)	0000122 // negative regulation of transcription from RNA polymerase II promoter // not
NM_1748	0.0232			1.03	-1.21	1.01	1.03	***Crybg3***	beta-gamma crystallin domain containing 3	
NM_178395///XM_		0.017	0.005	-1.15	-1.05	1.01	1.08	***Zdhhc2***	zinc finger, DHHC domain containing 2	0006612 // protein targeting to membrane // ---///0018230 // peptidyl-L-cysteine S-palm
NM_0103	0.0436			1.00	1.39	1.01	-1.15	***Gm20506///H2-Ob***	predicted gene 20506///histocompatibility 2, O region beta locus	0002376 // immune system process // inferred from electronic annotation///0002504 //
NM_0103	0.0436			1.00	1.39	1.01	-1.15	***Gm20506///H2-Ob***	predicted gene 20506///histocompatibility 2, O region beta locus	0002376 // immune system process // inferred from electronic annotation///0002504 //
NM_0090	0.0245			-1.02	1.12	1.01	1.12	***Rgs4***	regulator of G-protein signaling 4	0007186 // G-protein coupled receptor signaling pathway // traceable author statement/
NM_021288///NR_		0.02	0.022	-1.22	-1.02	1.01	1.21	***Tyms///Tyms-ps***	thymidylate synthase///thymidylate synthase, pseudogene	0006206 // pyrimidine nucleobase metabolic process // inferred from electronic annotati
NM_001311141///NM_00			0.048	1.15	1.01	1.01	-1.06	***Il12rb2***	interleukin 12 receptor, beta 2	0018108 // peptidyl-tyrosine phosphorylation // inferred from mutant phenotype///001
NM_1459	0.0295			1.02	-1.15	1.01	-1.16	***N4bp3***	NEDD4 binding protein 3	
NM_0013	0.0378	0.034	0.004	1.26	1.11	1.01	1.07	***Impdh1***	inosine 5'-phosphate dehydrogenase 1	0006164 // purine nucleotide biosynthetic process // inferred from mutant phenotype///
NM_0109	0.0404			1.14	-1.33	1.01	1.19	***Nov***	nephroblastoma overexpressed gene	0001525 // angiogenesis // not recorded///0001558 // regulation of cell growth // infer
NM_0258	0.0271			1.15	-1.03	1.01	1.22	***Slc25a23***	solute carrier family 25 (mitochondrial carrier; phosphate carrier), member 23	0002082 // regulation of oxidative phosphorylation // inferred from mutant phenotype//
NM_0108	0.0104			-1.12	1.23	1.01	-1.02	***Ncf2***	neutrophil cytosolic factor 2	0006742 // NADP catabolic process // not recorded///0006801 // superoxide metabolic
NM_0198	0.0196			1.02	-1.15	1.01	1.01	***Rbm14***	RNA binding motif protein 14	0006351 // transcription, DNA-templated // inferred from electronic annotation///00063
NM_0011	0.0133	0.046	0.04	1.15	1.03	1.01	1.03	***Kifc3***	kinesin family member C3	0007017 // microtubule-based process // inferred from direct assay///0007018 // microt
NM_1332	0.0264			1.05	-1.16	1.01	1.01	***Zfp87***	zinc finger protein 87	0006355 // regulation of transcription, DNA-templated // inferred from electronic annot
NM_0011	0.026			1.01	-1.41	1.01	1.05	***Fas***	Fas (TNF receptor superfamily member 6)	0002377 // immunoglobulin production // inferred from mutant phenotype///0003014 /
NM_0083	0.0033			1.09	1.40	1.01	1.13	***Il17ra***	interleukin 17 receptor A	0019221 // cytokine-mediated signaling pathway // not recorded///0032747 // positive
NM_134246///XM_00651			0.047	1.08	1.01	1.01	-1.08	***Acot3***	acyl-CoA thioesterase 3	0000038 // very long-chain fatty acid metabolic process // inferred from direct assay///0
NM_0083	0.0243			1.11	1.64	1.01	-1.28	***Gimap1***	GTPase, IMAP family member 1	0030183 // B cell differentiation // inferred from mutant phenotype///0030217 // T cell
NM_0101	0.0133			-1.04	1.14	1.01	1.04	***Fes***	feline sarcoma oncogene	0001578 // microtubule bundle formation // inferred from direct assay///0006468 // pro
NM_026185			0.04	1.06	1.03	1.01	-1.20	***Abhd15***	abhydrolase domain containing 15	0008152 // metabolic process // inferred from electronic annotation
NM_009329///XM_		0.042		-1.08	-1.00	1.01	-1.08	***Zfp354a***	zinc finger protein 354A	0000122 // negative regulation of transcription from RNA polymerase II promoter // not
NM_178616///XM_		0.009		-1.12	-1.03	1.01	-1.09	***Psmd11***	proteasome (prosome, macropain) 26S subunit, non-ATPase, 11	0006511 // ubiquitin-dependent protein catabolic process // not recorded///0043248 //
NM_0198	0.0026			1.06	-1.11	1.01	1.12	***Ivd***	isovaleryl coenzyme A dehydrogenase	0006552 // leucine catabolic process // not recorded///0008152 // metabolic process //
NM_133911		0.049		-1.19	-1.37	1.01	-1.02	***Adgra3***	adhesion G protein-coupled receptor A3	0007165 // signal transduction // inferred from electronic annotation///0007166 // cell s
NM_1331	0.0426			-1.20	1.03	1.01	1.06	***Pygl***	liver glycogen phosphorylase	0005975 // carbohydrate metabolic process // inferred from electronic annotation///000
NM_0105	0.0141			1.01	1.32	1.01	-1.10	***Il16***	interleukin 16	0006351 // transcription, DNA-templated // inferred from electronic annotation///00063
NM_0114	0.0428			1.14	-1.07	1.01	1.16	***Snai1***	snail family zinc finger 1	0000122 // negative regulation of transcription from RNA polymerase II promoter // infe
NM_025586///XM_00651			0.006	1.01	1.08	1.01	-1.03	***Gm4581///Rpl15///Rpl15-ps3***	predicted gene 4581///ribosomal protein L15///ribosomal protein L15, pseudogene 3	0002181 // cytoplasmic translation // ---///0006412 // translation // inferred from electr
NM_019631///XM_00652			0.019	-1.06	-1.21	1.01	1.09	***Tmem45a***	transmembrane protein 45a	
NM_001162538///		0.022		-1.05	-1.04	1.02	-1.05	***Odf2l***	outer dense fiber of sperm tails 2-like	
NM_0116	0.0218			1.05	1.27	1.02	1.06	***Cd40***	CD40 antigen	0001934 // positive regulation of protein phosphorylation // not recorded///0002376 //
NM_1786	0.0078			1.01	1.22	1.02	1.02	***Samm50***	sorting and assembly machinery component 50 homolog (S. cerevisiae)	0033108 // mitochondrial respiratory chain complex assembly // not recorded///004240
NM_0011	0.0313			1.00	-1.22	1.02	1.07	***Yap1***	yes-associated protein 1	0000902 // cell morphogenesis // inferred from mutant phenotype///0001570 // vasculo
NM_0530	0.0047			-1.02	1.21	1.02	1.02	***Chrac1***	chromatin accessibility complex 1	0008152 // metabolic process // inferred from electronic annotation///0071897 // DNA
NM_025445///XM_		0.029		-1.12	-1.00	1.02	1.10	***Arfgap3***	ADP-ribosylation factor GTPase activating protein 3	0006810 // transport // inferred from electronic annotation///0009306 // protein secreti
NM_0011	0.005		0.007	-1.10	1.38	1.02	1.16	***Psap***	prosaposin	0006629 // lipid metabolic process // inferred from electronic annotation///0006665 // s
NM_013522			0.044	-1.02	1.06	1.02	1.37	***Frg1***	FSHD region gene 1	0006364 // rRNA processing // inferred from electronic annotation///0006397 // mRNA
NM_001282071///		0.001	0.017	1.14	1.20	1.02	-1.06	***Sftpb***	surfactant associated protein B	0006629 // lipid metabolic process // inferred from electronic annotation///0006665 // s
NM_0011	0.0327			1.12	1.27	1.02	-1.36	***Dapk3***	death-associated protein kinase 3	0006351 // transcription, DNA-templated // inferred from electronic annotation///00063
NM_008743///XM_00652			0.01	1.07	1.08	1.02	-1.12	***Nthl1***	nth (endonuclease III)-like 1 (E.coli)	0002230 // positive regulation of defense response to virus by host // not recorded///00
NM_001037938///NM_03			0.018	-1.02	1.02	1.02	1.12	***Dhrs4***	dehydrogenase/reductase (SDR family) member 4	0006066 // alcohol metabolic process // not recorded///0008152 // metabolic process //
NM_025467			0.037	-1.00	-1.05	1.02	-1.17	***Gkn2***	gastrokine 2	
NM_0010	0.0163			1.03	1.30	1.02	1.12	***Spata13***	spermatogenesis associated 13	0016477 // cell migration // not recorded///0030032 // lamellipodium assembly // not re
NM_016696///XM_		0.006		-1.20	-1.06	1.02	-1.10	***Gpc1***	glypican 1	0014037 // Schwann cell differentiation // not recorded///0030200 // heparan sulfate pr
NM_028024		0.05		1.13	1.15	1.02	1.03	***Nkiras2***	NFKB inhibitor interacting Ras-like protein 2	0007165 // signal transduction // inferred from electronic annotation///0007264 // small
NM_0268	0.0225			1.10	-1.26	1.02	1.03	***Rasl11b***	RAS-like, family 11, member B	0007165 // signal transduction // inferred from electronic annotation///0007264 // small
NM_010633///XM_00649			0.006	-1.08	-1.09	1.02	1.12	***Uhmk1***	U2AF homology motif (UHM) kinase 1	0000184 // nuclear-transcribed mRNA catabolic process, nonsense-mediated decay // ---
NM_0233	0.0027			-1.07	1.15	1.02	-1.02	***Sdhb***	succinate dehydrogenase complex, subunit B, iron sulfur (Ip)	0006099 // tricarboxylic acid cycle // inferred from electronic annotation///0006105 // s
NM_1774	0.014			1.03	-1.28	1.02	-1.13	***Cyp4a12a***	cytochrome P450, family 4, subfamily a, polypeptide 12a	0006631 // fatty acid metabolic process // inferred from sequence or structural similarity
NM_0081	0.0185			-1.15	1.10	1.02	-1.08	***Gclm***	glutamate-cysteine ligase, modifier subunit	0006534 // cysteine metabolic process // inferred from mutant phenotype///0006536 //
NM_026684			0.024	1.03	-1.01	1.02	-1.08	***Ndufb10***	NADH dehydrogenase (ubiquinone) 1 beta subcomplex, 10	0006810 // transport // inferred from electronic annotation///0055114 // oxidation-redu
NM_001081392///		0.039		-1.06	-1.03	1.02	-1.05	***Mdn1***	midasin homolog (yeast)	0000027 // ribosomal large subunit assembly // inferred from electronic annotation///00
NM_026056///XM_		0.008	0.038	-1.19	-1.11	1.02	-1.07	***Cap2***	CAP, adenylate cyclase-associated protein, 2 (yeast)	0000902 // cell morphogenesis // inferred from electronic annotation///0007010 // cyto
NM_0256	0.0433			1.02	-1.11	1.02	1.07	***Uqcrh***	ubiquinol-cytochrome c reductase hinge protein	0006122 // mitochondrial electron transport, ubiquinol to cytochrome c // inferred from
NM_0214	0.0436	0.047	0.035	1.11	1.01	1.02	-1.15	***Arhgap23***	Rho GTPase activating protein 23	0007165 // signal transduction // inferred from electronic annotation///0043547 // posit
NM_144793///XM_00651			0.035	1.03	-1.06	1.02	-1.11	***Slc25a38***	solute carrier family 25, member 38	0006783 // heme biosynthetic process // inferred from electronic annotation///0006810
NM_1726	0.04			1.10	-1.16	1.02	1.07	***6-Mar***	membrane-associated ring finger (C3HC4) 6	0010498 // proteasomal protein catabolic process // not recorded///0016567 // protein
NM_0011	0.0441			1.08	-1.08	1.02	-1.07	***Zbed6///Zc3h11a***	zinc finger, BED type containing 6///zinc finger CCCH type containing 11A	0000122 // negative regulation of transcription from RNA polymerase II promoter // infe
NM_0012	0.0442			1.10	-1.03	1.02	1.04	***Ppp3ca***	protein phosphatase 3, catalytic subunit, alpha isoform	0000082 // G1/S transition of mitotic cell cycle // inferred from mutant phenotype///000
NM_0244	0.0204			1.00	1.10	1.02	1.03	***LOC102642893///Scd3***	acyl-CoA desaturase 1-like///stearoyl-coenzyme A desaturase 3	0006629 // lipid metabolic process // inferred from electronic annotation///0006631 // f

RefSeq Tr	*p* 24h	*p* 48h	*p* 72h	FD Air	FD 24h	FD 48h	FD 72h	Gene Symbol	Gene Title	Gene Ontology Biological Process
NM_0092	0.003	0.046		1.13	-1.05	1.02	1.10	***Sptbn1***	spectrin beta, non-erythrocytic 1	0000281 // mitotic cytokinesis // not recorded///0007009 // plasma membrane organiza
NM_0012	0.0055			1.07	-1.04	1.02	1.05	***Foxj3***	forkhead box J3	0006351 // transcription, DNA-templated // inferred from electronic annotation///00063
NM_001038608///NM_00			0.03	1.04	1.08	1.02	-1.04	***Txnl4a***	thioredoxin-like 4A	0000245 // spliceosomal complex assembly // not recorded///0000398 // mRNA splicing,
NM_0099	0.0137			-1.30	1.06	1.02	1.03	***Ccr1***	chemokine (C-C motif) receptor 1	0006816 // calcium ion transport // not recorded///0006874 // cellular calcium ion home
NM_0012	0.0019			1.11	1.73	1.02	-1.01	***Fcer2a***	Fc receptor, IgE, low affinity II, alpha polypeptide	0002925 // positive regulation of humoral immune response mediated by circulating imm
NM_1341	0.013			1.09	1.35	1.02	1.25	***Gpsm3***	G-protein signalling modulator 3 (AGS3-like, C. elegans)	0002690 // positive regulation of leukocyte chemotaxis // inferred from mutant phenotyp
NM_001290817///		0.047		-1.19	-1.15	1.02	1.00	***Sparc***	secreted acidic cysteine rich glycoprotein	0001503 // ossification // inferred from electronic annotation///0001937 // negative regu
NM_0010	0.0148			1.03	-1.12	1.02	1.18	***Hnrnpul2***	heterogeneous nuclear ribonucleoprotein U-like 2	
NM_023732///XM		0.012		-1.04	1.13	1.02	-1.01	***Abcb6***	ATP-binding cassette, sub-family B (MDR/TAP), member 6	0006779 // porphyrin-containing compound biosynthetic process // not recorded///0006
NM_0298	0.0161			-1.07	-1.28	1.02	1.17	***Chmp5***	charged multivesicular body protein 5	0000920 // cell separation after cytokinesis // not recorded///0001919 // regulation of re
NM_0010	0.0223		0.017	1.01	-1.13	1.02	-1.20	***Glyctk***	glycerate kinase	0006468 // protein phosphorylation // not recorded///0016310 // phosphorylation // inf
NM_172710///XM_01124			0.017	-1.14	1.07	1.02	1.07	***Sel1l3***	sel-1 suppressor of lin-12-like 3 (C. elegans)	
NM_0013	0.0039			1.14	2.54	1.02	-1.02	***Cd79b***	CD79B antigen	0002250 // adaptive immune response // inferred from electronic annotation///0002376
NM_001304773///NM_00			0.03	-1.04	-1.13	1.02	1.07	***Pex26***	peroxisomal biogenesis factor 26	0006810 // transport // inferred from electronic annotation///0015031 // protein transp
NM_011295///XR_		0.016		1.02	1.15	1.02	-1.00	***Rps12///Rps12-ps10///Rps12-ps11///Rps1***	ribosomal protein S12///ribosomal protein S12, pseudogene 10///ribosomal protein S	0006412 // translation // inferred from electronic annotation
NM_0287	######			1.05	-1.05	1.02	-1.03	***Gm10651///Josd1***	predicted pseudogene 10651///Josephin domain containing 1	0006508 // proteolysis // inferred from electronic annotation
NM_0261	0.0329			1.12	-1.22	1.02	-1.22	***Dedd2***	death effector domain-containing DNA binding protein 2	0006351 // transcription, DNA-templated // inferred from electronic annotation///00063
NM_0263	0.0368			-1.15	1.25	1.02	-1.18	***Cdk2ap2***	CDK2-associated protein 2	
NM_0010	0.011			1.11	-1.01	1.02	1.17	***Arid1a***	AT rich interactive domain 1A (SWI-like)	0000122 // negative regulation of transcription from RNA polymerase II promoter // infer
NM_0294	0.0458			1.05	1.26	1.02	-1.08	***Ms4a4c***	membrane-spanning 4-domains, subfamily A, member 4C	
NM_0200	0.0109			1.04	1.29	1.02	1.10	***Tacstd2***	tumor-associated calcium signal transducer 2	0010633 // negative regulation of epithelial cell migration // inferred from direct assay///
NM_001286005///NM_02			0.019	1.03	-1.03	1.02	-1.15	***Abcg8***	ATP-binding cassette, sub-family G (WHITE), member 8	0006810 // transport // inferred from electronic annotation///0007584 // response to nu
NM_0010	0.0014			1.02	1.28	1.02	-1.18	***Akna***	AT-hook transcription factor	0006351 // transcription, DNA-templated // inferred from electronic annotation///00063
NM_0011	0.0099			1.14	-1.03	1.02	1.08	***Cntn1***	contactin 1	0007155 // cell adhesion // inferred from electronic annotation///0007219 // Notch signa
NM_0194	0.0296			1.15	1.01	1.02	-1.03	***Hes6***	hairy and enhancer of split 6	0000122 // negative regulation of transcription from RNA polymerase II promoter // infer
NM_024465///XM_00650			0.043	1.12	1.16	1.02	1.01	***Abhd12***	abhydrolase domain containing 12	0006660 // phosphatidylserine catabolic process // inferred from mutant phenotype///00
NM_0011	0.0028			1.07	-1.06	1.02	1.04	***Senp3***	SUMO/sentrin specific peptidase 3	0006508 // proteolysis // inferred from electronic annotation///0016926 // protein desu
NM_001289848///NM_00			0.034	-1.01	1.11	1.02	1.15	***Cox6b2***	cytochrome c oxidase subunit VIb polypeptide 2	1902600 // hydrogen ion transmembrane transport // inferred from electronic annotatio
NM_0010	0.0088			-1.04	1.23	1.02	-1.05	***Ptpn6***	protein tyrosine phosphatase, non-receptor type 6	0002244 // hematopoietic progenitor cell differentiation // inferred from mutant phenoty
NM_0074	######			1.01	1.24	1.02	1.14	***Rplp0***	ribosomal protein, large, P0	0042254 // ribosome biogenesis // inferred from electronic annotation///0071353 // cell
NM_018779///XM_00650			0.02	1.03	-1.10	1.02	-1.20	***Pde3a***	phosphodiesterase 3A, cGMP inhibited	0001556 // oocyte maturation // inferred from direct assay///0006198 // cAMP catabolic
NM_0011	0.0123			-1.04	1.11	1.02	-1.07	***LOC102641289///Tbc1d14***	TBC1 domain family member 14///TBC1 domain family, member 14	0010507 // negative regulation of autophagy // not recorded///0043547 // positive regul
NM_0119	0.0495			-1.02	1.20	1.02	1.11	***Nubp2***	nucleotide binding protein 2	0016226 // iron-sulfur cluster assembly // inferred from electronic annotation///003003
NM_0010	0.0313			1.06	-1.15	1.02	1.06	***Phactr1***	phosphatase and actin regulator 1	0031032 // actomyosin structure organization // inferred from mutant phenotype///003
NM_0012	0.0428			1.15	-1.04	1.02	1.08	***Wdr13***	WD repeat domain 13	
NM_0256	0.0273	0.008		1.08	1.03	1.03	1.16	***Rnf181***	ring finger protein 181	0000209 // protein polyubiquitination // ---///0016567 // protein ubiquitination // inferre
NM_177638///XM_00652			0.027	1.11	1.08	1.03	-1.09	***Crb3***	crumbs family member 3	0045198 // establishment of epithelial cell apical/basal polarity // not recorded///004521
NM_1447	0.0074			1.11	1.39	1.03	-1.13	***Metrnl***	meteorin, glial cell differentiation regulator-like	0009409 // response to cold // inferred from direct assay///0014850 // response to musc
NM_033324///XM		0.032		-1.06	1.10	1.03	-1.05	***Dgcr8***	DiGeorge syndrome critical region gene 8	0031053 // primary miRNA processing // not recorded///0072091 // regulation of stem c
NM_012017///XM		0.003		1.20	1.11	1.03	1.11	***Zfp346***	zinc finger protein 346	0043065 // positive regulation of apoptotic process // inferred from mutant phenotype//
NM_0242	0.0389			1.00	1.10	1.03	1.04	***Ipo4///Tm9sf1***	importin 4///transmembrane 9 superfamily member 1	0000059 // protein import into nucleus, docking // not recorded///0000060 // protein im
NM_001277265///NM_00			0.004	1.13	1.03	1.03	-1.18	***Tbxa2r***	thromboxane A2 receptor	0006954 // inflammatory response // inferred from mutant phenotype///0007165 // sign
NM_0011	0.0291			-1.03	-1.17	1.03	1.03	***Bub1***	budding uninhibited by benzimidazoles 1 homolog (S. cerevisiae)	0006468 // protein phosphorylation // inferred from electronic annotation///0006915 //
NM_0091	0.0346	0.036		1.06	1.00	1.03	1.21	***Sepw1***	selenoprotein W, muscle 1	0045454 // cell redox homeostasis // inferred from electronic annotation///0055114 // o
NM_0087	0.0074			1.02	-1.19	1.03	1.00	***Notch1***	notch 1	0000122 // negative regulation of transcription from RNA polymerase II promoter // infer
NM_0137	0.0122			-1.18	1.65	1.03	-1.23	***Cd52***	CD52 antigen	
NM_0011	0.011			1.14	1.38	1.03	1.02	***Myc***	myelocytomatosis oncogene	0000122 // negative regulation of transcription from RNA polymerase II promoter // infer
NM_145134			0.007	1.03	1.03	1.03	-1.17	***Spsb4***	splA/ryanodine receptor domain and SOCS box containing 4	0016567 // protein ubiquitination // inferred from electronic annotation///0035556 // in
NM_0113	0.0273			1.08	-1.08	1.03	1.16	***Scnn1g***	sodium channel, nonvoltage-gated 1 gamma	0006810 // transport // inferred from electronic annotation///0006811 // ion transport /
NM_0010	0.0046			-1.08	1.58	1.03	1.00	***Evi2a***	ecotropic viral integration site 2a	
NM_0260	######			-1.06	1.12	1.03	-1.01	***Rrp15***	ribosomal RNA processing 15 homolog (S. cerevisiae)	
NM_001128609///		0.044		-1.01	1.01	1.03	1.04	***Dedd***	death effector domain-containing	0006351 // transcription, DNA-templated // inferred from electronic annotation///00063
NM_0013	0.0304			-1.10	1.10	1.03	1.23	***Rab5c***	RAB5C, member RAS oncogene family	0006810 // transport // inferred from electronic annotation///0006886 // intracellular pr
NM_0010	######			1.12	-1.03	1.03	1.34	***Rere***	arginine glutamic acid dipeptide (RE) repeats	0000122 // negative regulation of transcription from RNA polymerase II promoter // infer
NM_009201///XM		0.048		1.24	1.24	1.03	1.09	***Slc1a5***	solute carrier family 1 (neutral amino acid transporter), member 5	0003333 // amino acid transmembrane transport // not recorded///0006810 // transpor
NM_1769	0.0166			-1.01	-1.08	1.03	1.00	***Galm***	galactose mutarotase	0005975 // carbohydrate metabolic process // not recorded///0006006 // glucose metab
NM_0012	0.0046			-1.03	1.23	1.03	1.01	***Sulf2***	sulfatase 2	0001822 // kidney development // inferred from genetic interaction///0002063 // chond
NM_0011	0.0456			-1.07	-1.34	1.03	1.01	***Atp7a***	ATPase, Cu++ transporting, alpha polypeptide	0001568 // blood vessel development // inferred from mutant phenotype///0001701 // i
NM_027898///XM_00654			0.024	1.03	1.00	1.03	-1.28	***Gramd1a***	GRAM domain containing 1A	
NM_054094///XM		0.046		-1.17	-1.08	1.03	1.10	***Acsm1***	acyl-CoA synthetase medium-chain family member 1	0006629 // lipid metabolic process // inferred from electronic annotation///0006631 // fa
NM_0010	0.0261			1.11	-1.10	1.03	-1.06	***Arl4a***	ADP-ribosylation factor-like 4A	0007264 // small GTPase mediated signal transduction // inferred from electronic annota
NM_011657///XM_00650			0.009	-1.04	-1.02	1.03	1.08	***Tulp3***	tubby-like protein 3	0001841 // neural tube formation // inferred from mutant phenotype///0001843 // neur
NM_0101	0.0018			1.08	-1.20	1.03	-1.11	***Efnb1***	ephrin B1	0001755 // neural crest cell migration // inferred from mutant phenotype///0007275 // r
NM_008172///XM_00654			0.007	1.04	1.04	1.03	-1.24	***Grin2d***	glutamate receptor, ionotropic, NMDA2D (epsilon 4)	0001964 // startle response // inferred from mutant phenotype///0006810 // transport /

RefSeq Tr	p 24h	p 48h	p 72h	FD Air	FD 24h	FD 48h	FD 72h	Gene Symbol	Gene Title	Gene Ontology Biological Process
NM_0012	0.016			-1.03	1.11	1.03	1.05	***Pex5***	peroxisomal biogenesis factor 5	0000038 // very long-chain fatty acid metabolic process // inferred from mutant phenoty
NM_0083	0.0456			-1.01	1.11	1.03	-1.33	***Il3ra***	interleukin 3 receptor, alpha chain	0019221 // cytokine-mediated signaling pathway // inferred from electronic annotation
NM_198604///XM_00650			0.017	1.03	-1.01	1.03	-1.12	***Plekhg6***	pleckstrin homology domain containing, family G (with RhoGef domain) member 6	0035023 // regulation of Rho protein signal transduction // inferred from electronic anno
NM_0333	0.0343			1.00	1.39	1.03	-1.01	***Dock2***	dedicator of cyto-kinesis 2	0001766 // membrane raft polarization // inferred from mutant phenotype///0001768 /
NM_0010	0.0341			1.04	-1.10	1.03	1.16	***Tulp4***	tubby like protein 4	0016567 // protein ubiquitination // inferred from electronic annotation///0035556 // in
NM_0265	0.0358			1.08	-1.06	1.03	-1.15	***Cog8///Pdf***	component of oligomeric golgi complex 8///peptide deformylase (mitochondrial)	0006412 // translation // inferred from electronic annotation///0006810 // transport // i
NM_0011	0.0084		0.042	-1.03	1.15	1.03	1.15	***Lonp2***	lon peptidase 2, peroxisomal	0006508 // proteolysis // inferred from electronic annotation///0006515 // misfolded or
NM_029619///XM_00649			0.013	1.01	1.06	1.03	-1.21	***Msrb2***	methionine sulfoxide reductase B2	0006979 // response to oxidative stress // inferred from electronic annotation///003004
NM_0086	0.0307			1.02	1.23	1.03	1.07	***Mmp14***	matrix metallopeptidase 14 (membrane-inserted)	0001503 // ossification // inferred from genetic interaction///0001525 // angiogenesis //
NM_001190449///NM_01			0.007	1.10	1.12	1.03	-1.03	***Ddah2***	dimethylarginine dimethylaminohydrolase 2	0000052 // citrulline metabolic process // not recorded///0006525 // arginine metabolic
NM_001163754///		0.015	0.045	-1.08	-1.04	1.03	1.08	***Rab3gap2***	RAB3 GTPase activating protein subunit 2	0043087 // regulation of GTPase activity // not recorded///0043547 // positive regulation
NM_0098	0.0018			-1.02	-1.10	1.03	1.09	***Ctnna1***	catenin (cadherin associated protein), alpha 1	0001541 // ovarian follicle development // inferred from electronic annotation///00070
NM_0197	0.0031			-1.00	1.08	1.03	-1.07	***Ikbke***	inhibitor of kappaB kinase epsilon	0006468 // protein phosphorylation // not recorded///0006974 // cellular response to D
NM_0805	0.0197			1.06	-1.25	1.03	1.05	***Ssfa2***	sperm specific antigen 2	
NM_0012	0.0172			1.11	-1.06	1.03	1.14	***Gtf2h1***	general transcription factor II H, polypeptide 1	0006281 // DNA repair // inferred from electronic annotation///0006289 // nucleotide-e
NM_0011	0.0462			-1.06	1.07	1.03	1.10	***Ptprj///Gm38718///Gm39869***	protein tyrosine phosphatase, receptor type, J///predicted gene, 38718///predicted ge	0001570 // vasculogenesis // inferred from mutant phenotype///0001954 // positive reg
NM_0011	0.0349			-1.01	1.39	1.03	1.05	***Evl***	Ena-vasodilator stimulated phosphoprotein	0007411 // axon guidance // non-traceable author statement///0008154 // actin polyme
NM_0011	0.027			-1.08	1.07	1.03	1.09	***Snx10***	sorting nexin 10	0006810 // transport // inferred from electronic annotation///0006897 // endocytosis //
NM_0257	0.0094			-1.13	1.12	1.03	1.13	***Pgm1***	phosphoglucomutase 1	0005975 // carbohydrate metabolic process // inferred from electronic annotation///000
NM_011890			0.023	-1.00	-1.05	1.03	1.18	***Sgcb***	sarcoglycan, beta (dystrophin-associated glycoprotein)	0007517 // muscle organ development // inferred from electronic annotation///004874
NM_0169	0.03		0.03	-1.14	1.30	1.03	1.36	***Gm8096///Phgdh***	3-phosphoglycerate dehydrogenase pseudogene///3-phosphoglycerate dehydrogenas	0006541 // glutamine metabolic process // inferred from mutant phenotype///0006544 /
NM_1534	0.0138			1.21	-1.06	1.03	1.26	***Dusp7***	dual specificity phosphatase 7	0000188 // inactivation of MAPK activity // not recorded///0006470 // protein dephosph
NM_0011	0.044			-1.03	-1.11	1.03	1.07	***Nucb1***	nucleobindin 1	0072718 // response to cisplatin // inferred from electronic annotation///1903533 // reg
NM_145610			0.035	1.14	1.16	1.03	-1.16	***Ppan***	peter pan homolog (Drosophila)	0000027 // ribosomal large subunit assembly // ---///0001560 // regulation of cell growt
NM_0263	0.035			1.12	-1.06	1.03	1.17	***Ahctf1***	AT hook containing transcription factor 1	0000910 // cytokinesis // not recorded///0006351 // transcription, DNA-templated // inf
NM_1454	0.027			-1.01	1.08	1.03	-1.04	***Prmt7***	protein arginine N-methyltransferase 7	0000387 // spliceosomal snRNP assembly // not recorded///0006349 // regulation of ger
NM_0280	0.0231			1.02	1.12	1.03	1.07	***Rpusd4***	RNA pseudouridylate synthase domain containing 4	0001522 // pseudouridine synthesis // inferred from electronic annotation///0009451 //
NM_001252218///		0.041		-1.09	1.02	1.03	-1.02	***LOC102641215///Rpl31///Rpl31-ps12///Rp***	60S ribosomal protein L31 pseudogene///ribosomal protein L31///ribosomal protein L	0002181 // cytoplasmic translation // ---///0006412 // translation // inferred from electr
NM_033270///NR_		0.041		-1.14	-1.16	1.03	-1.02	***E2f6***	E2F transcription factor 6	0000083 // regulation of transcription involved in G1/S transition of mitotic cell cycle // in
NM_0196	0.0115	0.014		-1.40	1.00	1.03	-1.21	***Cpxm1***	carboxypeptidase X 1 (M14 family)	0006508 // proteolysis // inferred from direct assay///0006518 // peptide metabolic pro
NM_0115	0.0444			-1.02	1.12	1.03	1.04	***Gm9855///Tdg///Tdg-ps***	thymine DNA glycosylase pseudogene///thymine DNA glycosylase///thymine DNA glyc	0000122 // negative regulation of transcription from RNA polymerase II promoter // infe
NM_001289782///		0.003	0.026	-1.26	-1.25	1.03	-1.00	***Cryab***	crystallin, alpha B	0001666 // response to hypoxia // inferred from mutant phenotype///0002088 // lens de
NM_010809		0.012	0.027	-1.56	-1.37	1.03	-1.19	***Mmp3***	matrix metallopeptidase 3	0006508 // proteolysis // inferred from mutant phenotype///0006508 // proteolysis // no
NM_0094	0.0162			1.16	-1.46	1.03	1.16	***Top1***	topoisomerase (DNA) I	0006260 // DNA replication // inferred from mutant phenotype///0006265 // DNA topolo
NM_0013	0.0418			-1.03	1.18	1.03	1.06	***Ube2v1***	ubiquitin-conjugating enzyme E2 variant 1	0006301 // postreplication repair // ---///0042275 // error-free postreplication DNA repa
NM_009517///XM_		0.047	0.03	-1.07	-1.02	1.03	1.07	***Zmat3***	zinc finger matrin type 3	0006810 // transport // inferred from electronic annotation///0006915 // apoptotic proc
NM_0256	0.0021			1.08	-1.06	1.03	1.01	***Mad2l1bp***	MAD2L1 binding protein	0007093 // mitotic cell cycle checkpoint // not recorded///0007094 // mitotic spindle ass
NM_207531///XM_		0.034	0.045	-1.03	-1.09	1.04	1.03	***Agr3***	anterior gradient 3	
NM_0091	######			-1.00	-1.14	1.04	1.04	***Sdf2***	stromal cell derived factor 2	0000032 // cell wall mannoprotein biosynthetic process // not recorded///0007275 // mu
NM_001005605///NM_00		0.047		-1.03	-1.07	1.04	1.07	***Aebp2***	AE binding protein 2	0000122 // negative regulation of transcription from RNA polymerase II promoter // infe
NM_0287	0.005			-1.01	1.26	1.04	1.17	***Gga2***	golgi associated, gamma adaptin ear containing, ARF binding protein 2	0006810 // transport // inferred from electronic annotation///0006886 // intracellular pr
NM_001159329///		0.038		-1.11	1.01	1.04	1.02	***Gtpbp8***	GTP-binding protein 8 (putative)	0000917 // barrier septum assembly // inferred from electronic annotation
NM_0010	0.0094			-1.02	1.43	1.04	-1.03	***Evi2b///Gm21975***	ecotropic viral integration site 2b///predicted gene 21975	
NM_001277932///		0.029		-1.02	1.07	1.04	-1.02	***Casp9***	caspase 9	0006508 // proteolysis // not recorded///0006508 // proteolysis // inferred from reviewe
NM_001291105///NM_00			0.021	-1.06	-1.10	1.04	1.05	***E2f1***	E2F transcription factor 1	0000077 // DNA damage checkpoint // not recorded///0000122 // negative regulation of
NM_013770			0.013	1.26	1.27	1.04	1.04	***Slc25a10***	solute carrier family 25 (mitochondrial carrier, dicarboxylate transporter), member 10	0006810 // transport // inferred from electronic annotation///0006817 // phosphate ion
NM_0213	0.0151		0.043	1.32	1.87	1.04	-1.07	***Tnfrsf13b***	tumor necrosis factor receptor superfamily, member 13b	0001782 // B cell homeostasis // inferred from direct assay///0001782 // B cell homeosta
NM_138668///XM_00650			0.009	-1.04	1.02	1.04	1.13	***Ufsp2***	UFM1-specific peptidase 2	0006508 // proteolysis // not recorded///0006508 // proteolysis // inferred from direct a
NM_0305	0.0208	0.05		-1.16	1.03	1.04	-1.06	***Slc19a3***	solute carrier family 19, member 3	0006810 // transport // inferred from electronic annotation///0015884 // folic acid trans
NM_001111274///		0.044		-1.32	-1.17	1.04	-1.11	***Igf1***	insulin-like growth factor 1	0001649 // osteoblast differentiation // inferred from expression pattern///0001775 // c
NM_1831	0.01			1.03	1.17	1.04	1.03	***Pyroxd1***	pyridine nucleotide-disulphide oxidoreductase domain 1	0055114 // oxidation-reduction process // inferred from electronic annotation
NM_001291104///		0.017		-1.07	-1.05	1.04	-1.08	***Fgf11***	fibroblast growth factor 11	
NM_0010	0.0475			-1.15	1.19	1.04	-1.06	***Clec5a***	C-type lectin domain family 5, member a	0002076 // osteoblast development // inferred from direct assay///0002376 // immune s
NM_0010	0.0477			-1.01	1.07	1.04	1.01	***Erbb2***	v-erb-b2 erythroblastic leukemia viral oncogene homolog 2, neuro/glioblastoma derive	0001889 // liver development // inferred from electronic annotation///0001934 // positi
NM_016680///NR_125832			0.026	1.00	1.04	1.04	-1.27	***Clasrp***	CLK4-associating serine/arginine rich protein	0006397 // mRNA processing // inferred from electronic annotation///0008380 // RNA sp
NM_001290658///NM_02			0.044	1.04	-1.03	1.04	-1.04	***Pomgnt1***	protein O-linked mannose beta 1,2-N-acetylglucosaminyltransferase	0006486 // protein glycosylation // inferred from electronic annotation///0006493 // pro
NM_0281	0.021			-1.15	1.02	1.04	1.05	***Tram1***	translocating chain-associating membrane protein 1	0006810 // transport // inferred from electronic annotation///0015031 // protein transp
NM_0270	0.0351			1.00	-1.10	1.04	1.09	***Sec62***	SEC62 homolog (S. cerevisiae)	0006620 // posttranslational protein targeting to membrane // not recorded///0006810 /
NM_001048206///NM_00			0.032	1.00	-1.01	1.04	1.10	***Traf3***	TNF receptor-associated factor 3	0001817 // regulation of cytokine production // inferred from mutant phenotype///0002
NM_001290430///NM_13			0.032	-1.04	1.10	1.04	1.11	***Ftsj1***	FtsJ homolog 1 (E. coli)	0001510 // RNA methylation // inferred from electronic annotation///0002128 // tRNA n
NM_001122822///		0.02		-1.02	1.01	1.04	1.02	***LOC105242468///Wrn***	Werner syndrome ATP-dependent helicase homolog///Werner syndrome homolog (hu	0000723 // telomere maintenance // inferred from genetic interaction///0000723 // telo
NM_1336	0.0166			1.05	-1.12	1.04	1.07	***Ces1e***	carboxylesterase 1E	0008152 // metabolic process // inferred from electronic annotation///0016042 // lipid c
NM_1332	0.0189			-1.08	1.15	1.04	-1.12	***Pilrb1***	paired immunoglobin-like type 2 receptor beta 1	0001773 // myeloid dendritic cell activation // inferred from direct assay///0007169 // tr
NM_0231	######			-1.05	-2.56	1.04	-1.18	***Klf15***	Kruppel-like factor 15	0006351 // transcription, DNA-templated // inferred from electronic annotation///0006

RefSeq Tr	*p* 24h	*p* 48h	*p* 72h	FD Air	FD 24h	FD 48h	FD 72h	Gene Symbol	Gene Title	Gene Ontology Biological Process
NM_1455	0.0471			1.04	-1.07	1.04	1.12	***Tiprl***	TIP41, TOR signalling pathway regulator-like (S. cerevisiae)	0000077 // DNA damage checkpoint // not recorded///0034048 // negative regulation of
NM_1785	0.0442			-1.05	1.10	1.04	-1.10	***Ptrh1***	peptidyl-tRNA hydrolase 1 homolog (S. cerevisiae)	0008152 // metabolic process // inferred from electronic annotation
NM_0013	0.017			1.03	1.17	1.04	-1.02	***Ints7***	integrator complex subunit 7	0000077 // DNA damage checkpoint // not recorded///0006974 // cellular response to D
NM_001033293///XR_374			0.039	1.05	1.31	1.04	-1.01	***Uap1l1***	UDP-N-acteylglucosamine pyrophosphorylase 1-like 1	0006048 // UDP-N-acetylglucosamine biosynthetic process // ---///0008152 // metabolic
NM_001033136///		0.047		-1.04	-1.15	1.04	1.03	***Rmdn3***	regulator of microtubule dynamics 3	0006874 // cellular calcium ion homeostasis // not recorded///0006915 // apoptotic proc
NM_001044720///NM_00			0.021	-1.17	-1.11	1.04	1.17	***Abcc9***	ATP-binding cassette, sub-family C (CFTR/MRP), member 9	0006810 // transport // inferred from electronic annotation///0006813 // potassium ion
NM_0011	0.0425			1.01	1.11	1.04	-1.11	***Emc6***	ER membrane protein complex subunit 6	0000045 // autophagosome assembly // not recorded///0034975 // protein folding in en
NM_201407///XM_006501			0.046	1.05	1.08	1.04	-1.10	***Dennd4b***	DENN/MADD domain containing 4B	0032483 // regulation of Rab protein signal transduction // not recorded///0043547 // p
NM_009431			0.043	1.12	1.06	1.04	-1.09	***Ctr9***	Ctr9, Paf1/RNA polymerase II complex component, homolog (S. cerevisiae)	0000122 // negative regulation of transcription from RNA polymerase II promoter // infer
NM_1341	0.0155	0.012		-1.11	1.04	1.04	-1.07	***Yars***	tyrosyl-tRNA synthetase	0006412 // translation // inferred from electronic annotation///0006418 // tRNA aminoa
NM_028431			0.015	-1.03	1.00	1.04	1.12	***Pmpcb***	peptidase (mitochondrial processing) beta	0006122 // mitochondrial electron transport, ubiquinol to cytochrome c // not recorded/
NM_178589		0.026		-1.09	-1.13	1.04	1.18	***Tnfrsf21***	tumor necrosis factor receptor superfamily, member 21	0001783 // B cell apoptotic process // inferred from mutant phenotype///0002250 // ada
NM_0010	######	#####		-1.19	1.35	1.04	-1.13	***Mcoln2***	mucolipin 2	0006810 // transport // inferred from electronic annotation///0006811 // ion transport /
NM_0137	0.0393			-1.14	1.05	1.04	-1.03	***Nbn***	nibrin	0000077 // DNA damage checkpoint // not recorded///0000723 // telomere maintenanc
NM_0010	0.0456			1.01	-1.02	1.04	1.17	***Rap1gds1***	RAP1, GTP-GDP dissociation stimulator 1	0007264 // small GTPase mediated signal transduction // not recorded///0014829 // vasc
NM_0013	0.0316			1.21	-1.03	1.04	1.24	***Porcn***	porcupine homolog (Drosophila)	0006497 // protein lipidation // inferred from direct assay///0009100 // glycoprotein me
NM_025834///XM		0.024		-1.13	1.02	1.04	-1.14	***Proz***	protein Z, vitamin K-dependent plasma glycoprotein	0006508 // proteolysis // inferred from electronic annotation///0007596 // blood coagul
NM_1459	0.0495		0.025	-1.09	1.00	1.04	1.10	***Alg3***	asparagine-linked glycosylation 3 (alpha-1,3-mannosyltransferase)	0006486 // protein glycosylation // inferred from electronic annotation///0097502 // ma
NM_009261///NM		0.003		-1.12	1.01	1.04	-1.00	***Strbp***	spermatid perinuclear RNA binding protein	0006928 // movement of cell or subcellular component // inferred from mutant phenoty
NM_0012	0.0263			1.02	1.11	1.04	1.02	***Rad51d***	RAD51 homolog D	0000707 // meiotic DNA recombinase assembly // not recorded///0000723 // telomere m
NM_001290495///NM_00			0.018	1.04	1.13	1.04	1.12	***Trub2***	TruB pseudouridine (psi) synthase homolog 2 (E. coli)	0001522 // pseudouridine synthesis // inferred from electronic annotation///0006396 //
NM_0197	0.0058			-1.13	-1.63	1.04	1.09	***Foxo3***	forkhead box O3	0000122 // negative regulation of transcription from RNA polymerase II promoter // not r
NM_0091	0.0333		0.039	1.07	-1.04	1.04	1.16	***Slc1a1***	solute carrier family 1 (neuronal/epithelial high affinity glutamate transporter, system	0006810 // transport // inferred from electronic annotation///0006835 // dicarboxylic ac
NM_0198	0.0237			-1.04	-1.25	1.04	-1.02	***Ube2g2***	ubiquitin-conjugating enzyme E2G 2	0000209 // protein polyubiquitination // not recorded///0016567 // protein ubiquitinatio
NM_030153///XM_006517			0.021	-1.01	-1.12	1.04	1.14	***Naa35***	N(alpha)-acetyltransferase 35, NatC auxiliary subunit	0043066 // negative regulation of apoptotic process // not recorded///0048659 // smoot
NM_001134465///		0.043		-1.02	-1.05	1.04	1.01	***Dennd6a***	DENN/MADD domain containing 6A	0043547 // positive regulation of GTPase activity // not recorded///2000049 // positive re
NM_0268	0.0251	0.04		-1.05	1.10	1.04	-1.06	***Dus1l***	dihydrouridine synthase 1-like (S. cerevisiae)	0002943 // tRNA dihydrouridine synthesis // not recorded///0008033 // tRNA processing
NM_001038625///		0.018	0.035	1.23	1.26	1.04	1.08	***Sertad2***	SERTA domain containing 2	0006351 // transcription, DNA-templated // inferred from electronic annotation///00063
NM_138589///XM		0.025		-1.07	-1.12	1.04	-1.02	***Ubfd1***	ubiquitin family domain containing 1	
NM_0012	0.0267			-1.05	1.11	1.04	-1.06	***Ndufs8***	NADH dehydrogenase (ubiquinone) Fe-S protein 8	0006810 // transport // inferred from electronic annotation///0006979 // response to ox
NM_146247///XM_006522			0.007	1.03	1.06	1.04	-1.19	***Mettl22***	methyltransferase like 22	0032259 // methylation // inferred from electronic annotation
NM_0257	0.0113			-1.17	1.00	1.04	-1.07	***Acot13***	acyl-CoA thioesterase 13	0008152 // metabolic process // inferred from electronic annotation///0051289 // protei
NM_0012	0.0033		0.034	1.09	-1.04	1.04	-1.03	***Dpf2***	D4, zinc and double PHD fingers family 2	0006351 // transcription, DNA-templated // inferred from electronic annotation///00063
NM_020594///XM		0.023		-1.07	-1.02	1.04	-1.02	***Zc3h8***	zinc finger CCCH type containing 8	0000122 // negative regulation of transcription from RNA polymerase II promoter // infer
NM_001310837///NM_01			0.022	1.03	-1.11	1.04	1.21	***Sspn***	sarcospan	
NM_011914			0.039	1.01	1.05	1.04	-1.14	***Nelfa***	negative elongation factor complex member A, Whsc2	0006351 // transcription, DNA-templated // inferred from electronic annotation///00063
NM_0010	0.0187			1.01	-1.05	1.04	1.00	***Rtn3***	reticulon 3	0006810 // transport // inferred from electronic annotation///0006915 // apoptotic proc
NM_0080	0.0252			1.05	1.23	1.05	1.14	***G6pdx///G6pd2***	glucose-6-phosphate dehydrogenase X-linked///glucose-6-phosphate dehydrogenase	0001816 // cytokine production // inferred from mutant phenotype///0001998 // angiote
NM_1340	0.0462			1.08	-1.07	1.05	1.13	***Tmed4***	transmembrane emp24 protein transport domain containing 4	0006810 // transport // inferred from electronic annotation///0007165 // signal transdu
NM_0114	0.0497			-1.12	1.04	1.05	-1.16	***Strap***	serine/threonine kinase receptor associated protein	0000122 // negative regulation of transcription from RNA polymerase II promoter // infer
NM_0012	0.0378			1.08	-1.19	1.05	1.38	***Cldnd1***	claudin domain containing 1	
NM_145460///XR_		0.006		-1.08	-1.05	1.05	-1.08	***Oxnad1***	oxidoreductase NAD-binding domain containing 1	0055114 // oxidation-reduction process // inferred from electronic annotation
NM_001198823///NM_00			0.018	1.01	1.03	1.05	1.14	***App***	amyloid beta (A4) precursor protein	0001878 // response to yeast // not recorded///0001967 // suckling behavior // inferred
NM_133223			0.026	1.18	1.40	1.05	1.04	***Rac3***	RAS-related C3 botulinum substrate 3	0007264 // small GTPase mediated signal transduction // inferred from electronic annota
NM_016918///XM		0.041		-1.03	-1.04	1.05	1.02	***Nudt5***	nudix (nucleoside diphosphate linked moiety X)-type motif 5	0006139 // nucleobase-containing compound metabolic process // inferred from direct a
NM_001123327///XM_006			0.003	-1.11	-1.10	1.05	1.11	***Qser1***	glutamine and serine rich 1	
NM_0168	0.0194			1.01	1.17	1.05	1.24	***Rab25***	RAB25, member RAS oncogene family	0003382 // epithelial cell morphogenesis // inferred from mutant phenotype///0006810
NM_025901		0.027		-1.10	-1.04	1.05	-1.07	***Polr3k***	polymerase (RNA) III (DNA directed) polypeptide K	0002376 // immune system process // inferred from electronic annotation///0006351 //
NM_1455	0.0291			1.02	1.14	1.05	1.05	***Rabepk***	Rab9 effector protein with kelch motifs	
NM_001099298///		0.016		-1.05	-1.04	1.05	-1.14	***Scn2a1***	sodium channel, voltage-gated, type II, alpha 1	0006810 // transport // inferred from electronic annotation///0006811 // ion transport /
NM_1342	0.011			-1.03	-1.32	1.05	1.19	***Elovl5***	ELOVL family member 5, elongation of long chain fatty acids (yeast)	0006629 // lipid metabolic process // inferred from electronic annotation///0006631 // f
NM_028394///XM		0.027	0.034	-1.22	-1.15	1.05	-1.04	***Hspb11***	heat shock protein family B (small), member 11	0001501 // skeletal system development // inferred from mutant phenotype///0006810 /
NM_0253	0.0323			-1.04	1.16	1.05	1.04	***Dnajc15***	DnaJ (Hsp40) homolog, subfamily C, member 15	0006810 // transport // inferred from electronic annotation///0009267 // cellular respon
NM_0013	0.0378			-1.06	1.07	1.05	1.00	***Cox16***	cytochrome c oxidase assembly protein 16	
NM_0012	0.032			1.07	1.25	1.05	1.19	***Slc10a3***	solute carrier family 10 (sodium/bile acid cotransporter family), member 3	0006810 // transport // inferred from electronic annotation///0010033 // response to or
NM_0120	0.0014			-1.04	1.13	1.05	-1.06	***Srp9***	signal recognition particle 9	0006614 // SRP-dependent cotranslational protein targeting to membrane // inferred fro
NM_0271	0.0037			1.03	-1.06	1.05	1.13	***Esam***	endothelial cell-specific adhesion molecule	0007155 // cell adhesion // inferred from electronic annotation///0007156 // homophilic
NM_0259	0.0343			1.08	1.25	1.05	1.03	***Efhd2***	EF hand domain containing 2	
NM_0265	0.0227			-1.13	1.06	1.05	-1.03	***LOC102642137///Rps13///Rps13-ps1///Rp***	40S ribosomal protein S13///ribosomal protein S13///ribosomal protein S13, pseudog	0006412 // translation // not recorded///0033119 // negative regulation of RNA splicing /
NM_0012	0.0435			1.05	1.35	1.05	1.06	***Sqstm1***	sequestosome 1	0001934 // positive regulation of protein phosphorylation // not recorded///0002376 // i
NM_0233	0.0489	0.048		-1.03	1.09	1.05	1.10	***Snap29***	synaptosomal-associated protein 29	0006810 // transport // inferred from electronic annotation///0006887 // exocytosis // n
NM_0115	0.0021			-1.09	1.10	1.05	1.18	***Surf4***	surfeit gene 4	0007030 // Golgi organization // not recorded///0010638 // positive regulation of organe
NM_0011	0.049			-1.08	1.12	1.05	1.01	***Naip2***	NLR family, apoptosis inhibitory protein 2	0002376 // immune system process // inferred from electronic annotation///0006915 //
NM_0011	0.0037			1.03	-1.12	1.05	1.01	***Cacul1***	CDK2 associated, cullin domain 1	0000082 // G1/S transition of mitotic cell cycle // not recorded///0006511 // ubiquitin-de

RefSeq Tr	p 24h	p 48h	p 72h	FD Air	FD 24h	FD 48h	FD 72h	Gene Symbol	Gene Title	Gene Ontology Biological Process
NM_0135	0.014			-1.01	-1.28	1.05	-1.20	***Mbd1***	methyl-CpG binding domain protein 1	0000122 // negative regulation of transcription from RNA polymerase II promoter // not
NM_009837		0.009		-1.09	-1.17	1.05	-1.14	***Cct4***	chaperonin containing Tcp1, subunit 4 (delta)	0006457 // protein folding // inferred from electronic annotation///0007339 // binding o
NM_0271	0.0316			1.05	1.24	1.05	1.09	***Nup37***	nucleoporin 37	0006810 // transport // inferred from electronic annotation///0007049 // cell cycle // inf
NM_145946///XM_		0.03		-1.06	-1.04	1.05	-1.04	***Fanci***	Fanconi anemia, complementation group I	0006281 // DNA repair // inferred from electronic annotation///0006974 // cellular respo
NM_0311	0.0214	0.041		-1.27	-1.56	1.05	-1.00	***Hspa8***	heat shock protein 8	0001916 // positive regulation of T cell mediated cytotoxicity // not recorded///0006351
NM_0011	0.0291		0.027	-1.02	1.08	1.05	1.14	***Igsf5***	immunoglobulin superfamily, member 5	0016337 // single organismal cell-cell adhesion // inferred from direct assay///0050767 //
NM_0115	0.0268	0.031		-1.28	1.04	1.05	-1.26	***Alyref///Alyref2***	Aly/REF export factor///Aly/REF export factor 2	0006397 // mRNA processing // inferred from electronic annotation///0006406 // mRNA
NM_0168	0.0162			1.15	-1.13	1.05	1.17	***Usp2***	ubiquitin specific peptidase 2	0000122 // negative regulation of transcription from RNA polymerase II promoter // not
NM_0011	0.0125	0.041		-1.09	1.09	1.05	-1.08	***Col18a1***	collagen, type XVIII, alpha 1	0001525 // angiogenesis // inferred from direct assay///0001525 // angiogenesis // infer
NM_011817		0.033		-1.34	-1.04	1.05	-1.74	***Gadd45g***	growth arrest and DNA-damage-inducible 45 gamma	0000185 // activation of MAPKKK activity // not recorded///0000186 // activation of MA
NM_027256///XM_00650			0.044	-1.06	1.02	1.05	1.09	***Ints4***	integrator complex subunit 4	0016180 // snRNA processing // not recorded
NM_0093	0.0424			-1.01	1.13	1.05	-1.05	***Tfam***	transcription factor A, mitochondrial	0006338 // chromatin remodeling // not recorded///0006351 // transcription, DNA-temp
NM_133435///XM_00653			0.028	-1.01	1.11	1.05	1.26	***Nmnat1***	nicotinamide nucleotide adenylyltransferase 1	0006457 // protein folding // non-traceable author statement///0006511 // ubiquitin-de
NM_175195///XM_00651			0.047	1.07	1.08	1.05	-1.14	***Sppl2b***	signal peptide peptidase like 2B	0006508 // proteolysis // inferred from electronic annotation///0006509 // membrane p
NM_1337	0.0259			1.08	1.19	1.05	1.03	***Dcp1a***	DCP1 decapping enzyme homolog A (S. cerevisiae)	0000184 // nuclear-transcribed mRNA catabolic process, nonsense-mediated decay // inf
NM_0307	0.0408			1.02	1.10	1.05	-1.02	***Uck2***	uridine-cytidine kinase 2	0006206 // pyrimidine nucleobase metabolic process // not recorded///0006222 // UMP
NM_0111	0.0136			-1.11	1.18	1.05	1.09	***Psmb5***	proteasome (prosome, macropain) subunit, beta type 5	0006508 // proteolysis // inferred from electronic annotation///0006979 // response to
NM_022980		#####		-1.17	-1.06	1.05	-1.05	***Rcan3***	regulator of calcineurin 3	0019722 // calcium-mediated signaling // inferred from electronic annotation///0050790
NM_008389///XM_00650			0.034	1.02	1.06	1.05	1.10	***Ipp***	IAP promoted placental gene	0016567 // protein ubiquitination // not recorded
NM_172739///XM_		0.026		1.17	1.12	1.05	1.24	***Arhgap35***	Rho GTPase activating protein 35	0000122 // negative regulation of transcription from RNA polymerase II promoter // not
NM_0110	0.0362			-1.17	1.26	1.05	-1.13	***Pfn1***	profilin 1	0001843 // neural tube closure // inferred from genetic interaction///0006357 // regulat
NM_0011	0.0048			1.05	1.38	1.05	1.23	***Arpc4***	actin related protein 2/3 complex, subunit 4	0030041 // actin filament polymerization // inferred from electronic annotation///00343
NM_001252519///		0.016	0.049	-1.10	-1.03	1.05	-1.05	***Zfp82***	zinc finger protein 82	0006351 // transcription, DNA-templated // inferred from electronic annotation///00063
NM_0080	0.0474	0.01		-1.06	1.10	1.05	1.09	***Fgf7***	fibroblast growth factor 7	0001541 // ovarian follicle development // not recorded///0002230 // positive regulation
NM_0111	0.0428			-1.02	1.22	1.05	1.31	***Psme1***	proteasome (prosome, macropain) activator subunit 1 (PA28 alpha)	0002479 // antigen processing and presentation of exogenous peptide antigen via MHC c
NM_0263	0.0493			-1.06	1.11	1.05	-1.01	***Tceb2///Gm9238***	transcription elongation factor B (SIII), polypeptide 2///transcription elongation factor	0006351 // transcription, DNA-templated // inferred from electronic annotation///00063
NM_0012	0.0227			1.02	-1.37	1.06	1.04	***Cadps2***	Ca2+-dependent activator protein for secretion 2	0006810 // transport // inferred from electronic annotation///0006887 // exocytosis // i
NM_001190258///NM_02			0.039	1.07	1.09	1.06	-1.01	***Rps27///Rps27rt***	ribosomal protein S27///ribosomal protein S27, retrogene	0006412 // translation // inferred from electronic annotation
NM_0255	0.0024	0.005		-1.15	-1.01	1.06	-1.16	***Coprs***	coordinator of PRMT5, differentiation stimulator	0006351 // transcription, DNA-templated // inferred from electronic annotation///00063
NM_0235	0.032			-1.05	1.15	1.06	1.02	***Pecr***	peroxisomal trans-2-enoyl-CoA reductase	0006629 // lipid metabolic process // inferred from electronic annotation///0006631 // f
NM_0010	0.0177			1.03	-1.24	1.06	1.10	***Cadm1***	cell adhesion molecule 1	0001889 // liver development // not recorded///0002376 // immune system process // i
NM_001293644///NM_02			0.026	-1.07	1.03	1.06	1.22	***Ccdc109b***	coiled-coil domain containing 109B	0006810 // transport // inferred from electronic annotation///0006811 // ion transport /
NM_010683			0.041	1.01	-1.06	1.06	1.12	***Lamc1***	laminin, gamma 1	0006461 // protein complex assembly // not recorded///0007155 // cell adhesion // not
NM_001034097///NM_00			0.026	1.04	1.14	1.06	1.27	***Tnfsf13///Tnfsfm13***	tumor necrosis factor (ligand) superfamily, member 13///tumor necrosis factor (ligand	0001525 // angiogenesis // inferred from electronic annotation///0002376 // immune sy
NM_0010	0.0224			-1.07	-1.36	1.06	1.09	***Dusp16***	dual specificity phosphatase 16	0000188 // inactivation of MAPK activity // inferred from direct assay///0006470 // prote
NM_0012	0.0425			1.04	-1.20	1.06	1.08	***Slit2***	slit homolog 2 (Drosophila)	0001656 // metanephros development // inferred from mutant phenotype///0001657 //
NM_015814///XM_		0.01		-1.24	-1.26	1.06	-1.05	***Dkk3***	dickkopf homolog 3 (Xenopus laevis)	0007275 // multicellular organismal development // inferred from electronic annotation/
NM_0257	0.0088			1.01	-1.10	1.06	1.08	***Cyb5a***	cytochrome b5 type A (microsomal)	0006631 // fatty acid metabolic process // inferred by curator///0006810 // transport //
NM_0157	0.0336			1.10	1.02	1.06	1.34	***Tmprss2***	transmembrane protease, serine 2	0006508 // proteolysis // inferred from direct assay///0006508 // proteolysis // not recor
NM_008579///XM_00649			0.049	-1.03	-1.23	1.06	1.19	***Meig1***	meiosis expressed gene 1	0007283 // spermatogenesis // inferred from electronic annotation///0030154 // cell diff
NM_0012	0.0448	0.046		-1.06	1.05	1.06	1.03	***Kif3a***	kinesin family member 3A	0001701 // in utero embryonic development // inferred from genetic interaction///0001
NM_0011	0.0242			1.06	-1.09	1.06	1.21	***Shoc2***	soc-2 (suppressor of clear) homolog (C. elegans)	0046579 // positive regulation of Ras protein signal transduction // not recorded
NM_0254	0.0115			-1.16	1.19	1.06	-1.20	***Rps4l***	ribosomal protein S4-like	0006412 // translation // ---///0006412 // translation // inferred from electronic annotat
NM_0102	0.0339			1.11	2.51	1.06	1.09	***Fos***	FBJ osteosarcoma oncogene	0001661 // conditioned taste aversion // inferred from electronic annotation///0006355
NM_008231///XM_		0.042		1.13	1.06	1.06	1.01	***Hdgf***	hepatoma-derived growth factor	0000122 // negative regulation of transcription from RNA polymerase II promoter // not
NM_1979	0.0018			1.18	-1.11	1.06	1.12	***Ces2g***	carboxylesterase 2G	0008152 // metabolic process // inferred from electronic annotation
NM_0252	0.0011			1.01	-1.09	1.06	1.11	***Atp6v0e***	ATPase, H+ transporting, lysosomal V0 subunit E	0006810 // transport // inferred from electronic annotation///0006811 // ion transport /
NM_001122683///		0.001	0.047	-1.07	-1.03	1.06	1.14	***Bdh1***	3-hydroxybutyrate dehydrogenase, type 1	0008152 // metabolic process // inferred from electronic annotation///0055114 // oxida
NM_0214	0.0068	0.018		-1.03	1.05	1.06	1.08	***Slc35b4***	solute carrier family 35, member B4	0006111 // regulation of gluconeogenesis // inferred from mutant phenotype///0006810
NM_0086	0.0202			-1.08	1.02	1.06	1.04	***Nedd1***	neural precursor cell expressed, developmentally down-regulated gene 1	0007019 // microtubule depolymerization // not recorded///0007049 // cell cycle // infe
NM_1723	0.0496			1.16	1.38	1.06	1.05	***Limd2***	LIM domain containing 2	
NM_0011	0.0297			1.08	-1.08	1.06	1.24	***Vps26a***	vacuolar protein sorting 26 homolog A (yeast)	0006810 // transport // inferred from electronic annotation///0006886 // intracellular pr
NM_0012	0.041			1.25	1.06	1.06	1.10	***Eps8l1***	EPS8-like 1	0007266 // Rho protein signal transduction // not recorded///0016601 // Rac protein sig
NM_008182///XM_00651			0.042	1.10	1.14	1.06	1.34	***Gsta2///Gm3776///Gsta1***	glutathione S-transferase, alpha 2 (Yc2)///predicted gene 3776///glutathione S-transf	0006749 // glutathione metabolic process // inferred from sequence or structural similari
NM_0011	0.0114		0.013	-1.01	1.10	1.06	1.08	***Tlk2***	tousled-like kinase 2 (Arabidopsis)	0001672 // regulation of chromatin assembly or disassembly // not recorded///0006468
NM_0013	0.0147			1.04	-1.03	1.06	1.14	***Rnf10***	ring finger protein 10	0006351 // transcription, DNA-templated // inferred from electronic annotation///00063
NM_0011	0.038			1.07	-1.07	1.06	1.03	***Rnmt***	RNA (guanine-7-) methyltransferase	0006370 // 7-methylguanosine mRNA capping // not recorded///0006397 // mRNA proc
NM_001290486///		0.007		-1.13	-1.21	1.06	-1.06	***Lpar1***	lysophosphatidic acid receptor 1	0000187 // activation of MAPK activity // inferred from direct assay///0007165 // signal t
NM_0090	0.0011			1.10	1.40	1.06	1.08	***Rasa3***	RAS p21 protein activator 3	0007165 // signal transduction // inferred from electronic annotation///0034605 // cellu
NM_0011	0.0061			1.02	-1.30	1.06	-1.04	***Snrk***	SNF related kinase	0006468 // protein phosphorylation // not recorded///0016310 // phosphorylation // inf
NM_145705///XM_		0.02		1.16	1.15	1.06	-1.01	***Tinf2***	Terf1 (TRF1)-interacting nuclear factor 2	0010833 // telomere maintenance via telomere lengthening // not recorded///0010836 /
NM_011163///XM_		0.011		-1.02	1.06	1.06	1.02	***Eif2ak2***	eukaryotic translation initiation factor 2-alpha kinase 2	0000186 // activation of MAPKK activity // not recorded///0001819 // positive regulation
NM_0296	0.0113			1.02	1.15	1.06	-1.03	***Srxn1***	sulfiredoxin 1 homolog (S. cerevisiae)	0006979 // response to oxidative stress // inferred from direct assay///0006979 // respo
NM_001081183///		0.038		-1.05	-1.00	1.06	1.05	***1110037F02Rik***	RIKEN cDNA 1110037F02 gene	0006397 // mRNA processing // inferred from electronic annotation///0008380 // RNA s

RefSeq Tr	*p* 24h	*p* 48h	*p* 72h	FD Air	FD 24h	FD 48h	FD 72h	Gene Symbol	Gene Title	Gene Ontology Biological Process
NM_177150///XM_00653			0.032	-1.05	1.03	1.06	1.12	***Cenpt***	centromere protein T	0000278 // mitotic cell cycle // not recorded///0007049 // cell cycle // inferred from elec
NM_001146100///NM_01			0.014	1.05	1.10	1.06	-1.03	***Hk1///Gm10362///Rpl17///Rpl17-ps5///R***	hexokinase 1///predicted gene 10362///ribosomal protein L17///ribosomal protein L1	0001678 // cellular glucose homeostasis // not recorded///0002931 // response to ischer
NM_0010	0.0256			1.02	-1.13	1.06	1.26	***C1s1***	complement component 1, s subcomponent 1	0002376 // immune system process // inferred from electronic annotation///0006508 //
NM_144543///XM		0.022		-1.02	1.02	1.06	1.02	***Thyn1***	thymocyte nuclear protein 1	
NM_0216	0.0051			1.11	1.01	1.06	1.10	***Agrn***	agrin	0001932 // regulation of protein phosphorylation // not recorded///0001934 // positive
NM_0010	0.0283			-1.01	1.13	1.06	1.08	***Mtmr7***	myotubularin related protein 7	0006470 // protein dephosphorylation // inferred from electronic annotation///0016311
NM_001201413///		0.02		-1.09	-1.15	1.06	-1.10	***Apbb2***	amyloid beta (A4) precursor protein-binding, family B, member 2	0001764 // neuron migration // inferred from genetic interaction///0006355 // regulatio
NM_0102	0.0274			1.06	-1.04	1.06	1.02	***Gdap2***	ganglioside-induced differentiation-associated-protein 2	0032526 // response to retinoic acid // inferred from direct assay
NM_0012	0.0478			-1.02	1.22	1.06	1.10	***Rgs19***	regulator of G-protein signaling 19	0009968 // negative regulation of signal transduction // inferred from electronic annotat
NM_145600			0.023	-1.04	1.03	1.06	1.13	***Zfp330***	zinc finger protein 330	
NM_0012	0.0207			-1.03	-1.21	1.06	1.15	***Spin1***	spindlin 1	0007049 // cell cycle // inferred from electronic annotation///0007143 // female meiotic
NM_0011	0.0146			1.03	-1.08	1.06	1.00	***Pdik1l***	PDLIM1 interacting kinase 1 like	0006468 // protein phosphorylation // inferred from electronic annotation///0016310 //
NM_0271	0.0041			1.07	1.41	1.06	1.06	***Dph1///Ovca2***	DPH1 homolog (S. cerevisiae)///candidate tumor suppressor in ovarian cancer 2	0008283 // cell proliferation // inferred from mutant phenotype///0017183 // peptidyl-d
NM_0187	0.0196			1.00	1.18	1.06	1.28	***Eif3d***	eukaryotic translation initiation factor 3, subunit D	0001731 // formation of translation preinitiation complex // inferred from electronic ann
NM_013691///XM_00650			0.022	-1.23	-1.13	1.06	1.05	***Thbs3***	thrombospondin 3	0003417 // growth plate cartilage development // inferred from genetic interaction///00
NM_001305273///		0.015		-1.03	1.02	1.06	1.02	***Rngtt***	RNA guanylyltransferase and 5'-phosphatase	0006370 // 7-methylguanosine mRNA capping // not recorded///0006396 // RNA proces
NM_0010	0.0135			1.11	-1.35	1.06	1.25	***Fam117b***	family with sequence similarity 117, member B	
NM_0263	0.0119			1.02	-1.06	1.06	-1.12	***Tprgl***	transformation related protein 63 regulated like	
NM_009252///XM_01124			0.03	-1.20	-1.02	1.06	1.25	***Serpina3n***	serine (or cysteine) peptidase inhibitor, clade A, member 3N	0006953 // acute-phase response // inferred from electronic annotation///0010466 // ne
NM_0084	0.0037			-1.16	1.52	1.06	-1.04	***Itgb2***	integrin beta 2	0002523 // leukocyte migration involved in inflammatory response // not recorded///00
NM_0012	0.0154			1.06	-1.09	1.06	1.03	***Trim2***	tripartite motif-containing 2	0016567 // protein ubiquitination // inferred from electronic annotation///0016567 // pr
NM_0011	0.0358			-1.09	1.05	1.06	1.15	***Srp54a///Srp54b///Srp54c***	signal recognition particle 54A///signal recognition particle 54B///signal recognition pa	0006614 // SRP-dependent cotranslational protein targeting to membrane // not recorde
NM_175313///XM_00650			0.034	-1.06	-1.04	1.07	1.15	***Eogt***	EGF domain-specific O-linked N-acetylglucosamine (GlcNAc) transferase	0006493 // protein O-linked glycosylation // inferred from direct assay///0008152 // met
NM_0094	0.0013	#####		-1.14	1.03	1.07	1.13	***Tuba3a///Tuba3b***	tubulin, alpha 3A///tubulin, alpha 3B	0007017 // microtubule-based process // inferred from electronic annotation///0008152
NM_001146174///		0.047	0.021	-1.09	-1.05	1.07	1.17	***Rangap1***	RAN GTPase activating protein 1	0007165 // signal transduction // inferred from electronic annotation///0043547 // posit
NM_0010	0.007			-1.07	-1.33	1.07	1.03	***Cstf3***	cleavage stimulation factor, 3' pre-RNA, subunit 3	0006379 // mRNA cleavage // not recorded///0006396 // RNA processing // inferred fror
NM_0116	0.0239			-1.00	1.24	1.07	-1.21	***Vars***	valyl-tRNA synthetase	0006412 // translation // inferred from electronic annotation///0006418 // tRNA aminoa
NM_0010	0.0058			-1.03	1.09	1.07	1.01	***Cklf***	chemokine-like factor	0006935 // chemotaxis // inferred from electronic annotation///0008283 // cell prolifera
NM_001042593///NM_00			0.021	-1.06	-1.12	1.07	1.03	***Hbs1l***	Hbs1-like (S. cerevisiae)	0006412 // translation // inferred from electronic annotation///0006414 // translational
NM_001277273///		0.048		-1.04	1.09	1.07	-1.08	***Fancl***	Fanconi anemia, complementation group L	0002230 // positive regulation of defense response to virus by host // not recorded///00
NM_016866///XM		0.021		-1.03	-1.05	1.07	1.03	***Stk39***	serine/threonine kinase 39	0001933 // negative regulation of protein phosphorylation // inferred from direct assay//
NM_0110	0.0011			1.26	-1.30	1.07	1.47	***Per2***	period circadian clock 2	0000122 // negative regulation of transcription from RNA polymerase II promoter // infer
NM_0103	0.0018			-1.08	1.29	1.07	1.10	***Gsr***	glutathione reductase	0006749 // glutathione metabolic process // inferred by curator///0006749 // glutathion
NM_001256516///		0.012		-1.04	-1.09	1.07	-1.01	***Zfp672***	zinc finger protein 672	0006351 // transcription, DNA-templated // inferred from electronic annotation///00063
NM_0010	0.0099			1.05	-1.06	1.07	1.00	***Akirin2***	akirin 2	0000122 // negative regulation of transcription from RNA polymerase II promoter // not
NM_0095	0.0409			-1.02	1.13	1.07	1.02	***Vhl***	von Hippel-Lindau tumor suppressor	0000122 // negative regulation of transcription from RNA polymerase II promoter // not
NM_025338///XM_00653			0.023	1.12	1.13	1.07	-1.03	***Aurkaip1***	aurora kinase A interacting protein 1	0016310 // phosphorylation // inferred from electronic annotation///0016567 // protein
NM_0157	0.047			1.10	-1.19	1.07	1.03	***Ttpa***	tocopherol (alpha) transfer protein	0001890 // placenta development // inferred from mutant phenotype///0001892 // emb
NM_0010	0.0036			1.03	1.34	1.07	-1.09	***Gmfg***	glia maturation factor, gamma	0034316 // negative regulation of Arp2/3 complex-mediated actin nucleation // inferred
NM_0011	0.0018	0.002		-1.00	1.14	1.07	1.09	***Nt5c2***	5'-nucleotidase, cytosolic II	0006470 // protein dephosphorylation // ---///0006470 // protein dephosphorylation // i
NM_1339	0.0247			-1.09	1.14	1.07	-1.06	***Emilin1***	elastin microfibril interfacer 1	0007155 // cell adhesion // inferred from electronic annotation///0010811 // positive re
NM_026177		0.017		-1.04	1.01	1.07	1.04	***Gpalpp1***	GPALPP motifs containing 1	
NM_0273	######			-1.00	1.21	1.07	1.05	***Gorasp2***	golgi reassembly stacking protein 2	0006996 // organelle organization // not recorded///0007030 // Golgi organization // no
NM_1339	0.0149			1.07	-1.16	1.07	1.12	***Mlxip***	MLX interacting protein	0006351 // transcription, DNA-templated // inferred from electronic annotation///00063
NM_0187	0.0384			1.03	1.22	1.07	-1.18	***Gipc1***	GIPC PDZ domain containing family, member 1	0006605 // protein targeting // inferred from mutant phenotype///0006605 // protein ta
NM_001288578///NM_00			0.031	-1.04	-1.11	1.07	1.09	***Adh5***	alcohol dehydrogenase 5 (class III), chi polypeptide	0001523 // retinoid metabolic process // inferred from mutant phenotype///0003016 //
NM_0011	0.0271			1.04	-1.21	1.07	1.13	***Gtf3c4***	general transcription factor IIIC, polypeptide 4	0006351 // transcription, DNA-templated // inferred from electronic annotation///00165
NM_001032413///NM_00			0.047	1.14	1.08	1.07	-1.03	***Pear1***	platelet endothelial aggregation receptor 1	0043654 // recognition of apoptotic cell // inferred from direct assay///0043654 // recog
NM_0307	0.0396	0.049		-1.10	1.11	1.07	-1.12	***Lactb***	lactamase, beta	0008152 // metabolic process // inferred from electronic annotation
NM_0288	######			-1.03	1.19	1.07	1.02	***Arpc5l***	actin related protein 2/3 complex, subunit 5-like	0030833 // regulation of actin filament polymerization // inferred from electronic annota
NM_0010	0.0207			-1.05	1.07	1.07	1.19	***Furin***	furin (paired basic amino acid cleaving enzyme)	0006465 // signal peptide processing // not recorded///0006508 // proteolysis // inferred
NM_0010	0.0015	0.002		-1.05	1.04	1.07	-1.04	***Ogfrl1***	opioid growth factor receptor-like 1	
NM_0010	0.0255			-1.06	1.17	1.07	1.21	***H3f3a-ps1///H3f3b///H3f3c***	H3 histone, family 3A///H3 histone, family 3A, pseudogene 1///H3 histone, family 3B/	0000183 // chromatin silencing at rDNA // traceable author statement///0001649 // oste
NM_010162			0.041	-1.09	-1.13	1.07	1.11	***Ext1***	exostoses (multiple) 1	0001503 // ossification // not recorded///0006024 // glycosaminoglycan biosynthetic pro
NM_0076	######			-1.05	-1.47	1.07	-1.18	***Cdr2***	cerebellar degeneration-related 2	
NM_1813	0.0163			1.03	-1.03	1.07	1.02	***Anapc13***	anaphase promoting complex subunit 13	0007049 // cell cycle // inferred from electronic annotation///0007067 // mitotic nuclear
NM_0213	0.0091			-1.07	1.09	1.07	-1.10	***Itgax***	integrin alpha X	0007155 // cell adhesion // inferred from electronic annotation///0007229 // integrin-m
NM_0012	0.0439	0.023		-1.01	1.15	1.07	-1.03	***Nfam1***	Nfat activating molecule with ITAM motif 1	0007165 // signal transduction // not recorded///0007165 // signal transduction // infer
NM_0119	0.0407			1.13	-1.00	1.07	1.21	***Mapk3***	mitogen-activated protein kinase 3	0000165 // MAPK cascade // not recorded///0000189 // MAPK import into nucleus // no
NM_1724	0.0147			1.10	-1.01	1.07	1.12	***Dnajc8***	DnaJ (Hsp40) homolog, subfamily C, member 8	
NM_025934///XM_00652			0.05	-1.03	1.00	1.07	1.22	***Riok2***	RIO kinase 2 (yeast)	0006468 // protein phosphorylation // inferred from electronic annotation///0016310 //
NM_177326///XM_00652			0.01	-1.03	-1.05	1.07	1.07	***Pak2***	p21 protein (Cdc42/Rac)-activated kinase 2	0006468 // protein phosphorylation // not recorded///0006915 // apoptotic process // i
NM_0330	######			1.05	1.19	1.07	-1.06	***Nudt19***	nudix (nucleoside diphosphate linked moiety X)-type motif 19	0008152 // metabolic process // inferred from electronic annotation
NM_029896///XM_01124			0.034	-1.08	-1.11	1.07	1.05	***Wdr82***	WD repeat domain containing 82	0051568 // histone H3-K4 methylation // inferred from mutant phenotype///0051568 //

RefSeq Tr	*p* 24h	*p* 48h	*p* 72h	FD Air	FD 24h	FD 48h	FD 72h	Gene Symbol	Gene Title	Gene Ontology Biological Process
NM_0205	0.0303			-1.04	1.15	1.07	1.16	***Nelfcd***	negative elongation factor complex member C/D, Th1l	0006351 // transcription, DNA-templated // inferred from electronic annotation///00063
NM_1774	0.0337			-1.05	1.06	1.08	1.09	***Pum3***	pumilio RNA-binding family member 3	0006417 // regulation of translation // not recorded
NM_0011	0.0385			1.16	1.33	1.08	1.10	***Pkn1***	protein kinase N1	0001782 // B cell homeostasis // inferred from mutant phenotype///0001783 // B cell ap
NM_0078	0.0077			1.14	-1.03	1.08	1.05	***Dsg2***	desmoglein 2	0002934 // desmosome organization // not recorded///0003165 // Purkinje myocyte dev
NM_0010	0.0486		0.034	-1.08	1.13	1.08	1.33	***Rrn3***	RRN3 RNA polymerase I transcription factor homolog (yeast)	0001701 // in utero embryonic development // inferred from mutant phenotype///0006
NM_1448	0.0174			1.09	-1.05	1.08	1.07	***Taok1***	TAO kinase 1	0000165 // MAPK cascade // ---///0000186 // activation of MAPKK activity // not recorde
NM_0115	0.0494			1.08	1.02	1.08	1.13	***Timp2***	tissue inhibitor of metalloproteinase 2	0007417 // central nervous system development // inferred from electronic annotation//
NM_1705	0.0451			-1.06	-1.26	1.08	1.01	***Nupl1***	nucleoporin like 1	0006810 // transport // inferred from electronic annotation///0006913 // nucleocytopla
NM_025334			0.044	-1.02	-1.02	1.08	1.17	***Txndc12***	thioredoxin domain containing 12 (endoplasmic reticulum)	0045454 // cell redox homeostasis // inferred from electronic annotation///0055114 // o
NM_0075	0.0327			1.02	1.11	1.08	1.03	***Atp6v1b2***	ATPase, H+ transporting, lysosomal V1 subunit B2	0006810 // transport // inferred from electronic annotation///0006811 // ion transport /
NM_0011	0.0223			-1.01	1.25	1.08	1.09	***Sirpa***	signal-regulatory protein alpha	0002244 // hematopoietic progenitor cell differentiation // inferred from mutant phenot
NM_0117	0.0042			1.03	1.46	1.08	1.13	***Zfp36***	zinc finger protein 36	0000122 // negative regulation of transcription from RNA polymerase II promoter // infe
NM_0268	0.0146	0.018		-1.02	-1.29	1.08	1.03	***Trmt1l***	tRNA methyltransferase 1 like	0002940 // tRNA N2-guanine methylation // ---///0007610 // behavior // inferred from e
NM_0012	0.0172			-1.01	1.18	1.08	1.23	***Tmem173***	transmembrane protein 173	0002218 // activation of innate immune response // inferred from mutant phenotype///
NM_001044386///NM_01			0.034	1.01	-1.15	1.08	1.20	***Zfx***	zinc finger protein X-linked	0001541 // ovarian follicle development // inferred from mutant phenotype///0006351 /
NM_0215	0.0131			-1.07	1.19	1.08	-1.01	***Rcl1***	RNA terminal phosphate cyclase-like 1	0000479 // endonucleolytic cleavage of tricistronic rRNA transcript (SSU-rRNA, 5.8S rRNA
NM_1386	0.0331			-1.05	1.09	1.08	1.02	***Mvd***	mevalonate (diphospho) decarboxylase	0006629 // lipid metabolic process // inferred from electronic annotation///0006694 // s
NM_172015///XM		0.033		-1.09	-1.05	1.08	1.02	***Iars***	isoleucine-tRNA synthetase	0001649 // osteoblast differentiation // not recorded///0006412 // translation // inferre
NM_0010	0.017			1.02	-1.09	1.08	1.05	***Osbp***	oxysterol binding protein	0006810 // transport // inferred from electronic annotation///0006869 // lipid transport
NM_0199	0.0242			-1.11	1.14	1.08	1.05	***Litaf///Gm9861***	LPS-induced TN factor///predicted gene 9861	0001817 // regulation of cytokine production // inferred from mutant phenotype///0006
NM_028680		0.013		-1.10	-1.07	1.08	-1.14	***Ift57***	intraflagellar transport 57	0001843 // neural tube closure // inferred from mutant phenotype///0001947 // heart l
NM_1750	0.0085			-1.01	1.10	1.08	1.20	***Commd2***	COMM domain containing 2	0006351 // transcription, DNA-templated // inferred from electronic annotation///00063
NM_0011	0.0262	0.02	0.023	-1.19	-1.02	1.08	-1.01	***Ccdc127***	coiled-coil domain containing 127	
NM_0084	0.0162			-1.05	1.14	1.08	-1.02	***Itgb1bp1***	integrin beta 1 binding protein 1	0001525 // angiogenesis // inferred from electronic annotation///0002043 // blood vess
NM_0013	0.0058			1.02	1.29	1.08	1.06	***Usp3***	ubiquitin specific peptidase 3	0000122 // negative regulation of transcription from RNA polymerase II promoter // not
NM_025692///XM		0.034		-1.07	1.01	1.08	-1.20	***Uba5***	ubiquitin-like modifier activating enzyme 5	0033146 // regulation of intracellular estrogen receptor signaling pathway // not recorde
NM_0098	0.0245			-1.04	-1.20	1.08	-1.07	***Cebpg***	CCAAT/enhancer binding protein (C/EBP), gamma	0001889 // liver development // inferred from sequence or structural similarity///00018
NM_001316372///		0.028		-1.04	1.06	1.08	1.07	***Pctp***	phosphatidylcholine transfer protein	0006810 // transport // inferred from electronic annotation///0006869 // lipid transport
NM_013717///XM		0.035		-1.01	1.03	1.08	1.02	***B9d1***	B9 protein domain 1	0001701 // in utero embryonic development // inferred from mutant phenotype///0001
NM_019951			0.037	-1.09	1.03	1.08	1.21	***Sec11a***	SEC11 homolog A, signal peptidase complex subunit	0006465 // signal peptide processing // inferred from electronic annotation///0006508 /
NM_1462	0.0327			-1.04	1.12	1.08	1.05	***Apeh***	acylpeptide hydrolase	0006508 // proteolysis // not recorded///0050435 // beta-amyloid metabolic process //
NM_001110310///		0.015		-1.04	1.03	1.08	1.06	***Snx12***	sorting nexin 12	0006810 // transport // inferred from electronic annotation///0006897 // endocytosis //
NM_0116	0.0116	0.043		-1.19	1.11	1.08	1.17	***Tubb5***	tubulin, beta 5 class I	0006457 // protein folding // traceable author statement///0007017 // microtubule-bas
NM_019791		0.012		-1.20	-1.04	1.08	1.12	***Maged1***	melanoma antigen, family D, 1	0006357 // regulation of transcription from RNA polymerase II promoter // inferred from
NM_001253757///NM_00			0.034	1.00	1.00	1.08	1.33	***Anp32e***	acidic (leucine-rich) nuclear phosphoprotein 32 family, member E	0016568 // chromatin modification // inferred from electronic annotation///0043086 //
NM_001164223///NM_02			0.045	1.06	1.01	1.08	-1.02	***Rpa1***	replication protein A1	0000723 // telomere maintenance // not recorded///0000724 // double-strand break re
NM_001289621///		0.015		-1.01	1.03	1.08	1.05	***Mynn***	myoneurin	0006351 // transcription, DNA-templated // inferred from electronic annotation///00063
NM_029735///XM		0.003		-1.09	-1.01	1.08	1.03	***Eprs***	glutamyl-prolyl-tRNA synthetase	0006412 // translation // inferred from electronic annotation///0006417 // regulation of
NM_0206	0.0259			1.05	1.15	1.08	1.00	***Srd5a3***	steroid 5 alpha-reductase 3	0006486 // protein glycosylation // inferred from electronic annotation///0006488 // dol
NM_0137	######	0.044		-1.13	1.39	1.08	1.16	***Msrb1***	methionine sulfoxide reductase B1	0002376 // immune system process // inferred from electronic annotation///0006979 //
NM_033526///XM		0.02		-1.05	-1.07	1.08	1.05	***Ubqln4***	ubiquilin 4	0006914 // autophagy // inferred from electronic annotation///0032434 // regulation of
NM_0011	0.0299			1.09	1.29	1.08	1.00	***Sin3b***	transcriptional regulator, SIN3B (yeast)	0000122 // negative regulation of transcription from RNA polymerase II promoter // not
NM_0117	0.0233			-1.06	1.12	1.08	1.04	***Trpv2***	transient receptor potential cation channel, subfamily V, member 2	0006810 // transport // inferred from electronic annotation///0006811 // ion transport /
NM_019743		0.024		-1.01	1.06	1.08	-1.03	***Rybp***	RING1 and YY1 binding protein	0000122 // negative regulation of transcription from RNA polymerase II promoter // infe
NM_0011	0.0292			1.01	1.16	1.08	-1.10	***Ifnar2***	interferon (alpha and beta) receptor 2	0006357 // regulation of transcription from RNA polymerase II promoter // inferred from
NM_001161775///NM_01			0.04	-1.21	-1.21	1.08	1.17	***Myh11***	myosin, heavy polypeptide 11, smooth muscle	0006939 // smooth muscle contraction // inferred from direct assay///0006939 // smoot
NM_009068///XM		0.033		-1.03	-1.01	1.08	-1.05	***Ripk1***	receptor (TNFRSF)-interacting serine-threonine kinase 1	0001934 // positive regulation of protein phosphorylation // not recorded///0006468 //
NM_1454	0.0221			1.00	-1.08	1.08	1.05	***Nipal2***	NIPA-like domain containing 2	0015693 // magnesium ion transport // inferred from electronic annotation///1903830 /
NM_0188	0.0061			-1.03	1.05	1.08	1.06	***Uqcc1***	ubiquinol-cytochrome c reductase complex assembly factor 1	0034551 // mitochondrial respiratory chain complex III assembly // not recorded///0070
NM_0229	0.0096			-1.05	1.09	1.08	1.09	***Nif3l1***	Ngg1 interacting factor 3-like 1 (S. pombe)	0030182 // neuron differentiation // inferred from mutant phenotype///0045893 // posi
NM_0167	0.0287			-1.13	1.18	1.09	-1.02	***Atp5b***	ATP synthase, H+ transporting mitochondrial F1 complex, beta subunit	0001525 // angiogenesis // not recorded///0001649 // osteoblast differentiation // not r
NM_0012	0.0067			1.00	1.45	1.09	-1.14	***Ncf1***	neutrophil cytosolic factor 1	0001878 // response to yeast // inferred from mutant phenotype///0001909 // leukocyt
NM_012055///XM		0.014		-1.19	-1.02	1.09	-1.03	***Asns***	asparagine synthetase	0001889 // liver development // inferred from electronic annotation///0006520 // cellul
NM_030109///XM		0.038		1.00	1.03	1.09	1.21	***Sf3b2***	splicing factor 3b, subunit 2	0000398 // mRNA splicing, via spliceosome // not recorded
NM_0224	0.0138			1.08	1.36	1.09	1.06	***Ptges***	prostaglandin E synthase	0001516 // prostaglandin biosynthetic process // not recorded///0002526 // acute inflan
NM_133900///XM_00650			0.023	-1.08	-1.01	1.09	1.09	***Psph***	phosphoserine phosphatase	0006470 // protein dephosphorylation // ---///0006470 // protein dephosphorylation // i
NM_001305284///		0.05		-1.18	-1.28	1.09	1.06	***Mpdz***	multiple PDZ domain protein	0007155 // cell adhesion // inferred from direct assay///0042552 // myelination // not re
NM_080850///XM		0.007		-1.06	-1.00	1.09	-1.16	***Pask***	PAS domain containing serine/threonine kinase	0006468 // protein phosphorylation // not recorded///0016310 // phosphorylation // inf
NM_0076	0.0021			1.13	2.89	1.09	1.11	***Cd79a***	CD79A antigen (immunoglobulin-associated alpha)	0002250 // adaptive immune response // inferred from electronic annotation///0002376
NM_011653		0.008		-1.14	-1.00	1.09	-1.01	***Tuba1a***	tubulin, alpha 1A	0007017 // microtubule-based process // inferred from electronic annotation///0008152
NM_001110512///NM_14			0.02	1.04	1.09	1.09	1.18	***Mettl8***	methyltransferase like 8	0007519 // skeletal muscle tissue development // inferred from direct assay///0016573 /
NM_0119	0.0232			1.07	-1.07	1.09	1.22	***Fto***	fat mass and obesity associated	0001659 // temperature homeostasis // inferred from mutant phenotype///0006281 //
NM_0082	0.0037			1.02	1.54	1.09	-1.30	***Hlx***	H2.0-like homeobox	0001889 // liver development // inferred from mutant phenotype///0006351 // transcri
NM_0265	0.0246			-1.03	1.18	1.09	1.10	***Emc4***	ER membrane protein complex subunit 4	0006915 // apoptotic process // inferred from electronic annotation

RefSeq Tr	p 24h	p 48h	p 72h	FD Air	FD 24h	FD 48h	FD 72h	Gene Symbol	Gene Title	Gene Ontology Biological Process
NM_1338	0.0074			-1.07	1.11	1.09	-1.11	***Dpp3***	dipeptidylpeptidase 3	0006508 // proteolysis // not recorded
NM_001290826///NM_00			0.012	-1.04	1.04	1.09	1.14	***Cpeb3***	cytoplasmic polyadenylation element binding protein 3	0000122 // negative regulation of transcription from RNA polymerase II promoter // not r
NM_0092	0.034	0.007		-1.08	1.05	1.09	-1.05	***Sos1***	son of sevenless homolog 1 (Drosophila)	0001782 // B cell homeostasis // inferred from genetic interaction///0001942 // hair folli
NM_0011	0.0452			-1.05	-1.15	1.09	1.04	***2-Sep***	septin 2	0000910 // cytokinesis // non-traceable author statement///0002036 // regulation of L-g
NM_001163687///		0.025		-1.10	-1.01	1.09	-1.08	***Naaa***	N-acylethanolamine acid amidase	0006629 // lipid metabolic process // inferred from electronic annotation
NM_0013	######			1.09	-1.01	1.09	1.02	***Actg1***	actin, gamma, cytoplasmic 1	0001895 // retina homeostasis // inferred from electronic annotation///0045214 // sarco
NM_019836///XM_		0.013		1.05	1.04	1.09	-1.06	***Noa1***	nitric oxide associated 1	0006915 // apoptotic process // inferred from electronic annotation///0010941 // regula
NM_0087	0.0186			1.08	1.38	1.09	1.06	***Pafah1b3***	platelet-activating factor acetylhydrolase, isoform 1b, subunit 3	0006629 // lipid metabolic process // inferred from electronic annotation///0007283 // s
NM_1451	0.0379			1.02	1.21	1.09	1.16	***Eif3l***	eukaryotic translation initiation factor 3, subunit L	0001731 // formation of translation preinitiation complex // inferred from electronic ann
NM_001110211///		0.011		-1.08	1.05	1.09	1.39	***Anxa6***	annexin A6	0006816 // calcium ion transport // inferred from mutant phenotype///0006937 // regula
NM_025516///XM_		0.035		-1.00	-1.04	1.09	1.24	***Ergic3***	ERGIC and golgi 3	0006810 // transport // inferred from electronic annotation///0006888 // ER to Golgi ves
NM_199199		0.019		-1.08	1.00	1.09	-1.02	***Tmem199***	transmembrane protein 199	
NM_001306205///NM_02			0.009	1.11	1.14	1.09	-1.16	***Mvk***	mevalonate kinase	0006629 // lipid metabolic process // inferred from electronic annotation///0006694 // s
NM_0104	0.0164			-1.10	1.39	1.09	1.26	***Id2***	inhibitor of DNA binding 2	0000122 // negative regulation of transcription from RNA polymerase II promoter // infer
NM_001291197///NM_00			0.049	-1.05	-1.02	1.09	1.13	***Tpd52l2***	tumor protein D52-like 2	
NM_0136	0.0338			1.02	1.20	1.09	-1.01	***Odc1///Gm9115***	ornithine decarboxylase, structural 1///ornithine decarboxylase, structural 1 pseudoge	0001822 // kidney development // inferred from mutant phenotype///0006595 // polyar
NM_011794///XM_		0.012	0.015	-1.21	-1.20	1.09	1.16	***Bpnt1***	bisphosphate 3'-nucleotidase 1	0006139 // nucleobase-containing compound metabolic process // not recorded///00064
NM_0173	0.0181			-1.02	1.15	1.09	-1.15	***Ostf1***	osteoclast stimulating factor 1	
NM_0011	0.0458			1.01	-1.15	1.09	1.06	***Zfp275***	zinc finger protein 275	0006355 // regulation of transcription, DNA-templated // inferred from electronic annota
NM_1308	######			-1.01	1.18	1.09	1.04	***Anp32b***	acidic (leucine-rich) nuclear phosphoprotein 32 family, member B	0000082 // G1/S transition of mitotic cell cycle // not recorded///0001944 // vasculature
NM_0013	0.0384			-1.04	1.13	1.09	-1.17	***Mbd3***	methyl-CpG binding domain protein 3	0000122 // negative regulation of transcription from RNA polymerase II promoter // infer
NM_009148///XM_		0.012	0.046	-1.09	-1.06	1.09	1.01	***Exoc4***	exocyst complex component 4	0006612 // protein targeting to membrane // not recorded///0006810 // transport // infe
NM_0294	0.0226			-1.06	-1.44	1.09	-1.05	***Slc10a6***	solute carrier family 10 (sodium/bile acid cotransporter family), member 6	0006810 // transport // inferred from electronic annotation///0006811 // ion transport /
NM_0242	0.0037	0.031	0.035	-1.04	1.10	1.09	1.05	***Anapc4***	anaphase promoting complex subunit 4	0007049 // cell cycle // inferred from electronic annotation///0007067 // mitotic nuclear
NM_144884		0.026		-1.09	1.02	1.09	-1.08	***Tor1a***	torsin family 1, member A (torsin A)	0000338 // protein deneddylation // not recorded///0006979 // response to oxidative str
NM_173865			0.002	1.01	1.07	1.09	1.33	***Slc41a1***	solute carrier family 41, member 1	0006810 // transport // inferred from electronic annotation///0006811 // ion transport /
NM_022011///XM_		0.03	0.011	-1.13	-1.15	1.09	1.10	***Gtf2h2***	general transcription factor II H, polypeptide 2	0002031 // G-protein coupled receptor internalization // not recorded///0006281 // DNA
NM_001136070///		0.036		1.02	1.08	1.10	1.09	***Eif2d***	eukaryotic translation initiation factor 2D	0001731 // formation of translation preinitiation complex // not recorded///0002192 // I
NM_0138	0.0076			-1.09	-1.58	1.10	1.01	***Bag3***	BCL2-associated athanogene 3	0006915 // apoptotic process // inferred from electronic annotation///0007420 // brain d
NM_026521		0.032		-1.05	-1.02	1.10	-1.02	***Zfp706***	zinc finger protein 706	0006417 // regulation of translation // inferred from electronic annotation///0045892 //
NM_0011	0.0327			1.03	-1.14	1.10	1.07	***Zfp790***	zinc finger protein 790	0006355 // regulation of transcription, DNA-templated // inferred from electronic annota
NM_009730///XM_006498			0.021	1.15	1.04	1.10	-1.10	***Atrn***	attractin	0006954 // inflammatory response // inferred from electronic annotation///0006979 // r
NM_001291358///NM_14			0.044	1.04	-1.01	1.10	1.31	***Pgap2***	post-GPI attachment to proteins 2	0006506 // GPI anchor biosynthetic process // not recorded///0006974 // cellular respon
NM_172592///XM_00651			0.046	-1.01	1.04	1.10	1.21	***Srek1***	splicing regulatory glutamine/lysine-rich protein 1	0006397 // mRNA processing // inferred from electronic annotation///0008380 // RNA sp
NM_001042491///NM_00			0.017	1.10	1.09	1.10	-1.02	***Anapc5***	anaphase-promoting complex subunit 5	0007049 // cell cycle // inferred from electronic annotation///0007067 // mitotic nuclear
NM_133238		0.022		-1.23	-1.14	1.10	-1.05	***Cd209a***	CD209a antigen	0006897 // endocytosis // inferred from electronic annotation///0010468 // regulation o
NM_0013	0.0126			-1.05	1.18	1.10	1.04	***Ppp1r1b***	protein phosphatase 1, regulatory (inhibitor) subunit 1B	0001975 // response to amphetamine // inferred from genetic interaction///0006351 // t
NM_0010	0.0353			-1.04	1.11	1.10	1.02	***Nasp***	nuclear autoantigenic sperm protein (histone-binding)	0001824 // blastocyst development // inferred from mutant phenotype///0006260 // DN
NM_001170332///		0.016		-1.04	1.01	1.10	-1.04	***Clec4a2///Clec4b1***	C-type lectin domain family 4, member a2///C-type lectin domain family 4, member b1	0002250 // adaptive immune response // inferred from electronic annotation///0002376
NM_1461	0.0046			-1.04	1.22	1.10	1.09	***Tubb4b///Tubb4b-ps1***	tubulin, beta 4B class IVB///tubulin, beta 4B class IVB, pseudogene 1	0007017 // microtubule-based process // non-traceable author statement///0008152 //
NM_0011	0.0419			-1.02	1.11	1.10	1.01	***Cebpzos***	CCAAT/enhancer binding protein (C/EBP), zeta, opposite strand	
NM_0259	0.0483			1.09	-1.04	1.10	1.11	***Mettl6***	methyltransferase like 6	0032259 // methylation // inferred from electronic annotation
NM_0117	0.0455			-1.03	-1.19	1.10	-1.04	***Ercc5***	excision repair cross-complementing rodent repair deficiency, complementation group	0006281 // DNA repair // inferred from mutant phenotype///0006283 // transcription-co
NM_001314013///		0.03	0.027	1.02	1.06	1.10	1.15	***Atg5***	autophagy related 5	0000045 // autophagosome assembly // inferred from mutant phenotype///0000422 // r
NM_0076	0.0357			1.04	1.56	1.10	-1.14	***Cd48***	CD48 antigen	0007165 // signal transduction // inferred from direct assay///0042110 // T cell activation
NM_0012	0.0198			-1.02	1.19	1.10	1.35	***Gapdh///Gapdh-ps15///Gm10313///Gm12***	glyceraldehyde-3-phosphate dehydrogenase pseudogene///glyceraldehyde-3-phospha	0000226 // microtubule cytoskeleton organization // not recorded///0005975 // carbohy
NM_001161746///NM_01			0.041	1.24	1.34	1.10	-1.12	***Tnfrsf12a***	tumor necrosis factor receptor superfamily, member 12a	0001525 // angiogenesis // inferred from electronic annotation///0006915 // apoptotic p
NM_001136067///		0.04		1.01	1.06	1.10	1.18	***Ikbkg***	inhibitor of kappaB kinase gamma	0001782 // B cell homeostasis // inferred from mutant phenotype///0006351 // transcrip
NM_0013	0.0213			1.00	1.17	1.10	1.20	***Scfd1***	Sec1 family domain containing 1	0000902 // cell morphogenesis // inferred from sequence or structural similarity///00016
NM_001294322///		0.042		-1.07	-1.00	1.10	1.16	***Mpv17***	MpV17 mitochondrial inner membrane protein	0000002 // mitochondrial genome maintenance // not recorded///0007605 // sensory pe
NM_0010	0.0082			1.10	1.37	1.10	1.08	***Grk6***	G protein-coupled receptor kinase 6	0002029 // desensitization of G-protein coupled receptor protein signaling pathway // no
NM_009786		0.015		-1.37	-1.78	1.10	-1.35	***Cacybp***	calcyclin binding protein	0007507 // heart development // inferred from electronic annotation///0007568 // aging
NM_1751	0.0346			1.05	-1.01	1.10	1.05	***Emc3***	ER membrane protein complex subunit 3	0034975 // protein folding in endoplasmic reticulum // not recorded
NM_0011	0.038			1.01	1.22	1.10	-1.22	***Crlf2***	cytokine receptor-like factor 2	0002380 // immunoglobulin secretion involved in immune response // inferred from dire
NM_0105	######			1.20	2.94	1.10	1.08	***Cyr61***	cysteine rich protein 61	0001558 // regulation of cell growth // inferred from electronic annotation///0001649 //
NM_001098271///NM_02			0.015	-1.03	1.02	1.10	1.16	***Tmem176a***	transmembrane protein 176A	2001199 // negative regulation of dendritic cell differentiation // inferred from mutant ph
NM_0103	0.0289			1.02	1.43	1.10	1.27	***H2-DMb1///H2-DMb2///LOC102642102***	histocompatibility 2, class II, locus Mb1///histocompatibility 2, class II, locus Mb2///un	0002376 // immune system process // inferred from electronic annotation///0002504 //
NM_028131///XM_		0.014		-1.06	1.06	1.10	1.00	***Cenpn***	centromere protein N	0007059 // chromosome segregation // inferred from electronic annotation///0007067 /
NM_0306	0.0081			-1.12	1.27	1.10	1.27	***Gpx2***	glutathione peroxidase 2	0001659 // temperature homeostasis // inferred from genetic interaction///0002862 // n
NM_0114	0.0083			1.08	1.30	1.10	1.00	***Sptlc2***	serine palmitoyltransferase, long chain base subunit 2	0006629 // lipid metabolic process // inferred from electronic annotation///0006665 // s
NM_024475///XM_00653			0.026	-1.00	1.00	1.10	1.20	***Ublcp1***	ubiquitin-like domain containing CTD phosphatase 1	0006470 // protein dephosphorylation // not recorded
NM_1449	0.0236	0.013		1.00	1.14	1.10	-1.00	***Smyd5***	SET and MYND domain containing 5	0032259 // methylation // inferred from electronic annotation
NM_0011	0.0055			1.03	-1.06	1.10	1.10	***Aplp2***	amyloid beta (A4) precursor-like protein 2	0001967 // suckling behavior // inferred from genetic interaction///0006878 // cellular co
NM_001048057///		0.032		-1.07	-1.01	1.10	1.04	***Rpl38***	ribosomal protein L38	0001501 // skeletal system development // inferred from mutant phenotype///0001501 /

RefSeq Tr	p 24h	p 48h	p 72h	FD Air	FD 24h	FD 48h	FD 72h	Gene Symbol	Gene Title	Gene Ontology Biological Process
NM_0252	0.0394	0.018		-1.08	1.03	1.10	-1.02	***Mecr***	mitochondrial trans-2-enoyl-CoA reductase	0006629 // lipid metabolic process // inferred from electronic annotation///0006631 // f
NM_0011	0.0181		0.027	-1.03	1.13	1.10	1.15	***Slc35b3***	solute carrier family 35, member B3	0002230 // positive regulation of defense response to virus by host // not recorded///000
NM_008858///XM		0.014		-1.14	-1.23	1.10	1.03	***Prkd1***	protein kinase D1	0001525 // angiogenesis // inferred from electronic annotation///0001938 // positive reg
NM_027357		0.038		-1.11	-1.01	1.10	1.08	***Psmd1***	proteasome (prosome, macropain) 26S subunit, non-ATPase, 1	0042176 // regulation of protein catabolic process // inferred from electronic annotation
NM_026030		0.023		-1.10	1.01	1.10	1.01	***Eif2s2***	eukaryotic translation initiation factor 2, subunit 2 (beta)	0001701 // in utero embryonic development // inferred from mutant phenotype///0002
NM_012012///XM		0.02		-1.12	1.04	1.10	-1.06	***Exo1***	exonuclease 1	0002376 // immune system process // inferred from electronic annotation///0002455 //
NM_0010	0.0043			1.30	1.00	1.10	1.27	***Atp2b2***	ATPase, Ca++ transporting, plasma membrane 2	0000902 // cell morphogenesis // inferred from mutant phenotype///0006810 // transpo
NM_0012	0.0435			-1.02	1.05	1.11	1.01	***Pfkp***	phosphofructokinase, platelet	0006002 // fructose 6-phosphate metabolic process // not recorded///0006096 // glycoly
NM_009838		0.042		-1.07	-1.11	1.11	1.02	***Cct6a***	chaperonin containing Tcp1, subunit 6a (zeta)	0006457 // protein folding // inferred from electronic annotation///0007339 // binding o
NM_0120	0.0107			-1.03	1.13	1.11	1.18	***Cops6***	COP9 (constitutive photomorphogenic) homolog, subunit 6 (Arabidopsis thaliana)	0010388 // cullin deneddylation // not recorded
NM_001285452///		0.014	0.026	-1.33	-1.38	1.11	1.09	***Tmem33***	transmembrane protein 33	0034613 // cellular protein localization // not recorded///0051292 // nuclear pore compl
NM_001303423///NM_00			0.022	-1.01	-1.10	1.11	1.32	***Aldoc***	aldolase C, fructose-bisphosphate	0006096 // glycolytic process // inferred from electronic annotation///0006915 // apopto
NM_1810	0.0375			1.08	-1.00	1.11	-1.07	***Plekhh1***	pleckstrin homology domain containing, family H (with MyTH4 domain) member 1	
NM_0111	0.0086			1.17	2.12	1.11	1.14	***Pou2af1***	POU domain, class 2, associating factor 1	0006351 // transcription, DNA-templated // inferred from electronic annotation///00063
NM_0082	0.003			-1.14	1.36	1.11	1.09	***Hcls1***	hematopoietic cell specific Lyn substrate 1	0000122 // negative regulation of transcription from RNA polymerase II promoter // infer
NM_1537	0.0497			-1.00	1.18	1.11	1.03	***Pygb***	brain glycogen phosphorylase	0005975 // carbohydrate metabolic process // inferred from electronic annotation///000
NM_019814///XM		0.032		-1.14	-1.03	1.11	1.04	***Higd1a***	HIG1 domain family, member 1A	0006810 // transport // inferred from electronic annotation///0042149 // cellular respon
NM_021518///XM		0.011		-1.04	1.01	1.11	1.01	***Rab2a***	RAB2A, member RAS oncogene family	0006810 // transport // inferred from electronic annotation///0006886 // intracellular pr
NM_0091	0.0199			-1.12	1.11	1.11	1.11	***S100a10///LOC105242499***	S100 calcium binding protein A10 (calpactin)///protein S100-A10-like	0001765 // membrane raft assembly // not recorded///0006900 // membrane budding /
NM_0265	0.0445	0.031		1.01	1.06	1.11	-1.02	***N6amt2***	N-6 adenine-specific DNA methyltransferase 2 (putative)	0032259 // methylation // inferred from electronic annotation
NM_0011	0.0096			-1.05	-1.33	1.11	-1.01	***Erlin1***	ER lipid raft associated 1	0006629 // lipid metabolic process // inferred from electronic annotation///0008202 // s
NM_1340	0.0139	0.003		-1.10	1.13	1.11	-1.04	***Fbxo4***	F-box protein 4	0000209 // protein polyubiquitination // not recorded///0000723 // telomere maintenan
NM_1339	0.0159			1.07	-1.14	1.11	1.07	***Plekha1***	pleckstrin homology domain containing, family A (phosphoinositide binding specific) m	0001553 // luteinization // inferred from genetic interaction///0007283 // spermatogene
NM_0284	0.0417			1.02	1.17	1.11	-1.05	***Prrc1***	proline-rich coiled-coil 1	
NM_0010	0.0068			1.09	1.54	1.11	1.17	***Capg***	capping protein (actin filament), gelsolin-like	0030031 // cell projection assembly // inferred from direct assay///0051016 // barbed-er
NM_021457			0.023	-1.01	1.06	1.11	1.16	***Fzd1***	frizzled homolog 1 (Drosophila)	0001934 // positive regulation of protein phosphorylation // inferred from mutant phenc
NM_026792		0.033		-1.07	-1.06	1.11	1.02	***Agpat5***	1-acylglycerol-3-phosphate O-acyltransferase 5 (lysophosphatidic acid acyltransferase,	0002244 // hematopoietic progenitor cell differentiation // inferred from genetic interact
NM_024433		0.021		-1.02	1.07	1.11	1.03	***Mtap***	methylthioadenosine phosphorylase	0006166 // purine ribonucleoside salvage // inferred from electronic annotation///00091
NM_0010	0.0461	0.039		1.06	-1.01	1.11	1.09	***Vprbp***	Vpr (HIV-1) binding protein	0000122 // negative regulation of transcription from RNA polymerase II promoter // not
NM_0089	0.0107			-1.07	1.24	1.11	1.12	***Psmb6***	proteasome (prosome, macropain) subunit, beta type 6	0006508 // proteolysis // inferred from electronic annotation///0010499 // proteasomal
NM_021539		0.004		-1.07	1.01	1.11	1.05	***Wsb2***	WD repeat and SOCS box-containing 2	0016567 // protein ubiquitination // inferred from electronic annotation///0035556 // in
NM_0256	0.0401			1.05	1.24	1.11	1.04	***Pspc1***	paraspeckle protein 1	0000398 // mRNA splicing, via spliceosome // not recorded///0006351 // transcription, D
NM_0173	0.0071	0.011		1.55	1.11	1.11	1.74	***Tef***	thyrotroph embryonic factor	0006351 // transcription, DNA-templated // inferred from electronic annotation///00063
NM_0217	0.0187			-1.16	1.52	1.11	-1.09	***Ms4a4b***	membrane-spanning 4-domains, subfamily A, member 4B	
NM_010751///XM		0.025		-1.06	1.03	1.11	-1.20	***Mxd1***	MAX dimerization protein 1	0000122 // negative regulation of transcription from RNA polymerase II promoter // not
NM_001242349///		#####	0.005	-1.15	1.16	1.11	1.20	***Ano1***	anoctamin 1, calcium activated chloride channel	0006810 // transport // inferred from electronic annotation///0006811 // ion transport /
NM_0012	0.0017			-1.15	1.16	1.11	1.20	***Ano1***	anoctamin 1, calcium activated chloride channel	0006810 // transport // inferred from electronic annotation///0006811 // ion transport /
NM_1445	0.0111			1.05	1.19	1.11	1.23	***Bre***	brain and reproductive organ-expressed protein	0006281 // DNA repair // inferred from electronic annotation///0006302 // double-stran
NM_0264	0.0136			1.06	1.23	1.11	1.03	***Atp5g2///LOC102641133***	ATP synthase, H+ transporting, mitochondrial F0 complex, subunit C2 (subunit 9)///AT	0006810 // transport // inferred from electronic annotation///0006811 // ion transport /
NM_0084	0.0483			-1.07	1.13	1.11	1.12	***Itm2a***	integral membrane protein 2A	
NM_001039718///		0.023		-1.01	1.06	1.11	-1.14	***Cntf///Zfp91///Zfp91Cntf***	ciliary neurotrophic factor///zinc finger protein 91///Zfp91-Cntf readthrough transcrip	0007250 // activation of NF-kappaB-inducing kinase activity // not recorded///0007259 /
NM_026842///NM		0.034		-1.11	-1.11	1.11	-1.08	***Ubqln1***	ubiquilin 1	0006914 // autophagy // inferred from electronic annotation///0016241 // regulation of
NM_0010	0.0209			-1.01	1.19	1.11	1.10	***Med15***	mediator complex subunit 15	0006351 // transcription, DNA-templated // inferred from electronic annotation///00063
NM_0012	0.0282	0.014	0.02	-1.13	1.00	1.11	1.03	***Farsb***	phenylalanyl-tRNA synthetase, beta subunit	0006412 // translation // inferred from electronic annotation///0006432 // phenylalanyl-
NM_0286	0.047			1.04	-1.11	1.11	1.12	***Mcee***	methylmalonyl CoA epimerase	0046491 // L-methylmalonyl-CoA metabolic process // not recorded
NM_0012	0.0489			1.03	-1.08	1.11	-1.15	***Fam105a***	family with sequence similarity 105, member A	
NM_0263	0.0229			1.02	1.19	1.11	1.02	***Poldip2***	polymerase (DNA-directed), delta interacting protein 2	0070584 // mitochondrion morphogenesis // inferred from mutant phenotype
NM_0196	0.0487			1.00	-1.04	1.11	1.14	***Socs5***	suppressor of cytokine signaling 5	0006469 // negative regulation of protein kinase activity // not recorded///0007173 // ep
NM_001271531///NM_00			0.035	-1.01	1.03	1.11	1.15	***Insig2***	insulin induced gene 2	0006629 // lipid metabolic process // inferred from electronic annotation///0006641 // t
NM_0256	0.0487			1.01	1.08	1.11	1.14	***Smc6***	structural maintenance of chromosomes 6	0000722 // telomere maintenance via recombination // not recorded///0000724 // doub
NM_1535	0.0107			-1.02	1.12	1.11	1.07	***Bcl2l13***	BCL2-like 13 (apoptosis facilitator)	0006915 // apoptotic process // inferred from reviewed computational analysis///00429
NM_0011	0.0254			1.04	1.21	1.11	1.00	***H13***	histocompatibility 13	0006508 // proteolysis // inferred from electronic annotation///0033619 // membrane p
NM_0012	0.0139	0.01		-1.37	-2.16	1.11	-1.21	***Ahsa2***	AHA1, activator of heat shock protein ATPase 2	0006950 // response to stress // inferred from electronic annotation///0032781 // positiv
NM_0011	0.0366	0.031		1.01	1.13	1.12	1.07	***Orc3***	origin recognition complex, subunit 3	0006260 // DNA replication // traceable author statement///0061351 // neural precursor
NM_1986	0.0301			1.09	1.27	1.12	1.01	***Ap2s1***	adaptor-related protein complex 2, sigma 1 subunit	0006810 // transport // inferred from electronic annotation///0006886 // intracellular pr
NM_001163332///		0.043		-1.18	-1.49	1.12	1.07	***Cttnbp2nl***	CTTNBP2 N-terminal like	0006470 // protein dephosphorylation // inferred from direct assay///0006470 // protein
NM_1339	0.048			1.02	1.18	1.12	1.05	***Fig4***	FIG4 homolog (S. cerevisiae)	0007033 // vacuole organization // inferred from mutant phenotype///0007626 // locom
NM_1528	0.024	0.007		-1.08	1.11	1.12	-1.05	***Ttc27***	tetratricopeptide repeat domain 27	
NM_0267	0.0148			-1.03	1.23	1.12	-1.01	***Mphosph6***	M phase phosphoprotein 6	0000460 // maturation of 5.8S rRNA // not recorded///0006364 // rRNA processing // inf
NM_134010///XM		0.036		-1.01	1.09	1.12	1.08	***Nup107***	nucleoporin 107	0000973 // posttranscriptional tethering of RNA polymerase II gene DNA at nuclear perip
NM_025578		0.01		-1.02	-1.02	1.12	1.00	***Mrps25***	mitochondrial ribosomal protein S25	0032543 // mitochondrial translation // inferred by curator
NM_0260	0.006			-1.03	1.24	1.12	1.00	***Cdt1***	chromatin licensing and DNA replication factor 1	0000076 // DNA replication checkpoint // inferred from direct assay///0000076 // DNA re
NM_1462	0.0278			1.10	-1.12	1.12	1.04	***Msantd2***	Myb/SANT-like DNA-binding domain containing 2	
NM_021511		0.025		-1.10	1.02	1.12	1.08	***Rrs1***	RRS1 ribosome biogenesis regulator homolog (S. cerevisiae)	0000055 // ribosomal large subunit export from nucleus // ---///0000447 // endonucleol

RefSeq Tr	*p* 24h	*p* 48h	*p* 72h	FD Air	FD 24h	FD 48h	FD 72h	Gene Symbol	Gene Title	Gene Ontology Biological Process
NM_0083	0.0224			1.05	-1.07	1.12	1.12	***Sdc2***	syndecan 2	0007399 // nervous system development // inferred from electronic annotation///00164
NM_0261	0.0218	0.003	0.013	-1.11	1.02	1.12	1.05	***Ergic1***	endoplasmic reticulum-golgi intermediate compartment (ERGIC) 1	0006810 // transport // inferred from electronic annotation///0006888 // ER to Golgi ves
NM_001164406///		0.014		-1.02	1.06	1.12	1.00	***Apbb3///Sra1***	amyloid beta (A4) precursor protein-binding, family B, member 3///steroid receptor RI	0006351 // transcription, DNA-templated // inferred from electronic annotation///00063
NM_0265	0.0462			1.04	1.33	1.12	-1.05	***Psmd8***	proteasome (prosome, macropain) 26S subunit, non-ATPase, 8	0006508 // proteolysis // inferred from electronic annotation///0031647 // regulation of
NM_007512///XM_		0.05		-1.09	1.01	1.12	1.09	***Atpif1***	ATPase inhibitory factor 1	0001937 // negative regulation of endothelial cell proliferation // not recorded///000678
NM_0223	######			1.02	-1.56	1.12	-1.09	***Herpud1***	homocysteine-inducible, endoplasmic reticulum stress-inducible, ubiquitin-like domair	0006511 // ubiquitin-dependent protein catabolic process // inferred from direct assay//
NM_0254	0.0205	0.006		-1.08	1.12	1.12	-1.09	***Sec11c***	SEC11 homolog C, signal peptidase complex subunit	0006465 // signal peptide processing // inferred from electronic annotation///0006508 /
NM_001142357///		#####		-1.01	-1.01	1.12	1.04	***Alg12***	asparagine-linked glycosylation 12 (alpha-1,6-mannosyltransferase)	0006486 // protein glycosylation // inferred from electronic annotation///0006487 // prc
NM_0103	0.0029	0.004		-1.07	1.15	1.12	1.13	***Got2***	glutamatic-oxaloacetic transaminase 2, mitochondrial	0006103 // 2-oxoglutarate metabolic process // inferred from sequence or structural sim
NM_0256	0.024	0.037		-1.02	-1.10	1.12	1.05	***Aggf1***	angiogenic factor with G patch and FHA domains 1	0001525 // angiogenesis // inferred from electronic annotation///0001938 // positive reg
NM_0118	0.0086			1.04	1.27	1.12	1.16	***Esyt1***	extended synaptotagmin-like protein 1	0006810 // transport // inferred from electronic annotation///0006869 // lipid transport
NM_001164107///NM_00			0.048	-1.07	1.16	1.12	1.11	***Ripk3***	receptor-interacting serine-threonine kinase 3	0001914 // regulation of T cell mediated cytotoxicity // inferred from genetic interaction/
NM_008597			0.011	-1.09	-1.10	1.12	1.06	***Mgp***	matrix Gla protein	0001503 // ossification // inferred from electronic annotation///0006461 // protein comp
NM_0281	0.0066	0.004		1.01	1.15	1.12	1.17	***Scaf11***	SR-related CTD-associated factor 11	0000245 // spliceosomal complex assembly // not recorded///0000375 // RNA splicing, v
NM_008490		#####		-1.03	1.01	1.12	-1.03	***Lcat***	lecithin cholesterol acyltransferase	0006629 // lipid metabolic process // inferred from electronic annotation///0006644 // p
NM_0173	0.0193	0.005		-1.06	1.04	1.12	-1.04	***Ppp2cb***	protein phosphatase 2 (formerly 2A), catalytic subunit, beta isoform	0006470 // protein dephosphorylation // not recorded///0008637 // apoptotic mitochon
NM_009255///XM_		0.006		-1.16	-1.26	1.12	1.02	***Serpine2***	serine (or cysteine) peptidase inhibitor, clade E, member 2	0007275 // multicellular organismal development // inferred from electronic annotation/
NM_0076	0.0113			-1.02	1.23	1.12	-1.07	***Cd38***	CD38 antigen	0001666 // response to hypoxia // not recorded///0007204 // positive regulation of cyto
NM_031248		0.039		-1.02	1.06	1.12	1.09	***Lamtor2***	late endosomal/lysosomal adaptor, MAPK and MTOR activator 2	0000186 // activation of MAPKK activity // inferred from direct assay///0016049 // cell gr
NM_145531///NM		0.03		1.01	1.04	1.12	1.15	***Spg11***	spastic paraplegia 11	0007409 // axonogenesis // not recorded///0008088 // axon cargo transport // inferred f
NM_0101	0.0053			-1.00	1.78	1.12	-1.03	***Egr2***	early growth response 2	0006351 // transcription, DNA-templated // inferred from electronic annotation///00063
NM_0011	0.0476	0.015		-1.01	1.09	1.12	-1.00	***Slc25a13***	solute carrier family 25 (mitochondrial carrier, adenine nucleotide translocator), meml	0006754 // ATP biosynthetic process // not recorded///0006810 // transport // inferred f
NM_001177629///		0.012		-1.11	-1.17	1.12	1.08	***Grb10***	growth factor receptor bound protein 10	0007165 // signal transduction // traceable author statement///0008286 // insulin recep
NM_0266	0.0014			1.18	-1.14	1.12	-1.17	***Zmym1***	zinc finger, MYM domain containing 1	
NM_024188		0.012		-1.13	-1.09	1.12	-1.03	***Oxct1***	3-oxoacid CoA transferase 1	0007420 // brain development // inferred from electronic annotation///0007507 // heart
NM_0283	0.0159	0.011		-1.06	1.13	1.12	1.00	***Dis3***	DIS3 mitotic control homolog (S. cerevisiae)	0006364 // rRNA processing // not recorded///0006402 // mRNA catabolic process // no
NM_001301269		0.02	0.015	-1.11	-1.05	1.12	1.15	***Inafm2***	InaF motif containing 2	
NM_0011	0.0323			1.03	1.12	1.12	1.11	***Nol11***	nucleolar protein 11	0006351 // transcription, DNA-templated // inferred from electronic annotation///00063
NM_0012	0.0033			1.13	1.01	1.12	1.27	***Slc7a7***	solute carrier family 7 (cationic amino acid transporter, y+ system), member 7	0000821 // regulation of arginine metabolic process // inferred from mutant phenotype/
NM_0013	0.0404			-1.11	1.20	1.12	1.15	***Il2rg***	interleukin 2 receptor, gamma chain	0010468 // regulation of gene expression // inferred from mutant phenotype///0010628
NM_001039084///		0.021		1.02	1.02	1.12	1.01	***Mrpl1***	mitochondrial ribosomal protein L1	0006412 // translation // not recorded
NM_0200	0.043			1.06	1.26	1.12	-1.19	***Taf10***	TAF10 RNA polymerase II, TATA box binding protein (TBP)-associated factor	0000082 // G1/S transition of mitotic cell cycle // inferred from mutant phenotype///000
NM_0074	0.005			-1.10	1.19	1.12	1.21	***Slc25a5***	solute carrier family 25 (mitochondrial carrier, adenine nucleotide translocator), meml	0006810 // transport // inferred from electronic annotation///0007059 // chromosome s
NM_0086	0.003	0.004		-1.15	1.09	1.12	-1.14	***Mthfd2***	methylenetetrahydrofolate dehydrogenase (NAD+ dependent), methenyltetrahydrofol	0006730 // one-carbon metabolic process // not recorded///0008152 // metabolic proce
NM_0280	0.0293			-1.03	-1.10	1.12	-1.08	***Cnn3***	calponin 3, acidic	0030855 // epithelial cell differentiation // inferred from electronic annotation///003103
NM_009836		0.006	0.039	-1.11	-1.12	1.12	1.06	***Cct3***	chaperonin containing Tcp1, subunit 3 (gamma)	0006457 // protein folding // inferred from electronic annotation///0007339 // binding o
NM_001033201		0.027		-1.01	1.03	1.12	1.05	***Ncbp1***	nuclear cap binding protein subunit 1	0000184 // nuclear-transcribed mRNA catabolic process, nonsense-mediated decay // no
NM_010617///XM_		0.027		-1.04	-1.08	1.13	1.10	***Kif13a***	kinesin family member 13A	0000910 // cytokinesis // not recorded///0006810 // transport // inferred from electroni
NM_026555///XM_		0.025		-1.10	-1.03	1.13	-1.24	***Rcn3***	reticulocalbin 3, EF-hand calcium binding domain	
NM_1454	0.0323	0.008		1.03	1.12	1.13	1.04	***Ube4a***	ubiquitination factor E4A	0000209 // protein polyubiquitination // inferred from direct assay///0006511 // ubiquiti
NM_175451		0.023		-1.12	-1.01	1.13	1.15	***Ckap4***	cytoskeleton-associated protein 4	
NM_0011	0.0461			1.09	1.34	1.13	1.13	***Mef2c***	myocyte enhancer factor 2C	0000122 // negative regulation of transcription from RNA polymerase II promoter // infer
NM_019698///NM		0.005	0.05	-1.15	1.01	1.13	1.07	***Aldh18a1***	aldehyde dehydrogenase 18 family, member A1	0006536 // glutamate metabolic process // not recorded///0006561 // proline biosynthe
NM_0228	0.0216			1.11	-1.01	1.13	1.09	***Svep1***	sushi, von Willebrand factor type A, EGF and pentraxin domain containing 1	0007155 // cell adhesion // inferred from electronic annotation
NM_0010	0.0281			1.03	-1.11	1.13	1.14	***Cbx5***	chromobox 5	0045892 // negative regulation of transcription, DNA-templated // inferred from direct as
NM_173180///XM_		0.025		-1.00	-1.00	1.13	1.04	***Pmpca***	peptidase (mitochondrial processing) alpha	0006508 // proteolysis // not recorded///0006627 // protein processing involved in prote
NM_145552///XM_		0.03		-1.06	-1.01	1.13	1.03	***Gnl2***	guanine nucleotide binding protein-like 2 (nucleolar)	0042254 // ribosome biogenesis // not recorded
NM_0011	0.0313			1.06	-1.18	1.13	1.05	***Gpbp1***	GC-rich promoter binding protein 1	0006351 // transcription, DNA-templated // inferred from electronic annotation///00063
NM_0531	0.0137	#####		-1.03	1.13	1.13	-1.01	***Mrpl3***	mitochondrial ribosomal protein L3	0006412 // translation // not recorded
NM_0011	0.0079			1.04	1.23	1.13	1.16	***Pcx***	pyruvate carboxylase	0006090 // pyruvate metabolic process // not recorded///0006094 // gluconeogenesis //
NM_0112	0.0173			-1.07	1.23	1.13	-1.14	***Ranbp1***	RAN binding protein 1	0007051 // spindle organization // inferred from direct assay///0010976 // positive regul
NM_0254	0.0165			1.05	1.27	1.13	1.29	***Alg5***	asparagine-linked glycosylation 5 (dolichyl-phosphate beta-glucosyltransferase)	0006486 // protein glycosylation // inferred from electronic annotation///0006487 // prc
NM_146067///XM_006522			0.045	-1.04	1.08	1.13	1.09	***Cpped1***	calcineurin-like phosphoesterase domain containing 1	0006470 // protein dephosphorylation // inferred from electronic annotation///0008152
NM_0105	0.0312		0.022	1.20	1.11	1.13	1.12	***Jup***	junction plakoglobin	0002159 // desmosome assembly // inferred from mutant phenotype///0002159 // desm
NM_001301364///		0.029	0.049	-1.05	1.04	1.13	1.20	***Hdlbp***	high density lipoprotein (HDL) binding protein	0006629 // lipid metabolic process // inferred from electronic annotation///0006810 // t
NM_198103			0.042	1.03	-1.04	1.13	1.15	***Exoc8***	exocyst complex component 8	0001927 // exocyst assembly // not recorded///0006810 // transport // inferred from ele
NM_0011	0.0091			1.14	-1.01	1.13	1.23	***Rnf145***	ring finger protein 145	0000209 // protein polyubiquitination // ---///0042787 // protein ubiquitination involved
NM_031843///XR_		0.01	0.037	-1.06	-1.03	1.13	1.07	***Dpp7***	dipeptidylpeptidase 7	0006508 // proteolysis // not recorded
NM_026438		0.029		-1.15	1.03	1.13	-1.11	***Ppa1***	pyrophosphatase (inorganic) 1	0006796 // phosphate-containing compound metabolic process // inferred by curator
NM_0095	0.0176			-1.06	1.19	1.13	1.00	***Vcp***	valosin containing protein	0006281 // DNA repair // inferred from electronic annotation///0006302 // double-stran
NM_0010	0.0079	0.02	0.001	-1.12	1.05	1.13	1.22	***Klhl42***	kelch-like 42	0000209 // protein polyubiquitination // not recorded///0007049 // cell cycle // inferred
NM_023232		0.019		-1.03	1.06	1.13	1.13	***Diablo***	diablo homolog (Drosophila)	0006915 // apoptotic process // inferred from electronic annotation///0006919 // activa
NM_0265	0.0465			1.07	1.00	1.13	1.22	***Mrpl24***	mitochondrial ribosomal protein L24	0006412 // translation // inferred from electronic annotation
NM_0010	0.001	0.022		-1.15	1.23	1.13	-1.02	***Rnf149***	ring finger protein 149	0016567 // protein ubiquitination // inferred from electronic annotation///0031647 // re

RefSeq Tr	p 24h	p 48h	p 72h	FD Air	FD 24h	FD 48h	FD 72h	Gene Symbol	Gene Title	Gene Ontology Biological Process
NM_145972///XM		0.006		-1.02	-1.07	1.13	1.04	***Nepro***	nucleolus and neural progenitor protein	0045665 // negative regulation of neuron differentiation // inferred from direct assay///0
NM_0083	0.0089			-1.04	1.15	1.13	1.12	***Idh3g***	isocitrate dehydrogenase 3 (NAD+), gamma	0006099 // tricarboxylic acid cycle // not recorded///0006102 // isocitrate metabolic pro
NM_008661///NM		0.04		-1.06	-1.06	1.13	1.14	***Myo5b***	myosin VB	0006810 // transport // inferred from electronic annotation///0008152 // metabolic proc
NM_0011	0.0397	0.009		-1.58	-1.08	1.13	-1.14	***Pde4b***	phosphodiesterase 4B, cAMP specific	0001780 // neutrophil homeostasis // inferred from mutant phenotype///0006198 // cAM
NM_0134	0.0045			-1.01	1.72	1.13	1.11	***Cd2***	CD2 antigen	0007155 // cell adhesion // inferred from electronic annotation///0016337 // single orga
NM_0109	0.0179			1.07	-1.17	1.13	1.15	***Nfe2l2***	nuclear factor, erythroid derived 2, like 2	0006351 // transcription, DNA-templated // inferred from electronic annotation///00063
NM_001077411///		0.035		-1.04	1.01	1.13	1.13	***Gba***	glucosidase, beta, acid	0005975 // carbohydrate metabolic process // inferred from electronic annotation///000
NM_0012	0.0157			-1.04	1.26	1.13	1.42	***Gapdh///Gapdh-ps15///Gm10313///Gm12***	glyceraldehyde-3-phosphate dehydrogenase///glyceraldehyde-3-phosphate dehydrog	0000226 // microtubule cytoskeleton organization // not recorded///0005975 // carbohy
NM_025860///XM		0.012		-1.00	1.04	1.14	1.13	***Ddx18***	DEAD (Asp-Glu-Ala-Asp) box polypeptide 18	0010501 // RNA secondary structure unwinding // not recorded
NM_0260	0.0274	0.023		-1.09	1.12	1.14	1.17	***Eef1g***	eukaryotic translation elongation factor 1 gamma	0006412 // translation // inferred from electronic annotation///0006414 // translational
NM_0012	0.0471			-1.01	1.15	1.14	1.02	***Prnp***	prion protein	0001933 // negative regulation of protein phosphorylation // inferred from mutant phen
NM_0270	0.0213			-1.16	1.07	1.14	1.12	***Rfc3***	replication factor C (activator 1) 3	0006260 // DNA replication // not recorded///0006261 // DNA-dependent DNA replicatio
NM_019491		0.022		-1.03	1.05	1.14	-1.06	***Rala***	v-ral simian leukemia viral oncogene homolog A (ras related)	0000910 // cytokinesis // not recorded///0001843 // neural tube closure // inferred from
NM_0011	0.0235			1.01	-1.03	1.14	-1.06	***Ino80///Rpl35a///Rpl35a-ps2///Rpl35a-ps***	INO80 homolog ///ribosomal protein L35A///ribosomal protein L35A, pseudogene 2//	0000070 // mitotic sister chromatid segregation // not recorded///0000724 // double-str
NM_0076	0.0013	0.011		-1.04	1.08	1.14	1.15	***Casp7***	caspase 7	0006508 // proteolysis // not recorded///0006508 // proteolysis // inferred from reviewe
NM_0254	0.0241	0.036		-1.10	1.12	1.14	-1.05	***Mrps22***	mitochondrial ribosomal protein S22	
NM_0104	0.0337			-1.01	1.17	1.14	1.07	***Hexb***	hexosaminidase B	0001501 // skeletal system development // inferred from genetic interaction///0005975 ,
NM_0167	0.0187			1.09	1.25	1.14	1.07	***Smad3***	SMAD family member 3	0000122 // negative regulation of transcription from RNA polymerase II promoter // infer
NM_0010	0.0209			-1.03	1.15	1.14	1.04	***Eif4e2***	eukaryotic translation initiation factor 4E member 2	0001701 // in utero embryonic development // inferred from mutant phenotype///00064
NM_0104	0.0074	0.015		-1.10	1.09	1.14	-1.02	***Icam1***	intercellular adhesion molecule 1	0001541 // ovarian follicle development // inferred from electronic annotation///000166
NM_019639			0.042	1.07	-1.02	1.14	1.01	***Ubc***	ubiquitin C	0007623 // circadian rhythm // traceable author statement///0010992 // ubiquitin home
NM_001010937///		0.041		-1.09	-1.01	1.14	-1.28	***Gjb6***	gap junction protein, beta 6	0006915 // apoptotic process // inferred from electronic annotation///0007154 // cell co
NM_0012	0.0477			1.07	1.25	1.14	1.12	***Hgf***	hepatocyte growth factor	0000187 // activation of MAPK activity // inferred from direct assay///0000902 // cell mo
NM_025460		0.013	0.048	-1.14	1.01	1.14	1.14	***Tmem126a***	transmembrane protein 126A	0021554 // optic nerve development // not recorded
NM_021336///XR_391365			0.001	-1.03	1.03	1.14	1.23	***Snrpa1***	small nuclear ribonucleoprotein polypeptide A'	0000398 // mRNA splicing, via spliceosome // ---///0006397 // mRNA processing // inferr
NM_018884///XM		0.002		-1.08	-1.12	1.14	1.09	***Pdzrn3***	PDZ domain containing RING finger 3	0007528 // neuromuscular junction development // inferred from mutant phenotype///0
NM_172490///XM		0.006		-1.02	1.06	1.14	1.22	***Sepsecs***	Sep (O-phosphoserine) tRNA:Sec (selenocysteine) tRNA synthase	0001514 // selenocysteine incorporation // inferred from direct assay///0006412 // trans
NM_008079///XM		0.021	0.04	1.03	1.02	1.14	1.14	***Galc***	galactosylceramidase	0005975 // carbohydrate metabolic process // inferred from electronic annotation///000
NM_010914///XM		0.036		-1.03	1.03	1.14	1.22	***Nfyb***	nuclear transcription factor-Y beta	0006351 // transcription, DNA-templated // inferred from electronic annotation///00063
NM_0095	0.0293			1.19	-1.07	1.14	1.29	***Wee1***	WEE 1 homolog 1 (S. pombe)	0000226 // microtubule cytoskeleton organization // inferred from mutant phenotype///
NM_0112	0.0318			-1.02	1.13	1.14	1.16	***Rfc1***	replication factor C (activator 1) 1	0000122 // negative regulation of transcription from RNA polymerase II promoter // not r
NM_0011	0.0283	0.018		-1.02	1.16	1.14	-1.02	***Nek6***	NIMA (never in mitosis gene a)-related expressed kinase 6	0006468 // protein phosphorylation // not recorded///0006915 // apoptotic process // ir
NM_008971///XM_006508			0.022	-1.04	1.01	1.14	1.22	***Gm4887///Twf1***	predicted gene 4887///twinfilin, actin-binding protein, homolog 1 (Drosophila)	0018108 // peptidyl-tyrosine phosphorylation // inferred from direct assay///0030837 //
NM_0011	0.0054			-1.07	1.22	1.14	-1.07	***Raly***	hnRNP-associated with lethal yellow	0006397 // mRNA processing // inferred from electronic annotation///0008380 // RNA sp
NM_001304574///		0.017		1.02	1.11	1.14	1.20	***Arf2***	ADP-ribosylation factor 2	0006810 // transport // inferred from electronic annotation///0007264 // small GTPase r
NM_001291146///		0.008		-1.08	-1.10	1.14	1.09	***Derl2***	Der1-like domain family, member 2	0001967 // suckling behavior // inferred from mutant phenotype///0006986 // response
NM_172393///XM		0.036		-1.06	-1.03	1.14	1.11	***Aim1***	absent in melanoma 1	
NM_025331			0.008	-1.03	-1.04	1.14	-1.24	***Gng11***	guanine nucleotide binding protein (G protein), gamma 11	0007165 // signal transduction // inferred from electronic annotation///0007186 // G-pro
NM_0010	0.0281			1.12	-1.01	1.14	1.09	***Sdcbp***	syndecan binding protein	0007265 // Ras protein signal transduction // inferred from physical interaction///00072
NM_133895///XM		0.02		-1.05	-1.04	1.14	1.08	***Slc15a4***	solute carrier family 15, member 4	0006810 // transport // inferred from electronic annotation///0006857 // oligopeptide tr
NM_172146		0.042		-1.01	-1.04	1.14	1.14	***Ppat***	phosphoribosyl pyrophosphate amidotransferase	0000082 // G1/S transition of mitotic cell cycle // inferred from electronic annotation///0
NM_009191///XM		0.023		-1.02	-1.02	1.14	-1.03	***Clpb***	ClpB caseinolytic peptidase B	0008152 // metabolic process // inferred from electronic annotation///0034605 // cellula
NM_0010	0.0209			1.02	1.24	1.15	1.17	***Casp8***	caspase 8	0001525 // angiogenesis // inferred from mutant phenotype///0001841 // neural tube fo
NM_001008238///		0.042		-1.10	1.07	1.15	-1.04	***Bnip2***	BCL2/adenovirus E1B interacting protein 2	0001824 // blastocyst development // inferred from mutant phenotype///0006915 // apo
NM_0010	0.0024			-1.04	1.26	1.15	-1.03	***Nme2***	NME/NM23 nucleoside diphosphate kinase 2	0002762 // negative regulation of myeloid leukocyte differentiation // not recorded///00
NM_026850		0.041		-1.04	-1.02	1.15	1.04	***Pdcl3***	phosducin-like 3	0001525 // angiogenesis // inferred from electronic annotation///0001938 // positive reg
NM_0295	0.0328			1.05	-1.02	1.15	1.16	***Tax1bp3***	Tax1 (human T cell leukemia virus type I) binding protein 3	0007266 // Rho protein signal transduction // not recorded///0008285 // negative regula
NM_198831///NR		0.015		1.04	1.12	1.15	-1.03	***Mrpl48///LOC102643207///Mrpl48-ps***	mitochondrial ribosomal protein L48///uncharacterized LOC102643207///mitochondrial ribosomal protein L48 pseudogene	
NM_0011	0.011			1.10	1.37	1.15	1.23	***Trp53***	transformation related protein 53	0000060 // protein import into nucleus, translocation // inferred from direct assay///000
NM_0076	0.0033		0.043	-1.04	-1.10	1.15	1.15	***Scarb2***	scavenger receptor class B, member 2	0006622 // protein targeting to lysosome // inferred from mutant phenotype///0006622
NM_001033257///		0.048		1.01	-1.18	1.15	-1.04	***Phactr2***	phosphatase and actin regulator 2	0043086 // negative regulation of catalytic activity // inferred from electronic annotation
NM_007735///XM		0.019		1.02	1.09	1.15	1.01	***Col4a4***	collagen, type IV, alpha 4	0032836 // glomerular basement membrane development // inferred from mutant phen
NM_001285852///NM_00			0.041	1.02	1.06	1.15	1.16	***Sgip1***	SH3-domain GRB2-like (endophilin) interacting protein 1	0002021 // response to dietary excess // inferred from sequence or structural similarity//
NM_025319///XM		0.023	0.009	-1.21	-1.03	1.15	1.08	***0610009B22Rik***	RIKEN cDNA 0610009B22 gene	0006351 // transcription, DNA-templated // inferred from electronic annotation///00063
NM_134101///NR		0.014		-1.04	1.10	1.15	1.20	***Psmd2***	proteasome (prosome, macropain) 26S subunit, non-ATPase, 2	0042176 // regulation of protein catabolic process // inferred from electronic annotation
NM_0010	0.0407			1.05	1.23	1.15	1.34	***Slc39a7***	solute carrier family 39 (zinc transporter), member 7	0006810 // transport // inferred from electronic annotation///0006811 // ion transport /
NM_019673///XM		0.029		-1.04	-1.08	1.15	1.06	***Actl6a***	actin-like 6A	0003407 // neural retina development // inferred from electronic annotation///0006281
NM_019656		0.006		-1.00	-1.05	1.15	1.02	***Tspan6***	tetraspanin 6	0007165 // signal transduction // ---///0007165 // signal transduction // inferred from ele
NM_144835		0.007		-1.05	1.12	1.15	-1.04	***Heatr1***	HEAT repeat containing 1	0000462 // maturation of SSU-rRNA from tricistronic rRNA transcript (SSU-rRNA, 5.8S rRN
NM_001291134///NM_00			0.009	1.04	1.12	1.15	1.23	***Rprd1b***	regulation of nuclear pre-mRNA domain containing 1B	0006351 // transcription, DNA-templated // inferred from electronic annotation///00063
NM_0259	0.0015			-1.07	1.22	1.15	1.04	***Rpl11***	ribosomal protein L11	0000027 // ribosomal large subunit assembly // ---///0006364 // rRNA processing // not r
NM_1453	0.0189	0.035		-1.05	1.18	1.15	1.00	***Nsun2***	NOL1/NOP2/Sun domain family member 2	0007049 // cell cycle // inferred from electronic annotation///0007067 // mitotic nuclear
NM_0076	0.0226	0.021		-1.18	1.03	1.15	1.08	***Cct5***	chaperonin containing Tcp1, subunit 5 (epsilon)	0006457 // protein folding // inferred from electronic annotation///0007339 // binding o
NM_025387///XM		0.006		-1.07	1.07	1.15	1.20	***Gm4246///Tmem14c***	predicted gene 4246///transmembrane protein 14C	0006783 // heme biosynthetic process // inferred from electronic annotation///0006839

RefSeq Tr	*p* 24h	*p* 48h	*p* 72h	FD Air	FD 24h	FD 48h	FD 72h	Gene Symbol	Gene Title	Gene Ontology Biological Process
NM_0011	0.002			-1.00	1.25	1.15	1.18	***Rnh1***	ribonuclease/angiogenin inhibitor 1	0043086 // negative regulation of catalytic activity // not recorded///0045765 // regulati
NM_0011	0.0097	0.018		-1.12	1.18	1.15	1.18	***Kars***	lysyl-tRNA synthetase	0006412 // translation // inferred from electronic annotation///0006418 // tRNA aminoa
NM_0808	0.0368			-1.03	1.16	1.15	-1.02	***Mydgf***	myeloid derived growth factor	0001525 // angiogenesis // inferred from electronic annotation///0001934 // positive reg
NM_001313906///NM_00			0.029	-1.11	1.02	1.15	1.36	***Prmt5***	protein arginine N-methyltransferase 5	0000122 // negative regulation of transcription from RNA polymerase II promoter // infer
NM_0089	0.0264	0.009		-1.08	-1.01	1.15	1.06	***Prim1***	DNA primase, p49 subunit	0006260 // DNA replication // inferred from electronic annotation///0006269 // DNA rep
NM_0258	0.0341			-1.04	1.08	1.15	1.11	***Tax1bp1***	Tax1 (human T cell leukemia virus type I) binding protein 1	0006915 // apoptotic process // inferred from electronic annotation///0032088 // negati
NM_021457		0.002		-1.01	1.05	1.16	1.16	***Fzd1***	frizzled homolog 1 (Drosophila)	0001934 // positive regulation of protein phosphorylation // inferred from mutant phenc
NM_145630		0.031		-1.05	1.01	1.16	1.07	***Pdk3***	pyruvate dehydrogenase kinase, isoenzyme 3	0005975 // carbohydrate metabolic process // inferred from electronic annotation///000
NM_0258	######			-1.18	-2.54	1.16	-1.08	***Chordc1***	cysteine and histidine-rich domain (CHORD)-containing, zinc-binding protein 1	0010824 // regulation of centrosome duplication // inferred from mutant phenotype///0
NM_026234			0.038	1.16	1.02	1.16	-1.06	***Pigm***	phosphatidylinositol glycan anchor biosynthesis, class M	0006506 // GPI anchor biosynthetic process // not recorded///0097502 // mannosylatior
NM_0110	0.0083			-1.02	-1.57	1.16	-1.17	***Piga***	phosphatidylinositol glycan anchor biosynthesis, class A	0006506 // GPI anchor biosynthetic process // inferred from mutant phenotype///0009
NM_025820		0.01		1.02	1.12	1.16	1.08	***Crnkl1***	Crn, crooked neck-like 1 (Drosophila)	0000245 // spliceosomal complex assembly // not recorded///0000398 // mRNA splicing,
NM_001136086///		0.024		-1.08	-1.07	1.16	1.10	***Dpysl3***	dihydropyrimidinase-like 3	0007399 // nervous system development // inferred from direct assay///0008152 // met
NM_0254	0.0086			-1.08	1.04	1.16	1.13	***Mrps14***	mitochondrial ribosomal protein S14	0006412 // translation // not recorded
NM_001277309///		0.036		-1.06	-1.00	1.16	1.08	***Mea1***	male enhanced antigen 1	0007275 // multicellular organismal development // inferred from electronic annotation/
NM_172410///XM		0.031		1.02	1.13	1.16	1.20	***Nup93***	nucleoporin 93	0002230 // positive regulation of defense response to virus by host // not recorded///00
NM_0107	0.0281			-1.01	1.10	1.16	1.03	***Lmnb1***	lamin B1	0031662 // positive regulation of cyclin-dependent protein serine/threonine kinase activi
NM_0010	0.0055			1.01	1.19	1.16	1.22	***Capzb***	capping protein (actin filament) muscle Z-line, beta	0000902 // cell morphogenesis // not recorded///0007010 // cytoskeleton organization /
NM_026141///XM		0.024	0.016	-1.11	1.02	1.16	1.20	***Ppil4***	peptidylprolyl isomerase (cyclophilin)-like 4	0000413 // protein peptidyl-prolyl isomerization // not recorded///0006457 // protein fo
NM_0260	0.0262	0.027		-1.10	1.06	1.16	-1.06	***Cmtm6***	CKLF-like MARVEL transmembrane domain containing 6	0006935 // chemotaxis // inferred from electronic annotation
NM_183106///XM		0.014		-1.02	1.06	1.16	1.07	***Ttc17***	tetratricopeptide repeat domain 17	0030030 // cell projection organization // inferred from electronic annotation///0030041
NM_001164677///		0.004		1.04	1.02	1.16	1.04	***Pdcd6ip***	programmed cell death 6 interacting protein	0000920 // cell separation after cytokinesis // not recorded///0006810 // transport // inf
NM_0081	0.0163			1.04	-1.11	1.16	1.48	***Gstm2***	glutathione S-transferase, mu 2	0006749 // glutathione metabolic process // not recorded///0008152 // metabolic proce
NM_0012	0.0023	0.041		1.01	1.21	1.16	1.08	***Gabpb1***	GA repeat binding protein, beta 1	0006351 // transcription, DNA-templated // inferred from electronic annotation///00063
NM_001277266///		0.05		-1.05	1.09	1.16	1.15	***Adam17***	a disintegrin and metallopeptidase domain 17	0001666 // response to hypoxia // not recorded///0001934 // positive regulation of prot
NM_008948		0.003		-1.09	1.05	1.16	1.17	***Psmc3***	proteasome (prosome, macropain) 26S subunit, ATPase 3	0001824 // blastocyst development // inferred from mutant phenotype///0030163 // pro
NM_0119	######			-1.01	1.32	1.16	1.04	***Tlr2***	toll-like receptor 2	0001774 // microglial cell activation // not recorded///0002224 // toll-like receptor signa
NM_0013	0.0082	0.045		-1.05	1.14	1.16	1.06	***Rps27l***	ribosomal protein S27-like	0000028 // ribosomal small subunit assembly // ---///0006281 // DNA repair // not recor
NM_001038612///		0.003		-1.02	1.03	1.16	1.05	***Olfm1***	olfactomedin 1	0003190 // atrioventricular valve formation // inferred from sequence or structural simila
NM_0206	0.013	0.037		-1.08	1.06	1.16	1.18	***Smarce1***	SWI/SNF related, matrix associated, actin dependent regulator of chromatin, subfamil	0006337 // nucleosome disassembly // not recorded///0006338 // chromatin remodeling
NM_025828		0.012		-1.08	-1.02	1.16	1.09	***Lman2***	lectin, mannose-binding 2	0006810 // transport // inferred from electronic annotation///0006890 // retrograde ves
NM_025679		0.034		1.04	1.04	1.16	1.04	***Sike1***	suppressor of IKBKE 1	
NM_013492///XM		0.005	0.003	-1.10	1.02	1.17	1.33	***Clu***	clusterin	0000902 // cell morphogenesis // not recorded///0001774 // microglial cell activation //
NM_008303		0.031		-1.47	-1.56	1.17	-1.23	***Hspe1***	heat shock protein 1 (chaperonin 10)	0001649 // osteoblast differentiation // not recorded///0006457 // protein folding // infe
NM_0206	0.0028			1.03	1.28	1.17	1.07	***Taf1b***	TATA box binding protein (Tbp)-associated factor, RNA polymerase I, B	0001189 // RNA polymerase I transcriptional preinitiation complex assembly at the prom
NM_0081	0.043			1.26	1.47	1.17	1.23	***H1f0***	H1 histone family, member 0	0006334 // nucleosome assembly // inferred from electronic annotation
NM_0119	0.0091			1.13	1.46	1.17	1.00	***Nubp1***	nucleotide binding protein 1	0006879 // cellular iron ion homeostasis // not recorded///0010826 // negative regulatio
NM_001316716///		0.023		-1.02	-1.15	1.17	1.20	***Ddo***	D-aspartate oxidase	0006531 // aspartate metabolic process // inferred from mutant phenotype///0006533 /
NM_133653///XM_006518			0.037	1.03	1.11	1.17	1.31	***Mat1a***	methionine adenosyltransferase I, alpha	0006556 // S-adenosylmethionine biosynthetic process // not recorded///0006730 // one
NM_008434///XM		0.026		-1.01	1.05	1.17	1.03	***Kcnq1***	potassium voltage-gated channel, subfamily Q, member 1	0002230 // positive regulation of defense response to virus by host // not recorded///00
NM_0011	0.0266			1.07	1.37	1.17	1.21	***Lgals3***	lectin, galactose binding, soluble 3	0001501 // skeletal system development // inferred from genetic interaction///0002376
NM_031256///XM		0.025		-1.01	1.00	1.17	-1.08	***Plekha3***	pleckstrin homology domain-containing, family A (phosphoinositide binding specific) member 3	
NM_001313906///		0.003		-1.11	1.02	1.17	1.36	***Prmt5***	protein arginine N-methyltransferase 5	0000122 // negative regulation of transcription from RNA polymerase II promoter // infer
NM_0261	0.0298			-1.04	1.05	1.17	1.04	***Med4***	mediator complex subunit 4	0006351 // transcription, DNA-templated // inferred from electronic annotation///00063
NM_016752///XM		0.015		-1.21	1.04	1.17	1.13	***Slc35b1***	solute carrier family 35, member B1	0006810 // transport // inferred from electronic annotation///0008643 // carbohydrate t
NM_0218	0.0019			1.06	1.38	1.17	1.11	***Fads3***	fatty acid desaturase 3	0006629 // lipid metabolic process // inferred from electronic annotation///0006631 // f
NM_0223	######			-1.08	1.28	1.17	1.06	***Ctsz***	cathepsin Z	0006508 // proteolysis // inferred from electronic annotation///0051603 // proteolysis in
NM_1535	######	0.011	0.013	-1.17	1.15	1.17	-1.10	***Pilra***	paired immunoglobin-like type 2 receptor alpha	0007165 // signal transduction // not recorded///0007169 // transmembrane receptor p
NM_008569///XM		0.035		1.05	1.07	1.17	1.11	***Anapc1***	anaphase promoting complex subunit 1	0007049 // cell cycle // inferred from electronic annotation///0007067 // mitotic nuclear
NM_011192///XM		0.025		1.04	1.14	1.17	1.12	***Psme3***	proteaseome (prosome, macropain) activator subunit 3 (PA28 gamma, Ki)	0002479 // antigen processing and presentation of exogenous peptide antigen via MHC c
NM_0013	0.011			1.02	1.78	1.17	-1.01	***Il6***	interleukin 6	0001781 // neutrophil apoptotic process // inferred from direct assay///0001781 // neut
NM_0275	0.0108			1.06	1.30	1.17	1.05	***P2ry12***	purinergic receptor P2Y, G-protein coupled 12	0001973 // adenosine receptor signaling pathway // not recorded///0006930 // substrat
NM_0010	0.0316			1.11	1.76	1.17	-1.08	***Fam65b***	family with sequence similarity 65, member B	0007605 // sensory perception of sound // not recorded
NM_0103	0.0371			-1.01	1.16	1.17	1.03	***Emb***	embigin	0007155 // cell adhesion // not recorded///0035879 // plasma membrane lactate transp
NM_011937///NR_028061			0.031	-1.06	1.11	1.17	1.15	***Gm8615///Gnpda1***	glucosamine-6-phosphate deaminase 1 pseudogene///glucosamine-6-phosphate dear	0005975 // carbohydrate metabolic process // inferred from electronic annotation///000
NM_010887			0.034	1.01	1.01	1.17	1.04	***Ndufs4***	NADH dehydrogenase (ubiquinone) Fe-S protein 4	0001932 // regulation of protein phosphorylation // not recorded///0006810 // transpor
NM_0083	0.0382			-1.03	1.72	1.17	1.02	***Il1b***	interleukin 1 beta	0000122 // negative regulation of transcription from RNA polymerase II promoter // not
NM_001039718///		0.01		1.03	1.12	1.18	-1.01	***Cntf///Zfp91///Zfp91Cntf***	ciliary neurotrophic factor///zinc finger protein 91///Zfp91-Cntf readthrough transcrip	0007250 // activation of NF-kappaB-inducing kinase activity // not recorded///0007259 /
NM_001291448///		0.038		1.01	1.08	1.18	-1.00	***Foxred1***	FAD-dependent oxidoreductase domain containing 1	0006810 // transport // inferred from electronic annotation///0032981 // mitochondrial
NM_026423///XM_006531			0.013	1.18	1.20	1.18	-1.23	***Mri1***	methylthioribose-1-phosphate isomerase homolog (S. cerevisiae)	0008652 // cellular amino acid biosynthetic process // inferred from electronic annotatio
NM_0258	0.0205	0.004	0.012	-1.12	1.10	1.18	1.14	***Psmd12***	proteasome (prosome, macropain) 26S subunit, non-ATPase, 12	0043161 // proteasome-mediated ubiquitin-dependent protein catabolic process // not r
NM_001252332///		0.033		1.05	1.02	1.18	1.16	***8-Sep***	septin 8	
NM_027994			0.045	-1.07	1.02	1.18	1.26	***Cand1***	cullin associated and neddylation disassociated 1	0010265 // SCF complex assembly // not recorded///0016567 // protein ubiquitination //
NM_0119	0.0484			-1.03	1.17	1.18	-1.01	***Psma1***	proteasome (prosome, macropain) subunit, alpha type 1	0002376 // immune system process // inferred from electronic annotation///0002862 //

RefSeq Tr	*p* 24h	*p* 48h	*p* 72h	FD Air	FD 24h	FD 48h	FD 72h	Gene Symbol	Gene Title	Gene Ontology Biological Process
NM_144902///XM_		0.012		-1.01	1.04	1.18	1.02	***Slc35a3***	solute carrier family 35 (UDP-N-acetylglucosamine (UDP-GlcNAc) transporter), membe	0006810 // transport // inferred from electronic annotation///0008643 // carbohydrate t
NM_0012	0.032			1.11	-1.28	1.18	1.06	***Nnat***	neuronatin	0007275 // multicellular organismal development // inferred from electronic annotation/
NM_001253700///		0.034	0.04	-1.03	1.06	1.18	1.25	***Elp5***	elongator acetyltransferase complex subunit 5	0006351 // transcription, DNA-templated // inferred from electronic annotation///00063
NM_0012	0.0193	0.023	0.025	-1.10	1.10	1.18	1.09	***Txndc5***	thioredoxin domain containing 5	0006457 // protein folding // not recorded///0008152 // metabolic process // inferred fr
NM_0012	0.0066			1.01	1.24	1.18	1.14	***Casp3***	caspase 3	0001666 // response to hypoxia // inferred from electronic annotation///0001782 // B ce
NM_026913///XM_		0.008		-1.06	1.06	1.18	1.11	***Mitd1***	MIT, microtubule interacting and transport, domain containing 1	0000281 // mitotic cytokinesis // not recorded///0000920 // cell separation after cytokin
NM_133933		0.013		-1.17	-1.06	1.18	-1.12	***Rpn1***	ribophorin I	0006486 // protein glycosylation // inferred from electronic annotation///0018279 // pro
NM_1531	0.0429			1.23	1.63	1.18	1.04	***Gimap6***	GTPase, IMAP family member 6	
NM_183225///XM_		0.011		1.01	1.06	1.18	1.10	***Usp24***	ubiquitin specific peptidase 24	0006508 // proteolysis // inferred from electronic annotation///0006511 // ubiquitin-dep
NM_001008497///NM_00			0.028	-1.04	1.05	1.18	1.16	***P2ry14***	purinergic receptor P2Y, G-protein coupled, 14	0006955 // immune response // not recorded///0007165 // signal transduction // inferre
NM_0012	0.0033			-1.04	1.45	1.18	1.12	***Cd84***	CD84 antigen	0007155 // cell adhesion // inferred from electronic annotation
NM_0169	0.0264			1.04	1.23	1.18	1.08	***Sec61a1***	Sec61 alpha 1 subunit (S. cerevisiae)	0006614 // SRP-dependent cotranslational protein targeting to membrane // not recorde
NM_0010	0.0278			1.00	1.10	1.18	1.15	***Pdxdc1***	pyridoxal-dependent decarboxylase domain containing 1	0019752 // carboxylic acid metabolic process // inferred from electronic annotation
NM_001290368///		0.029		1.00	-1.03	1.18	1.12	***Nmt2***	N-myristoyltransferase 2	0006499 // N-terminal protein myristoylation // traceable author statement///0018008 /
NM_001164197///		0.01		-1.08	-1.03	1.18	1.03	***Mmp19***	matrix metallopeptidase 19	0001525 // angiogenesis // inferred from electronic annotation///0001541 // ovarian foll
NM_1339	0.0389			-1.03	1.13	1.18	1.00	***Lsm8***	LSM8 homolog, U6 small nuclear RNA associated (S. cerevisiae)	0000398 // mRNA splicing, via spliceosome // not recorded///0006397 // mRNA processi
NM_0079	0.0412			-1.01	1.21	1.18	1.00	***Fbl///LOC102643269***	fibrillarin///rRNA 2'-O-methyltransferase fibrillarin	0000494 // box C/D snoRNA 3'-end processing // not recorded///0001649 // osteoblast d
NM_0135	0.0155	0.04		-1.07	1.12	1.18	1.21	***Emg1***	EMG1 nucleolar protein homolog (S. cerevisiae)	0001824 // blastocyst development // inferred from mutant phenotype///0006364 // rRI
NM_0010	0.0051			1.04	1.53	1.18	1.20	***Gm10052///Gm5643///Gm5803///Hnrnpa***	heterogeneous nuclear ribonucleoprotein A1 pseudogene///predicted gene 5803///he	0000380 // alternative mRNA splicing, via spliceosome // inferred from direct assay///000
NM_0260	0.0485	0.042		-1.01	1.13	1.18	1.15	***Med31***	mediator complex subunit 31	0006351 // transcription, DNA-templated // inferred from electronic annotation///00063
NM_001314041///		0.002	0.049	-1.08	1.03	1.18	1.21	***Itga5***	integrin alpha 5 (fibronectin receptor alpha)	0007044 // cell-substrate junction assembly // inferred from direct assay///0007155 // ce
NM_013625///NR_		0.046		-1.02	1.08	1.18	1.11	***Pafah1b1***	platelet-activating factor acetylhydrolase, isoform 1b, subunit 1	0000132 // establishment of mitotic spindle orientation // not recorded///0000226 // mi
NM_0113	0.0093			-1.04	1.26	1.18	1.09	***Gm7285///Rps7***	predicted gene 7285///ribosomal protein S7	0001843 // neural tube closure // inferred from mutant phenotype///0006364 // rRNA p
NM_0012	0.0423	0.007	0.002	-1.01	1.22	1.18	1.18	***Clip4***	CAP-GLY domain containing linker protein family, member 4	
NM_1722	0.0014	0.026		-1.05	1.18	1.18	1.07	***Eif2b5***	eukaryotic translation initiation factor 2B, subunit 5 epsilon	0001541 // ovarian follicle development // not recorded///0006412 // translation // infer
NM_001204165///		0.043		1.00	-1.05	1.18	1.28	***Rapgef4***	Rap guanine nucleotide exchange factor (GEF) 4	0001932 // regulation of protein phosphorylation // inferred from electronic annotation/
NM_0098	######			1.04	2.32	1.18	-1.02	***Ch25h***	cholesterol 25-hydroxylase	0006629 // lipid metabolic process // inferred from electronic annotation///0006633 // f
NM_0011	0.0031			1.36	-1.13	1.18	1.61	***Pfkfb3***	6-phosphofructo-2-kinase/fructose-2,6-biphosphatase 3	0006000 // fructose metabolic process // inferred from electronic annotation///0006003
NM_0079	0.0332			1.00	1.16	1.19	1.09	***Aimp1***	aminoacyl tRNA synthetase complex-interacting multifunctional protein 1	0001525 // angiogenesis // inferred from electronic annotation///0001937 // negative re
NM_0011	0.0043			1.01	1.11	1.19	1.26	***Rnf14***	ring finger protein 14	0000209 // protein polyubiquitination // not recorded///0006351 // transcription, DNA-t
NM_0013	0.0088			-1.05	1.57	1.19	1.14	***Arhgdib***	Rho, GDP dissociation inhibitor (GDI) beta	0008152 // metabolic process // inferred from electronic annotation///0043547 // positiv
NM_008775///XM_		0.019		-1.06	1.00	1.19	1.08	***Pafah1b2***	platelet-activating factor acetylhydrolase, isoform 1b, subunit 2	0006629 // lipid metabolic process // inferred from electronic annotation///0006928 // n
NM_018856///NM_20767			0.039	1.23	1.10	1.19	1.11	***Ccnl2***	cyclin L2	0000079 // regulation of cyclin-dependent protein serine/threonine kinase activity // infe
NM_007743		0.039		-1.26	-1.12	1.19	1.01	***Col1a2***	collagen, type I, alpha 2	0001501 // skeletal system development // not recorded///0001568 // blood vessel deve
NM_011809			0.042	1.17	1.13	1.19	1.07	***Ets2***	E26 avian leukemia oncogene 2, 3' domain	0000122 // negative regulation of transcription from RNA polymerase II promoter // not r
NM_001159908///		0.018		1.05	-1.05	1.19	1.09	***Zfand2a***	zinc finger, AN1-type domain 2A	0032436 // positive regulation of proteasomal ubiquitin-dependent protein catabolic pro
NM_0084	0.0016			1.26	1.90	1.19	1.03	***Junb***	jun B proto-oncogene	0000122 // negative regulation of transcription from RNA polymerase II promoter // not r
NM_008065///XM_		0.018	0.018	-1.02	1.03	1.19	1.14	***Gabpa***	GA repeat binding protein, alpha	0000122 // negative regulation of transcription from RNA polymerase II promoter // infer
NM_0194	0.0402			1.03	1.23	1.19	1.19	***Myo10***	myosin X	0006810 // transport // inferred from electronic annotation///0007165 // signal transduc
NM_153774///XM_		0.005	0.039	-1.15	-1.03	1.19	1.14	***Ipo9***	importin 9	0006606 // protein import into nucleus // inferred from direct assay///0006610 // riboso
NM_0011	0.0011			-1.23	-2.44	1.19	-1.16	***Dnaja1***	DnaJ (Hsp40) homolog, subfamily A, member 1	0006457 // protein folding // inferred from electronic annotation///0007283 // spermato
NM_0109	0.0257	0.041		-1.02	1.20	1.19	1.01	***Mrpl40***	mitochondrial ribosomal protein L40	
NM_0075	0.0311			-1.01	1.22	1.19	-1.04	***Atp1b3***	ATPase, Na+/K+ transporting, beta 3 polypeptide	0006810 // transport // inferred from electronic annotation///0006811 // ion transport /
NM_0119	0.0135			1.03	1.18	1.19	1.10	***Cops3***	COP9 (constitutive photomorphogenic) homolog, subunit 3 (Arabidopsis thaliana)	0001701 // in utero embryonic development // inferred from mutant phenotype///00065
NM_1455	0.0196			-1.04	1.20	1.19	1.08	***Dars***	aspartyl-tRNA synthetase	0006412 // translation // inferred from electronic annotation///0006418 // tRNA aminoa
NM_0012	######	0.041	0.002	-1.19	1.13	1.19	1.09	***Asnsd1***	asparagine synthetase domain containing 1	0006529 // asparagine biosynthetic process // not recorded///0006541 // glutamine met
NM_0012	0.0022			1.00	1.24	1.19	-1.05	***Psma7***	proteasome (prosome, macropain) subunit, alpha type 7	0006508 // proteolysis // inferred from electronic annotation///0006511 // ubiquitin-dep
NM_172924///XM_		0.02		1.06	1.09	1.19	-1.05	***Peak1***	pseudopodium-enriched atypical kinase 1	0006468 // protein phosphorylation // not recorded///0016310 // phosphorylation // inf
NM_0010	0.05			1.04	1.31	1.19	1.09	***Adar***	adenosine deaminase, RNA-specific	0001649 // osteoblast differentiation // inferred from mutant phenotype///0001701 // in
NM_009763///XM_		0.009	0.003	-1.22	1.02	1.19	1.08	***Bst1***	bone marrow stromal cell antigen 1	0008152 // metabolic process // not recorded///0030890 // positive regulation of B cell p
NM_001080932///		0.004		-1.05	-1.09	1.19	1.11	***Foxk2***	forkhead box K2	0006351 // transcription, DNA-templated // inferred from electronic annotation///00063
NM_0010	######	0.004		-1.06	1.11	1.19	1.34	***Banf1***	barrier to autointegration factor 1	0015074 // DNA integration // inferred from direct assay///0075713 // establishment of i
NM_145512		0.02		1.02	1.06	1.19	1.04	***Sft2d2***	SFT2 domain containing 2	0006810 // transport // inferred from electronic annotation///0015031 // protein transpo
NM_007898		0.017		1.04	1.07	1.19	1.09	***Ebp***	phenylalkylamine Ca2+ antagonist (emopamil) binding protein	0006629 // lipid metabolic process // inferred from electronic annotation///0006694 // s
NM_0013	0.0189			-1.20	1.29	1.19	1.05	***Alox5ap***	arachidonate 5-lipoxygenase activating protein	0002540 // leukotriene production involved in inflammatory response // inferred from m
NM_019833///XM_		0.031		1.04	1.05	1.20	1.13	***Fam69b***	family with sequence similarity 69, member B	
NM_026667		0.044		-1.04	-1.01	1.20	1.06	***Fam114a1***	family with sequence similarity 114, member A1	
NM_0012	0.0036			1.04	1.19	1.20	1.19	***Dnajc5***	DnaJ (Hsp40) homolog, subfamily C, member 5	0043524 // negative regulation of neuron apoptotic process // inferred from genetic inter
NM_0205	0.0433			-1.13	1.05	1.20	1.02	***Atp5j2***	ATP synthase, H+ transporting, mitochondrial F0 complex, subunit F2	0006754 // ATP biosynthetic process // inferred from electronic annotation///0006810 //
NM_134138///XM_		0.026		1.07	1.18	1.20	1.14	***Psmg2***	proteasome (prosome, macropain) assembly chaperone 2	0007094 // mitotic spindle assembly checkpoint // inferred from direct assay///0043066
NM_001311790///		0.032	0.036	-1.12	1.09	1.20	1.14	***Ctsc***	cathepsin C	0001913 // T cell mediated cytotoxicity // inferred from genetic interaction///0006508 //
NM_010485///XM_		0.033	0.015	-1.07	-1.02	1.20	1.15	***Elavl1***	ELAV (embryonic lethal, abnormal vision)-like 1 (Hu antigen R)	0006417 // regulation of translation // not recorded///0045727 // positive regulation of t
NM_008255///XM_		0.024		1.05	1.18	1.20	1.07	***Hmgcr***	3-hydroxy-3-methylglutaryl-Coenzyme A reductase	0006629 // lipid metabolic process // inferred from electronic annotation///0006694 // s
NM_0114	0.0163	0.011		-1.00	1.09	1.20	1.20	***LOC100862468///Nhp2l1***	NHP2-like protein 1///NHP2 non-histone chromosome protein 2-like 1 (S. cerevisiae)	0006397 // mRNA processing // inferred from electronic annotation///0007338 // single

RefSeq Tr	p 24h	p 48h	p 72h	FD Air	FD 24h	FD 48h	FD 72h	Gene Symbol	Gene Title	Gene Ontology Biological Process
NM_0116	0.0274			1.04	1.23	1.20	1.04	***Ugcg***	UDP-glucose ceramide glucosyltransferase	0006629 // lipid metabolic process // inferred from electronic annotation///0006665 // s
NM_0306	0.0134			1.04	1.29	1.20	-1.07	***Serp1***	stress-associated endoplasmic reticulum protein 1	0001501 // skeletal system development // inferred from mutant phenotype///0006006 ,
NM_001080974///		0.045		-1.02	1.07	1.20	1.21	***Sri***	sorcin	0006508 // proteolysis // not recorded///0006816 // calcium ion transport // inferred fro
NM_0011	0.0225			1.02	1.24	1.20	1.01	***Hmbs***	hydroxymethylbilane synthase	0001666 // response to hypoxia // inferred from electronic annotation///0006779 // por
NM_001289770///		0.043	0.006	-1.04	1.01	1.20	1.09	***Exoc1***	exocyst complex component 1	0006810 // transport // inferred from electronic annotation///0006887 // exocytosis // n
NM_025664		0.018		1.00	1.15	1.20	-1.02	***Snx9***	sorting nexin 9	0000281 // mitotic cytokinesis // not recorded///0006810 // transport // inferred from el
NM_1451	0.0133	0.018	0.018	-1.07	1.22	1.20	1.22	***Lpcat3***	lysophosphatidylcholine acyltransferase 3	0006629 // lipid metabolic process // inferred from electronic annotation///0006644 // p
NM_0010	0.0352			1.00	1.16	1.20	1.05	***Trappc1***	trafficking protein particle complex 1	0006810 // transport // inferred from electronic annotation///0006888 // ER to Golgi ves
NM_001111043///		0.011		-1.42	-1.57	1.20	1.02	***Serpinh1***	serine (or cysteine) peptidase inhibitor, clade H, member 1	0003433 // chondrocyte development involved in endochondral bone morphogenesis //
NM_019953///XM		0.049		1.02	1.09	1.20	1.03	***Cnpy2***	canopy 2 homolog (zebrafish)	0010629 // negative regulation of gene expression // inferred from mutant phenotype///
NM_011149		0.038		-1.02	1.05	1.20	1.05	***Ppib***	peptidylprolyl isomerase B	0000413 // protein peptidyl-prolyl isomerization // not recorded///0006457 // protein fo
NM_0167	0.0022	0.04		-1.08	1.10	1.20	1.14	***Hsd17b10***	hydroxysteroid (17-beta) dehydrogenase 10	0007569 // cell aging // inferred from electronic annotation///0008033 // tRNA processir
NM_0268	0.0066			1.03	1.25	1.20	1.23	***Chmp2a***	charged multivesicular body protein 2A	0000920 // cell separation after cytokinesis // not recorded///0006810 // transport // inf
NM_001037997///		0.019		1.02	1.02	1.20	-1.01	***Fer***	fer (fms/fps related) protein kinase	0000226 // microtubule cytoskeleton organization // not recorded///0000278 // mitotic
NM_0255	######			-1.09	1.09	1.20	1.21	***Cyb5b***	cytochrome b5 type B	0006810 // transport // inferred from electronic annotation///0043085 // positive regula
NM_001166480///		0.042		-1.00	1.12	1.20	1.14	***Ssr4***	signal sequence receptor, delta	
NM_0083	0.0077	0.017		-1.08	1.38	1.20	1.00	***Itga7***	integrin alpha 7	0007155 // cell adhesion // inferred from mutant phenotype///0007155 // cell adhesion
NM_176979///XM		0.008		-1.08	1.03	1.21	1.06	***Topbp1***	topoisomerase (DNA) II binding protein 1	0006281 // DNA repair // inferred from electronic annotation///0006974 // cellular respo
NM_1391	0.038			-1.01	1.85	1.21	1.44	***Plac8***	placenta-specific 8	0008284 // positive regulation of cell proliferation // inferred from direct assay///00094C
NM_001290725///		0.044		1.03	1.04	1.21	1.18	***Ppp1r8***	protein phosphatase 1, regulatory (inhibitor) subunit 8	0006351 // transcription, DNA-templated // inferred from electronic annotation///00063
NM_010481		0.045		-1.00	1.07	1.21	-1.06	***Hspa9***	heat shock protein 9	0006457 // protein folding // inferred from electronic annotation///0006611 // protein e
NM_001136059///		0.021		-1.67	-1.64	1.21	-1.57	***Cyp1a1***	cytochrome P450, family 1, subfamily a, polypeptide 1	0001666 // response to hypoxia // inferred from electronic annotation///0001889 // liver
NM_0116	0.0104			-1.11	1.65	1.21	-1.15	***Tyrobp***	TYRO protein tyrosine kinase binding protein	0002281 // macrophage activation involved in immune response // inferred from mutant
NM_1340	0.0261	0.002		-1.10	1.11	1.21	-1.11	***Kdelr3***	KDEL (Lys-Asp-Glu-Leu) endoplasmic reticulum protein retention receptor 3	0006621 // protein retention in ER lumen // inferred from electronic annotation///00068
NM_0012	0.0351	0.002		-1.08	1.05	1.21	1.04	***Pus7***	pseudouridylate synthase 7 homolog (S. cerevisiae)	0001522 // pseudouridine synthesis // inferred from electronic annotation///0001522 //
NM_0011	0.0083			-1.05	1.29	1.21	1.28	***Fxyd5***	FXYD domain-containing ion transport regulator 5	0006810 // transport // inferred from electronic annotation///0006811 // ion transport /
NM_008083		0.024		-1.26	1.02	1.21	-1.12	***Gap43***	growth associated protein 43	0007275 // multicellular organismal development // inferred from electronic annotation/
NM_0205	0.0195	0.007	0.022	-1.44	1.37	1.21	1.04	***Retnla***	resistin like alpha	
NM_023065		0.003		-1.03	1.16	1.21	1.01	***Ifi30***	interferon gamma inducible protein 30	0002376 // immune system process // inferred from electronic annotation///0019886 //
NM_0254	0.0424	0.028		-1.01	1.17	1.21	1.08	***Llph***	LLP homolog, long-term synaptic facilitation (Aplysia)	
NM_0117	0.0062	0.047		1.00	1.15	1.21	1.43	***Coro1c***	coronin, actin binding protein 1C	0000147 // actin cortical patch assembly // traceable author statement///0001755 // neu
NM_0104	0.0375			1.02	1.23	1.21	1.03	***Hsd17b7***	hydroxysteroid (17-beta) dehydrogenase 7	0006629 // lipid metabolic process // inferred from electronic annotation///0006694 // s
NM_011284		0.034		1.06	1.10	1.21	1.17	***Rpa2***	replication protein A2	0000723 // telomere maintenance // not recorded///0000724 // double-strand break rep
NM_0253	0.0468			1.03	1.34	1.21	1.03	***Aaed1***	AhpC/TSA antioxidant enzyme domain containing 1	
NM_016737		0.038		-1.54	-1.95	1.21	1.28	***Stip1***	stress-induced phosphoprotein 1	
NM_001146222///		0.029		-1.05	1.05	1.21	1.11	***Dpy30***	dpy-30 homolog (C. elegans)	0006348 // chromatin silencing at telomere // not recorded///0006351 // transcription, D
NM_010158///XM		0.033		-1.05	1.13	1.21	1.14	***Khdrbs3***	KH domain containing, RNA binding, signal transduction associated 3	0006351 // transcription, DNA-templated // inferred from electronic annotation///00063
NM_026330///XM		0.005		-1.04	1.14	1.21	1.08	***Nsmce1***	non-SMC element 1 homolog (S. cerevisiae)	0000724 // double-strand break repair via homologous recombination // not recorded///
NM_0266	0.0136			-1.05	1.19	1.21	1.28	***Fam96a***	family with sequence similarity 96, member A	0007059 // chromosome segregation // not recorded///0016226 // iron-sulfur cluster ass
NM_023397///NR		0.01		1.06	1.08	1.21	1.18	***Mdp1***	magnesium-dependent phosphatase 1	0006470 // protein dephosphorylation // inferred from electronic annotation///0016311
NM_001113358///		0.016		-1.12	-1.02	1.21	1.13	***Dad1***	defender against cell death 1	0001824 // blastocyst development // inferred from mutant phenotype///0006486 // pro
NM_0254	0.0209			1.02	1.28	1.21	-1.10	***Mrps28***	mitochondrial ribosomal protein S28	
NM_009007///XM		0.039		1.01	1.14	1.21	1.02	***Rac1***	RAS-related C3 botulinum substrate 1	0001934 // positive regulation of protein phosphorylation // not recorded///0002093 //
NM_022018///XM		0.042		1.06	1.03	1.21	1.18	***Fam129a***	family with sequence similarity 129, member A	0001933 // negative regulation of protein phosphorylation // inferred from mutant phen
NM_0252	0.0146			-1.03	1.21	1.21	1.29	***Tbrg1***	transforming growth factor beta regulated gene 1	0006260 // DNA replication // not recorded///0006260 // DNA replication // inferred fro
NM_0012	0.0419			1.02	1.24	1.22	1.28	***Ramp2///Vps25***	receptor (calcitonin) activity modifying protein 2///vacuolar protein sorting 25 (yeast)	0001525 // angiogenesis // not recorded///0001570 // vasculogenesis // not recorded///
NM_0104	######			1.12	1.87	1.22	-1.19	***Ier2***	immediate early response 2	0030182 // neuron differentiation // not recorded///0048870 // cell motility // not recor
NM_0084	0.0308			-1.19	1.08	1.22	1.11	***Lgals1***	lectin, galactose binding, soluble 1	0002317 // plasma cell differentiation // inferred from direct assay///0006915 // apopto
NM_1814	0.0403			1.14	-1.27	1.22	1.10	***Gm15583///Zbed4***	predicted gene 15583///zinc finger, BED type containing 4	
NM_001172062///		0.011		-1.04	1.03	1.22	1.09	***Lman1***	lectin, mannose-binding, 1	0006810 // transport // inferred from electronic annotation///0006888 // ER to Golgi ves
NM_001166368///		0.017		-1.21	1.05	1.22	1.27	***Fkbp2***	FK506 binding protein 2	0000413 // protein peptidyl-prolyl isomerization // not recorded///0006457 // protein fo
NM_145851///XM_00650(			0.013	1.12	1.19	1.22	1.05	***Cables2***	CDK5 and Abl enzyme substrate 2	0007049 // cell cycle // inferred from electronic annotation///0051301 // cell division // i
NM_008704		0.034		-1.05	1.10	1.22	1.02	***Nme1***	NME/NM23 nucleoside diphosphate kinase 1	0002762 // negative regulation of myeloid leukocyte differentiation // not recorded///00
NM_027959		0.004		-1.30	-1.12	1.22	1.09	***Pdia6***	protein disulfide isomerase associated 6	0006457 // protein folding // not recorded///0008152 // metabolic process // inferred fro
NM_054044///XM		0.036		-1.03	-1.01	1.22	-1.00	***Adgra2***	adhesion G protein-coupled receptor A2	0001525 // angiogenesis // inferred from mutant phenotype///0002040 // sprouting ang
NM_0010	0.045			1.18	1.66	1.22	1.41	***Elovl1***	elongation of very long chain fatty acids (FEN1/Elo2, SUR4/Elo3, yeast)-like 1	0006629 // lipid metabolic process // inferred from electronic annotation///0006631 // f
NM_0113	0.0245		0.014	-1.05	1.42	1.22	1.35	***Ccl11***	chemokine (C-C motif) ligand 11	0001938 // positive regulation of endothelial cell proliferation // not recorded///000254
NR_002840///NR_028543			0.043	1.56	1.70	1.22	-1.04	***Gas5///Snord47***	growth arrest specific 5///small nucleolar RNA, C/D box 47	
NM_001134692///		0.045		-1.01	1.06	1.22	1.22	***LOC102632739///Ost4***	dolichyl-diphosphooligosaccharide--protein glycosyltransferase subunit 4-like///oligosaccharyltransferase 4 homolog (S. cerevisiae)	
NM_0081	0.0473	0.034		1.03	1.25	1.22	1.05	***Glud1***	glutamate dehydrogenase 1	0006520 // cellular amino acid metabolic process // inferred from electronic annotation/
NM_0011	0.0394		0.007	1.01	1.09	1.22	1.22	***Dlg5***	discs, large homolog 5 (Drosophila)	0006461 // protein complex assembly // inferred from mutant phenotype///0030859 // p
NM_0101	0.0051	#####		-1.11	1.18	1.22	1.02	***F7***	coagulation factor VII	0002690 // positive regulation of leukocyte chemotaxis // not recorded///0006508 // pro
NM_018829///XM		0.048		1.04	1.08	1.22	1.15	***Ap3m1***	adaptor-related protein complex 3, mu 1 subunit	0006810 // transport // inferred from electronic annotation///0006886 // intracellular pr
NM_0119	0.0053	0.037		-1.09	1.15	1.22	1.13	***Psmb2***	proteasome (prosome, macropain) subunit, beta type 2	0006508 // proteolysis // inferred from electronic annotation///0010243 // response to c

RefSeq Tr	p 24h	p 48h	p 72h	FD Air	FD 24h	FD 48h	FD 72h	Gene Symbol	Gene Title	Gene Ontology Biological Process
NM_010941		0.009		-1.01	1.10	1.22	1.05	Nsdhl	NAD(P) dependent steroid dehydrogenase-like	0001942 // hair follicle development // inferred from mutant phenotype///0006629 // lip
NM_022324		0.003		-1.43	-1.20	1.22	-1.27	Sdf2l1	stromal cell-derived factor 2-like 1	0000032 // cell wall mannoprotein biosynthetic process // not recorded///0007275 // mu
NM_0010	0.0253			1.47	1.09	1.22	1.18	Pcdha1///Pcdha10///Pcdha11///Pcdha12/	protocadherin alpha 1///protocadherin alpha 10///protocadherin alpha 11///protoca	0007155 // cell adhesion // inferred from electronic annotation///0007155 // cell adhesi
NM_026532///NR_		0.04		-1.04	1.02	1.22	1.29	Nutf2///Nutf2-ps1///Nutf2-ps2	nuclear transport factor 2///nuclear transport factor 2, pseudogene 1///nuclear trans	0000060 // protein import into nucleus, translocation // not recorded///0006606 // prot
NM_0259	0.041			1.11	-1.15	1.23	1.01	Dnajb4	DnaJ (Hsp40) homolog, subfamily B, member 4	0006457 // protein folding // inferred from electronic annotation
NM_007426			0.005	1.20	1.20	1.23	-1.14	Angpt2	angiopoietin 2	0001525 // angiogenesis // inferred from genetic interaction///0001666 // response to h
NM_0012	0.0194			-1.13	1.25	1.23	1.15	Cd302	CD302 antigen	0006909 // phagocytosis // not recorded
NM_0079	######			1.17	4.07	1.23	1.25	Egr1	early growth response 1	0000122 // negative regulation of transcription from RNA polymerase II promoter // infer
NM_011234		0.045		-1.07	1.11	1.23	1.09	Rad51	RAD51 homolog	0000724 // double-strand break repair via homologous recombination // inferred from d
NM_0275	0.0083	#####	0.006	-1.13	1.07	1.23	1.17	Pvr	poliovirus receptor	0002860 // positive regulation of natural killer cell mediated cytotoxicity directed against
NM_0262	0.0135	0.008		-1.00	1.17	1.23	1.08	Tmed9	transmembrane emp24 protein transport domain containing 9	0006810 // transport // inferred from electronic annotation///0007030 // Golgi organizat
NM_0095	0.0315		0.02	-1.10	1.17	1.23	1.07	Xrcc5	X-ray repair complementing defective repair in Chinese hamster cells 5	0000237 // leptotene // inferred from mutant phenotype///0000723 // telomere mainte
NM_001113554///		0.004		-1.13	-1.00	1.23	1.12	Nudcd1	NudC domain containing 1	
NM_0103	0.0352	0.021		-1.06	1.18	1.23	1.10	Gusb	glucuronidase, beta	0005975 // carbohydrate metabolic process // inferred from direct assay///0006027 // gl
NM_0119	0.0322			-1.01	1.13	1.23	1.11	Psma5	proteasome (prosome, macropain) subunit, alpha type 5	0006508 // proteolysis // inferred from electronic annotation///0006511 // ubiquitin-dep
NM_0010	0.0302	0.01		1.01	1.18	1.23	1.16	Slc7a2	solute carrier family 7 (cationic amino acid transporter, y+ system), member 2	0002537 // nitric oxide production involved in inflammatory response // inferred from m
NM_175158		0.003		1.02	1.10	1.23	-1.00	Utp20	UTP20, small subunit (SSU) processome component, homolog (yeast)	0006364 // rRNA processing // not recorded
NM_0011	0.0144	0.041		-1.05	1.35	1.23	1.13	Max	Max protein	0006351 // transcription, DNA-templated // inferred from electronic annotation///00063
NM_028295///XR_		0.042		-1.01	-1.01	1.23	-1.03	Pdia5	protein disulfide isomerase associated 5	0006457 // protein folding // not recorded///0008152 // metabolic process // inferred fr
NM_001290541///NM_00			0.038	-1.28	-1.32	1.23	1.03	Acer2	alkaline ceramidase 2	0001953 // negative regulation of cell-matrix adhesion // not recorded///0006629 // lipic
NM_001029895///		0.007		1.05	1.11	1.23	1.16	Ate1	arginyltransferase 1	0016598 // protein arginylation // inferred from direct assay
NM_0075	0.0024			1.04	1.76	1.23	1.16	Btg2	B cell translocation gene 2, anti-proliferative	0000122 // negative regulation of transcription from RNA polymerase II promoter // not r
NM_0114	0.0351			1.09	-1.10	1.23	1.18	Smarca2	SWI/SNF related, matrix associated, actin dependent regulator of chromatin, subfamil	0006325 // chromatin organization // traceable author statement///0006334 // nucleosc
NM_0012	0.0451			1.03	1.14	1.23	1.10	B3gnt9	UDP-GlcNAc:betaGal beta-1,3-N-acetylglucosaminyltransferase 9	0006486 // protein glycosylation // inferred from electronic annotation
NM_001190804///		0.019		-1.25	-1.06	1.23	-1.03	Dnajb11	DnaJ (Hsp40) homolog, subfamily B, member 11	0006457 // protein folding // inferred from electronic annotation///0016556 // mRNA m
NM_0010	0.0331			-1.11	1.18	1.23	1.14	Cd44	CD44 antigen	0001558 // regulation of cell growth // not recorded///0001658 // branching involved in
NM_007523///XR_		0.028		1.04	1.10	1.23	1.50	Bak1	BCL2-antagonist/killer 1	0001776 // leukocyte homeostasis // inferred from genetic interaction///0001782 // B ce
NM_0196	0.0122	0.049	0.035	-1.14	1.28	1.23	1.20	Hsd17b12	hydroxysteroid (17-beta) dehydrogenase 12	0006629 // lipid metabolic process // inferred from electronic annotation///0006633 // fa
NM_133859///XR_		0.01	0.006	-1.11	1.01	1.23	1.23	Olfml3///LOC269472	olfactomedin-like 3///uncharacterized LOC269472	0007275 // multicellular organismal development // inferred from electronic annotation
NM_0011	0.0223	0.043		-1.08	1.06	1.24	1.03	Galnt1	UDP-N-acetyl-alpha-D-galactosamine:polypeptide N-acetylgalactosaminyltransferase 1	0006486 // protein glycosylation // inferred from electronic annotation///0006493 // pro
NM_0215	0.0126			1.01	1.17	1.24	1.18	Rdh11	retinol dehydrogenase 11	0006281 // DNA repair // inferred from electronic annotation///0006281 // DNA repair /,
NM_0091	######			1.06	1.39	1.24	1.28	St3gal4	ST3 beta-galactoside alpha-2,3-sialyltransferase 4	0001574 // ganglioside biosynthetic process // not recorded///0006486 // protein glycos
NM_009274///XM_		0.037		1.01	1.06	1.24	1.30	Srpk2	serine/arginine-rich protein specific kinase 2	0000245 // spliceosomal complex assembly // inferred from direct assay///0000245 // sp
NM_001113246///		0.007		-1.09	1.05	1.24	1.14	Chn1	chimerin 1	0007165 // signal transduction // inferred from electronic annotation///0007399 // nerv
NM_145494///XM_		0.043		1.00	1.22	1.24	1.11	Me2	malic enzyme 2, NAD(+)-dependent, mitochondrial	0006108 // malate metabolic process // inferred from electronic annotation///0008152 /
NM_0110	0.0108			1.00	-1.08	1.24	1.13	Pea15a	phosphoprotein enriched in astrocytes 15A	0000077 // DNA damage checkpoint // inferred from electronic annotation///0006810 //
NM_001001493///XM_001			0.01	1.02	1.03	1.24	1.23	Gm2573///Wdr83os	predicted gene 2573///WD repeat domain 83 opposite strand	
NM_0011	0.0025	0.025		-1.01	1.19	1.24	1.16	Acp1///LOC102642088	acid phosphatase 1, soluble///low molecular weight phosphotyrosine protein phospha	0006470 // protein dephosphorylation // inferred from electronic annotation///0035335
NM_001033420///		0.006		-1.07	-1.02	1.24	1.03	Dock1	dedicator of cytokinesis 1	0002244 // hematopoietic progenitor cell differentiation // inferred from genetic interact
NM_001039351///		0.001		1.03	1.07	1.24	1.30	Nolc1	nucleolar and coiled-body phosphoprotein 1	0007000 // nucleolus organization // inferred from direct assay
NM_178111///XM_00650			0.031	-1.04	-1.03	1.24	1.21	Trp53inp2	transformation related protein 53 inducible nuclear protein 2	0000045 // autophagosome assembly // not recorded///0001649 // osteoblast differenti
NM_0215	0.0227			1.01	1.22	1.24	1.15	Smu1	smu-1 suppressor of mec-8 and unc-52 homolog (C. elegans)	
NM_019710///XM_		0.032		-1.09	1.03	1.24	1.22	Smc1a	structural maintenance of chromosomes 1A	0006281 // DNA repair // inferred from electronic annotation///0006974 // cellular respo
NM_181470///XM_		0.039		1.02	1.13	1.24	1.21	Ltv1	LTV1 homolog (S. cerevisiae)	
NM_0010	0.0249	0.014		-1.03	1.18	1.24	1.12	Ids	iduronate 2-sulfatase	0008152 // metabolic process // inferred from electronic annotation
NM_0119	0.005			-1.02	1.52	1.24	1.25	Abcd2	ATP-binding cassette, sub-family D (ALD), member 2	0000038 // very long-chain fatty acid metabolic process // not recorded///0006635 // fat
NM_0012	0.0079	0.004		-1.12	1.22	1.24	1.16	Fn1	fibronectin 1	0001525 // angiogenesis // inferred from electronic annotation///0001775 // cell activati
NM_008448		0.048		1.09	1.11	1.25	1.19	Kif5b	kinesin family member 5B	0006839 // mitochondrial transport // traceable author statement///0007017 // microtu
NM_0093	0.0328	0.031		-1.09	1.14	1.25	1.18	Tgfbr2	transforming growth factor, beta receptor II	0001569 // patterning of blood vessels // inferred from mutant phenotype///0001570 //
NM_0289	0.002			1.06	1.46	1.25	1.35	Polr3c	polymerase (RNA) III (DNA directed) polypeptide C	0002376 // immune system process // inferred from electronic annotation///0006351 //
NM_007591		0.018	0.022	-1.13	-1.11	1.25	1.22	Calr	calreticulin	0000122 // negative regulation of transcription from RNA polymerase II promoter // not r
NM_029701///XM_		0.029	0.043	-1.16	1.01	1.25	1.14	Spcs3	signal peptidase complex subunit 3 homolog (S. cerevisiae)	0006465 // signal peptide processing // inferred from electronic annotation
NM_001039089///		0.017		-1.02	1.02	1.25	1.12	Sel1l	sel-1 suppressor of lin-12-like (C. elegans)	0007219 // Notch signaling pathway // inferred from electronic annotation///0030970 //
NM_1460	0.0059	0.015		-1.05	1.14	1.25	-1.05	Clptm1l	CLPTM1-like	0006915 // apoptotic process // inferred from electronic annotation
NM_001297606///		0.027		1.04	1.21	1.25	1.32	Rad23a	RAD23a homolog (S. cerevisiae)	0006281 // DNA repair // inferred from electronic annotation///0006289 // nucleotide-e
NM_0158	0.0018			-1.09	1.08	1.25	1.19	Copb2	coatomer protein complex, subunit beta 2 (beta prime)	0006810 // transport // inferred from electronic annotation///0006886 // intracellular pr
NM_0012	0.0084			1.03	1.42	1.25	1.09	Esd	esterase D/formylglutathione hydrolase	0046294 // formaldehyde catabolic process // inferred from electronic annotation
NM_0010	0.0142			1.03	1.43	1.25	-1.05	Parl	presenilin associated, rhomboid-like	0006508 // proteolysis // inferred from genetic interaction///0006508 // proteolysis // in
NM_001277970///		0.01		-1.15	-1.22	1.25	1.12	Itga6	integrin alpha 6	0007155 // cell adhesion // inferred from electronic annotation///0007160 // cell-matrix
NM_0013	0.0321			1.05	1.19	1.25	1.06	Psma3	proteasome (prosome, macropain) subunit, alpha type 3	0006508 // proteolysis // inferred from electronic annotation///0006511 // ubiquitin-dep
NM_010104		0.016		-1.27	-1.17	1.25	-1.14	Edn1	endothelin 1	0000122 // negative regulation of transcription from RNA polymerase II promoter // not r
NM_001313758///		0.032		1.00	1.12	1.25	1.14	Prps2	phosphoribosyl pyrophosphate synthetase 2	0006015 // 5-phosphoribose 1-diphosphate biosynthetic process // not recorded///0006
NM_001161419///		0.016		-1.06	1.16	1.25	1.15	Atp5g1	ATP synthase, H+ transporting, mitochondrial F0 complex, subunit C1 (subunit 9)	0006810 // transport // inferred from electronic annotation///0006811 // ion transport /
NM_001290421///		0.015		1.06	1.14	1.25	1.31	Flna	filamin, alpha	0001837 // epithelial to mesenchymal transition // inferred from genetic interaction///00

RefSeq Tr	*p* 24h	*p* 48h	*p* 72h	FD Air	FD 24h	FD 48h	FD 72h	Gene Symbol	Gene Title	Gene Ontology Biological Process
NM_153806		0.037	0.02	-1.00	1.04	1.26	1.25	***Dnttip2***	deoxynucleotidyltransferase, terminal, interacting protein 2	0006351 // transcription, DNA-templated // inferred from electronic annotation///00063
NM_001035854///		0.037		1.00	1.22	1.26	1.05	***Ap2b1***	adaptor-related protein complex 2, beta 1 subunit	0003279 // cardiac septum development // inferred from mutant phenotype///0003281
NM_0011	######	0.003		-1.08	1.38	1.26	1.10	***Mal***	myelin and lymphocyte protein, T cell differentiation protein	0042552 // myelination // not recorded///1902043 // positive regulation of extrinsic apo
NM_001199177///		0.012		-1.05	1.11	1.26	1.04	***Opa1***	optic atrophy 1	0000002 // mitochondrial genome maintenance // not recorded///0001843 // neural tub
NM_025936///XM		0.031		-1.12	1.05	1.26	1.31	***Rars***	arginyl-tRNA synthetase	0006412 // translation // inferred from electronic annotation///0006418 // tRNA aminoa
NM_0531	0.0225			-1.05	1.16	1.26	1.45	***Echs1***	enoyl Coenzyme A hydratase, short chain, 1, mitochondrial	0006629 // lipid metabolic process // inferred from electronic annotation///0006631 // f
NM_021446///XM		0.025		1.04	1.13	1.26	1.27	***0610007P14Rik***	RIKEN cDNA 0610007P14 gene	0006629 // lipid metabolic process // inferred from electronic annotation///0006694 // s
NM_0078	0.0156	0.012		-1.12	1.13	1.26	-1.02	***Ddost***	dolichyl-di-phosphooligosaccharide-protein glycotransferase	0006486 // protein glycosylation // inferred from sequence or structural similarity///000
NM_0214	0.0122			1.14	1.57	1.26	1.17	***Stk4***	serine/threonine kinase 4	0000165 // MAPK cascade // ---///0000186 // activation of MAPKK activity // not recorde
NM_007802///XM		0.046		-1.09	1.08	1.26	1.04	***Ctsk***	cathepsin K	0001957 // intramembranous ossification // inferred from electronic annotation///00065
NM_0012	0.047			-1.06	1.24	1.26	-1.05	***Scarb1***	scavenger receptor class B, member 1	0001935 // endothelial cell proliferation // inferred from mutant phenotype///0006702 /
NM_001081635///		0.046		-1.02	-1.07	1.26	-1.05	***Ccnd3***	cyclin D3	0001934 // positive regulation of protein phosphorylation // not recorded///0007049 //
NM_0010	0.0032			1.03	1.48	1.26	1.21	***Gm10052///Gm5803///Hnrnpa1///Gm564***	heterogeneous nuclear ribonucleoprotein A1 pseudogene///predicted gene 5803///he	0000380 // alternative mRNA splicing, via spliceosome // inferred from direct assay///00
NM_0115	0.0105	0.02		1.08	1.30	1.26	1.15	***Sdc1***	syndecan 1	0001657 // ureteric bud development // inferred from electronic annotation///0006954
NM_026554		0.013		-1.03	-1.01	1.26	1.16	***Ncbp2***	nuclear cap binding protein subunit 2	0000184 // nuclear-transcribed mRNA catabolic process, nonsense-mediated decay // no
NM_024180///XM		0.032		-1.05	1.01	1.26	1.19	***Ormdl2***	ORM1-like 2 (S. cerevisiae)	0006672 // ceramide metabolic process // not recorded///0090155 // negative regulatio
NM_0012	0.0237			-1.05	1.15	1.26	1.16	***Immt***	inner membrane protein, mitochondrial	0042407 // cristae formation // not recorded///0051560 // mitochondrial calcium ion ho
NM_029578		0.036		-1.02	1.15	1.26	1.11	***Tgds***	TDP-glucose 4,6-dehydratase	0009225 // nucleotide-sugar metabolic process // inferred from electronic annotation
NM_019824		0.012		-1.19	1.09	1.26	1.17	***Arpc3***	actin related protein 2/3 complex, subunit 3	0030833 // regulation of actin filament polymerization // inferred from electronic annota
NM_133225		0.024		1.06	1.21	1.27	1.03	***Acbd3***	acyl-Coenzyme A binding domain containing 3	0006629 // lipid metabolic process // inferred from electronic annotation///0006694 // s
NM_009840		0.013		-1.18	-1.10	1.27	1.31	***Cct8***	chaperonin containing Tcp1, subunit 8 (theta)	0006457 // protein folding // inferred from electronic annotation///0007339 // binding o
NM_001177556///		0.003		-1.05	1.03	1.27	1.14	***Gng12***	guanine nucleotide binding protein (G protein), gamma 12	0007165 // signal transduction // inferred from electronic annotation///0007186 // G-pr
NM_010477///XM		0.02		-1.41	-1.53	1.27	1.02	***Hspd1***	heat shock protein 1 (chaperonin)	0001666 // response to hypoxia // inferred from electronic annotation///0002236 // det
NM_172685		0.005	0.024	-1.08	1.15	1.27	1.31	***Slc25a24***	solute carrier family 25 (mitochondrial carrier, phosphate carrier), member 24	0006810 // transport // inferred from electronic annotation///0006839 // mitochondrial
NM_144514///XM		#####		-1.07	1.06	1.27	1.16	***Commd1***	COMM domain containing 1	0006351 // transcription, DNA-templated // inferred from electronic annotation///00063
NM_1332	0.0105			1.01	-1.21	1.27	1.16	***Mtmr4***	myotubularin related protein 4	0006470 // protein dephosphorylation // inferred from electronic annotation///0007179
NM_001310746///		0.04		-1.05	1.10	1.27	1.01	***Ubl5***	ubiquitin-like 5	0000398 // mRNA splicing, via spliceosome // not recorded///0006464 // cellular protein
NM_0085	0.0136	0.008		1.02	1.19	1.27	1.16	***Mcm3***	minichromosome maintenance deficient 3 (S. cerevisiae)	0006260 // DNA replication // inferred from electronic annotation///0006270 // DNA rep
NM_025388		0.006		1.02	1.16	1.27	1.13	***Ufc1***	ubiquitin-fold modifier conjugating enzyme 1	0034976 // response to endoplasmic reticulum stress // not recorded///0071569 // prote
NM_1460	0.0293	0.02	0.022	-1.06	1.12	1.27	1.07	***Cbwd1***	COBW domain containing 1	
NM_008096		0.008		-1.39	1.01	1.28	-1.02	***Gc***	group specific component	0006810 // transport // inferred from electronic annotation///0007565 // female pregna
NM_1338	0.0396	0.045		-1.06	1.23	1.28	1.12	***Lrrc59***	leucine rich repeat containing 59	
NM_001159384///		0.032		1.04	1.06	1.28	1.15	***Mlx***	MAX-like protein X	0000122 // negative regulation of transcription from RNA polymerase II promoter // not
NM_0078	0.0388	#####	0.015	-1.22	1.10	1.28	1.08	***Cyp7b1***	cytochrome P450, family 7, subfamily b, polypeptide 1	0006629 // lipid metabolic process // inferred from electronic annotation///0006699 // b
NM_008981///XM		0.034		1.01	1.14	1.28	1.20	***Ptprg***	protein tyrosine phosphatase, receptor type, G	0006470 // protein dephosphorylation // inferred from electronic annotation///0010633
NM_0119	0.0043			1.06	1.35	1.28	1.32	***Xrn2***	5'-3' exoribonuclease 2	0000738 // DNA catabolic process, exonucleolytic // not recorded///0006139 // nucleoba
NM_0012	0.0229			1.04	-1.66	1.28	1.30	***Klf10***	Kruppel-like factor 10	0006351 // transcription, DNA-templated // inferred from electronic annotation///00063
NM_010790///XM		0.017		-1.07	1.16	1.28	1.07	***Melk***	maternal embryonic leucine zipper kinase	0006468 // protein phosphorylation // inferred from electronic annotation///0006915 //
NM_0291	0.0181	0.003	0.048	-1.02	1.13	1.28	1.28	***Sf3a3***	splicing factor 3a, subunit 3	0000398 // mRNA splicing, via spliceosome // not recorded///0006397 // mRNA processi
NM_026609		0.011		1.00	1.21	1.28	1.14	***Leprotl1***	leptin receptor overlapping transcript-like 1	
NM_025360		#####		-1.13	-1.01	1.29	1.13	***Tmed3***	transmembrane emp24 domain containing 3	0006810 // transport // inferred from electronic annotation///0015031 // protein transp
NM_0236	0.0112			1.09	1.39	1.29	-1.12	***Isyna1***	myo-inositol 1-phosphate synthase A1	0006021 // inositol biosynthetic process // inferred from electronic annotation///000662
NM_001205286///		0.045		-1.03	-1.08	1.29	1.09	***Tmem39a***	transmembrane protein 39a	0002230 // positive regulation of defense response to virus by host // not recorded///00
NM_0094	0.013	0.028		1.01	1.24	1.29	1.16	***Gm10145///Gm10705///Ube2l3***	predicted gene 10145///predicted gene 10705///ubiquitin-conjugating enzyme E2L 3	0000209 // protein polyubiquitination // not recorded///0006351 // transcription, DNA-t
NM_0011	0.029	0.039		-1.05	1.18	1.29	1.33	***Uhrf1***	ubiquitin-like, containing PHD and RING finger domains, 1	0000122 // negative regulation of transcription from RNA polymerase II promoter // infe
NM_0108	0.0392			1.17	1.44	1.29	1.45	***Ncl***	nucleolin	0001525 // angiogenesis // not recorded///0006897 // endocytosis // not recorded///00
NM_0280	0.0483	0.015		-1.06	1.08	1.29	1.35	***Cyb5r1***	cytochrome b5 reductase 1	0006629 // lipid metabolic process // inferred from electronic annotation///0006694 // s
NM_010295///XM		0.044	0.042	-1.05	1.17	1.29	1.18	***Gclc***	glutamate-cysteine ligase, catalytic subunit	0006534 // cysteine metabolic process // not recorded///0006536 // glutamate metaboli
NM_008720		0.04		-1.22	-1.03	1.29	1.36	***Npc1***	Niemann-Pick type C1	0006486 // protein glycosylation // inferred from direct assay///0006486 // protein glyco
NM_0010	0.0097			-1.06	-1.73	1.29	1.38	***Tsc22d3***	TSC22 domain family, member 3	0000122 // negative regulation of transcription from RNA polymerase II promoter // infe
NM_1724	0.0393			1.01	-1.29	1.30	1.19	***Slc38a5***	solute carrier family 38, member 5	0003333 // amino acid transmembrane transport // not recorded///0015804 // neutral a
NM_001304758///		0.021		1.04	1.18	1.30	1.18	***Coq7***	demethyl-Q 7	0000122 // negative regulation of transcription from RNA polymerase II promoter // not
NM_0093	0.0125	0.018		-1.12	1.72	1.30	1.02	***Tfpi2***	tissue factor pathway inhibitor 2	0007596 // blood coagulation // inferred from electronic annotation///0007599 // hemo
NM_1528	0.0143			1.17	1.66	1.30	1.20	***Plk2***	polo-like kinase 2	0000082 // G1/S transition of mitotic cell cycle // not recorded///0000278 // mitotic cell
NM_0261	0.046	0.006		-1.11	1.14	1.30	1.08	***Gtf3c6***	general transcription factor IIIC, polypeptide 6, alpha	0006351 // transcription, DNA-templated // not recorded///0006383 // transcription fro
NM_175096		0.016		-1.10	-1.59	1.31	-1.07	***Stbd1***	starch binding domain 1	0005975 // carbohydrate metabolic process // inferred from electronic annotation///000
NM_0010	0.0119	0.013		-1.04	1.16	1.31	1.48	***Ralgapa1***	Ral GTPase activating protein, alpha subunit 1	0006355 // regulation of transcription, DNA-templated // inferred from direct assay///00
NM_0215	0.0251			-1.01	1.22	1.31	1.35	***LOC102642514///Nsa2***	ribosome biogenesis protein NSA2 homolog///NSA2 ribosome biogenesis homolog (S.	0006364 // rRNA processing // inferred from electronic annotation///0042254 // riboson
NM_0010	0.0034	0.016		1.00	1.19	1.31	1.20	***Gnptab***	N-acetylglucosamine-1-phosphate transferase, alpha and beta subunits	0007040 // lysosome organization // not recorded///0009306 // protein secretion // infe
NM_019642///XM		0.029		-1.17	1.02	1.31	1.26	***Rpn2***	ribophorin II	0006486 // protein glycosylation // inferred from electronic annotation///0006487 // pro
NM_0117	0.0048	#####		-1.01	1.16	1.31	1.40	***Wdr1***	WD repeat domain 1	0007605 // sensory perception of sound // not recorded///0030036 // actin cytoskeleton
NM_010480		0.022		-1.39	-1.59	1.31	1.05	***Hsp90aa1***	heat shock protein 90, alpha (cytosolic), class A member 1	0001764 // neuron migration // not recorded///0006457 // protein folding // inferred fro
NM_0010	0.0046			-1.10	-1.55	1.31	1.28	***Tmem2***	transmembrane protein 2	0007275 // multicellular organismal development // inferred from electronic annotation
NM_001164190///		0.043		1.08	1.05	1.32	1.46	***Mtm1***	X-linked myotubular myopathy gene 1	0006470 // protein dephosphorylation // not recorded///0006629 // lipid metabolic proc

RefSeq Tr	*p* 24h	*p* 48h	*p* 72h	FD Air	FD 24h	FD 48h	FD 72h	Gene Symbol	Gene Title	Gene Ontology Biological Process
NM_0242	0.0069	0.002		-1.18	1.17	1.32	1.19	***Sec13***	SEC13 homolog, nuclear pore and COPII coat complex component	0006810 // transport // inferred from electronic annotation///0006888 // ER to Golgi ves
NM_0174	0.0062		0.028	-1.09	1.27	1.32	1.18	***Copg2***	coatomer protein complex, subunit gamma 2	0006810 // transport // inferred from electronic annotation///0006886 // intracellular pr
NM_011057///XM_00652			0.005	1.42	1.40	1.32	1.19	***Pdgfb***	platelet derived growth factor, B polypeptide	0001568 // blood vessel development // inferred from expression pattern///0001666 // r
NM_028303///XM		0.022		-1.04	1.08	1.32	1.09	***Pdzd11***	PDZ domain containing 11	0006887 // exocytosis // not recorded///0007269 // neurotransmitter secretion // not re
NM_0010	0.0432	0.015		-1.02	1.35	1.32	1.32	***Kdm7a***	lysine (K)-specific demethylase 7A	0006351 // transcription, DNA-templated // inferred from electronic annotation///00063
NM_023579///XM		0.031		1.04	1.11	1.32	1.33	***Ipo5***	importin 5	0000059 // protein import into nucleus, docking // not recorded///0000060 // protein im
NM_0104	0.0396			1.27	3.72	1.32	1.07	***Nr4a1***	nuclear receptor subfamily 4, group A, member 1	0001938 // positive regulation of endothelial cell proliferation // not recorded///0002042
NM_0011	0.0229			-1.10	1.48	1.32	-1.20	***Hist1h2ao///Hist1h2ap***	histone cluster 1, H2ao///histone cluster 1, H2ap	0006325 // chromatin organization // not recorded///0006342 // chromatin silencing // r
NM_0089	0.0466			1.03	1.21	1.33	1.21	***Psma2***	proteasome (prosome, macropain) subunit, alpha type 2	0006508 // proteolysis // inferred from electronic annotation///0006511 // ubiquitin-dep
NM_0237	0.0237			1.16	1.64	1.33	1.65	***Etv5***	ets variant 5	0006355 // regulation of transcription, DNA-templated // inferred from electronic annota
NM_145953///XM		0.021		-1.16	-1.24	1.33	-1.05	***Cth***	cystathionase (cystathionine gamma-lyase)	0006749 // glutathione metabolic process // not recorded///0008285 // negative regulat
NM_0012	0.0071	0.04		-1.13	1.32	1.33	1.18	***Psat1***	phosphoserine aminotransferase 1	0006564 // L-serine biosynthetic process // inferred from electronic annotation///00081
NM_1451	0.0188	0.03		-1.02	1.16	1.33	1.18	***Mgl2***	macrophage galactose N-acetyl-galactosamine specific lectin 2	
NM_0135	0.0224	0.018		-1.89	-4.51	1.33	-1.17	***Hsph1***	heat shock 105kDa/110kDa protein 1	0006986 // response to unfolded protein // traceable author statement///0043524 // ne
NM_001164787///		0.015		-1.01	1.01	1.34	1.24	***Sprr2a1///Sprr2a2///Sprr2a3***	small proline-rich protein 2A1///small proline-rich protein 2A2///small proline-rich pro	0008360 // regulation of cell shape // traceable author statement///0008544 // epidermi
NM_0011	0.0134	0.044	0.048	-1.09	1.33	1.34	1.50	***Kit***	kit oncogene	0000187 // activation of MAPK activity // not recorded///0001541 // ovarian follicle deve
NM_010860///XM		0.021		-1.07	1.02	1.34	1.31	***Myl6///Gm10080***	myosin, light polypeptide 6, alkali, smooth muscle and non-muscle///predicted gene 1	0006936 // muscle contraction // traceable author statement///0007519 // skeletal musc
NM_025310		0.028	0.011	1.01	1.17	1.34	1.20	***Ftsj3***	FtsJ homolog 3 (E. coli)	0000453 // enzyme-directed rRNA 2'-O-methylation // inferred from electronic annotatio
NM_001301211///		0.045		1.03	1.11	1.34	1.14	***Ap1g1***	adaptor protein complex AP-1, gamma 1 subunit	0000226 // microtubule cytoskeleton organization // not recorded///0006810 // transpo
NM_001025192///		0.039		1.07	1.17	1.35	1.27	***Cxadr***	coxsackie virus and adenovirus receptor	0007005 // mitochondrion organization // inferred from mutant phenotype///0007155 //
NR_030708///XM_		0.009		1.08	1.16	1.35	1.31	***6820431F20Rik///Gm21811***	cadherin 11 pseudogene///predicted gene, 21811	0007156 // homophilic cell adhesion via plasma membrane adhesion molecules // infere
NM_025968///XM		0.029		1.03	1.03	1.35	1.36	***Ptgr1***	prostaglandin reductase 1	0006693 // prostaglandin metabolic process // inferred from electronic annotation///000
NM_0092	0.0043			-1.01	2.18	1.35	-1.13	***Sprr1a***	small proline-rich protein 1A	0018149 // peptide cross-linking // not recorded///0030216 // keratinocyte differentiatio
NM_020259///XM		0.008	0.036	1.05	1.07	1.35	1.42	***Hhip***	Hedgehog-interacting protein	0005975 // carbohydrate metabolic process // inferred from electronic annotation///000
NM_0012	0.0262	0.021		1.05	1.27	1.36	1.20	***Ddx46***	DEAD (Asp-Glu-Ala-Asp) box polypeptide 46	0000398 // mRNA splicing, via spliceosome // not recorded///0006397 // mRNA processi
NM_0079	0.0087	0.033		-1.07	1.20	1.36	1.19	***Erh***	enhancer of rudimentary homolog (Drosophila)	0001649 // osteoblast differentiation // inferred from direct assay///0006221 // pyrimidi
NM_001145799///		0.035		-1.19	-1.15	1.36	1.06	***Ctla2a///Ctla2b***	cytotoxic T lymphocyte-associated protein 2 alpha///cytotoxic T lymphocyte-associate	0010951 // negative regulation of endopeptidase activity // traceable author statement/
NM_0119	######		0.033	-1.05	1.20	1.36	1.21	***Psma4***	proteasome (prosome, macropain) subunit, alpha type 4	0006508 // proteolysis // inferred from electronic annotation///0006511 // ubiquitin-dep
NM_0085	0.0374	0.025		1.03	1.26	1.36	1.22	***Met***	met proto-oncogene	0000122 // negative regulation of transcription from RNA polymerase II promoter // not r
NM_0089	0.0364			-1.11	1.11	1.36	1.42	***Psmc1***	protease (prosome, macropain) 26S subunit, ATPase 1	0030163 // protein catabolic process // inferred from electronic annotation///0030433 //
NM_030889///XM		0.016		1.02	1.10	1.37	1.25	***Sorcs2***	sortilin-related VPS10 domain containing receptor 2	0006810 // transport // inferred from electronic annotation
NM_001033375///		0.041		1.03	1.07	1.37	1.20	***A230046K03Rik***	RIKEN cDNA A230046K03 gene	0006810 // transport // inferred from electronic annotation///0007032 // endosome org
NM_001313711///		0.018		1.09	1.22	1.37	1.11	***Prdx4***	peroxiredoxin 4	0007283 // spermatogenesis // inferred from mutant phenotype///0008584 // male gona
NM_010830		0.034	#####	-1.17	-1.05	1.37	1.13	***Msh6***	mutS homolog 6 (E. coli)	0000710 // meiotic mismatch repair // not recorded///0006281 // DNA repair // not reco
NM_0011	######			-1.04	1.03	1.37	1.29	***Kif20a***	kinesin family member 20A	0000281 // mitotic cytokinesis // not recorded///0000910 // cytokinesis // not recorded/
NM_0255	0.0445	0.036		-1.14	1.02	1.37	1.26	***Ostc***	oligosaccharyltransferase complex subunit	
NM_0011	0.0346			1.05	1.00	1.37	1.57	***Cldn18***	claudin 18	0016338 // calcium-independent cell-cell adhesion via plasma membrane cell-adhesion m
NM_0011	0.0177	0.034		-1.06	1.10	1.37	1.20	***Glo1***	glyoxalase 1	0005975 // carbohydrate metabolic process // not recorded///0006357 // regulation of t
NM_033075///XR_		0.05		-1.12	-1.11	1.38	1.50	***D17H6S56E-5***	DNA segment, Chr 17, human D6S56E 5	0019058 // viral life cycle // inferred from electronic annotation
NM_172118///XM		0.019		-1.14	-1.03	1.38	1.39	***Myl9***	myosin, light polypeptide 9, regulatory	0070527 // platelet aggregation // not recorded
NM_1832	0.0327		0.043	-1.12	1.24	1.38	1.26	***Wfdc21***	WAP four-disulfide core domain 21	0010466 // negative regulation of peptidase activity // inferred from electronic annotatio
NM_009443///NM		0.011		1.04	1.21	1.38	1.23	***Tgoln1///Tgoln2***	trans-golgi network protein///trans-golgi network protein 2	0006895 // Golgi to endosome transport // not recorded
NM_0011	0.0021			1.10	1.40	1.38	1.36	***Dynlt1-ps1///Dynlt1a///Dynlt1b///Dynlt1c***	dynein light chain Tctex-type 1, pseudogene 1///dynein light chain Tctex-type 1A///d	0000132 // establishment of mitotic spindle orientation // inferred from mutant phenoty
NM_013463		0.015		1.10	1.13	1.38	1.22	***Gla***	galactosidase, alpha	0005975 // carbohydrate metabolic process // inferred from electronic annotation///000
NM_007392///XM		0.049		-1.25	-1.13	1.38	1.12	***Acta2***	actin, alpha 2, smooth muscle, aorta	0006936 // muscle contraction // not recorded///0008217 // regulation of blood pressur
NM_0111	0.0274	0.005		-1.03	1.25	1.39	1.00	***Papola***	poly (A) polymerase alpha	0000398 // mRNA splicing, via spliceosome // ---///0006369 // termination of RNA polym
NM_007856///XM		0.033		1.08	1.24	1.39	-1.04	***Dhcr7***	7-dehydrocholesterol reductase	0001568 // blood vessel development // inferred from mutant phenotype///0006629 // l
NM_001109971///		0.022		-1.08	1.11	1.39	-1.04	***Sec61g***	protein transport protein Sec61 subunit gamma///protein transport protein Sec61 sub	0006605 // protein targeting // inferred from electronic annotation///0006810 // transpo
NM_0011	0.0272	0.039		1.09	1.44	1.40	1.47	***Magi3***	membrane associated guanylate kinase, WW and PDZ domain containing 3	0007165 // signal transduction // inferred from electronic annotation///0016310 // phos
NM_0101	0.023	0.042		1.07	1.57	1.40	1.26	***S1pr3***	sphingosine-1-phosphate receptor 3	0001816 // cytokine production // inferred from mutant phenotype///0003376 // sphing
NM_001286062///		0.029		1.08	1.29	1.41	1.09	***Angpt1***	angiopoietin 1	0001525 // angiogenesis // inferred from direct assay///0001525 // angiogenesis // infer
NM_173006///XM		0.023		-1.02	1.13	1.41	1.31	***Pon3***	paraoxonase 3	0006470 // protein dephosphorylation // inferred from electronic annotation///0009636
NM_008924		0.002		-1.04	1.21	1.41	1.37	***Prkar2a***	protein kinase, cAMP dependent regulatory, type II alpha	0001932 // regulation of protein phosphorylation // inferred from electronic annotation/
NM_019699		0.014		1.04	1.18	1.41	1.06	***Fads2***	fatty acid desaturase 2	0006629 // lipid metabolic process // inferred from electronic annotation///0006631 // f
NM_1725	0.0055			1.29	1.14	1.41	1.41	***A730008H23Rik///Hjurp***	RIKEN cDNA A730008H23 gene///Holliday junction recognition protein///RIKEN cDNA	0007049 // cell cycle // inferred from electronic annotation///0007059 // chromosome s
NM_0011	0.0081			1.06	1.59	1.41	1.11	***Gm10693///Gm14548///Lilra6///Pira1///P***	predicted pseudogene 10693///predicted gene 14548///leukocyte immunoglobulin-lik	0001782 // B cell homeostasis // inferred from mutant phenotype///0002250 // adaptive
NM_009610		0.009	0.037	-1.50	-1.14	1.42	1.19	***Actg2***	actin, gamma 2, smooth muscle, enteric	0010628 // positive regulation of gene expression // inferred from sequence or structural
NM_001277101///		0.033		1.07	1.18	1.42	1.25	***Cops5***	COP9 (constitutive photomorphogenic) homolog, subunit 5 (Arabidopsis thaliana)	0000338 // protein deneddylation // not recorded///0006355 // regulation of transcripti
NM_011631		0.04		-1.29	-1.22	1.42	1.34	***Hsp90b1***	heat shock protein 90, beta (Grp94), member 1	0001666 // response to hypoxia // not recorded///0006457 // protein folding // inferred
	0.0042			1.67	4.36	1.42	1.10	***Ighd***	immunoglobulin heavy constant delta	0016064 // immunoglobulin mediated immune response // inferred from mutant phenot
NM_0084	0.0041	0.011		-1.14	1.67	1.43	1.37	***Lcn2***	lipocalin 2	0002376 // immune system process // inferred from electronic annotation///0006810 //
NM_0119	######	0.007		1.02	1.64	1.45	1.38	***LOC102643033///Zfp146***	zinc finger protein OZF///zinc finger protein 146	0006355 // regulation of transcription, DNA-templated // not recorded
NM_0013	0.0032	0.008		-1.10	1.27	1.45	1.28	***Mcm5***	minichromosome maintenance deficient 5, cell division cycle 46 (S. cerevisiae)	0006260 // DNA replication // inferred from electronic annotation///0006270 // DNA rep
NM_019802///XM		0.008	0.035	-1.06	1.09	1.45	1.39	***Ggcx***	gamma-glutamyl carboxylase	0017187 // peptidyl-glutamic acid carboxylation // not recorded

RefSeq Tr	*p* 24h	*p* 48h	*p* 72h	FD Air	FD 24h	FD 48h	FD 72h	Gene Symbol	Gene Title	Gene Ontology Biological Process
NM_0297	0.003	0.005		-1.07	2.00	1.45	-1.04	***Lrg1***	leucine-rich alpha-2-glycoprotein 1	0001938 // positive regulation of endothelial cell proliferation // inferred from mutant ph
NM_0010	######			1.00	1.37	1.45	1.24	***Ddhd1***	DDHD domain containing 1	0006629 // lipid metabolic process // inferred from electronic annotation///0016042 // li
NM_1725	0.0073			1.30	1.14	1.46	1.45	***6430706D22Rik///A730008H23Rik///Hjurp***	RIKEN cDNA 6430706D22 gene///RIKEN cDNA A730008H23 gene///Holliday junction r	0007049 // cell cycle // inferred from electronic annotation///0007059 // chromosome s
NM_0012	0.0025	0.015		1.01	1.87	1.46	1.24	***Cd68***	CD68 antigen	0071310 // cellular response to organic substance // inferred from direct assay
NM_001025431///		0.023		1.05	1.24	1.46	1.32	***Btbd3***	BTB (POZ) domain containing 3	0007399 // nervous system development // inferred from electronic annotation///00219
NM_0254	0.0138	0.002		-1.12	1.11	1.47	1.18	***Ssr2***	signal sequence receptor, beta	
NM_010111///XM		0.041		1.09	1.03	1.47	1.13	***Efnb2***	ephrin B2	0001525 // angiogenesis // inferred from electronic annotation///0001945 // lymph vess
NM_008969///XM		0.021		1.04	1.40	1.47	1.19	***Ptgs1***	prostaglandin-endoperoxide synthase 1	0001516 // prostaglandin biosynthetic process // inferred from mutant phenotype///000
NM_0011	0.0011	0.048		1.09	1.55	1.48	1.03	***Acot7***	acyl-CoA thioesterase 7	0009062 // fatty acid catabolic process // inferred from direct assay///0015937 // coenzy
NM_0531	######	0.037		-1.12	1.32	1.49	1.34	***Glrx***	glutaredoxin	0006810 // transport // inferred from electronic annotation///0032024 // positive regula
NM_0013	0.0307	0.016		1.05	1.24	1.51	1.17	***Mcm6***	minichromosome maintenance deficient 6 (MIS5 homolog, S. pombe) (S. cerevisiae)	0006260 // DNA replication // inferred from electronic annotation///0006268 // DNA unv
NM_026931		0.015		-1.10	-1.32	1.52	1.15	***1810011O10Rik***	RIKEN cDNA 1810011O10 gene	0002264 // endothelial cell activation involved in immune response // not recorded///00
NM_0134	0.0142	0.043		-1.21	1.49	1.52	1.06	***Ankrd1***	ankyrin repeat domain 1 (cardiac muscle)	0000122 // negative regulation of transcription from RNA polymerase II promoter // not r
NM_0012	0.009	0.03		-1.07	1.65	1.52	1.02	***Lilr4b///Lilrb4a***	leukocyte immunoglobulin-like receptor, subfamily B, member 4B///leukocyte immuno	0002250 // adaptive immune response // inferred from electronic annotation///0002376
NM_008302		0.026		-1.30	-1.30	1.53	1.51	***Hsp90ab1***	heat shock protein 90 alpha (cytosolic), class B member 1	0001890 // placenta development // inferred from mutant phenotype///0006457 // prot
NM_0011	######	0.01		-1.26	1.33	1.53	1.09	***Clec4n***	C-type lectin domain family 4, member n	0002250 // adaptive immune response // inferred from electronic annotation///0002376
NM_0011	0.0074			-1.10	1.45	1.53	1.02	***Itih4***	inter alpha-trypsin inhibitor, heavy chain 4	0006953 // acute-phase response // inferred from electronic annotation///0010466 // ne
NM_0111	0.0435			1.65	1.25	1.53	1.93	***Plod1***	procollagen-lysine, 2-oxoglutarate 5-dioxygenase 1	0001666 // response to hypoxia // inferred from electronic annotation///0008544 // epic
NM_009226		0.029		-1.07	1.15	1.53	1.17	***Snrpd1***	small nuclear ribonucleoprotein D1	0000245 // spliceosomal complex assembly // not recorded///0000387 // spliceosomal s
NM_0093	0.0018	0.009		-1.01	1.39	1.54	1.17	***Tgfbi***	transforming growth factor, beta induced	0001525 // angiogenesis // inferred from electronic annotation///0002062 // chondrocyt
NM_0098	0.0058			-1.07	1.88	1.54	1.27	***Chil3///Chil4***	chitinase-like 3///chitinase-like 4	0000272 // polysaccharide catabolic process // inferred from electronic annotation///00
NM_0011	0.0152	0.024		-1.05	1.46	1.55	1.36	***Il33***	interleukin 33	0002686 // negative regulation of leukocyte migration // inferred from genetic interactio
NM_001313977///		0.036		1.03	1.31	1.56	1.16	***Prkch***	protein kinase C, eta	0006468 // protein phosphorylation // not recorded///0010744 // positive regulation of
NM_0116	0.0087	0.006		-1.65	1.30	1.57	1.11	***Tnc***	tenascin C	0001649 // osteoblast differentiation // not recorded///0007155 // cell adhesion // infer
NM_026835		0.048		-1.09	1.14	1.58	1.15	***Ms4a6d***	membrane-spanning 4-domains, subfamily A, member 6D	
NM_001159564///		0.039		1.14	1.35	1.58	1.44	***Itgb6***	integrin beta 6	0006954 // inflammatory response // inferred from mutant phenotype///0007155 // cell
NM_0074	0.0087	0.015		-1.25	1.36	1.58	1.11	***Arg1***	arginase, liver	0000050 // urea cycle // not recorded///0001889 // liver development // inferred from e
NM_009363		0.024		-1.08	-1.01	1.59	1.12	***Tff2***	trefoil factor 2 (spasmolytic protein 1)	0008284 // positive regulation of cell proliferation // inferred from genetic interaction///
NM_0195	0.0397			1.29	1.84	1.60	1.11	***Cxcl14***	chemokine (C-X-C motif) ligand 14	0006955 // immune response // inferred from electronic annotation///0045662 // negati
NM_0011	0.0054			-1.03	1.47	1.61	1.48	***Eif4a-ps4///Eif4a1***	eukaryotic translation initiation factor 4A, pseudogene 4///eukaryotic translation initia	0006412 // translation // inferred from electronic annotation///0006413 // translational
NM_011526		0.003		-1.16	1.02	1.61	1.41	***Tagln***	transgelin	0007010 // cytoskeleton organization // inferred from mutant phenotype///0030855 // e
NM_001168525///		0.042		1.01	-1.01	1.67	1.48	***Sgms1***	sphingomyelin synthase 1	0006629 // lipid metabolic process // inferred from electronic annotation///0006665 // s
NM_0011	0.0131			1.60	1.39	1.68	1.61	***Wdfy1***	WD repeat and FYVE domain containing 1	0034141 // positive regulation of toll-like receptor 3 signaling pathway // inferred from m
NM_008230///XM		0.005		-1.05	1.31	1.68	1.22	***Hdc***	histidine decarboxylase	0001692 // histamine metabolic process // not recorded///0001694 // histamine biosynt
NM_0010	0.013	#####		-1.04	1.79	1.70	1.04	***Timp1***	tissue inhibitor of metalloproteinase 1	0001775 // cell activation // not recorded///0007568 // aging // inferred from electronic
NM_0012	0.0219			1.32	1.82	1.71	1.44	***Pira1///Pira2///Pira6///Gm10693///Gm14***	paired-Ig-like receptor A1///paired-Ig-like receptor A2///paired-Ig-like receptor A6///p	0001782 // B cell homeostasis // inferred from mutant phenotype///0002250 // adaptive
NM_0010	0.0145	0.005		1.07	1.47	1.71	1.28	***Lrp2***	low density lipoprotein receptor-related protein 2	0003281 // ventricular septum development // inferred from mutant phenotype///00067
NM_0012	0.0061	0.011		1.04	1.59	1.76	1.46	***Fdps***	farnesyl diphosphate synthetase	0006629 // lipid metabolic process // inferred from electronic annotation///0006694 // s
NM_001166173///		0.024	0.031	1.00	1.20	1.77	1.64	***Dmkn***	dermokine	0030154 // cell differentiation // inferred from electronic annotation///1903575 // cornif
NM_001289462///		0.024		1.10	1.37	1.81	1.64	***Mme***	membrane metallo endopeptidase	0001822 // kidney development // inferred from sequence or structural similarity///0001
NM_1451	######	0.026		-1.16	2.24	1.81	1.48	***Chil4///Chil3***	chitinase-like 4///chitinase-like 3	0005975 // carbohydrate metabolic process // inferred from electronic annotation///000
NM_001166672///		0.041		1.18	2.04	1.83	1.34	***Gm10693///Gm14548///Lilra6///Pira1///P***	predicted pseudogene 10693///predicted gene 14548///leukocyte immunoglobulin-lik	0001782 // B cell homeostasis // inferred from mutant phenotype///0002250 // adaptive
NM_0118	0.0038	0.002	0.047	-1.06	2.42	1.84	1.35	***Slc26a4***	solute carrier family 26, member 4	0006810 // transport // inferred from electronic annotation///0006821 // chloride transp
NM_1776	######			1.92	1.41	1.96	2.12	***Cep104***	centrosomal protein 104	
NM_0012	0.0059			1.41	2.23	2.03	1.62	***Pira1///Pira2///Pira6///Gm10693///Gm14***	paired-Ig-like receptor A1///paired-Ig-like receptor A2///paired-Ig-like receptor A6///p	0001782 // B cell homeostasis // inferred from mutant phenotype///0002250 // adaptive
NM_028757///XM		0.015		1.09	1.45	2.23	1.77	***Nebl***	nebulette	0071691 // cardiac muscle thin filament assembly // inferred from sequence or structura
NM_017474///XM		0.015		-1.13	1.13	3.08	1.26	***Clca1///Clca3a1***	chloride channel accessory 1///chloride channel accessory 3A1	0006508 // proteolysis // inferred from electronic annotation///0006810 // transport // i
XM_011238903///		0.033		2.54	2.61	3.75	3.71	***Gm37416///Gm39743///Gm40611///Gm4***	predicted gene, 37416///predicted gene, 39743///predicted gene, 40611///predicted	0006278 // RNA-dependent DNA replication // inferred from electronic annotation///000

Table S5. NFKB1-dependent basal lung genes.

Slected genes varied >1.5-fold between Nfkb1-WT and Nfkb1-KO mice (positive-higher in KO, negative-lower in KO).

Moderated t-test (p<0.05). Order by fold difference (FD) between WT and KO mice exposed to air.

RefSeq Transcript ID	p	FD Air	*Gene Symbol*	Gene Title	Gene Ontology
NM_152839	0.006	-20.028	***Jchain***	immunoglobulin joining chain	0001895 // retina homeostasis // inferred from electronic annotation///0002250 // adaptive i
	0.034	-8.807	***Igkv15-103***	immunoglobulin kappa chain variable 15-103	0006898 // receptor-mediated endocytosis // ---///0006910 // phagocytosis, recognition // ---
NM_023835///XM_01:	0.044	-6.990	***Trim12a***	tripartite motif-containing 12A	0008270 // zinc ion binding // inferred from electronic annotation///0046872 // metal ion bir
NM_139142	0.009	-5.330	***Slc6a20a***	solute carrier family 6 (neurotransmitter transporter), member 2(	0003333 // amino acid transmembrane transport // not recorded///0006810 // transport // ir
NM_018866	0.015	-4.263	***Cxcl13***	chemokine (C-X-C motif) ligand 13	0002518 // lymphocyte chemotaxis across high endothelial venule // inferred from mutant pł
NM_001281969///NM_	0.000	-3.620	***Glrb***	glycine receptor, beta subunit	0001964 // startle response // not recorded///0001964 // startle response // inferred from se
NM_001131054///NM_	0.000	-3.506	***Pttg1***	pituitary tumor-transforming gene 1	0001558 // regulation of cell growth // not recorded///0006281 // DNA repair // inferred fror
NM_053128	0.011	-3.332	***Pcdhb3***	protocadherin beta 3	0007155 // cell adhesion // inferred from electronic annotation///0007156 // homophilic cell
NM_001146049///NM_	0.048	-3.218	***Htatip2***	HIV-1 tat interactive protein 2, homolog (human)	0001525 // angiogenesis // inferred from electronic annotation///0006915 // apoptotic proce
NM_009234	0.010	-3.214	***Sox11***	SRY (sex determining region Y)-box 11	0000122 // negative regulation of transcription from RNA polymerase II promoter // inferred
NM_001310602///XM_	0.041	-3.163	***Trim5***	tripartite motif-containing 5	0002221 // pattern recognition receptor signaling pathway // ---///0016567 // protein ubiquit
NM_001164612///NM_	0.001	-3.009	***Atp13a4***	ATPase type 13A4	0006812 // cation transport // inferred from electronic annotation///0006874 // cellular calci
NM_153405///NM_17:	0.000	-2.895	***Rbm45***	RNA binding motif protein 45	0007275 // multicellular organismal development // inferred from electronic annotation///00
NM_021050///XM_00(	0.009	-2.823	***Cftr***	cystic fibrosis transmembrane conductance regulator	0003254 // regulation of membrane depolarization // inferred from mutant phenotype///000
NM_009052	0.001	-2.817	***Bex1***	brain expressed gene 1	0002052 // positive regulation of neuroblast proliferation // not recorded///0007275 // multi
NM_011704///XM_00(	0.003	-2.732	***Vnn1***	vanin 1	0002526 // acute inflammatory response // inferred from direct assay///0002544 // chronic ir
NM_001081971	0.042	-2.693	***Ankrd63***	ankyrin repeat domain 63	
NM_010766	0.001	-2.584	***Marco***	macrophage receptor with collagenous structure	0002376 // immune system process // inferred from electronic annotation///0006897 // endo
NM_008079///XM_00(	0.036	-2.581	***Galc***	galactosylceramidase	0005975 // carbohydrate metabolic process // inferred from electronic annotation///000662(
NM_001202500///XM_	0.012	-2.537	***Armcx4***	armadillo repeat containing, X-linked 4	
NM_026516	0.007	-2.444	***Tmem178***	transmembrane protein 178	0016020 // membrane // inferred from electronic annotation///0016021 // integral compone
NM_054084///XM_00(	0.001	-2.372	***Calcb***	calcitonin-related polypeptide, beta	0005576 // extracellular region // inferred from electronic annotation
NM_029569///XM_00(	0.045	-2.360	***Asb5***	ankyrin repeat and SOCs box-containing 5	0016567 // protein ubiquitination // inferred from electronic annotation///0035556 // intrace
NM_009364///XM_00(	0.024	-2.345	***Tfpi2***	tissue factor pathway inhibitor 2	0007596 // blood coagulation // inferred from electronic annotation///0007599 // hemostasi:
NM_008730	0.006	-2.300	***Nptx1***	neuronal pentraxin 1	0006839 // mitochondrial transport // not recorded///0035865 // cellular response to potassi
NM_153389///NR_003	0.001	-2.291	***Atp10d***	ATPase, class V, type 10D	0006810 // transport // inferred from electronic annotation///0006812 // cation transport //
NM_022315///XM_00(	0.010	-2.287	***Smoc2***	SPARC related modular calcium binding 2	0007165 // signal transduction // inferred from electronic annotation///0010811 // positive r
NM_053169///XM_00(	0.017	-2.262	***Trim16***	tripartite motif-containing 16	0043966 // histone H3 acetylation // not recorded///0043967 // histone H4 acetylation // not
NM_001111274///NM_	0.008	-2.242	***Igf1***	insulin-like growth factor 1	0001649 // osteoblast differentiation // inferred from expression pattern///0001775 // cell ac
NM_010140	0.039	-2.202	***Epha3***	Eph receptor A3	0003197 // endocardial cushion development // inferred from mutant phenotype///0006468
NM_178931///XM_00(	0.006	-2.191	***Tnfrsf14***	tumor necrosis factor receptor superfamily, member 14 (herpesv	0002741 // positive regulation of cytokine secretion involved in immune response // inferred
NM_023186	0.004	-2.189	***Chia1***	chitinase, acidic 1	0000272 // polysaccharide catabolic process // inferred from electronic annotation///000237
NM_001310659///NM_	0.028	-2.144	***Igfbp2***	insulin-like growth factor binding protein 2	0001558 // regulation of cell growth // inferred from electronic annotation///0007165 // sign
NM_001164210///NM_	0.002	-2.142	***Sptssb***	serine palmitoyltransferase, small subunit B	0006629 // lipid metabolic process // inferred from electronic annotation///0006665 // sphin
NM_001289755///NM_	0.004	-2.131	***Apoc3***	apolipoprotein C-III	0006629 // lipid metabolic process // inferred from electronic annotation///0006641 // triglyc
NM_175473///XM_00(	0.014	-2.131	***Fras1***	Fraser syndrome 1 homolog (human)	0002009 // morphogenesis of an epithelium // inferred from mutant phenotype///0003338 /,
NM_001293800///NM_	0.048	-2.104	***Ocel1***	occludin/ELL domain containing 1	
NR_035452	0.002	-2.096	***Mir1931***	microRNA 1931	
NM_021456	0.007	-2.084	***Ces1g***	carboxylesterase 1G	0008152 // metabolic process // inferred from electronic annotation///0010468 // regulation
NM_017399	0.016	-2.080	***Fabp1***	fatty acid binding protein 1, liver	0002230 // positive regulation of defense response to virus by host // not recorded///000681
NM_025748///XM_01:	0.045	-2.075	***Adat2***	adenosine deaminase, tRNA-specific 2	0002100 // tRNA wobble adenosine to inosine editing // inferred from electronic annotation/,
NM_001302531///NM_	0.031	-2.029	***Esr1***	estrogen receptor 1 (alpha)	0001547 // antral ovarian follicle growth // inferred from mutant phenotype///0002064 // ep
NR_015524	0.010	-2.017	***Cep83os***	centrosomal protein 83, opposite strand	
NM_008689///XM_00(	0.000	-2.012	***Nfkb1***	nuclear factor of kappa light polypeptide gene enhancer in B cells	0000122 // negative regulation of transcription from RNA polymerase II promoter // not recor
NM_013546///XM_01:	0.017	-1.970	***Hebp1***	heme binding protein 1	0042168 // heme metabolic process // inferred by curator
NM_001081342///NM_	0.042	-1.960	***Adgrd1***	adhesion G protein-coupled receptor D1	0007166 // cell surface receptor signaling pathway // inferred from electronic annotation///0
NR_015487///NR_027ɛ	0.000	-1.956	***A930005H10Rik***	RIKEN cDNA A930005H10 gene	
NM_001252627///NM_	0.007	-1.955	***Cdh16***	cadherin 16	0007155 // cell adhesion // inferred from electronic annotation///0007156 // homophilic cell
NM_001081328	0.021	-1.953	***Chsy3***	chondroitin sulfate synthase 3	0008152 // metabolic process // inferred from electronic annotation
NM_001159401///NM_	0.009	-1.953	***Upp1***	uridine phosphorylase 1	0006218 // uridine catabolic process // inferred from direct assay///0006220 // pyrimidine nu
NM_177303///XM_01:	0.010	-1.952	***Lrrn4***	leucine rich repeat neuronal 4	0007616 // long-term memory // inferred from mutant phenotype///0008542 // visual learni

RefSeq Transcript ID	p	FD Air	*Gene Symbol*	Gene Title	Gene Ontology
NM_011606	0.005	-1.923	***Clec3b***	C-type lectin domain family 3, member b	0001501 // skeletal system development // inferred from mutant phenotype///0001503 // os
NM_172757	0.027	-1.897	***Heatr3***	HEAT repeat containing 3	
NM_001164733///NM_	0.010	-1.890	***Mpp6***	membrane protein, palmitoylated 6 (MAGUK p55 subfamily mem	0046037 // GMP metabolic process // inferred from sequence or structural similarity///00467
NM_181420///XM_006	0.029	-1.875	***Fn3krp***	fructosamine 3 kinase related protein	0016310 // phosphorylation // not recorded
NM_001113560///NM_	0.001	-1.869	***Glo1***	glyoxalase 1	0005975 // carbohydrate metabolic process // not recorded///0006357 // regulation of trans
NM_172471///XM_006	0.005	-1.845	***Itih5***	inter-alpha (globulin) inhibitor H5	0010466 // negative regulation of peptidase activity // inferred from electronic annotation///
NM_175309	0.013	-1.830	***Upk3b***	uroplakin 3B	0010629 // negative regulation of gene expression // not recorded///0046325 // negative reg
NM_172399///XM_006	0.032	-1.830	***Ndnf***	neuron-derived neurotrophic factor	0001525 // angiogenesis // not recorded///0001764 // neuron migration // not recorded///0
XR_879970///XR_8799	0.000	-1.806	***Tnfsf13os***	tumor necrosis factor (ligand) superfamily, member 13, opposite strand	
NM_153127	0.002	-1.803	***Mmrn2***	multimerin 2	0001525 // angiogenesis // inferred from electronic annotation///0030948 // negative regula
NM_001109988///NM_	0.002	-1.791	***Nrep***	neuronal regeneration related protein	0017015 // regulation of transforming growth factor beta receptor signaling pathway // infer
NM_001166377///NM_	0.023	-1.771	***Armcx1***	armadillo repeat containing, X-linked 1	0016020 // membrane // inferred from electronic annotation///0016021 // integral compone
NM_001110850///NM_	0.045	-1.770	***Crem***	cAMP responsive element modulator	0006006 // glucose metabolic process // inferred from electronic annotation///0006351 // tra
NM_010291	0.014	-1.759	***Gjb5***	gap junction protein, beta 5	0007154 // cell communication // inferred from electronic annotation///0060707 // trophobl
NM_009022	0.046	-1.736	***Aldh1a2***	aldehyde dehydrogenase family 1, subfamily A2	0001523 // retinoid metabolic process // traceable author statement///0001568 // blood ves
NM_010809	0.000	-1.730	***Mmp3***	matrix metallopeptidase 3	0006508 // proteolysis // inferred from mutant phenotype///0006508 // proteolysis // not re
NM_008409	0.012	-1.722	***Itm2a***	integral membrane protein 2A	0005515 // protein binding // inferred from electronic annotation
NM_001198565///NM_	0.014	-1.712	***Sulf1***	sulfatase 1	0001502 // cartilage condensation // inferred from sequence or structural similarity///00018
NM_001083917///NM_	0.000	-1.710	***Scn3b***	sodium channel, voltage-gated, type III, beta	0006810 // transport // inferred from electronic annotation///0006811 // ion transport // inf
NM_007562///XM_011	0.018	-1.708	***Bnc1***	basonuclin 1	0006351 // transcription, DNA-templated // inferred from electronic annotation///0006355 /
NM_007984	0.001	-1.705	***Fscn1***	fascin homolog 1, actin bundling protein (Strongylocentrotus pur	0007015 // actin filament organization // inferred from electronic annotation///0007043 // ce
NM_001111111///XM_	0.027	-1.700	***Atg16l2***	autophagy related 16-like 2 (S. cerevisiae)	0000045 // autophagosome assembly // not recorded///0006810 // transport // inferred fron
NM_138683///XM_006	0.029	-1.692	***Rspo1***	R-spondin 1	0001934 // positive regulation of protein phosphorylation // not recorded///0002090 // regul
NM_001025364///NM_	0.010	-1.686	***Rtn2***	reticulon 2 (Z-band associated protein)	0046324 // regulation of glucose import // inferred from mutant phenotype///0065002 // int
NM_172793	0.034	-1.684	***Btnl9***	butyrophilin-like 9	0016020 // membrane // inferred from electronic annotation///0016021 // integral compone
NM_001111051///NM_	0.014	-1.682	***Dclk1***	doublecortin-like kinase 1	0001764 // neuron migration // inferred from genetic interaction///0006468 // protein phosp
NM_001025564///NR_	0.010	-1.680	***Gm15698***	transcription elongation factor B (SIII), polypeptide 2 pseudogene	0006368 // transcription elongation from RNA polymerase II promoter // not recorded
NM_001164528///XM_	0.020	-1.679	***Ildr2***	immunoglobulin-like domain containing receptor 2	0009749 // response to glucose // inferred from mutant phenotype///0030073 // insulin secr
NM_134102///XM_006	0.000	-1.676	***Pla1a***	phospholipase A1 member A	0006629 // lipid metabolic process // inferred from electronic annotation///0016042 // lipid c
NM_001276446///NM_	0.001	-1.663	***Alad***	aminolevulinate, delta-, dehydratase	0001666 // response to hypoxia // inferred from electronic annotation///0006779 // porphyri
NM_020052	0.043	-1.658	***Scube2***	signal peptide, CUB domain, EGF-like 2	0005509 // calcium ion binding // inferred from electronic annotation///0042802 // identical
NM_027391	0.045	-1.655	***Iyd***	iodotyrosine deiodinase	0055114 // oxidation-reduction process // inferred from electronic annotation
NM_001166173///NM_	0.037	-1.654	***Dmkn***	dermokine	0030154 // cell differentiation // inferred from electronic annotation///1903575 // cornified e
NM_011758	0.012	-1.651	***Zfp39***	zinc finger protein 39	0006351 // transcription, DNA-templated // inferred from electronic annotation///0006355 /
NM_001205369///NM_	0.033	-1.645	***Casc4***	cancer susceptibility candidate 4	0005794 // Golgi apparatus // not recorded///0016020 // membrane // inferred from electro
NM_013813///XM_006	0.008	-1.634	***Epb41l3***	erythrocyte membrane protein band 4.1 like 3	0001558 // regulation of cell growth // inferred from electronic annotation///0002175 // prot
NM_026864	0.028	-1.634	***Rasl11a***	RAS-like, family 11, member A	0006351 // transcription, DNA-templated // inferred from electronic annotation///0006355 /
NM_028130///XM_006	0.017	-1.631	***Zfp157***	zinc finger protein 157	0006355 // regulation of transcription, DNA-templated // inferred from electronic annotation
NM_029360	0.020	-1.626	***Tm4sf5***	transmembrane 4 superfamily member 5	0016020 // membrane // inferred from electronic annotation///0016021 // integral compone
NM_175140///XM_006	0.045	-1.624	***Chst8***	carbohydrate (N-acetylgalactosamine 4-0) sulfotransferase 8	0005975 // carbohydrate metabolic process // inferred from electronic annotation///000679
NM_025949///XM_006	0.040	-1.624	***Rps6ka6***	ribosomal protein S6 kinase polypeptide 6	0006417 // regulation of translation // not recorded///0006468 // protein phosphorylation //
NM_010516	0.001	-1.620	***Cyr61***	cysteine rich protein 61	0001558 // regulation of cell growth // inferred from electronic annotation///0001649 // oste
NM_008083	0.042	-1.619	***Gap43***	growth associated protein 43	0007275 // multicellular organismal development // inferred from electronic annotation///00
NM_175666	0.002	-1.617	***Hist2h2bb***	histone cluster 2, H2bb	0006334 // nucleosome assembly // not recorded
NM_001166364///NM_	0.010	-1.612	***Fam219b***	family with sequence similarity 219, member B	
NM_001024720///XM_	0.007	-1.607	***Hmcn1***	hemicentin 1	0005509 // calcium ion binding // inferred from electronic annotation
NM_013476	0.010	-1.602	***Ar***	androgen receptor	0001701 // in utero embryonic development // inferred from mutant phenotype///0003073 /
NM_001313978///NM_	0.019	-1.596	***Colec11***	collectin sub-family member 11	0007275 // multicellular organismal development // inferred from electronic annotation///00
NM_001017525///NM_	0.010	-1.596	***Btbd11***	BTB (POZ) domain containing 11	0060395 // SMAD protein signal transduction // inferred from direct assay
NM_001039536///NM_	0.008	-1.593	***Pigl***	phosphatidylinositol glycan anchor biosynthesis, class L	0006506 // GPI anchor biosynthetic process // not recorded
NM_028095///XM_006	0.004	-1.589	***Mettl10***	methyltransferase like 10	0032259 // methylation // inferred from electronic annotation
NM_001025208///NM_	0.026	-1.584	***Gm38417///H2-Bl///H2-D4///H2-T3-li***	predicted gene, 38417///histocompatibility 2, blastocyst///histoc	0001916 // positive regulation of T cell mediated cytotoxicity // inferred from direct assay///0
NM_001136077///NM_	0.037	-1.584	***Enpp2***	ectonucleotide pyrophosphatase/phosphodiesterase 2	0001953 // negative regulation of cell-matrix adhesion // not recorded///0006629 // lipid me
NM_013912	0.002	-1.584	***Apln***	apelin	0002026 // regulation of the force of heart contraction // not recorded///0007165 // signal tr

RefSeq Transcript ID	p	FD Air	*Gene Symbol*	Gene Title	Gene Ontology
NM_001136260///NM_	0.033	-1.580	***Slc4a4***	solute carrier family 4 (anion exchanger), member 4	0006810 // transport // inferred from electronic annotation///0006811 // ion transport // infe
NM_001141933///NM_	0.024	-1.574	***Nkain4***	Na+/K+ transporting ATPase interacting 4	0002028 // regulation of sodium ion transport // ---
NM_011985///XM_006	0.005	-1.574	***Mmp23***	matrix metallopeptidase 23	0000003 // reproduction // inferred from sequence or structural similarity///0000003 // repro
NM_001081324///XM_	0.030	-1.571	***Neto2***	neuropilin (NRP) and tolloid (TLL)-like 2	2000312 // regulation of kainate selective glutamate receptor activity // inferred from electro
NM_007646	0.011	-1.567	***Cd38***	CD38 antigen	0001666 // response to hypoxia // not recorded///0007204 // positive regulation of cytosolic
NM_024273///XM_006	0.001	-1.565	***Timmdc1***	translocase of inner mitochondrial membrane domain containing	0005515 // protein binding // inferred from electronic annotation
NM_009738///XM_011	0.019	-1.562	***Bche***	butyrylcholinesterase	0006581 // acetylcholine catabolic process // ---///0007268 // synaptic transmission // ---///0
NM_028097///XM_006	0.038	-1.551	***Tmem68***	transmembrane protein 68	0008152 // metabolic process // inferred from electronic annotation
NM_170778///XM_011	0.031	-1.547	***Dpyd***	dihydropyrimidine dehydrogenase	0006145 // purine nucleobase catabolic process // not recorded///0006208 // pyrimidine nuc
NM_001308535///NM_	0.003	-1.538	***Il2rg***	interleukin 2 receptor, gamma chain	0010468 // regulation of gene expression // inferred from mutant phenotype///0010628 // po
NM_001165939///NM_	0.011	-1.535	***Tshb***	thyroid stimulating hormone, beta subunit	0033189 // response to vitamin A // inferred from electronic annotation///0043627 // respon
NM_001083912///NM_	0.025	-1.530	***Plekhg2***	pleckstrin homology domain containing, family G (with RhoGef do	0035023 // regulation of Rho protein signal transduction // inferred from electronic annotatio
NM_009778///XM_011	0.004	-1.530	***C3***	complement component 3	0001798 // positive regulation of type IIa hypersensitivity // inferred from mutant phenotype,
NM_011352///XM_006	0.015	-1.528	***Sema7a***	sema domain, immunoglobulin domain (Ig), and GPI membrane a	0001649 // osteoblast differentiation // not recorded///0001755 // neural crest cell migration
NM_018874	0.013	-1.528	***Pnliprp1***	pancreatic lipase related protein 1	0006629 // lipid metabolic process // inferred from electronic annotation
NM_001310635///NM_	0.015	-1.527	***Crispld2***	cysteine-rich secretory protein LCCL domain containing 2	0030198 // extracellular matrix organization // inferred from direct assay///0030324 // lung d
NM_008727///XM_006	0.001	-1.524	***Npr1***	natriuretic peptide receptor 1	0006182 // cGMP biosynthetic process // not recorded///0006468 // protein phosphorylation
NM_024472	0.005	-1.523	***Cptp***	ceramide-1-phosphate transfer protein	0006810 // transport // inferred from electronic annotation///0006869 // lipid transport // in
NM_199446///XM_006	0.010	-1.521	***Phkb***	phosphorylase kinase beta	0005975 // carbohydrate metabolic process // inferred from electronic annotation///0005977
NM_001252563///NM_	0.029	-1.519	***Matn4***	matrilin 4	0048678 // response to axon injury // inferred from direct assay
NM_001033416///XM_	0.036	-1.516	***Gal3st4***	galactose-3-O-sulfotransferase 4	0009058 // biosynthetic process // inferred from electronic annotation///0009101 // glycopro
NM_021344///XR_387	0.026	-1.515	***Tesc***	tescalcin	0006810 // transport // inferred from electronic annotation///0006883 // cellular sodium ion
NM_001289780///NM_	0.005	-1.514	***Pus7***	pseudouridylate synthase 7 homolog (S. cerevisiae)	0001522 // pseudouridine synthesis // inferred from electronic annotation///0001522 // pseu
NM_001290457///NM_	0.002	-1.512	***Relb***	avian reticuloendotheliosis viral (v-rel) oncogene related B	0000122 // negative regulation of transcription from RNA polymerase II promoter // not recor
NM_011823///XM_006	0.036	-1.510	***Gpr34***	G protein-coupled receptor 34	0007165 // signal transduction // inferred from electronic annotation///0007186 // G-protein
NM_001039201///NM_	0.024	-1.510	***Hdhd2***	haloacid dehalogenase-like hydrolase domain containing 2	0005515 // protein binding // inferred from electronic annotation///0019899 // enzyme bindi
NM_001025373	0.038	-1.507	***Zfp943***	zinc finger prtoein 943	0006355 // regulation of transcription, DNA-templated // inferred from electronic annotation
NM_008939	0.007	-1.505	***Prss12***	protease, serine 12 neurotrypsin (motopsin)	0006508 // proteolysis // inferred from direct assay///0006508 // proteolysis // inferred from
NM_011075///XM_006	0.024	-1.499	***Abcb1b***	ATP-binding cassette, sub-family B (MDR/TAP), member 1B	0006810 // transport // not recorded///0006855 // drug transmembrane transport // not reco
NM_172947///XM_006	0.002	-1.497	***Lsm12***	LSM12 homolog (S. cerevisiae)	
NM_001290708///NM_	0.046	-1.496	***Smarca1***	SWI/SNF related, matrix associated, actin dependent regulator of	0000733 // DNA strand renaturation // not recorded///0006338 // chromatin remodeling // n
NM_001008549///XM_	0.036	-1.495	***Zfp658***	zinc finger protein 658	0006355 // regulation of transcription, DNA-templated // inferred from electronic annotation
NM_173781///XM_006	0.028	-1.495	***Rab6b***	RAB6B, member RAS oncogene family	0006810 // transport // inferred from electronic annotation///0006886 // intracellular protein
NM_001013785///XR_	0.001	-1.492	***Akr1c19***	aldo-keto reductase family 1, member C19	0055114 // oxidation-reduction process // inferred from electronic annotation
NM_138672///XM_006	0.028	-1.489	***Stab1***	stabilin 1	0006898 // receptor-mediated endocytosis // ---///0006898 // receptor-mediated endocytosis
NM_018732///XM_006	0.019	-1.488	***Scn3a***	sodium channel, voltage-gated, type III, alpha	0006810 // transport // inferred from electronic annotation///0006811 // ion transport // infe
NM_009199	0.021	-1.488	***Slc1a1***	solute carrier family 1 (neuronal/epithelial high affinity glutamate	0006810 // transport // inferred from electronic annotation///0006835 // dicarboxylic acid tra
NM_001168505///NM_	0.006	-1.486	***Shoc2***	soc-2 (suppressor of clear) homolog (C. elegans)	0046579 // positive regulation of Ras protein signal transduction // not recorded
NM_013705	0.005	-1.486	***Zfp30***	zinc finger protein 30	0006351 // transcription, DNA-templated // inferred from electronic annotation///0006355 //
NM_001243008///NM_	0.019	-1.485	***Col6a3***	collagen, type VI, alpha 3	0010951 // negative regulation of endopeptidase activity // inferred from electronic annotatio
NM_001110100///NM_	0.049	-1.485	***Banp***	BTG3 associated nuclear protein	0006351 // transcription, DNA-templated // inferred from electronic annotation///0006355 //
NM_152812///XM_011	0.038	-1.484	***Otud6b***	OTU domain containing 6B	0006508 // proteolysis // inferred from electronic annotation
NM_001163847///NM_	0.042	-1.480	***Ntn3///Tbc1d24***	netrin 3///TBC1 domain family, member 24	0006355 // regulation of transcription, DNA-templated // not recorded///0007409 // axonoge
NM_001037842///NM_	0.007	-1.480	***Cml3///Gm11128///Gm4477***	camello-like 3///predicted gene 11128///predicted gene 4477	0001702 // gastrulation with mouth forming second // non-traceable author statement///000
NM_001033633	0.048	-1.480	***Slc2a13***	solute carrier family 2 (facilitated glucose transporter), member 1	0006810 // transport // inferred from electronic annotation///0008643 // carbohydrate trans
NM_023320///XM_006	0.006	-1.480	***Plekho1***	pleckstrin homology domain containing, family O member 1	0007520 // myoblast fusion // inferred from direct assay///0007520 // myoblast fusion // infe
NM_011221	0.005	-1.477	***Purb***	purine rich element binding protein B	0000122 // negative regulation of transcription from RNA polymerase II promoter // inferred
NM_001081401///NM_	0.018	-1.477	***Adamts3***	a disintegrin-like and metallopeptidase (reprolysin type) with thro	0006508 // proteolysis // inferred from electronic annotation///0007229 // integrin-mediated
NM_001033310///NM_	0.022	-1.474	***Cox18***	cytochrome c oxidase assembly protein 18	0008535 // respiratory chain complex IV assembly // not recorded///0015031 // protein trans
NM_024257	0.002	-1.472	***Hdhd3***	haloacid dehalogenase-like hydrolase domain containing 3	0008152 // metabolic process // inferred from electronic annotation
NM_134160///XM_006	0.004	-1.470	***Mcoln3***	mucolipin 3	0006810 // transport // inferred from electronic annotation///0006811 // ion transport // infe
NM_001042592///NM_	0.015	-1.469	***Arrdc4***	arrestin domain containing 4	0051443 // positive regulation of ubiquitin-protein transferase activity // not recorded
NM_001253754///NM_	0.029	-1.469	***Gpm6a***	glycoprotein m6a	0001764 // neuron migration // not recorded///0003407 // neural retina development // infe
NM_028279///XM_006	0.009	-1.468	***Naalad2***	N-acetylated alpha-linked acidic dipeptidase 2	0006508 // proteolysis // inferred from direct assay///0008152 // metabolic process // inferre

RefSeq Transcript ID	p	FD Air	*Gene Symbol*	Gene Title	Gene Ontology
NM_025864	0.006	-1.467	***Tmem206***	transmembrane protein 206	0009986 // cell surface // not recorded///0016020 // membrane // inferred from electronic a
NM_029658	0.008	-1.467	***Fam101b***	family with sequence similarity 101, member B	0001837 // epithelial to mesenchymal transition // inferred from genetic interaction///00300
NM_001205095///XM_	0.014	-1.467	***Gm4944***	predicted gene 4944	0006355 // regulation of transcription, DNA-templated // not recorded
NM_001313897///NM_	0.008	-1.466	***Lta4h***	leukotriene A4 hydrolase	0006508 // proteolysis // inferred from electronic annotation///0006691 // leukotriene meta
NM_133972///XM_01	0.044	-1.466	***Armc6***	armadillo repeat containing 6	0002244 // hematopoietic progenitor cell differentiation // inferred from genetic interaction
NM_139063///XM_00	0.005	-1.463	***Bloc1s5***	biogenesis of lysosomal organelles complex-1, subunit 5, muted	0008089 // anterograde axon cargo transport // inferred from mutant phenotype///0016192
NM_026062	0.021	-1.462	***Fam69a***	family with sequence similarity 69, member A	0005515 // protein binding // inferred from physical interaction
NM_019472///XM_00	0.012	-1.461	***Myo10***	myosin X	0006810 // transport // inferred from electronic annotation///0007165 // signal transduction
NM_026504///XR_387	0.017	-1.460	***Coq5***	coenzyme Q5 homolog, methyltransferase (yeast)	0006744 // ubiquinone biosynthetic process // inferred from electronic annotation///00322
NR_045175	0.015	-1.459	***Smc2os***	structural maintenance of chromosomes 2, opposite strand	
NM_146106///XM_01	0.041	-1.457	***Lyplal1***	lysophospholipase-like 1	0002084 // protein depalmitoylation // inferred from mutant phenotype///0042997 // negati
NM_026796///XM_00	0.030	-1.457	***Smyd2***	SET and MYND domain containing 2	0000122 // negative regulation of transcription from RNA polymerase II promoter // not reco
NM_008369///XM_00	0.010	-1.455	***Il3ra***	interleukin 3 receptor, alpha chain	0019221 // cytokine-mediated signaling pathway // inferred from electronic annotation
NM_001159564///NM_	0.034	-1.455	***Itgb6***	integrin beta 6	0006954 // inflammatory response // inferred from mutant phenotype///0007155 // cell adh
NM_029494///XM_00	0.015	-1.452	***Rab30***	RAB30, member RAS oncogene family	0006886 // intracellular protein transport // not recorded///0007030 // Golgi organization //
NM_176952///XM_00	0.033	-1.450	***6430573F11Rik***	RIKEN cDNA 6430573F11 gene	0008033 // tRNA processing // inferred from electronic annotation///0008152 // metabolic pr
NM_023245	0.003	-1.450	***Palmd***	palmdelphin	0008360 // regulation of cell shape // inferred from electronic annotation
NR_033626	0.004	1.451	***1810010D01Rik***	RIKEN cDNA 1810010D01 gene	0006810 // transport // inferred from electronic annotation///0006811 // ion transport // inf
NM_001289428///NM_	0.005	1.452	***Pira1///Pira2///Pira6///Gm10693///***	paired-Ig-like receptor A1///paired-Ig-like receptor A2///paired-I	0001782 // B cell homeostasis // inferred from mutant phenotype///0002250 // adaptive imn
NM_008184///XM_00	0.016	1.454	***Gstm6***	glutathione S-transferase, mu 6	0008152 // metabolic process // inferred from electronic annotation
NM_029152	0.008	1.454	***Efcab10***	EF-hand calcium binding domain 10	0005509 // calcium ion binding // inferred from electronic annotation
NM_008030	0.010	1.462	***Fmo3***	flavin containing monooxygenase 3	0017144 // drug metabolic process // not recorded///0055114 // oxidation-reduction process
NM_001313712///NM_	0.001	1.467	***Sp100***	nuclear antigen Sp100	0000723 // telomere maintenance // inferred from sequence or structural similarity///00063
NM_001040005///XM_	0.003	1.469	***Rnf213***	ring finger protein 213	0008152 // metabolic process // inferred from electronic annotation///0016567 // protein ub
NM_153598	0.006	1.469	***Ugt2b34***	UDP glucuronosyltransferase 2 family, polypeptide B34	0008152 // metabolic process // inferred from electronic annotation///0009813 // flavonoid
NM_001037138///NM_	0.015	1.471	***Cd300lb***	CD300 antigen like family member B	0002376 // immune system process // inferred from electronic annotation///0002446 // neut
NM_001161667///NM_	0.011	1.472	***Acox2***	acyl-Coenzyme A oxidase 2, branched chain	0006629 // lipid metabolic process // inferred from electronic annotation///0006631 // fatty
NM_001002898///NM_	0.003	1.474	***Sirpb1a///Sirpb1b***	signal-regulatory protein beta 1-like///signal-regulatory protein b	0035556 // intracellular signal transduction // inferred from direct assay///0050766 // positiv
NM_030098///XM_00	0.025	1.481	***Rnase6***	ribonuclease, RNase A family, 6	0008152 // metabolic process // inferred from electronic annotation///0090305 // nucleic aci
NM_001134399///NM_	0.009	1.483	***Megf11***	multiple EGF-like-domains 11	0010842 // retina layer formation // inferred from mutant phenotype///0034109 // homotyp
NM_001161775///NM_	0.012	1.483	***Myh11***	myosin, heavy polypeptide 11, smooth muscle	0006939 // smooth muscle contraction // inferred from direct assay///0006939 // smooth mu
NM_011452	0.002	1.487	***Serpinb9b***	serine (or cysteine) peptidase inhibitor, clade B, member 9b	0010951 // negative regulation of endopeptidase activity // not recorded///0019835 // cytoly
NM_008630	0.006	1.487	***Mt2***	metallothionein 2	0006882 // cellular zinc ion homeostasis // inferred from mutant phenotype///0007263 // nit
NM_001205313///NM_	0.000	1.487	***Stat1***	signal transducer and activator of transcription 1	0000122 // negative regulation of transcription from RNA polymerase II promoter // inferred
NM_001113468///NR_	0.002	1.489	***Vmn2r29***	vomeronasal 2, receptor 29	0007186 // G-protein coupled receptor signaling pathway // inferred from electronic annotati
NM_001313970///NM_	0.025	1.490	***Ppp1r1b***	protein phosphatase 1, regulatory (inhibitor) subunit 1B	0001975 // response to amphetamine // inferred from genetic interaction///0006351 // trans
NM_010734	0.004	1.490	***Lst1***	leukocyte specific transcript 1	0000902 // cell morphogenesis // inferred from electronic annotation///0002376 // immune
NM_001164289///NM_	0.000	1.490	***Phf11c///Phf11d***	PHD finger protein 11C///PHD finger protein 11D	0006351 // transcription, DNA-templated // inferred from electronic annotation///0006355 /
NM_001103156///NM_	0.001	1.496	***Steap2***	six transmembrane epithelial antigen of prostate 2	0006810 // transport // inferred from electronic annotation///0006811 // ion transport // inf
NM_009999	0.016	1.498	***Cyp2b10***	cytochrome P450, family 2, subfamily b, polypeptide 10	0006805 // xenobiotic metabolic process // not recorded///0008202 // steroid metabolic pro
NM_001081029///XR_	0.000	1.501	***Tmem243///Gm31539***	transmembrane protein 243, mitochondrial///predicted gene, 31	0016020 // membrane // inferred from electronic annotation///0016021 // integral compone
NM_016681///XM_00	0.029	1.503	***Chek2***	checkpoint kinase 2	0000077 // DNA damage checkpoint // not recorded///0000086 // G2/M transition of mitotic
NM_001291892///NM_	0.037	1.503	***Lilr4b///Lilrb4a***	leukocyte immunoglobulin-like receptor, subfamily B, member 4	0002250 // adaptive immune response // inferred from electronic annotation///0002376 // i
NM_001291892///NM_	0.037	1.503	***Lilr4b///Lilrb4a***	leukocyte immunoglobulin-like receptor, subfamily B, member 4	0002250 // adaptive immune response // inferred from electronic annotation///0002376 // i
NM_007440///XM_01	0.002	1.504	***Alox12***	arachidonate 12-lipoxygenase	0006629 // lipid metabolic process // inferred from electronic annotation///0006631 // fatty
NM_029309	0.047	1.505	***Morn5***	MORN repeat containing 5	
NM_001168469///NM_	0.013	1.506	***Ube2m***	ubiquitin-conjugating enzyme E2M	0006464 // cellular protein modification process // not recorded///0016567 // protein ubiqu
NM_010745	0.032	1.509	***Ly86***	lymphocyte antigen 86	0002376 // immune system process // inferred from electronic annotation///0006954 // infla
NM_172796///XM_00	0.001	1.510	***Slfn9***	schlafen 9	0051607 // defense response to virus // inferred from electronic annotation///2000134 // ne
NM_001081957///XM_	0.049	1.510	***Wfdc17***	WAP four-disulfide core domain protein 18-like///WAP four-disul	0010466 // negative regulation of peptidase activity // inferred from electronic annotation///
NM_010185	0.009	1.514	***Fcer1g***	Fc receptor, IgE, high affinity I, gamma polypeptide	0001798 // positive regulation of type IIa hypersensitivity // inferred from mutant phenotype
NM_001160386///XM_	0.026	1.516	***Dnah7b***	dynein, axonemal, heavy chain 7B	0003341 // cilium movement // inferred from electronic annotation///0007018 // microtubul
NM_008148	0.001	1.520	***Gp5***	glycoprotein 5 (platelet)	0007160 // cell-matrix adhesion // inferred from mutant phenotype///0007409 // axonogene
NM_001033444///XM_	0.005	1.530	***Capn13***	calpain 13	0006508 // proteolysis // not recorded

RefSeq Transcript ID	p	FD Air	*Gene Symbol*	Gene Title	Gene Ontology
NM_013478	0.012	1.533	***Azgp1***	alpha-2-glycoprotein 1, zinc	0001580 // detection of chemical stimulus involved in sensory perception of bitter taste // no
NM_133365///XM_006	0.005	1.535	***Dnah5***	dynein, axonemal, heavy chain 5	0003341 // cilium movement // inferred from mutant phenotype///0003341 // cilium movem
NM_022430	0.027	1.536	***Ms4a8a***	membrane-spanning 4-domains, subfamily A, member 8A	0016020 // membrane // inferred from electronic annotation///0016021 // integral compone
NM_023124///NM_20	0.017	1.540	***H2-Q6///H2-Q8///H2-Q4***	histocompatibility 2, Q region locus 6///histocompatibility 2, Q re	0002474 // antigen processing and presentation of peptide antigen via MHC class I // not reco
NM_001001892///NM_	0.013	1.543	***H2-D1///H2-K1///H2-L///H2-Q2///H2***	histocompatibility 2, D region locus 1///histocompatibility 2, K1, I	0001916 // positive regulation of T cell mediated cytotoxicity // inferred from direct assay///0
NM_001177900///NM_	0.029	1.543	***Rd3***	retinal degeneration 3	0007601 // visual perception // inferred from electronic annotation///0050896 // response to
NM_001170851///NM_	0.034	1.544	***Klra2***	killer cell lectin-like receptor, subfamily A, member 2	0007155 // cell adhesion // inferred from electronic annotation
NM_019440	0.001	1.545	***Irgm2***	immunity-related GTPase family M member 2	0034341 // response to interferon-gamma // inferred from direct assay///0034341 // respons
NM_001081369///XM_	0.029	1.549	***Ccdc153***	coiled-coil domain containing 153	0005515 // protein binding // inferred from electronic annotation
NM_001039160///NM_	0.001	1.552	***Gm4070///Gvin1***	predicted gene 4070///GTPase, very large interferon inducible 1	0000166 // nucleotide binding // inferred from electronic annotation///0005525 // GTP bindi
NM_053085///XM_006	0.009	1.552	***Tcf23***	transcription factor 23	0007275 // multicellular organismal development // inferred from electronic annotation///00
NM_030150	0.007	1.553	***Dhx58***	DEXH (Asp-Glu-X-His) box polypeptide 58	0002376 // immune system process // inferred from electronic annotation///0008152 // meta
NM_001172207///NM_	0.047	1.555	***Lrtm2***	leucine-rich repeats and transmembrane domains 2	0007409 // axonogenesis // not recorded///0051965 // positive regulation of synapse assemb
NM_001080707///NM_	0.001	1.556	***Gpr155***	G protein-coupled receptor 155	0035556 // intracellular signal transduction // inferred from electronic annotation///0050890
NM_130452///XM_006	0.006	1.557	***Bbox1***	butyrobetaine (gamma), 2-oxoglutarate dioxygenase 1 (gamma-b	0045329 // carnitine biosynthetic process // not recorded///0055114 // oxidation-reduction p
NM_009252///XM_011	0.031	1.558	***Serpina3n***	serine (or cysteine) peptidase inhibitor, clade A, member 3N	0006953 // acute-phase response // inferred from electronic annotation///0010466 // negativ
NM_008240///XM_006	0.002	1.561	***Foxj1***	forkhead box J1	0000122 // negative regulation of transcription from RNA polymerase II promoter // inferred
NM_177546///NM_21	0.001	1.562	***Pcyt1b***	phosphate cytidylyltransferase 1, choline, beta isoform	0001541 // ovarian follicle development // inferred from mutant phenotype///0006629 // lipi
NM_021332	0.022	1.563	***Glp1r***	glucagon-like peptide 1 receptor	0016020 // membrane // inferred from electronic annotation///0016021 // integral compone
NM_027209///XM_011	0.003	1.564	***Ms4a6b***	membrane-spanning 4-domains, subfamily A, member 6B	
NM_008039	0.000	1.567	***Fpr2***	formyl peptide receptor 2	0006935 // chemotaxis // inferred from electronic annotation///0007165 // signal transductic
NM_029762	0.015	1.567	***Hyls1***	hydrolethalus syndrome 1	
NM_009201///XM_006	0.022	1.572	***Slc1a5***	solute carrier family 1 (neutral amino acid transporter), member !	0003333 // amino acid transmembrane transport // not recorded///0006810 // transport // ir
NM_133990	0.003	1.572	***Il13ra1***	interleukin 13 receptor, alpha 1	0019221 // cytokine-mediated signaling pathway // inferred from electronic annotation///003
NM_198095	0.001	1.572	***Bst2***	bone marrow stromal cell antigen 2	0002376 // immune system process // inferred from electronic annotation///0002737 // nega
NM_001302338///NM_	0.005	1.573	***Nfe2***	nuclear factor, erythroid derived 2	0006351 // transcription, DNA-templated // inferred from electronic annotation///0006355 /,
NM_175467///XM_006	0.032	1.573	***Sptlc3***	serine palmitoyltransferase, long chain base subunit 3	0006629 // lipid metabolic process // inferred from electronic annotation///0006665 // sphin
NM_001159417///NM_	0.001	1.584	***Irf9***	interferon regulatory factor 9	0006351 // transcription, DNA-templated // inferred from electronic annotation///0006355 /,
NM_001204241///NM_	0.030	1.585	***Clec4a3***	C-type lectin domain family 4, member a3	0006508 // proteolysis // inferred from electronic annotation
NM_001112725///NM_	0.017	1.588	***Aldh3a1***	aldehyde dehydrogenase family 3, subfamily A1	0001666 // response to hypoxia // not recorded///0006081 // cellular aldehyde metabolic pro
NM_001166376///NM_	0.009	1.590	***Ms4a6c///Gm8369***	membrane-spanning 4-domains, subfamily A, member 6C///pred	0016020 // membrane // inferred from electronic annotation///0016021 // integral compone
NM_145825	0.014	1.594	***Cetn4***	centrin 4	0005509 // calcium ion binding // inferred from direct assay///0031683 // G-protein beta/gar
NM_029495///NM_17	0.000	1.602	***Epsti1***	epithelial stromal interaction 1 (breast)	
NM_009841	0.013	1.605	***Cd14***	CD14 antigen	0002237 // response to molecule of bacterial origin // inferred from direct assay///0002376 /,
NM_028377///XM_006	0.011	1.609	***Bbof1***	basal body orientation factor 1	0044458 // motile cilium assembly // inferred from sequence or structural similarity
NM_001110323///NM_	0.004	1.611	***Klra7///LOC100862438***	killer cell lectin-like receptor, subfamily A, member 7///killer cell I	0007155 // cell adhesion // inferred from electronic annotation
NM_007807///XM_006	0.019	1.613	***Cybb***	cytochrome b-245, beta polypeptide	0006801 // superoxide metabolic process // inferred from mutant phenotype///0006801 // su
NM_008326///XM_006	0.000	1.619	***Irgm1***	immunity-related GTPase family M member 1	0002376 // immune system process // inferred from electronic annotation///0006914 // auto
NM_029112///XM_006	0.019	1.620	***Morn3***	MORN repeat containing 3	
NM_001042451///NM_	0.019	1.620	***Snca***	synuclein, alpha	0001774 // microglial cell activation // inferred from mutant phenotype///0001921 // positive
NM_029007	0.005	1.632	***Fam84a***	family with sequence similarity 84, member A	
NM_194336///XM_006	0.000	1.634	***Gbp6***	guanylate binding protein 6	0006955 // immune response // inferred from mutant phenotype///0008152 // metabolic pro
NM_029383	0.029	1.637	***Cldn22***	claudin 22	0016338 // calcium-independent cell-cell adhesion via plasma membrane cell-adhesion molec
NM_177753	0.009	1.640	***Sox21***	SRY (sex determining region Y)-box 21	0001942 // hair follicle development // inferred from mutant phenotype///0006351 // transc
NM_144559	0.001	1.641	***Fcgr4***	Fc receptor, IgG, low affinity IV	0001866 // NK T cell proliferation // not recorded///0051930 // regulation of sensory percept
NM_019807///NM_20	0.049	1.646	***Acpp***	acid phosphatase, prostate	0006144 // purine nucleobase metabolic process // inferred from mutant phenotype///00064
NM_001316689///NM_	0.030	1.647	***Ern2***	endoplasmic reticulum (ER) to nucleus signalling 2	0006351 // transcription, DNA-templated // inferred from electronic annotation///0006355 /,
NM_001145948///NM_	0.007	1.649	***Ttc39a***	tetratricopeptide repeat domain 39A	
NM_021407///XM_006	0.007	1.653	***Trem3***	triggering receptor expressed on myeloid cells 3	0002374 // cytokine secretion involved in immune response // inferred from genetic interacti
NM_016925///XM_006	0.014	1.653	***Fanca***	Fanconi anemia, complementation group A	0006281 // DNA repair // inferred from electronic annotation///0006974 // cellular response
NM_009915///XM_006	0.003	1.654	***Ccr2***	chemokine (C-C motif) receptor 2	0001525 // angiogenesis // traceable author statement///0001974 // blood vessel remodeling
NM_178386	0.014	1.657	***Slc25a31***	solute carrier family 25 (mitochondrial carrier; adenine nucleotide	0006810 // transport // inferred from electronic annotation///0055085 // transmembrane tra
NM_007417	0.015	1.658	***Adra2a***	adrenergic receptor, alpha 2a	0001819 // positive regulation of cytokine production // not recorded///0002526 // acute infl
NM_001122635///NM_	0.006	1.662	***Cdhr4***	cadherin-related family member 4	0007155 // cell adhesion // inferred from electronic annotation///0007156 // homophilic cell

RefSeq Transcript ID	p	FD Air	*Gene Symbol*	Gene Title	Gene Ontology
NM_028275///XM_006	0.025	1.665	***1700112E06Rik***	RIKEN cDNA 1700112E06 gene	0030154 // cell differentiation // inferred from electronic annotation///0030318 // melanocyt
NM_029662///XM_006	0.016	1.671	***Mfsd2a***	major facilitator superfamily domain containing 2A	0006810 // transport // inferred from electronic annotation///0006869 // lipid transport // in
NM_001174053///NM_	0.044	1.679	***Camk2b***	calcium/calmodulin-dependent protein kinase II, beta	0000082 // G1/S transition of mitotic cell cycle // inferred from mutant phenotype///000203C
NM_011408	0.000	1.681	***Slfn2***	schlafen 2	0008285 // negative regulation of cell proliferation // inferred from direct assay
NM_011023	0.036	1.686	***Otx1***	orthodenticle homolog 1	0006355 // regulation of transcription, DNA-templated // inferred from electronic annotation
NM_181589///XM_006	0.001	1.689	***Ckap2l***	cytoskeleton associated protein 2-like	0000922 // spindle pole // inferred from electronic annotation///0005737 // cytoplasm // infe
NM_007689	0.001	1.697	***Chad***	chondroadherin	0001502 // cartilage condensation // inferred from electronic annotation///0060348 // bone
NM_019948///XM_011	0.044	1.699	***Clec4e***	C-type lectin domain family 4, member e	0002292 // T cell differentiation involved in immune response // inferred from mutant pheno
NM_145226	0.033	1.700	***Oas3***	2'-5' oligoadenylate synthetase 3	0002376 // immune system process // inferred from electronic annotation///0006164 // puri
NM_001166627///NM_	0.000	1.702	***Dynlt1a///Dynlt1b///Dynlt1c///Dynlt***	dynein light chain Tctex-type 1A///dynein light chain Tctex-type 1	0000132 // establishment of mitotic spindle orientation // inferred from mutant phenotype//
NM_001101605///NM_	0.002	1.703	***Ifit1bl1***	interferon induced protein with tetratricpeptide repeats 1B like 1	0051607 // defense response to virus // not recorded///0060337 // type I interferon signaling
NM_001167743///NM_	0.048	1.704	***Slfn8***	schlafen 8	0051607 // defense response to virus // inferred from electronic annotation///2000134 // ne
NM_024444///XM_006	0.006	1.710	***Cyp4f18***	cytochrome P450, family 4, subfamily f, polypeptide 18	0000038 // very long-chain fatty acid metabolic process // not recorded///0001676 // long-ch
NM_153094///NR_024	0.003	1.715	***Klrb1f***	killer cell lectin-like receptor subfamily B member 1F	0004872 // receptor activity // inferred from direct assay///0005515 // protein binding // infe
NM_010654///XM_006	0.019	1.727	***Klrd1***	killer cell lectin-like receptor, subfamily D, member 1	0002228 // natural killer cell mediated immunity // not recorded
NM_001301353///NM_	0.018	1.731	***Apod***	apolipoprotein D	0000302 // response to reactive oxygen species // inferred from mutant phenotype///00030
NM_023785	0.009	1.732	***Ppbp***	pro-platelet basic protein	0002523 // leukocyte migration involved in inflammatory response // inferred from mutant p
XM_006521609///XM_	0.036	1.734	***Fer1l6***	fer-1-like 6 (C. elegans)	
NM_001033170///XM_	0.023	1.735	***Fam83e***	family with sequence similarity 83, member E	0019901 // protein kinase binding // not recorded
NM_001162878///NM_	0.015	1.742	***Fam183b***	family with sequence similarity 183, member B	
NM_009610	0.032	1.746	***Actg2***	actin, gamma 2, smooth muscle, enteric	0010628 // positive regulation of gene expression // inferred from sequence or structural sim
NM_010186	0.000	1.754	***Fcgr1***	Fc receptor, IgG, high affinity I	0001788 // antibody-dependent cellular cytotoxicity // inferred from mutant phenotype///00
NM_011073///XM_006	0.000	1.756	***Prf1***	perforin 1 (pore forming protein)	0001771 // immunological synapse formation // not recorded///0002357 // defense response
NM_016974///XM_006	0.033	1.765	***Dbp***	D site albumin promoter binding protein	0001889 // liver development // inferred from electronic annotation///0006351 // transcripti
NM_001033450///NM_	0.026	1.770	***Ifi204///Ifi205///Mnda///Mndal***	interferon activated gene 204///interferon activated gene 205///	0000122 // negative regulation of transcription from RNA polymerase II promoter // ---///000
NM_001166627///NM_	0.000	1.785	***Dynlt1-ps1///Dynlt1a///Dynlt1b///Dy***	dynein light chain Tctex-type 1, pseudogene 1///dynein light cha	0000132 // establishment of mitotic spindle orientation // inferred from mutant phenotype//
NM_080457///NM_183	0.004	1.789	***Muc4***	mucin 4	0001953 // negative regulation of cell-matrix adhesion // not recorded///0002244 // hemato
NM_001281852///NM_	0.000	1.799	***S100a9***	S100 calcium binding protein A9 (calgranulin B)	0002376 // immune system process // inferred from electronic annotation///0002523 // leuk
NM_019507	0.001	1.807	***Tbx21***	T-box 21	0006351 // transcription, DNA-templated // inferred from electronic annotation///0006355 //
NM_011397///XM_011	0.001	1.819	***Slc23a1***	solute carrier family 23 (nucleobase transporters), member 1	0006810 // transport // inferred from electronic annotation///0006811 // ion transport // infe
NM_001146275///NM_	0.000	1.834	***Iigp1***	interferon inducible GTPase 1	0002376 // immune system process // inferred from electronic annotation///0008152 // meta
NM_018738	0.000	1.837	***Igtp***	interferon gamma induced GTPase	0035458 // cellular response to interferon-beta // inferred from direct assay
NM_021475	0.002	1.838	***Adamdec1***	ADAM-like, decysin 1	0006508 // proteolysis // inferred from electronic annotation
NM_007769///XM_006	0.002	1.841	***Dmbt1***	deleted in malignant brain tumors 1	0001824 // blastocyst development // inferred from mutant phenotype///0001833 // inner ce
NM_001302559///NM_	0.002	1.848	***Slfn3///Slfn4***	schlafen 3///schlafen 4	0008285 // negative regulation of cell proliferation // inferred from direct assay
NM_145209///XM_006	0.000	1.851	***Oasl1***	2'-5' oligoadenylate synthetase-like 1	0002376 // immune system process // inferred from electronic annotation///0006955 // imm
NM_001081746///XM_	0.001	1.853	***Csprs***	component of Sp100-rs///predicted pseudogene 15433///predict	0006338 // chromatin remodeling // ---///0006355 // regulation of transcription, DNA-templa
NM_001291066///NM_	0.049	1.858	***Adam8***	a disintegrin and metallopeptidase domain 8	0000902 // cell morphogenesis // inferred from mutant phenotype///0001525 // angiogenesi
NM_001289492///NM_	0.000	1.866	***Gbp3***	guanylate binding protein 3	0008152 // metabolic process // inferred from electronic annotation///0035458 // cellular res
NM_009978///XM_006	0.001	1.876	***Cst8***	cystatin 8 (cystatin-related epididymal spermatogenic)	0010466 // negative regulation of peptidase activity // inferred from electronic annotation///
NM_001139519///NM_	0.000	1.876	***Zbp1***	Z-DNA binding protein 1	0002376 // immune system process // inferred from electronic annotation///0008152 // meta
NM_008329///XM_006	0.009	1.885	***Ifi204///Ifi205///Mnda///Mndal***	interferon activated gene 204///interferon activated gene 205///	0006351 // transcription, DNA-templated // inferred from electronic annotation///0006355 //
NM_153590///XM_006	0.000	1.887	***Klre1***	killer cell lectin-like receptor family E member 1	0002223 // stimulatory C-type lectin receptor signaling pathway // inferred from direct assay/
NM_001110323///NM_	0.049	1.896	***Klra7///LOC100862438***	killer cell lectin-like receptor, subfamily A, member 7///killer cell	0007155 // cell adhesion // inferred from electronic annotation
NM_008489	0.001	1.899	***Lbp***	lipopolysaccharide binding protein	0001889 // liver development // inferred from electronic annotation///0002232 // leukocyte
NM_001142706///NM_	0.000	1.899	***Cfb***	complement factor B	0002376 // immune system process // inferred from electronic annotation///0006508 // prot
NM_008611	0.001	1.906	***Mmp8***	matrix metallopeptidase 8	0006508 // proteolysis // not recorded///0030574 // collagen catabolic process // inferred fro
NM_139198///XM_011	0.000	1.932	***Plac8***	placenta-specific 8	0008284 // positive regulation of cell proliferation // inferred from direct assay///0009409 //
NM_178594	0.013	1.934	***Vtcn1***	V-set domain containing T cell activation inhibitor 1	0001562 // response to protozoan // inferred from mutant phenotype///0002250 // adaptive
NM_013650	0.000	1.942	***S100a8***	S100 calcium binding protein A8 (calgranulin A)	0002376 // immune system process // inferred from electronic annotation///0002523 // leuk
NM_001136068///NM_	0.026	1.945	***Klrc1***	killer cell lectin-like receptor subfamily C, member 1	0023024 // MHC class I protein complex binding // not recorded
NM_001270495///NM_	0.009	1.952	***Tmem254a///Tmem254b///Tmem254***	transmembrane protein 254a///transmembrane protein 254b///transmembrane protein 254c	
NM_001198560///NM_	0.006	1.955	***H2-D4///H2-Q7///H2-Q9***	histocompatibility 2, D region locus 4///histocompatibility 2, Q re	0001916 // positive regulation of T cell mediated cytotoxicity // inferred from direct assay///0
NM_011909	0.000	1.958	***Usp18***	ubiquitin specific peptidase 18	0006508 // proteolysis // inferred from electronic annotation///0006511 // ubiquitin-depend

RefSeq Transcript ID	p	FD Air	*Gene Symbol*	Gene Title	Gene Ontology
NM_001037713///NM_	0.000	1.967	***Xaf1***	XIAP associated factor 1	0006915 // apoptotic process // inferred from electronic annotation///0031333 // negative re
NM_001270495///NM_	0.009	1.985	***Tmem254a///Tmem254b///Tmem254***	transmembrane protein 254a///transmembrane protein 254b///transmembrane protein 254c	
NM_026630	0.014	1.990	***Gtsf1l***	gametocyte specific factor 1-like	0046872 // metal ion binding // inferred from electronic annotation
NM_001281845///NM_	0.040	1.994	***Anxa8***	annexin A8	0007596 // blood coagulation // inferred from electronic annotation///0007599 // hemostasis
NM_010501///XM_01	0.000	2.012	***Ifit3***	interferon-induced protein with tetratricopeptide repeats 3	0002376 // immune system process // inferred from electronic annotation///0008285 // nega
NM_021274	0.016	2.018	***Cxcl10***	chemokine (C-X-C motif) ligand 10	0002690 // positive regulation of leukocyte chemotaxis // not recorded///0006935 // chemot
NM_009363	0.015	2.022	***Tff2***	trefoil factor 2 (spasmolytic protein 1)	0008284 // positive regulation of cell proliferation // inferred from genetic interaction///0008
NM_013653	0.001	2.029	***Ccl5***	chemokine (C-C motif) ligand 5	0000165 // MAPK cascade // not recorded///0002230 // positive regulation of defense respor
NM_011150	0.000	2.055	***Lgals3bp***	lectin, galactoside-binding, soluble, 3 binding protein	0006898 // receptor-mediated endocytosis // inferred from sequence or structural similarity/
NM_001081746///NM_	0.002	2.059	***Csprs***	component of Sp100-rs///predicted pseudogene 15433///predict	0006338 // chromatin remodeling // ---///0006355 // regulation of transcription, DNA-templa
NM_001033632	0.012	2.059	***Ifitm6***	interferon induced transmembrane protein 6	0009607 // response to biotic stimulus // inferred from electronic annotation
NM_016675///XM_006	0.001	2.069	***Cldn2***	claudin 2	0016338 // calcium-independent cell-cell adhesion via plasma membrane cell-adhesion molec
NM_001145164///NM_	0.000	2.094	***Tgtp1///Tgtp2***	T cell specific GTPase 1///T cell specific GTPase 2	0006955 // immune response // non-traceable author statement///0009615 // response to vi
NM_010172	0.005	2.098	***F7***	coagulation factor VII	0002690 // positive regulation of leukocyte chemotaxis // not recorded///0006508 // proteol
NM_020002///XM_006	0.048	2.103	***Rec8***	REC8 meiotic recombination protein	0000724 // double-strand break repair via homologous recombination // inferred from geneti
NM_001161621///NM_	0.043	2.105	***Aoc1***	amine oxidase, copper-containing 1	0006812 // cation transport // ---///0009308 // amine metabolic process // inferred from elec
NM_001204910///XM_	0.047	2.111	***AI607873***	expressed sequence AI607873	0000122 // negative regulation of transcription from RNA polymerase II promoter // ---
NM_134157	0.022	2.150	***Atp6v1b1***	ATPase, H+ transporting, lysosomal V1 subunit B1	0001503 // ossification // not recorded///0001503 // ossification // inferred from sequence o
NM_029796	0.002	2.192	***Lrg1***	leucine-rich alpha-2-glycoprotein 1	0001938 // positive regulation of endothelial cell proliferation // inferred from mutant pheno
NM_001277944///NM_	0.003	2.205	***Apoc2///Apoc4-apoc2***	apolipoprotein C-II///Apoc4-Apoc2 readthrough	0006629 // lipid metabolic process // inferred from electronic annotation///0006810 // trans
NM_001101620///NM_	0.011	2.209	***Klra21///Klra3///Klra8///Klra9***	killer cell lectin-like receptor subfamily A, member 21///killer cell	0007155 // cell adhesion // inferred from electronic annotation///0009615 // response to viru
NM_023386///XM_006	0.000	2.221	***Rtp4***	receptor transporter protein 4	0001580 // detection of chemical stimulus involved in sensory perception of bitter taste // no
NM_001304551///NM_	0.029	2.234	***Stil***	Scl/Tal1 interrupting locus	0000578 // embryonic axis specification // inferred from mutant phenotype///0001701 // in u
NM_130878	0.000	2.236	***Cdhr1***	cadherin-related family member 1	0007155 // cell adhesion // inferred from electronic annotation///0007156 // homophilic cell
NM_001033207///XM_	0.000	2.253	***Nlrc5***	NLR family, CARD domain containing 5	0002376 // immune system process // inferred from electronic annotation///0032088 // nega
NM_145600	0.047	2.267	***Zfp330***	zinc finger protein 330	0008270 // zinc ion binding // inferred from electronic annotation///0046872 // metal ion bir
NM_001033384///NM_	0.003	2.271	***Pydc4***	pyrin domain containing 4	0000122 // negative regulation of transcription from RNA polymerase II promoter // ---
NM_001289604///NM_	0.004	2.279	***Klra3///Klra9***	killer cell lectin-like receptor, subfamily A, member 3///killer cell	0007155 // cell adhesion // inferred from electronic annotation
NM_010846///NR_003	0.000	2.300	***Mx1***	MX dynamin-like GTPase 1	0002376 // immune system process // inferred from electronic annotation///0008152 // meta
NM_029499///XM_006	0.001	2.305	***Ms4a4c***	membrane-spanning 4-domains, subfamily A, member 4C	0005887 // integral component of plasma membrane // inferred from electronic annotation//
NM_001033409///XM_	0.018	2.307	***Lgr6***	leucine-rich repeat-containing G protein-coupled receptor 6	0007165 // signal transduction // inferred from electronic annotation///0007186 // G-protein
NM_010362	0.001	2.316	***Gsto1***	glutathione S-transferase omega 1	0006749 // glutathione metabolic process // not recorded///0008152 // metabolic process //
NM_008331	0.000	2.325	***Ifit1***	interferon-induced protein with tetratricopeptide repeats 1	0002376 // immune system process // inferred from electronic annotation///0009615 // resp
NM_146050///XM_006	0.040	2.337	***Oit1***	oncoprotein induced transcript 1	0046676 // negative regulation of insulin secretion // not recorded///0046676 // negative reg
NM_001136059///NM_	0.009	2.357	***Cyp1a1***	cytochrome P450, family 1, subfamily a, polypeptide 1	0001666 // response to hypoxia // inferred from electronic annotation///0001889 // liver dev
NM_145838///XM_006	0.002	2.396	***St8sia6***	ST8 alpha-N-acetyl-neuraminide alpha-2,8-sialyltransferase 6	0001574 // ganglioside biosynthetic process // inferred from direct assay///0006486 // protei
NM_026671///XM_006	0.010	2.427	***Lypd2***	Ly6/Plaur domain containing 2	0005576 // extracellular region // ---///0005886 // plasma membrane // inferred from electro
NM_175026	0.004	2.427	***Pyhin1***	pyrin and HIN domain family, member 1	0000122 // negative regulation of transcription from RNA polymerase II promoter // ---///003
NM_153576	0.001	2.430	***Cxcl17***	chemokine (C-X-C motif) ligand 17	0001525 // angiogenesis // inferred from electronic annotation///0006935 // chemotaxis // ir
NM_013542	0.000	2.441	***Gzmb***	granzyme B	0001913 // T cell mediated cytotoxicity // inferred from mutant phenotype///0006508 // prot
XM_011238903///XM_	0.041	2.560	***Gm37416///Gm39743///Gm40611///***	predicted gene, 37416///predicted gene, 39743///predicted gene	0006278 // RNA-dependent DNA replication // inferred from electronic annotation///000815
NM_015783///XR_168	0.000	2.611	***Gm9706///Isg15***	predicted gene 9706///ISG15 ubiquitin-like modifier	0019941 // modification-dependent protein catabolic process // inferred from direct assay///
NM_027399	0.007	2.612	***Steap1***	six transmembrane epithelial antigen of the prostate 1	0006810 // transport // inferred from electronic annotation///0006811 // ion transport // infe
NM_011407	0.008	2.635	***Slfn1***	schlafen 1	0000122 // negative regulation of transcription from RNA polymerase II promoter // inferred
NM_145227///XM_006	0.000	2.656	***Oas2***	2'-5' oligoadenylate synthetase 2	0002376 // immune system process // inferred from electronic annotation///0006164 // purir
NM_010370	0.000	2.669	***Gzma***	granzyme A	0006508 // proteolysis // inferred from electronic annotation///0006915 // apoptotic process
NM_001281830///NM_	0.030	2.683	***Ifi27l2a***	interferon, alpha-inducible protein 27 like 2A	0007568 // aging // inferred from direct assay///0009615 // response to virus // inferred from
NM_015783///XR_168	0.000	2.852	***Gm9706///Isg15***	predicted gene 9706///ISG15 ubiquitin-like modifier	0019941 // modification-dependent protein catabolic process // inferred from direct assay///
NM_001001179	0.001	2.933	***BC048546***	cDNA sequence BC048546	0010466 // negative regulation of peptidase activity // inferred from electronic annotation///
NM_001162883///NM_	0.000	2.948	***Apol9a///Apol9b***	apolipoprotein L 9a///apolipoprotein L 9b	0006869 // lipid transport // inferred from electronic annotation///0042157 // lipoprotein me
NM_001081180///XM_	0.020	3.086	***Spink5***	serine peptidase inhibitor, Kazal type 5	0002787 // negative regulation of antibacterial peptide production // inferred from mutant pl
NM_145423	0.043	3.187	***Slc5a8***	solute carrier family 5 (iodide transporter), member 8	0006810 // transport // inferred from electronic annotation///0006811 // ion transport // infe
NM_016771	0.037	3.249	***Sult1d1***	sulfotransferase family 1D, member 1	0000103 // sulfate assimilation // inferred from direct assay///0006584 // catecholamine met
NM_018751	0.000	3.269	***Sult1c1***	sulfotransferase family, cytosolic, 1C, member 1	0006790 // sulfur compound metabolic process // inferred from direct assay

RefSeq Transcript ID	p	FD Air	*Gene Symbol*	Gene Title	Gene Ontology
NM_001252600///NM_	0.000	3.343	***Irf7***	interferon regulatory factor 7	0002376 // immune system process // inferred from electronic annotation///0002819 // regu
NM_011854///XM_006	0.000	3.351	***Oasl2***	2'-5' oligoadenylate synthetase-like 2	0002376 // immune system process // inferred from electronic annotation///0006164 // purir
NM_010243	0.007	3.358	***Fut9***	fucosyltransferase 9	0006486 // protein glycosylation // inferred from direct assay///0007399 // nervous system d
NM_177406	0.042	3.418	***Cyp4a12a***	cytochrome P450, family 4, subfamily a, polypeptide 12a	0006631 // fatty acid metabolic process // inferred from sequence or structural similarity///0(
NM_028801///XM_006	0.000	3.518	***Muc5b***	mucin 5, subtype B, tracheobronchial	0042742 // defense response to bacterium // inferred from mutant phenotype///0043030 //
NM_021384///XM_011	0.000	3.553	***Rsad2***	radical S-adenosyl methionine domain containing 2	0001503 // ossification // not recorded///0002376 // immune system process // inferred fron
NM_145211///XM_006	0.000	3.660	***Oas1a***	2'-5' oligoadenylate synthetase 1A	0002376 // immune system process // inferred from electronic annotation///0006164 // purii
NM_027416	0.044	3.685	***Calml3***	calmodulin-like 3	0005509 // calcium ion binding // inferred from electronic annotation///0005515 // protein b
NM_054037///NM_170	0.044	3.847	***Scgb3a1***	secretoglobin, family 3A, member 1	1901741 // positive regulation of myoblast fusion // inferred from mutant phenotype
NM_133238	0.009	4.009	***Cd209a***	CD209a antigen	0006897 // endocytosis // inferred from electronic annotation///0010468 // regulation of ger
NM_001033767///XM_	0.000	4.068	***Gm4951***	predicted gene 4951	0035458 // cellular response to interferon-beta // inferred from direct assay
XM_011238903///XM_	0.022	4.822	***Gm37416///Gm39743///Gm40611///***	predicted gene, 37416///predicted gene, 39743///predicted gene	0006278 // RNA-dependent DNA replication // inferred from electronic annotation///000815
NM_133871///XM_006	0.000	5.044	***Ifi44***	interferon-induced protein 44	0005737 // cytoplasm // inferred from electronic annotation
NM_009244///XM_006	0.011	5.159	***Serpina1b***	serine (or cysteine) preptidase inhibitor, clade A, member 1B	0001701 // in utero embryonic development // inferred from mutant phenotype///0006487 /
NM_011783	0.002	5.455	***Agr2***	anterior gradient 2	0010628 // positive regulation of gene expression // not recorded///0010811 // positive regu
NM_017474///XM_006	0.010	5.554	***Clca1///Clca3a1///Clca2///Clca3a2***	chloride channel accessory 1///chloride channel accessory 3A1///	0006508 // proteolysis // inferred from electronic annotation///0006810 // transport // inferr
NM_001302559///NM_	0.000	6.148	***Slfn4///Slfn3***	schlafen 4///schlafen 3	0008285 // negative regulation of cell proliferation // inferred from direct assay
NM_011315	0.000	6.202	***Saa3***	serum amyloid A 3	0006953 // acute-phase response // inferred from electronic annotation///0007252 // I-kappa
XM_011238903///XM_	0.046	6.504	***Gm3579///Gm40514///Gm40814///G***	predicted gene 3579///predicted gene, 40514///predicted gene,	0015074 // DNA integration // inferred from electronic annotation///0090502 // RNA phosph
NM_031367	0.000	8.278	***Ifi44l***	interferon-induced protein 44 like	0006955 // immune response // inferred from direct assay
NM_001012392///NM_	0.016	8.872	***Bpifb1***	BPI fold containing family B, member 1	0002227 // innate immune response in mucosa // not recorded///0002376 // immune system
NM_025989///XM_006	0.000	10.017	***Gp2***	glycoprotein 2 (zymogen granule membrane)	0002412 // antigen transcytosis by M cells in mucosal-associated lymphoid tissue // inferred f
NM_011260	0.00421	25.756	***Reg3g***	regenerating islet-derived 3 gamma	0002755 // MyD88-dependent toll-like receptor signaling pathway // inferred from mutant pł

Table S6. Nfkb1-dependently modulated lung genes after ozone (0.3 ppm, 48 hr) exposure.

Slected genes varied >1.5-fold between Nfkb1-WT and Nfkb1-KO mice (positive-higher in KO-O3, negative-lower in KO-O3, compared to WT-O3).

Affymetrix_GeneChip_Mouse430_2. 2-Way ANOVA (p<0.05). Order by fold difference (FD) between WT and KO. FC=fold change by O3 in each genotype.

RefSeq Transcript ID	p (Genotype) (	p (Genotype-Exposure) (2	p (Exposure) (	FC WT Air:O3	FC KO Air:O3	FD Air WT:KO	FD O3 WT:KO	Gene Symbol	Gene Title	Gene Ontology Biological Process
NM_028216	2.08E-04	0.002125263	4.99E-04	8.88	1.24	-1.51	**-10.87**	***Psca***	prostate stem cell antigen	
NM_007994	0.003934348	0.012876396	0.004998094	5.31	1.19	-1.27	**-5.70**	***Fbp2***	fructose bisphosphatase 2	0005975 // carbohydrate metabolic process // inferred from electronic annotation///0006094 // gluconeogenesis
NM_001131054///NM_013917///XM_006533515///XM_006533516				-2.12	-1.70	-3.51	**-2.81**	***Pttg1***	pituitary tumor-transforming gene 1	0001558 // regulation of cell growth // not recorded///0006281 // DNA repair // inferred from electronic annotati
NM_001081328				3.07	2.21	-1.95	**-2.71**	***Chsy3***	chondroitin sulfate synthase 3	0008152 // metabolic process // inferred from electronic annotation
NM_025748///XM_011243200				1.98	1.56	-2.07	**-2.63**	***Adat2***	adenosine deaminase, tRNA-specific 2	0002100 // tRNA wobble adenosine to inosine editing // inferred from electronic annotation///0006400 // tRNA m
NM_009264	0.31510517	0.002185997	7.96E-07	21.03	5.00	1.67	**-2.52**	***Sprr1a***	small proline-rich protein 1A	0018149 // peptide cross-linking // not recorded///0030216 // keratinocyte differentiation // not recorded///0031
NM_030069///XM_	1.26E-04	0.005971318	0.008376999	1.83	-1.09	-1.26	**-2.51**	***Nxpe2***	neurexophilin and PC-esterase domain family, member 2	
NM_172301///XM_	0.27635863	0.026451888	0.03953187	3.23	-1.15	1.52	**-2.44**	***Ccnb1***	cyclin B1	0000278 // mitotic cell cycle // inferred from electronic annotation///0001556 // oocyte maturation // not recorde
NM_001177544///N	0.06522717	0.04535243	0.01881624	2.88	1.23	-1.03	**-2.42**	***Hist1h2ao///Hist1h2ap***	histone cluster 1, H2ao///histone cluster 1, H2ap	0006325 // chromatin organization // not recorded///0006342 // chromatin silencing // not recorded///0008285
NM_010295///XM_	1.68E-04	0.003448708	0.004016006	1.97	1.07	-1.30	**-2.38**	***Gclc***	glutamate-cysteine ligase, catalytic subunit	0006534 // cysteine metabolic process // not recorded///0006536 // glutamate metabolic process // not recorded
NM_016974///XM_	0.6920544	0.00983078	0.032890383	1.20	-3.34	1.77	**-2.27**	***Dbp***	D site albumin promoter binding protein	0001889 // liver development // inferred from electronic annotation///0006351 // transcription, DNA-templated
NM_007659	0.31935605	0.042050056	0.056642193	2.77	-1.04	1.32	**-2.18**	***Cdk1***	cyclin-dependent kinase 1	0006461 // protein complex assembly // not recorded///0006468 // protein phosphorylation // inferred from direc
NM_011332///XM_	0.025220744	0.015234006	1.71E-05	4.96	2.29	1.01	**-2.16**	***Ccl17***	chemokine (C-C motif) ligand 17	0002548 // monocyte chemotaxis // ---///0006935 // chemotaxis // traceable author statement///0006954 // infla
NM_028039	0.3708932	0.043012165	0.06698847	2.62	-1.07	1.35	**-2.07**	***Esco2***	establishment of cohesion 1 homolog 2 (S. cerevisiae)	0002244 // hematopoietic progenitor cell differentiation // inferred from genetic interaction///0006275 // regulat
NM_026785	0.10046281	0.006311119	0.1293555	1.96	-1.23	1.18	**-2.04**	***Ube2c***	ubiquitin-conjugating enzyme E2C	0000209 // protein polyubiquitination // not recorded///0006511 // ubiquitin-dependent protein catabolic process
NM_019810	4.43E-04	0.003642625	1.30E-04	2.13	1.15	-1.09	**-2.02**	***Slc5a1***	solute carrier family 5 (sodium/glucose cotransporter), member 1	0001656 // metanephros development // inferred from mutant phenotype///0006810 // transport // inferred fron
NM_009364///XM_006505043///XM_006505044///XM_011241055///XM_011				2.14	2.49	-2.34	**-2.02**	***Tfpi2***	tissue factor pathway inhibitor 2	0007596 // blood coagulation // inferred from electronic annotation///0007599 // hemostasis // inferred from ele
NM_026560	0.170261	0.041151717	0.01817487	2.44	1.01	1.22	**-1.98**	***Cdca8***	cell division cycle associated 8	0000070 // mitotic sister chromatid segregation // ---///0007049 // cell cycle // inferred from electronic annotatio
NM_010809				2.06	1.81	-1.73	**-1.97**	***Mmp3***	matrix metallopeptidase 3	0006508 // proteolysis // inferred from mutant phenotype///0006508 // proteolysis // not recorded///0010727 //
NM_010104	0.007884334	0.020289894	0.14191255	1.62	-1.05	-1.16	**-1.96**	***Edn1***	endothelin 1	0000122 // negative regulation of transcription from RNA polymerase II promoter // not recorded///0001501 // sk
NM_009137	0.002550701	0.022045342	0.032397464	1.60	-1.07	-1.14	**-1.96**	***Ccl22***	chemokine (C-C motif) ligand 22	0002548 // monocyte chemotaxis // ---///0006935 // chemotaxis // inferred from electronic annotation///000695
NM_011369	0.07999367	0.01631937	0.001532258	2.69	1.18	1.16	**-1.95**	***Shcbp1***	Shc SH2-domain binding protein 1	0008543 // fibroblast growth factor receptor signaling pathway // inferred from mutant phenotype///2000177 // r
NM_172301///XM_	0.22189197	0.0355218	0.03068305	2.35	-1.07	1.30	**-1.95**	***Ccnb1///Gm5593***	cyclin B1///predicted gene 5593	0000278 // mitotic cell cycle // inferred from electronic annotation///0001556 // oocyte maturation // not recorde
NM_008689///XM_006501106///XM_006501107				-1.08	-1.05	-2.01	**-1.94**	***Nfkb1***	nuclear factor of kappa light polypeptide gene enhancer in B cells 1, p105	0000122 // negative regulation of transcription from RNA polymerase II promoter // not recorded///0001818 // ne
NM_177351	3.61E-04	0.03394541	0.7943103	1.21	-1.24	-1.29	**-1.94**	***Hykk***	hydroxylysine kinase 1	0016310 // phosphorylation // inferred from electronic annotation
NM_145434	0.14153165	0.012387881	0.001671302	-1.20	-2.64	1.14	**-1.94**	***Nr1d1***	nuclear receptor subfamily 1, group D, member 1	0000122 // negative regulation of transcription from RNA polymerase II promoter // not recorded///0005978 // gl
NM_009828	0.102943376	0.006373226	0.001945413	2.53	1.11	1.20	**-1.90**	***Ccna2***	cyclin A2	0000079 // regulation of cyclin-dependent protein serine/threonine kinase activity // inferred from electronic anno
NM_008021///NM_	0.08359548	0.013229165	0.04609279	1.93	-1.15	1.20	**-1.86**	***Foxm1***	forkhead box M1	0000086 // G2/M transition of mitotic cell cycle // not recorded///0000122 // negative regulation of transcription
NM_010766				2.15	2.98	-2.58	**-1.86**	***Marco***	macrophage receptor with collagenous structure	0002376 // immune system process // inferred from electronic annotation///0006897 // endocytosis // inferred fr
NM_001285997///N	0.11426556	0.011221865	0.005312019	2.29	1.02	1.21	**-1.85**	***Prc1***	protein regulator of cytokinesis 1	0000226 // microtubule cytoskeleton organization // inferred from electronic annotation///0000910 // cytokinesis
NM_001286404///N	0.01847303	0.028604146	0.014477238	1.79	1.02	-1.03	**-1.81**	***Ghrl***	ghrelin	0000187 // activation of MAPK activity // not recorded///0001696 // gastric acid secretion // not recorded///0001
NM_001243008///NM_001243009///NM_009935				-1.21	-1.48	-1.48	**-1.81**	***Col6a3***	collagen, type VI, alpha 3	0010951 // negative regulation of endopeptidase activity // inferred from electronic annotation
NM_010400				1.51	1.03	-1.23	**-1.80**	***H60a***	histocompatibility 60a	0001913 // T cell mediated cytotoxicity // ---///0002839 // positive regulation of immune response to tumor cell //
NM_011121	0.09216006	0.021945084	0.010812664	2.06	1.01	1.13	**-1.80**	***Plk1***	polo-like kinase 1	0000070 // mitotic sister chromatid segregation // not recorded///0000086 // G2/M transition of mitotic cell cycle
NM_011607///XM_006537774///XM_006537775///XM_006537776///XM_006				4.38	4.39	-1.79	**-1.79**	***Tnc***	tenascin C	0001649 // osteoblast differentiation // not recorded///0007155 // cell adhesion // inferred from electronic annot
NM_007499///XM_006509956///XM_011242385///XM_011242386///XR_870				1.38	1.12	-1.41	**-1.75**	***Atm***	ataxia telangiectasia mutated	0000077 // DNA damage checkpoint // inferred from mutant phenotype///0000723 // telomere maintenance // in
NM_010474	6.41E-04	0.002802173	0.011012673	1.54	1.03	-1.16	**-1.73**	***Hs3st1***	heparan sulfate (glucosamine) 3-O-sulfotransferase 1	0008152 // metabolic process // inferred from electronic annotation
NM_008120	6.36E-04	0.01632262	0.007604335	-1.02	-1.41	-1.25	**-1.73**	***Gja4***	gap junction protein, alpha 4	0001568 // blood vessel development // inferred from mutant phenotype///0003158 // endothelium development
NM_172505///NM_198652///NR_040291///XM_006529720///XM_006529721				1.49	1.06	-1.23	**-1.73**	***Hjurp***	Holliday junction recognition protein	0007049 // cell cycle // inferred from electronic annotation///0007059 // chromosome segregation // not recorde
NM_172947///XM_006533414				1.28	1.11	-1.50	**-1.72**	***Lsm12***	LSM12 homolog (S. cerevisiae)	
NM_001042652///N	0.22683874	0.021436276	0.021979421	2.02	-1.04	1.22	**-1.72**	***Nusap1***	nucleolar and spindle associated protein 1	0000070 // mitotic sister chromatid segregation // not recorded///0000226 // microtubule cytoskeleton organizat
NM_153127				-1.37	-1.30	-1.80	**-1.72**	***Mmrn2***	multimerin 2	0001525 // angiogenesis // inferred from electronic annotation///0030948 // negative regulation of vascular endo
NM_011496///XM_	0.123693265	0.01574747	0.003241606	2.20	1.06	1.22	**-1.71**	***Aurkb***	aurora kinase B	0000122 // negative regulation of transcription from RNA polymerase II promoter // not recorded///0002903 // ne
NM_178357				-1.12	-1.48	-1.28	**-1.69**	***Klf11***	Kruppel-like factor 11	0000083 // regulation of transcription involved in G1/S transition of mitotic cell cycle // not recorded///0000122 /
NM_010790///XM_	0.60653466	0.034828283	0.025739746	2.35	-1.05	1.48	**-1.68**	***Melk***	maternal embryonic leucine zipper kinase	0006468 // protein phosphorylation // inferred from electronic annotation///0006915 // apoptotic process // not
NM_021451				1.72	1.37	-1.33	**-1.67**	***Pmaip1***	phorbol-12-myristate-13-acetate-induced protein 1	0001836 // release of cytochrome c from mitochondria // inferred from direct assay///0001836 // release of cytoc
NM_011606				-2.05	-1.78	-1.92	**-1.67**	***Clec3b***	C-type lectin domain family 3, member b	0001501 // skeletal system development // inferred from mutant phenotype///0001503 // ossification // inferred
NM_029568///XM_006534422///XM_011249299				-1.15	-1.41	-1.36	**-1.66**	***Mfap4***	microfibrillar-associated protein 4	0007155 // cell adhesion // inferred from electronic annotation///0009650 // UV protection // not recorded///001
NM_021886///XM_006517676///XM_006517678///XM_011244667				1.66	1.06	-1.05	**-1.65**	***Cenph***	centromere protein H	0007059 // chromosome segregation // not recorded///0007067 // mitotic nuclear division // not recorded///005
NM_019568///XM_006517307///XM_006517308///XM_011244545				2.31	1.74	-1.24	**-1.65**	***Cxcl14***	chemokine (C-X-C motif) ligand 14	0006955 // immune response // inferred from electronic annotation///0045662 // negative regulation of myoblast
NM_009930				-1.37	-1.57	-1.42	**-1.63**	***Col3a1***	collagen, type III, alpha 1	0001501 // skeletal system development // inferred from electronic annotation///0001568 // blood vessel develop
NM_010406				-1.20	-1.37	-1.41	**-1.62**	***Hc***	hemolytic complement	0001701 // in utero embryonic development // inferred from genetic interaction///0002376 // immune system pro
NR_024067	0.001099549	0.034218304	0.004641574	1.51	1.18	-1.26	**-1.62**	***Snhg6***	small nucleolar RNA host gene 6	
NM_011584	0.023310691	0.003704031	0.08615867	1.18	-1.43	1.04	**-1.62**	***Nr1d2***	nuclear receptor subfamily 1, group D, member 2	0006351 // transcription, DNA-templated // inferred from electronic annotation///0006355 // regulation of transc
NM_013552///XM_	0.024337515	0.001527545	0.043383528	1.53	-1.23	1.17	**-1.61**	***Hmmr***	hyaluronan mediated motility receptor (RHAMM)	
NM_001204201///N	0.5873361	0.00583395	3.89E-06	5.64	2.60	1.35	**-1.61**	***Spp1***	secreted phosphoprotein 1	0001503 // ossification // inferred from electronic annotation///0001649 // osteoblast differentiation // not recor
NM_011446	0.04378882	0.013421392	0.13991171	1.14	-1.37	-1.02	**-1.60**	***Sox7***	SRY (sex determining region Y)-box 7	0001706 // endoderm formation // not recorded///0006351 // transcription, DNA-templated // inferred from elec
NM_001145830///NM_019677///XM_006498930				-1.18	-1.42	-1.33	**-1.60**	***Plcb1***	phospholipase C, beta 1	0000086 // G2/M transition of mitotic cell cycle // inferred from direct assay///0000086 // G2/M transition of mit
NM_009579///XM_	2.84E-04	0.022632362	0.44732496	1.22	-1.02	-1.28	**-1.60**	***Slc30a1***	solute carrier family 30 (zinc transporter), member 1	0001701 // in utero embryonic development // inferred from mutant phenotype///0006810 // transport // inferre
NM_001159299///N	0.22589071	0.04807575	0.10612984	1.72	1.02	1.06	**-1.59**	***Itih4***	inter alpha-trypsin inhibitor, heavy chain 4	0006953 // acute-phase response // inferred from electronic annotation///0010466 // negative regulation of pepti
NM_007742				-1.19	-1.62	-1.16	**-1.57**	***Col1a1***	collagen, type I, alpha 1	0001501 // skeletal system development // inferred from mutant phenotype///0001501 // skeletal system develop
NM_001160012///N	0.009046308	0.012056153	2.17E-05	2.17	1.38	1.00	**-1.57**	***Gjb3***	gap junction protein, beta 3	0001701 // in utero embryonic development // inferred from electronic annotation///0001890 // placenta develop
NM_011784				-2.31	-2.70	-1.34	**-1.56**	***Aplnr***	apelin receptor	0007165 // signal transduction // inferred from electronic annotation///0007186 // G-protein coupled receptor sig
NM_001304757///NM_007731///XM_006513170///XM_006513171///XM_006				1.52	1.32	-1.36	**-1.56**	***Col13a1***	collagen, type XIII, alpha 1	0001503 // ossification // inferred from electronic annotation///0001763 // morphogenesis of a branching structu
NM_144557///XM_	0.3194705	0.049879316	0.002016708	2.40	1.43	1.08	**-1.55**	***Myrip***	myosin VIIA and Rab interacting protein	0006886 // intracellular protein transport // inferred from electronic annotation///0030050 // vesicle transport al
NM_008127///XM_	0.020179005	0.002136478	9.87E-06	2.31	1.30	1.14	**-1.55**	***Gjb4***	gap junction protein, beta 4	0007154 // cell communication // inferred from electronic annotation///0007608 // sensory perception of smell //
NM_019438///XM_011240756				1.69	1.13	-1.03	**-1.55**	***Ncapg***	non-SMC condensin I complex, subunit G	0007076 // mitotic chromosome condensation // not recorded
NM_011823///XM_006527624///XM_006527625///XM_006527626				-1.32	-1.35	-1.51	**-1.55**	***Gpr34***	G protein-coupled receptor 34	0007165 // signal transduction // inferred from electronic annotation///0007186 // G-protein coupled receptor sig
NM_001033217///X	0.003885978	0.001949741	0.001605649	1.01	-1.45	-1.05	**-1.54**	***Prickle1***	prickle homolog 1 (Drosophila)	0001843 // neural tube closure // not recorded///0006606 // protein import into nucleus // not recorded///00313
NM_001164493				1.90	1.57	-1.26	**-1.53**	***Klhl29***	kelch-like 29	0016567 // protein ubiquitination // not recorded
NM_130448///XM_	0.002940856	0.026478754	0.8978053	1.17	-1.12	-1.16	**-1.52**	***Pcdh18***	protocadherin 18	0007155 // cell adhesion // inferred from electronic annotation///0007156 // homophilic cell adhesion via plasma
NM_009135///XM_	3.65E-04	0.013911554	1.12E-04	-1.20	-1.51	-1.21	**-1.52**	***Scn7a***	sodium channel, voltage-gated, type VII, alpha	0006810 // transport // inferred from electronic annotation///0006811 // ion transport // inferred from electronic
NM_001081363///X	0.2492267	0.002802824	0.745116	1.37	-1.43	1.29	**-1.52**	***Cenpf***	centromere protein F	0000278 // mitotic cell cycle // not recorded///0002724 // regulation of T cell cytokine production // inferred from
NM_010615	0.3561519	0.019100692	0.04531508	1.72	-1.14	1.29	**-1.51**	***Kif11***	kinesin family member 11	0007018 // microtubule-based movement // not recorded///0007049 // cell cycle // inferred from electronic anno
NM_001302094///NM_001302095///NM_008115///XM_011244958				-1.25	-1.32	-1.44	**-1.51**	***Gfra2***	glial cell line derived neurotrophic factor family receptor alpha 2	0007169 // transmembrane receptor protein tyrosine kinase signaling pathway // inferred from genetic interactio
NM_026254///XM_006522484///XM_006522485///XM_006522486///XM_006				1.35	1.24	-1.38	**-1.51**	***Tbc1d23***	TBC1 domain family, member 23	0032680 // regulation of tumor necrosis factor production // inferred from mutant phenotype///0032755 // positiv
NM_001316749///NM_027526///XM_006506595///XM_006506596///XM_006				-1.01	-1.30	-1.17	**-1.50**	***Rasgef1a***	RasGEF domain family, member 1A	0007264 // small GTPase mediated signal transduction // inferred from electronic annotation///0016477 // cell mi
NM_001165939///NM_001165940///NM_009432///XM_006501299///XM_006				2.53	2.59	-1.53	**-1.50**	***Tshb***	thyroid stimulating hormone, beta subunit	0033189 // response to vitamin A // inferred from electronic annotation///0043627 // response to estrogen // infe
NM_010516				2.16	2.35	-1.62	**-1.49**	***Cyr61***	cysteine rich protein 61	0001558 // regulation of cell growth // inferred from electronic annotation///0001649 // osteoblast differentiatio
NM_001308535///NM_013563				-1.44	-1.40	-1.54	**-1.49**	***Il2rg***	interleukin 2 receptor, gamma chain	0010468 // regulation of gene expression // inferred from mutant phenotype///0010628 // positive regulation of g
NM_013462///XM_006508987///XM_006508988///XM_011242116///XM_011				-1.85	-2.14	-1.28	**-1.48**	***Adrb3***	adrenergic receptor, beta 3	0002024 // diet induced thermogenesis // inferred from genetic interaction///0002025 // vasodilation by norepine
NM_183312///NM_201639///NM_207663				-1.47	-1.52	-1.44	**-1.48**	***Synm***	synemin, intermediate filament protein	0045104 // intermediate filament cytoskeleton organization // inferred from electronic annotation
NM_008568///XM_	0.11804359	0.039903216	0.051822733	1.56	1.07	-1.02	**-1.48**	***Mcm7***	minichromosome maintenance deficient 7 (S. cerevisiae)	0006260 // DNA replication // inferred from electronic annotation///0006268 // DNA unwinding involved in DNA r
NM_010721	0.042766806	0.002243199	0.02098873	1.52	-1.01	1.04	**-1.47**	***Lmnb1***	lamin B1	0031662 // positive regulation of cyclin-dependent protein serine/threonine kinase activity involved in G2/M trans
NM_023320///XM_006501930///XM_011240206				-1.15	-1.14	-1.48	**-1.47**	***Plekho1***	pleckstrin homology domain containing, family O member 1	0007520 // myoblast fusion // inferred from direct assay///0007520 // myoblast fusion // inferred from mutant ph
NM_001289780///NM_001289781///NM_178403///XM_011249796///XR_880				1.28	1.32	-1.51	**-1.47**	***Pus7***	pseudouridylate synthase 7 homolog (S. cerevisiae)	0001522 // pseudouridine synthesis // inferred from electronic annotation///0001522 // pseudouridine synthesis
NM_010590	0.00398895	0.0230666	2.46E-04	1.70	1.33	-1.16	**-1.47**	***Ajuba***	ajuba LIM protein	0000122 // negative regulation of transcription from RNA polymerase II promoter // inferred from mutant phenoty
NM_013811///XM_006523588///XM_006523590///XM_006523591///XM_006				-1.55	-1.67	-1.36	**-1.47**	***Dnah8***	dynein, axonemal, heavy chain 8	0007018 // microtubule-based movement // inferred from electronic annotation///0008152 // metabolic process
NM_026793	0.043098956	0.014344383	5.88E-04	-1.16	-1.66	-1.02	**-1.47**	***Myct1***	myc target 1	
NM_026014	0.21927778	0.047128662	0.0602955	1.58	1.04	1.04	**-1.46**	***Cdt1***	chromatin licensing and DNA replication factor 1	0000076 // DNA replication checkpoint // inferred from direct assay///0000076 // DNA replication checkpoint // n
NM_001111051///NM_001111052///NM_001111053///NM_001195538///NM				1.35	1.56	-1.68	**-1.46**	***Dclk1***	doublecortin-like kinase 1	0001764 // neuron migration // inferred from genetic interaction///0006468 // protein phosphorylation // inferre

RefSeq Transcript ID	p (Genotype) (	p (Genotype-Exposure) (2	p (Exposure) (	FC WT Air:O3	FC KO Air:O3	FD Air WT:KO	FD O3 WT:KO	Gene Symbol	Gene Title	Gene Ontology Biological Process
NM_010225	0.007460019	0.014400821	0.22995287	1.10	-1.21	-1.10	**-1.46**	***Foxf2***	forkhead box F2	0006351 // transcription, DNA-templated // inferred from electronic annotation///0006355 // regulation of transc
NM_009610	0.4044168	0.009947431	0.023610901	1.10	-2.31	1.75	**-1.46**	***Actg2***	actin, gamma 2, smooth muscle, enteric	0010628 // positive regulation of gene expression // inferred from sequence or structural similarity///0090131 //
NM_001159564///NM_021359///XM_006498808///XM_006498809///XM_006				1.64	1.64	-1.45	**-1.45**	***Itgb6***	integrin beta 6	0006954 // inflammatory response // inferred from mutant phenotype///0007155 // cell adhesion // inferred from
NM_001004140///X	0.31412748	0.039913304	0.09432041	1.53	-1.13	1.19	**-1.45**	***Ckap2***	cytoskeleton associated protein 2	0000281 // mitotic cytokinesis // not recorded///0006915 // apoptotic process // inferred from electronic annotat
NM_001083917///NM_001286614///NM_153522///NM_178227///XM_00651				-1.86	-1.58	-1.71	**-1.45**	***Scn3b***	sodium channel, voltage-gated, type III, beta	0006810 // transport // inferred from electronic annotation///0006811 // ion transport // inferred from electronic
NM_133774///XM_006525694///XM_011246851				1.29	1.14	-1.28	**-1.45**	***Stard4***	StAR-related lipid transfer (START) domain containing 4	0006810 // transport // inferred from electronic annotation///0006869 // lipid transport // inferred from electroni
NM_001025779///N	0.8211958	0.034671698	1.82E-04	4.13	1.73	1.65	**-1.45**	***Cdc6***	cell division cycle 6	0006260 // DNA replication // inferred from electronic annotation///0006270 // DNA replication initiation // inferr
NM_001308327///NM_001308329///NM_008813///XM_006512597///XM_006				1.41	1.15	-1.18	**-1.45**	***Enpp1***	ectonucleotide pyrophosphatase/phosphodiesterase 1	0006091 // generation of precursor metabolites and energy // not recorded///0006796 // phosphate-containing co
NM_008842	0.003017425	0.03932411	0.004323326	1.09	1.54	1.02	**1.45**	***Pim1***	proviral integration site 1	0006468 // protein phosphorylation // not recorded///0007346 // regulation of mitotic cell cycle // not recorded//
NM_134117///XM_011246234				-1.44	-1.06	1.07	**1.45**	***Pkdcc***	protein kinase domain containing, cytoplasmic	0001501 // skeletal system development // inferred from mutant phenotype///0001503 // ossification // inferred
NM_178911///XM_011243975				-1.25	-1.11	1.29	**1.45**	***Pld4***	phospholipase D family, member 4	0002244 // hematopoietic progenitor cell differentiation // inferred from mutant phenotype///0006629 // lipid me
NM_001033308///XM_011250230				-1.60	-1.38	1.26	**1.46**	***Themis2***	thymocyte selection associated family member 2	0002376 // immune system process // inferred from electronic annotation///0006954 // inflammatory response //
NM_011756				1.21	1.73	1.02	**1.46**	***Zfp36***	zinc finger protein 36	0000122 // negative regulation of transcription from RNA polymerase II promoter // inferred from mutant phenoty
NM_029084///XM_	0.22718892	0.00252347	0.4994762	-1.32	1.30	-1.18	**1.46**	***Slamf8***	SLAM family member 8	0033860 // regulation of NAD(P)H oxidase activity // inferred from mutant phenotype///0035690 // cellular respor
NM_023738	2.03E-06	0.027224055	1.26E-04	-1.28	-1.03	1.18	**1.47**	***Uba7***	ubiquitin-like modifier activating enzyme 7	0006464 // cellular protein modification process // not recorded///0006974 // cellular response to DNA damage st
NM_001278269///NM_011815///XM_006519982///XM_006519983///XM_006				-1.35	-1.31	1.43	**1.48**	***Fyb***	FYN binding protein	0006955 // immune response // inferred from electronic annotation///0045576 // mast cell activation // inferred f
NM_001289755///N	0.40118688	0.026039016	0.6852822	-2.03	1.56	-2.13	**1.48**	***Apoc3***	apolipoprotein C-III	0006629 // lipid metabolic process // inferred from electronic annotation///0006641 // triglyceride metabolic proc
NM_134133///XM_	0.00701278	0.025884103	0.71766454	-1.21	1.26	-1.03	**1.48**	***Smim3***	small integral membrane protein 3	
NM_025989///XM_	0.008962023	0.03866082	0.034857918	-1.06	-7.17	10.02	**1.48**	***Gp2***	glycoprotein 2 (zymogen granule membrane)	0002412 // antigen transcytosis by M cells in mucosal-associated lymphoid tissue // inferred from direct assay///0
NM_009780///XM_006523531///XM_006523532///XM_006525280///XM_006				-1.23	-1.16	1.40	**1.48**	***C4b***	complement component 4B (Chido blood group)///complement C4-B	0002376 // immune system process // inferred from electronic annotation///0006954 // inflammatory response //
NM_023258				-1.43	1.05	-1.01	**1.49**	***Pycard***	PYD and CARD domain containing	0001773 // myeloid dendritic cell activation // not recorded///0002218 // activation of innate immune response //
NM_007689				-1.98	-2.24	1.70	**1.50**	***Chad***	chondroadherin	0001502 // cartilage condensation // inferred from electronic annotation///0060348 // bone development // infer
NM_008706	0.017970597	0.04884449	0.08275565	-1.07	1.36	1.03	**1.50**	***Nqo1***	NAD(P)H dehydrogenase, quinone 1	0006801 // superoxide metabolic process // not recorded///0006979 // response to oxidative stress // inferred fro
NM_010172				1.89	1.35	2.10	**1.50**	***F7***	coagulation factor VII	0002690 // positive regulation of leukocyte chemotaxis // not recorded///0006508 // proteolysis // inferred from e
NM_001013373///XM_006510132///XM_011242445				-1.66	-1.49	1.34	**1.50**	***Tmprss13***	transmembrane protease, serine 13	0006508 // proteolysis // inferred from electronic annotation///0006898 // receptor-mediated endocytosis // infer
NM_001159393///N	0.020029576	0.032851394	0.090463966	-1.04	1.49	-1.03	**1.50**	***Irf1***	interferon regulatory factor 1	0002376 // immune system process // inferred from electronic annotation///0002819 // regulation of adaptive im
NM_001029841///NM_009192///XM_006520668///XM_011245530				1.15	1.51	1.15	**1.50**	***Sla***	src-like adaptor	
NM_001252651///N	9.13E-04	0.018762708	0.020152634	-1.39	1.00	1.09	**1.51**	***Csf3r***	colony stimulating factor 3 receptor (granulocyte)	0007155 // cell adhesion // inferred from electronic annotation///0019221 // cytokine-mediated signaling pathwa
NM_011562///XM_011242957				1.15	1.60	1.08	**1.51**	***Tdgf1***	teratocarcinoma-derived growth factor 1	0000187 // activation of MAPK activity // not recorded///0001570 // vasculogenesis // inferred from mutant phen
NM_001164708///N	0.1330411	0.01662339	0.11330092	-1.52	1.21	-1.22	**1.51**	***Hspb2***	heat shock protein 2	0007525 // somatic muscle development // inferred from genetic interaction///0009408 // response to heat // not
NM_020557///XR_381188				-1.39	-1.16	1.27	**1.53**	***Cmpk2***	cytidine monophosphate (UMP-CMP) kinase 2, mitochondrial	0006165 // nucleoside diphosphate phosphorylation // not recorded///0006221 // pyrimidine nucleotide biosynth
NM_009099///XM_006507471///NM_001167828///NM_199146///XM_00650				-1.58	-1.24	1.21	**1.53**	***Trim30a///Trim30d***	tripartite motif-containing 30A///tripartite motif-containing 30D	0006351 // transcription, DNA-templated // inferred from electronic annotation///0006355 // regulation of transc
NM_001081029///XR_868243///XR_888236				1.86	1.91	1.50	**1.54**	***Tmem243***	transmembrane protein 243, mitochondrial	
NM_029495///NM_178825///XM_011244910				-1.19	-1.24	1.60	**1.55**	***Epsti1***	epithelial stromal interaction 1 (breast)	
NM_011150	1.09E-05	0.020499367	0.02188839	1.01	-1.31	2.06	**1.55**	***Lgals3bp***	lectin, galactoside-binding, soluble, 3 binding protein	0006898 // receptor-mediated endocytosis // inferred from sequence or structural similarity///0006898 // recepto
NM_001164289///NM_001164323///NM_199015///XM_006518862///XM_006				-1.26	-1.21	1.49	**1.55**	***Phf11c///Phf11d***	PHD finger protein 11C///PHD finger protein 11D	0006351 // transcription, DNA-templated // inferred from electronic annotation///0006355 // regulation of transc
NM_001164525				-1.57	-1.29	1.28	**1.56**	***Tstd1***	thiosulfate sulfurtransferase (rhodanese)-like domain containing 1	
NM_001159738///NM_016960///XM_006496447///XM_006496448				1.45	1.96	1.16	**1.56**	***Ccl20***	chemokine (C-C motif) ligand 20	0002548 // monocyte chemotaxis // ---///0006935 // chemotaxis // not recorded///0006954 // inflammatory resp
NM_007686///XM_	0.082811646	0.046391945	0.31540507	-1.19	1.35	-1.02	**1.56**	***Cfi***	complement component factor i	0002376 // immune system process // inferred from electronic annotation///0006508 // proteolysis // inferred fro
NM_026601///NR_	0.068584606	0.040043514	0.5474951	-1.20	1.46	-1.12	**1.57**	***Hyi***	hydroxypyruvate isomerase homolog (E. coli)	0008152 // metabolic process // inferred from electronic annotation
NM_001040005///XM_006534630///XM_006534631///XM_006534632///XM_				-1.23	-1.15	1.47	**1.57**	***Rnf213***	ring finger protein 213	0008152 // metabolic process // inferred from electronic annotation///0016567 // protein ubiquitination // not re
NM_001160711///N	5.36E-05	0.010786352	0.061238483	-1.30	1.00	1.21	**1.57**	***Cd300lg***	CD300 antigen like family member G	0002376 // immune system process // inferred from electronic annotation///0002414 // immunoglobulin transcyt
NM_016780///XM_006532312///XM_011248766				-1.44	-1.18	1.29	**1.58**	***Itgb3***	integrin beta 3	0001938 // positive regulation of endothelial cell proliferation // not recorded///0001954 // positive regulation of
NM_007421	0.0185965	0.033065483	0.001208518	1.22	2.02	-1.05	**1.58**	***Adssl1***	adenylosuccinate synthetase like 1	0006163 // purine nucleotide metabolic process // inferred from direct assay///0006164 // purine nucleotide biosy
NM_013640///XM_	0.004284681	0.001195478	0.60894126	-1.23	1.44	-1.12	**1.58**	***Psmb10***	proteasome (prosome, macropain) subunit, beta type 10	0000902 // cell morphogenesis // inferred from mutant phenotype///0006508 // proteolysis // inferred from elect
NM_001313712///NM_001313713///NM_001313714///NM_013673///XM_00				-1.46	-1.34	1.47	**1.60**	***Sp100***	nuclear antigen Sp100	0000723 // telomere maintenance // inferred from sequence or structural similarity///0006338 // chromatin remo
NM_011530///XM_	5.77E-04	5.73E-04	0.5744587	-1.21	1.42	-1.07	**1.60**	***Tap2***	transporter 2, ATP-binding cassette, sub-family B (MDR/TAP)	0001916 // positive regulation of T cell mediated cytotoxicity // inferred from direct assay///0001916 // positive re
NM_013612///XM_006495773///XM_006495774				-1.19	-1.01	1.36	**1.61**	***Slc11a1***	solute carrier family 11 (proton-coupled divalent metal ion transporters), member 1	0000060 // protein import into nucleus, translocation // inferred from mutant phenotype///0000165 // MAPK casc
NM_001170853///XM_006496597///XM_006496598///XM_006496599///XM_				-1.64	-1.59	1.57	**1.61**	***Ifi204///Ifi205///Mnda***	interferon activated gene 204///205///myeloid cell nuclear differentiation antigen	0000122 // negative regulation of transcription from RNA polymerase II promoter // ---///0030308 // negative regu
NM_011452				1.41	1.53	1.49	**1.61**	***Serpinb9b***	serine (or cysteine) peptidase inhibitor, clade B, member 9b	0010951 // negative regulation of endopeptidase activity // not recorded///0019835 // cytolysis // inferred from d
NM_001254744///N	0.005418789	0.028454479	0.06375431	-1.06	1.37	1.11	**1.62**	***E030030I06Rik***	RIKEN cDNA E030030I06 gene	0007049 // cell cycle // inferred from electronic annotation///0042127 // regulation of cell proliferation // inferred
NM_207281	0.1870459	0.018142508	0.001903932	1.16	2.11	-1.13	**1.62**	***Mettl21e***	methyltransferase like 21E	0032259 // methylation // inferred from electronic annotation
NM_198095				-1.24	-1.18	1.57	**1.64**	***Bst2***	bone marrow stromal cell antigen 2	0002376 // immune system process // inferred from electronic annotation///0002737 // negative regulation of pla
NM_029847///XM_006517456				-1.46	-1.02	1.16	**1.65**	***Arsk***	arylsulfatase K	0008152 // metabolic process // inferred from electronic annotation
NM_028801///XM_006508653				-2.91	-6.20	3.52	**1.65**	***Muc5b***	mucin 5, subtype B, tracheobronchial	0042742 // defense response to bacterium // inferred from mutant phenotype///0043030 // regulation of macropl
NM_183249				1.28	1.48	1.44	**1.66**	***Wfdc21***	WAP four-disulfide core domain 21	0010466 // negative regulation of peptidase activity // inferred from electronic annotation///0032496 // response
NM_008360///XM_	0.034024853	0.011973868	0.37563986	-1.21	1.58	-1.15	**1.66**	***Il18***	interleukin 18	0000165 // MAPK cascade // not recorded///0001525 // angiogenesis // not recorded///0001525 // angiogenesis
NM_013930				-1.91	-1.36	1.18	**1.66**	***Aass***	aminoadipate-semialdehyde synthase	0006091 // generation of precursor metabolites and energy // traceable author statement///0008152 // metabolic
NM_008611				1.38	1.21	1.91	**1.67**	***Mmp8***	matrix metallopeptidase 8	0006508 // proteolysis // not recorded///0030574 // collagen catabolic process // inferred from electronic annota
NM_023785				-1.89	-1.94	1.73	**1.69**	***Ppbp***	pro-platelet basic protein	0002523 // leukocyte migration involved in inflammatory response // inferred from mutant phenotype///0002690
NM_010734				-1.56	-1.38	1.49	**1.69**	***Lst1***	leukocyte specific transcript 1	0000902 // cell morphogenesis // inferred from electronic annotation///0002376 // immune system process // inf
NM_011517	0.6025549	0.04851214	0.54293686	-1.80	1.27	-1.34	**1.71**	***Sycp3***	synaptonemal complex protein 3	0000711 // meiotic DNA repair synthesis // inferred from genetic interaction///0007049 // cell cycle // inferred fro
NM_001317105///NM_133862				1.37	1.48	1.59	**1.71**	***Fgg***	fibrinogen gamma chain	0002250 // adaptive immune response // inferred from electronic annotation///0002376 // immune system proces
NM_001291892///NM_001291893///NM_001291894///NM_008147///NM_01				1.56	1.77	1.50	**1.71**	***Lilr4b///Lilrb4a***	leukocyte immunoglobulin-like receptor, subfamily B, member 4B///4A	0002250 // adaptive immune response // inferred from electronic annotation///0002376 // immune system proces
NM_026790///NM_194066///NM_194067///NM_194068///NM_194069///XM				-1.48	-1.01	1.17	**1.71**	***Ifi27***	interferon, alpha-inducible protein 27	0000122 // negative regulation of transcription from RNA polymerase II promoter // not recorded///0046825 // re
NM_030098///XM_006519698				-1.64	-1.40	1.48	**1.73**	***Rnase6***	ribonuclease, RNase A family, 6	0008152 // metabolic process // inferred from electronic annotation///0090305 // nucleic acid phosphodiester bo
NM_017371				1.16	1.74	1.16	**1.73**	***Hpx***	hemopexin	0002639 // positive regulation of immunoglobulin production // inferred from mutant phenotype///0002925 // po
NM_010746///XM_	4.05E-05	0.013767784	0.5017644	-1.26	1.07	1.29	**1.74**	***Ncr1***	natural cytotoxicity triggering receptor 1	0009597 // detection of virus // inferred from mutant phenotype///0051607 // defense response to virus // inferre
NM_009016///NM_	0.017242404	0.029976845	0.2069715	-1.18	1.38	1.07	**1.75**	***Raet1a///Raet1b///Raet1c///Raet1d///Raet1e***	retinoic acid early transcript 1, alpha/// beta/// gamma/// delta///1E	0001913 // T cell mediated cytotoxicity // not recorded///0001913 // T cell mediated cytotoxicity // inferred from
NM_009140				1.21	1.63	1.31	**1.75**	***Cxcl2***	chemokine (C-X-C motif) ligand 2	0001975 // response to amphetamine // inferred from electronic annotation///0002237 // response to molecule o
NM_001037138///NM_199221///XM_006533072				-1.57	-1.30	1.47	**1.77**	***Cd300lb***	CD300 antigen like family member B	0002376 // immune system process // inferred from electronic annotation///0002446 // neutrophil mediated imm
NM_021718				-1.80	-1.20	1.18	**1.77**	***Ms4a4b***	membrane-spanning 4-domains, subfamily A, member 4B	
NM_001111099///NM_007669				1.64	2.51	1.16	**1.78**	***Cdkn1a***	cyclin-dependent kinase inhibitor 1A (P21)	0000079 // regulation of cyclin-dependent protein serine/threonine kinase activity // inferred from mutant phenot
NM_008039				1.37	1.57	1.57	**1.79**	***Fpr2***	formyl peptide receptor 2	0006935 // chemotaxis // inferred from electronic annotation///0007165 // signal transduction // inferred from el
NM_009985///XM_	2.42E-04	0.023352936	0.114798434	-1.39	1.05	1.23	**1.80**	***Ctsw***	cathepsin W	0006508 // proteolysis // inferred from electronic annotation///0006955 // immune response // not recorded///0
NM_001166376///NM_028595///XM_006527381///XM_006527382///XR_877				-1.53	-1.35	1.59	**1.80**	***Ms4a6c///Gm8369***	membrane-spanning 4-domains, subfamily A, member 6C///predicted gene 8369	
NM_009026///XM_011248826				1.33	2.14	1.13	**1.81**	***Rasd1***	RAS, dexamethasone-induced 1	0007165 // signal transduction // inferred from electronic annotation///0007263 // nitric oxide mediated signal tr
NM_001168392///N	1.83E-05	0.023048088	0.003570888	-1.43	1.01	1.27	**1.83**	***Ramp1***	receptor (calcitonin) activity modifying protein 1	0001525 // angiogenesis // not recorded///0006810 // transport // inferred from electronic annotation///000681
NM_138654///XM_	0.01189704	0.005208184	2.45E-04	1.20	2.22	1.00	**1.84**	***Sugct***	succinyl-CoA glutarate-CoA transferase	0008152 // metabolic process // inferred from electronic annotation
NM_007498	0.00460487	0.003487884	3.81E-06	1.70	3.37	-1.07	**1.84**	***Atf3***	activating transcription factor 3	0000122 // negative regulation of transcription from RNA polymerase II promoter // inferred from direct assay///0
NM_001002898///NM_001173459///NM_001173460///NM_178792///XM_00				1.20	1.51	1.47	**1.85**	***Sirpb1a///Sirpb1b***	signal-regulatory protein beta 1-like///signal-regulatory protein beta 1A///1B	0035556 // intracellular signal transduction // inferred from direct assay///0050766 // positive regulation of phag
NM_178214				-1.58	-1.17	1.41	**1.89**	***Hist2h2be***	histone cluster 2, H2be	0002227 // innate immune response in mucosa // not recorded///0006334 // nucleosome assembly // not recorde
NM_010745				-1.68	-1.34	1.51	**1.89**	***Ly86***	lymphocyte antigen 86	0002376 // immune system process // inferred from electronic annotation///0006954 // inflammatory response //
NM_001164314///N	6.74E-06	1.62E-04	4.07E-05	1.07	1.92	1.06	**1.90**	***Wars***	tryptophanyl-tRNA synthetase	0001525 // angiogenesis // inferred from electronic annotation///0006412 // translation // inferred from electron
NM_001164059///NM_011346///XM_006496716///XM_006496717///XM_006				-1.52	-1.05	1.32	**1.91**	***Sell***	selectin, lymphocyte	0007155 // cell adhesion // inferred from electronic annotation///0033198 // response to ATP // inferred from dir
NM_019949				-1.54	-1.05	1.30	**1.91**	***Ube2l6***	ubiquitin-conjugating enzyme E2L 6	0016567 // protein ubiquitination // inferred from electronic annotation///0019941 // modification-dependent pro
NM_010395///NM_	1.37E-05	0.014577702	0.75237536	-1.14	1.30	1.30	**1.93**	***H2-T10///H2-T22///H2-T9***	histocompatibility 2, T region locus 10/// locus 22///locus 9	0002474 // antigen processing and presentation of peptide antigen via MHC class I // not recorded///0006955 // in
NM_145158///XM_006524285///XM_006524286///XM_011246461				-1.85	-1.12	1.17	**1.93**	***Emilin2***	elastin microfibril interfacer 2	0007155 // cell adhesion // inferred from electronic annotation
NM_015767///XM_	0.042712994	0.015395308	0.0317231	-1.07	2.10	-1.15	**1.96**	***Ttpa***	tocopherol (alpha) transfer protein	0001890 // placenta development // inferred from mutant phenotype///0001892 // embryonic placenta developm
NM_145227///XM_	2.01E-06	0.01974411	4.54E-04	-1.26	-1.71	2.66	**1.96**	***Oas2***	2'-5' oligoadenylate synthetase 2	0002376 // immune system process // inferred from electronic annotation///0006164 // purine nucleotide biosynt
NM_001042451///NM_009221				-1.64	-1.35	1.62	**1.97**	***Snca***	synuclein, alpha	0001774 // microglial cell activation // inferred from mutant phenotype///0001921 // positive regulation of recept
NM_010234				1.64	3.43	-1.06	**1.97**	***Fos***	FBJ osteosarcoma oncogene	0001661 // conditioned taste aversion // inferred from electronic annotation///0006355 // regulation of transcript
NM_011909				-1.19	-1.18	1.96	**1.98**	***Usp18***	ubiquitin specific peptidase 18	0006508 // proteolysis // inferred from electronic annotation///0006511 // ubiquitin-dependent protein catabolic
NM_011819///XM_	0.012883321	0.02579039	7.50E-04	1.29	2.42	1.06	**1.99**	***Gdf15***	growth differentiation factor 15	0010862 // positive regulation of pathway-restricted SMAD protein phosphorylation // not recorded///0042981 //

RefSeq Transcript ID	p (Genotype) (	p (Genotype-Exposure) (2	p (Exposure) (	FC WT Air:O3	FC KO Air:O3	FD Air WT:KO	FD O3 WT:KO	Gene Symbol	Gene Title	Gene Ontology Biological Process
NM_017373///XM_	0.15306924	0.002679221	4.19E-05	1.48	3.96	-1.34	2.00	Nfil3	nuclear factor, interleukin 3, regulated	0000122 // negative regulation of transcription from RNA polymerase II promoter // not recorded///0006351 // tra
NM_025416	0.020313757	0.042609792	0.08676615	-1.10	1.72	1.10	2.08	Them5	thioesterase superfamily member 5	0006629 // lipid metabolic process // inferred from electronic annotation///0006631 // fatty acid metabolic proces
NM_010724///XM_	2.24E-05	0.002021546	0.09661372	-1.14	1.55	1.17	2.08	Psmb8	proteasome (prosome, macropain) subunit, beta type 8 (large multifunctional peptidase 7)	0002376 // immune system process // inferred from electronic annotation///0006508 // proteolysis // inferred fro
NM_001310640///N	0.00892747	0.026918758	2.12E-05	2.09	4.28	1.01	2.08	Pmvk	phosphomevalonate kinase	0006629 // lipid metabolic process // inferred from electronic annotation///0006694 // steroid biosynthetic proces
NM_028967///XM_	6.93E-04	0.013054218	0.014988259	-1.04	1.80	1.12	2.11	Batf2	basic leucine zipper transcription factor, ATF-like 2	0006351 // transcription, DNA-templated // inferred from electronic annotation///0006355 // regulation of transc
NM_001271005///N	4.63E-05	0.011145398	0.96271	-1.21	1.36	1.30	2.14	H2-T23	histocompatibility 2, T region locus 23	0001916 // positive regulation of T cell mediated cytotoxicity // inferred from direct assay///0001916 // positive re
NM_010531///XM_	6.64E-05	0.01810247	0.001231183	1.09	1.78	1.32	2.16	Il18bp	interleukin 18 binding protein	0032496 // response to lipopolysaccharide // inferred from electronic annotation///0042088 // T-helper 1 type im
NM_001037713///NM_001291153///XM_006533567///XM_006533568///XM_				-1.24	-1.12	1.97	2.19	Xaf1	XIAP associated factor 1	0006915 // apoptotic process // inferred from electronic annotation///0031333 // negative regulation of protein c
NM_027209///XM_011247337				-1.66	-1.18	1.56	2.20	Ms4a6b	membrane-spanning 4-domains, subfamily A, member 6B	
NM_015783///XR_168557///XR_397495				-1.27	-1.62	2.85	2.24	Isg15	ISG15 ubiquitin-like modifier	0019941 // modification-dependent protein catabolic process // inferred from direct assay///0031397 // negative
NM_153590///XM_006506121///XM_006506122///XM_006506123///XM_006				-1.54	-1.28	1.89	2.26	Klre1	killer cell lectin-like receptor family E member 1	0002223 // stimulatory C-type lectin receptor signaling pathway // inferred from direct assay///0002859 // negativ
NM_001302559///N	1.27E-06	8.77E-04	1.11E-05	-1.66	-4.51	6.15	2.27	Slfn4///Slfn3	schlafen 4///schlafen 3	0008285 // negative regulation of cell proliferation // inferred from direct assay
NM_001305935///N	0.03148155	0.012993463	0.24723457	-1.92	1.48	-1.18	2.40	Bckdhb	branched chain ketoacid dehydrogenase E1, beta polypeptide	0008152 // metabolic process // inferred from electronic annotation///0009063 // cellular amino acid catabolic pr
NM_175026				-1.78	-1.76	2.43	2.45	Pyhin1	pyrin and HIN domain family, member 1	0000122 // negative regulation of transcription from RNA polymerase II promoter // ---///0035458 // cellular respo
NM_008331				-1.83	-1.70	2.32	2.51	Ifit1	interferon-induced protein with tetratricopeptide repeats 1	0002376 // immune system process // inferred from electronic annotation///0009615 // response to virus // inferr
NM_001080941///X	0.033015296	0.047486916	0.3625288	-1.97	1.24	1.06	2.60	Zfp429	zinc finger protein 429	0006355 // regulation of transcription, DNA-templated // inferred from electronic annotation
NM_011073///XM_	2.40E-06	0.013736106	0.49245003	-1.33	1.12	1.76	2.61	Prf1	perforin 1 (pore forming protein)	0001771 // immunological synapse formation // not recorded///0002357 // defense response to tumor cell // infer
NM_001314054///NM_031168				5.31	11.59	1.20	2.63	Il6	interleukin 6	0001781 // neutrophil apoptotic process // inferred from direct assay///0001781 // neutrophil apoptotic process /
NM_011403				-2.95	-1.40	1.28	2.69	Slc4a1	solute carrier family 4 (anion exchanger), member 1	0006810 // transport // inferred from electronic annotation///0006811 // ion transport // inferred from electronic
NM_139198///XM_011249430				-1.70	-1.21	1.93	2.72	Plac8	placenta-specific 8	0008284 // positive regulation of cell proliferation // inferred from direct assay///0009409 // response to cold // in
NM_029499///XM_006527250///XM_006527251				-1.85	-1.55	2.31	2.75	Ms4a4c	membrane-spanning 4-domains, subfamily A, member 4C	
NM_001143686				-2.40	-1.78	2.05	2.76	Apol11b	apolipoprotein L 11b	0006869 // lipid transport // inferred from electronic annotation///0042157 // lipoprotein metabolic process // inf
NM_013585///XM_	1.19E-05	5.73E-04	0.5460914	-1.50	1.52	1.23	2.80	Psmb9	proteasome (prosome, macropain) subunit, beta type 9 (large multifunctional peptidase 2)	0002376 // immune system process // inferred from electronic annotation///0006508 // proteolysis // inferred fro
NM_023386///XM_006522491				-1.57	-1.23	2.22	2.83	Rtp4	receptor transporter protein 4	0001580 // detection of chemical stimulus involved in sensory perception of bitter taste // not recorded///000661
NM_001161730///N	6.23E-06	7.33E-04	0.080780625	-1.29	1.70	1.32	2.90	Tap1	transporter 1, ATP-binding cassette, sub-family B (MDR/TAP)	0002250 // adaptive immune response // inferred from electronic annotation///0002376 // immune system proces
NM_173869	0.002842786	0.025394054	0.031699836	-1.03	2.22	1.28	2.94	Stfa2l1	stefin A2 like 1	0010951 // negative regulation of endopeptidase activity // inferred from electronic annotation
NM_001281830///NM_029803				-2.98	-2.64	2.68	3.02	Ifi27l2a	interferon, alpha-inducible protein 27 like 2A	0007568 // aging // inferred from direct assay///0009615 // response to virus // inferred from direct assay
NM_010654///XM_006505655///XM_006505656				-2.08	-1.19	1.73	3.03	Klrd1	killer cell lectin-like receptor, subfamily D, member 1	0002228 // natural killer cell mediated immunity // not recorded
NM_172777///XM_	7.16E-05	1.21E-04	0.010784728	-2.16	1.44	-1.03	3.04	Gbp9	guanylate-binding protein 9	0008152 // metabolic process // inferred from electronic annotation///0042832 // defense response to protozoan
NM_001205313///N	1.71E-06	9.54E-04	0.1928924	-1.26	1.65	1.49	3.08	Stat1	signal transducer and activator of transcription 1	0000122 // negative regulation of transcription from RNA polymerase II promoter // inferred from sequence or stru
NM_001252600///NM_001252601///NM_016850///XM_006536214				-1.35	-1.46	3.34	3.09	Irf7	interferon regulatory factor 7	0002376 // immune system process // inferred from electronic annotation///0002819 // regulation of adaptive im
NM_008326///XM_	4.82E-07	0.001143392	6.02E-04	1.04	1.99	1.62	3.09	Irgm1	immunity-related GTPase family M member 1	0002376 // immune system process // inferred from electronic annotation///0006914 // autophagy // inferred fro
NM_145211///XM_	9.80E-09	0.03495188	1.72E-05	-1.43	-1.68	3.66	3.10	Oas1a	2'-5' oligoadenylate synthetase 1A	0002376 // immune system process // inferred from electronic annotation///0006164 // purine nucleotide biosynt
NM_001139519///N	1.96E-06	0.021276742	0.010490653	-1.01	1.65	1.88	3.11	Zbp1	Z-DNA binding protein 1	0002376 // immune system process // inferred from electronic annotation///0008152 // metabolic process // infer
NM_001162883///NM_001168660///NM_173743///NM_173786///XM_00652				-1.48	-1.38	2.95	3.15	Apol9a///Apol9b	apolipoprotein L 9a///apolipoprotein L 9b	0006869 // lipid transport // inferred from electronic annotation///0042157 // lipoprotein metabolic process // inf
NM_144559	3.09E-06	0.00304968	0.002918902	-1.04	1.89	1.64	3.21	Fcgr4	Fc receptor, IgG, low affinity IV	0001866 // NK T cell proliferation // not recorded///0051930 // regulation of sensory perception of pain // not rec
NM_011854///XM_006530314///XM_006530315///XM_006530316				-1.39	-1.38	3.35	3.37	Oasl2	2'-5' oligoadenylate synthetase-like 2	0002376 // immune system process // inferred from electronic annotation///0006164 // purine nucleotide biosynth
NM_001293690///N	0.0037732	0.002331509	0.021634238	-1.29	2.66	1.00	3.43	Ido1	indoleamine 2,3-dioxygenase 1	0002376 // immune system process // inferred from electronic annotation///0002534 // cytokine production invol
NM_029509///XM_	0.025589565	0.007223136	0.11731671	-1.48	2.60	-1.11	3.45	Gbp8	guanylate-binding protein 8	0008152 // metabolic process // inferred from electronic annotation///0071346 // cellular response to interferon-g
NM_178386	1.03E-04	0.011639653	0.06928708	-1.16	1.80	1.66	3.45	Slc25a31	solute carrier family 25 (mitochondrial carrier; adenine nucleotide translocator), member 31	0006810 // transport // inferred from electronic annotation///0055085 // transmembrane transport // inferred fro
NM_010260	5.92E-05	1.37E-04	0.80003136	-1.75	2.04	1.01	3.59	Gbp2	guanylate binding protein 2	0008152 // metabolic process // inferred from electronic annotation///0035458 // cellular response to interferon-
NM_175449	0.002981814	0.044325806	0.6242128	-1.50	1.62	1.52	3.70	Fam26f	family with sequence similarity 26, member F	0006810 // transport // inferred from electronic annotation///0006811 // ion transport // inferred from electronic
NM_011315				4.04	2.44	6.20	3.74	Saa3	serum amyloid A 3	0006953 // acute-phase response // inferred from electronic annotation///0007252 // I-kappaB phosphorylation /
NM_001271676///N	1.75E-06	7.61E-05	0.052070796	-1.42	2.20	1.36	4.24	Ifi47	interferon gamma inducible protein 47	0006952 // defense response // inferred from mutant phenotype///0007165 // signal transduction // inferred from
NM_024253	4.18E-04	0.038715716	0.90266514	-1.64	1.48	1.75	4.25	Nkg7	natural killer cell group 7 sequence	
NM_001039160///N	3.29E-07	5.64E-05	0.29797998	-1.50	1.92	1.55	4.47	Gvin1	GTPase, very large interferon inducible 1	
NM_021384///XM_011243886				-1.82	-1.44	3.55	4.48	Rsad2	radical S-adenosyl methionine domain containing 2	0001503 // ossification // not recorded///0002376 // immune system process // inferred from electronic annotatio
NM_001136059///NM_009992///XM_006510810				-6.59	-3.43	2.36	4.53	Cyp1a1	cytochrome P450, family 1, subfamily a, polypeptide 1	0001666 // response to hypoxia // inferred from electronic annotation///0001889 // liver development // inferred
NM_030679///NM_	0.9685817	0.034358054	0.9833375	-4.93	4.86	-4.93	4.87	Myh1///Myh4	myosin, heavy polypeptide 1, skeletal muscle, adult///myosin, heavy polypeptide 4, skeletal muscle	0006936 // muscle contraction // not recorded///0008152 // metabolic process // inferred from electronic annota
NM_001083312///N	1.08E-05	3.38E-04	0.22118247	-1.58	2.29	1.36	4.93	Gbp7	guanylate binding protein 7	0008152 // metabolic process // inferred from electronic annotation///0042832 // defense response to protozoan
NM_013542	4.09E-07	0.007064547	0.08473857	-1.69	1.23	2.44	5.07	Gzmb	granzyme B	0001913 // T cell mediated cytotoxicity // inferred from mutant phenotype///0006508 // proteolysis // inferred fro
NM_001033207///X	3.61E-06	0.012560561	0.24143435	-1.29	1.75	2.25	5.08	Nlrc5	NLR family, CARD domain containing 5	0002376 // immune system process // inferred from electronic annotation///0032088 // negative regulation of NF
NM_019440	1.35E-06	1.46E-04	0.035187297	-1.41	2.39	1.55	5.20	Irgm2	immunity-related GTPase family M member 2	0034341 // response to interferon-gamma // inferred from direct assay///0034341 // response to interferon-gamm
NM_001145164///N	3.88E-07	6.50E-04	0.00193326	-1.06	2.44	2.09	5.42	Tgtp1///Tgtp2	T cell specific GTPase 1///T cell specific GTPase 2	0006955 // immune response // non-traceable author statement///0009615 // response to virus // inferred from d
NM_021893///XM_	3.39E-05	3.35E-04	5.14E-04	-1.07	4.44	1.23	5.88	Cd274	CD274 antigen	0006955 // immune response // not recorded///0007165 // signal transduction // not recorded///0007166 // cell
NM_018738	1.79E-07	3.68E-05	0.022523012	-1.51	2.59	1.84	7.20	Igtp	interferon gamma induced GTPase	0035458 // cellular response to interferon-beta // inferred from direct assay
NM_001289492///N	2.14E-06	4.39E-04	0.07993907	-2.35	1.66	1.87	7.28	Gbp3	guanylate binding protein 3	0008152 // metabolic process // inferred from electronic annotation///0035458 // cellular response to interferon-
NM_001146275///N	4.10E-06	6.57E-04	0.057767134	-1.47	2.75	1.83	7.39	Iigp1	interferon inducible GTPase 1	0002376 // immune system process // inferred from electronic annotation///0008152 // metabolic process // infer
NM_133871///XM_006502485///XM_006502486				-2.92	-1.92	5.04	7.66	Ifi44	interferon-induced protein 44	
NM_010370	2.06E-06	0.004724333	0.009065636	-2.57	1.16	2.67	7.98	Gzma	granzyme A	0006508 // proteolysis // inferred from electronic annotation///0006915 // apoptotic process // inferred from elec
NM_194336///XM_	7.76E-07	3.69E-05	0.37096202	-2.64	2.32	1.63	10.01	Gbp6	guanylate binding protein 6	0006955 // immune response // inferred from mutant phenotype///0008152 // metabolic process // inferred from
NM_001033767///X	4.80E-06	0.043635536	0.7827524	-1.50	1.72	4.07	10.48	Gm4951	predicted gene 4951	0035458 // cellular response to interferon-beta // inferred from direct assay

Table S7. Common genes altered by O3 in Tnfr- and Nfkb1-dependent manner (n=341)

Venn Diagram analysis of Tnfr-dependent genes (n=2180, pooled from 24-, 48-, and 72 hr O3) and Nfkb1-dependent genes (n=2397).

Fold difference (FD); Positive-higher in KO-O3, negative-lower in KO-O3, compared to corresponding WT-O3.

RefSeq Transcript ID	p Tnfr 24 hr	p Tnfr 48hr	p Tnfr 72 hr	p Nfkb1 (	FD Tnfr 24 hr (	FD Tnfr 48 hr (	FD Tnfr 72 hr (\	FD Nfkb1 4	Gene Symbol	Gene Title	Gene Ontology
NM_010370	0.0319457			0.03195	-1.27	-1.89	-1.36	7.98	Gzma	granzyme A	0006508 // proteolysis // inferred from electronic annotation///0006915 // apoptotic process // inferred from electro
NM_001136059///NM_009992///X		0.021329		0.02133	-1.64	1.21	-1.57	4.53	Cyp1a1	cytochrome P450, family 1, subfamily a, polypeptide 1	0001666 // response to hypoxia // inferred from electronic annotation///0001889 // liver development // inferred fro
NM_024253	0.0126742			0.01267	-1.03	-1.48	-1.30	4.25	Nkg7	natural killer cell group 7 sequence	0005515 // protein binding // inferred from electronic annotation
NM_001293690///NM_008324		0.0474173		0.04742	-1.02	-1.31	-1.08	3.43	Ido1	indoleamine 2,3-dioxygenase 1	0002376 // immune system process // inferred from electronic annotation///0002534 // cytokine production involved
NM_144559	0.0320214			0.03202	1.04	-1.04	-1.07	3.21	Fcgr4	Fc receptor, IgG, low affinity IV	0001866 // NK T cell proliferation // not recorded///0051930 // regulation of sensory perception of pain // not record
NM_013585///XM_006523730///XI		0.0428359		0.04284	1.16	-1.25	1.14	2.80	Psmb9	proteasome (prosome, macropain) subunit, beta type 9 (large multifunctional pept	0002376 // immune system process // inferred from electronic annotation///0006508 // proteolysis // inferred from e
NM_029499///XM_00	0.0458086			0.04581	1.26	1.02	-1.08	2.75	Ms4a4c	membrane-spanning 4-domains, subfamily A, member 4C	0005887 // integral component of plasma membrane // inferred from electronic annotation///0016020 // membrane ,
NM_139198///XM_01	0.0379849			0.03798	1.85	1.21	1.44	2.72	Plac8	placenta-specific 8	0008284 // positive regulation of cell proliferation // inferred from direct assay///0009409 // response to cold // infer
NM_001314054///NM	0.0109972			0.011	1.78	1.17	-1.01	2.63	Il6	interleukin 6	0001781 // neutrophil apoptotic process // inferred from direct assay///0001781 // neutrophil apoptotic process // in
NM_001271005///NM_010398///X		0.0493594		0.04936	1.02	-1.46	-1.13	2.14	H2-T23	RIKEN cDNA C920025E04 gene///histocompatibility 2, T region locus 23	0001916 // positive regulation of T cell mediated cytotoxicity // inferred from direct assay///0001916 // positive regul
NM_017373///XM_006516875///XI		0.0236738		0.02367	-1.31	-1.09	-1.34	2.00	Nfil3	nuclear factor, interleukin 3, regulated	0000122 // negative regulation of transcription from RNA polymerase II promoter // not recorded///0006351 // trans
NM_010234	0.0339224			0.03392	2.51	1.06	1.09	1.97	Fos	FBJ osteosarcoma oncogene	0001661 // conditioned taste aversion // inferred from electronic annotation///0006355 // regulation of transcription,
NM_015767///XM_01	0.0470459			0.04705	-1.19	1.07	1.03	1.96	Ttpa	tocopherol (alpha) transfer protein	0001890 // placenta development // inferred from mutant phenotype///0001892 // embryonic placenta development
NM_001164059///NM	0.0140566			0.01406	1.72	-1.04	-1.05	1.91	Sell	selectin, lymphocyte	0007155 // cell adhesion // inferred from electronic annotation///0033198 // response to ATP // inferred from direct a
NM_001111099///NM	0.0477121			0.04771	-1.83	-1.03	-1.41	1.78	Cdkn1a	cyclin-dependent kinase inhibitor 1A (P21)	0000079 // regulation of cyclin-dependent protein serine/threonine kinase activity // inferred from mutant phenotype,
NM_021718	0.0186814			0.01868	1.52	1.11	-1.09	1.77	Ms4a4b	membrane-spanning 4-domains, subfamily A, member 4B	0005887 // integral component of plasma membrane // inferred from direct assay///0016020 // membrane // inferred
NM_001291892///NM	0.0090002	0.0301939		0.03019	1.65	1.52	1.02	1.71	Lilr4b///Lilrb4a	leukocyte immunoglobulin-like receptor, subfamily B, member 4B///leukocyte imn	0002250 // adaptive immune response // inferred from electronic annotation///0002376 // immune system process /,
NM_010734	0.0188023			0.0188	1.27	-1.13	-1.09	1.69	Lst1	leukocyte specific transcript 1	0000902 // cell morphogenesis // inferred from electronic annotation///0002376 // immune system process // inferre
NM_008611	0.0280208			0.02802	1.07	-1.02	1.04	1.67	Mmp8	matrix metallopeptidase 8	0006508 // proteolysis // not recorded///0030574 // collagen catabolic process // inferred from electronic annotation
NM_183249	0.0327021		0.0431278	0.0327	1.24	1.38	1.26	1.66	Wfdc21	WAP four-disulfide core domain 21	0010466 // negative regulation of peptidase activity // inferred from electronic annotation///0032496 // response to l
NM_001159738///NM	0.0021256	0.0141236	0.0087143	0.01412	-1.33	-1.18	-1.22	1.56	Ccl20	chemokine (C-C motif) ligand 20	0002548 // monocyte chemotaxis // ---///0006935 // chemotaxis // not recorded///0006954 // inflammatory respons
NM_029495///NM_17	0.0158681			0.01587	1.09	-1.07	-1.03	1.55	Epsti1	epithelial stromal interaction 1 (breast)	
NM_001252651///NM	0.0126991			0.0127	1.24	-1.23	-1.21	1.51	Csf3r	colony stimulating factor 3 receptor (granulocyte)	0007155 // cell adhesion // inferred from electronic annotation///0019221 // cytokine-mediated signaling pathway //
NM_001013373///XM_006510132/		0.0305592		0.03056	-1.12	-1.21	-1.18	1.50	Tmprss13	transmembrane protease, serine 13	0006508 // proteolysis // inferred from electronic annotation///0006898 // receptor-mediated endocytosis // inferred
NM_010172	0.0051493	8.73E-04		8.73E-04	1.18	1.22	1.02	1.50	F7	coagulation factor VII	0002690 // positive regulation of leukocyte chemotaxis // not recorded///0006508 // proteolysis // inferred from elec
NM_009780///XM_00	0.0110776	0.0215402	0.0368332	0.02154	-1.21	-1.31	1.11	1.48	C4b	complement component 4B (Chido blood group)///complement C4-B	0002376 // immune system process // inferred from electronic annotation///0006954 // inflammatory response // inf
NM_025989///XM_006508121///XI		0.0297166		0.02972	-1.11	1.00	1.06	1.48	Gp2	glycoprotein 2 (zymogen granule membrane)	0002412 // antigen transcytosis by M cells in mucosal-associated lymphoid tissue // inferred from direct assay///0002
NM_134133///XM_006525481///XI		0.0145032		0.0145	-1.23	1.00	-1.03	1.48	Smim3	small integral membrane protein 3	0005515 // protein binding // inferred from electronic annotation
NM_001278269///NM	0.0166895			0.01669	1.40	-1.05	-1.10	1.48	Fyb	FYN binding protein	0006955 // immune response // inferred from electronic annotation///0045576 // mast cell activation // inferred from
NM_011756	0.0041983			0.0042	1.46	1.08	1.13	1.46	Zfp36	zinc finger protein 36	0000122 // negative regulation of transcription from RNA polymerase II promoter // inferred from mutant phenotype/
NM_178911///XM_01	0.0032975			0.0033	1.50	-1.10	-1.05	1.45	Pld4	phospholipase D family, member 4	0002244 // hematopoietic progenitor cell differentiation // inferred from mutant phenotype///0006629 // lipid metab
NM_008842	0.0062451			0.00625	1.29	-1.11	1.01	1.45	Pim1	proviral integration site 1	0006468 // protein phosphorylation // not recorded///0007346 // regulation of mitotic cell cycle // not recorded///00
NM_007440///XM_011248676///XI		0.0202748		0.02027	-1.04	-1.25	-1.01	1.42	Alox12	arachidonate 12-lipoxygenase	0006629 // lipid metabolic process // inferred from electronic annotation///0006631 // fatty acid metabolic process /,
NM_010501///XM_01	0.048251			0.04825	1.29	-1.08	1.28	1.41	Ifit3	interferon-induced protein with tetratricopeptide repeats 3	0002376 // immune system process // inferred from electronic annotation///0008285 // negative regulation of cell pr
NM_009396///XM_006515792///XI		0.0045507		0.00455	1.09	-1.20	1.02	1.40	Tnfaip2	tumor necrosis factor, alpha-induced protein 2	0001525 // angiogenesis // inferred from electronic annotation///0006887 // exocytosis // not recorded///0007275 //
NM_001127348///NM	0.0269581			0.02696	1.07	1.03	1.09	1.37	Snx10	sorting nexin 10	0006810 // transport // inferred from electronic annotation///0006897 // endocytosis // not recorded///0007032 // e
NM_138683///XM_006502862		0.0042547		0.00425	-1.05	-1.21	-1.09	1.37	Rspo1	R-spondin 1	0001934 // positive regulation of protein phosphorylation // not recorded///0002090 // regulation of receptor interna
NM_019521	4.51E-04	0.0107763		0.01078	-1.25	-1.26	1.04	1.36	Gas6	growth arrest specific 6	0001764 // neuron migration // inferred from genetic interaction///0001934 // positive regulation of protein phospho
NM_025427	0.0063318			0.00633	-1.65	-1.17	-1.05	1.35	Rgcc	regulator of cell cycle	0000082 // G1/S transition of mitotic cell cycle // not recorded///0001100 // negative regulation of exit from mitosis /
NM_009151///XM_00	0.0478423			0.04784	1.21	-1.01	-1.06	1.35	Selplg	selectin, platelet (p-selectin) ligand	0007155 // cell adhesion // inferred from electronic annotation///0050901 // leukocyte tethering or rolling // inferred
NM_013599///XM_00	0.0026954			0.0027	1.07	-1.29	-1.46	1.34	Mmp9	matrix metallopeptidase 9	0001501 // skeletal system development // inferred from genetic interaction///0001501 // skeletal system developme
NM_026835		0.0483779		0.04838	1.14	1.58	1.15	1.33	Ms4a6d	membrane-spanning 4-domains, subfamily A, member 6D	0016020 // membrane // inferred from electronic annotation///0016021 // integral component of membrane // inferr
NM_153071///XM_01	0.0285745			0.02857	-1.10	-1.02	-1.07	1.33	Gprc6a	G protein-coupled receptor, family C, group 6, member A	0007165 // signal transduction // inferred from electronic annotation///0007186 // G-protein coupled receptor signali
NM_001033301///NM_001205355,		0.02936		0.02936	-1.57	-1.60	-1.14	1.31	Fhdc1	FH2 domain containing 1	
NM_015786		0.0114646		0.01146	-1.04	-1.37	-1.14	1.31	Hist1h1c	histone cluster 1, H1c	0000122 // negative regulation of transcription from RNA polymerase II promoter // inferred from genetic interaction/
NM_001001892///NM_001267808,		0.0411866		0.04119	1.05	-1.18	-1.08	1.30	H2-D1///H2-K1///H2-L///H2-Q2///H2-Q6	histocompatibility 2, D region locus 1///histocompatibility 2, K1, K region///histocc	0001916 // positive regulation of T cell mediated cytotoxicity // inferred from direct assay///0002250 // adaptive imm
NM_145953///XM_006500815		0.0206117		0.02061	-1.24	1.33	-1.05	1.29	Cth	cystathionase (cystathionine gamma-lyase)	0006749 // glutathione metabolic process // not recorded///0008285 // negative regulation of cell proliferation // not
NM_001164885///NM	0.0081215	0.0186868		0.01869	-1.24	-1.06	1.16	1.27	Lpin2	lipin 2	0006351 // transcription, DNA-templated // inferred from electronic annotation///0006355 // regulation of transcripti
NM_001160163///NM_001160164,		0.03408		0.03408	-1.02	-1.23	-1.05	1.26	Neu2	neuraminidase 2	0005975 // carbohydrate metabolic process // inferred from electronic annotation///0006629 // lipid metabolic proce
NM_013566///XM_00	0.0116526			0.01165	1.31	-1.13	-1.16	1.25	Itgb7	integrin beta 7	0003366 // cell-matrix adhesion involved in ameboidal cell migration // not recorded///0007155 // cell adhesion // inf
NM_016675///XM_00	0.0374343			0.03743	1.14	1.00	-1.01	1.25	Cldn2	claudin 2	0016338 // calcium-independent cell-cell adhesion via plasma membrane cell-adhesion molecules // inferred from dire
NM_029688	0.0112739			0.01127	1.15	1.06	-1.03	1.24	Srxn1	sulfiredoxin 1 homolog (S. cerevisiae)	0006979 // response to oxidative stress // inferred from direct assay///0006979 // response to oxidative stress // not r
NM_001252479///NM	0.0489366			0.04894	1.19	-1.03	-1.19	1.24	Cmtm7	CKLF-like MARVEL transmembrane domain containing 7	0002337 // B-1a B cell differentiation // inferred from mutant phenotype///0006935 // chemotaxis // inferred from el
NM_001316372///NM_008796///X		0.0276464		0.02765	1.06	1.08	1.07	1.24	Pctp	phosphatidylcholine transfer protein	0006810 // transport // inferred from electronic annotation///0006869 // lipid transport // inferred from electronic ar
NM_011401///XM_00	0.0387578			0.03876	1.08	-1.29	-1.11	1.23	Slc2a3	solute carrier family 2 (facilitated glucose transporter), member 3	0006810 // transport // inferred from electronic annotation///0008643 // carbohydrate transport // inferred from ele
NM_001100109///NM	0.0357954			0.0358	1.05	1.06	1.15	1.23	Srp54a///Srp54b///Srp54c	signal recognition particle 54A///signal recognition particle 54B///signal recognitio	0006614 // SRP-dependent cotranslational protein targeting to membrane // not recorded///0008152 // metabolic pr
NM_011189	0.0427805			0.04278	1.22	1.05	1.31	1.22	Psme1	proteasome (prosome, macropain) activator subunit 1 (PA28 alpha)	0002479 // antigen processing and presentation of exogenous peptide antigen via MHC class I, TAP-dependent // not r
NM_001037859///NM_007779///X		0.0395433		0.03954	1.03	-1.12	-1.02	1.22	Csf1r	colony stimulating factor 1 receptor	0001934 // positive regulation of protein phosphorylation // not recorded///0002376 // immune system process // inf
NM_007802///XM_006500974		0.046115		0.04611	1.08	1.26	1.04	1.21	Ctsk	cathepsin K	0001957 // intramembranous ossification // inferred from electronic annotation///0006508 // proteolysis // inferred f
NM_022331///XM_00	1.41E-04			1.41E-04	-1.56	1.12	-1.09	1.21	Herpud1	homocysteine-inducible, endoplasmic reticulum stress-inducible, ubiquitin-like don	0006511 // ubiquitin-dependent protein catabolic process // inferred from direct assay///0006511 // ubiquitin-depend
NM_153510	4.81E-04	0.0113849	0.0129494	0.01138	1.15	1.17	-1.10	1.21	Pilra	paired immunoglobin-like type 2 receptor alpha	0007165 // signal transduction // not recorded///0007169 // transmembrane receptor protein tyrosine kinase signalin
NM_010555///XM_00	0.0030509	0.0411284		0.04113	1.20	-1.03	-1.20	1.21	Il1r2	interleukin 1 receptor, type II	0019221 // cytokine-mediated signaling pathway // inferred from electronic annotation
NM_181821	0.0450951			0.0451	-1.12	-1.06	1.01	1.20	Hcfc1r1	host cell factor C1 regulator 1 (XPO1-dependent)	0005634 // nucleus // inferred from electronic annotation///0005654 // nucleoplasm // not recorded///0005737 // cy
NM_001111015///NM_013681///X		0.0076582	0.0071612	0.00766	-1.04	-1.14	-1.13	1.20	Syn2	synapsin II	0007269 // neurotransmitter secretion // inferred from genetic interaction///0007269 // neurotransmitter secretion /
NM_001177945///NM	0.0309011			0.0309	-1.10	-1.01	1.02	1.19	Aamdc	adipogenesis associated Mth938 domain containing	0043066 // negative regulation of apoptotic process // inferred from mutant phenotype///0045600 // positive regulat
NM_001286037///NM	0.0066966			0.0067	1.45	1.09	-1.14	1.19	Ncf1	neutrophil cytosolic factor 1	0001878 // response to yeast // inferred from mutant phenotype///0001909 // leukocyte mediated cytotoxicity // infe
NM_008490		6.30E-05		6.30E-05	1.01	1.12	-1.03	1.19	Lcat	lecithin cholesterol acyltransferase	0006629 // lipid metabolic process // inferred from electronic annotation///0006644 // phospholipid metabolic proces
NM_001113248///NM	0.0299019			0.0299	1.29	1.08	1.00	1.19	Sin3b	transcriptional regulator, SIN3B (yeast)	0000122 // negative regulation of transcription from RNA polymerase II promoter // not recorded///0006351 // transc
NM_001290525///NM	0.0023673			0.00237	1.19	-1.02	-1.20	1.19	Blvrb	biliverdin reductase B (flavin reductase (NADPH))	0042167 // heme catabolic process // not recorded///0055114 // oxidation-reduction process // inferred from electro
NM_010476///XM_00	0.0374562			0.03746	1.23	1.21	1.03	1.18	Hsd17b7	hydroxysteroid (17-beta) dehydrogenase 7	0006629 // lipid metabolic process // inferred from electronic annotation///0006694 // steroid biosynthetic process //
NM_001310705///NM	0.0397531			0.03975	-1.20	-1.13	-1.14	1.18	Slc16a4	solute carrier family 16 (monocarboxylic acid transporters), member 4	0006810 // transport // inferred from electronic annotation///0015718 // monocarboxylic acid transport // ---///0034
NM_001253751///NM	0.006113	0.0114804		0.01148	1.59	1.76	1.46	1.17	Fdps	farnesyl diphosphate synthetase	0006629 // lipid metabolic process // inferred from electronic annotation///0006694 // steroid biosynthetic process //
NM_009999		0.0400147		0.04001	-1.22	-1.24	1.26	1.17	Cyp2b10	cytochrome P450, family 2, subfamily b, polypeptide 10	0006805 // xenobiotic metabolic process // not recorded///0008202 // steroid metabolic process // not recorded///0
NM_028024		0.0497831		0.04978	1.15	1.02	1.03	1.17	Nkiras2	NFKB inhibitor interacting Ras-like protein 2	0007165 // signal transduction // inferred from electronic annotation///0007264 // small GTPase mediated signal tran
NM_001033297		0.040065		0.04007	-1.08	1.05	-1.11	1.17	Gm561	predicted gene 561	0005739 // mitochondrion // inferred from direct assay
NM_010344	0.0017858			0.00179	1.29	1.07	1.10	1.16	Gsr	glutathione reductase	0006749 // glutathione metabolic process // inferred by curator///0006749 // glutathione metabolic process // not re
NM_001311156///NM_133986///X		0.0248389		0.02484	1.10	-1.02	1.05	1.16	Tcta	T cell leukemia translocation altered gene	0016020 // membrane // inferred from electronic annotation///0016021 // integral component of membrane // inferr
NM_001083959///NM_001083960,		0.0283362	0.0177956	0.02834	-1.06	-1.23	-1.16	1.16	Spo11	SPO11 meiotic protein covalently bound to DSB	0000237 // leptotene // inferred from mutant phenotype///0000737 // DNA catabolic process, endonucleolytic // infe
NM_001038604///NM	0.0475167			0.04752	1.19	1.04	-1.06	1.16	Clec5a	C-type lectin domain family 5, member a	0002076 // osteoblast development // inferred from direct assay///0002376 // immune system process // inferred fro
NM_024220///XM_00	0.0349543			0.03495	-1.07	-1.06	1.05	1.14	LOC102641347///Ndufc2	NADH dehydrogenase [ubiquinone] 1 subunit C2///NADH dehydrogenase (ubiquin	0006120 // mitochondrial electron transport, NADH to ubiquinone // inferred from electronic annotation///0006810 /
NM_001252578///NM	0.0046366			0.00464	1.23	1.03	1.01	1.14	Sulf2	sulfatase 2	0001822 // kidney development // inferred from genetic interaction///0002063 // chondrocyte development // inferre
NM_013711	0.007202			0.0072	1.16	-1.03	-1.10	1.14	Txnrd2	thioredoxin reductase 2	0000305 // response to oxygen radical // traceable author statement///0007507 // heart development // inferred fro
NM_001080707///NM	0.0263567			0.02636	-1.21	-1.10	1.62	1.14	Gpr155	G protein-coupled receptor 155	0035556 // intracellular signal transduction // inferred from electronic annotation///0050890 // cognition // not recor

RefSeq Transcript ID	p Tnfr 24 hr	p Tnfr 48hr	p Tnfr 72 hr	p Nfkb1 (	FD Tnfr 24 hr (	FD Tnfr 48 hr (	FD Tnfr 72 hr (	FD Nfkb1 (	Gene Symbol	Gene Title	Gene Ontology
NM_008304	0.0223832			0.02238	-1.07	1.12	1.12	1.14	***Sdc2***	syndecan 2	0007399 // nervous system development // inferred from electronic annotation///0016477 // cell migration // not rec
NM_021472///NM_20	0.0040024	0.0497113		0.04971	-1.12	-1.02	-1.01	1.13	***Rnase4***	ribonuclease, RNase A family 4	0001525 // angiogenesis // inferred from electronic annotation///0001525 // angiogenesis // ---///0001525 // angioge
NM_001039373///NM	0.0017875	0.0108495	0.010648	0.01085	-1.12	-1.05	-1.05	1.13	***Cmc4///Mtcp1***	C-x(9)-C motif containing 4///mature T cell proliferation 1	0005739 // mitochondrion // inferred from direct assay///0005739 // mitochondrion // not recorded
NM_001177833///NM	0.0479175			0.04792	-1.08	-1.06	-1.12	1.13	***Smox***	spermine oxidase	0006598 // polyamine catabolic process // not recorded///0046208 // spermine catabolic process // inferred from dir
NM_019814///XM_006512201///XI	0.0321375			0.03214	-1.03	1.11	1.04	1.12	***Higd1a***	HIG1 domain family, member 1A	0006810 // transport // inferred from electronic annotation///0042149 // cellular response to glucose starvation // inf
NM_001039373///XM_	0.0017875	0.0108495	0.010648	0.01085	-1.12	-1.05	-1.05	1.12	***Mtcp1///Cmc4***	mature T cell proliferation 1///C-x(9)-C motif containing 4	0005739 // mitochondrion // inferred from direct assay///0005739 // mitochondrion // not recorded
XM_011238903///XM_011238926/	0.0332051			0.03321	2.61	3.75	3.71	1.12	***Gm37416///Gm39743///Gm40611///Gm408***	predicted gene, 37416///predicted gene, 39743///predicted gene, 40611///predic	0006278 // RNA-dependent DNA replication // inferred from electronic annotation///0008152 // metabolic process //
NM_008416	0.001622			0.00162	1.90	1.19	1.03	1.12	***Junb***	jun B proto-oncogene	0000122 // negative regulation of transcription from RNA polymerase II promoter // not recorded///0001570 // vascu
NM_001289476///NM	0.0021963			0.0022	1.24	1.19	-1.05	1.12	***Psma7***	proteasome (prosome, macropain) subunit, alpha type 7	0006508 // proteolysis // inferred from electronic annotation///0006511 // ubiquitin-dependent protein catabolic pro
NM_011794///XM_006497159///XI	0.0120047	0.0153885		0.012	-1.20	1.09	1.16	1.12	***Bpnt1***	bisphosphate 3'-nucleotidase 1	0006139 // nucleobase-containing compound metabolic process // not recorded///0006470 // protein dephosphoryla
NM_001038587///NM	0.0499708			0.04997	1.31	1.19	1.09	1.12	***Adar***	adenosine deaminase, RNA-specific	0001649 // osteoblast differentiation // inferred from mutant phenotype///0001701 // in utero embryonic developme
NM_019777///XM_00	0.0031129			0.00311	1.08	1.03	-1.07	1.12	***Ikbke***	inhibitor of kappaB kinase epsilon	0006468 // protein phosphorylation // not recorded///0006974 // cellular response to DNA damage stimulus // inferre
NM_153059///XM_00	0.0430095			0.04301	1.03	-1.04	-1.14	1.11	***Tmem5***	transmembrane protein 5	0006486 // protein glycosylation // inferred from electronic annotation///0035269 // protein O-linked mannosylation
NM_008086	0.0454716	0.0028526		0.00285	-1.00	-1.12	-1.06	1.10	***Gas1***	growth arrest specific 1	0002053 // positive regulation of mesenchymal cell proliferation // inferred from mutant phenotype///0007049 // cell
NM_029796	0.0029807	0.0054629		0.00546	2.00	1.45	-1.04	1.10	***Lrg1***	leucine-rich alpha-2-glycoprotein 1	0001938 // positive regulation of endothelial cell proliferation // inferred from mutant phenotype///0030511 // positiv
NM_001136077///NM	0.0211292			0.02113	-1.06	-1.00	1.04	1.10	***Enpp2***	ectonucleotide pyrophosphatase/phosphodiesterase 2	0001953 // negative regulation of cell-matrix adhesion // not recorded///0006629 // lipid metabolic process // inferre
NM_010902///NR_132	0.0178916			0.01789	-1.17	1.13	1.15	1.10	***Nfe2l2///LOC105244211***	nuclear factor, erythroid derived 2, like 2///uncharacterized LOC105244211	0006351 // transcription, DNA-templated // inferred from electronic annotation///0006355 // regulation of transcripti
NM_025912///XM_006500059		0.0208285		0.02083	1.02	-1.01	-1.09	1.10	***Fam210b***	family with sequence similarity 210, member B	0016020 // membrane // inferred from electronic annotation///0016021 // integral component of membrane // inferr
NM_007435		0.0338033		0.0338	1.17	-1.04	1.12	1.10	***Abcd1***	ATP-binding cassette, sub-family D (ALD), member 1	0006635 // fatty acid beta-oxidation // inferred from mutant phenotype///0006635 // fatty acid beta-oxidation // not
NM_001101430///NM	0.0037818			0.00378	1.18	1.01	-1.14	1.10	***Psmg4***	proteasome (prosome, macropain) assembly chaperone 4	0043248 // proteasome assembly // inferred from electronic annotation
NM_029096///NM_133908///XR_8	0.026953	0.0095988		0.02695	1.04	-1.12	-1.10	1.10	***Rita1///Gm38465***	RBPJ interacting and tubulin associated 1///predicted gene, 38465	0007219 // Notch signaling pathway // inferred from electronic annotation///0007399 // nervous system developmen
NM_145156///XM_006527097///XI	0.0138632			0.01386	1.12	-1.09	-1.11	1.10	***Slc25a28***	solute carrier family 25, member 28	0006810 // transport // inferred from electronic annotation///0006811 // ion transport // inferred from electronic anr
NM_175309		0.0163726		0.01637	1.05	-1.14	1.18	1.09	***Upk3b***	uroplakin 3B	0010629 // negative regulation of gene expression // not recorded///0046325 // negative regulation of glucose import
NM_001161769///NM	0.0088986			0.0089	1.11	1.01	-1.11	1.09	***Lmo4***	LIM domain only 4	0001843 // neural tube closure // inferred from mutant phenotype///0003281 // ventricular septum development // in
NM_001177594///NM_001177595,	0.0282329			0.02823	-1.01	-1.10	1.04	1.09	***Slc8b1***	solute carrier family 8 (sodium/lithium/calcium exchanger), member B1	0006810 // transport // inferred from electronic annotation///0006811 // ion transport // inferred from electronic anr
NM_024432///XM_006524796		0.0342473		0.03425	1.02	-1.11	-1.08	1.09	***Ubxn6***	UBX domain protein 6	0005634 // nucleus // not recorded///0005730 // nucleolus // not recorded///0005737 // cytoplasm // inferred from e
NM_021789///XR_870	0.0358103	0.0389616		0.03896	-1.07	-1.10	-1.14	1.08	***Trappc4***	trafficking protein particle complex 4	0006810 // transport // inferred from electronic annotation///0006888 // ER to Golgi vesicle-mediated transport // no
NM_001314013///NM_053069///X	0.0303102	0.0273162		0.03031	1.06	1.10	1.15	1.07	***Atg5***	autophagy related 5	0000045 // autophagosome assembly // inferred from mutant phenotype///0000422 // mitophagy // not recorded///
NM_001045514///NM	0.0013916			0.00139	1.28	1.02	-1.18	1.07	***Akna***	AT-hook transcription factor	0006351 // transcription, DNA-templated // inferred from electronic annotation///0006355 // regulation of transcripti
NM_008344	0.0206806	0.0259694		0.02597	1.09	-1.19	-1.07	1.06	***Igfbp6***	insulin-like growth factor binding protein 6	0001558 // regulation of cell growth // inferred from electronic annotation///0043567 // regulation of insulin-like grov
NM_053159	0.0137382	9.06E-04		9.06E-04	1.13	1.13	-1.01	1.06	***Mrpl3***	mitochondrial ribosomal protein L3	0006412 // translation // not recorded
NM_001291503///NM	0.0243809			0.02438	-1.17	-1.05	1.07	1.06	***Birc2***	baculoviral IAP repeat-containing 2	0000209 // protein polyubiquitination // not recorded///0001666 // response to hypoxia // not recorded///0001890 /
NM_007621///XM_00	0.0096765	0.0158171		0.01582	-1.03	-1.10	1.26	1.05	***Cbr2***	carbonyl reductase 2	0006116 // NADH oxidation // traceable author statement///0008152 // metabolic process // inferred from electronic
NM_153516///XM_00	0.0107404			0.01074	1.12	1.11	1.07	1.05	***Bcl2l13***	BCL2-like 13 (apoptosis facilitator)	0006915 // apoptotic process // inferred from reviewed computational analysis///0042981 // regulation of apoptotic p
NM_009025///XM_00	0.0010731			0.00107	1.40	1.06	1.08	1.05	***Rasa3***	RAS p21 protein activator 3	0007165 // signal transduction // inferred from electronic annotation///0034605 // cellular response to heat // not rec
NM_031248		0.0389777		0.03898	1.06	1.12	1.09	1.05	***Lamtor2***	late endosomal/lysosomal adaptor, MAPK and MTOR activator 2	0000186 // activation of MAPKK activity // inferred from direct assay///0016049 // cell growth // not recorded///0032
NM_023232		0.0192033		0.0192	1.06	1.13	1.13	1.05	***Diablo***	diablo homolog (Drosophila)	0006915 // apoptotic process // inferred from electronic annotation///0006919 // activation of cysteine-type endopep
NM_026124	0.0058328			0.00583	1.15	-1.03	-1.07	1.04	***1110008F13Rik***	RIKEN cDNA 1110008F13 gene	0005739 // mitochondrion // inferred from direct assay
NM_011512///XM_00	0.0020724			0.00207	1.10	1.05	1.18	1.03	***Surf4***	surfeit gene 4	0007030 // Golgi organization // not recorded///0010638 // positive regulation of organelle organization // not recorc
NM_031381///XM_01	0.0018739			0.00187	-1.13	-1.11	-1.02	1.03	***Tex13***	testis expressed gene 13	
NM_139197///XM_006497918///XI	0.0320381			0.03204	1.00	-1.20	-1.14	1.03	***Gbgt1***	globoside alpha-1,3-N-acetylgalactosaminyltransferase 1	0005975 // carbohydrate metabolic process // inferred from electronic annotation///0006486 // protein glycosylation
NM_175263///XM_006534494///XI	0.0257043	0.0320829		0.0257	-1.03	-1.26	-1.16	1.03	***Notum***	notum pectinacetylesterase homolog (Drosophila)	0006507 // GPI anchor release // inferred from direct assay///0016055 // Wnt signaling pathway // inferred from elect
NM_001206367///NM	0.0299643			0.02996	-1.21	-1.21	-1.01	1.02	***Gsn***	gelsolin	0006911 // phagocytosis, engulfment // inferred from mutant phenotype///0006915 // apoptotic process // not recor
NM_001037940///NM_001037941,	0.0184684			0.01847	-1.06	-1.08	1.05	1.02	***Dnajb6***	DnaJ (Hsp40) homolog, subfamily B, member 6	0006457 // protein folding // not recorded///0030036 // actin cytoskeleton organization // inferred from mutant pher
NM_145513///XM_00	0.0471327			0.04713	-1.07	1.04	1.12	1.01	***Tiprl***	TIP41, TOR signalling pathway regulator-like (S. cerevisiae)	0000077 // DNA damage checkpoint // not recorded///0034048 // negative regulation of protein phosphatase type 2A
NM_001290437///NM_033134///N	0.0400683	0.0426836		0.04007	-1.01	-1.03	-1.03	1.01	***Inpp5e***	inositol polyphosphate-5-phosphatase E	0006470 // protein dephosphorylation // inferred from direct assay///0006470 // protein dephosphorylation // inferre
NM_001048057///NM_001048058,	0.0323493			0.03235	-1.01	1.10	1.04	1.01	***Rpl38***	ribosomal protein L38	0001501 // skeletal system development // inferred from mutant phenotype///0001501 // skeletal system developme
NM_053068	0.0047056			0.00471	1.21	1.02	1.02	1.01	***Chrac1***	chromatin accessibility complex 1	0008152 // metabolic process // inferred from electronic annotation///0071897 // DNA biosynthetic process // inferre
NM_008403///XM_00	0.0161564			0.01616	1.14	1.08	-1.02	-1.01	***Itgb1bp1***	integrin beta 1 binding protein 1	0001525 // angiogenesis // inferred from electronic annotation///0002043 // blood vessel endothelial cell proliferation
NM_013536	0.0155215	0.0396649		0.03966	1.12	1.18	1.21	-1.01	***Emg1***	EMG1 nucleolar protein homolog (S. cerevisiae)	0001824 // blastocyst development // inferred from mutant phenotype///0006364 // rRNA processing // inferred from
NM_145404///XM_00	0.0270387			0.02704	1.08	1.03	-1.04	-1.01	***Prmt7***	protein arginine N-methyltransferase 7	0000387 // spliceosomal snRNP assembly // not recorded///0006349 // regulation of gene expression by genetic impri
NM_001033201		0.0271094		0.02711	1.03	1.12	1.05	-1.02	***Ncbp1***	nuclear cap binding protein subunit 1	0000184 // nuclear-transcribed mRNA catabolic process, nonsense-mediated decay // not recorded///0006370 // 7-m
NM_026113///XM_00	0.0459785	0.0056938		0.00569	1.14	1.30	1.08	-1.02	***Gtf3c6***	general transcription factor IIIC, polypeptide 6, alpha	0006351 // transcription, DNA-templated // not recorded///0006383 // transcription from RNA polymerase III promot
NM_026526///XM_00	0.0444804	0.0311002		0.0311	1.06	1.11	-1.02	-1.02	***N6amt2***	N-6 adenine-specific DNA methyltransferase 2 (putative)	0032259 // methylation // inferred from electronic annotation
NM_030109///XM_006531771		0.0383358		0.03834	1.03	1.09	1.21	-1.02	***Sf3b2***	splicing factor 3b, subunit 2	0000398 // mRNA splicing, via spliceosome // not recorded
NM_025919///XM_00	0.0015412			0.00154	1.22	1.15	1.04	-1.02	***Rpl11***	ribosomal protein L11	0000027 // ribosomal large subunit assembly // ---///0006364 // rRNA processing // not recorded///0006412 // transla
NM_001159354///NM	0.0272379	0.039371		0.03937	1.44	1.40	1.47	-1.03	***Magi3***	membrane associated guanylate kinase, WW and PDZ domain containing 3	0007165 // signal transduction // inferred from electronic annotation///0016310 // phosphorylation // inferred from e
NM_001166506///NM	0.0202679			0.02027	-1.18	-1.02	1.06	-1.03	***Sec14l1***	SEC14-like lipid binding 1	0006810 // transport // inferred from electronic annotation
NM_018874	0.0080419	0.0112728		0.01127	-1.04	-1.06	1.01	-1.03	***Pnliprp1***	pancreatic lipase related protein 1	0006629 // lipid metabolic process // inferred from electronic annotation
NM_008409	0.0482514			0.04825	1.13	1.11	1.12	-1.03	***Itm2a***	integral membrane protein 2A	0005515 // protein binding // inferred from electronic annotation
NM_001110251///NM	0.0225088			0.02251	1.24	1.20	1.01	-1.04	***Hmbs***	hydroxymethylbilane synthase	0001666 // response to hypoxia // inferred from electronic annotation///0006779 // porphyrin-containing compound
NM_001102455///NM	0.0055096			0.00551	-1.06	1.10	1.10	-1.04	***Aplp2***	amyloid beta (A4) precursor-like protein 2	0001967 // suckling behavior // inferred from genetic interaction///0006878 // cellular copper ion homeostasis // infe
NM_011482///XM_00	0.016299	0.0106377		0.01064	1.09	1.20	1.20	-1.04	***LOC100862468///Nhp2l1***	NHP2-like protein 1///NHP2 non-histone chromosome protein 2-like 1 (S. cerevisia	0006397 // mRNA processing // inferred from electronic annotation///0007338 // single fertilization // inferred from c
NM_026030		0.0230266		0.02303	1.01	1.10	1.01	-1.04	***Eif2s2***	eukaryotic translation initiation factor 2, subunit 2 (beta)	0001701 // in utero embryonic development // inferred from mutant phenotype///0002176 // male germ cell prolifera
NM_001098227///NM	0.0280715			0.02807	-1.01	1.14	1.09	-1.05	***Sdcbp***	syndecan binding protein	0007265 // Ras protein signal transduction // inferred from physical interaction///0007268 // synaptic transmission //
NM_001033481///XM_	0.0375939			0.03759	-1.12	1.01	1.09	-1.05	***Myrf***	myelin regulatory factor	0006351 // transcription, DNA-templated // inferred from electronic annotation///0006355 // regulation of transcripti
NM_001081392///NM_133874///N	0.0393782			0.03938	-1.03	1.02	-1.05	-1.05	***Mdn1***	midasin homolog (yeast)	0000027 // ribosomal large subunit assembly // inferred from electronic annotation///0008152 // metabolic process /
NM_008947	0.0364044			0.0364	1.11	1.36	1.42	-1.05	***Psmc1***	protease (prosome, macropain) 26S subunit, ATPase 1	0030163 // protein catabolic process // inferred from electronic annotation///0030433 // ER-associated ubiquitin-dep
NM_016906	0.0263981			0.0264	1.23	1.18	1.08	-1.05	***Sec61a1***	Sec61 alpha 1 subunit (S. cerevisiae)	0006614 // SRP-dependent cotranslational protein targeting to membrane // not recorded///0006620 // posttranslatio
NM_033523///XM_006514456///XI	0.0225539			0.02255	-1.01	-1.04	1.08	-1.05	***Spred2***	sprouty-related, EVH1 domain containing 2	0000188 // inactivation of MAPK activity // inferred from mutant phenotype///0007275 // multicellular organismal de
NM_001003930///NM	0.0186533			0.01865	-1.05	1.04	1.00	-1.05	***Rtn3***	reticulon 3	0006810 // transport // inferred from electronic annotation///0006915 // apoptotic process // inferred from electroni
NM_025828		0.0120557		0.01206	-1.02	1.16	1.09	-1.06	***Lman2***	lectin, mannose-binding 2	0006810 // transport // inferred from electronic annotation///0006890 // retrograde vesicle-mediated transport, Golg
NM_018749///XM_00	0.019631			0.01963	1.18	1.06	1.28	-1.06	***Eif3d***	eukaryotic translation initiation factor 3, subunit D	0001731 // formation of translation preinitiation complex // inferred from electronic annotation///0001732 // format
NM_021535	0.0227045			0.0227	1.22	1.24	1.15	-1.06	***Smu1***	smu-1 suppressor of mec-8 and unc-52 homolog (C. elegans)	0005634 // nucleus // inferred from electronic annotation///0005737 // cytoplasm // inferred from electronic annotat
NM_001291146///NM_001291147,	0.0080358			0.00804	-1.10	1.14	1.09	-1.06	***Derl2***	Der1-like domain family, member 2	0001967 // suckling behavior // inferred from mutant phenotype///0006986 // response to unfolded protein // inferre
NM_011715///XM_00	0.0047672	9.85E-04		9.85E-04	1.16	1.31	1.40	-1.06	***Wdr1***	WD repeat domain 1	0007605 // sensory perception of sound // not recorded///0030036 // actin cytoskeleton organization // inferred from
NM_001081373///NM_172531///X	0.0272016	0.0014043		0.0272	1.04	-1.09	-1.23	-1.06	***Cep164***	centrosomal protein 164	0006281 // DNA repair // inferred from electronic annotation///0006974 // cellular response to DNA damage stimulus
NM_025834///XM_006508856///XI	0.0235327			0.02353	1.02	1.04	-1.14	-1.06	***Proz***	protein Z, vitamin K-dependent plasma glycoprotein	0006508 // proteolysis // inferred from electronic annotation///0007596 // blood coagulation // inferred from direct a
NM_001290368///NM_001290369,	0.0292121			0.02921	-1.03	1.18	1.12	-1.06	***Nmt2***	N-myristoyltransferase 2	0006499 // N-terminal protein myristoylation // traceable author statement///0018008 // N-terminal peptidyl-glycine
NM_145479///XM_006522039///XI	0.0195267			0.01953	-1.05	-1.08	-1.07	-1.06	***Klhl22***	kelch-like 22	0000070 // mitotic sister chromatid segregation // not recorded///0006513 // protein monoubiquitination // not reco
NM_013765///XM_00	0.0111855			0.01119	1.19	-1.05	-1.01	-1.06	***LOC102642963///Rps26***	40S ribosomal protein S26-like///ribosomal protein S26	0006412 // translation // inferred from electronic annotation///0033119 // negative regulation of RNA splicing // not r
NM_011653		0.0078703		0.00787	-1.00	1.09	-1.01	-1.06	***Tuba1a***	tubulin, alpha 1A	0007017 // microtubule-based process // inferred from electronic annotation///0008152 // metabolic process // infer
NM_001160404///NM	0.0223027	0.043067		0.04307	1.06	1.24	1.03	-1.06	***Galnt1***	UDP-N-acetyl-alpha-D-galactosamine:polypeptide N-acetylgalactosaminyltransfera	0006486 // protein glycosylation // inferred from electronic annotation///0006493 // protein O-linked glycosylation //
NM_025445///XM_006521253///XI	0.0286346			0.02863	-1.00	1.02	1.10	-1.06	***Arfgap3***	ADP-ribosylation factor GTPase activating protein 3	0006810 // transport // inferred from electronic annotation///0009306 // protein secretion // inferred from electronic
NM_001008542///NM_001008543,	0.0058271			0.00583	1.04	-1.11	1.07	-1.07	***Mxi1***	MAX interactor 1, dimerization protein	0000122 // negative regulation of transcription from RNA polymerase II promoter // inferred from direct assay///0006
NM_011070	0.0317024			0.0317	1.05	-1.01	1.14	-1.07	***Pfdn2***	prefoldin 2	0006457 // protein folding // inferred from direct assay///0051495 // positive regulation of cytoskeleton organization

RefSeq Transcript ID	p Tnfr 24 hr	p Tnfr 48hr	p Tnfr 72 hr	p Nfkb1 (	FD Tnfr 24 hr (	FD Tnfr 48 hr (	FD Tnfr 72 hr (\	FD Nfkb1 (	Gene Symbol	Gene Title	Gene Ontology
NM_145940		0.0444011		0.0444	-1.03	-1.07	1.02	-1.07	***Wipi1***	WD repeat domain, phosphoinositide interacting 1	0000045 // autophagosome assembly // not recorded///0000422 // mitophagy // not recorded///0006497 // protein
NM_019447///XM_00	0.0164451			0.01645	-1.17	-1.16	1.03	-1.07	***Hgfac***	hepatocyte growth factor activator	0006508 // proteolysis // inferred from electronic annotation
NM_011072	0.0362198			0.03622	1.26	1.05	-1.13	-1.07	***Pfn1***	profilin 1	0001843 // neural tube closure // inferred from genetic interaction///0006357 // regulation of transcription from RNA
NM_001110310///NM_001110311,		0.0153699		0.01537	1.03	1.08	1.06	-1.07	***Snx12***	sorting nexin 12	0006810 // transport // inferred from electronic annotation///0006897 // endocytosis // not recorded///0010629 // r
NM_008638///XM_00	0.0030243	0.0035288		0.00353	1.09	1.12	-1.14	-1.07	***Mthfd2***	methylenetetrahydrofolate dehydrogenase (NAD+ dependent), methenyltetrahydr	0006730 // one-carbon metabolic process // not recorded///0008152 // metabolic process // inferred from electronic
NM_011952	0.0407381			0.04074	-1.00	1.07	1.21	-1.07	***Mapk3***	mitogen-activated protein kinase 3	0000165 // MAPK cascade // not recorded///0000189 // MAPK import into nucleus // not recorded///0001934 // posi
NM_001252332///NM	0.033312			0.03331	1.02	1.18	1.16	-1.07	***8-Sep***	septin 8	0000166 // nucleotide binding // inferred from electronic annotation///0005515 // protein binding // inferred from ele
NM_019426///XM_00	0.0166639			0.01666	1.31	-1.02	1.06	-1.07	***Atf7ip***	activating transcription factor 7 interacting protein	0000122 // negative regulation of transcription from RNA polymerase II promoter // inferred from direct assay///0006
NM_009503	0.0176248			0.01762	1.19	1.13	1.00	-1.07	***Vcp***	valosin containing protein	0006281 // DNA repair // inferred from electronic annotation///0006302 // double-strand break repair // not recorde
NM_010617///XM_006516861///XI		0.0273545		0.02735	-1.08	1.13	1.10	-1.08	***Kif13a***	kinesin family member 13A	0000910 // cytokinesis // not recorded///0006810 // transport // inferred from electronic annotation///0006886 // in
NM_001145034	0.0461378			0.04614	1.29	-1.21	-1.20	-1.08	***Gm13889***	predicted gene 13889	
NM_029416///XM_00	0.0297776	0.030468	0.0099994	0.03047	-1.06	-1.04	-1.18	-1.08	***Klf17***	Kruppel-like factor 17	0000122 // negative regulation of transcription from RNA polymerase II promoter // inferred from direct assay///0006
NM_009226		0.0290011		0.029	1.15	1.53	1.17	-1.08	***Snrpd1***	small nuclear ribonucleoprotein D1	0000245 // spliceosomal complex assembly // not recorded///0000387 // spliceosomal snRNP assembly // not record
NM_019427///XM_00	0.022712			0.02271	-1.20	-1.11	1.15	-1.08	***Epb41l4b***	erythrocyte membrane protein band 4.1 like 4b	0010628 // positive regulation of gene expression // not recorded///0031032 // actomyosin structure organization //
NM_025578		0.0099199		0.00992	-1.02	1.12	1.00	-1.08	***Mrps25***	mitochondrial ribosomal protein S25	0032543 // mitochondrial translation // inferred by curator
NM_001039184///XM	0.031606			0.03161	-1.23	-1.02	1.16	-1.09	***Cep350***	centrosomal protein 350	0034453 // microtubule anchoring // inferred from electronic annotation
NM_023773///XM_006519640///XI		0.042539	0.0209159	0.04254	-1.02	-1.05	-1.11	-1.09	***Mphosph8***	M-phase phosphoprotein 8	0006351 // transcription, DNA-templated // inferred from electronic annotation///0006355 // regulation of transcripti
NM_001190804///NM_001190805,		0.0188629		0.01886	-1.06	1.23	-1.03	-1.09	***Dnajb11***	DnaJ (Hsp40) homolog, subfamily B, member 11	0006457 // protein folding // inferred from electronic annotation///0016556 // mRNA modification // inferred from di
NM_001081196///XM	0.014817			0.01482	-1.12	1.02	1.18	-1.09	***Hnrnpul2***	heterogeneous nuclear ribonucleoprotein U-like 2	0005515 // protein binding // inferred from physical interaction///0044822 // poly(A) RNA binding // not recorded
NM_001163662///NM	0.0439748			0.04397	-1.11	1.03	1.07	-1.09	***Nucb1***	nucleobindin 1	0072718 // response to cisplatin // inferred from electronic annotation///1903533 // regulation of protein targeting //
NM_001306143///NM	0.038381			0.03838	1.13	1.09	-1.17	-1.10	***Mbd3***	methyl-CpG binding domain protein 3	0000122 // negative regulation of transcription from RNA polymerase II promoter // inferred from direct assay///0001
NM_001081144///NM	0.006173			0.00617	-1.10	-1.06	-1.10	-1.10	***Zfp518b***	zinc finger protein 518B	0006351 // transcription, DNA-templated // inferred from electronic annotation///0006355 // regulation of transcripti
NM_001305284///NM_001305286,		0.0499463		0.04995	-1.28	1.09	1.06	-1.10	***Mpdz***	multiple PDZ domain protein	0007155 // cell adhesion // inferred from direct assay///0042552 // myelination // not recorded
NM_001161782///NM	0.041009			0.04101	-1.22	-1.09	1.13	-1.10	***Trim32***	tripartite motif-containing 32	0000209 // protein polyubiquitination // not recorded///0001894 // tissue homeostasis // inferred from mutant phen
NM_026514///XM_006524295		0.003048		0.00305	-1.08	-1.24	-1.08	-1.10	***Cdc42ep3***	CDC42 effector protein (Rho GTPase binding) 3	0007266 // Rho protein signal transduction // not recorded///0008360 // regulation of cell shape // not recorded///00
NM_010485///XM_006508698		0.0330496	0.014663	0.03305	-1.02	1.20	1.15	-1.10	***Elavl1***	ELAV (embryonic lethal, abnormal vision)-like 1 (Hu antigen R)	0006417 // regulation of translation // not recorded///0045727 // positive regulation of translation // inferred from di
NM_144918	0.0236148	0.012548		0.01255	1.14	1.10	-1.00	-1.10	***Smyd5***	SET and MYND domain containing 5	0032259 // methylation // inferred from electronic annotation
NM_133933		0.012904		0.0129	-1.06	1.18	-1.12	-1.10	***Rpn1***	ribophorin I	0006486 // protein glycosylation // inferred from electronic annotation///0018279 // protein N-linked glycosylation vi
NM_172015///XM_006516826		0.0330718		0.03307	-1.05	1.08	1.02	-1.10	***Iars***	isoleucine-tRNA synthetase	0001649 // osteoblast differentiation // not recorded///0006412 // translation // inferred from electronic annotation/
NM_133895///XM_006504271///XI		0.0200086		0.02001	-1.04	1.14	1.08	-1.10	***Slc15a4***	solute carrier family 15, member 4	0006810 // transport // inferred from electronic annotation///0006857 // oligopeptide transport // inferred from elec
NM_001163513///NM	0.039438		0.0068113	0.03944	1.09	1.22	1.22	-1.10	***Dlg5***	discs, large homolog 5 (Drosophila)	0006461 // protein complex assembly // inferred from mutant phenotype///0030859 // polarized epithelial cell differe
NM_134010///XM_006513018		0.0363082		0.03631	1.09	1.12	1.08	-1.10	***Nup107***	nucleoporin 107	0000973 // posttranscriptional tethering of RNA polymerase II gene DNA at nuclear periphery // ---///0006355 // regul
NM_001286040///NM	0.015112			0.01511	-1.15	-1.11	1.00	-1.11	***Anks1***	ankyrin repeat and SAM domain containing 1	0006929 // substrate-dependent cell migration // inferred from direct assay///0016322 // neuron remodeling // inferr
NM_011966///XM_00	2.60E-04		0.0334973	2.60E-04	1.20	1.36	1.21	-1.11	***Psma4***	proteasome (prosome, macropain) subunit, alpha type 4	0006508 // proteolysis // inferred from electronic annotation///0006511 // ubiquitin-dependent protein catabolic pro
NM_001113470///NM_146012		0.0247841		0.02478	1.16	-1.03	1.04	-1.11	***Ctdsp2***	CTD (carboxy-terminal domain, RNA polymerase II, polypeptide A) small phosphata	0001933 // negative regulation of protein phosphorylation // inferred from direct assay///0006470 // protein dephosp
NM_001290486///NM_010336///N		0.0072765		0.00728	-1.21	1.06	-1.06	-1.11	***Lpar1***	lysophosphatidic acid receptor 1	0000187 // activation of MAPK activity // inferred from direct assay///0007165 // signal transduction // inferred from
NM_001311790///NM_009982///X		0.0318398	0.0362418	0.03184	1.09	1.20	1.14	-1.11	***Ctsc***	cathepsin C	0001913 // T cell mediated cytotoxicity // inferred from genetic interaction///0006508 // proteolysis // inferred from
NM_009538	0.0410406			0.04104	-1.44	-1.10	-1.13	-1.11	***Plagl1***	pleiomorphic adenoma gene-like 1	0006351 // transcription, DNA-templated // inferred from electronic annotation///0006357 // regulation of transcripti
XR_378553///XR_404558///XR_862		0.030526		0.03053	-1.18	-1.18	-1.04	-1.11	***1500002F19Rik***	RIKEN cDNA 1500002F19 gene	
NM_007637///XM_00	0.0226375	0.0214593		0.02146	1.03	1.15	1.08	-1.11	***Cct5***	chaperonin containing Tcp1, subunit 5 (epsilon)	0006457 // protein folding // inferred from electronic annotation///0007339 // binding of sperm to zona pellucida // i
NM_007591		0.0175542	0.0223384	0.01755	-1.11	1.25	1.22	-1.11	***Calr***	calreticulin	0000122 // negative regulation of transcription from RNA polymerase II promoter // not recorded///0002502 // pepti
NM_133942///XM_00	0.0158775			0.01588	-1.14	1.11	1.07	-1.11	***Plekha1***	pleckstrin homology domain containing, family A (phosphoinositide binding specifi	0001553 // luteinization // inferred from genetic interaction///0007283 // spermatogenesis // inferred from mutant pl
NM_001039351///NM_001039352,		0.0012504		0.00125	1.07	1.24	1.30	-1.12	***Nolc1***	nucleolar and coiled-body phosphoprotein 1	0007000 // nucleolus organization // inferred from direct assay
NM_026554		0.0129665		0.01297	-1.01	1.26	1.16	-1.12	***Ncbp2***	nuclear cap binding protein subunit 2	0000184 // nuclear-transcribed mRNA catabolic process, nonsense-mediated decay // not recorded///0000398 // mR
NM_011594	0.0493615			0.04936	1.02	1.08	1.13	-1.12	***Timp2***	tissue inhibitor of metalloproteinase 2	0007417 // central nervous system development // inferred from electronic annotation///0007568 // aging // inferred
NM_017376///NM_15	0.0071118	0.0109324		0.01093	1.11	1.11	1.74	-1.12	***Tef***	thyrotroph embryonic factor	0006351 // transcription, DNA-templated // inferred from electronic annotation///0006355 // regulation of transcripti
NM_011119	0.035631			0.03563	1.08	1.01	1.17	-1.12	***Pa2g4***	proliferation-associated 2G4	0006351 // transcription, DNA-templated // inferred from electronic annotation///0006355 // regulation of transcripti
NM_198885///XM_00	0.0320757			0.03208	-1.04	1.01	-1.07	-1.13	***Scx***	scleraxis	0001707 // mesoderm formation // inferred from mutant phenotype///0001894 // tissue homeostasis // inferred from
NM_001080126///NM	0.0209222			0.02092	1.24	1.15	1.17	-1.13	***Casp8***	caspase 8	0001525 // angiogenesis // inferred from mutant phenotype///0001841 // neural tube formation // inferred from mut
NM_001253857///NM	0.0490024			0.049	-1.09	-1.11	-1.09	-1.13	***Tet1***	tet methylcytosine dioxygenase 1	0001826 // inner cell mass cell differentiation // inferred from mutant phenotype///0006351 // transcription, DNA-ten
NM_001177371///NM	0.0117465			0.01175	1.09	-1.07	-1.12	-1.13	***Dbn1***	drebrin 1	0007015 // actin filament organization // inferred from direct assay///0007275 // multicellular organismal developme
NM_001290817///NM_009242		0.0465537		0.04655	-1.15	1.02	1.00	-1.13	***Sparc***	secreted acidic cysteine rich glycoprotein	0001503 // ossification // inferred from electronic annotation///0001937 // negative regulation of endothelial cell pro
NM_001290696///NM	0.0054834			0.00548	-1.04	1.02	1.05	-1.13	***Foxj3***	forkhead box J3	0006351 // transcription, DNA-templated // inferred from electronic annotation///0006355 // regulation of transcripti
NM_018826		0.0260593		0.02606	1.10	-1.27	-1.14	-1.14	***Irx5***	Iroquois related homeobox 5 (Drosophila)	0002027 // regulation of heart rate // inferred from mutant phenotype///0006351 // transcription, DNA-templated //
NM_001040690///NM	0.0455639			0.04556	-1.02	1.04	1.17	-1.14	***Rap1gds1***	RAP1, GTP-GDP dissociation stimulator 1	0007264 // small GTPase mediated signal transduction // not recorded///0014829 // vascular smooth muscle contract
NM_026532///NR_033574///XM_0		0.0396536		0.02483	1.02	1.22	1.29	-1.14	***Nutf2///Nutf2-ps1///Nutf2-ps2***	nuclear transport factor 2///nuclear transport factor 2, pseudogene 1///nuclear tr	0000060 // protein import into nucleus, translocation // not recorded///0006606 // protein import into nucleus // infe
NM_001168672///NM	0.0411733			0.04117	-1.03	-1.04	-1.05	-1.14	***Gtse1***	G two S phase expressed protein 1	0005515 // protein binding // inferred from electronic annotation
NM_001291818///NM	0.0134032			0.0134	-1.02	-1.11	-1.02	-1.14	***Rhbdf1***	rhomboid family 1 (Drosophila)	0006508 // proteolysis // inferred from electronic annotation///0008283 // cell proliferation // not recorded///0016
NM_001160229///NM	0.0458186			0.04582	-1.15	1.09	1.06	-1.14	***Zfp275***	zinc finger protein 275	0006355 // regulation of transcription, DNA-templated // inferred from electronic annotation
NM_001038625///NM_021372///X		0.0183489	0.0351841	0.01835	1.26	1.04	1.08	-1.14	***Sertad2***	SERTA domain containing 2	0006351 // transcription, DNA-templated // inferred from electronic annotation///0006355 // regulation of transcripti
NM_009840		0.013385		0.01338	-1.10	1.27	1.31	-1.15	***Cct8***	chaperonin containing Tcp1, subunit 8 (theta)	0006457 // protein folding // inferred from electronic annotation///0007339 // binding of sperm to zona pellucida // i
NM_001081225///NM	9.44E-04			9.44E-04	-1.40	-1.08	1.12	-1.15	***Fam178a***	family with sequence similarity 178, member A	0006281 // DNA repair // inferred from electronic annotation///0006974 // cellular response to DNA damage stimulus
NM_001085492///XM	3.17E-04			3.17E-04	-1.03	1.03	1.34	-1.15	***Rere***	arginine glutamic acid dipeptide (RE) repeats	0000122 // negative regulation of transcription from RNA polymerase II promoter // inferred from mutant phenotype/
NM_011526		0.0031575		0.00316	1.02	1.61	1.41	-1.15	***Tagln***	transgelin	0007010 // cytoskeleton organization // inferred from mutant phenotype///0030855 // epithelial cell differentiation //
NM_001109991///NM	0.0124967	0.0411962		0.0412	1.09	1.05	-1.08	-1.15	***Col18a1***	collagen, type XVIII, alpha 1	0001525 // angiogenesis // inferred from direct assay///0001525 // angiogenesis // inferred from mutant phenotype//
NM_018829///XM_006519292///XI		0.048007		0.04801	1.08	1.22	1.15	-1.15	***Ap3m1***	adaptor-related protein complex 3, mu 1 subunit	0006810 // transport // inferred from electronic annotation///0006886 // intracellular protein transport // inferred fro
NM_017474///XM_006501444///N		0.0154733		0.01547	1.13	3.08	1.26	-1.15	***Clca1///Clca3a1///Clca2///Clca3a2***	chloride channel accessory 1///chloride channel accessory 3A1///chloride channel	0006508 // proteolysis // inferred from electronic annotation///0006810 // transport // inferred from electronic anno
NM_001079908///NM	0.0271286		0.0430669	0.02713	-1.03	-1.05	1.39	-1.15	***Fgfr1***	fibroblast growth factor receptor 1	0000122 // negative regulation of transcription from RNA polymerase II promoter // inferred from mutant phenotype/
NM_009320///XM_006505870///XI		0.029736		0.02974	1.20	-1.02	1.12	-1.16	***Slc6a6***	solute carrier family 6 (neurotransmitter transporter, taurine), member 6	0001762 // beta-alanine transport // inferred from direct assay///0006810 // transport // inferred from electronic ann
NM_175251	0.0033594			0.00336	-1.21	-1.04	1.00	-1.16	***Arid2***	AT rich interactive domain 2 (ARID, RFX-like)	0006337 // nucleosome disassembly // not recorded///0006355 // regulation of transcription, DNA-templated // infer
NM_028149///NM_134030///XM_		0.03244		0.03244	1.03	-1.12	1.04	-1.16	***Fbxl20***	F-box and leucine-rich repeat protein 20	0001662 // behavioral fear response // inferred from mutant phenotype///0016567 // protein ubiquitination // ---///0
NM_080850///XM_006529665///XI		0.0067312		0.00673	-1.00	1.09	-1.16	-1.16	***Pask***	PAS domain containing serine/threonine kinase	0006468 // protein phosphorylation // not recorded///0016310 // phosphorylation // inferred from electronic annota
NM_001253822///NM	0.0169116			0.01691	1.38	-1.04	1.06	-1.16	***Irx3***	Iroquois related homeobox 3	0001656 // metanephros development // inferred from expression pattern///0001822 // kidney development // not re
NM_001025427///NM	0.0353727			0.03537	1.24	-1.13	1.14	-1.16	***Hmga1///Hmga1-rs1***	high mobility group AT-hook 1///high mobility group AT-hook I, related sequence 1	0006284 // base-excision repair // not recorded///0006351 // transcription, DNA-templated // inferred from electroni
NM_016671///XM_01	0.0179603			0.01796	1.14	-1.12	1.01	-1.17	***Il27ra***	interleukin 27 receptor, alpha	0002827 // positive regulation of T-helper 1 type immune response // inferred from mutant phenotype///0002829 // r
NM_007991///XM_00	0.0411783			0.04118	1.21	1.18	1.00	-1.17	***Fbl///LOC102643269***	fibrillarin///rRNA 2'-O-methyltransferase fibrillarin	0000494 // box C/D snoRNA 3'-end processing // not recorded///0001649 // osteoblast differentiation // not recorded
NM_026375///XM_00	0.0350196			0.03502	-1.06	1.03	1.17	-1.17	***Ahctf1***	AT hook containing transcription factor 1	0000910 // cytokinesis // not recorded///0006351 // transcription, DNA-templated // inferred from electronic annota
NM_013630///XM_006523834///XI		0.0106132		0.01061	-1.12	-1.11	1.15	-1.17	***Pkd1***	polycystic kidney disease 1 homolog	0001502 // cartilage condensation // inferred from mutant phenotype///0001568 // blood vessel development // infer
NM_001177629///NM_010345///X		0.01247		0.01247	-1.17	1.12	1.08	-1.18	***Grb10***	growth factor receptor bound protein 10	0007165 // signal transduction // traceable author statement///0008286 // insulin receptor signaling pathway // not re
NM_001290421///NM_010227///X		0.0147036		0.0147	1.14	1.25	1.31	-1.18	***Flna***	filamin, alpha	0001837 // epithelial to mesenchymal transition // inferred from genetic interaction///0007195 // adenylate cyclase-in
NM_009371///NM_02	0.0327741	0.0313765		0.03138	1.14	1.25	1.18	-1.18	***Tgfbr2***	transforming growth factor, beta receptor II	0001569 // patterning of blood vessels // inferred from mutant phenotype///0001570 // vasculogenesis // inferred fro
NM_011655	0.0116306	0.0433744		0.04337	1.11	1.08	1.17	-1.18	***Tubb5***	tubulin, beta 5 class I	0006457 // protein folding // traceable author statement///0007017 // microtubule-based process // inferred from di
NM_001201413///NM_001201414,		0.0197478		0.01975	-1.15	1.06	-1.10	-1.18	***Apbb2***	amyloid beta (A4) precursor protein-binding, family B, member 2	0001764 // neuron migration // inferred from genetic interaction///0006355 // regulation of transcription, DNA-templ
NM_010880///XM_00	0.0391968			0.0392	1.44	1.29	1.45	-1.19	***Ncl***	nucleolin	0001525 // angiogenesis // not recorded///0006897 // endocytosis // not recorded///0007283 // spermatogenesis //
NM_001003817	0.0477071			0.04771	1.07	1.04	1.01	-1.19	***Erbb2***	v-erb-b2 erythroblastic leukemia viral oncogene homolog 2, neuro/glioblastoma d	0001889 // liver development // inferred from electronic annotation///0001934 // positive regulation of protein phosp
NM_010480		0.0218822		0.02188	-1.59	1.31	1.05	-1.19	***Hsp90aa1***	heat shock protein 90, alpha (cytosolic), class A member 1	0001764 // neuron migration // not recorded///0006457 // protein folding // inferred from electronic annotation///00

RefSeq Transcript ID	p Tnfr 24 hr	p Tnfr 48hr	p Tnfr 72 hr	p Nfkb1 (	FD Tnfr 24 hr (	FD Tnfr 48 hr (	FD Tnfr 72 hr (	FD Nfkb1 (	Gene Symbol	Gene Title	Gene Ontology
NM_001080819///XM_	0.0110352			0.01104	-1.01	1.02	1.17	-1.19	*Arid1a*	AT rich interactive domain 1A (SWI-like)	0000122 // negative regulation of transcription from RNA polymerase II promoter // inferred from direct assay///0001
NM_008491	0.004128	0.0110278		0.01103	1.67	1.43	1.37	-1.20	*Lcn2*	lipocalin 2	0002376 // immune system process // inferred from electronic annotation///0006810 // transport // inferred from ele
NM_001122958///NM_001122959,		0.0142584		0.01426	-1.04	1.01	-1.01	-1.20	*Rad54l*	RAD54 like (S. cerevisiae)	0000724 // double-strand break repair via homologous recombination // inferred from genetic interaction///0000724
NM_001039537///NM	0.033169			0.03317	1.17	-1.05	-1.08	-1.20	*Lif*	leukemia inhibitory factor	0001974 // blood vessel remodeling // inferred from genetic interaction///0006955 // immune response // inferred fro
NM_011063///XM_00	0.0108294			0.01083	-1.08	1.24	1.13	-1.21	*Pea15a*	phosphoprotein enriched in astrocytes 15A	0000077 // DNA damage checkpoint // inferred from electronic annotation///0006810 // transport // inferred from el
NM_001267622///NM	0.0262599	0.0426134		0.04261	-1.08	-1.15	1.02	-1.21	*Ttc28*	tetratricopeptide repeat domain 28	0007049 // cell cycle // inferred from electronic annotation///0007067 // mitotic nuclear division // inferred from elec
NM_008057	0.0300766			0.03008	-1.01	-1.03	1.15	-1.21	*Fzd7*	frizzled homolog 7 (Drosophila)	0006355 // regulation of transcription, DNA-templated // not recorded///0007165 // signal transduction // inferred fro
NM_007392///XM_006526606		0.0490329		0.04903	-1.13	1.38	1.12	-1.22	*Acta2*	actin, alpha 2, smooth muscle, aorta	0006936 // muscle contraction // not recorded///0008217 // regulation of blood pressure // inferred from mutant phe
NM_178595///XR_374	0.044167			0.04417	1.10	1.04	-1.10	-1.23	*Ptrh1*	peptidyl-tRNA hydrolase 1 homolog (S. cerevisiae)	0008152 // metabolic process // inferred from electronic annotation
NM_022022///XM_00	0.0354374			0.03544	1.05	-1.06	-1.09	-1.23	*Ube4b*	ubiquitination factor E4B	0000209 // protein polyubiquitination // inferred from direct assay///0000209 // protein polyubiquitination // inferred
NM_001079686///NM	0.008956			0.00896	-1.12	-1.00	1.06	-1.23	*Syne1*	spectrin repeat containing, nuclear envelope 1	0007030 // Golgi organization // not recorded///0040023 // establishment of nucleus localization // inferred from mut
NM_016966///NR_03	0.0299773		0.0302086	0.02998	1.30	1.03	1.36	-1.23	*Gm8096///Phgdh*	3-phosphoglycerate dehydrogenase pseudogene///3-phosphoglycerate dehydroge	0006541 // glutamine metabolic process // inferred from mutant phenotype///0006544 // glycine metabolic process /
NM_001146707///NM_015781///X		0.0012336	0.0369231	0.00123	-1.06	-1.09	-1.03	-1.23	*Gm4204///Nap1l1*	nucleosome assembly protein 1-like 1 pseudogene///nucleosome assembly protei	0006334 // nucleosome assembly // inferred from electronic annotation///0014010 // Schwann cell proliferation // in
NM_021457		0.0017932		0.00179	1.05	1.16	1.16	-1.23	*Fzd1*	frizzled homolog 1 (Drosophila)	0001934 // positive regulation of protein phosphorylation // inferred from mutant phenotype///0003149 // membran
NM_011416///NM_02	0.0351283			0.03513	-1.10	1.23	1.18	-1.24	*Smarca2*	SWI/SNF related, matrix associated, actin dependent regulator of chromatin, subfa	0006325 // chromatin organization // traceable author statement///0006334 // nucleosome assembly // traceable au
NM_133229		0.0442596		0.04426	-1.18	-1.34	-1.16	-1.24	*Ripply3*	ripply3 homolog (zebrafish)	0000122 // negative regulation of transcription from RNA polymerase II promoter // inferred from direct assay///0006
NM_021288///NR_000040///NR_03		0.020285	0.0216754	0.014	-1.02	1.01	1.21	-1.24	*Tyms///Tyms-ps*	thymidylate synthase///thymidylate synthase, pseudogene	0006206 // pyrimidine nucleobase metabolic process // inferred from electronic annotation///0006231 // dTMP biosy
NM_009697///NM_183261///XM_(		0.0330305		0.03303	1.05	-1.03	1.06	-1.24	*Nr2f2*	nuclear receptor subfamily 2, group F, member 2	0000122 // negative regulation of transcription from RNA polymerase II promoter // inferred from direct assay///0000
NM_001163847///NM	0.0118024			0.0118	1.11	-1.03	-1.09	-1.25	*Ntn3///Tbc1d24*	netrin 3///TBC1 domain family, member 24	0006355 // regulation of transcription, DNA-templated // not recorded///0007409 // axonogenesis // traceable autho
NM_001039129///NM	0.0050777			0.00323	1.53	1.18	1.20	-1.25	*Hnrnpa1*	heterogeneous nuclear ribonucleoprotein A1 pseudogene	0000380 // alternative mRNA splicing, via spliceosome // inferred from direct assay///0006397 // mRNA processing //
NM_133218///XM_00	0.0087123	0.0344265		0.03443	-1.24	-1.12	1.06	-1.26	*Zfp704*	zinc finger protein 704	0046872 // metal ion binding // inferred from electronic annotation
NM_029682///XM_00	0.0475493			0.04755	1.03	-1.10	-1.09	-1.27	*Stambpl1*	STAM binding protein like 1	0006508 // proteolysis // inferred from electronic annotation
NM_001276408///NM	0.007884	0.0043191		0.00432	1.22	1.24	1.16	-1.27	*Fn1*	fibronectin 1	0001525 // angiogenesis // inferred from electronic annotation///0001775 // cell activation // not recorded///000193
NM_197999///XM_00	0.0017728			0.00177	-1.11	1.06	1.12	-1.27	*Ces2g*	carboxylesterase 2G	0008152 // metabolic process // inferred from electronic annotation
NM_001111043///NM_001111044,		0.0108745		0.01087	-1.57	1.20	1.02	-1.29	*Serpinh1*	serine (or cysteine) peptidase inhibitor, clade H, member 1	0003433 // chondrocyte development involved in endochondral bone morphogenesis // inferred from mutant phenot
NM_023850///XM_006500388///XI		0.0050417		0.00504	-1.03	-1.24	-1.06	-1.30	*Chst1*	carbohydrate (keratan sulfate Gal-6) sulfotransferase 1	0005975 // carbohydrate metabolic process // inferred from electronic annotation///0006012 // galactose metabolic
NM_133918///XM_00	0.024689			0.02469	1.14	1.07	-1.06	-1.30	*Emilin1*	elastin microfibril interfacer 1	0007155 // cell adhesion // inferred from electronic annotation///0010811 // positive regulation of cell-substrate adhe
NM_008250	0.0036699			0.00367	1.54	1.09	-1.30	-1.31	*Hlx*	H2.0-like homeobox	0001889 // liver development // inferred from mutant phenotype///0006351 // transcription, DNA-templated // inferr
NM_009363		0.0242412		0.02424	-1.01	1.59	1.12	-1.31	*Tff2*	trefoil factor 2 (spasmolytic protein 1)	0008284 // positive regulation of cell proliferation // inferred from genetic interaction///0008284 // positive regulatio
NM_001136086///NM_001291455,		0.0243615		0.02436	-1.07	1.16	1.10	-1.31	*Dpysl3*	dihydropyrimidinase-like 3	0007399 // nervous system development // inferred from direct assay///0008152 // metabolic process // inferred fror
NM_028232///XM_01	0.0261336	0.0267846		0.02678	-1.06	-1.06	1.07	-1.32	*Sgol1*	shugoshin-like 1 (S. pombe)	0007049 // cell cycle // inferred from electronic annotation///0007059 // chromosome segregation // not recorded//
NM_001122733///NM	0.0134443	0.0438303	0.0482141	0.04383	1.33	1.34	1.50	-1.33	*Kit*	kit oncogene	0000187 // activation of MAPK activity // not recorded///0001541 // ovarian follicle development // inferred from mu
NM_001171187///NM	2.53E-04	0.0034257		0.00343	1.38	1.26	1.10	-1.35	*Mal*	myelin and lymphocyte protein, T cell differentiation protein	0042552 // myelination // not recorded///1902043 // positive regulation of extrinsic apoptotic signaling pathway via d
NM_007743		0.0392629		0.03926	-1.12	1.19	1.01	-1.36	*Col1a2*	collagen, type I, alpha 2	0001501 // skeletal system development // not recorded///0001568 // blood vessel development // not recorded///0
NM_008861///XM_00	0.0468099			0.04681	-1.20	-1.13	1.08	-1.37	*Pkd2*	polycystic kidney disease 2	0001658 // branching involved in ureteric bud morphogenesis // inferred from electronic annotation///0001822 // kid
NM_172924///XM_006511167///XI		0.0201199		0.02012	1.09	1.19	-1.05	-1.37	*Peak1*	pseudopodium-enriched atypical kinase 1	0006468 // protein phosphorylation // not recorded///0016310 // phosphorylation // inferred from electronic annota
NM_026555///XM_006540986///XI		0.0247598		0.02476	-1.03	1.13	-1.24	-1.41	*Rcn3*	reticulocalbin 3, EF-hand calcium binding domain	0005509 // calcium ion binding // inferred from electronic annotation///0005515 // protein binding // inferred from el
NM_012012///XM_006496862///XI		0.0195294		0.01953	1.04	1.10	-1.06	-1.41	*Exo1*	exonuclease 1	0002376 // immune system process // inferred from electronic annotation///0002455 // humoral immune response m
NM_134102///XM_00	0.0311144		0.0477859	0.03111	1.15	-1.07	1.07	-1.43	*Pla1a*	phospholipase A1 member A	0006629 // lipid metabolic process // inferred from electronic annotation///0016042 // lipid catabolic process // inferr
NM_009791///XM_00	0.0184453			0.01845	-1.32	-1.22	1.12	-1.44	*Aspm*	asp (abnormal spindle)-like, microcephaly associated (Drosophila)	0001764 // neuron migration // inferred from genetic interaction///0001764 // neuron migration // inferred from mut
NM_001308327///NM_001308329,		0.0079678		0.00797	1.13	-1.10	-1.15	-1.45	*Enpp1*	ectonucleotide pyrophosphatase/phosphodiesterase 1	0006091 // generation of precursor metabolites and energy // not recorded///0006796 // phosphate-containing comp
NM_001159564///NM_021359///X		0.0385212		0.03852	1.35	1.58	1.44	-1.45	*Itgb6*	integrin beta 6	0006954 // inflammatory response // inferred from mutant phenotype///0007155 // cell adhesion // inferred from ele
NM_009610		0.0093985	0.037104	0.0094	-1.14	1.42	1.19	-1.46	*Actg2*	actin, gamma 2, smooth muscle, enteric	0010628 // positive regulation of gene expression // inferred from sequence or structural similarity///0090131 // mese
NM_026014	0.0059587			0.00596	1.24	1.12	1.00	-1.46	*Cdt1*	chromatin licensing and DNA replication factor 1	0000076 // DNA replication checkpoint // inferred from direct assay///0000076 // DNA replication checkpoint // not r
NM_013811///XM_006523588///XI		0.0173194		0.01732	1.05	-1.06	-1.04	-1.47	*Dnah8*	dynein, axonemal, heavy chain 8	0007018 // microtubule-based movement // inferred from electronic annotation///0008152 // metabolic process // ir
NM_001289780///NM	0.0351398	0.0019182		0.00192	1.05	1.21	1.04	-1.47	*Pus7*	pseudouridylate synthase 7 homolog (S. cerevisiae)	0001522 // pseudouridine synthesis // inferred from electronic annotation///0001522 // pseudouridine synthesis // ---
NM_010721	0.0280595			0.02806	1.10	1.16	1.03	-1.47	*Lmnb1*	lamin B1	0031662 // positive regulation of cyclin-dependent protein serine/threonine kinase activity involved in G2/M transitior
NM_013462///XM_006508987///XI		0.0337532		0.03375	1.05	-1.40	-1.17	-1.48	*Adrb3*	adrenergic receptor, beta 3	0002024 // diet induced thermogenesis // inferred from genetic interaction///0002025 // vasodilation by norepinephr
NM_001308535///NM	0.0404131			0.04041	1.20	1.12	1.15	-1.49	*Il2rg*	interleukin 2 receptor, gamma chain	0010468 // regulation of gene expression // inferred from mutant phenotype///0010628 // positive regulation of gene
NM_010516	4.41E-04			4.41E-04	2.94	1.10	1.08	-1.49	*Cyr61*	cysteine rich protein 61	0001558 // regulation of cell growth // inferred from electronic annotation///0001649 // osteoblast differentiation // i
NM_033075///XR_871926///XR_87		0.0498891		0.04989	-1.11	1.38	1.50	-1.51	*D17H6S56E-5*	DNA segment, Chr 17, human D6S56E 5	0019058 // viral life cycle // inferred from electronic annotation
NM_001033217///XM_006520264/		0.0161053		0.01611	1.22	-1.28	1.24	-1.54	*Prickle1*	prickle homolog 1 (Drosophila)	0001843 // neural tube closure // not recorded///0006606 // protein import into nucleus // not recorded///0031398 /
NM_001159299///NM	0.0073735			0.00737	1.45	1.53	1.02	-1.59	*Itih4*	inter alpha-trypsin inhibitor, heavy chain 4	0006953 // acute-phase response // inferred from electronic annotation///0010466 // negative regulation of peptidas
NM_019568///XM_00	0.039746			0.03975	1.84	1.60	1.11	-1.65	*Cxcl14*	chemokine (C-X-C motif) ligand 14	0006955 // immune response // inferred from electronic annotation///0045662 // negative regulation of myoblast dif
NM_010790///XM_006537640		0.0169902		0.01699	1.16	1.28	1.07	-1.68	*Melk*	maternal embryonic leucine zipper kinase	0006468 // protein phosphorylation // inferred from electronic annotation///0006915 // apoptotic process // not reco
NM_172505///NM_19	0.0072999			0.0073	1.14	1.46	1.45	-1.69	*Hjurp*	Holliday junction recognition protein	0007049 // cell cycle // inferred from electronic annotation///0007059 // chromosome segregation // not recorded//
NM_010474		0.0356842		0.03568	1.36	-1.38	1.15	-1.73	*Hs3st1*	heparan sulfate (glucosamine) 3-O-sulfotransferase 1	0008152 // metabolic process // inferred from electronic annotation
NM_011607///XM_00	0.0087337	0.0063502		0.00635	1.30	1.57	1.11	-1.79	*Tnc*	tenascin C	0001649 // osteoblast differentiation // not recorded///0007155 // cell adhesion // inferred from electronic annotatio
NM_011121		0.0320034		0.032	1.03	-1.04	1.04	-1.80	*Plk1*	polo-like kinase 1	0000070 // mitotic sister chromatid segregation // not recorded///0000086 // G2/M transition of mitotic cell cycle // r
NM_010766		0.0334347		0.03343	1.21	-1.26	-1.04	-1.86	*Marco*	macrophage receptor with collagenous structure	0002376 // immune system process // inferred from electronic annotation///0006897 // endocytosis // inferred from
NM_010104		0.01573		0.01573	-1.17	1.25	-1.14	-1.96	*Edn1*	endothelin 1	0000122 // negative regulation of transcription from RNA polymerase II promoter // not recorded///0001501 // skelet
NM_010809		0.0118081	0.0270064	0.01181	-1.37	1.03	-1.19	-1.97	*Mmp3*	matrix metallopeptidase 3	0006508 // proteolysis // inferred from mutant phenotype///0006508 // proteolysis // not recorded///0010727 // neg
NM_009364///XM_00	0.012514	0.017611		0.01761	1.72	1.30	1.02	-2.02	*Tfpi2*	tissue factor pathway inhibitor 2	0007596 // blood coagulation // inferred from electronic annotation///0007599 // hemostasis // inferred from electro
NM_010295///XM_006510812///XI		0.0441905	0.0416133	0.04419	1.17	1.29	1.18	-2.38	*Gclc*	glutamate-cysteine ligase, catalytic subunit	0006534 // cysteine metabolic process // not recorded///0006536 // glutamate metabolic process // not recorded///
NM_001177544///NM	0.0228937			0.02289	1.48	1.32	-1.20	-2.42	*Hist1h2ao///Hist1h2ap*	histone cluster 1, H2ao///histone cluster 1, H2ap	0006325 // chromatin organization // not recorded///0006342 // chromatin silencing // not recorded///0008285 // ne
NM_009264	0.0042823			0.00428	2.18	1.35	-1.13	-2.52	*Sprr1a*	small proline-rich protein 1A	0018149 // peptide cross-linking // not recorded///0030216 // keratinocyte differentiation // not recorded///0031424
NM_025748///XM_01	0.0136061			0.01361	1.09	-1.05	-1.08	-2.63	*Adat2*	adenosine deaminase, tRNA-specific 2	0002100 // tRNA wobble adenosine to inosine editing // inferred from electronic annotation///0006400 // tRNA modif
NM_001131054///NM_013917///X		0.0144328	2.02E-04	0.01443	-4.12	-3.09	-1.84	-2.81	*Pttg1*	pituitary tumor-transforming gene 1	0001558 // regulation of cell growth // not recorded///0006281 // DNA repair // inferred from electronic annotation//
NM_028216			0.0013	4.99E-04	1.06	-1.09	-1.17	-10.87	*Psca*	prostate stem cell antigen	0016020 // membrane // traceable author statement///0070062 // extracellular exosome // not recorded

Table S8. Common genes altered by O3 in Tnfr-, Tlr4-, and Nfkb1-dependent manner.

Venn Diagram analysis of Tnfr-dependent genes (n=2180, pooled from 24-72 hr O3), Tlr4-dependent genes (n=2320, pooled from 24- and 48-hr O3; Bauer et al. 2011 Env Health Perspect, GEO#GSE20715)and Nfkb1-dependent genes (n=2397, 48-hr O3).

Tlr4 mutant strain-C3H/HeJ (HeJ), Tlr4 normal strain-C3H/HeOuJ (OuJ).

Fold difference (FD); positive-higher in KO or HeJ-O3, negative-lower in KO or HeJ-O3, compared to corresponding WT or OuJ-O3.

RefSeq Transcript ID	Tnfr FD 24 hr (	Tnfr FD 48 hr	Tnfr FD 72 hr	Tlr4 FD 24 hr (	Tlr4 FD 48 hr	Nfkb1 FD 4	Gene Symbol	Gene Title	Gene Ontology Biological Process
NM_001252332///NM_0012	1.02	1.18	1.16	-1.05	-1.02	-1.07	***Sept8***	septin 8	0000166 // nucleotide binding // inferred from electronic annotation///0005515 // protein bin
NM_001177945///NM_0011	-1.10	-1.01	1.02	-1.12	-1.01	1.19	***Aamdc***	adipogenesis associated Mth938 domain containing	0043066 // negative regulation of apoptotic process // inferred from mutant phenotype///004
NM_001038587///NM_0011	1.31	1.19	1.09	1.06	-1.24	1.12	***Adar***	adenosine deaminase, RNA-specific	0001649 // osteoblast differentiation // inferred from mutant phenotype///0001701 // in uter
NM_025748///XM_0112432	1.09	-1.05	-1.08	1.08	1.12	-2.63	***Adat2***	adenosine deaminase, tRNA-specific 2	0002100 // tRNA wobble adenosine to inosine editing // inferred from electronic annotation//
NM_007440///XM_0112486	-1.04	-1.25	-1.01	1.43	1.00	1.42	***Alox12***	arachidonate 12-lipoxygenase	0006629 // lipid metabolic process // inferred from electronic annotation///0006631 // fatty a
NM_001286040///NM_0012	-1.15	-1.11	1.00	1.04	-1.03	-1.11	***Anks1***	ankyrin repeat and SAM domain containing 1	0006929 // substrate-dependent cell migration // inferred from direct assay///0016322 // neu
NM_018829///XM_0065192	1.08	1.22	1.15	-1.04	1.03	-1.15	***Ap3m1***	adaptor-related protein complex 3, mu 1 subunit	0006810 // transport // inferred from electronic annotation///0006886 // intracellular protein
NM_001314013///NM_0530	1.06	1.10	1.15	-1.05	1.13	1.07	***Atg5***	autophagy related 5	0000045 // autophagosome assembly // inferred from mutant phenotype///0000422 // mitop
NM_007591	-1.11	1.25	1.22	-1.38	1.05	-1.11	***Calr***	calreticulin	0000122 // negative regulation of transcription from RNA polymerase II promoter // not recor
NM_001080126///NM_0012	1.24	1.15	1.17	1.06	1.15	-1.13	***Casp8***	caspase 8	0001525 // angiogenesis // inferred from mutant phenotype///0001841 // neural tube format
NM_001159738///NM_0169	-1.33	-1.18	-1.22	-1.04	1.53	1.56	***Ccl20***	chemokine (C-C motif) ligand 20	0002548 // monocyte chemotaxis // ---///0006935 // chemotaxis // not recorded///0006954 //
NM_001111099///NM_0076	-1.83	-1.03	-1.41	2.20	1.26	1.78	***Cdkn1a***	cyclin-dependent kinase inhibitor 1A (P21)	0000079 // regulation of cyclin-dependent protein serine/threonine kinase activity // inferred
NM_016675///XM_0065284	1.14	1.00	-1.01	1.04	1.16	1.25	***Cldn2***	claudin 2	0016338 // calcium-independent cell-cell adhesion via plasma membrane cell-adhesion molec
NM_001252479///NM_1339	1.19	-1.03	-1.19	1.05	1.20	1.24	***Cmtm7***	CKLF-like MARVEL transmembrane domain containing 7	0002337 // B-1a B cell differentiation // inferred from mutant phenotype///0006935 // chemo
NM_001037859///NM_0077	1.03	-1.12	-1.02	-1.10	-1.10	1.22	***Csf1r***	colony stimulating factor 1 receptor	0001934 // positive regulation of protein phosphorylation // not recorded///0002376 // immu
NM_001136086///NM_0012	-1.07	1.16	1.10	1.04	1.23	-1.31	***Dpysl3***	dihydropyrimidinase-like 3	0007399 // nervous system development // inferred from direct assay///0008152 // metabolic
NM_010104	-1.17	1.25	-1.14	1.62	1.18	-1.96	***Edn1***	endothelin 1	0000122 // negative regulation of transcription from RNA polymerase II promoter // not recor
NM_010485///XM_0065086	-1.02	1.20	1.15	-1.11	-1.03	-1.10	***Elavl1***	ELAV (embryonic lethal, abnormal vision)-like 1 (Hu antigen R)	0006417 // regulation of translation // not recorded///0045727 // positive regulation of transl
NM_001136077///NM_0012	-1.06	-1.00	1.04	1.05	-1.16	1.10	***Enpp2***	ectonucleotide pyrophosphatase/phosphodiesterase 2	0001953 // negative regulation of cell-matrix adhesion // not recorded///0006629 // lipid met
NM_001081225///NM_1726	-1.40	-1.08	1.12	-1.24	-1.20	-1.15	***Fam178a***	family with sequence similarity 178, member A	0006281 // DNA repair // inferred from electronic annotation///0006974 // cellular response t
NM_025912///XM_0065000	1.02	-1.01	-1.09	1.08	1.17	1.10	***Fam210b***	family with sequence similarity 210, member B	0016020 // membrane // inferred from electronic annotation///0016021 // integral componen
NM_008086	-1.00	-1.12	-1.06	-1.17	-1.04	1.10	***Gas1***	growth arrest specific 1	0002053 // positive regulation of mesenchymal cell proliferation // inferred from mutant phen
NM_001039129///NM_0011	1.53	1.18	1.20	1.10	-1.18	-1.25	***Hnrnpa1***	heterogeneous nuclear ribonucleoprotein A1 pseudogene///predicted gene 5803///heterogeneous nuclea	0000380 // alternative mRNA splicing, via spliceosome // inferred from direct assay///000639
XM_011238903///XM_01123	2.61	3.75	3.71	1.18	-1.42	1.12	***Gm37416///Gm39743///Gm***	predicted gene, 37416///predicted gene, 39743///predicted gene, 40611///predicted gene, 40814///predi	0006278 // RNA-dependent DNA replication // inferred from electronic annotation///0008152
NM_001080707///NM_0011	-1.21	-1.10	1.62	-1.30	-1.49	1.14	***Gpr155***	G protein-coupled receptor 155	0035556 // intracellular signal transduction // inferred from electronic annotation///0050890
NM_153071///XM_0112431	-1.10	-1.02	-1.07	-1.14	-1.43	1.33	***Gprc6a***	G protein-coupled receptor, family C, group 6, member A	0007165 // signal transduction // inferred from electronic annotation///0007186 // G-protein
NM_010344	1.29	1.07	1.10	1.07	1.15	1.16	***Gsr***	glutathione reductase	0006749 // glutathione metabolic process // inferred by curator///0006749 // glutathione met
NM_026113///XM_0065128	1.14	1.30	1.08	-1.08	1.27	-1.02	***Gtf3c6***	general transcription factor IIIC, polypeptide 6, alpha	0006351 // transcription, DNA-templated // not recorded///0006383 // transcription from RN
NM_022331///XM_0065312	-1.56	1.12	-1.09	-1.22	-1.48	1.21	***Herpud1***	homocysteine-inducible, endoplasmic reticulum stress-inducible, ubiquitin-like domain member 1	0006511 // ubiquitin-dependent protein catabolic process // inferred from direct assay///0006
NM_019447///XM_0065040	-1.17	-1.16	1.03	1.09	-1.20	-1.07	***Hgfac***	hepatocyte growth factor activator	0006508 // proteolysis // inferred from electronic annotation
NM_010474	1.36	-1.38	1.15	1.36	1.39	-1.73	***Hs3st1***	heparan sulfate (glucosamine) 3-O-sulfotransferase 1	0008152 // metabolic process // inferred from electronic annotation
NM_172015///XM_0065168	-1.05	1.08	1.02	-1.12	1.06	-1.10	***Iars***	isoleucine-tRNA synthetase	0001649 // osteoblast differentiation // not recorded///0006412 // translation // inferred from
NM_010501///XM_0112471	1.29	-1.08	1.28	-1.13	1.07	1.41	***Ifit3***	interferon-induced protein with tetratricopeptide repeats 3	0002376 // immune system process // inferred from electronic annotation///0008285 // negat
NM_001308535///NM_0135	1.20	1.12	1.15	-1.06	1.02	-1.49	***Il2rg***	interleukin 2 receptor, gamma chain	0010468 // regulation of gene expression // inferred from mutant phenotype///0010628 // po
NM_008403///XM_0065149	1.14	1.08	-1.02	1.11	1.14	-1.01	***Itgb1bp1***	integrin beta 1 binding protein 1	0001525 // angiogenesis // inferred from electronic annotation///0002043 // blood vessel end
NM_001159299///NM_0012	1.45	1.53	1.02	-1.19	1.14	-1.59	***Itih4***	inter alpha-trypsin inhibitor, heavy chain 4	0006953 // acute-phase response // inferred from electronic annotation///0010466 // negativ
NM_008409	1.13	1.11	1.12	1.04	-1.03	-1.03	***Itm2a***	integral membrane protein 2A	0005515 // protein binding // inferred from electronic annotation
NM_031248	1.06	1.12	1.09	-1.03	1.14	1.05	***Lamtor2***	late endosomal/lysosomal adaptor, MAPK and MTOR activator 2	0000186 // activation of MAPKK activity // inferred from direct assay///0016049 // cell growth
NM_008491	1.67	1.43	1.37	-1.10	1.54	-1.20	***Lcn2***	lipocalin 2	0002376 // immune system process // inferred from electronic annotation///0006810 // trans
NM_025828	-1.02	1.16	1.09	-1.06	-1.13	-1.06	***Lman2***	lectin, mannose-binding 2	0006810 // transport // inferred from electronic annotation///0006890 // retrograde vesicle-m
NM_001164885///NM_0228	-1.24	-1.06	1.16	1.02	-1.18	1.27	***Lpin2***	lipin 2	0006351 // transcription, DNA-templated // inferred from electronic annotation///0006355 //
NM_010766	1.21	-1.26	-1.04	2.27	4.07	-1.86	***Marco***	macrophage receptor with collagenous structure	0002376 // immune system process // inferred from electronic annotation///0006897 // endo
NM_001081392///NM_1338	-1.03	1.02	-1.05	-1.08	1.10	-1.05	***Mdn1***	midasin homolog (yeast)	0000027 // ribosomal large subunit assembly // inferred from electronic annotation///00081
NM_025578	-1.02	1.12	1.00	-1.04	1.12	-1.08	***Mrps25***	mitochondrial ribosomal protein S25	0032543 // mitochondrial translation // inferred by curator
NM_001008542///NM_0010	1.04	-1.11	1.07	1.09	-1.13	-1.07	***Mxi1***	MAX interactor 1, dimerization protein	0000122 // negative regulation of transcription from RNA polymerase II promoter // inferred f
NM_026526///XM_0065194	1.06	1.11	-1.02	-1.05	1.07	-1.02	***N6amt2***	N-6 adenine-specific DNA methyltransferase 2 (putative)	0032259 // methylation // inferred from electronic annotation
NM_026554	-1.01	1.26	1.16	-1.22	1.10	-1.12	***Ncbp2***	nuclear cap binding protein subunit 2	0000184 // nuclear-transcribed mRNA catabolic process, nonsense-mediated decay // not rec
NM_001286037///NM_0108	1.45	1.09	-1.14	-1.06	-1.38	1.19	***Ncf1***	neutrophil cytosolic factor 1	0001878 // response to yeast // inferred from mutant phenotype///0001909 // leukocyte med
NM_010880///XM_0065292	1.44	1.29	1.45	1.02	1.04	-1.19	***Ncl***	nucleolin	0001525 // angiogenesis // not recorded///0006897 // endocytosis // not recorded///0007283
NM_134010///XM_0065130	1.09	1.12	1.08	1.06	1.10	-1.10	***Nup107***	nucleoporin 107	0000973 // posttranscriptional tethering of RNA polymerase II gene DNA at nuclear periphery
NM_011119	1.08	1.01	1.17	-1.02	1.12	-1.12	***Pa2g4***	proliferation-associated 2G4	0006351 // transcription, DNA-templated // inferred from electronic annotation///0006355 //
NM_172924///XM_0065111	1.09	1.19	-1.05	1.02	-1.15	-1.37	***Peak1***	pseudopodium-enriched atypical kinase 1	0006468 // protein phosphorylation // not recorded///0016310 // phosphorylation // inferred
NM_011072	1.26	1.05	-1.13	-1.17	1.17	-1.07	***Pfn1***	profilin 1	0001843 // neural tube closure // inferred from genetic interaction///0006357 // regulation of
NM_133942///XM_0065071	-1.14	1.11	1.07	-1.02	1.20	-1.11	***Plekha1***	pleckstrin homology domain containing, family A (phosphoinositide binding specific) member 1	0001553 // luteinization // inferred from genetic interaction///0007283 // spermatogenesis //
NM_008947	1.11	1.36	1.42	1.02	1.32	-1.05	***Psmc1***	protease (prosome, macropain) 26S subunit, ATPase 1	0030163 // protein catabolic process // inferred from electronic annotation///0030433 // ER-a
NM_011189	1.22	1.05	1.31	-1.06	1.17	1.22	***Psme1***	proteasome (prosome, macropain) activator subunit 1 (PA28 alpha)	0002479 // antigen processing and presentation of exogenous peptide antigen via MHC class I
NM_001085492///XM_0065	-1.03	1.03	1.34	-1.24	-1.40	-1.15	***Rere***	arginine glutamic acid dipeptide (RE) repeats	0000122 // negative regulation of transcription from RNA polymerase II promoter // inferred f
NM_133933	-1.06	1.18	-1.12	-1.05	1.27	-1.10	***Rpn1***	ribophorin I	0006486 // protein glycosylation // inferred from electronic annotation///0018279 // protein N
NM_001166506///NM_0011	-1.18	-1.02	1.06	-1.01	-1.07	-1.03	***Sec14l1***	SEC14-like lipid binding 1	0006810 // transport // inferred from electronic annotation
NM_001164059///NM_0113	1.72	-1.04	-1.05	1.10	-1.18	1.91	***Sell***	selectin, lymphocyte	0007155 // cell adhesion // inferred from electronic annotation///0033198 // response to ATP
NM_009151///XM_0065302	1.21	-1.01	-1.06	1.23	-1.02	1.35	***Selplg***	selectin, platelet (p-selectin) ligand	0007155 // cell adhesion // inferred from electronic annotation///0050901 // leukocyte tether
NM_145156///XM_0065270	1.12	-1.09	-1.11	1.17	1.05	1.10	***Slc25a28***	solute carrier family 25, member 28	0006810 // transport // inferred from electronic annotation///0006811 // ion transport // infe
NM_021535	1.22	1.24	1.15	1.03	1.12	-1.06	***Smu1***	smu-1 suppressor of mec-8 and unc-52 homolog (C. elegans)	0005634 // nucleus // inferred from electronic annotation///0005737 // cytoplasm // inferred
NM_144918	1.14	1.10	-1.00	-1.06	1.13	-1.10	***Smyd5***	SET and MYND domain containing 5	0032259 // methylation // inferred from electronic annotation
NM_001110310///NM_0011	1.03	1.08	1.06	-1.08	1.07	-1.07	***Snx12***	sorting nexin 12	0006810 // transport // inferred from electronic annotation///0006897 // endocytosis // not r
NM_009264	2.18	1.35	-1.13	1.87	2.22	-2.52	***Sprr1a***	small proline-rich protein 1A	0018149 // peptide cross-linking // not recorded///0030216 // keratinocyte differentiation // r
NM_011512///XM_0064978	1.10	1.05	1.18	1.00	1.12	1.03	***Surf4***	surfeit gene 4	0007030 // Golgi organization // not recorded///0010638 // positive regulation of organelle or
NM_001311156///NM_1339	1.10	-1.02	1.05	1.09	1.15	1.16	***Tcta***	T cell leukemia translocation altered gene	0016020 // membrane // inferred from electronic annotation///0016021 // integral componen
NM_017376///NM_153484//	1.11	1.11	1.74	-1.43	-1.66	-1.12	***Tef***	thyrotroph embryonic factor	0006351 // transcription, DNA-templated // inferred from electronic annotation///0006355 //
NM_009364///XM_0065050	1.72	1.30	1.02	-1.03	1.20	-2.02	***Tfpi2***	tissue factor pathway inhibitor 2	0007596 // blood coagulation // inferred from electronic annotation///0007599 // hemostasis
NM_011607///XM_0065377	1.30	1.57	1.11	-1.35	1.70	-1.79	***Tnc***	tenascin C	0001649 // osteoblast differentiation // not recorded///0007155 // cell adhesion // inferred fr
NM_009396///XM_0065157	1.09	-1.20	1.02	1.10	1.32	1.40	***Tnfaip2***	tumor necrosis factor, alpha-induced protein 2	0001525 // angiogenesis // inferred from electronic annotation///0006887 // exocytosis // not
NM_011653	-1.00	1.09	-1.01	1.03	1.24	-1.06	***Tuba1a***	tubulin, alpha 1A	0007017 // microtubule-based process // inferred from electronic annotation///0008152 // m
